# Gene Expression over Time during Cell Transformation Due to Non-Genotoxic Carcinogen Treatment of Bhas 42 Cells

**DOI:** 10.3390/ijms23063216

**Published:** 2022-03-16

**Authors:** Kiyomi Ohmori, Asuka Kamei, Yuki Watanabe, Keiko Abe

**Affiliations:** 1 Chemical Division, Kanagawa Prefectural Institute of Public Health, Chigasaki 2530087, Japan; 2 Research Initiatives and Promotion Organization, Yokohama National University, Yokohama 2408501, Japan; 3 Group for Food Functionality Assessment, Kanagawa Institute of Industrial Science and Technology, Kawasaki 2100821, Japan; kamei@kistec.jp (A.K.); aka7308@mail.ecc.u-tokyo.ac.jp (K.A.); 4 Health and Anti-Aging Project, Kanagawa Academy of Science and Technology, Kawasaki 2130012, Japan; onuki.yuki0205@mail.u-tokyo.ac.jp; 5 Department of Applied Biological Chemistry, Graduate School of Agricultural and Life Sciences, The University of Tokyo, Tokyo 1138657, Japan

**Keywords:** Bhas 42 cells, cell-transformation assay, non-genotoxic carcinogen, 12-*O*-tetradecanoylphorbol-13-acetate, transcriptomics, hallmarks of cancer, over time

## Abstract

The Bhas 42 cell transformation assay (Bhas 42 CTA) is the first Organization for Economic Cooperation and Development (OECD)-certificated method used as a specific tool for the detection of the cell-transformation potential of tumor-promoting compounds, including non-genotoxic carcinogens (NGTxCs), as separate from genotoxic carcinogens. This assay offers the great advantage of enabling the phenotypic detection of oncotransformation. A key benefit of using the Bhas 42 CTA in the study of the cell-transformation mechanisms of tumor-promoting compounds, including non-genotoxic carcinogens, is that the cell-transformation potential of the chemical can be detected directly without treatment with a tumor-initiating compound since Bhas 42 cell line was established by transfecting the v-Ha-*ras* gene into a mouse fibroblast cloned cell line. Here, we analyzed the gene expression over time, using DNA microarrays, in Bhas 42 cells treated with the tumor-promoting compound 12-*O*-tetradecanoylphorbol-13-acetate (TPA), and NGTxC, with a total of three repeat experiments. This is the first paper to report on gene expression over time during the process of cell transformation with only a tumor-promoting compound. Pathways that were activated or inactivated during the process of cell transformation in the Bhas 42 cells treated with TPA were related not only directly to *RAS* but also to various pathways in the hallmarks of cancer.

## 1. Introduction

Approximately 20% of known carcinogenic substances score negatively in the existing genotoxicity assays [1]. Non-genotoxic mechanisms, which are at least initially independent of direct DNA damage, play a causal role in carcinogenesis [2], and many NGTxCs are likely to be tumor-promoting compounds. Therefore, assays that predict the tumor-promoting activity of chemicals are needed. Using Bhas 42 cells, Ohmori et al. developed a short-term cell-transformation assay to detect the cell-transformation activity of tumor-promoting NGTxCs, an assay that has been used to test the ability of Bhas 42 CTA to promote tumor [3]. The Bhas 42 cell line was established by transfecting the v-Ha-*ras* oncogene into a mouse fibroblast cloned cell line [4]. To increase the understanding of this method, a collaborative study of the promotion test for Bhas 42 CTA was conducted among 14 laboratories, which revealed high rates of consistency and sensitivity for the Bhas CTA, as well as important factors regarding the reproducibility of the test method [5]. Thereby, this collaborative effort resulted in a highly robust framework for the protocol, which is suitable for application according to the OECD test guidelines. Next, a test for the prediction of the genotoxicity of chemicals was added [6]. The characteristics and performance of Bhas 42 CTA for predicting chemical carcinogenicity, which includes non-genotoxic carcinogenicity and genotoxic carcinogenicity, were verified using two protocols, including an “initiation test” for the detection of genotoxic carcinogens and a “promotion test” for the detection of NGTxC [7,8,9], and the Bhas 42 CTA was authorized as a cell-transformation assay method in the 2016 OECD guidance document [10]. The Bhas 42 CTA is the first internationally recognized in vitro assay for the detection of NGTxC as distinguished from genotoxic carcinogens. However, OECD-member countries have been making efforts to expand the use of alternative methods for assessing chemicals, and the OECD has been developing integrated approaches for testing and assessment (IATAs) [11]. IATAs are pragmatic, science-based approaches for chemical-hazard or risk characterization that rely on an integrated analysis of existing information via a weight-of-evidence assessment coupled with the generation of new information using testing strategies. Furthermore, the OECD has been developing an IATA for non-genotoxic carcinogenic chemical substances [12,13]. Therefore, demonstrating the mode of action of NGTxC in Bhas 42 cells, and providing evidence for the involvement of the chemically induced cell transformation of Bhas 42 cells in tumorigenesis and carcinogenesis, are important. The greatest advantage of using Bhas 42 CTA for studying the mechanism of NGTxC is that the mechanism of cell transformation induced by NGTxC can be investigated directly, without treatment using genotoxic compounds. 12-*O*-tetradecanoylphorbol-13-acetate (TPA), the test compound in this study, is a typical tumor-promoting compound used as a positive-control compound in the promotion test of OECD GD No. 231. Regarding the tumorigenicity of TPA, skin tumors were induced via repeated treatment with TPA alone instead of using two steps in hairless mice [14]. Moreover, TPA was found to be negative in the genotoxicity test used in that study (the Ames test) [15]. Thus, to demonstrate the mode of action of NGTxC in Bhas 42 CTA via transcriptome analysis, we analyzed the gene expression of Bhas 42 cells treated over time with TPA, which is both a typical tumor-promoting compound and an NGTxC. Three biological replicates from the independent thawing of stock cells in Bhas 42 CTA were used for the microarray analysis. In this report, Bhas 42 CTA was shown to be an assay that provides much information via data, the reproducibility of which was proved by statistically significant increases and decreases in gene expression, including variability with repeated experiments, in mechanism analyses via transcriptomics.

## 2. Results

### 2.1. Morphological Changes and Focus Formation by Bhas 42 Cells at the Tumor-Promotion Stage of the Cell-Transformation Assay

**Figure 1:**
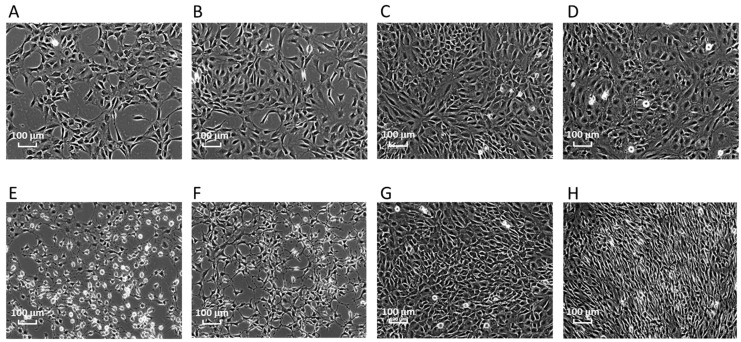
Morphological changes of Bhas 42 cells after treatment with TPA. (**A**) Solvent control, 1 h; (**B**) solvent control, 6 h; (**C**) solvent control, 24 h; (**D**) solvent control, 8 days; (**E**) TPA, 1 h; (**F**) TPA, 6 h; (**G**) TPA, 24 h; (**H**) TPA, 8 days.

Bhas 42 cells treated with TPA or dimethylsulfoxide (DMSO) (solvent-only control) for 1, 6, or 24 h, or 8 days were evaluated morphologically using light microscopy (Figure 1). The treatment of Bhas 42 cells with TPA for 1 or 6 h induced significant changes in cell shape. However, the difference in cell morphology between the TPA-treated group and the solvent-control group disappeared, and the cell density of Bhas 42 cells treated with TPA for 24 h was further increased compared to that of those treated with DMSO. The treatment of Bhas 42 cells with TPA for 8 days led to morphologic changes on Day 12 of culture that were consistent with early-stage cell transformation, including the dense multi-layering of cells with randomly orientated cells at the edges. On Day 21, TPA (5 ng/mL) induced 16 ± 4 (mean ± standard deviation) transformed foci per well, whereas the solvent control yielded 5 ± 3 transformed foci per well. The statistical significance of the TPA-induced transformation frequency was evaluated using a one-sided Student’s *t*-test (*p* < 0.05, upper-sided) depending on the results of the F-test for homoscedasticity. The cell transformation was judged to be significantly induced by TPA (5 ng/mL).

### 2.2. Quantification of DNA Microarray Data and Detection of Differentially Expressed Genes

**Figure 2:**
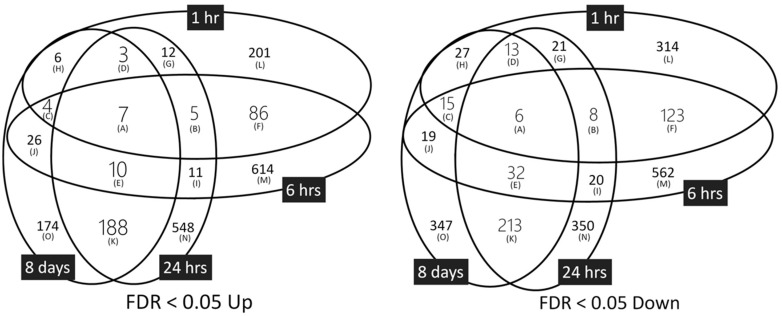
Genes showing up (Up)-or down (Down)-regulation in response to TPA treatment. FDR, false-discovery rate.

To determine the gene-expression profiles of TPA-treated Bhas 42 cells, three biological replicates were prepared for each TPA treatment time (i.e., 1, 6, and 24 h, and 8 days). We applied a distribution-free weighted (DFW) method for the quantification of the raw data (Affymetrix CEL files). Hierarchical clustering analysis revealed that each experimental group formed its own cluster (Figure S1), indicating that the gene-expression patterns of the groups differed between the TPA treatment times. For each TPA treatment time, the DMSO group was separated from the TPA group. Furthermore, the analysis revealed that the 1 and 6 h-treatment groups formed a large cluster, as did the 24 h and 8-day TPA treatment groups. Applying this information to our microarray data, we then selected the up- and down-regulated probes and genes in each of the four TPA treatment groups. When the false-discovery rate (FDR) was less than 0.05, the number of genes up-regulated by TPA treatment at 24 h was the greatest in the up-regulated genes, and the number down-regulated by TPA treatment at 6 h was the greatest in the down-regulated genes (Table S2). A total of 102 up-regulated genes and 152 down-regulated genes were common between the 1 and 6 h TPA treatment groups, and 208 up-regulated genes and 264 down-regulated genes were common between the 24 h and 8-day treatment groups (Figure 2 and Table S3). Seven genes—*ANGPTL4* (angiopoietin-like 4), *HMGA1* (high-mobility group AT-hook 1), *HMGA2* (high-mobility group AT-hook 2), *MPP6* (membrane protein, palmitoylated 6), *RBM3* (RNA-binding motif protein 3), *SLPIi* (secretory leukocyte peptidase inhibitor), and *ZWINT* (Zeste white 10 interactor)—were common among the up-regulated genes of all the TPA treatment groups. Six genes—*AKAP12* (A kinase (PRKA) anchor protein (gravin) 12), *MAF* (MAF1 homolog), *NFIB* (nuclear factor I/B), *PTN* (pleiotrophin), *THBS11* (thrombospondin 1), and *TBM1* (tropomyosin 1, alpha)—were common among the down-regulated genes in all the TPA treatment groups.

### 2.3. Gene Ontology (GO) Terms

**Figure 3:**
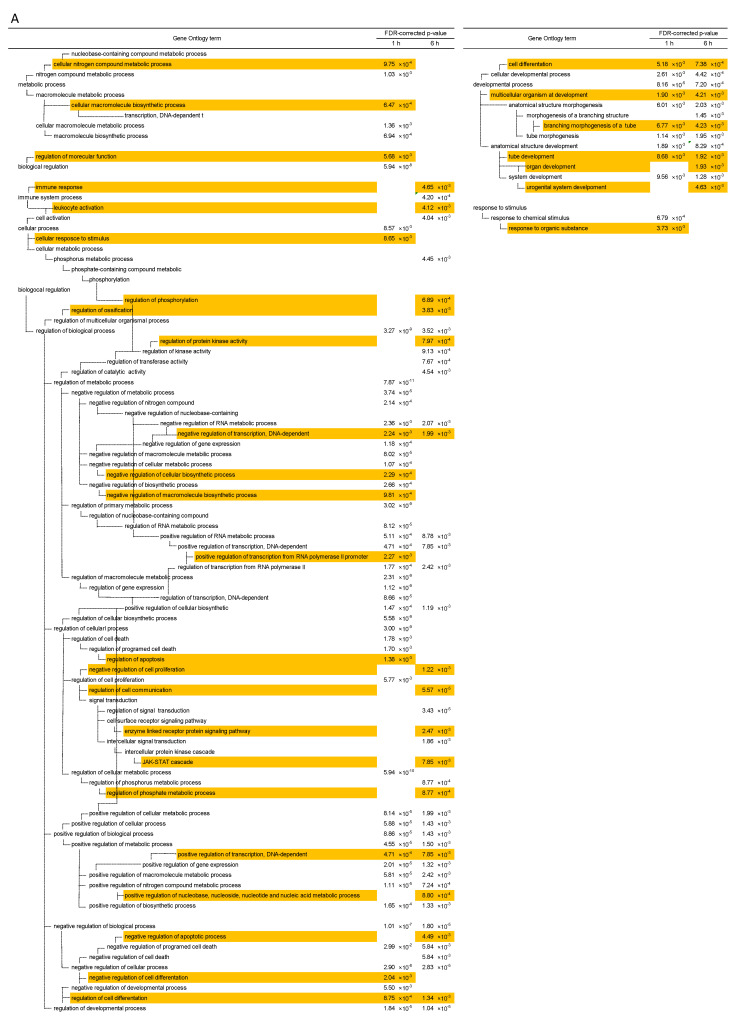

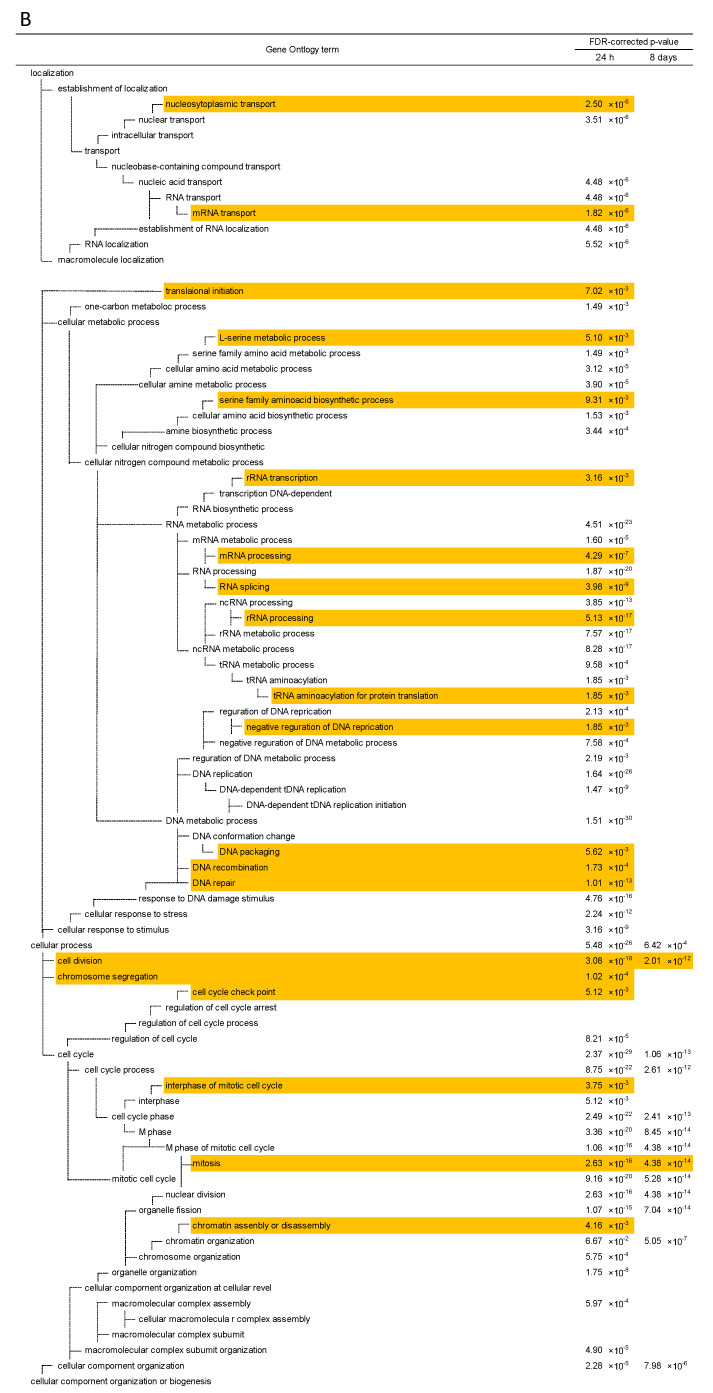
Gene Ontology terms for selected genes. The GO terms up-regulated due to TPA treatment for (**A**) 1 h and 6 h. Gene Ontology terms for selected genes (continued). The GO terms up-regulated due to TPA treatment for (**B**) 24 h and 8 days.

**Figure 4:**
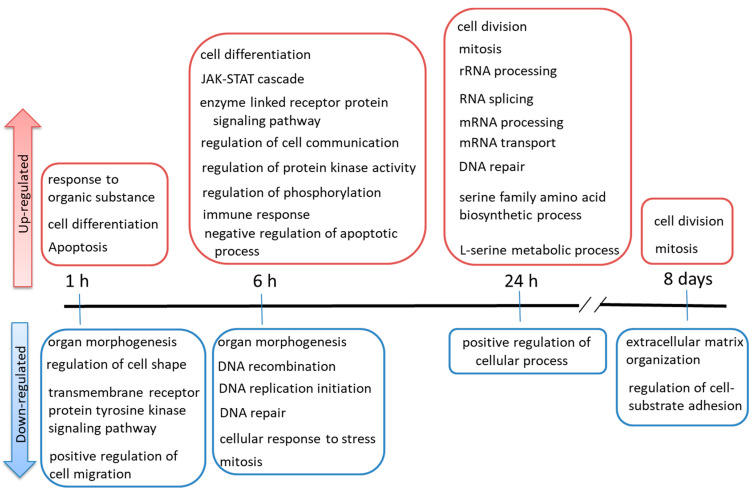
Summary of Gene Ontology terms in chronological order.

To identify GO terms that were over-represented among the selected genes, we used the online software Database for Annotation, Visualization, and Integrated Discovery (DAVID). Among the up-regulated genes, several GO terms common between the 1 and 6 h TPA treatments were not significantly enriched in the other treatment groups (Figure 3A). The GO terms that were common among the genes that were up-regulated by TPA treatment for 1 or 6 h were as follows: negative regulation of transcription DNA-dependent, positive regulation of transcription DNA-dependent, regulation of cell differentiation, cell differentiation, multicellular organism at development, branching morphogenesis of a tube, and tube development. The GO terms that were only significantly enriched in the group treated with TPA for 1 h consisted of regulation of apoptosis, cellular response to stimulus, response to organic substance, and negative regulation of cell differentiation. Those significantly enriched only in the group treated with TPA for 6 h were immune response, regulation of phosphorylation, regulation of protein kinase activity, enzyme-linked receptor protein signaling pathway, regulation of cell communication, *JAK-STAT* cascade, regulation of phosphate metabolic process, and nucleotide and nucleic acid metabolic process. The GO terms that were common between the genes that were up-regulated after TPA treatment for 24 h and 8 days (Figure 3B) were cell division and mitosis. The GO terms that were only significantly enriched in the group treated with TPA for 24 h were translational initiation, cell cycle checkpoint, and interphase of mitotic cell cycle. Moreover, all the GO terms associated with TPA treatment for 8 days were included among those related to TPA treatment for 24 h. The GO terms significantly enriched among the gene sets that were down-regulated by TPA treatments of different durations are summarized in Figure S4—they were not significantly involved in cancer. These are summarized in chronological order in Figure 4.

### 2.4. Pathway Analysis Using IPA Software

#### 2.4.1. Gene Expression Characteristics for 1 H TPA Treatment

**Figure 5:**
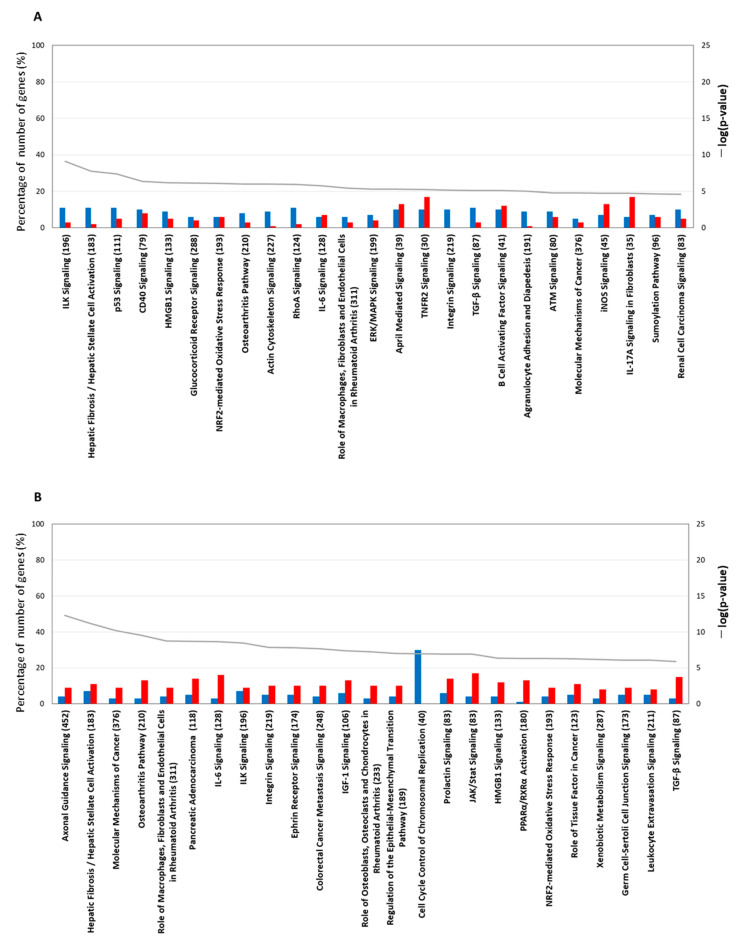

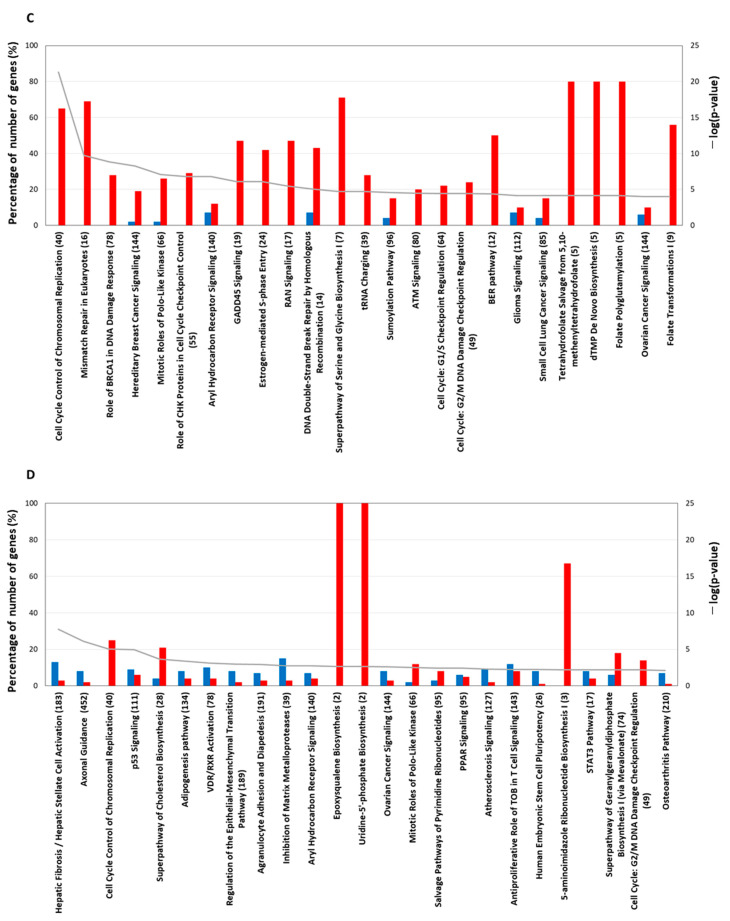
The top-25 canonical pathways. TPA treatment for (**A**) 1 h and (**B**) 6 h. The line graph shows a list of genes whose expression increased or decreased at each treatment time and the −log value (*p*-value) obtained by testing a list of genes of the canonical pathway via Fisher’s exact test. The bar graph shows the rate (%) of the number of genes that were up-regulated (red) and the number of genes that were down-regulated (blue) when the number of genes in a known pathway was 100%. The top-25 canonical pathways (continued). TPA treatment for (**C**) 24 h and (**D**) 8 days. Other explanations are the same as for the graphs of the 1 h treatment and the 6 h treatment.

In the pathway analysis of the gene-expression characteristics for the 1 h TPA treatment, the top-25 canonical pathways are listed in descending order of –log (*p*-value) (Figure 5A). The percentage of down-regulated genes was higher than that of those up-regulated in the top-10 pathways. Bhas 42 cells treated with TPA for 1 h showed morphological changes in which the cells became small and round-shaped. Gene expression related to significant changes in cell shape appeared as a decrease in filamentous actin *(F-ACTIN*), which is related to cell adhesion and integrin-linked kinase (*ILK*) signaling (Figure S5). Most genes related to signal transduction in the cytoplasm were inactivated in the pathway of the Molecular Mechanism of Cancer (Figure S6); however, the gene expression of *NF-κB*, *c-FOS*, *c-JUN*, *MDM2*, *AURORA-A*, *BBC*3, *BIM*, and *p21CIP1* was significantly increased. The increase in NF-κB expression was enhanced by insulin receptor substrate 1 (*IRS1*) and phosphatidylinositol-3 kinase in signal transduction related to cell survival. The gene expression of *c-FOS* and *c-JUN*, which are immediate early genes, was up-regulated in the nucleus without accompanying protein synthesis when the cells were subjected to various stimuli [16]. Thus, transcription was increased, but signaling from the cytoplasm was not. The gene expression of *MDM2* was also increased. *MDM2* directly inhibits its transcriptional activating ability by binding to *P53*, and indirectly ubiquitinates *P53* as ubiquitin E3 ligase, leading to degradation by the proteasome. CDK-interacting protein 1 (*p21CIP1*) is a cell-cycle-regulatory protein that interacts with cyclin-CDK2 and CDK4, inhibiting cell-cycle progression at G1 and genes expressed by stress responses related to transforming growth factor (TGF)-β1 [17]. However, the gene expression of TGF-β was decreased in Bhas 42 cells treated with TPA for 1 h. Although many pathways were inactivated by TPA, tumor necrosis factor receptor 2 (TNFR2) signaling was activated by the up-regulation of TNF receptor-associated factor 1 (TRAF1), which mediates anti-apoptotic signals from TNF receptors and is related to NF-kB (Figure S7). The mammalian target of the rapamycin (mTOR) pathway was also activated by the up-regulation of the gene expression of *PLD*, *IRS1*, *REDD1*, and *elF4E* (Figure S8). In the NRF2 oxidative stress response pathway, the up-regulation of the gene expression of Jun led to an increase in the gene expression of *NRF2*, and, furthermore, to an increase in the gene expression of *SQSTM1* and heme oxygenase 1 (*HO-1*), which are antioxidant proteins; GCLC, which is a detoxification factor; and HSP22/40/90, which is a stress-response protein (Figure S9). In the xenobiotic metabolism pathway, the gene expression of *CYP1A1* and *CYP1B1*, which are related to the aryl-hydrocarbon receptor (*AHR*) ligand, was increased (Figure S10). In addition, the integrin pathway and paxillin pathway, which are related to cell motility, were inactivated. At 1 h after TPA treatment, reactions to maintain homeostasis in response to stimulation by TPA were activated (Figure S11).

#### 2.4.2. Gene-Expression Characteristics of 6 H TPA Treatment

The distance between the cells at 6 h after TPA treatment was the same as that at 1 h after treatment. However, the cell morphology after 6 h showed the invasion of surrounding cells. We performed pathway analysis of the gene-expression characteristics of the 6 h treatment group, and the pathways are listed in descending order of –log (*p*-value) (Figure 5B). The −log (*p*-value) of axonal guidance signaling was the highest, and that for tubulin and nuclear factor of activated T-cells (*NFAT*), which is related to axon outgrowth, was up-regulated (Figure S12). On the other hand, paxillin (*PXN*), which is related to axon repulsion; Arp2/3; and G-actin-Profilin (*WASP*), which is related to cytoskeleton reorganization, were also up-regulated. In addition, semaphorins such as 4, 6, and 7A, which are involved in numerous functions including axon guidance and morphogenesis, were up-regulated. In ILK signaling, matrix metalloproteinase 9 (*MMP9*) (Figure S13), which is related to tissue inversion, and desmoplakin (DSP), which is related to desmosome assembly, were up-regulated. In the molecular mechanism of cancer pathway, which is the third pathway, the up-regulated expression of *RASGEF* and the up-regulated expression of *RAS* were linked; however, the up-regulated expression of *RASGAP* led to the suppression of *RAS* gene expression (Figure S14). Thus, the expression of *RAS* involves both up-regulation and down-regulation. However, the expression of *RAS* was equivocal, since *RALGEF* and *RALA/B* were up-regulated, and the switching of the signals related to *RAS* was ON. The up-regulation of TGF-β led to the gene expression of *SMAD 2/3* and *p21 CIP1*. In the nucleus, the gene expression of *WNT*, *MYC*, *BRCA1*, and *CHK1* was down-regulated, and cell-cycle progression in the S phase was inhibited by activated *p21 CIP1*. Therefore, the accelerator of cell cancerization in the nucleus was not increased. For interleukin (IL)-6 signaling, the up-regulation of *IL-1R* and *TNFR1* led to transcriptional activation related to *NIK* [18] and *NF-κB*. *JAK/STAT*, which acts as a receptor for *IL-6*, was activated by the up-regulation of *IL-6R*, which subsequently led to the transcriptional activation of vascular endothelial growth factor A (*VEGFA*) (Figure S15). On the other hand, the gene expression of several antioxidant proteins, including detoxifying factors in the *NRF2* oxidative stress response pathway, were increased following 1 h of treatment with TPA. In the pathway ATP binding cassette subfamily C member 4 (*MRP4*), sequestosome 1 (*SQSTM1*), *HO-1*, superoxidase dismutase (*SOD*), small musculoaponeurotic fibrosarcoma oncogene (*MAF*), nuclear factor, erythroid 2-like 2 (*NRF*2), and glutathione s-transferase (*GST*) were up-regulated (Figure S9). These are detoxification factors, antioxidation factors, or anti-inflammatory factors. NRF2 suppresses inflammation via the suppression of the gene expression of *IL-6* and *IL-1* [19]. However, the transcriptional activation of genes that were activated by IL-6 signaling and IL-1 signaling in Bhas 42 cells treated with TPA for 6 h was not suppressed by the up-regulation of *NRF2*. In the pathway cell cycle control of chromosomal replication, 16 genes were reduced (Figure S16), including those associated with the G1–S transition and subsequent DNA replication. In the xenobiotic metabolism pathway, the up-regulation of *AHR* led to the activation of *GST* in phase 1 metabolizing enzymes; however, the gene expression of *CYP1B1* in phase 2 metabolizing enzymes was decreased (Figure S10). The above switch in transcriptional activation and cell proliferation was activated by *IL-6* signaling (Figure S15), IL-1 signaling (Figure S17), and JAK/STAT signaling (Figure S18). However, homeostasis for cancer suppression was activated by NRF2 signaling, and cell cycle control was down-regulated by the inactivation of DNA replication in the G1–S phase of the cell cycle in Bhas 42 cells treated with TPA for 6 h. In the pathways of cancer suppression, such as NRF2 signaling, inactivation of DNA replication at the G1-S phase of the cell cycle was activated. On the other hand, the activation switches for cancer, such as the transcriptional activation and cell proliferation caused by IL-6 signaling (Figure S15), IL-1 signaling (Figure S17), and JAK/STAT signaling (Figure S18), were activated at 6 h after treatment with TPA.

#### 2.4.3. Gene-Expression Characteristics of 24 H TPA Treatment

Based on the analysis of genes whose expression was altered by 24 h of TPA treatment, the top-25 pathways are listed in descending order of –log (*p*-value) (Figure 5C). No genes were down-regulated in the 17 pathways. The 25 pathways were divided according to the goal of the system and comprise the following five groups: “cell cycle”, “DNA repair”, “RAN (*RAS*-related nuclear protein)”, “sumoylation”, and “synthesis of folic acid and nucleic acid”. The 14 pathways belonging to “cell cycle” are cell cycle control of chromosomal replication, role of BRCA1 in DNA damage response, hereditary breast cancer signaling, mitotic roles of polo-like kinase, role of CHK proteins in cell cycle checkpoint control, aryl hydrocarbon receptor signaling, GADD45 signaling, estrogen-mediated S-phase entry, ATM signaling, cell cycle: G1/S checkpoint regulation, cell cycle: G2/M DNA damage checkpoint regulation, glioma signaling, small-cell lung cancer signaling, and ovarian cancer signaling. The “DNA repair” group pathways are mismatch repair in eukaryotes, DNA double-strand break repair by homologous recombination, and the BER pathway. In the pathway cell cycle control of chromosomal replication, all 16 represented genes were increased (Figure S19). Chromosomal DNA replication in eukaryotic cells, which includes the recognition of origins, binding to replication origins, the loading of DNA polymerase onto origins, and the elongation of newly synthesized DNA, was activated. In mismatch repair in eukaryotes, the expression of all 11 genes was increased (Figure S20). These genes were related to mismatch recognition, strand discrimination, strand excision, and DNA resynthesis. In the pathway role of BRCA1 in DNA damage response, genes with the annotations of DNA repair following S phase and G1 arrest, homologous recombination, and checkpoint regulation were up-regulated (Figure S21). In the hereditary breast cancer signaling pathway, DNA repair, DNA damage responses and checkpoint control, protein ubiquitination, transcription control and chromatin remodeling, and *P53* were increased (Figure S22). In the mitotic roles of polo-like kinase pathway, which includes key regulators of the cell cycle in mammalian species and proteins that activate the separation of centromeres to opposite poles of the cell in the G1 and S phases of the cell cycle [20], the expression of 17 genes (including polo-like kinase 1 and polo-like kinase 4) was increased, and that of a single gene was decreased (Figure S23). In role of CHK proteins in cell cycle checkpoint control, 16 genes increased, and no genes showed a decrease in expression (Figure S24). Among the genes that were increased, claspin and Chk1, in a complex, regulate each other to ensure appropriate cell-cycle progression and replication-checkpoint control. In GADD45 signaling, the expression of eight genes, including growth arrest and DNA damage-inducible 45 (*GADD45*), which is one of several known *P53* target genes, were increased. However, cyclin B1 was not inhibited by GADD45 (Figure S25). In estrogen-mediated S-phase entry, all 10 genes were increased (Figure S26); this pathway moderates cellular functions in a wide variety of tissues and influences cell proliferation in the female reproductive tract and mammary gland. Cyclin D1, cyclin A, and cyclin E activated by *c-MYC* increased the transcription factor Dp 1 (*DP1*). S-phase factors, such as cyclin A2, cyclin E1, cyclin E2, cyclin-dependent kinase 1 (*CDC2*), cell division cycle 25A (*CDC2*5), and S-phase kinase-associated protein 2 (*SKP*2), were activated. In the RAN signaling pathway, which was classified as part of the “*RAN*” group, the expression of all eight genes was increased (Figure S27). *RAN*, a member of the *RAS* GTPase family, plays important roles in nucleocytoplasmic transport, and the overexpression of *RAN* in pancreatic cancer tissues is highly correlated with histological grade, suggesting that *RAN* plays a role in tumor progression [21]. *RAN* has also been implicated in cell-cycle regulation [22]. In the sumoylation pathway (Figure S28), which was classified as part of the “sumoylation” group, the expression of six genes was increased. The up-regulation of the *SUMO1*-activating enzyme subunit 1 activated *SUMO,* and the Ran GTPase-activating protein 1 was stabilized via sumoylation by *SUMO*.

**Figure 6:**
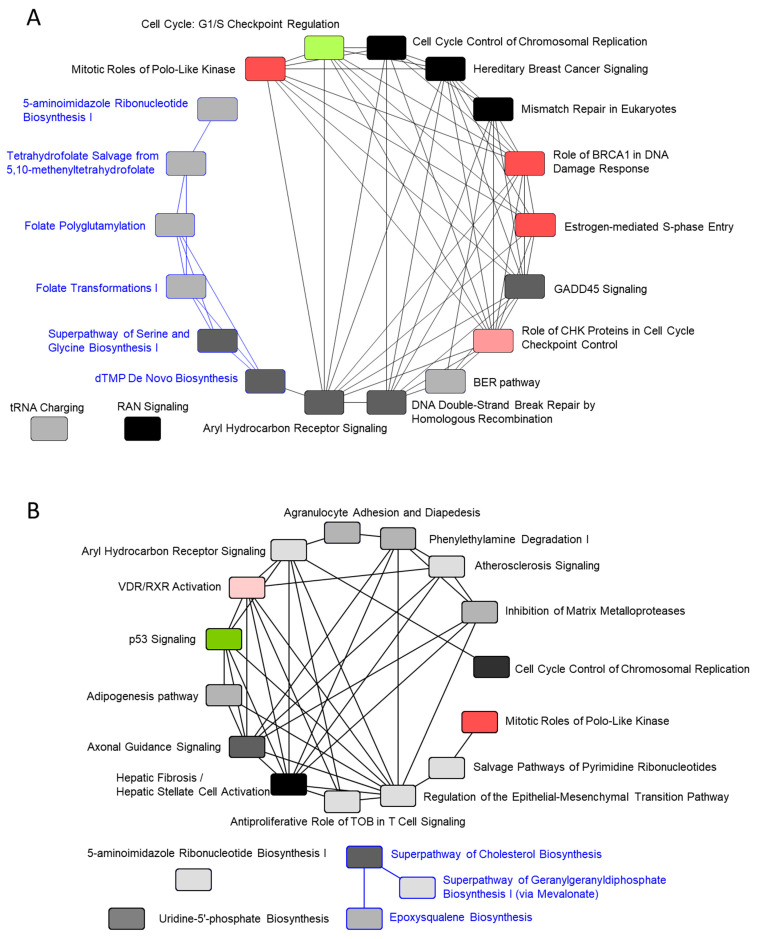
Overlapping of the top-25 canonical pathways. TPA treatment for (**A**) 24 h and (**B**) 8 days. The color of the pathway is red when the z-score is larger than 0, and greenish when it is below 0. When the z-score is 0, the color is distinguished by the brightness of the monotone according to the value of the −log (*p*-value). Common genes among the respective pathways are connected by lines. The pathway connected by the blue line in (**A**) is the pathway of folate metabolism. The pathway connected by the blue line in (**B**) is the pathway for the biosynthesis of cholesterol.

**Figure 7:**
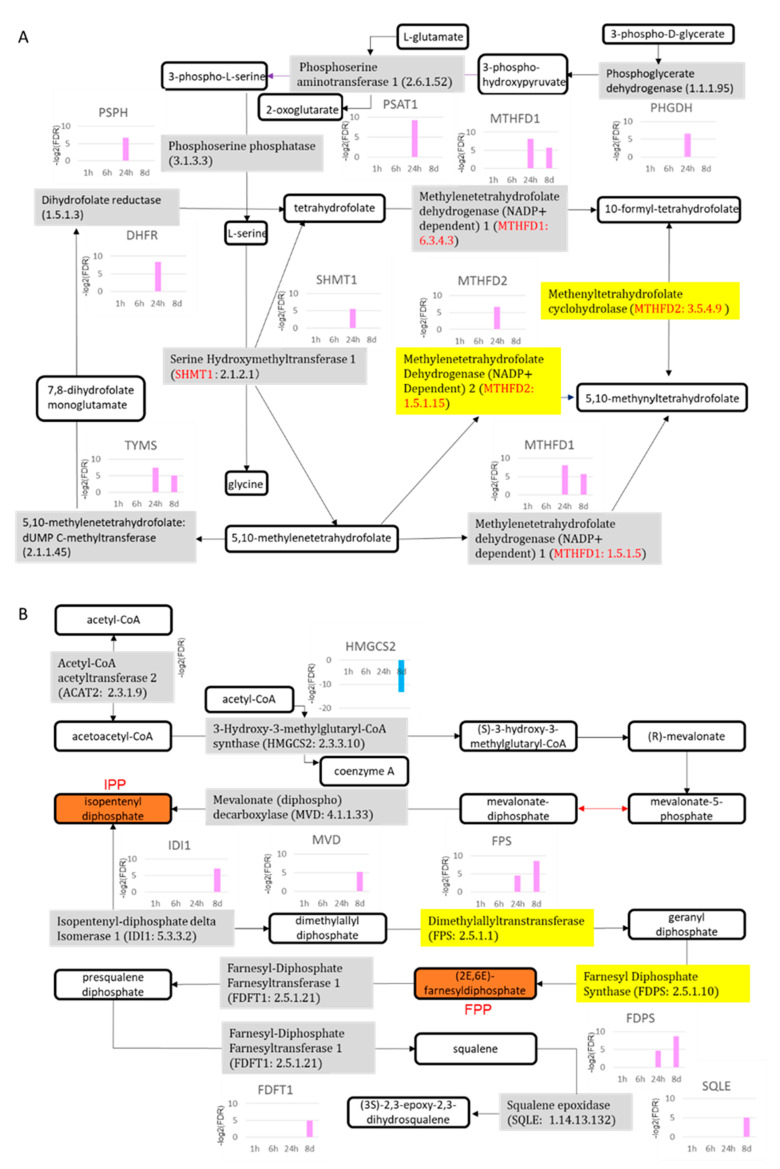
Folate metabolic and cholesterol biosynthetic pathways. (**A**) Folate metabolic pathway; (**B**) cholesterol biosynthetic pathway. Gray and yellow are enzymes; others are biological products. 8d: 8 days.

Overlapping the canonical pathways within the top-25 according to −log (*p*-value) yielded a cluster comprising five pathways that were classified as the “synthesis of folic acid and nucleic acid” group: superpathway of serine and glycine biosynthesis I, tetrahydrofolate salvage from 5,10-methenyltetrahydrofolate, dTMP de novo biosynthesis, folate polyglutamylation, and folate transformations I, the last of which formed a separate cluster from the other 20 pathways (Figure 6A). The gene expression of all 10 enzymes related to the metabolites of folate shown in Figure 7A was increased. The gene expression of 5,10-methylenetetrahydrofolate (1.5.1.15) and 5,10-methenyltetrahydrofolate (3.5.4.9) 5-hydrolase, named methylenetetrahydrofolate dehydrogenase 2 (*MTHFD2*), was increased in the pathway folate transformations, I.

#### 2.4.4. Gene-Expression Characteristics of 8-Day TPA Treatment

When analyzing the canonical pathways that overlap within the top-25 −log (*p*-value) for the 8-day TPA treatment condition (Figure 5D), two clusters related to folate and cholesterol formed a cluster separate from the other 20 pathways. The third and fourth among those 20 pathways were cell cycle control of chromosomal replication and p53 signaling axonal guidance signaling (Figure 6B). In cell cycle control of chromosomal replication, the expression of all nine genes was increased (Figure S28); among these, genes annotated to G1-S transition and following DNA replication were enhanced. Among members of the p53 signaling pathway, the gene expression of Wilms’ tumor 1 (*WT1*) was increased remarkably (Figure S29). A recent study of the involvement of *WT1* in malignant cells unexpectedly revealed a potential role for *WT1* as an oncogene [23,24]. The prime evidence supporting this role is the overexpression of wild-type *WT1* in a variety of human cancers of both hematological and non-hematological origin.

Another cluster of canonical pathways associated with 8-day TPA treatment consisted of the superpathway of cholesterol biosynthesis, in which the expression of the genes for mevalonate (diphospho) decarboxylase, isopentenyl-diphosphate delta isomerase 1, dimethylallyl-diphosphate, farnesyl diphosphate synthase, farnesyl- diphosphate farnesyltransferase 1, squalene epoxidase, and 24-dehydrocholesterol reductase was increased, and that of squalene synthase (*SQS*) was particularly enhanced (Figure 7B). The remaining cluster included pathways related to metabolites of folate; six enzymes, including 5,10-methylenetetrahydrofolate (1.5.1.15) and 5,10-methenyltetrahydrofolate 5-hydrolase (3.5.4.9) (*MTHFD2*), showed increased gene expression (Figure 7A). Substantially similar pathways related to folate metabolism were activated in both the 24 h and 8-day treatment groups; however, no gene in the superpathway of serine and glycine biosynthesis I pathway was increased in expression in the 8-day group.

#### 2.4.5. DNA Methylation

DNA methylation is a well-known transcriptional regulator, and many studies have addressed the relationship between DNA methylation and carcinogenesis, including non-genotoxic carcinogenesis [25,26]. Epigenetic profiles define the signatures of xenobiotic exposure [27]. We thus analyzed the pathways related to DNA methylation (Figure S30 and Table S31). In the pathway DNA methylation and transcriptional repression, *SAP180* gene expression was down-regulated after 1 h of TPA treatment, whereas *DNMT1* was down-regulated and *DNMT3A* was up-regulated at 6 h. After 24 h of TPA treatment, *DNMT1*, *Mi2*, and *MDB3* were up-regulated, whereas *DNMT3A* was down-regulated. After 8 days, the gene expression of *DNMT1* and *SAP30* was up-regulated, but *DNMT3A* was down-regulated. These results show that the transcription of *DNMT3A*, which initiates DNA methylation, was up-regulated at 6 h but down-regulated after TPA treatment for 24 h and 8 days. In contrast to *DNMT3A*, the transcription of *DNMT1*, which maintains DNA methylation, was up-regulated.

#### 2.4.6. γ-Glutamyl Cycle and Cytochrome P450 Family

The relevance of Glutathione (GSH), gamma-glutamyltransferase (GGT), gamma-glutamyl cyclotransferase (*GGCT*) and gamma-glutamylcysteine synthetase (GGCS), which are part of the γ-glutamyl cycle, to tumorigenesis has been reported [28,29]. GSH detoxifies xenobiotics by such as antioxidant effect while also conferring therapeutic resistance to cancer cells, therefore, GSH metabolism plays both beneficial and pathogenic roles in a variety of malignancies. After 6 h of treatment with TPA, *GGCT* was increased, and after 1 h, 24 h and 8 days of treatment with TPA GGCS was increased. By contrast, *GGCS* was decreased after 24 h and 8 days of treatment with TPA (Figure S32). 

When considering the effect of metabolism on the transformation of Bhas 42 cells, it is important to obtain information regarding the CYP enzymes in Bhas cells. Regarding the gene expression variation associated with cytochromes P450 (Cyp), cytochrome P450, family 1, subfamily a, polypeptide 1 (*CYP1a1*), and cytochrome P450, family 1, subfamily b, polypeptide 1 (*CYP1b1*) were increased (FDR, 0.010) in Bhas 42 cells treated for 1 h with TPA (Figure S10 and Table S33); in addition, *CYP1b1* expression was decreased (FDR, 0.001) after 6 h of TPA treatment.

### 2.5. Collation of the Pathways of Cell Transformation and the Hallmarks of Cancer

In recent years, various discussions of cancer biology have been advanced, and several “hallmarks of cancer” have been proposed [13,30,31,32]. We describe the relationship between the pathway analysis results and the hallmarks of cancer (Table 1).

#### 2.5.1. Hallmarks of Cancer with 1 H TPA Treatment

The first tumor-accelerated responses at 1 h after TPA treatment began with genes in “deregulated cellular metabolism”, with the up-regulation of *CYP1a1* and *CYP1b1* (Figure S10), and with genes in “tumor-promoting inflammation”, with the up-regulation of prostaglandin E synthase in prostanoid biosynthesis (Figure S34). Additionally, the suppression of anti-growth signaling by the down-regulation of *LATS1/2* in HIPPO signaling was also initiated (Figure S35). In "resisting programmed cell death", *BIRC6*, anti-apoptosis gene, was up-regulated. 

#### 2.5.2. Hallmarks of Cancer with 6 H TPA Treatment

Following 6 h of treatment with TPA, the inactivation of HIPPO signaling, which was observed after 1 h of treatment, was moderated by the up-regulation of *YAP/TAZ* and *TEAD* (Figure S36). The tumor-suppressed response was activated by the activation of evading anti-growth signaling via the up-regulation of prostaglandin D2 synthase in prostanoid biosynthesis (Figure S37). On the other hand, gap junction signaling with the down-regulation of CONNEXIN was also activated (Figure S39) as a tumor-accelerated response by “evading anti-growth signaling”. The characteristic reaction after 6 h of treatment was the activation of “tumor-promoting inflammation”, such as IL-1 signaling with the up-regulation of *IL1RAP* and *TOLLIP* (Figure S17); IL-2 signaling with the up-regulation of *IL2Rg* and STAT5 (Figure S40); IL-6 signaling with the up-regulation of *IL-6R*, *JAK2*, and *STAT3* (Figure S15); prostanoid biosynthesis with the up-regulation of prostaglandin E2 synthase (Figure S37); and *TNFR2* signaling with the up-regulation of *TNFR2* (Figure S6). Avoiding immune destruction via the activation of interferon signaling with the up-regulation of *IFNg Rb* and *JAK2* (Figure S41); PD-1 prevents immune destruction in tumors due to T-cell activation and apoptosis; PD-L1 activators, such as *TNFR* and *IFNγR2*, were increased by 6 h of treatment with TPA; on the other hand, T cell proliferators and activators, such as *IL2R*, *JAK* and *STAT5*, were also increased in the PD-1 and PD-L1 cancer immunotherapy pathways (Figure S42); the “tumor microenvironment” with the activation of JAK/STAT signaling and the up-regulation of *JAK2* and *STAT3* (Figure S18); and integrin signaling with the up-regulation of *PDGF*β, *MLK3*, *PARVIN-*β, *PXN*, *NEDD9*, and *FYN* (Figure S43) were also activated as factors that promote carcinogenesis. In addition, “sustained growth signaling”, aryl hydrocarbon receptor signaling with the up-regulation of *AHR* (Figure S44), and genetic instability including *DNMT3A*, which initiates DNA methylation, were up-regulated at 6 h (Figure S30). Tissue invasion and metastasis, which includes glioma invasiveness signaling with the up-regulation of *CD44* and matrix metalloproteinase 9 (*MMP*9) (Figure S45), was activated after 6 h of treatment with TPA.

#### 2.5.3. Hallmarks of Cancer with 24 H TPA Treatment

After 24 h of treatment with TPA, hyperplasia- and neoplasia-related gene expression was activated in the hallmarks of deregulated cellular metabolism, evading anti-growth signaling, sustained growth signaling, genetic instability, enabled replication immortality, avoiding immune destruction, inducing new blood flow, and tissue invasion and metastasis. Tumor-accelerated responses were activated by evading anti-growth signaling with the down-regulation of prostaglandin D2 synthase. Deregulated cellular metabolism, which contains folate biosynthesis with the up-regulation of *DHFR*, *GART*, *MTHFD*1, *MTHFD1L*, *MTHFD*2, *PHGDH*, *PSAT*1, *PSPH*, *SHMT1*, *SHMT*2, and *TYMS* (Figure 7A), and cholesterol biosynthesis with the up-regulation of *ACAT2*, *FDPS*, *FPS*, and *LBR* (Figure 7B), was activated. On the other hand, the tumor-suppressed responses included the activation of gap junction signaling with the up-regulation of CONNEXIN as evading anti-growth signaling (Figure S46), and the inactivation of tumor-promoting inflammation by the down-regulation of prostaglandin E2 synthase in prostanoid biosynthesis (Figure S37). In “resisting programmed cell death”, the up-regulation of *BIRC6* and down-regulation of *clAP* and *CASP6* suppressed apoptosis (Figure S47). At the same time, the activation of *PARP*, which is factor that promotes apoptosis, was also observed. The activation of “sustained growth signaling” contained cell cycle: G1/S checkpoint regulation with the up-regulation of *CDK4/6*, *CDC25A*, cyclin D, *c-MYC*, cyclin E, *RB*, *DP-*1, and *E2F* (Figure S48); cell cycle: G2/M DNA damage checkpoint regulation with the up-regulation of *CDC2*, cyclin B, and *CKS1* (Figure S49); VEGF signaling with the up-regulation of *VEGF*, *EIF*, and *RAS* (Figure S50); and mTOR signaling with the up-regulation of *PP2A*, *elF4E*, *elF4G*, *elF3*, and 40S ribosome (Figure S51). Genetic instability contained the role of BRCA1 in DNA damage response with the up-regulation of *BRCA1* (Figure S21); role of CHK proteins in cell cycle checkpoint control with the up-regulation of *CHK1* (Figure S24); telomerase signaling with up-regulation of *DKC1* (Figure S52); mismatch repair in eukaryotes with the up-regulation of *EXO*1, *FEN1*, *Polδ*, *PCNA*, *MSH6*, and *RPA* (Figure S20); DNA methylation and transcriptional repression signaling with the up-regulation of *DNMT1A*, *MBD3*, and *MI2* (Figure S30); and mitochondrial dysfunction with the up-regulation of *CYTC*, *GSR*, *HTRA2*, and *ATP5G1* (Figure S53). The activation of telomerase signaling with the up-regulation of *DKC1*, *HSP90*, and p23 (Figure S52) likely induces the activation of enabled replication immortality as well. Regarding “avoiding immune destruction”, PD-1 prevents immune destruction in tumors due to T-cell activation and apoptosis. T cell proliferators and activators, *PDCD4* and *PTEN* respectively, were decreased. (Figure S42).

Inducing new blood flow contained VEGF signaling with the up-regulation of *VEGF*, *EIF*, and *RAS* (Figure S50), and mTOR signaling with the up-regulation of *PPA2*, *elF4E*, *elF4G*, *elF3*, and 40S ribosome (Figure S51). Tissue invasion and metastasis contained glioma invasiveness signaling with the up-regulation of *RAMM* and *UPAR* (Figure S54).

#### 2.5.4. Hallmarks of Cancer with 8-Day TPA Treatment

At 8 days after TPA treatment, the tumor-accelerated responses included the activation of deregulated cellular metabolism containing folate and cholesterol biosynthesis, genetic instability containing role of CHK proteins in cell cycle checkpoint control and DNA methylation, and transcriptional repression signaling; these remained activated after 8 days of treatment with TPA. The tumor-suppressed responses included the activation of CONNEXIN in gap junction signaling for evading anti-growth signaling and the down-regulation of prostaglandin E2 synthase in prostanoid biosynthesis (Figure S55) for the inactivation of tumor-promoting inflammation. On the other hand, the activation of evading anti-growth signaling with the down-regulation of prostaglandin D2 synthase was a tumor-accelerated response that continued after 24 h of treatment with TPA.

As shown in the simplified diagram in Table 1, at 1 h after TPA treatment, CYP metabolism began as a molecular initiating event. And resisting programmed cell death was up-regulated with anti-apoptosis, and a key event in the suppression of carcinogenesis was also activated. Although resisting programmed cell death was decreased after 6 h of TPA treatment, tumor microenvironment, tumor-promoting inflammation, avoiding immune destruction, sustained growth signaling, genetic instability, and tissue invasion and metastasis, which are key events that accelerate carcinogenesis, were activated. After 24 h of TPA treatment, deregulated cellular metabolism, avoiding immune destruction, sustained growth signaling, genetic instability, tissue invasion and metastasis, and inducing new blood flow, which are key events in the acceleration of carcinogenesis, were activated. In addition, resisting programmed cell death was inhibited, and the carcinogenesis brake was released. After 8 days of TPA treatment, deregulated cellular metabolism and genetic instability remained enhanced, as did three other key events, which were also observed at 24 h.

## 3. Discussion

Seven genes (*ANGPTL4*, *HMGA1*, *HMGA2*, *MPP6*, *RBM3*, *SLPI*, and *ZWINT*) showed increased expression in all the TPA treatment groups. The elevated expression of angiopoietin-like 4 (*ANGPTL4*) is a common feature of many human tumor types, and its suppression impairs tumor growth through the enhancement of anoikis or apoptosis [33]. Notably, a previous study showed that c*ANGPTL4* regulates cancer-cell proliferation and that *STAT1* induction is dependent on the NADPH oxidase-mediated production of superoxide (O_2_^−^), as well as on the Src and mitogen-activated protein kinase (MAPK) pathways [34]. 

High-mobility group AT-hook 1 (*HMGA1*) and high-mobility group AT-hook 2 (*HMGA2*), a non-histone protein, are overexpressed in diverse cancers, where they regulate developmental genes [35,36,37,38,39,40,41,42,43,44,45]. Work on murine intestinal stem cells (ISCs) revealed a novel role for Hmga1 in enhancing self-renewal by amplifying Wnt signaling by inducing both genes expressing Wnt agonist receptors and Wnt effectors [46]. *HMGA1* also builds a stem-cell niche by up-regulating *SOX9*, a factor required for differentiation to Paneth cells. These cells constitute an epithelial niche by secreting Wnt and other factors to support ISCs. *HMGA1* is also highly up-regulated in colon cancer compared with nonmalignant epithelium, and *SOX9* becomes overexpressed during colon carcinogenesis. *SOX9* mRNA expression was significantly increased 1 and 6 h after the TPA 5 ng/mL treatment of Bhas 42 CTA (Figure S56). Bhas 42 CTA may have “constructed” *HMGA1* into the stem-cell niche by up-regulating *SOX9*. *HMGA2* increases cancer-cell proliferation by promoting cell-cycle entry and inhibiting apoptosis [47]. In addition, *HMGA2* influences different DNA-repair mechanisms and promotes epithelial-to-mesenchymal transition by activating signaling via the MAPK/ERK, TGFβ/Smad, PI3K/AKT/mTOR, NF-kB, and STAT3 pathways.

*MPP6* is consistently expressed in glioblastoma stem-cell cultures and is inconsistently expressed in neural stem-cell cultures [48].

*RBM3*, a translation-regulating protein, is significantly up-regulated in human tumors, showing stage-dependent increases in colorectal tumors. *RBM3* regulates global mRNA translation by interacting with the 60S ribosome [49,50,51]. *RBM3* overexpression in NIH3T3 mouse fibroblasts and SW480 human colon epithelial cells increases cell proliferation and the development of compact, multicellular spheroids in soft agar, suggesting its ability to induce anchorage-independent growth [52]. By contrast, the down-regulation of *RBM3* in HCT116 colon-cancer cells using specific siRNAs decreases cell growth in culture; this decrease is partially overcome by treatment with prostaglandin E2, a product of cyclooxygenase-2 enzyme activity. The knockdown of *RBM3* also led to the growth arrest of tumor xenografts. Through these data, Sureban et al. demonstrated that the RNA-stabilizing and translation-regulating protein *RBM3* was a novel proto-oncogene that induced transformation when overexpressed and was essential for cells to progress through mitosis.

In their review [53], Bouchard et al. reported that secretory leukocyte peptidase inhibitor (*SLPI*) expression was highly up-regulated in pancreatic, papillary thyroid, uterine cervix, endometrial, and ovarian cancers; by contrast, *SLPI* was underexpressed in nasopharyngeal carcinoma, bladder tumors, and some breast carcinomas, although the overexpression of this protein correlates with more invasive forms of breast carcinoma [54,55,56].

The expression of *ZWINT* is correlated with that of the proliferation marker gene *MKI67*. Therefore, the up-regulation of *ZWINT* observed in TPA-treated Bhas 42 cells may reflect an increased proliferation index for muscle-invasive tumors. *ZWINT* plays a role in mitotic checkpoints [57], an essential step for cell division. Moreover, a high expression of *ZWINT* is associated with a poor prognosis in pulmonary carcinomas [58].

DNA methylation was preserved at 24 h after TPA treatment, and the activation of carcinogenesis was maintained or enhanced. The GO terms of cell division and cell cycle checkpoint continued to be up-regulated 8 days after TPA treatment. In addition, the response after 8 days of TPA treatment was similar to (albeit weaker than) that after 24 h. The GO terms associated with the 24 h-treatment group included all those of the 8-day group, and many genes that showed similar increases and decreases in expression in both treatment groups were extracted. This analysis suggests that the gene expression after 24 h of treatment is predictive of the gene expression that leads to the malignant transformation of Bhas 42 cells when the test substance is TPA. TPA-associated genes differentially expressed in the Bhas 42 CTA included markers such as *RAN*, *MTHFD2*, *WT1*, and *AURORA-A*, which lead to carcinoma formation and malignant transformation in humans and test animals. Intriguingly, the pathway of one-carbon metabolism, which is related to the synthesis of folic acid and was expressed in the 24 h-treatment group, and the pathways of cholesterol synthesis and the synthesis of folic acid, which were characteristic of 8-day TPA treatment, were enhanced in these treatment groups (Figure 7A,B). Nilsson et al. compared the mRNA profiles of 1454 metabolic enzymes across 1981 tumors spanning 19 cancer types to identify enzymes that were consistently differentially expressed [59]. In particular, the authors found that the *MTHFD2* RNA and protein were markedly elevated in many cancers, and these increased levels are correlated with poor survival in breast cancer. In addition, *MTHFD2* is expressed in developing embryos but is absent from most healthy adult tissues, even those that are proliferating. This study showed the importance of the mitochondrial compartmentalization of one-carbon metabolism in cancer and raises important therapeutic hypotheses. *AURORA-A*, the expression of which was increased in the pathway of molecular mechanism of cancer after 1 h (Figure S6), 24 h (Figure S57) and 8 days (Figure S58) of TPA treatment, regulates genomic instability and tumorigenesis through cell cycle dysregulation and *BRCA2* suppression [60]. The knockdown of *AURORA-A* reduces centrosome amplification, the malformation of mitotic spindles, and chromosome aberrations, leading to decreased tumor growth. The overexpression of *AURORA-A* represses p21, pRb, and *BRCA2*, thus promoting cell-cycle progression, suppressing apoptosis, and promoting genomic instability, leading to increased tumorigenesis [61].

In the pathway for cholesterol biosynthesis shown in Figure 7B, the generation of isopentenyl diphosphate (IPP) by mevalonate (diphospho) decarboxylase (MVD: 4.1.1.33) was activated. The generation of (2E, 6E)-farnesyl diphosphate (FPP) was activated by isopentenyl diphosphate delta isomerase 1 (IDI1: 5.3.3.2), dimethylallyltranstransferase (FPS: 2.5.1.1), and farnesyl diphosphate synthase (FDPS: 2.5.1.10). Furthermore, farnesyl diphosphate farnesyltransferase 1 (FDFT1: 2.5.1.21), which generates squalene, was also activated. These observations suggest that the mevalonate (MVA) pathway was activated. Several papers have reported that the MVA pathway is an essential metabolic pathway that uses acetyl-CoA to produce sterols and isoprenoids that are integral to tumor growth and progression [62]. Glioblastoma, the most aggressive brain cancer, is highly dependent on the MVA pathway for the synthesis of lipid moieties critical for cell proliferation, and FDPS is a key intermediate enzyme in this pathway [63]. Many oncogenes such as *RAS* encode proteins produced in the aqueous environment of the cell cytosol but require relocation to the lipophilic cell plasma membrane for activation. This is achieved by the attachment of farnesol, supplied from FPP, via a specialized enzyme system [64,65]. In addition, the farnesyl tail of K-RASs binds proteins to cell membranes and restricts K-RAS from diffusing freely throughout the cytoplasm [66,67,68]. This farnesyl tail also plays an important role in transporting RAS to appropriate intracellular compartments for cellular signaling events [69]. Furthermore, the prevention of farnesylation prevents the activation of these RAS proteins as signal-transducing agents in the regulation of cell-transforming activity [70]. Thus, in the activation of cholesterol biosynthesis at 8 days, the production pathways for IPP and FPP, which are the sources of prenylation for activating RAS via posttranslational modification, are activated. These results suggest that the increased expression of RAS protein after 24 h and 8 days of treatment with TPA is post-translationally modified to cause the activation of *RAS* via the increased expression of *FPS* and *FPDS*. In addition, cholesterol is probably a raw material for the synthesis of the cell membrane, along with cell proliferation. Therefore, the increased expression of *RAS* after 24 h and 8 days of treatment with TPA may be related to increased expression of *FPS* and *FPDS*. Regarding the relationship between SQS and cancer, cholesterol is required for the growth of cancer cells [71,72], and Koen et al. reported that SQS activity and de novo cholesterol synthesis were the determinants of membrane microdomain-associated cholesterol in cancer cells [73].

The Bhas 42 cell line was derived by the stable transfection of the v-Ha-*ras* oncogene into a mouse fibroblast cloned cell line [4]. The expression of *RAS* in this study was down-regulated at 6 h after TPA treatment and was up-regulated at 24 h and 8 days after TPA treatment. However, this pathway was not only directly related to *RAS,* but various other pathways in the hallmarks of cancer were activated or inactivated in the process of cell transformation in Bhas 42 cells following TPA treatment.

**Figure 8:**
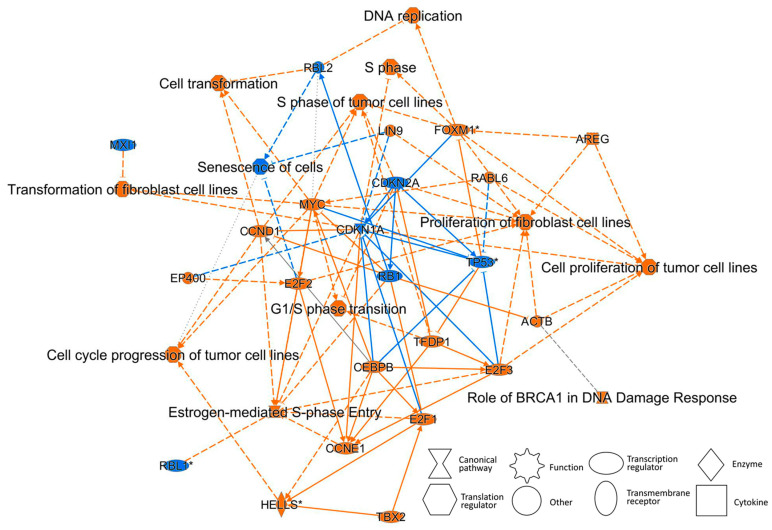
Graphical summary of the molecule and its function following treatment with TPA for 24 h. Graphical summary was analyzed using IPA software. The activated molecule or function has a positive z-score and is therefore orange. The suppressed molecule or function has a negative z-score and is therefore blue. Estimated edges are shown as dotted lines (not solid lines).

As a hallmark of cancer, cancer activators were activated 24 h after TPA treatment (Table 1). The “Graphical summary” in the analysis, performed using the Ingenuity Pathway Analysis (IPA) software, shows that cell transformation, the promotion of cancer activators, and the suppression of tumor-suppressor factors occurred in a relatively short period of 24 h of TPA treatment (Figure 8 and Figure S59). In the graphical summary, the cancer-activation response is clear. The conclusion of this IPA is considered to be consistent with that cancer activation occurs at 24 h remarkably, as a hallmark of cancer.

In vitro cell-transformation assays, which are accompanied by a focus formation composed of transformed cells, may mimic different stages of the in vivo neoplastic process and represent an excellent alternative for studying carcinogenesis and therapeutic options. Bhas 42 CTA is the first method to be tested that is accompanied by focus formation for the detection of tumor-promoting compounds, including NGTxC, and it has been certificated by the OECD [10]. Poburski et al. and Mascolo et al. reported the usefulness of using the two-step focus formation assay, Balb c/3T3 CTA, as a tool for investigating cancer mechanisms and therapies [74,75]. The cell-transformation assay, the result of which is judged by the focus formation at the endpoint, is a great tool for studying the mechanism of a fundamental step in the alteration of hyperplastic or dysplastic lesions to neoplastic lesions. Following epigenetic-mechanism analysis in Bhas 42 CTA, it was reported that four NGTxCs enriched differentially methylated regions at the CpG sites in cancer-related categories, including “cell-to-cell signaling and interaction” as well as “cell death and survival” [76]. In the report, cells cloned from the transformed foci formed on the 21st day of the Bhas 42 CTA experiment were analyzed. On the other hand, our report shows actual changes in gene expression over time at 1 h to 8 days after treatment with the test substance. Notably, a key benefit of this assay is that transcriptomics analysis in Bhas 42 CTA can provide focus-formation results for gene expression directly following treatment with a tumor-promoting compound alone. This is the first paper to describe variations in gene expression following treatment with a tumor-promoting and cell-transforming compound at 1 h to 8 days after TPA treatment in the Bhas 42 CTA. The gene-expression analysis of Bhas 42 cells treated with TPA, a typical tumor promoter, has suggested the mechanism that underlies cell transformation in these cells, and revealed the transformation-concurrent expression of various genes involved in cancers and tumors. In addition, the current results show that GO terms, biological functions, and pathways related to the GO category cancer were up-regulated during cell transformation in TPA-treated Bhas 42 cells. These results for Bhas 42 CTA indicate a cancerous process taking place in cells over time that can only be confirmed in vitro, and which we consider as having contributed to adverse outcome pathways and IATA. These findings suggest that Bhas 42 CTA can accurately demonstrate the tumorigenicity and carcinogenicity of the tested chemicals.

## 4. Conclusions

In this study, to measure gene expression in Bhas 42 cells treated with TPA 5 ng/mL, we performed three complete repeat experiments of gene expression over time—from 1 h after treatment to 8 days after treatment—at the initial stage of cell transformation. At the same time, three complete repeats of Bhas 42 CTA were extended until Day-21 for the transformation assay. A large number of mRNAs that were significantly increased or decreased with respect to the solvent control at FDR <0.05 were analyzed by GO and IPA pathway analysis and further identified as hallmarks of cancer.

As a result, the following changes in cancer activation or suppression were observed with the passage of time after TPA treatment. As shown in Figure 1 and Table 1, the expression of cancer-related genes was accelerated earlier than changes in cell transformation morphology, *CYP* gene expression was observed as MIE 1 h after treatment, and gene expression that accelerates carcinogenesis began 6 h later without remarkably increased cell density. Furthermore, after 24 h, the increase in gene expression for malignant transformation accelerated with the increased cell density, and after 8 days, in the early-stage cell transformation morphology, some of the gene expression of malignant transformation was persistent. That is, one hour after TPA treatment, CYP metabolism began as a molecular initiation event. Six hours after TPA treatment, programmed cell death was upregulated, suggesting a tendency to suppress cancer. Meanwhile, it was suggested that the important events that accelerate carcinogenesis had begun, including the tumor microenvironment, tumor-promoting inflammation, avoidance of immune destruction, sustained growth signaling, genetic instability, and cancer activation of tissue infiltration and metastasis. After 24 h of TPA treatment, deregulated cellular metabolism, avoiding immune destruction, sustained growth signaling, genetic instability, tissue invasion and metastasis, and inducing new blood flow, which are key events in the acceleration of carcinogenesis, were activated. In addition, programmed cell death was down-regulated, and the brakes on cancer suppression were in the process of being released. Eight days after TPA treatment, deregulated cell metabolism and genetic instability, which began after 24 h of treatment, were still ongoing.

As cancer-related marker genes, among the seven genes that showed increased expression at all time points in the TPA-treated group, *HMGA1*, *HMGA2*, *MPP6, RBM3*, and *ZWINT* were genes that were closely associated with cancer progression. In addition, the expression of many cancer-driving genes increased. Other genes that are differentially expressed in Bhas 42 CTA by TPA treatment include *RAN*, *MTHFD2*, *WT1*, and *AURORA-A*, which are markers that cause cancer formation and malignant transformation in humans and laboratory animals.

Since Bhas 42 cell line was established by transfecting the v-Ha-*ras* gene into a mouse fibroblast cloned cell line, it is premised that the transfected *RAS* gene could be one of the MIEs. However, we were able to confirm that Bhas 42 CTA exhibits not only the mechanism of *RAS*-related carcinogenesis, but also the broad mechanism of cell tumorigenesis and malignancy by chemical substances in this study. It has been suggested that the Bhas 42 CTA promotion test (stationary phase test) is an assay that can reproduce in vitro the cellular mechanisms of tumor formation and malignant transformation by NGTxC that cannot be detected in genotoxicity tests.

## 5. Methods

### 5.1. Cell Culture

Bhas 42 cells were obtained from the Hadano Research Institute, Food and Drug Safety Center (Kanagawa, Japan), which is the institution to which the cell-line establisher belongs, and a laboratory has been certified according to the good laboratory practice for non-clinical safety studies of drugs in Japan. The stock cells used were the fourth passage in our laboratory, and the number of passages was small. Although mycoplasma detection was not performed, cell experiments were conducted in a class 100,000 clean room to prevent mycoplasma contamination, and antibiotics were never used in the cell experiments. The morphological observation and doubling time of Bhas 42 cells were confirmed during the pre-culture of Bhas 42 CTA.

MEM was obtained from Nissui Pharmaceutical Co. (Tokyo, Japan). DMEM/F12 was purchased from GIBCO Laboratories (Grand Island, NY, USA). Fetal bovine serum (FBS) was purchased from Nichirei Biosciences Inc. (Lot No. S.028K8458, Australia). Bhas 42 cells were cultured in a medium consisting of MEM supplemented with 10% FBS at 37 °C in a 95% air/5% CO2 atmosphere for stock cells. Cells were treated with 0.25% trypsin (Wako Pure Chemical Industries, Osaka, Japan) and subcultured. The cell density was maintained at 70% confluence. After cell expansion, the cells were kept frozen at −80 °C in aliquots. Each experiment was performed using an aliquot of these stock cells. Plastic culture dishes and plates were purchased from Sumitomo Bakelite (Tokyo, Japan).

### 5.2. Cell-Transformation Assay at the Stationary Phase Using Bhas 42 Cells (Bhas 42 CTA Promotion Test)

The original Bhas 42 cell-promotion assay protocol [5] was employed with several modifications [10]. The frozen working stock cells were rapidly thawed, suspended in a medium consisting of DMEM/F12 supplemented with 5% FBS, and cultured in 100 mm culture plates in a volume of 10 mL of medium. When the cells reached approximately 70% confluence, they were trypsinized, suspended in DF5F at an appropriate density (10,000 cells/mL), and cultured in 90 mm culture plates (day −3). When these cells reached approximately 70% confluence, a cell suspension of 7 × 10^3^ cells/mL in a medium consisting of DF5F was prepared from the mother culture, 2 mL of which was distributed into each well of 6-well plates (1.4 × 10^4^ cells/well) (day 0). To assay the test chemicals, each dose group consisted of six wells. After cultivating the cells for 4 days, the medium was replaced with fresh DF5F medium containing 5 ng/mL TPA or DMSO. The final concentration of DMSO in the medium of the group treated with TPA and the control was 0.1%. The medium was replaced with fresh medium containing the test chemicals on days 7 and 11, and then fresh DF5F medium without TPA on day 14. On day 21, the cells were fixed with methanol for 30 min and stained with 5% Giemsa solution for 1 h. Transformed foci were characterized by the following morphological criteria: deep basophilic staining, the dense multi-layering of cells, the random orientation of cells at the edge of the foci, and more than 100 cells within a focus.

### 5.3. Statistical Analysis and Criteria of Judgment

The following criteria of positive control were used to evaluate the transformation results for TPA [10]: The statistical significance was evaluated using a one-sided Student’s *t*-test or an Aspin–Welch test (*p* < 0.05, upper-sided) depending on the results of the F-test for homoscedasticity. A chemical that satisfied these two criteria was judged as positive. If only the first criterion was met, the chemical was judged as equivocal. A chemical was deemed negative if it induced no statistically significant increase in transformed foci at any concentration.

### 5.4. Isolation of Total RNA

Three biological replicates from the independent thawing of stock cells for the microarray analysis were prepared for each group of TPA treatment times. Total RNA samples were subjected to DNA-microarray analysis as follows: Bhas 42 cells, after treatment with TPA, were washed three times with 2 mL of PBS(−) per well. The cells were dissolved with ISOGEN (Nippon Gene, Japan, Tokyo, Japan), and the total RNA was extracted and purified from the dissolution of the Bhas 42 cells in accordance with the protocol for the product. Furthermore, the total RNA was purified with an RNeasy Mini Kit (Qiagen, Tokyo, Japan) in accordance with the protocol (QIAGEN Supplementary Protocol: Purification of cytoplasmic RNA from animal cells using the RNeasy^®^ Mini Kit for the product. The quality and quantity of the total RNA were spectrophotometrically evaluated with an Agilent 2100 Bioanalyzer (Agilent Technologies Japan, Tokyo, Japan) and an RNA 6000 Nano Series II Kit (quality). The RNA integrity number (RIN) was computed using the 2100 Expert Software (Agilent Technologies Japan) to indicate the integrity of the total RNA samples on a scale of 1–10 [77]; the RNA integrity number of our total RNA isolated from the cells cultured in each plate was higher than 9.0.

### 5.5. DNA-Microarray Assay

cDNA was synthesized from 2 μg of purified total RNA, and then biotinylated aRNA was transcribed with T7 RNA polymerase. The aRNA quality was assessed with an Agilent 2100 Bioanalyzer, which showed sufficiently long viability for the purpose of our experiments. The aRNA was fragmented and hybridized to an Affymetrix Mouse Genome 430 2.0 Array (Affymetrix, Santa Clara, CA, USA) that contained probes for approximately 14,000 mouse genes. After hybridization at 45 °C for 16 h, the array was washed and stained with phycoerythrin. Fluorescence signals were scanned with the Affymetrix GeneChip System. The Affymetrix GeneChip Command Console (AGCC) software was used to reduce the array images to the intensity values for each probe (CEL files).

### 5.6. DNA-Microarray Data Analysis

The CEL files were quantified with the DFW method [78] using the statistical language R [79], version 2.7.1, and Bioconductor [80], version 2.2. Hierarchical clustering was then performed with the pvclust function [81] in R. To identify individual differences between the DMSO group and TPA group by treatment time in terms of differentially expressed genes, the rank products method [82] was applied to the DFW-quantified data. The annotation file for the Mouse Genome 430 2.0Array was downloaded from the Affymetrix Web site (10 August 2010; Mouse430_2.na31.annot.csv). By applying the rank products method to the DFW-quantified data, we selected the probe sets that were up-regulated and down-regulated (FDR < 0.05).

### 5.7. GO Analysis

The selected probe sets were functionally classified according to the biological process in Gene Ontology with the functional annotation tool DAVID [83,84] version 6.7. The probe set IDs provided by Affymetrix were used as the input data. For the gene-list manager on the DAVID website (http://david.abcc.ncifcrf.gov/, accessed on 21 February 2015), we selected the species option to limit the annotations exclusively to *Mus musculus*. For the population manager option, the mouse genome 430_2 platform was selected as the background. The functional annotation chart was analyzed based on biological processes in GO, GOTERM_BP_ALL. To extract the statistically over-represented GO terms in each group of differentially expressed genes and to correct the results by multiple testing, we used EASE scores to modify Fisher’s exact test *p*-values [85] and Benjamini and Hochberg FDR corrections [86]. A Benjamini and Hochberg FDR-corrected *p*-value < 0.01 indicated a significantly enriched GO term. To recognize the hierarchical structure of the selected GO terms, we used Quick GO (https://www.ebi.ac.uk/QuickGO/, accessed on 21 February 2015), an online analysis tool.

### 5.8. Functional Analysis and Pathway Analysis

FDRs that were up-regulated (FDR < 0.05) were transformed by log 2, and those that were down-regulated (FDR < 0.05) were transformed by −log 2. A transformed value with an FDR of 0 was entered as 20 for up-regulation and as −20 for down-regulation. The data set for the transformed FDRs and the probe IDs of the Mouse Genome 430 2.0Array were analyzed using the QIAGEN Ingenuity Pathway Analysis software package (QIAGEN, Redwood City, CA, USA). Biological function and canonical pathways were analyzed using the IPA software. The activation z-score was used to infer likely activation states of biological functions based on a comparison with a model that assigns random regulation directions (Ingenuity Downstream Effects Analysis in IPA). Under ideal circumstances (the “un-biased” case described below), the activation z-score can also be used to predict implicated biological functions independently from their associated *p*-values, based on a significant pattern match of up-/down-regulation. The primary purpose of the activation z-score is to infer the activation states (“increased” or “decreased”) of implicated diseases and biological functions.

## Figures and Tables

**Table 1:** Pathway and the hallmark of cancer.

Hallmark	Pathway	1 H	6 H	24 H	8 Days
Deregulated Cellular Metabolism	Aryl Hydrocarbon Receptor Signaling	↑*CYP1A1*, ↑*CYP1B1*			
Evading Anti-growth Signaling	HIPPO Signaling	↓*LATS*1/2	↓*LATS*1/2, ↑*YAP*/*TAZ*, ↑*TEAD*		
	Gap Junction Signaling		↓*CONNEXIN*		
Resisting Programmed Cell Death	Apoptosis Signaling	↑*BIRC6*	↑*CASP9*, ↑,*CASP3*	↓*CASP6*,↓*clAP*, ↑*PARP*,↑*BIRC6*	↓*GAS2*, ↓*clAP*
Avoiding Immune Destruction	Interferon Signaling		↑*IFNgRb*,↑*JAK2*	↑*STAT2*	
	PD-1, PDL-1 cancer immunotherapy pathway	↓ *TGF*-β	↑*CBLB*,↑*TGF*-β ↑*TNFR*, ↑*IFNγR2*, ↑*IL2R*	↓*CBLB*, ↓*PTEN*	↓*CDKN1B*(*P27kip*),↓*PDCD4*,↓*TGF*-β
Tumor-Promoting Inflammation	IL-1 Signaling		↑*IL1RAP*, ↑*TOLLIP*		
	IL-2 Signaling		↑*IL2Rg*, ↑*STAT5*		
	IL-6 Signaling		↑*IL-6R*,↑*JAK2*, ↑*STAT3*		
	TNFR2 Signaling		↑*TNFR2*		
Tumor microenvironment	JAK/STAT signaling		↑*JAK2*,↑*STAT3*		
	Integrin signaling		↑*PDGFβ*,↑*MLK3*, ↑*PARVIN*-*β*, ↑*PXN*,↑*NEDD9*, ↑*FYN*		
Tissue Invasion and Metastasis	Glioma Invasiveness Signaling		↑*CD44*,↑*MMP9*	↑*RAMM*, ↑*UPAR*	
Sustained Growth Signaling	Aryl Hydrocarbon Receptor Signaling		↑*AHR*		
	Cell Cycle: G1/S Checkpoint Regulation			↑*CDK*4/6, ↑*CDC25A*, ↑*Cyclin D*, ↑c-*MYC*, ↑*Cyclin E*,↑*RB*, ↑*DP*-1,↑*E2F*	
	Cell Cycle: G2/M DNA Damage Checkpoint Regulation			↑*CDC2*, ↑*Cyclin B*, ↑*CKS1*	
	VEGF Signaling			↑*VEGF*,↑*EIF*, ↑*RAS*	
	mTOR Signaling			↑*PPA2*,↑*elF4E*, ↑*elF4G*,↑*elF3*, ↑*40S*,↑*Ribosome*	
Genetic Instability	DNA Methylation and Transcriptional Repression Signaling		↑*DNMT3A*	↑*DNMT1A*, ↑*MBD3*,↑*Mi2*	↑*DNMT1A*, ↑*SAP30*
	Role of BRCA1 in DNA Damage Response			↑*BRCA1*	
	Telomerase Signaling			↑*DKC1*	
	Mismatch Repair in Eukaryotes			↑*Exo1*,↑*FEN1*, ↑*Polδ*,↑*PCNA*, ↑*MSH6*,↑*RPA*	
	Mitocondrial Dysfunction			↑*CYTC*,↑*GSR*, ↑*HtrA2*, ↑*ATP5G1*	
	Role of CHK Proteins in Cell Cycle Checkpoint Control			↑*Chk1*	↑*Chk1*
Enabled Replication Immortality	Telomerase Signaling			↑*DKC1*,↑*HSP90*, ↑*p23*	
Inducing New Blood Flow	VEGF Signaling			↑*VEGF*,↑*EIF*, ↑*RAS*	
	mTOR Signaling			↑*PPA2*,↑*elF4E*, ↑*elF4G*,↑*elF3*, ↑*40S*,↑*Ribosome*	
Deregulated Cellular Metabolism	Folate Biosynthesis			↑*DHFR*,↑*GART*, ↑*MTHFD1*, ↑*MTHFD1L*, ↑*MTHFD2*, ↑*PHGDH*, ↑*PSAT1*,↑*PSPH*,↑*SHMT1*, ↑*SHMT2*,↑*TYMS*	↑*GART*, ↑*MTHFD1*, ↑*SHMT2*,↑*TYMS*
	Cholesterol Biosynthesis			↑*ACAT2*,↑*FDPS*, ↑*FPS*,↑*LBR*	↑*DHCR24*, ↑*FDFT1*,↑*FDPS*, ↑*FPS*,↑*HMGCS2*,↑*IDI1*,↑*MVD*, ↑*SQLE*

↑: up-regulated, ↓: down-regulated.

## Data Availability

Not applicable.

## Supplementary Materials

The following supporting information can be downloaded at: https://www.mdpi.com/article/10.3390/ijms23063216/s1.

## Abbreviations

Bhas 42 CTA	Bhas 42 cell-transformation assay
OECD	Organization for Economic Cooperation and Development
NGTxC	Non-genotoxic carcinogen
TPA	12-*O*-Tetradecanoylphorbol-13-acetate
IATA	Integrated approaches for testing and assessment
DMSO	Dimethyl sulfoxide
FDR	False-discovery rate
GO	Gene Ontology
DAVID	Database for Annotation, Visualization, and Integrated Discovery
MIE	Molecular initiating event
FBS	Fetal bovine serum
