# Supplementary material for: Gene Expression over Time during Cell Transformation Due to Non-Genotoxic Carcinogen Treatment of Bhas 42 Cells
Source: Int J Mol Sci. 2022 Mar 16;23(6):3216. doi: 10.3390/ijms23063216 (PMC8954493; doi:10.3390/ijms23063216)
Supplement: Supplementary file 1 [file ijms-23-03216-s001.zip › ijms-1568087-supplementary.pdf]

Supplementary Materials

Gene Expression Over Time During Cell Transformation Due to Non-Genotoxic Carcinogen Treatment of Bhas 42 Cells

Kiyomi Ohmori [1, 2,*], Asuka Kamei [3], Yuki Watanabe [4] and Keiko Abe [3,5]

1 Chemical Division, Kanagawa Prefectural Institute of Public Health, Chigasaki 2530087, Kanagawa, Japan
2 Research Initiatives and Promotion Organization, Yokohama National University, Yokohama 2408501, Kanagawa, Japan
3 Group for Food Functionality Assessment, Kanagawa Institute of Industrial Science and Technology, Kawasaki 2100821, Kanagawa , Japan; kamei@kistec.jp
4 Health and Anti-Aging Project, Kanagawa Academy of Science and Technology, Kawasaki 2130012, Kanagawa, Japan; onuki.yuki0205@mail.u-tokyo.ac.jp
5 Department of Applied Biological Chemistry, Graduate School of Agricultural and Life Sciences, The University of Tokyo, Bunkyo-ku 1138657, Tokyo, Japan; aka7308@mail.ecc.u-tokyo.ac.jp
* Correspondence: ohmori.n4yf@pref.kanagawa.lg.jp or ohmori-kiyomi-kz@ynu.ac.jp; Tel./Fax: +81-046-783-4400 or +81-045-339-4448

Table of contents

Page	Content
2	Figure S1. Hierarchical clustering analysis
3	Table S2. Number of up- and down-regulated genes
4-5	Table S3-1. Gene symbol of Ben diagram FDR<0.05 Up-regulated
6-7	Table S3-2. Gene symbol of Ben diagram FDR<0.05 Down-regulated
8-9	Figure S4. Gene Ontology terms for selected genes. GO terms down-regulated due to TPA treatment for 1 h, 6 h, 24 h, and 8 days (C).
10-11	Figure S5. ILK signaling at 1 h
12-14	Figure S6. Molecular mechanism of cancer at 1 h
15-16	Figure S7. TNFR signaling
17-18	Figure S8. mTOR signaling at 1 h
19-20	Figure S9. NRF2-mediated oxidative stress response
21-23	Figure S10. Xenobiotic metabolism signaling
24-25	Figure S11. Integrin signaling at 1 h
26-29	Figure S12. Axonal guidance signaling at 6 h
30-31	Figure S13. ILK signaling at 6 h
32-34	Figure S14. Molecular mechanism of cancer at 6 h
35-36	Figure S15. IL-6 signaling at 6 h
37-38	Figure S16. Cell cycle control of chromosomal replication at 6 h
39-40	Figure S17. IL-1 signaling at 6 h
41-42	Figure S18. JAK/stat signaling at 6 h
43-44	Figure S19. Cell cycle control of chromosomal replication at 24 h
45-46	Figure S20. Mismatch repair in eukaryotes at 24 h
47-48	Figure S21. Role of BRCA1 in DNA damage response at 24 h
49-50	Figure S22. Hereditary breast cancer signaling at 24 h
51-52	Figure S23. Mitotic roles of polo-like kinase at 24 h
53-54	Figure S24. Role of CHK proteins in cell cycle checkpoint control at 24 h
55-56	Figure S25. GADD45 signaling at 24 h
57-58	Figure S26. Estrogen-mediated S-phase entry at 24 h
59-60	Figure S27. RAN signaling at 24 h
61-62	Figure S28. Sumoylation pathway at 24 h
63-64	Figure S29. p53 signaling at 8 days

Page	Content
65-66	Figure S30. DNA methylation and transcriptional repression signaling
67	Table S31. DNA methylation
68	Figure S32. Glutamyl cycle
69	Table S33. Gene expression variation associated with cytochromes P450
70-71	Figure S34. Prostanoid biosynthesis at 1 h
72-73	Figure S35. Hippo signaling at 1 h
74-75	Figure S36. HIPPO signaling at 6 h
76-77	Figure S37. Prostanoid biosynthesis at 6 h
78-79	Figure S38. Apoptosis signaling at 6 h
80-81	Figure S39. Gap junction signaling at 6 h
82-83	Figure S40. IL-2 signaling at 6 h
84-85	Figure S41. Interferon signaling at 6 h
86-87	Figure S42. PD-1, PD-L1 cancer immunotherapy pathway
88-89	Figure S43. Integrin signaling at 6 h
90-93	Figure S44. Aryl hydrocarbon receptor signaling at 6 h
94-95	Figure S45. Glioma invasiveness signaling at 6 h
96-97	Figure S46. Gap junction signaling at 24 h
98-99	Figure S47. Apoptosis signaling at 24 h
100-101	Figure S48. Cell cycle. G1/S checkpoint regulation at 24 h
102-103	Figure S49. Cell cycle. G2/M DNA damage checkpoint regulation at 24 h
104-105	Figure S50. VEGF signaling at 24 h
106-107	Figure S51. mTOR signaling at 24 h
108-109	Figure S52. Telomerase signaling at 24 h
110-114	Figure S53. Mitochondrial dysfunction at 24 h
115-116	Figure S54. Glioma invasiveness signaling at 24 h
117-118	Figure S55. Prostanoid Biosynthesis at 24 h and 8 days
119	Figure S56. SOX9 gene expression
120-122	Figure S57. Molecular mechanism of cancer at 24 h
123-125	Figure S58. Molecular mechanism of cancer at 8 days
126	Figure S59. Legend of Figure 8

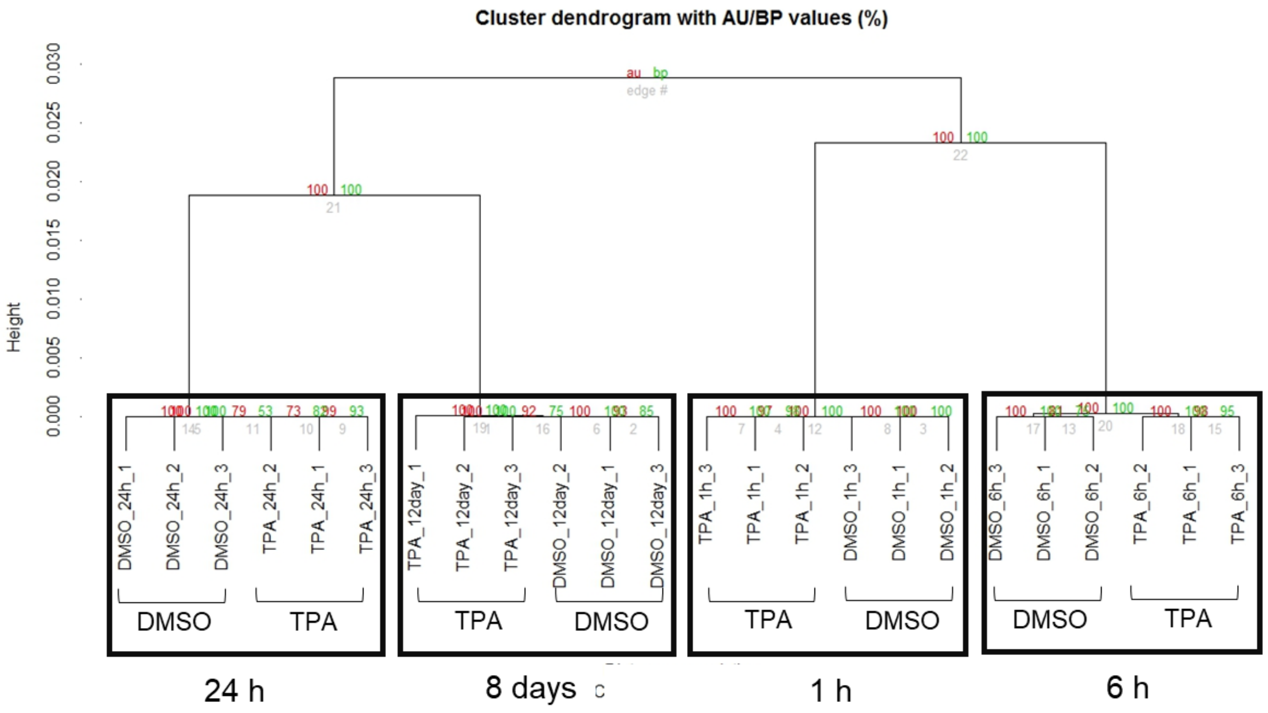

Figure S1. Hierarchical clustering analysis

Table S2. Number of up- and down-regulated genes

Processing time	Up-regulated	Down-regulated
1 h	324 (538)	527 (766)
6 h	763 (1211)	785 (1183)
24 h	784 (1039)	663 (931)
8 days	418 (595)	673 (983)

( ): probe sets

Table S3-1. Gene symbol of Ben diagram FDR<0.05 Up-regulated

Treatment time		Gene
1 hour & 6 hour & 24 hour & 8 day	A	Angptl4, Hmga1///Hmga1-rs1, Rbm3, Zwint, Mpp6, Slpi, Hmga2
1 hour & 6 hour & 24 hour	B	Slc20a1, Cxcl11, Slc25a33, Mapk6, Spred2
1 hour & 6 hour & 8 day	C	Bcl6, Trib1, 1810011O10Rik, Gla
1 hour & 24 hour & 8 day	D	Timp1, Aurka, Cdt1
6 hour & 24 hour & 8 day	E	Ngef, Pik3r5, Mtm1, Prkg2, 2310002L13Rik, Cebpd, Hivep3, Tnfrsf22///Tnfrsf23, Il13ra1, Skil
1 hour & 6 hour	F	Eya1, Ermp1, Cdkn1a, Krt16, Btg2, Dab2, Lhfpl2, Efna1, Trp53inp1, Mt1, Top1, Vps18, Flcn, Nfat5, Vegfa, Rsad2, Dnm3os, Spry2, Ptges, Sat1, Ankrd57, Sema4c, Psme4, Arl5b, Gabarapl1, Rab43, Egr2, Ddit4, 2410042D21Rik, Tgfb1, Plat, 1500032P08Rik, Bhlhe40, Tgif1, Hes1, Sqstm1, Purg, Srxn1, Cald1, Zmiz1, Egr3, Vps37b, Socs1, Arrdc3, Foxc2, Ccng2, Zfand2a, Dpp7, Sox9, Zfp217, Slc4a7, Adam8, 9530028C05, Smcr8, Atp6v1h, 9930031P18Rik, Kdm6b, 1700023H06Rik, Kdm3a, Ier3, Sema4b, Zfp36, Pnrc1, Hmox1, Bag3, Ndfip2, Stx3, Oaf, Bcl2l11, Nfkb2, Tnfsf9, Zswim6, Lpin1, Jun, Jmjd1c, BC022687, Traf1, B230312A22Rik, Impact, Irf2bp2, Dusp4, Tob2, D4Wsu53e, Nfe2l2, Ctsb, Ahcyl2
1 hour & 24 hour	G	Birc6, Zfp367, Gas2l3, E330016A19Rik, Cxcl10, Dnajb1, Gm16516, Gclc, Hnrnpab, Chac1, Ifrd1, Eif4e
1 hour & 8 day	H	Cenpl, Fam83d, Ccnf, Kifc1///LOC100044746, Junb, Nfkbiz
6 hour & 24 hour	I	Scd2, Slk, Ppm1l, Dpy19l1, AU018778, Ptpla, Ero1l, Expi, Pvr, Arl4c///LOC632433, Mgp
6 hour & 8 day	J	Fam19a5, Nav3, Tbx3, Gprc5b, Cytip, Klf5, Gdnf, Foxf2, Tnfrsf22, Bcl3, 6430527G18Rik, Btc, 4930523C07Rik, Pkig, Pcdh7, Tnfrsf23, Anxa8, Tnnt2, Tgfbi, Igf2bp2, Rnf150, Casp4, Gstt4, Nrn1, Fmnl3, Tes
24 hour & 8 day	K	Ifrd2, Nup50, Fam60a, St5, Lmnb2, Nsg1, Ctps, Mrto4, Utp20, Gm11428, Tagln2, Ddx21, Ppa1, Ccl7, Trmt61a, Apex1, Rcl1, Foxm1, Ppp1r14b, LOC100046344///Nme1, Ddx39, Prr11, Grwd1, Espl1, Hmgb3, Cdca5, Ipo5, Lrrc59, D17H6S56E-5, Gar1, Taf1d, Dlk2, Ldlr, Tacc3, Steap1, Hells, Bcat1, Racgap1, Man2a1, Timeless, Wdr77, St3gal5, Nop56, Rcc2, Lsm2, Serpina3g, Cad, Nup93, Suv39h2, 2700038G22Rik, Ttf2, Gas5///Snord47, Banf1, Ccnb1///Gm5593///Gm8416, Gpr176, Noc4l, Nucks1, Shb, Xpo5, Spc24, LOC100047155///Snrpa1, Shmt2, Cdk1, Mcm10, Cdca7, Stip1, Gpr137b///Gpr137b-ps///LOC100044979, Plk1, Lrp8, 4933413G19Rik///Foxm1///Pebp1, Cycs///Gm10053, Fam3c, Cdc20, Nubp1, Fkbp4, 2610019E17Rik, Mthfd1, Dnmt1, Arhgap22, Fabp5///Gm6166, Il1rl1, Tdrkh, LOC100046166///Tbrg4, Gins1, Spag5, Cdkn2aipnl, Gm5529///Slc25a5, Polr1a, Dtymk, Fabp5, Snhg1, Mcm6, Nhp2, Dph5, LOC100045677///Mcm3, Uhrf1, Ddx11, Osmr, Sfrs2, Rangap1, Col8a1, Cct3, Akr1b3, Runx1, Pdss1, Aurkb, Ppih, Aimp2, Ccrn4l///LOC100047134, Siglecg, Fosl1, 2610002D18Rik, Ivns1abp, Apitd1, Snhg3, Ppan, Cks1b, Rras2, Mcm2, Masp1, Eif5a, Chek1, Uck2, Lyar, Cp, Vars, Cdc6, Ncapd2, Matr3, Pfas, Ccl2, Rpf2, Rnaseh2a, Pola1, Gja1, Umps, Heatr1, Gmnn, Cep55, Cdca8, Kif22, Atp10a, Rcc1, Asf1b, LOC100045833///Ly6c1///Ly6c2, Tyms///Tyms-ps, Mcm4, Snrpa1, BC055324, Ccna2, Nasp, Wisp2, Cdca7l, Gart, Kif11, Nolc1, Kti12, Mcm7, Aspm, Ccdc86, Fbl, Exo1, Cdc34///LOC100046898, LOC100047340///Rcc2, Ube2s, Sephs2, Orc1l, Psmd13, Pdgfc, Dusp9, 1700029F09Rik, Timm8a1, Mcm5, Has2, Rrp12, Birc5, Kif23, Pkm2, Tmem97, Psme3, Prl2c2///Prl2c3///Prl2c4, Steap2, Anxa3, Fert2, Polr1b, Farp1, Fdps, Dctd
1 hour	L	Abi2, LOC100046186///Ramp3, Urgcp, AB041803, Bscl2, LOC269472, Rnf213, D430033H22Rik, Taf7, Baiap2, Nrp1, Bbc3, Mbd5, Ier2, Ints6, Spag9, Klf10, Nfkbia, Ssbp2, Rbbp6, Micall1, C330019G07Rik, Slit2, C130039O16Rik, 4833441D16Rik, Mcl1, Mnt, Ptprs, Chka, Narf, Irgm2, Rab11b, Mospd2, Hexim1, Mysm1, 1700109H08Rik, Pim3, BC049807, Usp32, Setd1b, Ndel1, Mad2l1bp, Fos, 1200015M12Rik///A130040M12Rik///E430024C06Rik, Ppp1r15a, Fam133b, Sertad1, Rbm43, Nab2, Insig1, Mx2, Dusp1, S100a10, 2900011L18Rik, Mylip, Sertad3, Trim25, Gdap10, Klf16, 130004C03, Irf1, Cyp1a1, LOC100270747, B430007K19Rik, Cks2, Whsc1l1, Mll2, 4921513D23Rik, Zc3hav1l, 5031426D15Rik, G2e3, Lpar2, Mxd1, Irs1, Cebpb, Dppa5a, Nipbl, Iigp1, 1810013L24Rik, LOC72520, Csrnp1, Tra2b, Zfp655, Fam196a, Cflar, 6330564D18Rik, Rtp4, Gm14446, Ipo7, Plekhm2, Mtdh, Emilin2, Mepce, Zfp52, D8Ertd56e, Rrad, Tcfap2a, 1190002F15Rik, 5830469G19Rik, Piga, Fosb, D13Ertd787e, Pdcd7, Fzd2, Mycn, Dclk1, Il11, 9930017N22Rik, C330016O10Rik, Hic2, Sec24a, Snai1, Rbm16, Gm2213///Gm8801///LOC677319///Ppp1r10, 6720403M19Rik, Rapgef6, Bach2, 1200003I10Rik///1200015M12Rik///A130040M12Rik///E430024C06Rik, Mbtd1, 6720460K10Rik, Pramef8, Nab1, Pex1, Pi4kb, Tmem55b, 2310016C08Rik, E130006N16Rik, Trim39, Trpm7, C77027, Terf2, Cdc42ep4, AU018740, Cyp1b1, Ahnak2, 1700056N10Rik, Tiparp, Saps3, Klf4, 2410131K14Rik, Klf11, Med13, Ttll3, Itpkc, Acsl1, A930017K11Rik, Fermt3, Atxn2///LOC100047323, Lcorl, Rnf169, Prkd2, 9330112F22Rik, Trex1, C80258, Pcf11, 2510017J16Rik, Egr1, D5Ertd121e, D5Wsu152e, Fam55c, Tmem140, Dusp2, Igf1r, Gm8801///LOC677319///Ppp1r10, AI845619, Ick, LOC74457, H2afx, Pttg1, Ddit3, D230019N24Rik, Per1, Dnajc1, 2210402A03Rik, Brd2, B930068K11Rik, Taf5, Zcchc11, 2610020C07Rik, Mdm2, Spry3, Ndufa12, Hist1h1c, Gpcpd1, Clk1, Tpp2, C530014P21Rik, B230334L07Rik, Ddhd1, Cdk12, Gspt1, Hjurp, Dnajb9, Depdc5, Mat2a, Mllt3, Cltc, Nudt7, Kcnq1ot1, D5Ertd798e, Itsn2
8 day	O	Cdca3, Myh1, Ep400, Fmn1, 2610034E01Rik, Tmem194, Foxp1, Cyp7b1, 2700094K13Rik, Gpr137b, Nkain1, Gm7788, Nek6, Wt1, Rnaseh2c, Mamdc2, Pnpt1, Tmem183a, Cfl1, Ly6e, Gpr137b///LOC100044979, Trim37, Kif20a, Idi1, Dcaf7, Pop1, Casp12, Baz1a, Cela1, Clca1///Clca2, Opa3, Bnc2, Pfkfb3, Diras2, Fam64a, 0610012H03Rik, Kif2c///LOC631653, Gpr137b///Gpr137b-ps, Lce1h, Hspa8///LOC624853///LOC641192, Gclm, Whsc1, Ccnb1, Gtse1, Hey1, Erap1, Apol6, Serpina3n, Akr1c18, Als2cr12, Lcn2, Cstf2, Slc15a2, Kif18b, Cacna1a, Gadd45g, Thg1l, Minpp1, Mvd, Sbno2, 2010317E24Rik, Gm8300, Usp12, Apobec1, Txn1, Gbp2, Ada, Il33, Gapdh///Gm10358///Gm2076///Gm2308///LOC100044981///LOC100045908///LOC100047352///LOC100048329, Fabp4, Sox5, Fdft1, Saa3, Hivep3///LOC100045240, 2610318N02Rik, Tubb3, BC005512///F630007L15Rik///Gm6958, Heg1, Car13, Ampd3, Cda, Plekho1, Mettl7b, Gm12816///Gm5879///Rpl3, Pkp3, Fam110a, Kif18a, Ly75, Phex, Lrrc4c, 2810004I08Rik, AA763515, Cyb561, Fosl2///LOC634417, Rasal2, D2Ertd750e, Wdr18, Megf6, Pde1a, Ube2cbp, 1810009A15Rik, Sqle, Med1, Acsl3, Lbp, Nt5dc2, Depdc1b, Dcps, Rtkn2, Susd1, Gm7040///Prl2c1///Prl2c2///Prl2c3///Prl2c4///Prl2c5, Rabif, 9630033F20Rik, Ccl8, Ccnb2, Socs3, Lce1i, Baalc, Anp32e, Fam54a, 2610036L11Rik, Podxl, Clip4, A630023A22Rik, Sfpq, Rtel1, Rbm38, Chi3l1, Pif1, Clca1, Lzic, Shroom3, 4931408D14Rik, Msln, Acpl2, Gsdmc2///Gsdmc4, Ube2t, Ccnb1///Gm4870///Gm5593///Gm8416///LOC635091, Orai2, Nek2, Pcdh19, Bub1b, BC048355, Ubqln4, Troap, Cpeb3, Tirap, Hp, BB287469///Eif1a///Gm2022///Gm4027///Gm5039///Gm5662///Gm5788///Gm6804///LOC641136, Sap30, Ly6a, 2810025M15Rik, LOC100046998///Opa1, Rbm14, Fgf23, Ccdc123, Stard4, Gpr137b-ps, Cenpa, Ifi204, Ubd, Gcnt2, Dhcr24, Pim1, Cytsb, Cdk2ap1///LOC100047490, Pmepa1, Il15, Tnfsf10, Sfxn5, Nav1, Actr3b, Slamf8, Fam164a

Table S3-1. Gene symbol of Ben diagram FDR<0.05 Up-regulated (continued)

Treatment time		Gene
6 hour	M	Btbd3, Frrs1///LOC100046401, Plin3, Vps26a, Tapbpl, Abcd4, Fam117a, Rhob, Dusp6, Tmem158, Pde10a, Bcr, Steap3, BC030336, Ggct, Ndrg1, Myo1b, Gad1, Klk7, Synpo, A930001N09Rik, Pqlc2, Tmed8, Mex3b, C78505, Dcn, Rtkn, Tcf4, Ppp4r2, Sox4, Stap2, Rnh1, Abhd1, Atp6v1d, Stk40, Klhl2, Ttc39c, Mpzl1, Klhl21, Sod2, Riok3, Bdkrb1, Mafg, Tmem65, Etv3, Smurf1, Map1lc3b, Adam19///LOC100045780, Hs1bp3, Ccl5, Cstb, Atp2b1, Trps1, Lrrc8d, Arl8b, Smox, Cdc42se1, Casp9, Adam17, Cep170, Pxdn, Jag1, Agpat9, Vgll4, Fam117b, Sgms2, Slc7a2, 1600029D21Rik, Tor3a, Smtn, LOC100047565///Mmd, Tnfaip6, Gm10397, Clcn5, Chic2, Ankrd37, Itga6, Tspan5, C3, Zfp703, Dusp16, Rgs17, Gigyf2, E130112L23Rik, Pnkd, Cdk20, Gsto2, Rasa2, Vasn, Mndal, Pde1b, Serpinb8, Atp6ap2, Adamts7, Litaf, Fgfrl1///LOC100046239, Cpeb4, C230081A13Rik, Pla2g7, Btg1///LOC100047353, Mpa2l, Robo1, Frmd4a, Dot1l, Mfsd11, Chmp1b, Bach1, Il2rg, Apeh, Nrip1, Tjp2, Snapc1, Kif3c, Parp8, Tgfb3, 1110018J18Rik, Reep3, Zfp326, Ypel5, Stxbp5, Tnfrsf1b, Fbxo11, Otud7b, Fyb, D8Ertd82e, 2610110G12Rik, Runx2, Rdh10, Dlgap4, Brwd3, Gna14, Adcy7, Ecm1, Ado, Procr, Igfbp4, LOC100048460///Lzts2, Ttyh2, Anxa7, Katnal1, Spred1, Purb, Stam2, Slc5a10, Pgm2, Afap1l2, Map3k11, Atp7a, Piwil4, Polr3g, Socs5, Tmc6, Stam, Slc6a8, Nfkbie, Ifit3, Tanc2, Adamts4, Akr1b8, Rassf8, Samd5, Cpt1a, Rb1, Ralgapa2, Phlda1, Tspan14, Nav2, Zdhhc18, Neu1, Fbxo33, Aff1, Plcd1, Crmp1, Cyp2j6, Fam53b, Jak2, Dsp, Capn5, Col18a1, Rsf1, Foxs1, Usp27x, Stard3, Ctsl, Plxnd1, Ercc1, Ssfa2, Hdac5, Cdkn2b, 9630019E01Rik, Sdcbp, Rnf149, Btg1, Hip1r, Akap13, Evi2a, Col5a3, Zfp239, Atrx, Ctns, Ostm1, Avl9, Csnk1g3, Ifi203, Synj2, Tspan12, Ifi205///Mnda, Ehd3, Endod1, Socs7, Ano6, Spib, Neo1, Atp6v0d1, Mras, Abca1, Iffo2, Camk2d, Plscr1, Klk8, Gramd1a, Ccdc88a, Pdpn, H60a, Fam126a, Mmp9, Arhgef2, Trib2, Serac1, Tob1, Fam20c, Fyn, Vat1, Fam63a, Slc41a2, 2610035D17Rik, Fgfr1op, Ppard, Fnip2, Klhl24, Casp3, Atp6v0b, Mmd, Fut8, Spry4, Fpgt, Pfkp, LOC100046168///Ndfip1, Grasp, Acsbg1, Stx6, Camsap1l1, Tnfaip8, LOC100044115///Rin2, Mfsd9, St3gal1, Plxnb1, Slc27a1, Gdpd5, Wwtr1, Slc22a4, Pdxk, 5930433N17Rik, Cdk5r1, Cyth2, Etv5, Etnk1, C1galt1, Ltbp3, Ypel2, Mapkbp1, Trim47, Fndc3a, Tubb2a, Slfn2, Ptpre, Hivep1, Abtb2, Il1rl2, Ptprj, Dip2c, Cdk17, Npc1, Pxn, Zcchc4, Lpgat1, Sbk1, Ttc39b, Map4k4, Alox5ap, Ogt, Ralgds, Antxr2, Nmnat2, Stom, Gnpda1, Hsd3b7, Osbpl8, Acox1, 5230400M03Rik, Obfc1, Map3k4, Agpat4, Slc11a1, Tsc22d1, Mxi1, Abcc4, Fchsd2, Megf10, Stxbp3a, Slc23a2, Mef2a, S1pr2, Capg, Emilin1, Spred3, Dmp1, Tollip, Atp6v1c1, Scd1, Trim8, Bcl10, Arl8a, Tmcc2, Sdc1, Prkx, Mlx, Renbp, Pi4k2b, Dyrk3, Rc3h1, Mmp10, 5330426P16Rik, Nrp2, Dagla, Tmem55a, Mmp13, Cmtm6, Gls, Sin3b, Ereg, Rab27b, Tmem2, Mtmr11, Rab32, 2700078E11Rik, Cblb, Gas7, Tnfaip2, Bnip3, Sh3pxd2a, Ankrd12, Pcdhb17, Ppp2r5a, Tead4, Spon2, Neat1, Cic, Peli2, Ly96, Klk6, Dock5, Myo10, Cd300lb, Trp53inp2, Ceacam1///Ceacam2, Etv4, Fgfr1, 1500031L02Rik, Il1rap, Hs6st1///LOC100047260, Zfand5, Znrf1, Plekhh3, Lif, Nudt9, Ttll5, 3110043O21Rik, Sik1, Gdpd1, Gsta1///Gsta2, Aebp2, Ifi203///Ifi204///Ifi205///LOC640890///Mnda///Mndal, Trio, Nfatc1, Znrf2, 4930402H24Rik, Hipk2, Zfp238, Ralb, Slc43a2, Irf2, Lypla2, Akirin1, Eda2r, Gna13, 5730559C18Rik, Snx16, Rhoq, Upp1, Nedd9, Hbp1, Tmbim1, Itga5, Tle3, Ceacam1, Hspb8, Zswim4, Epn2, Efhd2, Zeb1, Wwp2, Col17a1, Pgd, Pcmtd1, Angptl2, Dennd2a, Wipf1, Icam1, Tmem201, Sema6d, Mex3c, Gjb4, Chst11, Rap1b, Armcx1, Mospd1, Mex3d, Dgkd, Tsc1, Mrc1, Aqp3, Slc24a6, Inpp5f, LOC640502///Uap1, Cd164, Csrp2, Klf13, Pias4, Jhdm1d, Dusp5, Cgref1, Tacc2, Zc3h12c, Sh3pxd2b, Foxc1, Mblac2, Plin4, Rasa1, Ahr, Ptpn12, Slc36a1, 9030420J04Rik, Slfn4, Cd44, LOC100045795///Rsbn1, Rnf11, Mobkl1a, Ttll7, Tgm2, Gramd4, Ets2, Rai14, D030041H20Rik, Trim13, Gfod1, D930015E06Rik, Krt6a, Arhgef12, Abcb1a, B630005N14Rik, Tshz1, Esd, Pdp1, 0610031J06Rik, Apbb2, Pdgfb, Arntl, Mmp14, Mpz, Gsta2, Abr, Pold4, Ino80c, Rreb1, Jarid2, Ptprn, Uap1l1, Hsd17b11, Acvr1, Dnajb4, Thbs2, Tnip1, Efna4, Smad3, Sipa1l2, Chml, Cmtm3///LOC100046883, Sh3gl3, Sesn3, Gm16515, Furin, Spsb1, Slc39a14, Wbp2, Sestd1, LOC100047588///Mark3, Schip1, Fam129b, Gm10307///Paox, LOC14210, Samd8, Map4k3, Arhgap17, Flrt3, Il6ra, Gramd1b, 2210008F06Rik, Abca5, Trak2, 6330577E15Rik, Arhgef3, Plbd2, Ing1, Rc3h2, Fam69a, Caprin2, Inpp1, Slc2a1, Gpc1, Ezr, Dync1li1, Lrig1, Ifngr2, Atp6v1b2, Cttnbp2nl, Relb, Dhps, Slc25a37, AI607873, Syne2, Rnf19b, Mast4, Irx1, Fam176a, Myo1e, Mgat5, Rab31, Inhba, Mll5, Fndc3b, Kcnn4, Cd9, Ern1, Gm22, 4732423E21Rik, Serinc5, Il18rap, Fam100b, Igsf3, Cd1d1, Eaf1, Pip4k2c, Hivep2, Gsto1, Mfap3l, Hk1, Pion, Clcf1, Mylk2, Klrb1a, 5031439G07Rik, C330006P03Rik///Homer1, Serpinb2, Zfp608, Itgb3, Parvb, Tnc, Asap2, Phldb1, Chic1, Spire1, BC028528, Pik3cd, Clic1, Stat3, Sema7a, Padi2, Abhd5, Stx11, Stat5a, Ank, Mx1, Dnmt3a, Sgtb, Enpp2, Gm8615///Gnpda1, Map2k6, St6gal1, Uhrf1bp1l, Slc41a1, Tspan11, Acvr1b, Cxcr7, Clock, Fnip1, Leprotl1, Mfsd1, 2900064B18Rik, 5830408B19Rik, Zfc3h1, Rfxank, Sept9, C76876
24 hour	N	Dhx9, Srrm1, Ruvbl2, 8430406I07Rik, Dnajc21, Wdr33, Rif1, Rbm10, Strap, Cenpn, Hat1, Parp1, Stat2, Cenph, Tpx2, Sh3kbp1, Psph, Sdad1, Nutf2, Plcxd2, Lsg1, Rad54l, Bop1, Hdgf, Immt, Dnttip2, Kat2a, Dis3, Gm7669///Gm7901///Gm9347///Phgdh, Tmed2, Bbx, Utrn, Eif4e2, Trip13, Pole3, Wdr75, Cdk6, Tubb6, Ewsr1, Topbp1, Asap1, Prpf4, Exosc1, Tcerg1, Lpp, Rad23a, 4922501C03Rik, Dtl, E130303B06Rik, Glce, Fhl1, Fus, Gbf1, Hbegf, Eif3c, Atad5, Mre11a, Eftud2, Polr2h, Mbd3, Malat1, Myc, Gm5256///Gm5529///Slc25a5, Prmt1, Gins3, Fgf7, C1qbp, Gm9790///Higd1a///LOC100045763, Cggbp1, Shmt1, Hsp90b1, Nudc, Dnajc5, Ndufaf2, Cdc25a, H2-Ke2, Tpm3, Eif2s1, Tomm40, Cct6a, Pold2, LOC100046672///Wars, Scarb1, Polr3e, 1810029B16Rik, Plaur, Trp53, Rfc4, Prpf40a, Rnf168, Gm10171///Timm23, Casc5, Myst3, Fignl1, Fancd2, Metrn, Acat2///Acat3, Ran, Glb1, Brp16, Eri1, Usp39, Klf7, Rad51, Prpf19, Idh3a, Rangrf, Pnn, Atic, Hspa4, Tbl3, Polr1e, Nup133, Gm6742///Gm7993///Gm9115///LOC677259///Odc1, Sfxn2, Tmpo, Eif3a, Clspn, Mthfd1l, Pola2, Qtrt1, Gm5081///Prps1, Eno1, Gars, Got1, Csf1, Phf10, E2f8, Smc6, Tbl1x, Ebna1bp2, Aars///Exosc6, Mett10d, Ndufaf4, Ilf3, Gjc1, Iqgap3, 2210411K11Rik, Cyc1, Cntf///Zfp91///Zfp91-Cntf, Ung, Ssb, Ccnd1, Car6, Tmem48, Usp36, Xrcc6, Zwilch, Eif1ad, F730047E07Rik, Nsun2, Naa38, Ncl, Uxs1, Mlf1ip, Exosc7, Rrp1, Thrap3, Gm6742///Gm7450///Gm7993///Gm9115///Odc1, Pbk, Ldha, Dnajc2, Lig1, Chaf1b, Msh6, Nkrf, Setd8, Haus6, P4ha1, Cdca2, Timm9, Hsd17b12, Nup85, Ttc27, Gm10043///Gm2926///Lsm6, Ces1, Prim1, Eif6, Galk1, Rrm2, Lamc1, LOC100047619///Slc7a5, Bclaf1, Dkc1, Ddx46, Fanca, Gtf2f2, Syncrip, Kri1, Wdhd1, Abcf2, Nudt19, Nrm, Sgol1, Rfc3, Dynll2, Mki67ip, Sigmar1, Pwp2, Farsa, Farsb, Cbx5, Azin1, Luc7l, Pitpnb, Wdr12, Ddx56, Snrpb, Il6, Nup205, Cct7, Eef1e1, Kpna3, D19Bwg1357e, LOC639633///Npm3///Npm3-ps1, Eif1a, Skp2, Ppil5, Wbp11, Tcf19, Alkbh2, Cycs, Mobkl1b, Cdca4, Cyb5b, Ciapin1, Dut, Ddx18, Htra2, Wdr36, Plk4, Sfrs13a, Chchd4, Nsl1, Phf15, Nars, Exosc5, Cdc45, Eps8, Ppat, Pgp, Gm7901///Gm8096///Gm8341///Gm9252///Phgdh, Iars, Kntc1, Rad18, Ncbp1, Slc25a5, Gm11276///Hist1h2ad///Hist1h2ao, Naf1, Prmt3, Thoc4, Wdr43, Rrp15, Rfc1, Dna2, Ltbp1, Fam111a, Dctpp1, Haus1, Rfc5, Wisp1, Rsl1d1, Ube2c, Tomm70a, Tk1, Taf4b, Tardbp, Cth, Mybbp1a, Tnfrsf12a, Aatf, Erdr1, Fndc4, Nop16, Abce1, Pcna, Fam195a, Gcn1l1, Snrpd1, Chd4, Alad///LOC100046072, Thyn1, Nip7, Gm10333///Gm10349///Nutf2, Yrdc, Naa50, Etf1, Hspa14, Trmt6, Kif20b, Slc20a2, Gins2, Usp1, Znhit6, Set, Pno1, Ddx10, Hsph1, Nup62, Bzw1, Eif2b3, Ccdc101///LOC100047712, Prps1, 6430706D22Rik///Hjurp, Ttll1, Top2a, Csda, 1300001I01Rik, Clip1, Nup43, Tsr1, Pycr2, Gstcd, Pdap1, Ftsj3, Zc3h15, Clns1a, Gsg2, Tpm4, Cenpf, Naa15, Traip, Cenpi, Lamb1-1, Gadd45a, Cse1l, Nop2, Recql4, S100a3, Bysl, Pank3, Cklf, LOC100047009///Pus7, Efnb2, Ugcg, Pa2g4, Exosc2, Ube2g2, Magi3, Diap3, Kras, Ddx27, Trim59, Ddah1, Gtpbp4, 1110034A24Rik, Ergic1, Mecr, Loxl4, 2810417H13Rik, Rsrc2, Nhp2l1, Pole2, Nomo1, Mrpl9, Fkbp5, Orc2l, Suv39h1, Hars, Snrpd3, Fancb, Dhfr, Kif2a, Eif4g1, Eif3b, 2610507B11Rik, Tm4sf1, E2f7, Slfn9, Pus1, Baz1b, Aqp1, Zfp428, Rpl41, Smarcc1, Mphosph10, Eps8///LOC632638, 3110082I17Rik, Wdr46, Ppil1, Cdv3, Setd5, Ccne2, Sf3a3, Taf9, Pkmyt1, Aacs, Phb, Supt16h, Aen, Eme1, Prkar2a, Nol9, Cdc7, Slc35a3, Atad3a, Gemin5, Gemin6, Pgk1, Abcf1, Lbr, Dscc1, Mettl6, Gm11847///Gm6793///Hnrnpa3///LOC100045099, Otud4, Mars, Ola1, Pole, Rpa1, Eno1///Gm5506///Gm5855, Txnip, Fbxo5, Kpnb1, Nup160, Gm3223///Nudc///Nudc-ps1, Prim2, Pold1, Rae1, Smc1a, Nudt21, Exosc8, Got2, Pprc1, Klhdc4, Uchl3///Uchl4, Xpot, Gas5, LOC100048559///Sfrs1, Prep, Prmt7, Nol11, Pwp1, Ctu2, Cdk14, Mak16, Me2, Orc6l, Ehd4, Rrp9, Atp5g1, Lsm6, Rps24, Psma7, Snrnp70, Sumo3, Sae1, Ankrd1, Dck, Mthfd2, Erh, Myo1c, Psmc5, Ilf2, Hnrnpr, Rrs1, Atad2, Ptges3, Spop, Pgam1, Hspd1, Nup54, Rbl1, Phgdh, Rad51ap1, Hirip3, Cars, Nup107, Gpatch4, Brca1, Uchl5, Ptbp1, Utp18, Ltv1, LOC100047481///Sec24b, Tfdp1, 2810408I11Rik, Pak1ip1, Safb2, Nap1l1, Rpa2, Nop58, Zfp91, Ggta1, Refbp2///Thoc4, Chtf18, Ncapg, Cttn, Mybl2, Pcgf6, Ints7, Ccne1, Smc2, Tcof1, Noc2l, Wdr74, Bzw2, Adsl, Gnl3, Fen1, Ckap4, Sf3b2, Ccdc99, Ubtf, Enoph1, Ranbp1, Macrod1, Slc19a1, 4632434I11Rik, Coro1c, Ak2///LOC100047005, Hnrnpu, Sssca1, Rrm1, Pkp4, Cisd1, Incenp, Tubg1, Wars, Zmynd19, Hmmr, LOC630729, Hsp90aa1, Slc16a1, Ssrp1, Slc25a30, Chd1, Dcaf15, Mrpl22, Tssc4, Fam20b, Brip1, Eno1///Gm5506///LOC100047043, Aars, Alg3, Nuf2, Amd1, Psat1, Nxt1, Ide, Rpl12, Elovl6, Mad2l1, Srm, Sart3, Mtbp, Tipin, Magohb, Trib3

Table S3-2. Gene symbol of Ben diagram FDR<0.05 Down-regulated

Treatment time		Gene
1 hour & 6 hour & 24 hour & 8 day	A	Maf, Akap12, Thbs1, Ptn, Nfib, Tpm1
1 hour & 6 hour & 24 hour	B	Cyr61, Ptger4, Plekhf1, Col1a2, 9930013L23Rik, Cxcl5, Cited2, Cxcl1
1 hour & 6 hour & 8 day	C	Arhgap29, Ptprk, Ldb3, Snai2, 9930012K11Rik, Sdpr, Acta2, Klf6, Actg2, Epha4, Bcl2, Ptgs2, LOC640441/// Thbs1, Epha2, Larp1b
1 hour & 24 hour & 8 day	D	A930005H10Rik, Sfrs11, Pdgfa, Hectd2, Jhdm1d, Spnb2, Fzd7, Araf, Rgs3, Abtb2, Anxa6, Sntb2, Enc1
6 hour & 24 hour & 8 day	E	Islr, Thbd, Zhx1, Dusp1, Nfia, Vdr, Depdc6, Postn, Nr3c1, Mapre2, Sema3c, Gm2115, Col3a1, Pitpnc1, Npnt, Dlc1, Fam114a1, Ebf1, Col5a1, Aoc3, Ptx3, Fstl1, Ehbp1, Cd248, Col5a2, Il17rd, Ankrd50, Ncam1, Col1a1, Tcf7l2, Peg3, Sorbs1
1 hour & 6 hour	F	Mbnl1, Ctps, Plcxd2, Edn1, Ppp1r12b, Akap1, Ccl7, Asap1, Phf17, Nexn, Klf7, Pnn, Dlx2, Phldb2, LOC100046232/// Nfil3, 6330406I15Rik, Prkab2, F2r, Gadd45g, Tmem119, 9530009M10Rik, Bpgm, Camk2g/// LOC100045547, Pdlim5, Zcchc2, Fhl2, Vgll3, Luzp1, Bdnf, Id2, Palm2, Ampd3, Lcp1, Dph5, Mettl7b, Crim1, Ccrn4l/// LOC100047134, Fn1, Ccdc85b, Lats2, Loxl4, Prss23, Rhobtb3, Myh9, Stambpl1, Zzef1, Plscr2, Srf, Amotl2, Osr1, Tgfb1i1, Creb3l1, Irak2, Myo1c, Dusp8, Tsku, Atoh8, Kctd10, 2610203C20Rik, Plk2, Rnd3, Nuak1, Grem1, 8430408G22Rik, Csrp1, Cbx4, Jub, Acta1, 1110067D22Rik, Hoxa1, Fam107b, Prkar2b, Gadd45b, Nr2f2, Filip1l, Fhl1, Epb4.1l2, Fgf7, Diras2, Smad6, Swap70, Flnb, Dok1, Apln, Rab23, S1pr1, Csf1, Thy1, Ccl9, Htra1, Gvin1, 2810026P18Rik, Syde2, Serpine1, Hoxc13, Irs2, Chst1, Lasp1, Wisp1, Nuak2, Fscn1, Ccl17, Myl9, 5430416N02Rik, Sgms1, Adamts1, Igsf9, Pkdcc, Fst, Glipr2, Palld, R3hdm1, Ankrd1, Cnst, Kctd5, Fbln2, F3, Msrb3, Klhdc8a, Fblim1, Vcl, Enah, Rnd1
1 hour & 24 hour	G	Sox4, Il1r1, Wdfy1, Hoxa2, Snx18, Atrx, D530037H12Rik, 5430434G16Rik, Flrt3, Zfp40, Tnc, Prrx1, Zfp383, Med13l, Klf9, Tnrc6c, Krit1, Fam43a, LOC100045546, BC018101/// Zfp97, Brwd1
1 hour & 8 day	H	Mex3b, Six1, Ehbp1l1, Cx3cl1, Rab2b, Cyp26b1, Cdc42ep3, Pik3r1, Rcan1, Rgs16, Cald1, Tgfb2, Nedd9, Pdgfb, Slmap, Lancl3, 3110027N22Rik, Casc4, Frmd6, Grhl1/// LOC100043996, Zfx, Secisbp2l, C130026I21Rik/// LOC100041885, Sike1, Pdp1, Frk, Marveld1
6 hour & 24 hour	I	Arrdc4, Akr1c14, Olfml2b, Fam5c, Amot, Ephx1, Lox, Kdelr3, Ttc28, Sec16b, Gulp1, Fat4, Mprip, Gm13305/// Gm2002/// Il11ra1/// Il11ra2, Prrx2, Fgfr2, Slc9a3r1, Synm, Glrx, Ccdc80
6 hour & 8 day	J	Loxl1, Grk5, Nrp1, Zfp521, Atp1b1, Fam198b, 1190002N15Rik/// LOC100044725, Pcgf5, Ablim1, Tsen2, Ror1, Igf1, Bend5, Igfbp7, Bach2, Tgfbr2, Wnt5a, Runx1t1, Rps6ka5
24 hour & 8 day	K	Plekhg2, Ptplb, Tle2, Gdpd2, Fam117a, Tmem158, Cbr2, Tmem53, Dcn, LOC100047214/// Pink1, Trps1, Jag1, Malat1, Rusc1, Tmem106b, Mrc2, Aif1, Zeb2, N4bp2l1, A530047J11Rik, Cpeb4, Cdkl2, Carns1, Col6a1, Arvcf, Ypel5, Slc12a5, Prickle2, LOC100046560/// Maged2, Timp4, Ghr, Alx3, Gpc6, Enpp3, Adamts4, Slc15a2, Cldn19, Ccdc28b, Trim63, Boc, 2810055G20Rik, Fcgrt, Ddx26b, S100a16, Naip5, Klhl24, Amotl1, Ypel1, St3gal1, Aspn, Bace1, Ypel2, Appl2, Svep1, Ctf1, Atl1, Zscan21, Pfkfb2, 2210023G05Rik, Lipa, Lgals7, Cnksr3, Pdcd4, Rbms3, Egr3, Csad, Sncg, 2410066E13Rik, Col6a2, Trp53inp2, Sema3g, Lyz1, Ldlrad3, Gsta1/// Gsta2, 9630013D21Rik, Cib2, Gsta4, Klf11, Naalad2, Prokr1, Pbxip1, Tmem195, Slc45a4, Capn1, Ccl27a/// Gm13306, Dhrs3, Tnfsf12/// Tnfsf12-Tnfsf13/// Tnfsf13, Calml4, Egr1, S100b, Kitl, Matn2, Dlk1, Prelp, Sema3a, Tanc1, Socs2, Fetub, 6230424C14Rik, Igf2r, Pyroxd2, Gpcpd1, Cacna1g, Crlf1, Reck, Nr4a1, Mettl7a1, Bdh2, Irf2bp2, Fam82b, Lyz2, Kalrn, Sepp1, Ces3, Dleu2, Mmp2, Eya1, Cmbl, Zmat1, Ndrg1, Fbxo2, Lrp1, AU040829, Ppap2b, Slc9a3r2, Mxd4, BC031353, Marcks, Rnasel, Gpr88, Dact1, Vasn, Srgap3, C230081A13Rik, Robo1, Trp53inp1, Adamts12, Sned1, Nrbp2, Rab3il1, Slit3, Ggt5, Agpat5, Plscr4, Sorbs2, Neu1, Wipi1, Rnase4, Cabc1, Ptges, Plxnd1, Mylk, Aldh6a1, 9630025I21Rik, Gsn, 1110003E01Rik, Ppp1r3c, Olfm1, Caps2, 2310057J16Rik, Ube2h, Antxr1, Pgap1, LOC552880, Col4a1, Arnt2, Mtap2, Nmnat2, Stom, Srr, Sparcl1, Cfh/// LOC100048018, Npr2, Ift122, Lst1, Gm3932, Fmod, Ogn, Lrrc17, Col27a1, Tmem87b, S100a1, Higd1c/// Mettl7a1/// Mettl7a2, Serf2, Osr2, Dmxl2, Kdm6b, Pik3ip1, Mmp14, Scn2a1, Pcdhb9, Ankrd24, Fkbp7, B3gnt9, Emp2, Scara5, Lum, Ncor1, Tcp11l2, Clu/// LOC100046120, Ppargc1a, Cd1d1, Naga, Slc5a3, Clec11a, Apod, Ccl27a, BC028528, Dnmt3a, Enpp2, Gm8615/// Gnpda1, Bmf, Agt
1 hour	L	Gpr137b, Fam60a, Dusp6, Kpna1, Irf5, Zfp58, Acsl4, Aftph, Arf2, Utrn, LOC100216343, 5430404G13Rik, Dyrk2/// LOC100044376, 4921524J17Rik, Hbegf, Zswim1, Gigyf2, Gmfb, Eif2a, Sh3bp4, Lamc2, Fas, Rnf166, Ctgf, Ppm1a, Pls3, Rdh10, Kctd15, A630033H20Rik, Psmc6, Nppb, Gas5/// Snord47, Prepl, Nav2, Cux1, 9330156P08Rik, Tjap1, Prmt6, Nipa2, Pdcl3, 9130008F23Rik, 2610005L07Rik/// 6820431F20Rik, Mfsd2a, Slco4a1, Itih3, Plscr1, Trib2, Tln1, Fbxo30, Glipr1, Tuft1, Pfkp, Snhg1, Cwc22, Ctu1, Fam108c, Lima1, Zfp451, Lrrc8c, Maff, Gm4455, Ggnbp2, Tslp, Slc30a4, Twist1, Lbh, Clip1, Zfp281, Sept7, Zbtb46, Tmem123, Dub1, Efnb2, Cd300lb, Socs3, Armc8, Aebp2, Mapk14, Rhoj, Plekho2, AU014972, Fam118a, Six4, Rod1, AU021760, Rap2b, Micall2, Zfa/// Zfx, Epn2, Gorab, Fermt2, Lfng, Tgfbi, LOC100044953/// Ppp1cb, Krt80, Ets1, LOC100045795/// Rsbn1, 9830001H06Rik, Hivep3, Rai14, Orai2, Rad21, Snx13, Zfp748, Gm3426, Rnft1, 1110034B05Rik, Per2, Rrs1, Tpbg, Trit1, LOC100047481/// Sec24b, Spata5, Eif2ak4, S1pr3, Zxdc, Fam176a, Cd47, Inhba, Gm22, Pawr, Zswim6, Smad1, Gpr137b-ps, Has2, Cspp1, 4932431P20Rik/// Gm12587/// Gm13932/// Gm2509/// Gm2676/// Gm3307/// Gm3462/// Gm3899/// Gm4587/// Gm5473/// Gm5842/// Gm5853/// Gm5937/// Gm7890/// Hmg1l1/// Hmgb1/// Hmgb1l/// LOC100045876/// LOC637733/// LOC674543, Zfp422-rs1, Net1, Gcnt2, Zfp295, Myl6, Sema7a, 9630050E16Rik, Arid4b, Edn2, Trove2, Cxcr7, Zfp644, Appbp2, Prkaa1, Sh3kbp1, Cdc42ep1, 6820431F20Rik, Rprd2, Sdc4, Jrk, Gpr137b/// LOC100044979, Atp2b1, Zfp105, Clasp1, 2310044G17Rik, Bnc2, 4632415L05Rik, Ppp3r1, Fosl2, Plaur, Tjp2, Dusp10, Plau, D8Ertd82e, Fam126b, 100041874/// ENSMUSG00000063277/// ENSMUSG00000068790/// Gm10409/// Gm2897/// Gm3002/// Gm3194/// Gm3696/// Gm3703/// Gm8108/// Gm8206/// Gm8348/// LOC100036568/// LOC100041151/// LOC671957, Prkg2, Tle4, Wdr1, Traf5, Coq10b, Ndufaf4, Ddr1, Ccdc76, Tbc1d1, 2610030H06Rik, Rassf8, Zcchc10, LOC675567/// Rccd1, Tmem70, Aff1, 4930426L09Rik, Gpr137b/// Gpr137b-ps/// LOC100044979, Cldn12, Ndst2, Rufy3, Ccl20, Hif1a, Crk, Arhgap22, Syncrip, 2010109K09Rik, Zfp664, Il6, Cnbp, Ube2f, C80998, Rin1, Rnf19a, Rassf1, Lrrfip1, Jmjd4, A430108E01Rik, Zfp800, Tnfaip3, Ptpn2, Abcc1, B130052P14Rik, Rasl11b, 4930523C07Rik, Ddhd2, Gm12816/// Gm5879/// Rpl3, Hivep1, Gata3, Ankrd28, Cdk17, Map2k4, Pard6b, Itprip, Akap2, Osbpl8, Kif5b, Cobll1, Eif2s2/// Gm9892/// LOC100046208, Zfp867, Celf4, Cbx8, Anxa1, Rapgef6, Fam46b, Nup98, Gadd45a, Cdc42ep2, Ppp1r12a, Npas4, Dstn, 100041874/// ENSMUSG00000063277/// ENSMUSG00000068790/// Gm10406/// Gm2897/// Gm3194/// Gm3696/// Gm8108/// Gm8206/// LOC100036568/// LOC100041151/// LOC671957, 5033430I15Rik, Tlk1, Lce1f, Bag5/// LOC100047042, Gtpbp4, Cp, LOC100045866/// Tceb1, Bag2, Trio, Nfatc1, Hipk2, Zfp238, Denr, Tceb1, LOC100044968, Ube2w, Phf3, Vcam1, LOC676546/// Mmd, 2410002O22Rik/// LOC100044479, Wwp2, Arid5b/// LOC100044968, Ier5l, Setd5, Id4/// LOC100045546, Huwe1, Zfp697, Gm6245/// Ubxn2a, Zfml, Foxc1, Tlr4, Cd44, Ppfia1, Pde4dip, Acpl2, Ddx28, Casp4, Aasdhppt, Gas5, Zfp719, Trim16, Card10, 5430414B19Rik, Bok, Rps24, Acvr1, Rps9, Arid5b, Pdlim7, Tank, 1700109F18Rik, Schip1, Tpcn2, Adm, Sertad4, Tlk2, Zfp568, Mgp, Cttnbp2nl, Stxbp1, Oxct1, Myo1e, Ubtf, 9530006C21Rik, Hivep2, Usp53, Hspa1b, Rcor1, C330006P03Rik/// Homer1, Lactb, 4930422N03Rik, Kpna4, Cytsb, Rnf160, Pmepa1, Zbtb24, 2010111I01Rik, Fgd3, Bcar1, Csrnp2

Table S3-2. Gene symbol of Ben diagram FDR<0.05 Down-regulated (continued)

Treatment time		Gene
6 hour	M	Mtmr10, Cd3eap, Ifrd2, 8430406I07Rik, Pkia, Bcl7a, Cenpn, Parp1, Cenph, Nsg1, Rad54l, Chst12, Ebf3, Slc39a13, Snx24, Tcfap4, LOC100048247, Pole3, Metrnl, Cdon, Accn2, Topbp1, Chek2, Ly6e, Mdc1, Zcchc24, E130303B06Rik, Idi1, Gbf1, Atad5, Sh3d19, Ccdc134, Rrp1b, Myc, Fam110b, Casp12, Fat1, Fez2, Dclre1b, Nmral1, Polh, Cdc25a, Zfp472, Lipt1, Fam64a, Nrg1, Tmtc4, Aaas, Pdlim2, Pim3, BC049807, Rfc4, Rnf168, Metrn, Racgap1, Acat2/// Acat3, Mtm1, Creld2, Timeless, Rad51, Fbn1, Col12a1, Gtse1, Nop56, Atic, Dhrs13, Pcdh18, Pmp22, Gne, Haus7/// LOC100044098, Pola2, Fam171a2, Dimt1, Smc6, Ttf2, Dhodh, AI452195, Aars/// Exosc6, Prkci, Tfb1m, Flrt2, Ncapd3, 2310061C15Rik, Tnk2, Cdk1, 2610039C10Rik, Xrcc6, 2900073G15Rik, Mcm10, D13Ertd37e, F730047E07Rik, Cep68, Bicc1, Cdca7, Pbk, Dhcr7, Lrp8, Lig1, Chaf1b, Msh6, Haus6, Cdca2, Snap47, Dusp12, Atl3, Figf, BC049349, Ces1, Xrcc6bp1, Rrm2, Nsdhl, Pank1, Dnmt1, Pak3, Fanca, Wdhd1, Nrm, Eid1, Shisa4, Apcdd1, En1, Pak1, Cdkn2aipnl, Wwc2, Brca2, C79407, Ppil5, Wbp11, Tcf19, Fabp5, Zfp354c, Smarcd3, A930004D18Rik, Plk4, Grb14, Rab30, Fads2, Mcm6, Cdc45, Ppbp, Zfp81, Mvk, Rad18, Uhrf1, Pnpla7, Gm11276/// Hist1h2ad/// Hist1h2ao, 4930422G04Rik, Wwc1, Gen1, 1600002H07Rik, Dna2, Ddb2, Tk1, Col8a1, Rad51c, Cth, Fndc4, Fzd2, Isoc1, Fam195a, Alad/// LOC100046072, Psrc1, Slc1a3, Pdss1, Gpr124, Blm, Hdlbp, Plxna2, Zfpm2, Prps1, Lsm3, Fkbp11, Sac3d1, Apitd1, Snhg3, Gsg2, Ube2cbp, Nek7, Traip, Arhgap11a, Cenpi, Zfp39, Ercc5, Tgtp1/// Tgtp2, Recql, Gbp6, 9430037O13Rik, Mcm2, Tlcd1, Exosc2, Chek1, Cyp1b1, Trim59, Ppap2a, Ptpn21, Esco2, Epb4.1l3, Tspan4, Esyt2, 2610036L11Rik, Matr3, Tyms, Manf, Slc19a2, Fam122b, Zfp809, Orc2l, Ifit1, Pvrl2, Aqp5/// LOC100046616, E2f7, Slfn9, Baz1b, 6820445E23Rik, Ccdc102a, Gja1, Umps, Mycbp, Stil, Gpsm2, LOC100048247/// Pcgf5, Ccne2, Gli2, Eno3, Gmnn, Dlx1, Rassf7, Slc6a6, Atp10a, Rcc1, Asf1b, Dscc1, Csf1r, Zfp503, Txnip, Osbpl3, Nfu1, Ephb2, Sdf2l1, Rasl11a, Dck, Nolc1, Nfkbil2, Atad2, Igf1r, Zranb3, Mcm7, 6030445D17Rik, Rad51ap1, Nup107, Polk, Cdt1, Exo1, Rhpn2, Cdr2, Ifi47, 2810408I11Rik, Ncapg, Palb2, Ly6a, Acat2, Smc2, Tpst2, Fen1, Fpgs, Orc1l, Neil3, Nme4, Pdgfc, Stard4, Zmynd19, Ehd2, Mcm5, Msh2, Fam98a, Birc5, Dse, Tssc4, 1700040I03Rik, Dusp14, Brip1, Mllt3, March5, Impa2, Nxt1, 9230114K14Rik, Lin9, Mlec, Anxa3, Tsc22d2, Copz2, Armcx4, Magohb, Pxk, Zmym1, 1700113I22Rik, Fmn1, Ncapg2, Unc13c, 5430417L22Rik, Zfp367, Rfc2, Bop1, Ssbp3, Pms2, Tbl1xr1, Trip13, Abcg2, Ect2, Mxra7, Ubr7, Ckap2l, 4933404O12Rik, Lpp, Cirbp, Arl4d, Dtl, Slit2, Rnpep, Greb1l, Fam33a, Fads3, Gins3, Hsp90b1, Cdca5, D17H6S56E-5, Tmem109, Dlk2, Fam13c, Wee1, Pold2, Pvt1, Steap1, Zfp101, Hells, Casc5, Fignl1, Zfp692, Prr5l, Cand2, Ebp, Rnf26, LOC676549/// Siva1, Ptplad2, Loxl3, Maoa, Haus4, Tmpo, Ppp1r15a, Clspn, Samd4, Suv39h2, Lmnb1, Gm5081/// Prps1, U90926, Peg12, Ccng1, E2f8, Phyhipl, Rsu1, Tbl1x, Lass4, Shb, Myadm, Spc24, Ung, Cacna1a, Apaf1, Sh3bp5, Zwilch, Ski, Gpr180, Mum1l1, 4933413G19Rik/// Foxm1/// Pebp1, Tmem41a, Cpox, Mvd, Wdr90, E2f1, Nup85, Fkbp4, Prim1, Unc5b, 1810032O08Rik, Morc4, LOC100047619/// Slc7a5, Rhod, Dkc1, Tmem107, Fabp5/// Gm6166, Tdrkh, Leprel1, Anln, Sgol1, Rfc3, 2410002F23Rik, Irs1, Slc44a2, Mecom, Gins1, Elf2, Itsn1, Mnat1, Skp2, Gpr85, Alkbh2, Scfd2, Phlpp1, Dut, Dnajc9, Htra2, Slc7a1, Dpysl3, Mogs, Nsl1, Phf15, Casp8ap2, Ppat, LOC100045677/// Mcm3, Tagln, Mbnl3, Slc38a4, Rfc1, Fam111a, Dctpp1, Rfc5, Spg20, Cnn2, Piga, Htatsf1, Foxd1, Fkbp14, 2900062L11Rik, Ctnnal1, Flnc, Aurkb, B230120H23Rik, Fosl2/// LOC634417, Spin4, D2Ertd750e, Gins2, Usp1, Siglecg, Nanp, Figf/// LOC100047108, Mrpl15, Nup43, Arhgef10, 2610002D18Rik, Rassf5, Nudt1, Haus8, Recql4, Trp63, LOC100048050/// Lphn2, Rras2, Ugcg, Ttk, Masp1, Magi3, LOC100046855, Diap3, Wnt4, Ddah1, 1110034A24Rik, Cdc6, Ergic1, 2810417H13Rik, Pole2, Pml, 5730471H19Rik, Suv39h1, Fancb, Dhfr, Sub1, Krt7, D10627, Styk1, Car5b, Aen, Eme1, Cdc7, Gm10774/// Polr2l/// Usp50, Oat, Gemin5, Gemin6, Oxr1, Ston1, Aim1, Pole, Rpa1, Tyms/// Tyms-ps, Mcm4, Fbxo5, Hdac7, Tada2a, 4930579G24Rik, Prim2, Pold1, Smc4, Dnajc3, BC055324, Zyx, Wnk1, Siva1, Ccna2, Nasp, Ercc6l, Wisp2, Trpc2, Pcdh19, Adss, Idh1, Cdca7l, D10Jhu81e, Orc6l, Ehd4, Ckap2, Hnrnpr, Lpl, Cxcl12, Hirip3, Pdia6, Itgb5, Brca1, 6530418L21Rik, Cpeb3, Kdelc2, Spc25, 2410017P07Rik, Rpa2, Mlf1, Chtf18, Mybl2, Ccne1, Prps2, R3hcc1, Tbx18, Tcof1, Sephs2, Ipmk, Col7a1, Tnrc18, 4632434I11Rik, Rrm1, Incenp, Lims1, Slc25a30, Pdxp, Sdc2, Slbp, Tmem97, Anp32a, Lrig3, Nfkbiz, Steap2, Cenpk, Dctd, Rad54b, Tipin, Dus4l, Fam164a
24 hour	N	Gstm2, Blvrb, Alg11, Zfyve26, A930001N09Rik, Hscb, Ier2, Adam23, Sgk1, Snapc3, BC094435, Rev3l, Cish, Rnf24, Hs1bp3, Cdh11, Ifitm1, Vgll4, Zfp703, Itpkb, Wls, Sord, Btg2, Dab2, Zfp677, Phip, Serpinb8, Arfgap3/// LOC100045474, Gpsm1, Naip6, Apool, Pf4, Ccpg1, Ddah2, Mt1, Fos, Runx2, Zfhx3, Brwd3, Arhgap31, Atxn1, Pcmtd2, Zfp760, Cacnb3, Atp7a, Pcdhb16, Zfp260, Phxr4, Sash1, Tanc2, Dnm3os, Pld3, 2610005L07Rik, Agrn, Fam53b, Arsa, Bmp2k, Capn5, Creb3l2, Syne1, Tpp1, Btg1, Calcoco1, Mll2, Tspan12, Per3, Phc3, Amdhd2, Dzip1, Zfand1, 5730446D14Rik, Arhgap21, Rcn3, Mmp9, Tob1, Vat1, Tcf12, Rmnd5a, Nktr, AW112010, Fpgt, Nisch, Casp6, 4932438A13Rik, A430081F14Rik, Kdm5a, Ndrg4, Serpinb1a, Lgr4, Ralgds, D13Ertd787e, Agpat4, A530054K11Rik, 4933407H18Rik, 9230108I15Rik, Gm608, Phf1, Scara3, Zfp36l1, Kidins220, Scai, 2810454L23Rik, Vamp7, D0H4S114, Mbtd1, Gas7, Sh3pxd2a, Ankrd12, Pcdhb17, Spon2, Ccng2, Ech1, P2rx4, Ctso, Mxra8, Gabarap, Ttyh3, Atf7ip, Rhoq, AI131651, Myo6, 4632427E13Rik, Afap1, Zfp647, Erbb2ip, Jup, Stard9, Armcx1, Serpinb9, Bbs2, Ptprv, Grina, Sh3pxd2b, Serhl, Abhd4, Dock7, Slc29a3, 2510017J16Rik, Aph1b, Igf2, Dnajb4, Irgq, Zfp36, Cmtm3/// LOC100046883, Tmem140, Sesn3, Gm16515, Wbp2, Hmox1, Glt8d1, Hba-a1/// Hba-a2, Setd7, Dcaf12, Taok1, Prnp, Ctsd, Pisd-ps1/// Pisd-ps3, Hist1h1c, Mapk1ip1, Adck2, Ncrna00081, Nlgn2, Serinc5, Sec61a1, Ypel3, Ncoa2, LOC100047324/// Sesn1, Lrpap1, Aff3, Impact, Nudt7, Fnip1, Fam63b, Cyp39a1, Rbm39, Acadm, Luc7l2, Igbp1, Plin3, Nagk, Crbn, 5730601F06Rik, Abhd14b, Pdgfrb, Cpeb2, Ulk1, Zfhx4, S100a13, 1110038D17Rik, Spa17, Lnpep, 4833427G06Rik, Rftn2, Zfp826, Wbscr27, Plac8, Zbtb20, Rnf122, Tcf7l1, Ssbp2, Rhou, Nbeal1, Pltp, Lnx1, Wdr19, Clip3, Adam23/// LOC100046035, Plin2, A930013F10Rik, Selm, Tef, Ube2b, Dbp, Nicn1, Laptm4b, Synj1, Gstm5, Mll3, Pten, Tmem14a, Atp11c, Flcn, Nxf3, Nfat5, D030029J20Rik, Rasa4, Pnrc2, Arid4a, Zfp229, Adh1, 5330406M23Rik, Tns1, Lamp2, D6Wsu116e, AU015680, Hexb, Notch2, Inpp5e, Fam176b, Akap13, Ankrd44, Ctns, Avl9, Pik3r3, Gaa, Dexi, 2210419D22Rik/// Lrp6, 9030624J02Rik, Atp6v0d1, Sec16a, Abca1, LOC100047093/// Ube2h, Zfyve1, Kifap3, Thra, A730054J21Rik, Egr2, 2010315B03Rik, C430003N24Rik, Pcdhb20, H3f3b, Zfp52, Fmnl2, Slc17a5, Zfp187, Fmo2, Tcfap2a, AI426330, Alox5ap, Zkscan1, Gnpda1, Vkorc1, Purg, Slc22a5, Baz2b, Foxn3, Golgb1, Vps13b, N4bp2, Phf21a, Akap9, 4933421E11Rik, Il18, Csprs/// Gm7582, Pdgfra, Sqrdl, Gm2397, Pex1, Inpp5k, Dlg1/// LOC100047603, Armcx3, Ddx60, Sik1, Slc25a36, Itfg3, Marcksl1, Hbp1, Pcmtd1, Adhfe1, Mospd1, Acaa2, Tnrc6b, Dusp5, Ttc30b, Tmem37, Gbp8, Lrsam1, Gm7609, 4933407C03Rik, Col6a3, Hexa, LOC75771, Fbxl20, Foxo3, Pold4, Uqcrh, Itm2c, Galnt4, Hsd17b11, 4921509J17Rik, Pnrc1, Pgpep1, Itga2b, Trak1, 1810020D17Rik, Tctex1d2, Grn, Helz, Limd1, Wbp1, Capn6, Mgst1, Htra3, Gata2, Ptgfr, Bcl2l11, Mll5, Gm10786, Wdr6, Lpin1, Kdm5b, Dcxr, B230312A22Rik, Trim7, Crip1, Stat5a, Kcnq1ot1, Fbxo32, Bbs1, 1810031K17Rik
8 day	O	Wnk4, E130110O22Rik, Tom1l2, 6720401G13Rik, Kbtbd7, Rnf112, 2810433D01Rik, D16Ertd472e, Fam40b, Dirc2, Ppp1r2, Hist2h2be, Neb, Smox, Rbbp6, Mfsd8, LOC100046963/// Pgm5, Letm2, 4933439C10Rik, Scpep1, Rsph10b2, Kera, 2310039F13Rik, Lgals4, 3110005L24Rik, Mustn1, Tfpi, Plekha4, Dpt, Abhd3, Mertk, A730091E23Rik, Ptgir, Gas2, Sorcs2, Vegfa, Dcun1d5, Papss2, Enox1, Cyhr1, 5930403N24Rik, Nckap5, Chst15, Sgcb, Rgs5, Lrp4, Cyp1a1, Tinagl1, Hist2h2bb, Fam73a, Mrgprf, Epyc, Col5a3, Ndn, Hlx, Phkg1, Tmem191c, Aplp2, Cacnb4, Rapgef3, Kcnt2, AI464131, Itga1, 2310035P21Rik, Syn2, 9430020K01Rik, Pla1a, Abi3bp, Npas3, B430203I24Rik, Etv5, 2900092E17Rik/// Prrt2, Tspan2, U46068, Acaa1b, Ecm2, Ccl6, Tnks2, Ogt, Pcx, 2210012G02Rik, Ifi44, Arhgap6, Lepre1, C8g, Tmem44, Zscan18, Jmy, 8430427H17Rik, Gm12824, Prkx, Smarca2, Rab6b, Tmem2, Ccdc62, 2810039B14Rik, Ppp4r1l, 4921517L17Rik, Cdkn1b, Dnaic2, Etv4, Clec3b, Cplx2, Gfra2, P2rx1, Fam115a, 6330439K17Rik, Ctbs, LOC100044395/// Rbpms, Slc22a2, Ttll3, Jakmip3, Gnas, 9030619P08Rik, 4930539J05Rik, H2-DMa, Sema3d, AA408456, Plin4, LOC100047682/// Phkg1, Gpr133, Aldh3a2, 4831440E17Rik, Chst2, Fam149a, Gsta2, Jarid2, Lama4, Amy1, Rrm2b, Colec11, Ddit3, 1500015O10Rik, Tiam2, Hcfc1r1, Zfp420, Dcun1d4, Ppnr, C030030A07Rik, Srpx2, Sept6, Cml3, 2210409E12Rik, 3110007F17Rik, Tmem169, Eprs, Acsl6, Erp44, Fam161a, Fhod3, Smo, 2310014F06Rik, Car9, 9430047G12Rik, Cd59a, Rbpms, 4930528F23Rik, Fam46c, Pnpla6, Adh7, Slc12a9, Arl4a, Gm11602, Hmgcs2, Trib3, Svil, 4930579G22Rik, Tulp2, Hint3, Flt3l, Etv1, Rassf2, Twist2, Tmem206, P2rx3, Hdac9, Orm3, Ophn1, Plekha6, Col4a2, Add3, Ccnd2, Eepd1, 6030451C04Rik, 5730414N17Rik, Fus, Fam82a1, Obsl1, Zfp2, Kctd1, Tnnt1, Rbm5, Lhfpl2, Plcb1, Fgfrl1/// LOC100046239, Mysm1, Calcrl, Limch1, Gm11696, 4932415G12Rik, Spata9, Slco2b1, Gng8, 5730407I07Rik, Aldh3a1, F13a1, 2010011I20Rik, Smg6, Pappa2, Pparg, Hist2h2aa1/// Hist2h2aa2/// Hist2h2ac/// Hist2h3c1, Rarres2, Masp2, Tspan9, Lrp5, Slc9a9, 3110057O12Rik, Fam20a, Gpr155, Ccni, Lmcd1, Adamts6, L1cam, Mitf, Cxcl15, Hist2h3c1, Cyp2j6, AI480653/// LOC100047112, Clcn2, Fam71e1, Ddrgk1, Serinc4, Lamb2, 9430091E24Rik, Sema3b, Adamts2, Adamtsl4, Gpr135, Inca1, Eny2, 9630023C09Rik, Spry4, Aox1, Tmcc3, AW011738, Klhl10, Sparc, Gab3, Tmem8b, Pter, 1700019G17Rik, Cdh26, Kifc2, Ddo, 3110052M02Rik, 4933411B09Rik, Lrrn4, Trim34, Zfp455, 2900054C01Rik, Srp54a/// Srp54b, Kazald1, Zfp68, Dmp1, Pcdhb12, Mmp3, BC029214, Gls, A930035D04Rik, A330076H08Rik, Terf2ip, 2010007H06Rik, Fzd4, Hspa4l, 4933431E20Rik, Slc1a5, Ypel4, Aplf, Anxa11/// Gm2260/// Gm2274, Trpm5, Tet1, Susd2, Vav3, 1700029J07Rik, Dock4, Slc43a2, Pilra, S100a8, Arhgap26, Nudt12, 5830411K21Rik, Col20a1, Mamstr, Pcdhb15, 4930578N16Rik, Mical1, Gm15698, Dmd, Nudt4, Gm2521/// Sema3a, Rfesd, LOC632073/// U46068, Fuca1, Mmrn2, Tmem57, Nudt21, Rgs8, Oas1c, Prickle1, Uap1l1, 5730410E15Rik, Chac1, E030016H06Rik, Fxyd1, Ccdc28a/// LOC100045472, Inmt, Zbtb4, Tnfrsf21, Tnnc2, 2210008F06Rik, Pipox, Tm6sf1, Shprh, Gnao1, Cyp4f14, Pappa, Hist2h3c2, Fxyd5, Efnb1, Phf20l1, 1810010H24Rik, Olfml1, Abca9, Pcdhb11, Erc2, Cldn9, Myh11, Lman1l, Wwox, Dnajb9, Lpcat2, Lrrk2, Ptpn3, 2810408A11Rik, D9Ertd720e, Cetn4, Gm6556, Gm14005

C

Gene Ontlogy term	FDR-corrected p-value			
	1 h	6 h	24 h	8 days
multicellular organismal process	3.52×10^{-3}		1.79×10^{-3}	1.16×10^{-3}
multicellular organismal development	1.18×10^{-5}	3.07×10^{-3}	5.49×10^{-5}	1.82×10^{-5}
nervous system development			9.96×10^{-3}	2.44×10^{-3}
system development	4.37×10^{-6}	2.15×10^{-3}	9.15×10^{-5}	1.99×10^{-5}
skeletal system development	7.20×10^{-4}		1.13×10^{-3}	1.26×10^{-3}
skeltal system morphogenesis			3.15×10^{-3}	
urogenital system development	1.89×10^{-3}			
kidney development	4.01×10^{-3}			
heart development	1.33×10^{-3}			
organ development	4.47×10^{-7}	1.59×10^{-3}		
organ morphogenesis	6.07×10^{-4}	1.21×10^{-4}		
muscle organ development	2.18×10^{-4}			
skeletal muscle organ development	9.11×10^{-4}			
skeletal muscle tissue development	7.83×10^{-4}			
striated muscle tissue development	5.37×10^{-5}			
muscle tissue development	3.99×10^{-5}			
tissue development	1.96×10^{-7}	8.13×10^{-4}		
tissue morphogenesis	4.41×10^{-3}	2.94×10^{-4}		
epithelium development		2.29×10^{-3}		
morphogenesis of an epithelium				
epithelial tube morphogenesis		1.49×10^{-3}		
mammary gland duct morphogenesis		2.87×10^{-4}		
mammary gland morphogenesis		6.17×10^{-3}		
gland morphogenesis		6.90×10^{-4}		
tube development	6.25×10^{-4}	4.06×10^{-4}		
tube morphogenesis		7.95×10^{-5}		
branching morphogenesis of a tube		7.89×10^{-4}		
morphogenesis of a branching structure	6.20×10^{-4}	8.09×10^{-5}		
anatomical structure morphogenesis	1.25×10^{-5}	2.37×10^{-4}	1.51×10^{-3}	1.80×10^{-3}
organ morphogenesis			2.95×10^{-3}	
organ development			5.16×10^{-3}	2.89×10^{-3}
vasculature development	4.16×10^{-5}	5.89×10^{-3}	1.23×10^{-3}	
blood vessel development	8.54×10^{-5}	3.97×10^{-3}	1.95×10^{-3}	
blood vessel morphogenesis	1.05×10^{-4}			
angiogenesis	2.98×10^{-3}			
anatomical structure formation involved in morphogenesis	1.31×10^{-3}			
anatomical structure development	4.27×10^{-5}	1.33×10^{-3}	7.80×10^{-5}	7.67×10^{-6}
developmental process	9.70×10^{-5}	2.73×10^{-3}	6.58×10^{-5}	5.90×10^{-6}
regulation of developmental process	4.49×10^{-6}		3.71×10^{-3}	8.19×10^{-3}
regulation of anatomical structure morphogenesis	4.79×10^{-4}	5.98×10^{-3}		
regulation of cell shape	7.20×10^{-4}			
regulation of cellular component organization	6.64×10^{-3}			
positive regulation of biological process			1.31×10^{-3}	3.06×10^{-3}
positive regulation of cellular process			3.29×10^{-4}	2.96×10^{-3}
biological regulation	6.67×10^{-7}		2.11×10^{-2}	
regulation of biological process	9.01×10^{-3}		1.54×10^{-2}	
negative regulation of biological process	3.18×10^{-3}			
negative regulation of cellular process	3.07×10^{-3}			
regulation of cell-substrate adhesion	7.59×10^{-3}			
regulation of cell adhesion	5.98×10^{-3}			
regulation of cellular process	5.88×10^{-7}			
enzyme linked receptor protein signaling pathway	4.06×10^{-5}			
transmembrane receptor protein tyrosine kinase signaling pathway	8.93×10^{-3}			
regulation of cellular component movement				
regulation of cell migration	2.01×10^{-5}			
positive regulation of cellular comporment				
positive regulation of cellular process	6.67×10^{-3}	2.29×10^{-3}		
positive regulation of cell migration	7.81×10^{-4}			
cell motility	8.51×10^{-3}			
cell migration	2.15×10^{-3}			
positive regulation of locomotion	2.25×10^{-3}			
regulation of locomotion	1.19×10^{-4}			
regulation of multicellular organismal process	9.04×10^{-3}		3.52×10^{-3}	1.75×10^{-3}
regulation of metabolic process	6.71×10^{-4}			
regulation of nitrogen compound metabolic process	8.07×10^{-3}			
regulation of cellular metabolic process	6.35×10^{-4}			
regulation of nucleobase, nucleoside, nucleotide and nucleic acid metabolic process	6.63×10^{-3}			
regulation of primary metabolic process	2.79×10^{-3}			
regulation of cellular biosynthetic process	8.56×10^{-3}			
regulation of biosynthetic process	9.01×10^{-3}			
regulation of macromolecule biosynthetic process	8.78×10^{-3}			
regulation of transcription DNA dependent	8.47×10^{-3}			
regulation of transcription from RNA polymerase II promoter	1.58×10^{-3}			
positive regulation of transcription from RNA polymerase II promoter	9.83×10^{-3}			
regulation of gene expression	5.89×10^{-3}			
regulation of macromolecule metabolic process	7.33×10^{-3}			

Figure S4. Gene Ontology terms for selected genes

GO terms down-regulated due to TPA treatment for 1 h, 6 h, 24 h, and 8 days (C).

C (continued)

Gene Ontlogy term	FDR-corrected p-value 1 h	6 h	24 h	8 days
nitrogen compound metabolic process		8.99×10^{-10}		
cellular nitrogen compound metabolic process		6.67×10^{-10}		
nucleobase-containing compaund metabolic process		9.74×10^{-11}		
DNA metabolic process		1.22×10^{-34}		
regulation of DNA metabolic process		3.59×10^{-3}		
negative regulation of DNA metabolic process		1.53×10^{-4}		
DNA recombination		9.45×10^{-9}		
DNA replication initiation		8.52×10^{-6}		
DNA-dependent DNA replication		2.66×10^{-9}		
DNA replication		1.97×10^{-29}		
DNA repair		5.73×10^{-14}		
cellular macromolecule metabolic process		3.82×10^{-4}		
cellular metabolic process		2.45×10^{-3}		
cell division		1.07×10^{-10}		
cellular process		9.14×10^{-5}		
response to stress		6.48×10^{-8}		
cellular response to stress		5.73×10^{-13}		
cellular response to stimulus		2.62×10^{-10}		
response to ionizing radiation		3.56×10^{-3}		
response to stimulus		1.81×10^{-3}		
cell cycle		1.68×10^{-22}		
regulation of cell cycle		4.10×10^{-4}		
mitotic cell cycle		6.31×10^{-10}		
M phase of mitotic cell cycle		6.69×10^{-10}		
M phase		3.77×10^{-11}		
cell cycle phase		1.51×10^{-11}		
cell cycle process		3.68×10^{-12}		
mitosis		3.75×10^{-10}		
nuclear division		3.75×10^{-10}		
organelle fission		8.89×10^{-10}		
cytoskeleton organization		6.77×10^{-3}		
organelle organization		8.37×10^{-8}		
cellular component organization		2.13×10^{-8}		
biological adhesion				1.72×10^{-3}
cell adhesion				1.81×10^{-3}
regulation of cell-substrate adhesion				9.09×10^{-3}
extracellular structure organization				4.99×10^{-4}
extracellular matrix organization				3.12×10^{-5}

Figure S4. Gene Ontology terms for selected genes

GO terms down-regulated due to TPA treatment for 1 h, 6 h, 24 h, and 8 days (C, continued).

Pathway Analysis Using IPA Software; canonical pathway

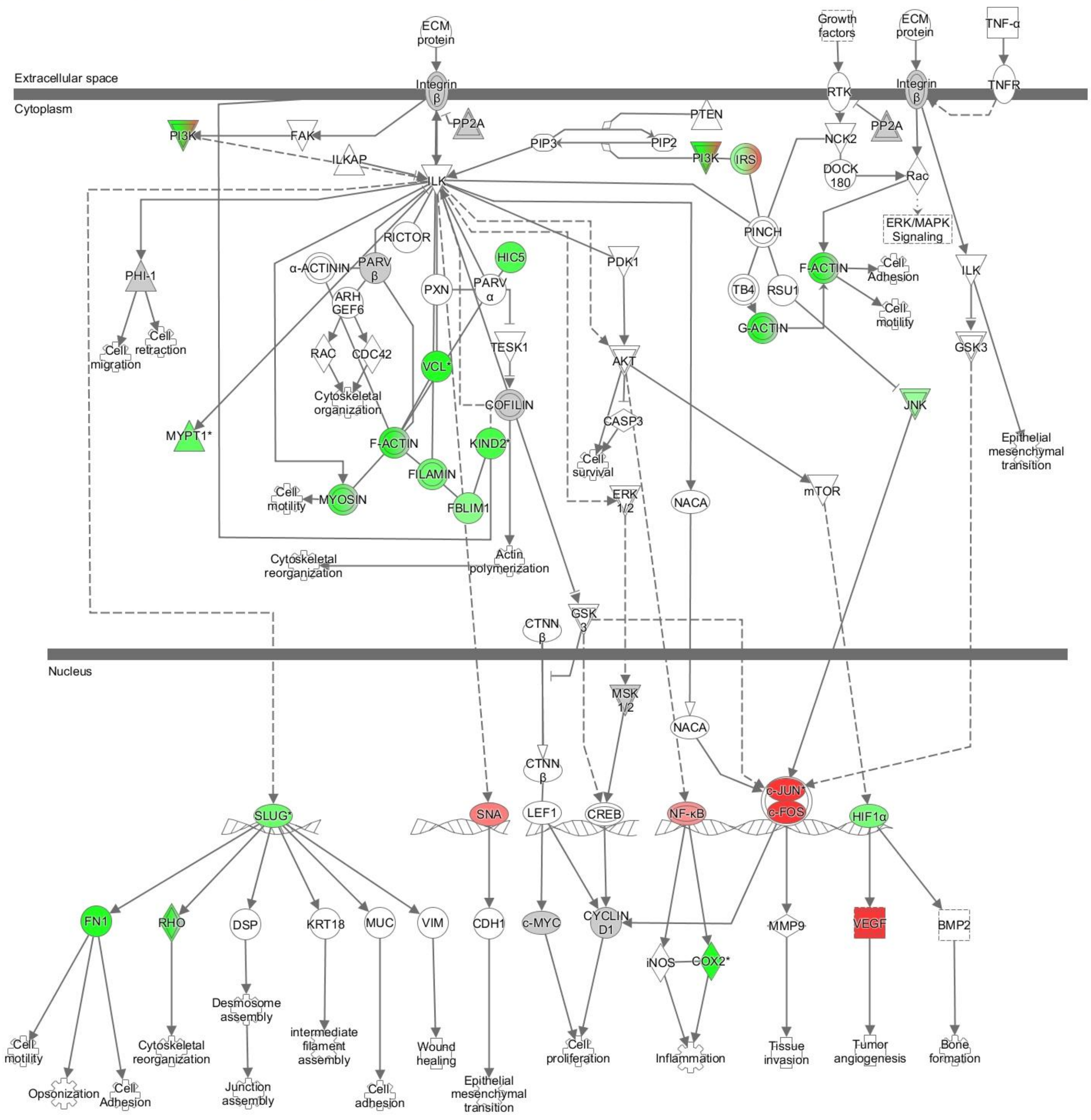

Figure S5. ILK Signaling at 1 h

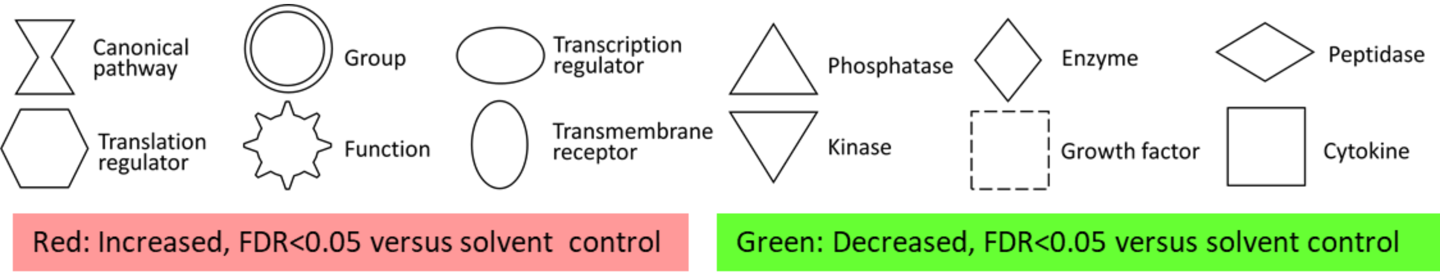

Symbol	Synonym(s)
AKT	AKT1/2/3, B/Akt, PKB, RAC-PK
Alphaactinin	ACTININ, Actinin alpha, Actinin α, ACTN, α-Actinin, α Actinin human
Ap1	activator protein-1, c-Jun
ARHGEF6	1600028C08Rik, 1700038J06Rik, 4930592P22RIK, alp, alpha-PIX, COOL2, MRX46, PIXA, Pix-alpha, Pix-α, Rac/Cdc42 guanine nucleotide exchange factor 6, Rac/Cdc42 guanine nucleotide exchange factor (GEF) 6, α-PIX
Betaintegrin	beta-Integrin, Integrin beta, β- Integrin
BMP2	AI467020, BDA2, Bmp, BMP2A, bone morphogenetic protein 2, BONE MORPHOGENIC protein 2, SSFSC1, SSFSC
CASP3	A830040C14Rik, AC-, AC-3, Casp, Caspase-3, CASPASE-3 p20, CC3, CPP, CPP-32, CPP32B, CPP32-beta, CPP32-β, Ice-like cysteine protease, Lice, mld, mldy, SCA-1, Ya, YAMA
CCND1	AI327039, B-CELL CLL/LYMPHOMA 1, bcl-, BCL1, cD1, CycD1, CYCLIN D1, Cyl-, Cyl-1, D11S287E, G1/S-Specific Cyclin D1, PR, PRAD1, U21B31
CDC42	AI747189, AU018915, CDC42Hs, cell division cycle 42, CELLULAR GROWTH REGULATING, G25K, TKS
CDH1	AA960649, ARC-1, BCDS1, cadherin 1, Cadherin E, CD324, CDHE, CSEIL, E-ca, ECAD, E-cadh, E-cadherin, L-C, L-CAM, Um, UVO, uvomorulin
COFILIN	CFL
CREB	Cbp, Cyclic AMP response element binding
CTNNB1	armadillo, Beta-cat, beta CATENIN, Bfc, Cat, CATENIN beta, catenin beta 1, catenin (cadherin associated protein), beta 1, catenin (cadherin associated protein), β 1, CATENIN β, catenin β 1, CATNB, CTNB1, CTNNB, CTNN beta, CTNN β, EVR7, Mesc, MRD19, NEDSDV, β-cat, β-catenin
DOCK1	9130006G06Rik, AI854900, b2b3190C, b2b3190Clo, ced5, D630004B07Rik, dedicator of cyto-kinesis 1, Dock18, DOCK180, LOC679295, RGD1566072
DSP	2300002E22Rik, 5730453H04RIK, AA407887, AA407888, AW109828, D, DCWHKTA, desmoplakin, DP, DPII, r, rul
ERK1/2	MAPK p44/42, MAPK p44/p42, p42/44 mapk, P42/p44 erk, P42/p44 mapk, p42/p44 MAP KINASE
F Actin	Filamentous Actin
FBLIM1	2410043F08Rik, C, CAL, DKFZp434G171, Fbl, FBLP-1, filamin binding LIM protein 1, Gt10, hnRNP L, mig, migfi, MIGFILIN, migfilin(s)
FERMT2	AA960555, FERM domain containing kindlin 2, fermitin family member 2, Kind, KIND2, KINDLIN 2, Mig, MIG2, Plekh, PLEKHC1, UNC112, UNC112B
FILAMIN	Abp, ABP280/FH1
FN1	cFn, CIG, E330027I09, ED-B, F, FIBNEC, Fibronectin, FIBRONECTIN 1, Fibronectin3 M1, Fibronectin i, FINC, FN, FN1 isoform 1, FNZ, GFND, GFND2, LETS, MSF, SMDCF
FOS	AP-1, c-f, C-FOS, D12Rfj, D12Rfj1, FBJ osteosarcoma oncogene, Fos proto-oncogene, AP-1 transcription factor subunit, p55
Gsk3	Glycogen synthase kinase, Gsk, GSK3 alpha/beta, GSK3 α/β
HIF1A	AA959795, bHLHe7, bHLHe78, HIF-1, HIF1-ALPHA, HIF-1alpha (hydroxylated), HIF-1-α, HIF-1α (hydroxylated), Hypoxia inducible factor 1 alpha subunit, hypoxia inducible factor 1, alpha subunit, hypoxia inducible factor 1 subunit alpha, hypoxia inducible factor 1 subunit α, Hypoxia inducible factor 1 α subunit, hypoxia inducible factor 1, α subunit, MO, MOP1, PASD8
ILK	AA511515, ESTM2, ESTM24, HEL-S-28, ILK-1, ILK-2, integrin-linked kinase, P59, p59ILK
ILKAP	0710007A14RIK, 1600009O09Rik, AF095927, AK055417, ILKAP2, ILKAP3, ILK associated serine/threonine phosphatase, integrin-linked kinase-associated serine/threonine phosphatase 2C, PP2C-D, PP2C-DELTA, PP2C-δ, PPM1O
IRS	INSULIN receptor SUBSTRATE
JNK	JNK 54/46, Jnk p56, JNK/SAPK, JUN KINASE, p40, p47, Sapk/Jnk
JUN	Activator protein 1, AP-1, API-1, c-ju, cJUN, Junc, jun proto-oncogene, Jun proto-oncogene, AP-1 transcription factor subunit, LOC100288387, LOC100291417, LOC100293034, p39, v-Jun, V-jun Avian Sarcoma Virus 17 Oncogene Homolog, V-jun Sarcoma Virus 17 Oncogene Homolog
KRT18	48-kD Keratin, CK-18, Ck D, CYK18, K18, K1cr, keratin 18, Keratin complex 1 acidic gene 18, Krt1-1, KRT1-18
LEF1	3000002B05, AI451430, Lef-, lymphoid enhancer binding factor 1, TCF10, TCF1ALPHA, TCF7L3, TCF/LEF
LIMS	PINCH
MAP2K6	MAPKK6, Mapk kinase 6, MEK6, mitogen-activated protein kinase kinase 6, MKK6, MKK6BE, Prkm, PRKMK6, Rac, SAP, SAPKK-3
MMP9	AW743869, B/MMP, B/MMP9, Clg4, CLG4B, COLLAGENASE type IV, Gelatinase B, GELB, GI 92-kda, MANDP2, matrix metallopeptidase 9, METALLOPROTEINASE 9, MMP-, pro-MMP-9
MTOR	2610315D21Rik, AI327068, fl, Flat, Fr, FRAP, FRAP1, FRAP2, FRB, mechanistic target of rapamycin kinase, RA, RAF, RAFT1, RAPT1, RRAFT1, SKS
MUC1	ADMCKD1, ADMCKD, ADTKD2, CA 15-3, CD227, EM, EMA, Episialin, H23AG, KL-6, MAM6, MCD, MCKD1, MCKD, MUC1-CT, Mucin1, Mucin, mucin 1, cell surface associated, Mucin-1 subunit beta, Mucin-1 subunit β, PANCREATIC MUCIN, PEM, PEMT, PUM
MYC	AU016757, bHLHe3, bHLHe39, CMYC, C-MYC-P64, mMyc, MRTL, Myc2, MYCC, MYC proto-oncogene, bHLH transcription factor, myelocytomatosis oncogene, N, Niard, Nird, RNCMYC
NACA	AL022831, AL024382, alpha-NAC, D12Mgi4, Gm1878, HSD48, mKIAA0363, NACA1, NAC-alpha, NAC-α, Nascent polypeptide associated complex alpha chain, Nascent polypeptide-associated complex alpha polypeptide, nascent polypeptide associated complex subunit alpha, nascent polypeptide associated complex subunit α, Nascent polypeptide associated complex α chain, Nascent polypeptide-associated complex α polypeptide, sk, skNAC, α-NAC
NCK2	4833426I10RIK, GRB4, Grb, LOC100503894, NCK adaptor protein 2, Nck β, NCKbe, NCKbeta, non-catalytic region of tyrosine kinase adaptor protein 2
NFkB	NF-KAPPA B, NF-κ B, nuclear factor-κ b, transcription factor nuclear factor κ b
NOS2	CALCIUM-INDEPENDENT NOS, Hepatocyte NOS, HEP-NOS, Inducible NOS, INOS, LOC497963, MAC-NOS, N, nitric oxide synthase 2, nitric oxide synthase 2, inducible, No, NOS, NOS2A, NOS-II, similar to Nitric oxide synthase, inducible (NOS type II) (Inducible NO synthase) (Inducible NOS) (iNOS)
PARVA	2010012A22Rik, 5430400F08Rik, act, Actopaxin, Actp, AI225929, alpha PARVIN, AU042898, CH-IL, CH-ILKBP, MXRA2, Parvin, Parvin-alpha, parvin, alpha, Parvin-α, parvin, α, α PARVIN
PARVB	aff, affixin, AI595373, AW742462, CGI-56, D15Gsk, D15Gsk1, Parvin-beta, parvin, beta, Parvin-β, parvin, β
PDPK1	3'-PDK, 3-phosphoinositide dependent protein kinase-1, Pdk, PDK1, PDPK2, PDPK2P, PRO0461
PI3K	1-phosphatidylinositol 3-kinase, 2.7.1.137, ATP:1-phosphatidyl-1D-myo-inositol 3-phosphotransferase, Phosphatidylinositol 3 kinase, phosphatidylinositol 3'-kinase, PI3-kinase, PtdIns 3 Kinase, type I phosphatidylinositol kinase, type III phosphoinositide 3-kinase, Vps34p
PIP2	1,2-diacyl-sn-glycero-3-phospho-(1'-myo-inositol-4',5'-bisphosphate), 1-O-(3-sn-phosphatidyl)-1D-myo-inositol 4,5-bis(dihydrogen phosphate), 1-phosphatidyl-1D-myo-inositol 4,5-bisphosphate, C11H19O19P3R2
PIP3	1-phosphatidyl-1D-myo-inositol 3,4,5-trisphosphate, phosphatidylinositol-3,4,5-trisphosphate, phosphoinositide (3,4,5) P3, PI(3,4,5)P3, PIns(3,4,5)P3, PIP3, PtdIns(3,4,5)P3
PP2A	protein PHOSPHATASE 2A, Protein Phosphatase Type2a
PPP1R12A	1200015F06Rik, 5730577I22Rik, AA792106, AV099298, D10Ertd625, D10Ertd625e, GUBS, M110, M130, MBS, MBS130, MBSP, Mypt, MYPT1, protein phosphatase 1 regulatory subunit 12A, protein phosphatase 1, regulatory subunit 12A
PPP1R14B	AOM1, AOM172, FG-4095, P, PHI, PHI-1, Phospholipase c neighboring, PLC, PLCB3N, PNG, Protein phosphatase 1, protein phosphatase 1 regulatory inhibitor subunit 14B, protein phosphatase 1, regulatory (inhibitor) subunit 14B, protein phosphatase 1, regulatory inhibitor subunit 14B, SOM172
PTEN	10q23del, 2310035O07RIK, A130070J02Rik, AI463227, B430203M17RIK, BZS, CWS1, DEC, GLM2, MHAM, MMAC, MMAC1, MUTATED IN MULTIPLE ADVANCED CANCERS, mutated in multiple advanced cancers 1, phosphatase and tensin homolog, PTEN1, PTENbeta, TEP, TEP1
PTGS2	COX, COX-2, CYCLO-OXYGENASE 2, GRIPGHS, hCox-2, hnCOX-2, INDUCIBLE CYCLOOXYGENASE, PES-2, PGG/HS, Pgh, PGHS, PGHS-2, PGH synthase 2, Pgi2 synthase, Pgs2, Pgsi, PHS, PHS-2, PHS II, Prostaglandin endoperoxide synthase 2, Tis, TIS10
PTK2	FA, Fad, FADK, FADK 1, FAK, FAK1, FAK related non-kinase, FR, p125FAK, pp125FAK, PPP1R71, protein tyrosine kinase 2, PTK2 protein tyrosine kinase 2, TYROSINE KINASE 2
PXN	AW108311, AW123232, FLJ23042, P, PAX, PAXILLIN
RHO	GTPase Rho, Rho, Rho Family, RHO-GTPASE, Rho-like Gtpase
RICTOR	4921505C17Rik, 6030405M08Rik, AVO3, AW492497, D530039E11Rik, hAVO3, KIAA1999, Mtorc2, PIA, RPTOR independent companion of MTOR complex 2, RPTOR independent companion of MTOR, complex 2
RSU1	LOC684929, R, Ras suppressor protein 1, rs, RSP-1, RsuI
SNAI1	AI194338, dJ710H13.1, S, SLUGH2, SNA, Sna1, SNAH, SNAIL, SNAIL1, snail family transcriptional repressor 1, snail family zinc finger 1
SNAI2	S, SI, Slu, SLUG, SLUGH, SLUGH1, SNAIL2, snail family transcriptional repressor 2, snail family zinc finger 2, WS2D
TESK1	AI326901, p36-MBPK, testis associated actin remodelling kinase 1, testis specific protein kinase 1
TGFB1I1	AR, ARA55, hic-, HIC-5, TGF beta 1I1, TGF β 1I1, transforming growth factor beta 1 induced transcript 1, transforming growth factor β 1 induced transcript 1, TSC-, TSC-5
TMSB4	TB4, THYMOSIN B4, Thymosin beta4, Thymosin β 4, thymosin β(4)
TNF	AT-TNF, DI, DIF, RATTNF, TMTNF, Tn, TNF-a, TNF-alpha, Tnfs, Tnfsf1a, TNFSF2, TNF-α, TNLG1F, tumor necrosis factor, Tumor Necrosis Factor α, tumor necrosis factor, α, tumour necrosis factor, tumour Necrosis Factor Alpha, tumour necrosis factor, alpha, tumour Necrosis Factor α, tumour necrosis factor, α
TNFRSF1A	CD120, CD120a, FPF, p5, p55, p55-R, p55 TNF alpha receptor, p55 TNF α receptor, p60, TBP1, TN, TNF-, TNF-a, TNFaI, TNF-alphaR1, Tnf alpha receptor p60, TNFAR, TNF-R, TNF-R1, Tnfr-2, TNF-R55, TNFR60, TNF receptor 1, TNF receptor superfamily member 1A, TNF-R-I, TNFRp55, Tnf-α Receptor 2, Tnf α receptor p60, Tnf α Receptor Type1, Tnf-α receptor type I, tumor necrosis factor receptor superfamily, member 1a, tumour necrosis factor receptor 1, tumour necrosis factor receptor superfamily, member 1a
VCL	9430097D22, AA571387, AI462105, AW545629, CMD1W, CMH15, HEL114, MV, MVCL, Vcl predicted, Vinculin
VIM	PAL-E, Vimentin

Pathway Analysis Using IPA Software; canonical pathway

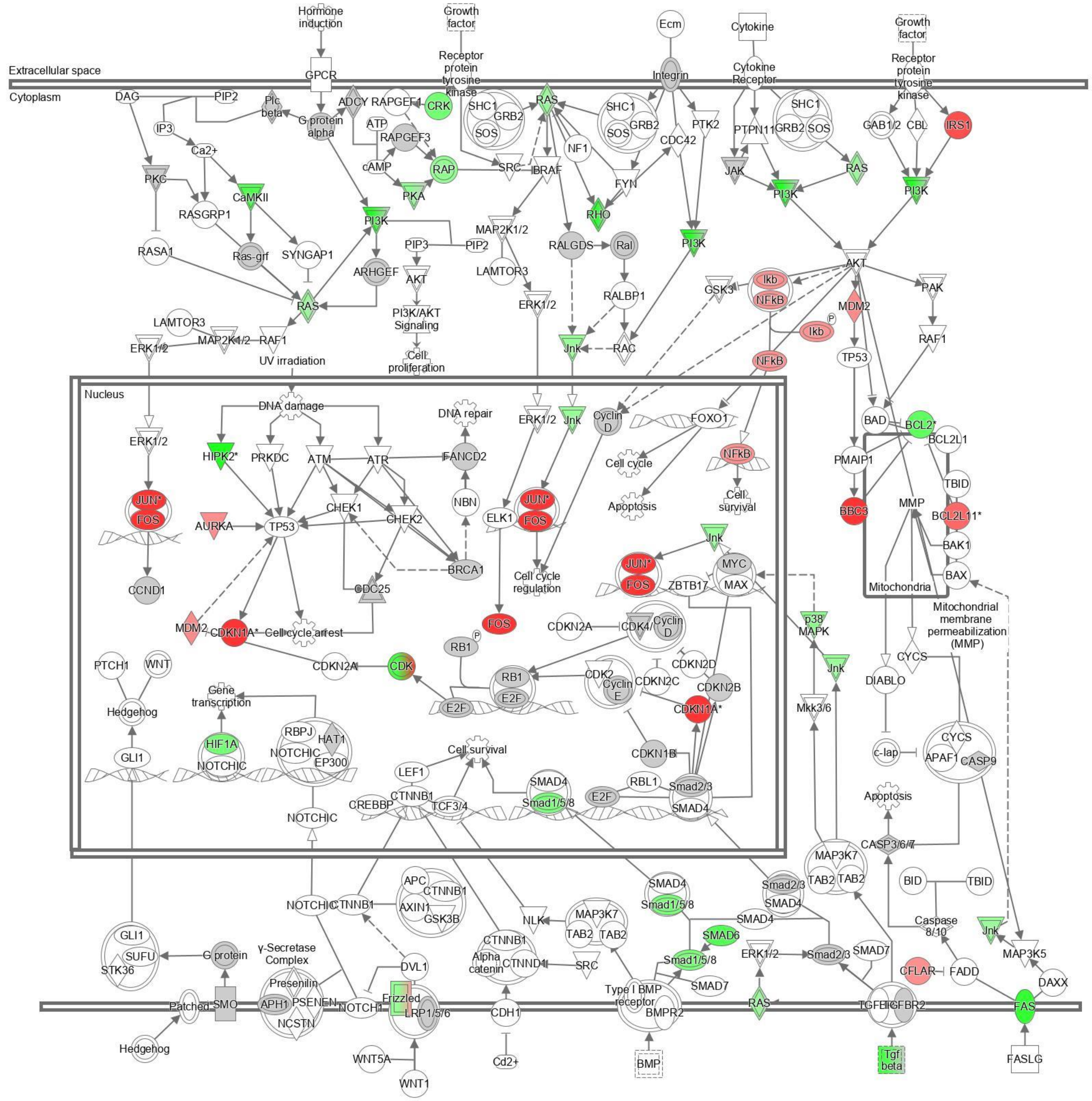

Figure S6. Molecular Mechanism of cancer at 1 h

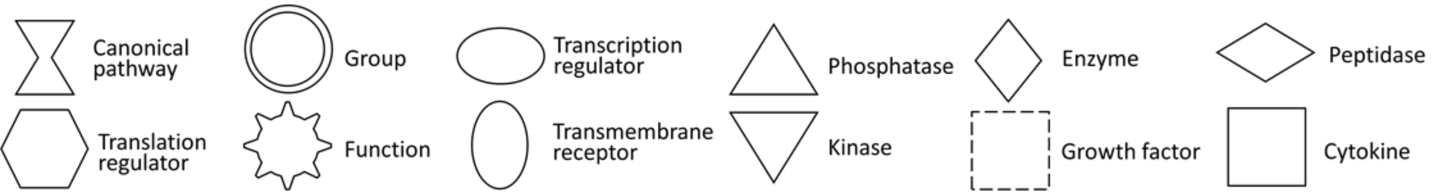

Red: Increased, FDR<0.05 versus solvent control

Green: Decreased, FDR<0.05 versus solvent control

Symbol	Synonym(s)
ADCY	3',5'-cyclic AMP synthetase, 4.6.1.1, AC, Adenylate Cyclase, Adenyl Cyclase, Adenylyl cyclase, ATP diphosphate-lyase (cyclizing), mAC, sAC
AKT	AKT1/2/3, B/Akt, PKB, RAC-PK
Alphacatenin	CTNN alpha, CTNN α, α catenin
Ap1	activator protein-1, c-Jun
APAF1	6230400I06RIK, Ap, Apaf1l, apoptotic peptidase activating factor 1, CED4, fog, mKIAA0413
APC	AI047805, APC1, Apc7, APC (PROC), APC regulator of WNT signaling pathway, APC, WNT signaling pathway regulator, AU020952, AW124434, BTPS2, CC1, DESMD, DP2, DP2.5, DP3, Familial adenomatous polyposis, GS, M, mAPC, Min, PPP1R46, RATAPC
Apoptosome	APAF1-Caspase 9-CytoC, apoptosis adaptor protein complex, Cytochrome C-APAF1-Caspase 9
ARHGEF	Ras GEF, RhOGEF
ATM	AI256621, AT1, ATA, ataxia telangiectasia mutated, ATC, ATD, ATDC, ATE, ATM serine/threonine kinase, C030026E19RIK, TEL1, TELO1
ATP	[[(2R,3S,4R,5R)-5-(6-aminopurin-9-yl)-3,4-dihydroxyoxolan-2-yl]methoxy-hydroxyphosphoryl] phosphono hydrogen phosphate, 56-65-5, 9-beta-D-arabinofuranosyladenine 5'-triphosphate, 9-β-D-arabinofuranosyladenine 5'-triphosphate, adenosine 5'-(tetrahydrogen triphosphate), adenosine 5'-triphosphate, ATP, ATP4-, C10H16N5O13P3
ATR	ataxia telangiectasia and Rad3 related, Ataxia-telangiectasia-like, ATR serine/threonine kinase, FCTCS, FRP1, LOC100365674, LOC367198, LOC684113, MEC1, SCKL, SCKL1
AURKA	AI, AIK, AIRK1, Ar, ARK-1, Au, AU019385, AURA, AURORA 2, AURORA A, AURORA KINASE, aurora kinase A, Aurora Related Kinase1, AW539821, Ayk, Ayk1, BTAK, I, IA, IAK, IAK1, PPP1R47, Stk, STK15, STK6, STK7
AXIN1	AI316800, AXIN, AXIN form I, Fu, fused, Kb, Ki, kinky, knobbly, PPP1R49
BAD	AI325008, Bad v1, Bad v2, BBC2, BCL2-associated agonist of cell death, BCL2L8
BAK1	Ba, BAK, BAK-LIKE, BCL2-antagonist/killer 1, BCL2L7, CDN1, N-B, N-BAK1
BAX	Bcl2-associated X, BCL2 associated X, apoptosis regulator, BCL2-associated X protein, BCL2L4
BBC3	BCL2 binding component 3, JFY-1, PU, PUMA, PUMA/JFY1
BCL2	AW986256, B cell leukaemia/lymphoma 2, B cell leukemia/lymphoma 2, Bcl-, Bcl2 alpha, BCL2 apoptosis regulator, BCL2, apoptosis regulator, Bcl2 α, C430015F12Rik, D630044D05RIK, D830018M01RIK, LOC100046608, ORF16, PPP1R50
BCL2L1	bBclxl, Bcl, BCL2L, BCL2-like 1, BCLX, Bcl-X beta, Bclx gamma, BCL-XL/S, Bcl-X β, Bclx γ, PPP1R52
BCL2L11	1500006F24RIK, BAM, BCL2 like 11, BCL2-like 11 (apoptosis facilitator), Bi, BIM, Bo, BOD, BODL, LOC150819
BMP	BMP3, BMP-3A, BONE MORPHOGENIC, Osteogenin
BMPR2	2610024H22RIK, AL117858, AW546137, BB189135, BM, BMP-, BMP-2, BMPR3, BMPR-II, BMR2, bone morphogenetic protein receptor type 2, bone morphogenetic protein receptor, type II (serine/threonine kinase), BRK-3, Gm20272, POVD1, PPH1, T-ALK, Type ii bmp receptor
BRAF	9930012E13RIK, AA120551, AA387315, AA473386, AI447469, Bra, B-RAF1, Braf-2, B-Raf proto-oncogene, serine/threonine kinase, Braf transforming gene, C230098H17, C87398, D6Ertd631, D6Ertd631e, NS7, RAFB, RAFB1
BRCA1	BRCA1 DNA repair associated, BRCA1, DNA repair associated, BRCAI, BRCC1, breast cancer 1, early onset, BROVCA1, FANCS, PNCA4, PPP1R53, PSCP, RNF53
c-Iap	IAP, NAIP
Ca2+	14127-61-8, Ca+2, calcium, calcium(2+), calcium cation, calcium citrate, calcium ion, calcium, ion (Ca2+), calcium ions, Citracal, tricalcium dicitrate
CaMKII	CA2+/CALMODULIN DEPENDENT KINASE II, Ca+/calmodulin-dependent protein kinase ii, calmodulin-dependent protein kinase 2, Calmodulin Kinase II, CAMK2, CaM Kinase II, Ccdpk ii
cAMP	11002-78-1, 33116-15-3, 3',5'-cyclic AMP, 3',5'-monophosphate, adenosine cyclic, 37839-81-9, (4aR,6R,7R,7aS)-6-(6-aminopurin-9-yl)-2-hydroxy-2-oxo-4a,6,7,7a-tetrahydro-4H-furo[3,2-d][1,3,2]dioxaphosphinin-7-ol, 54532-48-8, 55576-98-2, 60-92-4, 66067-13-8, 68407-13-6, adenosine 3',5'-phosphate, adenosine, cyclic 3',5'-(hydrogen phosphate), adenosine cyclic 3,5 monophosphate, adenosine cyclic 3',5'-monophosphate, adenosine cyclic monophosphate, C10H12N5O6P, cAMP, cyclic-3',5'-monophosphate, adenosine, cyclic adenosine monophosphate, cyclic adenylic acid, cyclic AMP, disodium salt, cyclic AMP, monoammonium salt, cyclic AMP, monopotassium salt, cyclic AMP, monosodium salt, cyclic AMP, sodium salt
CASP3/6/7	CASP3/6/7, Caspase 3, 6, 7, Caspase-3, -6, and -7
CASP9	AI115399, APAF-3, AW493809, Casp, Casp9 v1, Caspase-9, ICE-, ICE-LAP6, MCH6, PPP1R56
Caspase8/10	Casp8/10, Caspase 8,10
CBL	4732447J05Rik, Casitas B-lineage lymphoma, CBL2, CBLA, Cbl proto-oncogene, Cbl ubiquitin ligase, c-Cb, C-CBL, FRA11B, LOC283153, NSLL, p120 Cbl, RGD1561386, RNF55
CCND1	AI327039, B-CELL CLL/LYMPHOMA 1, bcl-, BCL1, cD1, CycD1, CYCLIN D1, Cyl-, Cyl-1, D11S287E, G1/S-Specific Cyclin D1, PR, PRAD1, U21B31
Cd2+	22537-48-0, cadmium(2+), cadmium acetate, cadmium cation, cadmium ion, cadmium, ion (Cd2+), Cd+2
CDC25	mRNA encoding Cdc25-like
CDC42	AI747189, AU018915, CDC42Hs, cell division cycle 42, CELLULAR GROWTH REGULATING, G25K, TKS
CDH1	AA960649, ARC-1, BCDS1, cadherin 1, Cadherin E, CD324, CDHE, CSEIL, E-ca, ECAD, E-cadh, E-cadherin, L-C, L-CAM, Um, UVO, uvomorulin
CDK	Cdks, cyclin-dependent kinase, Cyclin-Dependent Kinases, G1 CDK
CDK2	A630093N05Rik, CDC2-RELATED KINASE, CDKN2, Cyclin A associated kinase, cyclin-dependent kinase 2, CYCLIN E ASSOCIATED KINASE, p33(CDK2)
CDK2-CyclinE	Cyclin E-CDK2
CDKN1A	CAP, CAP20, CDK, CDKI, Cdkn, CDKN1, CDKNA1, CI, CIP1, cyclin-dependent kinase inhibitor 1A, cyclin-dependent kinase inhibitor 1A (P21), mda, MDA-6, P2, P21, p21C, p21Cip, p21CIP1, P21W, p21WAF, p21Waf1, PzI Cyclin-Dependent Kinase Inhibitor, SD, SDI1, UV96, Waf, WAF1
CDKN1B	AA408329, AI843786, Cdki1b, CDKN4, cyclin-dependent kinase inhibitor 1B, CYCLIN-DEPENDENT KINASE INHIBITOR P27, KIP1, MEN1B, MEN4, p2, p27, p27K, P27kip, P27KIP1, P28-ICK
CDKN2A	A, Arf, ARF-INK4a, CDK4I, CDKN2, CMM2, CYCLIN-DEPENDENT KINASE INHIBITOR 2A, INK4, INK4A, INK4a-ARF, Ink4a/Arf, MLM, MTS, MTS-1, p1, p14ARF/ p16INK4a, p16, p16/ARF, p16Cdkn2a, p16I, p16 INK4, p16/ INK4a, P19, p19ARF, Pct, PCTR1, TP16
CDKN2B	AV083695, CDK4I, cyclin-dependent kinase inhibitor 2B, INK4B, MTS, MTS2, p1, P15, p15IN, p15INK4, p15INK4b, p15(INK4b)
CDKN2C	C77269, CDKN6, cyclin-dependent kinase inhibitor 2C, INK, INK4C, p1, p18, p18IN, p18-INK4C, p18-INK6
CDKN2D	cyclin dependent kinase inhibitor 2D, INK, INK4D, p1, p19, p19IN, p19-INK4D
CFLAR	2310024N18RIK, A430105C05Rik, AU021929, Ca, CASH, CASP8 and FADD-like apoptosis regulator, CASP8AP1, Caspase 8 associated, Casper, c-F, c-FLIP, CLARP, F, FLAME, FLAME-1, FLICE-LIKE IP, FLIP, Gm9845, I-FLICE, LOC102724614, MRIT
CHEK1	C85740, checkpoint kinase 1, CHK1, rad27
CHEK2	CDS1, Check2, checkpoint kinase 2, CHK2, hCds1, HUCDS1, LFS2, PP1425, Rad, RAD53
CREBBP	AW558298, CB, CBP, CBP/p300, CREB binding protein, KAT, KAT3A, MKHK1, p300/CBP, RSTS, RSTS1, RTS
CRK	c-Crk, c-Crk2, Cr, CRK2, Crko, CRK proto-oncogene, adaptor protein, FLJ11558, p38, v-crk avian sarcoma virus CT10 oncogene homolog
CTNNB1	armadillo, Beta-cat, beta CATENIN, Bfc, Cat, CATENIN beta, catenin beta 1, catenin (cadherin associated protein), beta 1, catenin (cadherin associated protein), β 1, CATENIN β, catenin β 1, CATNB, CTNB1, CTNNB, CTNN beta, CTNN β, EVR7, Mesc, MRD19, NEDSDV, β-cat, β-catenin
CTNND1	AA409437, AU019353, BCDS2, Ca, CAS, catenin (cadherin associated protein), delta 1, catenin (cadherin associated protein), δ 1, catenin delta 1, catenin δ 1, CATNS, Ctn, CTNND, CTNN delta, CTNN delta1, CTNN δ, CTNN δ1, mKIAA0384, P12, P120, P120CAS, p120(CAS), p120-Catenin, P120CTN, p120(CTN), Pp120
CTNNα-CTNN β-CTNNδ	CTNNalpha-CTNNbeta-CTNNdelta
CyclinD	CycD, Cyclin D1
CYCS	CYC, CYCSA, CYTC, CYTOC, CYTOCHROME C, cytochrome c, somatic, cytochrome c, somatic-like, ENSMUSG00000058927, HCS, LOC100363502, THC4, X laevis XLCL2
DAG	DAG, diacylglycerides, diglyceride
DAXX	BING2, DAP6, death-domain associated protein, EAP1, Fas death domain-associated protein, PML ASSOCIATED FACTOR
DIABLO	0610041G12Rik, 1700006L01RIK, AU040403, DFNA64, diablo IAP-binding mitochondrial protein, diablo, IAP-binding mitochondrial protein, Sm, SMAC
DVL1	DISHEVELED, dishevelled segment polarity protein 1, DRS2, DSH, DVL, DVL1L1, DVL1P1, mKIAA4029
ELK1	ELK, ELK1, member of ETS oncogene family, ETS transcription factor ELK1, p62TCF, RGD:2549, TCF/ELK
EP300	A430090G16, A730011L11, E1A binding protein p300, KAT3, KAT3B, MKHK2, p30, p300, p300 HAT, RSTS2
ERK1/2	MAPK p44/42, MAPK p44/p42, p42/44 mapk, P42/p44 erk, P42/p44 mapk, p42/p44 MAP KINASE
FADD	DEATH domain-containing ADAPTOR, Fas associated via death domain, Fas (TNFRSF6)-associated via death domain, GIG3, MORT1, Mort1/F, Mort1/FADD
FANCD2	2410150O07RIK, AU015151, BB137857, FA4, FACD, FA complementation group D2, FAD, FA-D2, FANCD, Fanconi anaemia, complementation group D2, Fanconi anemia, complementation group D2
FAS	AI196731, ALPS1A, AP, APO-1, APT1, CD95, CD95L, CD95 receptor, FAS1, FAS/APO1, Fas cell surface death receptor, FasR, FASTM, Fas (TNF receptor superfamily member 6), lpr, Receptor for Fas Ligand, Receptors for Fas Ligand, TNF, Tnfr, TNFR6, Tnf receptor member 6, TNFRSF6
FASLG	ALPS1B, APT1, APT1LG1, APTL, CD178, CD95, CD95-L, F, Fa, FASL, Fas Ligand, Fas ligand (TNF superfamily, member 6), gld, mFasL, Tnfl6, Tnfs, TNFSF6, TNLG1A
FOS	AP-1, c-f, C-FOS, D12Rfj, D12Rfj1, FBJ osteosarcoma oncogene, Fos proto-oncogene, AP-1 transcription factor subunit, p55
FOXO1	Afx, Afxh, AI876417, FKH, FKH1, FKHR, FKHR1, Forkhead, forkhead box O1, Fox, FOXO1A
Frizzled	Frizzled receptor, FZ, FZD, Wnt receptor
Frizzled-LRP	FZD-LRP1/5/6
FYN	AI448320, AW552119, C-FYN, Fyn proto-oncogene, FYN proto-oncogene, Src family tyrosine kinase, FYNT, LOC102724705, p59-FYN, p59 Fyn B, SLK, SRC-LIKE KINASE, SYN
G protein	Galphabetagamma, Galpha-Gbeta-Ggamma, Galphai-Gbeta-Ggamma, Galphaq-Gbeta-Ggamma, Gpro, G protein alpha beta gamma, G protein alpha-G protein beta-GDP-G protein gamma, G protein alpha-G protein beta-G protein gamma, G-protein complex, G protein α-G protein β-GDP-G protein γ, G protein α-G protein β-G protein γ, G-protein α-β-γ, Guanine nucleotide binding protein, Gα-Gβ-Gγ, Gαi-Gβ-Gγ, Gαq-Gβ-Gγ, Gαβγ
G proteinalpha	Galpha, G-Protein Alpha Subunit, G protein α, G-Protein α Subunit, Gα
Gammasecretase	Gamma Secretase, Secretase γ, γ-Secretase
GLI1	AV235269, GL1, GLI, GLI family zinc finger 1, GLI-Kruppel family member GLI1, PAPA8, PPD1, Zfp-, ZFP5
GRB2	AA408164, ASH, Ash-psi, EGFRBP-GRB2, GRAB2, GRBS, growth factor receptor bound protein 2, MST084, MSTP084, NCKAP2
Grb2-Shc1-Sos	Grb2-Sos-Shc, SHC-GRB2-SOS
GSK3	Glycogen synthase kinase, Gsk, GSK3 alpha/beta, GSK3 α/β
GSK3beta-Axin-APC-Ctnnbeta	APC-CTNNbeta-AXIN-GSK3beta, APC-CTNNβ-AXIN-GSK3β, AXIN-APC-GSKbeta-CTNNbeta, AXIN-APC-GSKβ-CTNNβ, GSK3β-AXIN-APC-CTNNβ
GSK3B	7330414F15Rik, 8430431H08Rik, C86142, glycogen synthase kinase 3 beta, glycogen synthase kinase 3 β, GSK-, GSK-3, GSK-3be, GSK-3beta, GSK-3β, GSKbeta, GSKβ, Tpk1
HAT1	2410071B14Rik, AA536933, histone acetyltransferase 1, histone aminotransferase 1, KAT, KAT1
Hedgehog	Hh
HIF1A	AA959795, bHLHe7, bHLHe78, HIF-1, HIF1-ALPHA, HIF-1alpha (hydroxylated), HIF-1-α, HIF-1α (hydroxylated), Hypoxia inducible factor 1 alpha subunit, hypoxia inducible factor 1, alpha subunit, hypoxia inducible factor 1 subunit alpha, hypoxia inducible factor 1 subunit α, Hypoxia inducible factor 1 α subunit, hypoxia inducible factor 1, α subunit, MO, MOP1, PASD8

Symbol	Synonym(s)
HIF1alpha-NICI	HIF1α-NICD
HIPK2	1110014O20RIK, B230339E18RIK, homeodomain interacting protein kinase 2, LOC100505582, LOC653052, PRO0593, St, Stank
Ikb	I KAPPA B, Ikbeta, Ikβ, Iκ-B
IkB-NfkB	IkappaB-NFkappaB, IκB-NFκB, NFkB-IkB
Integrin	Integrin alpha-beta, integrin-extracellular matrix, INTEGRIN receptor, Integrin α-β
IP3	27121-73-9, inositol trisphosphate, IP3, myo-inositol, tris(dihydrogen phosphate)
IRS1	ENSMUSG00000022591, G972, G972R, HIRS-1, insulin receptor substrate 1, IR, IRS1IRM
JAK	JAK kinase
Jnk	JNK 54/46, Jnk p56, JNK/SAPK, JUN KINASE, p40, p47, Sapk/Jnk
JUN	Activator protein 1, AP-1, API-1, c-ju, cJUN, Junc, jun proto-oncogene, Jun proto-oncogene, AP-1 transcription factor subunit, LOC100288387, LOC100291417, LOC100293034, p39, v-Jun, V-jun Avian Sarcoma Virus 17 Oncogene Homolog, V-jun Sarcoma Virus 17 Oncogene Homolog
LAMTOR3	AW556229, late endosomal/lysosomal adaptor, MAPK and MTOR activator 3, LOC100132990, Map, Map2k, MAP2K1IP1, MAPBP, MAPKSP1, Mek binding partner 1, Mp, MP1, PRO0633, Ragulator3
LEF1	3000002B05, AI451430, Lef-, lymphoid enhancer binding factor 1, TCF10, TCF1ALPHA, TCF7L3, TCF/LEF
MAP2K1/2	MEK1/2, MKK1/2
MAP3K5	7420452D20Rik, A, APOPTOSIS SIGNAL REGULATED KINASE 1, AS, ASK, ASK1, M3K5, MAPKKK5, MEKK5, mitogen-activated protein kinase kinase kinase 5, RGD1306565
MAP3K7	CSCF, FMD2, Map3k7 predicted, MEKK7, mitogen-activated protein kinase kinase kinase 7, TAK1, TGF1a, tgf β activated kinase 1
Map3k7-Map3k7ip1-	Tab1-Tab2-Tak1
MAX	AA960152, AI875693, bHLHd, bHLHd4, bHLHd5, bHLHd6, bHLHd7, bHLHd8, Max protein, MYC associated factor X, Myn
Max-Myc	cMyc-MAX, Myc-MAX
MDM2	1700007J15Rik, AA415488, ACTFS, hdm2, HDMX, LSKB, MDM2-A1, MDM2 proto-oncogene, MGC5370, Transformed 3t3 cell double minute 2, transformed mouse 3T3 cell double minute 2
Mkk3/6	MEK3/6, Mkk3/6 (mitogen activated protein kinase kinase 3/6), MKK3/MKK6
MYC	AU016757, bHLHe3, bHLHe39, CMYC, C-MYC-P64, mMyc, MRTL, Myc2, MYCC, MYC proto-oncogene, bHLH transcription factor, myelocytomatosis oncogene, N, Niard, Nird, RNCMYC
NBN	ATV, AT-V1, AT-V2, Nb, NBS, NBS1, NIBRIN, P95
NCSTN	9430068N19Rik, AA727311, APH2, ATAG1874, D1Dau13, D1Dau13e, Kiaa0253, mKIAA0253, Nc, NCT, ni, NICASTRIN
NF1	AW494271, Dsk, Dsk9, E030030H24RIK, LOC646021, Mhdads, Mhdadsk9, Neurofibromatosis 1, NEUROFIBROMIN, neurofibromin 1, Nf-, NF1-GAP, NFNS, VRNF, WSS
NFkB	NF-KAPPA B, NF-κ B, nuclear factor-κ b, transcription factor nuclear factor κ b
NLK	AI194375, LOC100044468, nemo like kinase, RGD1561602
NOTCHIC	9930111A19Rik, AOS5, AOVD1, hN1, lin, lin-12, Mi, Mis6, N, N1, NOTCH, notch receptor 1, Ta, TAN1
p38MAPK	P38, p38 MAP KINASE, P38 MITOGEN-ACTIVATED protein KINASE
Patched	PTC, PTCH
PI3K	1-phosphatidylinositol 3-kinase, 2.7.1.137, ATP:1-phosphatidyl-1D-myo-inositol 3-phosphotransferase, Phosphatidylinositol 3 kinase, phosphatidylinositol 3'-kinase, PI3-kinase, PtdIns 3 Kinase, type III phosphoinositide 3-kinase, type I phosphatidylinositol kinase, Vps34p
PIP2	1,2-diacyl-sn-glycero-3-phospho-(1'-myo-inositol-4',5'-bisphosphate), 1-O-(3-sn-phosphatidyl)-1D-myo-inositol 4,5-bis(dihydrogen phosphate), 1-phosphatidyl-1D-myo-inositol 4,5-bisphosphate, C11H19O19P3R2
PIP3	1-phosphatidyl-1D-myo-inositol 3,4,5-trisphosphate, phosphatidylinositol-3,4,5-trisphosphate, phosphoinositide (3,4,5) P3, PI(3,4,5)P3, PIns(3,4,5)P3, PIP3, PtdIns(3,4,5)P3
PKA	A-Kinase, cAMP-Dependent Protein Kinase, cyclic AMP depended protein kinase, protein KINASE A
PKC	Cnpkc, PKC, Pkc(s), Protein Kinase C
Plcbeta	Phospholipase c beta, Phospholipase C β, PLCB, PLCβ
PMAIP1	APR, N, NOXA, phorbol-12-myristate-13-acetate-induced protein 1
Presenilin	PS, PS1/2, PSEN1/2
PRKDC	AI326420, AU019811, DNA-, DNA-DEPENDENT protein KINASE, DNAPDcs, DNAPK, DNA-PKC, DNA-PKcs, DNPK1, DOX, DOXNPH, dxn, dxnph, HYRC, HYRC1, IMD26, p350, p460, Prkdc predicted, protein kinase, DNA activated, catalytic polypeptide, protein kinase, DNA-activated, catalytic subunit, scid, slip, XRCC, XRCC7
PSENEN	1700023M09RIK, ACNINV2, MDS033, MSTP064, PEN-2, presenilin enhancer gamma secretase subunit, presenilin enhancer, gamma-secretase subunit, presenilin enhancer γ secretase subunit, presenilin enhancer, γ-secretase subunit, RGD1312037
PTCH1	A230106A15RIK, BCNS, mes, NBCCS, patched 1, Pt, PTC, PTC1, PTCH, Ptch2, wi, wig
PTK2	FA, Fad, FADK, FADK 1, FAK, FAK1, FAK related non-kinase, FR, p125FAK, pp125FAK, PPP1R71, protein tyrosine kinase 2, PTK2 protein tyrosine kinase 2, TYROSINE KINASE 2
PTPN11	2700084A17Rik, AW536184, BPTP3, CFC, JMML, METCDS, MGC14433, Noonan syndrome 1, NS1, protein tyrosine phosphatase non-receptor type 11, protein tyrosine phosphatase, non-receptor type 11, PTP, PTP-1D, PTP2C, S, SAP-2, Sh, SH-P, SHP-2, SH-PTP2, SH-PTP3, Src homology protein 2, SYP
RAF1	6430402F14Rik, AA990557, BB129353, CMD1NN, c-R, Cra, CRAF, Craf1, D830050J10Rik, leukaemiA ONCOGENE HOMOLOG1, LEUKEMIA ONCOGENE HOMOLOG1, NS5, Raf-1 proto-oncogene, serine/threonine kinase, v-, v-Raf, v-raf-leukaemia viral oncogene 1, v-raf-leukemia viral oncogene 1
Ral	Ral A/B
RALBP1	DNP-SG ATPase, R, ralA binding protein 1, Ral GAP, Rik, RIP1, RL, RLIP1, RLIP76
RALGDS	Gn, Gnds, Hs.560937, mKIAA1308, Ra, RalGDSB, RalGEF, ral guanine nucleotide dissociation stimulator, Rg, RGDS, RGF
RAPGEF1	4932418O06Rik, C3G, C3G-1, C3G-2, Grf, GRF2, Rap guanine nucleotide exchange factor 1, Rap guanine nucleotide exchange factor (GEF) 1
RAPGEF3	2310016P22Rik, 9330170P05Rik, bcm910, CAMP-GEFI, CGEF1, Epa, EPAC, EPAC1, HSU79275, Rap guanine nucleotide exchange factor 3, Rap guanine nucleotide exchange factor (GEF) 3
Ras-grf	Guanine Nucleotide Releasing
RASA1	CM-AVM, CMAVM1, G, GAP, GAPX, p120-, p120GAP, P120RASGAP, PKWS, RASA, RASGAP, RAS p21 protein activator 1
RASGRP1	CALDAG-GEFI, CALDAG-GEFII, IMD64, RASGRP, RAS guanyl releasing protein 1
Rb-E2Ftranscriptionrepression	Rb1-E2f1, Rb-E2F, Rb-E2F1
RB1	OSRC, p, p105, p105-Rb, p110 RB, p110-RB1, pp105, pp110, PPP1R130, pRb, R, RB, RB-ASSOCIATED, RB transcriptional corepressor 1, Retinoblastome tumor-suppression protein rb
RBL1	AW547426, CP107, LOC683869, p10, p107, PRB1, RB transcriptional corepressor like 1
RBPJ	AI843960, AOS3, CBF-1, csl, Igk, Igkj, IGKJRB, IGKJRB1, Igkrsbp, KBF2, RBP, RBP 2N, RBP-JK, RBP-J kappa, RBP-J κ, RBPSUH, Rbpsuh1, Recombination signal binding, recombination signal binding protein for immunoglobulin kappa J region, recombination signal binding protein for immunoglobulin κ J region, SUH
RHO	GTPase Rho, Rho, Rho Family, RHO-GTPASE, Rho-like Gtpase
SHC1	p52SHC, p6, p66, p66s, P66shc, Sh, SHC, Shc (46 kDa isoform), SHCA, SHC adaptor protein 1, Shc p66 isoform, src homology 2 domain-containing transforming protein C1
Smad1/5/8	SMAD1/5/8
Smad1/5/8-	SMAD 1,4,5,8
Smad2/3-	Smad 2/3/4
SMAD4	AW743858, D18Wsu70, D18Wsu70e, DPC, DPC4, JIP, Madh, MADH4, MYHRS, SMAD family member 4, Smaug1
SMAD6	AOVD2, b2b390C, b2b390Clo, HsT17432, Madh, MADH6, MADH7, SMAD family member 6
SMAD7	CRCS3, Madh, MADH7, MADH8, SMAD family member 7
SMO	bnb, CRJS, E130215L21Rik, FZD11, Gx, PHLS, SMOH, Smoothened, smoothened, frizzled class receptor
SRC	ASV, AW259666, BS27, c-SRC, p60-Src, PP60, Pp60/c-Src, pp60c, pp60c-src, Rous sarcoma oncogene, SRC1, SRC proto-oncogene, non-receptor tyrosine kinase, THC6, TVHUSC
STK36	1700112N14RIK, B930045J24, FU, Fuse, Fused, mKIAA1278, serine/threonine kinase 36, Stk36 (predicted)
SUFU	b2b273C, JBTS32, PRO1280, Su, SUFUH, SUFU negative regulator of hedgehog signaling, SUFUXL
SYNGAP1	Gm1963, MRD5, RASA1, RASA5, Sy, Synaptic Ras-GAP 1, synaptic Ras GTPase activating protein 1, synaptic Ras GTPase activating protein 1 homolog (rat), SYNGAP
TAB2	1110030N06Rik, A530078N03Rik, CHTD2, LOC101928709, Map3k, MAP3K7IP2, mKIAA0733, RP1 111D63, TGF-beta activated kinase 1 (MAP3K7) binding protein 2, TGF-beta activated kinase 1/MAP3K7 binding protein 2, TGF-β activated kinase 1 (MAP3K7) binding protein 2, TGF-β activated kinase 1/MAP3K7 binding protein 2
TBID	2700049M22Rik, AI875481, AU022477, BH3 interacting domain death agonist, cBid, FP497
Tgfbeta	Tgfb, TGF-beta 1, 2, and 3, TGF β, TGF-β 1, 2, and 3, transforming growth factor-β
Tgfbetarecept	TGFbetaR, TGFBR, TGFβR, Tgf β receptor
TGFBR1	AAT5, ACVRLK4, AL, Alk, ALK-5, AU017191, ESK2, ESS1, LDS1, LDS1A, LDS2A, LOC103690035, LOC666236, MSSE, SKR4, Tbet, Tbeta, TbetaR-I, TBR-I, TGFbeta1R1, TGF-beta1 receptor, TGF beta I receptor, Tgf-beta-r1, TGF beta receptor type I, TGFbetaRI, Tgfb receptor I, TGFR-1, TGFβ1R1, TGF β I receptor, TGFβ R1, Tgf -β R1/R4, Tgf-β receptor1, TGF β receptor type I, TGFβRI, TGF β type 1 receptors, Tgf β type i receptor, transforming growth factor beta receptor 1, transforming growth factor, beta receptor 1, transforming growth factor, beta receptor I, transforming growth factor β receptor 1, transforming growth factor, β receptor 1, transforming growth factor, β receptor I, transforming growth factor-β receptor type 1, T β R1
TGFBR2	1110020H15Rik, AAT3, AU042018, DNIIR, FAA3, LDS1B, LDS2, LDS2B, MFS2, RIIC, RIIDN, TAAD2, Tbet, Tbeta, TbetaR-II, TBR, TBR-II, TBRLL, TGFbeta1R2, TGF-beta 2, Tgf beta2 receptor, TGFbeta R2, TGF-beta receptor 2, TGF-beta receptor type 2, TGF beta receptor type II, TGFbeta-RII, Tgfbr2T, TGFB receptor II, Tgfbrii, TGFR-2, TGFβ1R2, TGF-β 2, TGFβ R2, TGF-β receptor 2, TGF-β receptor type 2, TGF β receptor type II, TGFβRII, Tgf-β type ii receptor, transforming growth factor beta receptor 2, transforming growth factor, beta receptor 2, transforming growth factor, beta receptor II, transforming growth factor β receptor 2, transforming growth factor, β receptor 2, transforming growth factor, β receptor II, type 2 TGF-β receptors, T β r2
TP53	bbl, BCC7, bfy, bhy, BMFS5, LFS1, p4, p44, p5, P53, P53 cellular tumour antigen, p53 tumor suppressor, transformation related protein 53, TRP53, tumor protein p53, tumour protein p53
Type I BMP	ALK 3,6, ALK 3/6, BMP2 receptor type1, BMP4 receptor type1, Bmpr1, Bmpri
WNT1	BMND16, Int, INT1, OI15, sw, swaying, Wg, wingless-type MMTV integration site family, member 1, Wnt-, Wnt family member 1
WNT5A	8030457G12Rik, hWNT5A, LOC102724616, wingless-type MMTV integration site family, member 5A, Wnt-, Wnt family member 5A
ZBTB17	AA589413, Lp-1, Miz, MIZ-1, mZ13, pHZ-67, Zfp10, Zfp100, zinc finger and BTB domain containing 17, ZNF151, ZNF60

Pathway Analysis Using IPA Software; canonical pathway

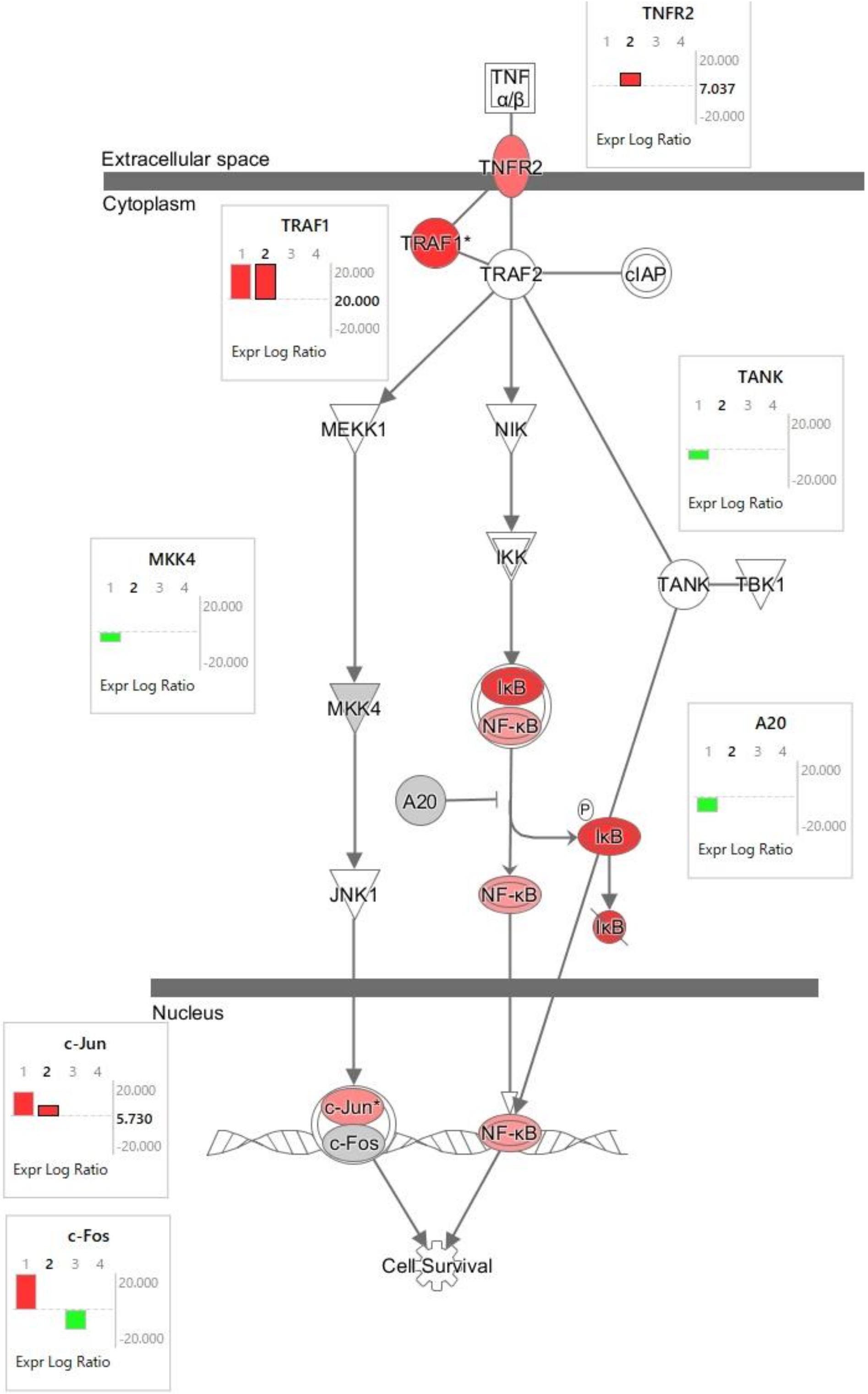

Figure S7. TNFR2 Signaling

1. 1 h; 2. 6 h; 3. 24 h; 4. 8 days.

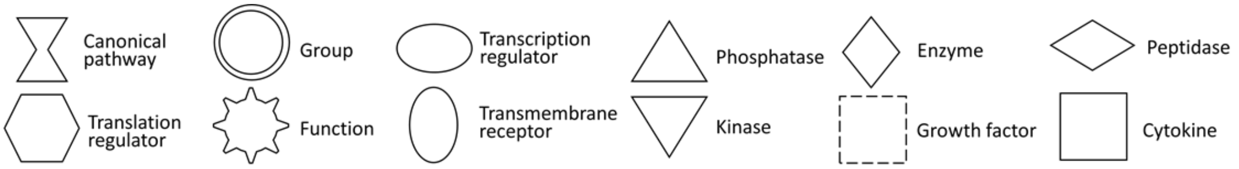

Red: Increased, FDR<0.05 versus solvent control

Green: Decreased, FDR<0.05 versus solvent control

Symbol	Synonym(s)
Ap1	activator protein-1, c-Jun
c-Iap	IAP, NAIP
FOS	AP-1, c-f, C-FOS, D12Rfj, D12Rfj1, FBJ osteosarcoma oncogene, Fos proto-oncogene, AP-1 transcription factor subunit, p55
Ikb	I KAPPA B, Ikbeta, Ikβ, Iκ-B
IkB-NfkB	IkappaB-NFkappaB, IκB-NFκB, NFkB-IkB
JUN	Activator protein 1, AP-1, API-1, c-ju, cJUN, Junc, jun proto-oncogene, Jun proto-oncogene, AP-1 transcription factor subunit, LOC100288387, LOC100291417, LOC100293034, p39, v-Jun, V-jun Avian Sarcoma Virus 17 Oncogene Homolog, V-jun Sarcoma Virus 17 Oncogene Homolog
MAP2K4	JNKK, JNKK1, MAPK/ERK KINASE-1, MAPKK4, MEK4, mitogen-activated protein kinase kinase 4, MKK4, PRKMK4, SAPKK-1, Sek, SEK1, Ser, SERK1, SKK1
MAP3K1	LOC100912399, MAPK, MAPKKK1, MEKK, MEKK 1, MEK KINASE, MEK KINASE 1, mitogen-activated protein kinase kinase kinase 1, mitogen-activated protein kinase kinase kinase 1-like, Raf, SRXY6
MAP3K14	aly, FTDCR1B, HS, HSNIK, mitogen-activated protein kinase kinase kinase 14, N, NFKB INDUCING KINASE, nf κ b inducing kinase, NIK
MAPK8	AI849689, C-JUN N-TERMINAL KINASE1, JNK, JNK1, JNK1A2, JNK21B1/2, JNK-46, mitogen-activated protein kinase 8, p46JNK1, p46JNK1 alpha, p46JNK1 α, Prk, PRKM8, SAPK1, SAPK1c, Sapk gamma, SAPK P46, Sapk γ, STRESS-ACTIVATED protein KINASE-LIKE KINASE
NFkB	NF-KAPPA B, NF-κ B, nuclear factor-κ b, transcription factor nuclear factor κ b
TANK	C86182, E430026L09Rik, I-T, I-TRAF, TRAF2, TRAF family member-associated Nf-kappa B activator, TRAF family member-associated NFKB activator, TRAF family member-associated Nf-κ B activator, TRAF-INTERACTING protein 1-TRAF
TBK1	1200008B05Rik, AI462036, AW048562, FTDALS4, IIAE8, LOC299827, NAK, T2K, TANK-binding kinase 1, Tbk
TNF-A/B	TNF alpha/beta, TNF α/β
TNFAIP3	A2, A20, AISBL, MAD6, OTUD7C, Tnf, TNFA1P2, TNF alpha-INDUCED protein 3, TNF-inducible early response, Tnfip3, TNF α-INDUCED protein 3, tumor necrosis factor, alpha-induced protein 3, tumor necrosis factor, α-induced protein 3, tumour necrosis factor, alpha-induced protein 3, tumour necrosis factor, α-induced protein 3
TNFRSF1B	CD120b, p7, p70TNFR, p75, p75TNFR, p80, P80 TNF receptor, TBPII, TN, TNF-, TNF-a, TNFaI, TNF-alphaR2, TNF alpha RII, TNFBR, TNF-R, Tnfr-1, TNFR1B, TNF-R2, TNF-R75, TNFR80, TNF receptor superfamily member 1B, TNF-R-II, TNF-RII/TNFRSF1B, TNFR P75, TNF α RII, tumor necrosis factor receptor superfamily, member 1b, tumour necrosis factor receptor superfamily, member 1b
TRAF1	4732496E14Rik, EBI6, ineligibletraf1, MGC:10353, TNF receptor-associated factor 1
TRAF2	AI325259, MGC:45012, RNF117, TNF receptor-associated factor 2, TRAP, TRAP3

Pathway Analysis Using IPA Software; canonical pathway

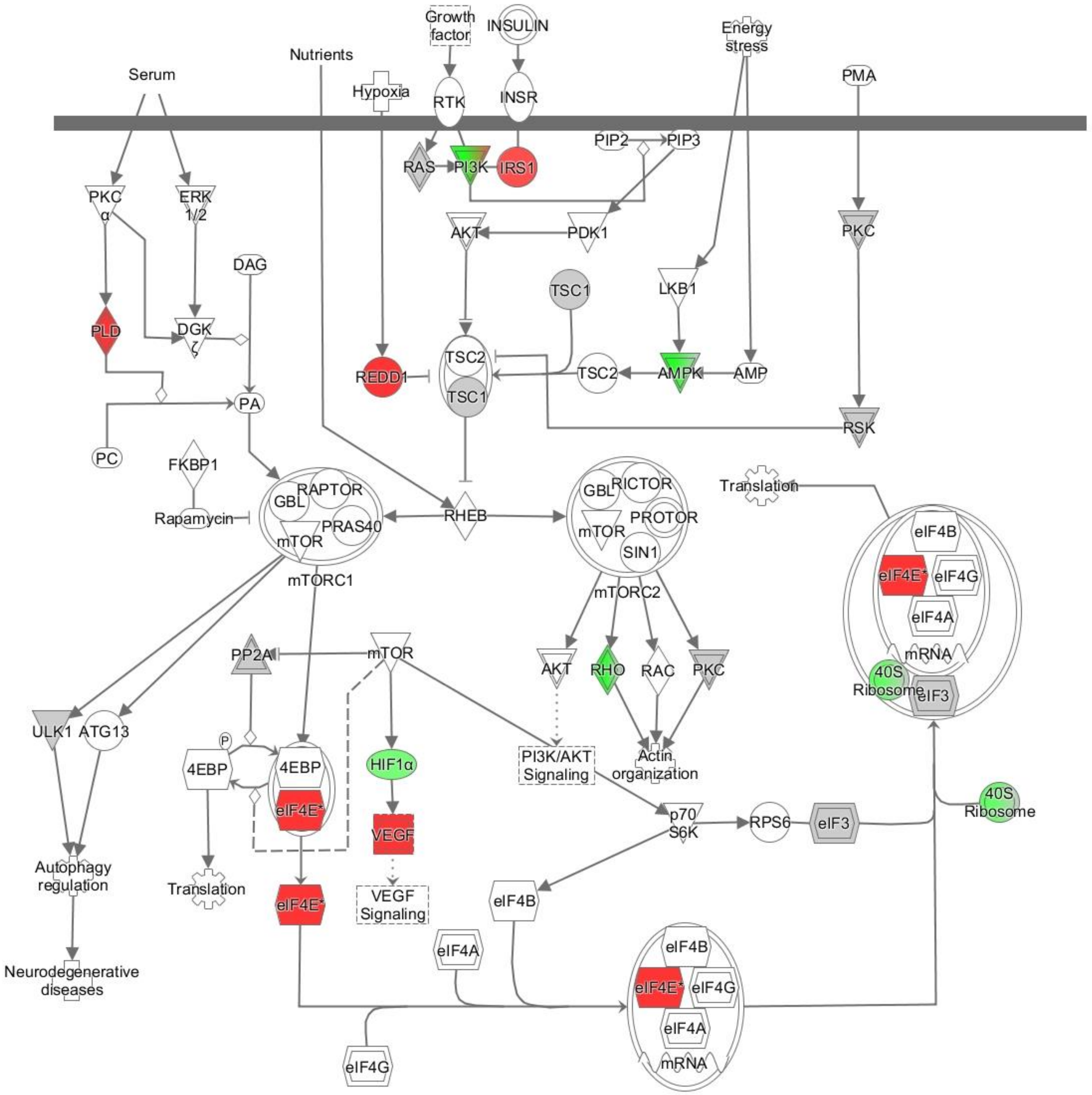

Figure S8. mTOR Signaling at 1 h

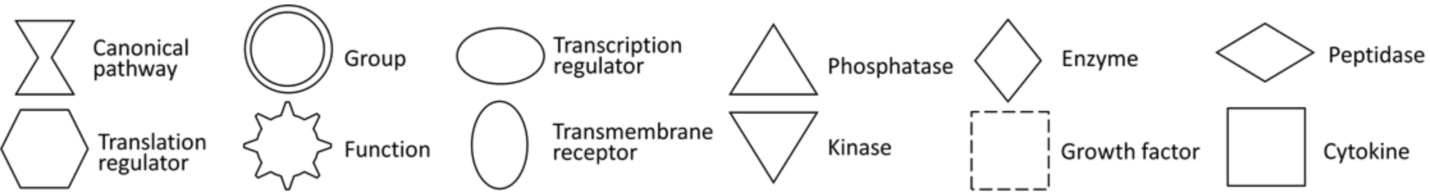

Red: Increased, FDR<0.05 versus solvent control

Green: Decreased, FDR<0.05 versus solvent control

Symbol	Synonym(s)
4EBP-eIF4E	eIF4E-eIF4EBP
AKT	AKT1/2/3, B/Akt, PKB, RAC-PK
AKT1S1	1110012J22RIK, AI227026, AI430011, AKT1 substrate 1, AKT1 substrate 1 (proline-rich), Lo, Lobe, Lobel, PR, PRAS, PRAS40, Proline-rich AKT substrate
AMP	149022-20-8, [(2R,3S,4R,5R)-5-(6-aminopurin-9-yl)-3,4-dihydroxyoxolan-2-yl]methyl dihydrogen phosphate, 5'-adenylic acid, 5' AMP, 5'-AMP, 61-19-8, adenosine-5-monophosphate, adenosine-5-phosphate, adenosine monophosphate, C10H14N5O7P
AMPK	AMP-activated kinase, AMP KINASE, Amp-pk
ATG13	1110053A20Rik, autophagy related 13, D2Ertd391, D2Ertd391e, Harbi1, Harbi1l, KIAA0652, PARATARG8, RGD1310685
DAG	DAG, diacylglycerides, diglyceride
DDIT4	5830413E08Rik, AA415483, Dig, Dig2, DKFZP564O2071, DNA-damage-inducible transcript 4, FLJ20500, REDD, REDD-1, Rtp8, Rtp801
DGKZ	80-kDa Dg Kinase, DAGK5, DAGK6, Dgk4, DGK-ZETA, DGK-ζ, Diacylglycerol kinase, diacylglycerol kinase zeta, diacylglycerol kinase ζ, E130307B02Rik, F730209L11Rik, hDGKzeta, KDGZ, mDGK[z]
Eif-4a	Eukaryotic translation initiation factor 4a, Homologous to SP P44586 ATP-dependent RNA helicase DEAD
EIF4B	2310046H11RIK, AL024095, C85189, Eif4a2, eIF4B, eukaryotic translation initiation factor 4B, Initiation Factor M3, PRO1843
EIF4E	AUTS19, CAP-binding, CBP, EG668879, eIF-4, EIF4E1, EIF4EL1, Eif4e-ps, EIF4F, eukaryotic translation initiation factor 4E, If4, If4e
EIF4EBP1	4e-bp, 4E-BP1, AA959816, BP-1, Eukaryotic translation initiation factor 4e binding protein 1, PH, PHAS-I
eIF4G	eIF4gamma, eIF4γ
ERK1/2	MAPK p44/42, MAPK p44/p42, p42/44 mapk, P42/p44 erk, P42/p44 mapk, p42/p44 MAP KINASE
FKBP1A	FK506 binding protein 1a, Fkb, Fkbp, FKBP1, FKBP-12, Fkbp2, FKBP prolyl isomerase 1A, FPK1, macrophilin-12, PKC12, PKCI2, PPIASE
HIF1A	AA959795, bHLHe7, bHLHe78, HIF-1, HIF1-ALPHA, HIF-1alpha (hydroxylated), HIF-1-α, HIF-1α (hydroxylated), Hypoxia inducible factor 1 alpha subunit, hypoxia inducible factor 1, alpha subunit, hypoxia inducible factor 1 subunit alpha, hypoxia inducible factor 1 subunit α, Hypoxia inducible factor 1 α subunit, hypoxia inducible factor 1, α subunit, MO, MOP1, PASD8
INSR	4932439J01Rik, alpha subunit INSULIN receptor, CD220, D630014A15RIK, HHF5, I, insulin receptor, INSULIN receptor B, Insulin receptor beta, INSULIN receptor KINASE, Insulin receptor β, INSULIN RPTK, IR, IR alpha, IR-B, IRK, IR α, α subunit INSULIN receptor
INSULIN	Ins, Ins1/2, proinsulin
IRS1	ENSMUSG00000022591, G972, G972R, HIRS-1, insulin receptor substrate 1, IR, IRS1IRM
MAPKAP1	AI591529, D230039K05Rik, JC310, MAPK associated protein 1, MIP1, mitogen-activated protein kinase associated protein 1, mSIN1, S, SIN1
MLST8	0610033N12Rik, AA409454, AI505104, AI851821, Gb, GbetaL, GBL, GβL, LST8, mLS, MTOR associated protein, LST8 homolog, MTOR associated protein, LST8 homolog (S. cerevisiae), POP3, WAT1
MTOR	2610315D21Rik, AI327068, fl, Flat, Fr, FRAP, FRAP1, FRAP2, FRB, mechanistic target of rapamycin kinase, RA, RAF, RAFT1, RAPT1, RRAFT1, SKS
PA	1,2-diacyl-sn-glycerol-3-phosphate, diacylglycerophosphates, PA, phospholipids alcohol, PtdOH
PC	3-sn-phosphatidylcholine, C10H18NO8PR2, choline glycerophospholipid, diacylglycerophosphocholines, lecithin, lecithins, lecithin, soy, lecithin, soybean, LT-02, PC, phosphotidylcholine, soya phosphatidyl choline, soybean phospholipids, soy lecithin
PDPK1	3'-PDK, 3-phosphoinositide dependent protein kinase-1, Pdk, PDK1, PDPK2, PDPK2P, PRO0461
PI3K	1-phosphatidylinositol 3-kinase, 2.7.1.137, ATP:1-phosphatidyl-1D-myo-inositol 3-phosphotransferase, Phosphatidylinositol 3 kinase, phosphatidylinositol 3'-kinase, PI3-kinase, PtdIns 3 Kinase, type III phosphoinositide 3-kinase, type I phosphatidylinositol kinase, Vps34p
PIP2	1,2-diacyl-sn-glycero-3-phospho-(1'-myo-inositol-4',5'-bisphosphate), 1-O-(3-sn-phosphatidyl)-1D-myo-inositol 4,5-bis(dihydrogen phosphate), 1-phosphatidyl-1D-myo-inositol 4,5-bisphosphate, C11H19O19P3R2
PIP3	1-phosphatidyl-1D-myo-inositol 3,4,5-trisphosphate, phosphatidylinositol-3,4,5-trisphosphate, phosphoinositide (3,4,5) P3, PI(3,4,5)P3, PIns(3,4,5)P3, PIP3, PtdIns(3,4,5)P3
PKC	Cnpkc, PKC, Pkc(s), Protein Kinase C
PLD	3.1.4.4, choline phosphatase, lecithinase D, lipophosphodiesterase II, phosphatidylcholine phosphatidohydrolase, PHOSPHOLIPASE D
PMA	12-O-tetradecanoylphorbol-13-acetate, 16561-29-8, [(1S,2S,6R,10S,11R,13S,14R,15R)-13-acetyloxy-1,6-dihydroxy-8-(hydroxymethyl)-4,12,12,15-tetramethyl-5-oxo-14-tetracyclo[8.5.0.02,6.011,13]pentadeca-3,8-dienyl] tetradecanoate, 4beta-PMA, beta-PMA, C36H56O8, myristic acid, 9-ester with 1,1a-alpha,1b-beta,4,4a,7a-alpha,7b,8,9,9a-decahydro-4a-beta,7b-alpha,9-beta,9a-alpha-tetrahydroxy-3-(hydroxymethyl)-1,1,6,8-alpha-tetramethyl-5H-cyclopropa(3,4)benz(1,2-e)azulen-5-one, 9a-acetate, myristic acid, 9-ester with 1,1a-α,1b-β,4,4a,7a-α,7b,8,9,9a-decahydro-4a-β,7b-α,9-β,9a-α-tetrahydroxy-3-(hydroxymethyl)-1,1,6,8-α-tetramethyl-5H-cyclopropa(3,4)benz(1,2-e)azulen-5-one, 9a-acetate, phorbol 12-myristate 13-acetate, phorbol myristate acetate, PMA, tetradecanoic acid, 9a-(acetyloxy)-1a,1b,4,4a,5,7a,7b,8,9,9a-decahydro-4a,7b-dihydroxy-3-(hydroxymethyl)-1,1,6,8-tetramethyl-5-oxo-1H-cyclopropa(3,4)benz(1,2-e)azulen-9-yl ester, (1aR-(1aalpha,1bbeta,4abeta,7aalpha,7balpha,8alpha,9beta,9aalpha))-, tetradecanoyl-phorbol-13-acetate, TPA, β-12-O-tetradecanoylphorbol-13-acetate, β-phorbol 12-myristate 13-acetate, β-phorbol-12 β-myristate-13 α-acetate, β-PMA
PP2A	protein PHOSPHATASE 2A, Protein Phosphatase Type2a
PRKCA	AAG6, AI875142, LOC146784, Pk, PKCA, PKC-alpha, PKCI+/-, PKC-α, PKRCA, PRKACA, protein kinase C alpha, protein kinase C, alpha, protein kinase C α, protein kinase C, α, α-protein kinase C
RAC1	AL023026, D5Ertd559, D5Ertd559e, MIG5, MRD48, p21-Rac1, p21-RAC, Rac, Rac family small GTPase 1, TC-25
Rapamycin	1402453-65-9, (1R,9S,12S,15R,16E,18R,19R,21R,23S,24E,26E,28E,30S,35R)-1,18-dihydroxy-12-[(2R)-1-[(1S,3R,4R)-4-hydroxy-3-methoxycyclohexyl]propan-2-yl]-19,30-dimethoxy-15,17,21,23,29,35-hexamethyl-11,36-dioxa-4-azatricyclo[30.3.1.04,9]hexatriaconta-16,24,26,28-tetraene-2,3,10,14,20-pentone, (3S,6R,7E,9R,10R,12R,14S,15E,17E,19E,21S,23S,26R,27R,34aS)-9,10,12,13,14,21,22,23,24,25,26,27,32,33,34,34a-Hexadecahydro-9,27-dihydroxy-3-[(1R)-2-[(1S,3R,4R)-4-hydroxy-3-methoxycyclohexyl]-1-methylethyl]-10,21-dimethoxy-6,8,12,14,20,26-hexamethyl-23,27-epoxy-3H-pyrido[2,1-c][1,4]oxaazacyclohentriacontine-1,5,11,28,29(4H,6H,31H)-pentone, 53123-88-9, ABI-009, AY 22-989, C51H79NO13, erapa, I-2190A, nab-rapamycin, nanoparticle albumin-bound rapamycin, NSC 226080, Rapamune, Rapamycin, SEL-110, SILA 9268A, SVP-rapamycin, WY-090217
RHEB	Ras homolog enriched in brain, Ras homolog, mTORC1 binding, RHEB1, RHEB2
RHO	GTPase Rho, Rho, Rho Family, RHO-GTPASE, Rho-like Gtpase
Ribosomal40s subunit	40s, 40S ribosomal subunit, 40S RIBOSOME
RICTOR	4921505C17Rik, 6030405M08Rik, AVO3, AW492497, D530039E11Rik, hAVO3, KIAA1999, Mtorc2, PIA, RPTOR independent companion of MTOR complex 2, RPTOR independent companion of MTOR, complex 2
RPS6	40S ribosomal protein S6-like, LOC100911372, pp33, Q9BZU1, RIBOSOMAL protein S6, S, S6, S6R, S6RP
RPS6KB1	2610318I15RIK, 4732464A07RIK, AA959758, AI256796, AI314060, P70, p70/85s, p70/85s6k, p70-alpha, p70s, p70S6, p70s6k, P70S6K1, p70 S6K-alpha, p70S6 kinase, p70 S6K-α, p70(S6K)-α, p70-α, PS6K, ribosomal protein S6 kinase B1, ribosomal protein S6 kinase, polypeptide 1, S6K, S6K1, S6K-beta-1, S6K-β-1, STK14A
RPTOR	4932417H02Rik, KOG1, Mip1, mKIAA1303, r, Rap, RAPTOR, regulatory associated protein of MTOR complex 1, regulatory associated protein of MTOR, complex 1, RGD1311784
RSK	p90RSK
STK11	AA408040, hLKB1, Lkb, LKB1, LKB1-L, LKB1(S), Pa, Par-4, PJS, R75140, serine/threonine kinase 11, Stk11 isoform 2, Stk11 short isoform
TSC1	ham, Hamartin, LAM, TSC, TSC complex subunit 1
Tsc1-Tsc2	TSC, TSC1/2
TSC2	LAM, Na, Nafld, PPP1R160, Rc, Tcs2, TSC4, TSC complex subunit 2, tube, TUBERIN
ULK1	ATG1, ATG1A, AU041434, hATG1, mKIAA0722, ULK, Ulk1 mapped, UNC51, Unc51., Unc51.1, unc-51 like autophagy activating kinase 1, unc-51 like kinase 1

Pathway Analysis Using IPA Software; canonical pathway

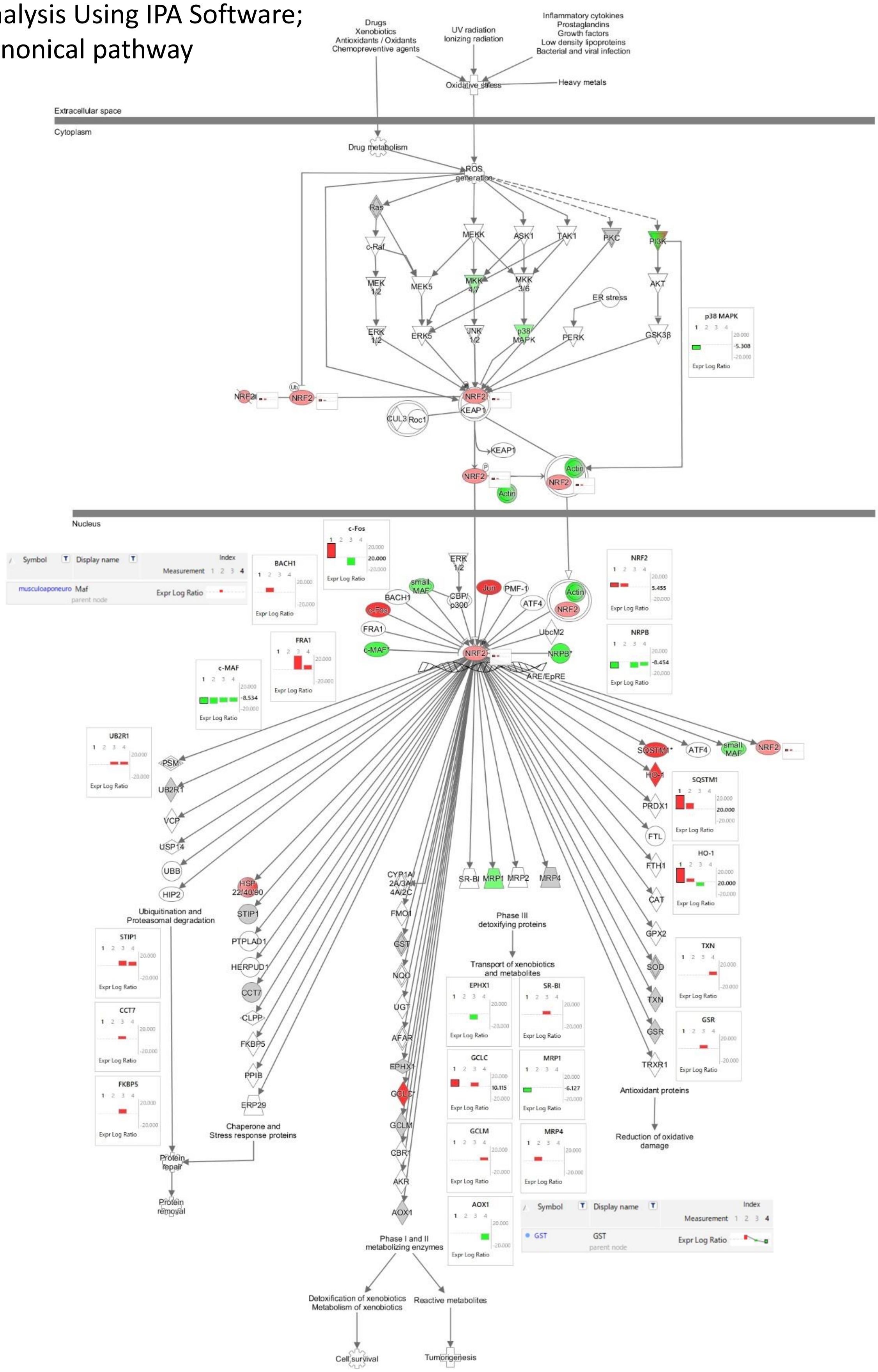

Figure S9. NRF2-mediated oxidative stress response

1. 1 h; 2. 6 h; 3. 24 h; 4. 8 days.

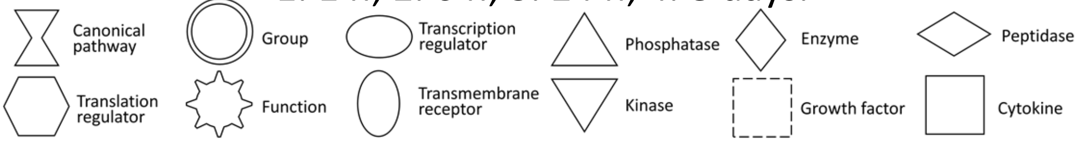

Red: Increased, FDR<0.05 versus solvent control

Green: Decreased, FDR<0.05 versus solvent control

Symbol	Synonym(s)
ABCC1	ABC29, ABCC, Abcc1a, Abcc1b, ATP-binding cassette, sub-family C (CFTR/MRP), member 1, ATP binding cassette subfamily C member 1, Avcc1a, DFNA77, GS-X, LOC100362747, LOC100365034, M, Md, Mdrap, Mr, MRP, MRP1
ABCC2	ABC30, AI173996, Atpase canalicular type, ATP-binding cassette, sub-family C (CFTR/MRP), member 2, ATP binding cassette subfamily C member 2, CANALICULAR MULTIDRUG RESISTANCE, Cmo, CMOAT, cMRP, DJS, Ebcr, Mr, mrp, MRP2
ABCC4	ABCC4-N1, ATP-binding cassette, sub-family C (CFTR/MRP), member 4, ATP binding cassette subfamily C member 4, D630049P08Rik, MOAT, MOAT-B, MR, MRP4, RGD1565953
Actin	CLEC9A Ligand, G-actin
AFAR	AFLATOXIN B1 ALDEHYDE REDUCTASE
AKR1A1	2610201A18Rik, Akr, AKR1A4, ALDEHYDE REDUCTASE, aldo-keto reductase family 1 member A1, aldo-keto reductase family 1, member A1 (aldehyde reductase), ALDR1, ALR, ARM, DD3, HEL-S-6, hs.474584
AKT1	Ak, AKT, AKT serine/threonine kinase 1, LTR-akt, PK, PKB, PKB/A, PKB/Akt, PKB-ALPHA, PKB-α, PRKBA, Protein kinase B, RAC, RAC-ALPHA, RAC-α, Thymoma proto-oncogene 1, thymoma viral proto-oncogene 1
AOX1	AI196512, AI255253, ALDEHYDE OXIDASE, aldehyde oxidase 1, AO, AOH1, Aox, Aox-2, Moro, Ro, XD
ATF4	activating transcription factor 4, Atf-, C/AT, C/ATF, CREB, CREB-2, TAXREB, TAXREB67, TXREB
BACH1	6230421P05RIK, AI323795, BTB and CNC Homology 1, BTB and CNC homology 1, basic leucine zipper transcription factor 1, BTBD24, BTB domain and CNC homolog 1, C21ORF41, LOC100911490
CAT	2210418N07, ACATALASIA, Ca, Cas, Cas-1, Cat01, Catalase, Catalase1, Catl, Cs-, CS1
CBP/p300	CBP, CBP-p300
CBR1	AW261796, C, Carbonyl Reductase, carbonyl reductase 1, carbonyl reductase [NADPH] 1-like, CBR, CR, CRN, hCBR1, inducible carbonyl reductase-like, LOC100360507, LOC100912203, LOC102556347, Ocr, PG-9-KR, SDR21C1
CCT7	AA408524, AL022769, CCTETA, CCTH, Cctz, Chaperonin 7, chaperonin containing TCP1 subunit 7, chaperonin containing Tcp1, subunit 7 (eta), Chaperonin subunit 7, NIP7-1, TCP1ETA
CDC34	AI327276, cell division cycle 34, cell division cycle 34, ubiqiutin conjugating enzyme, E2-CDC34, Serine Protease, UB2R1, UBC3, UBCH3, UBE2R, UBE2R1, Ubiquitin conjugating enzyme e2-32
CLPP	AU019820, caseinolytic mitochondrial matrix peptidase proteolytic subunit, D17Wsu160, D17Wsu160e, DFNB81, PRLTS3
CUL3	cullin 3, KIAA0617, PHA2E
Cul3-Roc1	Cul3-Rbx1
EIF2AK3	eukaryotic translation initiation factor 2 alpha kinase 3, eukaryotic translation initiation factor 2 α kinase 3, PE, PEK, PERK, WRS
ENC1	CCL28, ECTODERMAL-NEURAL CORTEX 1, KLHL35, KLHL37, N, NRPB, PIG, PIG10, TP53I10
EPHX1	EH, Ehm, Eph-, Eph-1, EPHX, EPOX, Epoxide Hydrolase, epoxide hydrolase 1, epoxide hydrolase 1, microsomal, EPXH1, HYL1, MEH, MEH8, Microsomal Colinesterase, MICROSOMAL EPOXIDE HYDROLASE
ERK1/2	MAPK p44/42, MAPK p44/p42, p42/44 mapk, P42/p44 erk, P42/p44 mapk, p42/p44 MAP KINASE
ERP29	1200015M03Rik, 2810446M09Rik, AW209030, C12orf8, endoplasmic reticulum protein 29, Erp, ERp28, ERp31, HEL-S-107, PDI-, PDIA9, PDI-DB
FKBP5	AIG6, D17Ertd592, D17Ertd592e, Dit, Dit1, FK506 binding protein 5, FKBP51, FKBP54, FKBP prolyl isomerase 5, P54, PPIase, Ptg-10
FMO1	flavin containing dimethylaniline monoxygenase 1, Flavin-containing monooxygenase, flavin containing monooxygenase 1, RFMO1A
FOS	AP-1, c-f, C-FOS, D12Rfj, D12Rfj1, FBJ osteosarcoma oncogene, Fos proto-oncogene, AP-1 transcription factor subunit, p55
FOSL1	AW538199, FOS like 1, AP-1 transcription factor subunit, fos-like antigen 1, Fr, FRA, FRA1
FTH1	APOFERRITIN H CHAIN, Ferritin heavy chain, ferritin heavy chain 1, ferritin heavy polypeptide 1, Ferritin subunit H, FHC, Frih, FTH, FTHL6, HFE5, H-fer, H-ferritin, HFt, hypothetical protein LOC689130, LOC689130, MFH, PIG15, PLIF
FTL	Ferritin, FERRITIN LIGHT CHAIN, ferritin light chain 1, ferritin light chain 1-like, ferritin light chain 1-like 1, ferritin light chain 2, ferritin light polypeptide 1, ferritin light polypeptide, pseudogene 2, FL, FRIL, Fril1, Ft, FTL1, Ftl1l1, Ftl1-ps2, Ftl-ps, Gm20746, L-fe, L-ferritin, LFTD, LOC100359668, LOC100360087, LOC100362384, LOC100363177, NBIA3, RGD1560687, RGD1561055, RGD1566189, similar to ferritin light chain, similar to Ferritin light chain (Ferritin L subunit), YB24D08
GCLC	D9Wsu168, D9Wsu168e, gamma GCS HEAVY CHAIN, Gamma Glutamylcysteine Synthetase, Gamma glutamylcysteine synthetase heavy subunit, Gamma Glutamyl Cysteine Synthetase Light Subunit, GCL, GCS, GCS, Catalytic, GCS-HS, Ggcs-hs, Glc, GLCL, GLCLC, GLCL-H, glutamate-cysteine ligase catalytic subunit, Glutamate-Cysteine Ligase, Catalytic Subunit, γ Gcs, γ-Gcsh, γ GCS HEAVY CHAIN, γ Glutamylcysteine Synthetase, γ glutamylcysteine synthetase heavy subunit, γ Glutamyl Cysteine Synthetase Light Subunit
GCLM	AI649393, Gamma gclm, gamma GCS LIGHT CHAIN, Gamma glutamylcysteine synthase (regulatory), gamma GLUTAMYLCYSTEINE SYNTHETASE, gamma-glutamylcysteine synthetase light (regulatory) subunit, Gcmc, Gcs-ls, Gcs, Regulatory, Gl, GLCLR, glutamat-cystein ligase, regulatory subunit, glutamate-cysteine ligase modifier subunit, Glutamate-Cysteine Ligase, Modifier Subunit, γ gclm, γ-GCS, γ GCS LIGHT CHAIN, γ glutamylcysteine synthase (regulatory), γ GLUTAMYLCYSTEINE SYNTHETASE, γ-glutamylcysteine synthetase light (regulatory) subunit
GPX2	GI-G, GI-GPx, glutathione peroxidase 2, GPRP, GPRP-2, GPx-, GPX-GI, GSHPx-2, GSHPX-GI
GSK3B	7330414F15Rik, 8430431H08Rik, C86142, glycogen synthase kinase 3 beta, glycogen synthase kinase 3 β, GSK-, GSK-3, GSK-3be, GSK-3beta, GSK-3β, GSKbeta, GSKβ, Tpk1
GSR	AI325518, D8Ertd238, D8Ertd238e, glutathione-disulfide reductase, Glutathione reductase, GR, Gr-1, Gred, GRX, GSRD, HEL-75, HEL-S-122m
GST	Glutathione s-transferase, GSH Transferase
HACD3	3-hydroxyacyl-CoA dehydratase 3, 4930523M17RIK, AW742319, B-in, B-IND1, Butyrate induced transcript 1, Hcad3, Hspc1, HSPC121, Ptp, PTPLAD1, RGD1565496
HERPUD1	He, HERP, HERPUD, homocysteine-inducible, endoplasmic reticulum stress-inducible, ubiquitin-like domain member 1, homocysteine inducible ER protein with ubiquitin like domain 1, Mi, Mif1, Mifl, SUP
HMOX1	bK286B10, D8Wsu38, D8Wsu38e, haemox, HEME OXYGENASE, Heme oxygenase 1, HEME OXYGENASE (DECYCLIZING) 1, Hemox, Heox, HEOXG, Hmo, Hmox, HMOX1D, HO-, HO-1, Hsp, HSP32
Hsp22/Hsp40/ Hsp90	HSP 22/40/90
JINK1/2	JNK1/2, JNK p54
Jun	AP-1, JUN, Jun dim
KEAP1	IN, INRF2, Kelch Like ECH-Associated Protein 1, KLHL19, mKIAA0132
Keap1-Nrf2	Keap1-Nfe2l2
Maf	MAF, SMALL MAF
MAF	2810401A20Rik, A230108G15RIK, avian musculoaponeurotic fibrosarcoma oncogene homolog, AW047063, AYGRP, CCA4, c-ma, c-MAF, CTRCT21, Maf2, MAF bZIP transcription factor, V-MAF MUSCULOAPONEUROTIC FIBROSARCOMA ONCOGENE HOMOLOG
MAP2K1/2	MEK1/2, MKK1/2
MAP2K4/7	Jnkk, MEK 4/7, MKK 4/7
MAP2K5	AI324775, AI428457, HsT17454, MAP kinase kinase 5, MAPKK5, MEK5, mitogen-activated protein kinase kinase 5, MKK5, PRKMK5
MAP3K1	LOC100912399, MAPK, MAPKKK1, MEKK, MEKK 1, MEK KINASE, MEK KINASE 1, mitogen-activated protein kinase kinase kinase 1, mitogen-activated protein kinase kinase kinase 1-like, Raf, SRXY6
MAP3K5	7420452D20Rik, A, APOPTOSIS SIGNAL REGULATED KINASE 1, AS, ASK, ASK1, M3K5, MAPKKK5, MEKK5, mitogen-activated protein kinase kinase kinase 5, RGD1306565
MAP3K7	CSCF, FMD2, Map3k7 predicted, MEKK7, mitogen-activated protein kinase kinase kinase 7, TAK1, TGF1a, tgf β activated kinase 1
MAPK14	Crk, CRK1, CSB, CSBP, CSBP1, CSBP2, CSPB1, EXIP, Hog, LOC101929346, MAPK p38, mitogen-activated protein kinase 14, Mxi, Mxi2, p3, p38-, p38a, p38ALPHA, p38Hog, p38 kinase, p38M, p38 MAPK, P38 Map Kinase, p38 MAP kinases alpha and beta, p38 MAP kinases α and β, p38 MAPK-α, p38-α, PRKM14, PRKM15, RK, SAPK2A, SAPK p38 alpha, SAPK p38 α
MAPK7	b2b2346C, b2b2346Clo, BMK-1, ERK, ERK4, ERK-5, Erk5-T, ERK7, FRK, LOC100912585, mitogen-activated protein kinase 7, mitogen-activated protein kinase 7-like, PRKM7
Mkk3/6	MEK3/6, Mkk3/6 (mitogen activated protein kinase kinase 3/6), MKK3/MKK6
NFE2L2	BM974200, HEBP1, IMDDHH, Nr, NRF2, nuclear factor, erythroid 2-like 2, nuclear factor, erythroid derived 2, like 2
NQO	Nadph-d, NADPH QUINONE OXIDOREDUCTASE
PI3K	1-phosphatidylinositol 3-kinase, 2.7.1.137, ATP:1-phosphatidyl-1D-myo-inositol 3-phosphotransferase, Phosphatidylinositol 3 kinase, phosphatidylinositol 3'-kinase, PI3-kinase, PtdIns 3 Kinase, type III phosphoinositide 3-kinase, type I phosphatidylinositol kinase, Vps34p
PKC	Cnpkc, PKC, Pkc(s), Protein Kinase C
PMF1	2600009M07Rik, AL033286, AW060657, LOC686408, PMF-1, PMF1-BGLAP, PMF1-BGLAP readthrough, polyamine-modulated factor 1
PPIB	AA408962, AA553318, AI844835, Cph, Cphn, Cphn-2, CyP-20, CyP-20b, CYPB, CYP-S1, HEL-S-39, OI9, peptidylprolyl isomerase B, SCYLP
PRDX1	ENHANCER, Hbp23, MSP, MSP23, NKEF-A, OSF, OSF-3, P, PAG, PAGA, PAGB, PEROXIREDOXIN 1, peroxiredoxin 1-like 1, PEROXYREDOXIN 1, pr, Prdx1l1, PrdxI, PRX1, PRXI, Tdpx, TDPX2, TDX2, TPxA
PSM	PROTEASOME SUBUNITS
RAF1	6430402F14Rik, AA990557, BB129353, CMD1NN, c-R, Cra, CRAF, Craf1, D830050J10Rik, leukaemiA ONCOGENE HOMOLOG1, LEUKEMIA ONCOGENE HOMOLOG1, NS5, Raf-1 proto-oncogene, serine/threonine kinase, v-, v-Raf, v-raf-leukaemia viral oncogene 1, v-raf-leukemia viral oncogene 1
RBX1	1500002P15Rik, AA517855, BA554C12.1, Gm9840, LOC100046417, Rbx1-ps, ring-box 1, ring-box 1, pseudogene, RNF75, RO, ROC1
SCARB1	AI120173, CD36, Cd36l, CD36L1, Chohd, Chohd1, Cla, CLA-1, D5Ertd460, D5Ertd460e, Hdlq, Hdlq1, HDLQTL6, HDL Receptor, Hlb39, Hlb398, mSR-BI, S, SCAVENGER receptor B1, Scavenger receptor class b1, scavenger receptor class B member 1, scavenger receptor class B, member 1, Sr, SR-B, SR-B1, SR-BI
SLC35A2	AI327289, CDG2M, CDGX, Had-, Had-1, Sfc, Sfc8, solute carrier family 35 member A2, UDP-Gal-Tr, Uga, UGALT, UGAT, UGT, UGT1, UGT2, UGTL
SOD	1.15.1.1, copper-zinc superoxide dismutase, cuprein, Cu,Zn-SOD, Cu-Zn superoxide dismutase, cytocuprein, erythrocuprein, ferrisuperoxide dismutase, Fe-SOD, haemocuprein, hemocuprein, hepatocuprein, Mn-SOD, Mn zinc sod, SOD-1, SOD-2, SOD-3, SOD-4, SODF, SODS, superoxidase dismutase, superoxide dismutase, superoxide dismutase I, superoxide dismutase II, superoxide:superoxide oxidoreductase
SQSTM1	A17, A170, AC008393.2, DMRV, FTDALS3, NADGP, OSF, OSF-6, Osi, OSIL, Osip, Oxidative Stress, p6, p60, p62, p62B, PDB3, Pkc zeta interacting, Pkc ζ interacting, S, sequestosome 1, STAP, STONE14, Ubiquitin-binding protein a, ZIP, ZIP3
STIP1	H, HEL-S-94n, HOP, IEF-SSP-3521, p6, P60, SIP1, ST, STI1, STI1L, Stress-induced phosphoprotein 1
TXN	ADF, AW550880, EOSINOPHIL CYTOTOXICITY FACTOR, thioredoxin, Thioredoxin-1, TRDX, TRX, TRX1, Txn1
TXNRD1	GRIM-12, KM 102 DERIVED REDUCTASE LIKE FACTOR, T, Tgr, Thioredoxin reductase, thioredoxin reductase 1, TR, TR1, Trx, TRXR1, TXNR
UBB	AL033289, HEL-S-50, RPS27A, UBA52, Ubb2, UBC, UBIQUITIN, UBIQUITIN B
UBE2E3	Ubc4-t, Ubce4, UBCH9, UbcM2, ubiquitin-conjugating enzyme E2E 3
UBE2K	AW492011, D5Ertd601, D5Ertd601e, E2-25K, Hi, HIP-2, HYPG, LIG, UBC1, UBIQUITIN CARRIER, ubiquitin-conjugating enzyme E2K
USP14	2610005K12Rik, 2610037B11RIK, a, at, AW107924, ax, C78769, NMF37, nmf375, TGT, ubiquitin specific peptidase 14, Ubiquitin specific protease 14
VCP	3110001E05, CDC48, FTDALS6, IBMPFD, p9, p97, p97/, p97/VCP, TERA, Ter atpase, Transitional Endoplasmic Reticulum Atpase, valosin-containing protein, XSG7

Pathway Analysis Using IPA Software; canonical pathway

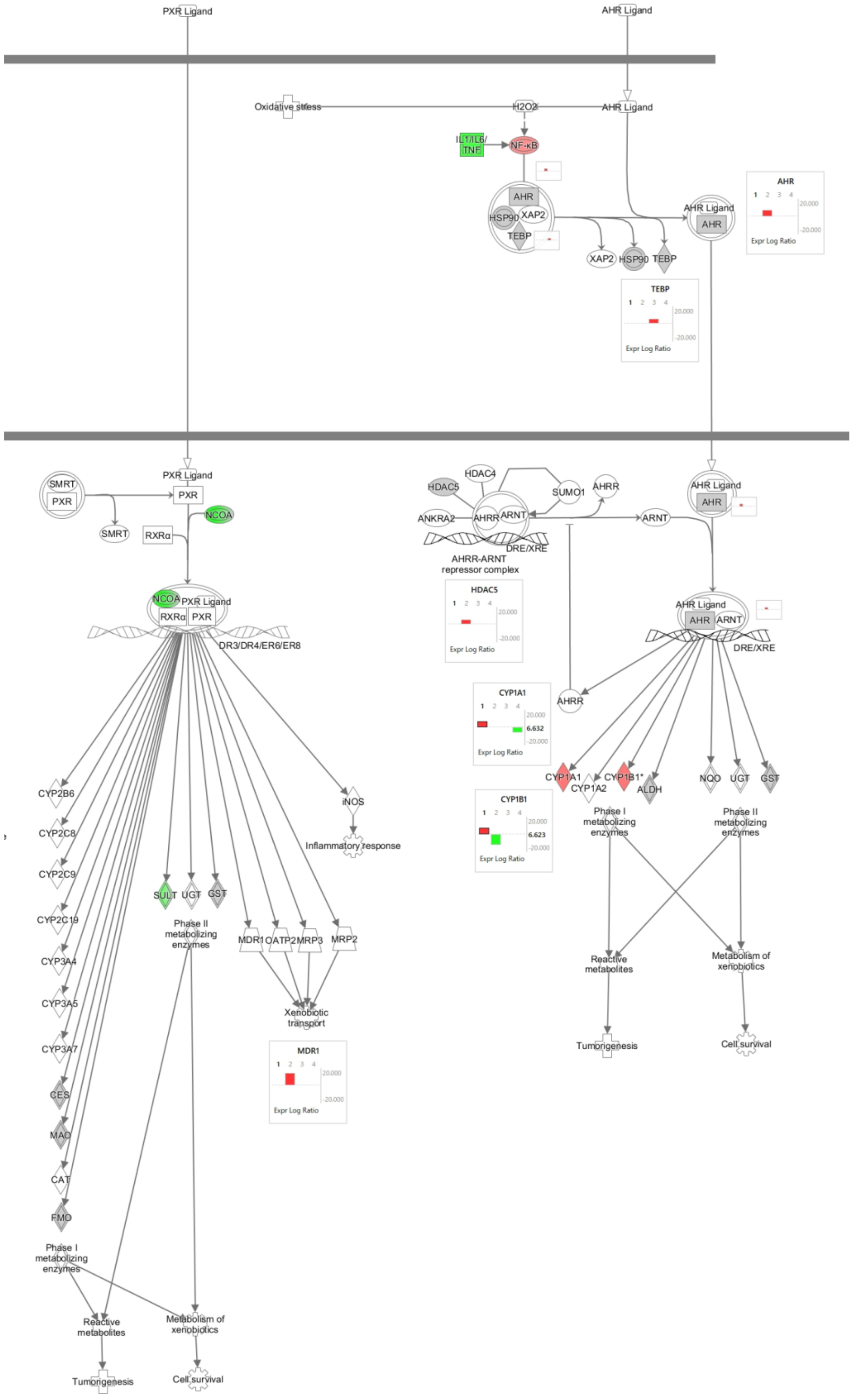

Figure S10. Xenobiotic Metabolism Signaling

1. 1 h; 2. 6 h; 3. 24 h; 4. 8 days.

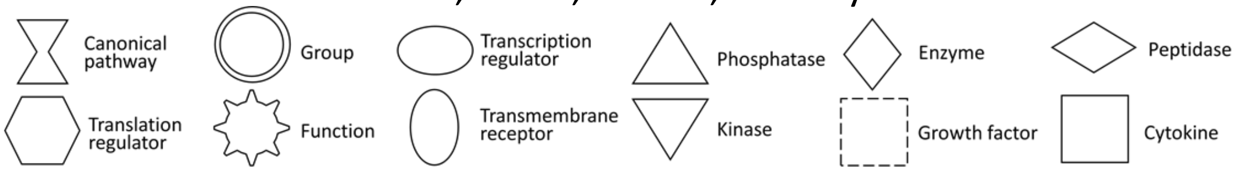

Red: Increased, FDR<0.05 versus solvent control

Green: Decreased, FDR<0.05 versus solvent control

Pathway Analysis Using IPA Software; canonical pathway

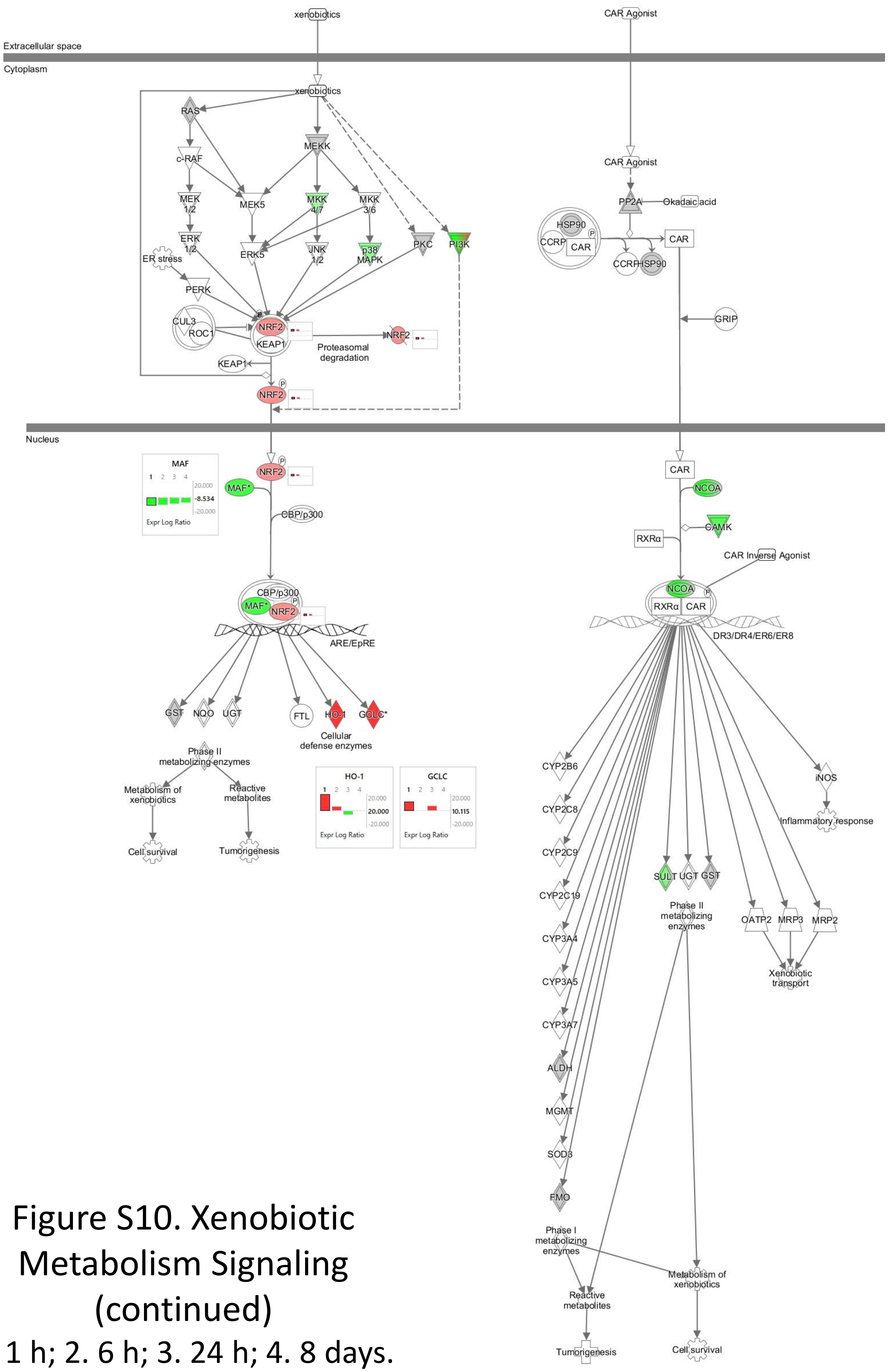

Figure S10. Xenobiotic Metabolism Signaling (continued)

1. 1 h; 2. 6 h; 3. 24 h; 4. 8 days.

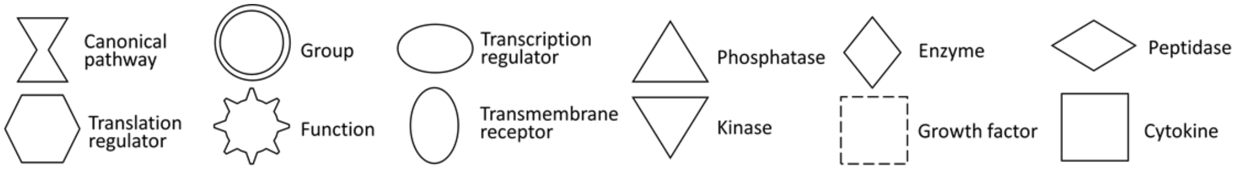

Red: Increased, FDR<0.05 versus solvent control

Green: Decreased, FDR<0.05 versus solvent control

Symbol	Synonym(s)
ABCB1	ABC20, Abcb1a, Abcb4, ATP-binding cassette, sub-family B (MDR/TAP), member 1A, ATP binding cassette subfamily B member 1, ATP binding cassette subfamily B member 1A, CD243, CLCS, Evi, Evi32, GP170, Mdr, MDR1, Mdr1a, Mdr3, P-, p-170, P-GLYCOPROTEIN, P-GP, Pgy-, PGY1, Pgy-3
ABCC2	ABC30, AI173996, Atpase canalicular type, ATP-binding cassette, sub-family C (CFTR/MRP), member 2, ATP binding cassette subfamily C member 2, CANALICULAR MULTIDRUG RESISTANCE, Cmo, CMOAT, cMRP, DJS, Ebcr, Mr, mrp, MRP2
ABCC3	1700019L09Rik, ABC31, ATP-binding cassette C3, ATP-binding cassette, sub-family C (CFTR/MRP), member 3, ATP binding cassette subfamily C member 3, cMOAT2, EST90757, MLP2, MOAT-D, MR, MRP3, Multidrug Resistant Protein 3
AHR	A, Ah, Ahh, Ahre, AH receptor, aryl-hydrocarbon receptor, bHLHe7, bHLHe76, DIOXIN receptor, In, RP85
AHR Ligand	AHR ligand, aromatic hydrocarbon
AHRR	AHH, AHHR, aryl-hydrocarbon receptor repressor, bHLHe77, mKIAA1234
AIP	A, AA408703, ARA9, aryl-hydrocarbon receptor-interacting protein, AW476050, D19Bwg1412e, Fkbp1, FKBP16, FKBP37, PITA1, SMTPHN, Xa, XAP-2
ALDH	ALDEHYDE DEHYDROGENASE
ANKRA2	1110004M18Rik, ANKRA, ankyrin repeat family A member 2, ankyrin repeat, family A (RFXANK-like), 2
ARNT	Arnt1, aryl hydrocarbon receptor nuclear translocator, bHLHe, bHLHe2, D3Ertd557, D3Ertd557e, DIOXIN receptor, Drnt, ESTM4, ESTM42, Hif1, HIF1B, HIF1BETA, HIF-1-β, HIF beta, HIF β, mKIAA4051, TANGO, W08714
CAMK	Ca2+/calmodulin-dependent protein kinase, Calmodulin Kinase
CAR Agonist	steroids
CAT	2210418N07, ACATALASIA, Ca, Cas, Cas-1, Cat01, Catalase, Catalase1, Catl, Cs-, CS1
CBP/p300	CBP, CBP-p300
CES	3.1.1.1, ali-esterase, alpha-carboxylesterase, B-esterase, BUTYRATE ESTERASE, butyryl esterase, Carboxyesterase, carboxylate esterase, CARBOXYLESTERASE, carboxylic acid esterase, carboxylic esterase, CE, cocaine esterase, esterase A, esterase B, esterase D, methylbutyrase, methylbutyrate esterase, monobutyrase, nonspecific carboxylesterase, NSE, procaine esterase, propionyl esterase, serine esterase, triacetin esterase, vitamin A esterase, α-carboxylesterase
CUL3	cullin 3, KIAA0617, PHA2E
Cul3-Roc1	Cul3-Rbx1
CYP1A1	AHH, AH Hydroxylase, AHRR, CP11, CYP1, Cyp45c, Cypc45c, CYPIA1, cytochrome P450 family 1 subfamily A member 1, cytochrome P450, family 1, subfamily a, polypeptide 1, EROD, P1-450, P450-, P450-1, P-450bnf-b, P450-C, P450DX, P450 IA1, P-450MC, P450-P1
CYP1A2	CP1, CP12, CYPD45, CYPIA2, cytochrome P450 family 1 subfamily A member 2, cytochrome P450, family 1, subfamily a, polypeptide 2, P3-450, P450-, P450-3, P-450d, P450 IA2, P-450isf-g, P450-P3, P450(PA), RATCYPD45
CYP1B1	ASGD6, CP1B, CYPIB1, cytochrome P450 family 1 subfamily B member 1, cytochrome P450, family 1, subfamily b, polypeptide 1, GLC3A, P4501B1, P450Rap
CYP2B6	CPB6, Cyp2, CYP2B, CYP2B1, Cyp2b10, CYP2B2, Cyp2b20, Cyp2b20/10, CYP2B3, Cyp2b6/7, CYP2B7, CYP2B7P, Cyp450e, Cypbe, Cype, CYPIIB2, CYPIIB3, CYPIIB6, Cytochrome P450, 2b19, cytochrome P450 family 2 subfamily B member 6, cytochrome P450, family 2, subfamily b, polypeptide 1, cytochrome P450, family 2, subfamily b, polypeptide 10, cytochrome P450, family 2, subfamily b, polypeptide 2, EFVM, IIB1, LOC361523, p1, p16, P450, P450iib1
CYP2C19	CPCJ, CYP2C, Cyp2c6, Cyp2c6v1, CYP450-2C, CYPIIC17, CYPIIC19, CYPIIC6, cytochrome P450 family 2 subfamily C member 19, cytochrome P450, family 2, subfamily C, polypeptide 6, variant 1, P450-11A, P450C2C, P450IIC19, PB1, PTF2
CYP2C8	AH, Ah-2, Ahh-, Ahh-1, AHO, AHOH, AHOHase, AI159681, AI196010, CPC8, Cyp2, Cyp2c, Cyp2c29, Cyp2c38, Cyp2c39, Cyp2c7, CYP2C8DM, Cyp2c9, CYPIIC39, CYPIIC8, Cytochrome p450, cytochrome P450 family 2 subfamily C member 8, cytochrome P450, family 2, subfamily c, polypeptide 29, cytochrome P450, family 2, subfamily c, polypeptide 38, cytochrome P450, family 2, subfamily c, polypeptide 39, cytochrome P450, family 2, subfamily c, polypeptide 7, LOC100361347, LOC100911552, LOC639008, MP-12/MP-20, P450-2, P450-2C
CYP2C9	2010301M18Rik, 2210009K14Rik, CPC9, CYP2C, CYP2C10, Cyp2c11, Cyp2c11l, Cyp2c4, Cyp2c65, Cyp2c66, CYP2CII, CYPIIC9, cytochrome P450 family 2 subfamily C member 9, cytochrome P450, family 2, subfamily c, polypeptide 65, cytochrome P450, family 2, subfamily c, polypeptide 66, cytochrome P450, subfamily 2, polypeptide 11, Hexobarbital hydroxylase, LOC100911826, p450 2c29, P4502C9, P450IIC9, Progesterone 2alpha hydroxylase, RGD: 632285
CYP3A4	CP33, CP34, CYP3A, Cyp3a11, Cyp3a2, CYP3A23, CYP3A3, CYPIIIA3, CYPIIIA4, CYTOCHROME P450 3A3, cytochrome P450 family 3 subfamily A member 4, cytochrome P450, family 3, subfamily a, polypeptide 2, HLP, NF-25, P450C3, P450PCN1, P-450ut-a, VDDR3
CYP3A5	AI256190, cDEX, CP35, CYP, Cyp3a, Cyp3a1, Cyp3a11, Cyp3a16, Cyp3a2, Cyp3a23, Cyp3a23-3a1, Cyp3a3, Cyp3a4, Cyp3a41, Cyp3a41a, Cyp3a41b, Cyp3a44, CYPIIIA5, cytochrome P450 3A1-like, cytochrome P450 family 3 subfamily A member 5, cytochrome P450, family 3, subfamily a, polypeptide 11, cytochrome P450, family 3, subfamily a, polypeptide 16, cytochrome P450, family 3, subfamily a, polypeptide 23-polypeptide 1, cytochrome P450, family 3, subfamily a, polypeptide 41A, cytochrome P450, family 3, subfamily a, polypeptide 41B, cytochrome P450, family 3, subfamily a, polypeptide 44, II, IIIAm1, LOC100910877, P, P450PCN3, Pcn, PCN3, RL33
CYP3A7	CP37, Cyp3a13, CYP3A6, Cyp3a9, CYPIIIA7, Cytochrome p450 3a9, cytochrome P450 family 3 subfamily A member 7, cytochrome P450, family 3, subfamily a, polypeptide 13, cytochrome P450, family 3, subfamily a, polypeptide 9, II, IIIAm2, P-450111A7, P-450(HFL33), P450-HFLA, P450HLp2, P450olf3
DNAJC7	2010003F24Rik, 2010004G07Rik, CCRP, DJ11, DJC7, DnaJ heat shock protein family (Hsp40) member C7, mDj11, mTp, mTpr2, TPR2, TTC2
EIF2AK3	eukaryotic translation initiation factor 2 alpha kinase 3, eukaryotic translation initiation factor 2 α kinase 3, PE, PEK, PERK, WRS
ERK1/2	MAPK p44/42, MAPK p44/p42, p42/44 mapk, P42/p44 erk, P42/p44 mapk, p42/p44 MAP KINASE
FMO	1.14.13.8, dimethylaniline monooxygenase (N-oxide-forming), dimethylaniline N-oxidase, dimethylaniline oxidase, DMA oxidase, FAD-containing monooxygenase, FAD monooxygenase, flavin-containing monooxygenase, flavin monooxygenase, FMO-I, FMO-II, mixed-function amine oxidase, N,N-dimethylaniline monooxygenase, N,N-dimethylaniline,NADPH2:oxygen oxidoreductase (N-oxide-forming)
FTL	Ferritin, FERRITIN LIGHT CHAIN, ferritin light chain 1, ferritin light chain 1-like, ferritin light chain 1-like 1, ferritin light chain 2, ferritin light polypeptide 1, ferritin light polypeptide, pseudogene 2, FL, FRIL, Fril1, Ft, FTL1, Ftl1l1, Ftl1-ps2, Ftl-ps, Gm20746, L-fe, L-ferritin, LFTD, LOC100359668, LOC100360087, LOC100362384, LOC100363177, NBIA3, RGD1560687, RGD1561055, RGD1566189, similar to ferritin light chain, similar to Ferritin light chain (Ferritin L subunit), YB24D08
GCLC	D9Wsu168, D9Wsu168e, gamma GCS HEAVY CHAIN, Gamma Glutamylcysteine Synthetase, Gamma glutamylcysteine synthetase heavy subunit, Gamma Glutamyl Cysteine Synthetase Light Subunit, GCL, GCS, GCS, Catalytic, GCS-HS, Ggcs-hs, Glc, GLCL, GLCLC, GLCL-H, glutamate-cysteine ligase catalytic subunit, Glutamate-Cysteine Ligase, Catalytic Subunit, γ Gcs, γ-Gcsh, γ GCS HEAVY CHAIN, γ Glutamylcysteine Synthetase, γ glutamylcysteine synthetase heavy subunit, γ Glutamyl Cysteine Synthetase Light Subunit
GRIP1	4931400F03Rik, eb, FRASRS3, glutamate receptor interacting protein 1, GRIP
GST	Glutathione s-transferase, GSH Transferase
H2O2	7722-84-1, A-101, Colgate Peroxyl, Eskata, H2O2, hydrogen dioxide, hydrogen peroxide, peroxyl mouthwash, urea hydrogen peroxide
HDAC4	4932408F19, 4932408F19Rik, AHO3, BDMR, HA6116, HD4, HDAC-A, histone deacetylase 4
HDAC5	AI426555, HD5, Hdac4, histone deacetylase 5, mHDA, mHDA1, mKIAA0600, NY-CO-9
HMOX1	bK286B10, D8Wsu38, D8Wsu38e, haemox, HEME OXYGENASE, Heme oxygenase 1, HEME OXYGENASE (DECYCLIZING) 1, Hemox, Heox, HEOXG, Hmo, Hmox, HMOX1D, HO-, HO-1, Hsp, HSP32
HSP90	HSC90, Hsp84
JINK1/2	JNK1/2, JNK p54
KEAP1	IN, INRF2, Kelch Like ECH-Associated Protein 1, KLHL19, mKIAA0132
Keap1-Nrf2	Keap1-Nfe2l2
MAF	2810401A20Rik, A230108G15RIK, avian musculoaponeurotic fibrosarcoma oncogene homolog, AW047063, AYGRP, CCA4, c-ma, c-MAF, CTRCT21, Maf2, MAF bZIP transcription factor, V-MAF MUSCULOAPONEUROTIC FIBROSARCOMA ONCOGENE HOMOLOG
MAO	1.4.3.4, adrenaline oxidase, adrenalin oxidase, amine oxidase, amine oxidase (flavin-containing), amine:oxygen oxidoreductase (deaminating) (flavin-containing), epinephrine oxidase, monoamine:O2 oxidoreductase (deaminating), Monoamine oxidase, polyamine oxidase, serotonin deaminase, spermidine oxidase, spermine oxidase, tyraminase, tyramine oxidase
MAP2K1/2	MEK1/2, MKK1/2
MAP2K4/7	Jnkk, MEK 4/7, MKK 4/7
MAP2K5	AI324775, AI428457, HsT17454, MAP kinase kinase 5, MAPKK5, MEK5, mitogen-activated protein kinase kinase 5, MKK5, PRKMK5
Map3k	MapKKK, MEKK, MEKKs, Mkkk
MAPK7	b2b2346C, b2b2346Clo, BMK-1, ERK, ERK4, ERK-5, Erk5-T, ERK7, FRK, LOC100912585, mitogen-activated protein kinase 7, mitogen-activated protein kinase 7-like, PRKM7
MGMT	AG, AGAT, AGT, AI267024, O6-ALKYLGUANINE DNA ALKYLTRANSFERASE, O-6-methylguanine-DNA methyltransferase
Mkk3/6	MEK3/6, Mkk3/6 (mitogen activated protein kinase kinase 3/6), MKK3/MKK6
NFE2L2	BM974200, HEBP1, IMDDHH, Nr, NRF2, nuclear factor, erythroid 2-like 2, nuclear factor, erythroid derived 2, like 2
NFkB	NF-KAPPA B, NF-κ B, nuclear factor-κ b, transcription factor nuclear factor κ b
NOS2	CALCIUM-INDEPENDENT NOS, Hepatocyte NOS, HEP-NOS, Inducible NOS, INOS, LOC497963, MAC-NOS, N, nitric oxide synthase 2, nitric oxide synthase 2, inducible, No, NOS, NOS2A, NOS-II, similar to Nitric oxide synthase, inducible (NOS type II) (Inducible NO synthase) (Inducible NOS) (iNOS)
NQO	Nadph-d, NADPH QUINONE OXIDOREDUCTASE
NR1I2	BXR, mP, mPXR, nuclear receptor subfamily 1 group I member 2, nuclear receptor subfamily 1, group I, member 2, ONR1, P, PAR, PAR1, PAR2, PARq, PRR, PXR, PXR1, PXR.1, PXR.2, S, SAR, SXR
NR1I3	AA209988, AI551208, C, CA, CAR, CAR1, CAR2, CAR-beta, Care2, CAR-β, Constitutive androstane receptor, ESTM3, ESTM32, MB67, mC, nuclear receptor subfamily 1 group I member 3, nuclear receptor subfamily 1, group I, member 3
Okadaic acid	(2R)-3-[(2S,6R,8S,11R)-2-[(E,2R)-4-[(2S,2'R,4R,6R,8aR)-4-hydroxy-2-[(1S,3S)-1-hydroxy-3-[(3R,6S)-3-methyl-1,7-dioxaspiro[5.5]undecan-2-yl]butyl]-3-methylidenespiro[4a,7,8,8a-tetrahydro-4H-pyrano[3,2-b]pyran-6,5'-oxolane]-2'-yl]but-3-en-2-yl]-11-hydroxy-4-methyl-1,7-dioxaspiro[5.5]undec-4-en-8-yl]-2-hydroxy-2-methylpropanoic acid, 78111-17-8, acanthifolicin, 9,10-deepithio-9,10-didehydro-, C44H68O13, OKA, okdanoic acid
p38MAPK	P38, p38 MAP KINASE, P38 MITOGEN-ACTIVATED protein KINASE
Phase2 detoxifyingenzyme	phase 2 detoxifying enzyme, phase II drug metabolizing enzyme, phase II metabolizing enzyme, phase II metabolizing enzymes
phaseI detoxifyingenzyme	phase I drug metabolizing enzyme, phase I metabolizing enzyme, phase I metabolizing enzymes
PI3K	1-phosphatidylinositol 3-kinase, 2.7.1.137, ATP:1-phosphatidyl-1D-myo-inositol 3-phosphotransferase, Phosphatidylinositol 3 kinase, phosphatidylinositol 3'-kinase, PI3-kinase, PtdIns 3 Kinase, type III phosphoinositide 3-kinase, type I phosphatidylinositol kinase, Vps34p
PKC	Cnpkc, PKC, Pkc(s), Protein Kinase C
PP2A	protein PHOSPHATASE 2A, Protein Phosphatase Type2a
PTGES3	5730442A20Rik, cPG, cPGES, Gm9769, p23, p23 COCHAPERONE, p23 PR RELATED, PGES3, prostaglandin E synthase 3, prostaglandin E synthase 3, pseudogene, Ptg, Ptges, Ptges3-ps, RGD1561913, sid31, sid3177, Teb, TEBP, Telomerase Binding Protein p23, Zhf6
PXR Ligand	17-ethyl-10,13-dimethyl-2,3,4,5,6,7,8,9,11,12,14,15,16,17-tetradecahydro-1H-cyclopenta[a]phenanthrene, 26856-62-2, C21H36, pregnanes
RAF1	6430402F14Rik, AA990557, BB129353, CMD1NN, c-R, Cra, CRAF, Craf1, D830050J10Rik, leukaemiA ONCOGENE HOMOLOG1, LEUKEMIA ONCOGENE HOMOLOG1, NS5, Raf-1 proto-oncogene, serine/threonine kinase, v-, v-Raf, v-raf-leukaemia viral oncogene 1, v-raf-leukemia viral oncogene 1
RBX1	1500002P15Rik, AA517855, BA554C12.1, Gm9840, LOC100046417, Rbx1-ps, ring-box 1, ring-box 1, pseudogene, RNF75, RO, ROC1
RXRA	9530071D11RIK, LOC101928454, NR2B1, retinoid 10 receptor α, Retinoid X receptor alpha, Retinoid X receptor α, RXR, RXR-alpha, RXRalpha1, RXR-α, Rxr α 1
SLCO1B1	HBLRR, LST-1, OATP1B1, OATP2, OATP-C, SLC21A5, SLC21A6, solute carrier organic anion transporter family member 1B1
SMRTalpha	CTG26, N-CoR, nuclear receptor co-repressor 2, RETINOID SILENCER, SM, SMAP270, SMR, SMRT, SMRTE, SMRTE-tau, TNRC14, TRAC, TRAC-1
SOD3	AI314465, EC-S, EC-SOD, ECSODPT, superoxide dismutase 3, superoxide dismutase 3, extracellular
Sulfotransferase	2.8.2.-, CHST1, CHST2, CHST4, CHST6, sulphotransferase, SULT, UST
SUMO1	DAP1, GMP1, OFC10, PI, PIC1, SE, SENP2, SENTRIN, small ubiquitin-like modifier 1, SMT3, SMT3C, SMT3H3, SMTP3, Ubl, UBL1
UGT	Glu-t, UDP-glucuronosyltransferase, UDP GLUCURONYLTRANSFERASE, UDP-GT

Pathway Analysis Using IPA Software; canonical pathway

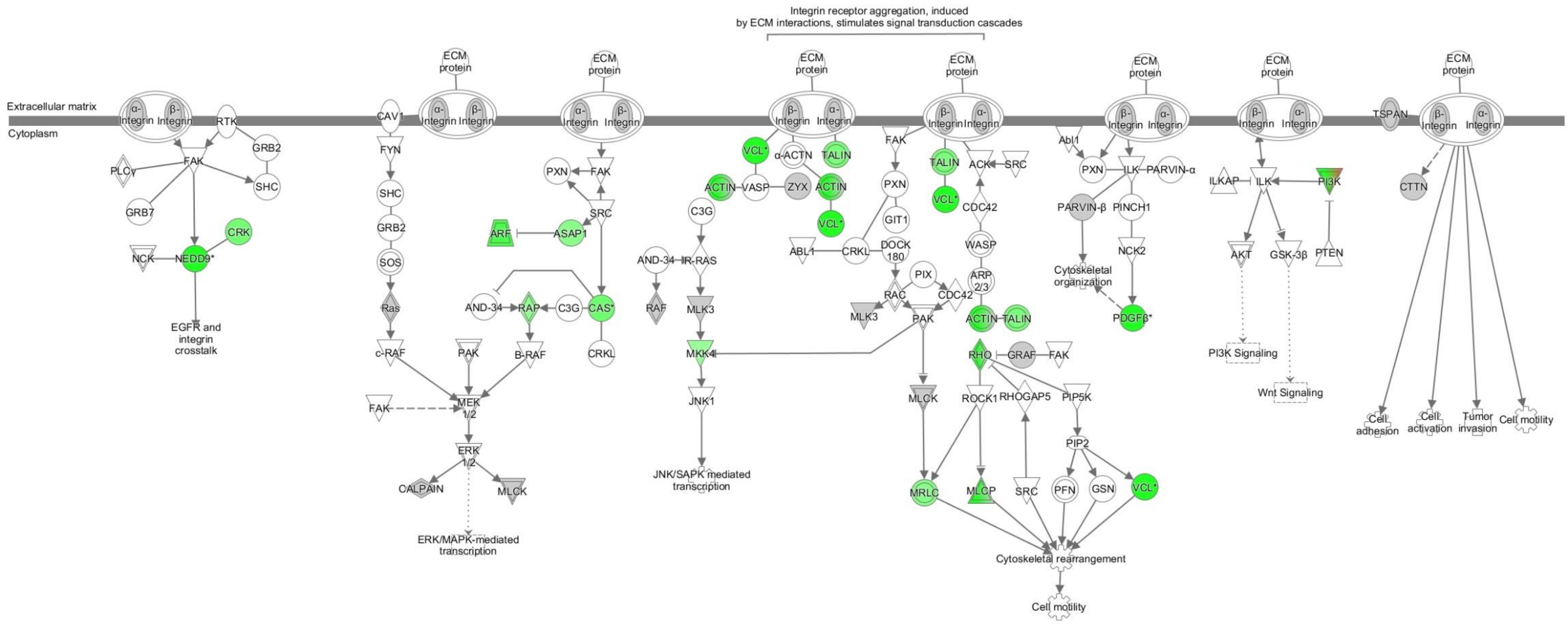

Figure S11. Integrin Signaling at 1 h

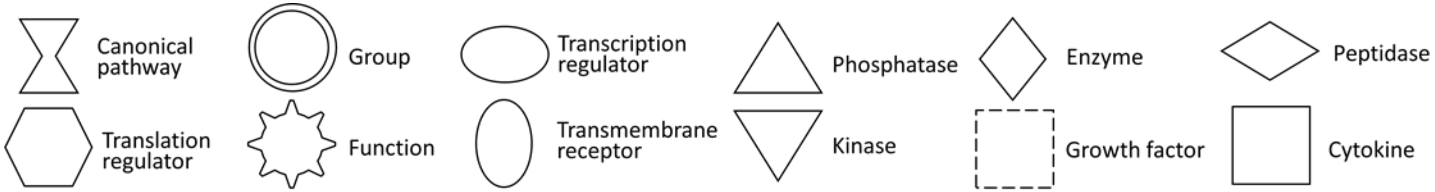

Red: Increased, FDR<0.05 versus control

Green: Decreased, FDR<0.05 versus control

Symbol	Synonym(s)
ABL1	ABL, ABL proto-oncogene 1, non-receptor tyrosine kinase, AI325092, BCR-ABL, c-A, c-ABL, CABL1, c-abl oncogene 1, non-receptor tyrosine kinase, CHDSKM, E430008G22Rik, JTK7, LOC100909750, p145Abl, p150, tyrosine-protein kinase ABL1-like, v-abl
ACTIN	CLEC9A Ligand, G-actin
AKT	AKT1/2/3, B/Akt, PKB, RAC-PK
Alphaactinin	ACTININ, Actinin alpha, Actinin α, ACTN, α-Actinin, α Actinin human
Alphaintegrin	Adhesion Receptors, alpha-Integrin, CD11, Cd11b/c, Integrin alpha, α- Integrin
ARHGAP26	1810044B20RIK, 2610010G17RIK, 4933432P15RIK, AI853435, GRAF, GRAF1, GTPASE REGULATOR ASSOCIATED with FOCAL ADHESION KINASE PP125(FAK), mKIAA0621, OLIGOPHRENIN-1 LIKE, OPHN1L, OPHN1L1, Rho GTPase activating protein 26
ARHGAP5	AU014947, GFI2, LRRGT00098, p190-, p190-B, p190BRhoGAP, p190Rhogap, p190RhoGAP-B, RhoGAP5, Rho GTPase Activating Protein 5
ARHGEF7	beta1PIX, betaPi, BETA-PIX, betaPix-b, betaPix-c, Beta-Pix Cool, C, coo, Cool, COOL-1, mKIAA0142, Nbla10314, P, P50, P50BP, p8, P85, P85 beta pix, P85COOL1, P85SPR, P85 β pix, PAK3, Pak3bp, PAK-INTERACTING EXCHANGE FACTOR beta, PAK-INTERACTING EXCHANGE FACTOR β, PIX, PIXB, Rho guanine nucleotide exchange factor 7, Rho guanine nucleotide exchange factor (GEF7), β-PIX, β Pix-a, β-Pix Cool
Arp2-3	Arp, Arp2-3, ARP2-3 (Actin-related protein complex), Arp Complex
ASAP1	AMAP1, ArfGAP with SH3 domain, ankyrin repeat and PH domain 1, AV239055, CENTB4, DDEF1, DEF-1, LOC100039024, mKIAA1249, PAG2, PAP, s19, ZG14P
BCAR1	AI385681, BCAR1 scaffold protein, Cas family member, breast cancer anti-estrogen resistance 1, C, CAS, CAS1, CASS1, Cr, CRKAS, LOC100131601, p130, P130CAP, P130CAS
BCAR3	AI131758, AND-, AND-34, BCAR3 adaptor protein, NSP family member, breast cancer anti-estrogen resistance 3, LOC101928013, MIG7, NSP2, RP11 488P31, SH2D3B
Betaintegrin	beta-Integrin, Integrin beta, β- Integrin
BRAF	9930012E13RIK, AA120551, AA387315, AA473386, AI447469, Bra, B-RAF1, Braf-2, B-Raf proto-oncogene, serine/threonine kinase, Braf transforming gene, C230098H17, C87398, D6Ertd631, D6Ertd631e, NS7, RAFB, RAFB1
CALPAIN	CALCIUM DEPENDENT PROTEASE, M calpain
CAV1	BSCL3, Cav, cave, Cavelolin 1, CAVEOLIN, Caveolin1, caveolin 1, caveolae protein, CGL3, LCCNS, LOC100362870, MSTP085, PPH3, VIP21
CDC42	AI747189, AU018915, CDC42Hs, cell division cycle 42, CELLULAR GROWTH REGULATING, G25K, TKS
CRK	c-Crk, c-Crk2, Cr, CRK2, Crko, CRK proto-oncogene, adaptor protein, FLJ11558, p38, v-crk avian sarcoma virus CT10 oncogene homolog
CRKL	1110025F07Rik, AA589403, AI325100, Cr, crk-like protein-like, CRK like proto-oncogene, adaptor protein, Crkol, LOC100911248, mgc94609, snoop, v-crk avian sarcoma virus CT10 oncogene homolog-like
CTTN	1110020L01Rik, amplaxin, Cortactin, Cttnb, Ems, EMS1
DOCK1	9130006G06Rik, AI854900, b2b3190C, b2b3190Clo, ced5, D630004B07Rik, dedicator of cyto-kinesis 1, Dock18, DOCK180, LOC679295, RGD1566072
ERK1/2	MAPK p44/42, MAPK p44/p42, p42/44 mapk, P42/p44 erk, P42/p44 mapk, p42/p44 MAP KINASE
FYN	AI448320, AW552119, C-FYN, Fyn proto-oncogene, FYN proto-oncogene, Src family tyrosine kinase, FYNT, LOC102724705, p59-FYN, p59 Fyn B, SLK, SRC-LIKE KINASE, SYN
GIT1	Cat-, Cat-1, GIT ArfGAP 1, p95C, p95Cat
GRB2	AA408164, ASH, Ash-psi, EGFRBP-GRB2, GRAB2, GRBS, growth factor receptor bound protein 2, MST084, MSTP084, NCKAP2
GRB7	growth factor receptor bound protein 7, mKIAA4028
GSK3B	7330414F15Rik, 8430431H08Rik, C86142, glycogen synthase kinase 3 beta, glycogen synthase kinase 3 β, GSK-, GSK-3, GSK-3be, GSK-3beta, GSK-3β, GSKbeta, GSKβ, Tpk1
GSN	ADF, AGEL, Gelsolin, Gelsolin plasma isoform, LOC105376337
ILK	AA511515, ESTM2, ESTM24, HEL-S-28, ILK-1, ILK-2, integrin-linked kinase, P59, p59ILK
ILKAP	0710007A14RIK, 1600009O09Rik, AF095927, AK055417, ILKAP2, ILKAP3, ILK associated serine/threonine phosphatase, integrin-linked kinase-associated serine/threonine phosphatase 2C, PP2C-D, PP2C-DELTA, PP2C-δ, PPM1O
Integrin	Integrin alpha-beta, integrin-extracellular matrix, INTEGRIN receptor, Integrin α-β
LIMS1	2310016J22Rik, 4921524A02Rik, AI507642, AU021743, AW551584, C430041B13RIK, Li, LIM and senescent cell antigen-like domains 1, Lims1l, LIM zinc finger domain containing 1, PIN, PINCH, PINCH-1, RGD1560732
MAP2K1/2	MEK1/2, MKK1/2
MAP2K4	JNKK, JNKK1, MAPK/ERK KINASE-1, MAPKK4, MEK4, mitogen-activated protein kinase kinase 4, MKK4, PRKMK4, SAPKK-1, Sek, SEK1, Ser, SERK1, SKK1
MAP3K11	2610017K16RIK, MEKK11, mitogen-activated protein kinase kinase kinase 11, Mlk, MLK-3, PTK1, RHOE, SPRK
MAPK8	AI849689, C-JUN N-TERMINAL KINASE1, JNK, JNK1, JNK1A2, JNK21B1/2, JNK-46, mitogen-activated protein kinase 8, p46JNK1, p46JNK1 alpha, p46JNK1 α, Prk, PRKM8, SAPK1, SAPK1c, Sapk gamma, SAPK P46, Sapk γ, STRESS-ACTIVATED protein KINASE-LIKE KINASE
MLCP	3.1.3.53, Myosin-bound phosphatase, myosin light chain kinase phosphatase, myosin-light-chain-phosphatase, [myosin-light-chain]-phosphate phosphohydrolase, Myosin Phosphatase, Myosin PPTase, MYPT, protein phosphatase 2A
MRLC	Myosin subunit regulatory light chain, Rlc
Mylk	MLCK, Mylk
NCK	NCK alpha,beta, NCK α,β
NCK2	4833426I10RIK, Grb, GRB4, LOC100503894, NCK adaptor protein 2, NCKbe, NCKbeta, Nck β, non-catalytic region of tyrosine kinase adaptor protein 2
NEDD9	C, Ca, CAS2, CAS-L, CASS2, enhancer of filamentation 1, HEF1, MEF1, neural precursor cell expressed, developmentally down-regulated 9, neural precursor cell expressed, developmentally down-regulated gene 9, p105, P105hef1
PARVA	2010012A22Rik, 5430400F08Rik, act, Actopaxin, Actp, AI225929, alpha PARVIN, AU042898, CH-IL, CH-ILKBP, MXRA2, Parvin, Parvin-alpha, parvin, alpha, Parvin-α, parvin, α, α PARVIN
PARVB	aff, affixin, AI595373, AW742462, CGI-56, D15Gsk, D15Gsk1, Parvin-beta, parvin, beta, Parvin-β, parvin, β
PDGFB	c-sis, IBGC5, PDGF-, PDGF-2, PDGF-BB, PDGF beta, PDGFbetaR, Pdgfrb, PDGFRbeta, PDGF-β, platelet derived growth factor, B polypeptide, platelet derived growth factor subunit B, SIS, SSV
PI3K	1-phosphatidylinositol 3-kinase, 2.7.1.137, ATP:1-phosphatidyl-1D-myo-inositol 3-phosphotransferase, Phosphatidylinositol 3 kinase, phosphatidylinositol 3'-kinase, PI3-kinase, PtdIns 3 Kinase, type III phosphoinositide 3-kinase, type I phosphatidylinositol kinase, Vps34p
PIKFYVE	5230400C17RIK, CFD, FAB1, HEL37, KIAA0981, P, p235, phosphoinositide kinase, FYVE-type zinc finger containing, PI5K, Pip, PIP5K, PIP5K3, Pipk5k3, PipkIII, Type III PI 5-kinase, ZFYVE29
PIP2	C11H19O19P3R2, phosphatidylinositol-4,5-bisphosphate, phosphatidyl-myo-inositol 4,5-bisphosphate, PI(4,5)P2, PI4,5P2, PIP2, PtdIns(4,5)P2
PLC-gamma	Phospholipase C gamma, Phospholipase C γ, PLCG, PLCγ
Profilin	PFN
PTEN	10q23del, 2310035O07RIK, A130070J02Rik, AI463227, B430203M17RIK, BZS, CWS1, DEC, GLM2, MHAM, MMAC, MMAC1, MUTATED IN MULTIPLE ADVANCED CANCERS, mutated in multiple advanced cancers 1, phosphatase and tensin homolog, PTEN1, PTENbeta, TEP, TEP1
PTK2	FA, Fad, FADK, FADK 1, FAK, FAK1, FAK related non-kinase, FR, p125FAK, pp125FAK, PPP1R71, protein tyrosine kinase 2, PTK2 protein tyrosine kinase 2, TYROSINE KINASE 2
PXN	AW108311, AW123232, FLJ23042, P, PAX, PAXILLIN
RAF1	6430402F14Rik, AA990557, BB129353, CMD1NN, c-R, Cra, CRAF, Craf1, D830050J10Rik, leukaemiA ONCOGENE HOMOLOG1, LEUKEMIA ONCOGENE HOMOLOG1, NS5, Raf-1 proto-oncogene, serine/threonine kinase, v-, v-Raf, v-raf-leukaemia viral oncogene 1, v-raf-leukemia viral oncogene 1
Ral	Ral A/B
RAPGEF1	4932418O06Rik, C3G, C3G-1, C3G-2, Grf, GRF2, Rap guanine nucleotide exchange factor 1, Rap guanine nucleotide exchange factor (GEF) 1
RHO	GTPase Rho, Rho, Rho Family, RHO-GTPASE, Rho-like Gtpase
ROCK1	1110055K06Rik, LOC100129157, P160ROCK, p160 ROCK-1, Rho-associated coiled-coil containing protein kinase 1, Roc, ROCK, ROCK-I, ROK, ROK beta, ROK β
RRAS	AI573426, p23, R, RAS related, related RAS viral (r-ras) oncogene, Rras1, Rras predicted
SHC1	p52SHC, p6, p66, p66s, P66shc, Sh, SHC, Shc (46 kDa isoform), SHCA, SHC adaptor protein 1, Shc p66 isoform, src homology 2 domain-containing transforming protein C1
SRC	ASV, AW259666, BS27, c-SRC, p60-Src, PP60, pp60c, Pp60/c-Src, pp60c-src, Rous sarcoma oncogene, SRC1, SRC proto-oncogene, non-receptor tyrosine kinase, THC6, TVHUSC
TALIN	TLN
TNK2	Ac, ACK, ACK1, Cdgip, LOC682784, p21cdc42Hs, Pyk, Pyk1, tyrosine kinase non receptor 2, tyrosine kinase, non-receptor, 2
TSPAN	TETRASPAN, TRANSMEMBRANE 4 SUPERFAMILY
VASP	vasodilator-stimulated phosphoprotein
VCL	9430097D22, AA571387, AI462105, AW545629, CMD1W, CMH15, HEL114, MV, MVCL, Vcl predicted, Vinculin
ZYX	9530098H06Rik, ESP-2, HED-2, R7515, R75157, ZIXIN, Zyxin

Pathway Analysis Using IPA Software; canonical pathway

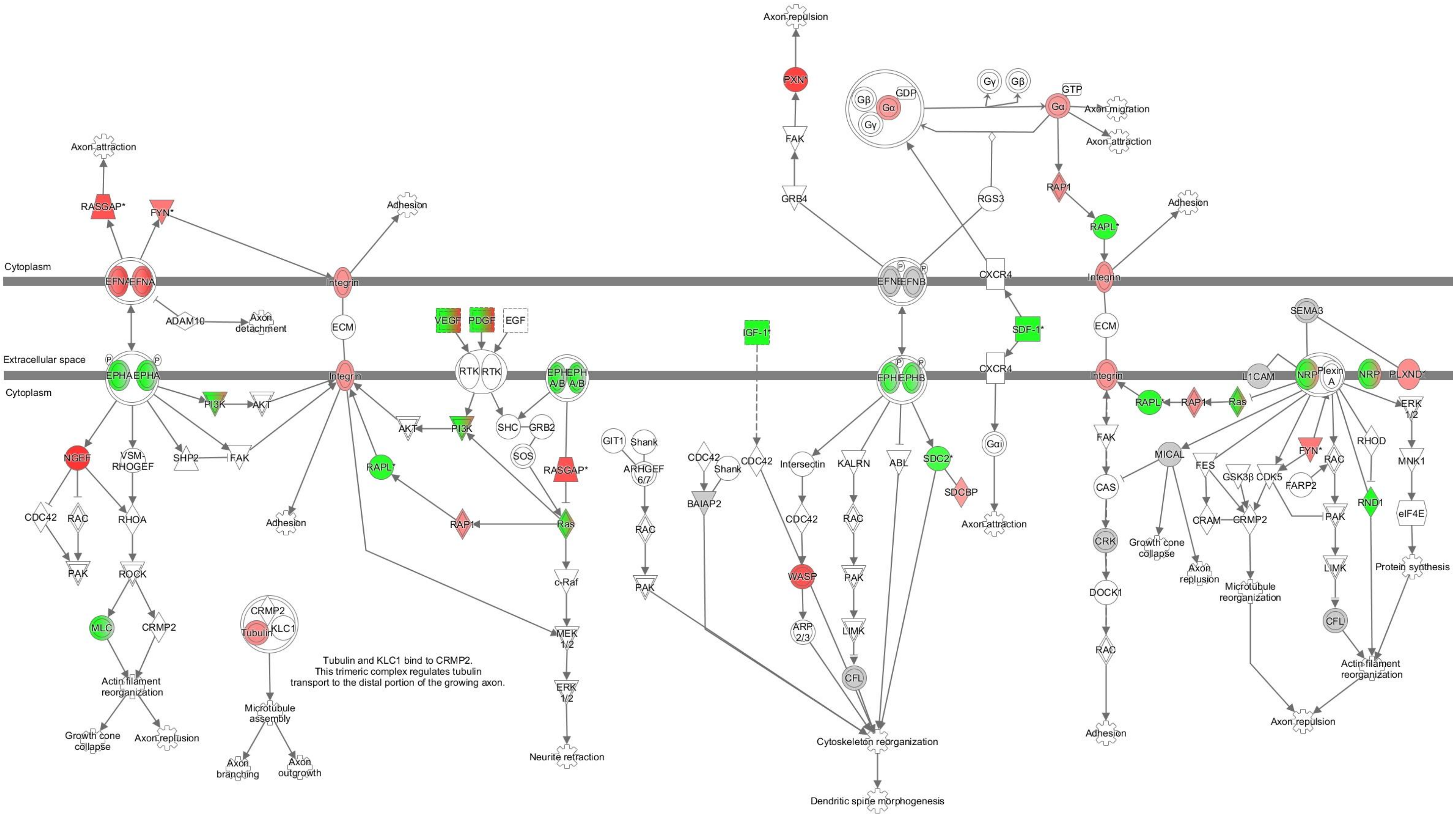

Figure S12. Axonal Guidance Signaling at 6 h

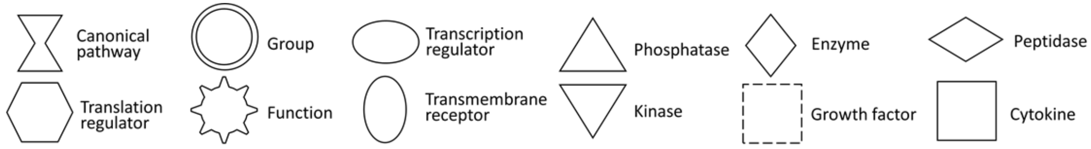

Red: Increased, FDR<0.05 versus control

Green: Decreased, FDR<0.05 versus control

Pathway Analysis Using IPA Software; canonical pathway

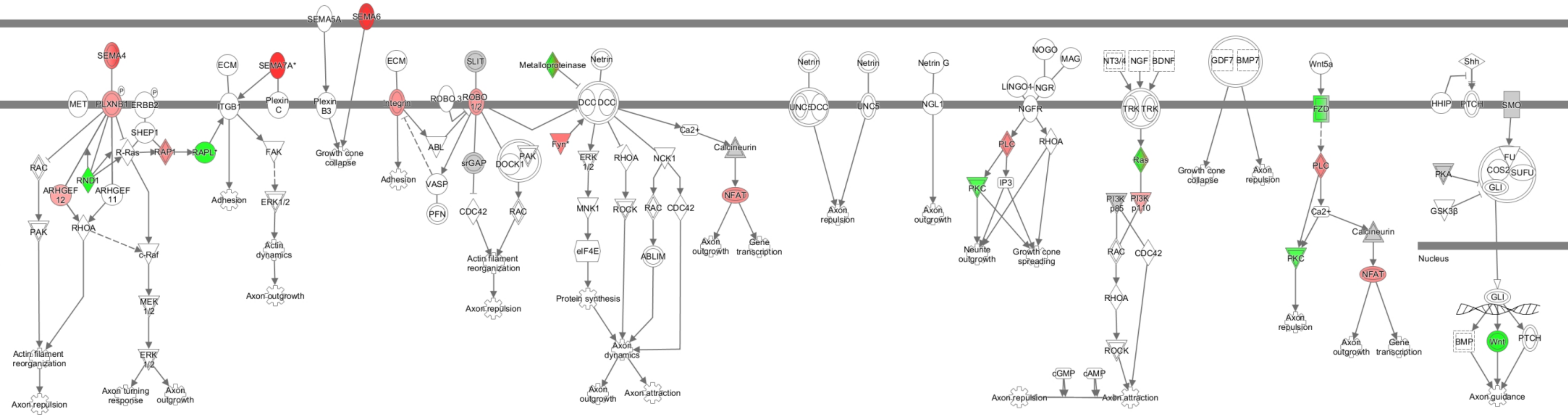

Figure S12. Axonal Guidance Signaling at 6 h (continued)

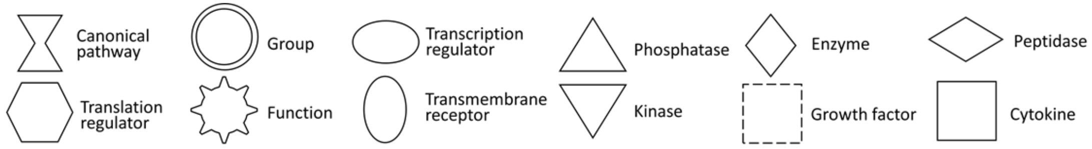

Red: Increased, FDR<0.05 versus control

Green: Decreased, FDR<0.05 versus control

Symbol	Synonym(s)
ABL1	ABL, ABL proto-oncogene 1, non-receptor tyrosine kinase, AI325092, BCR-ABL, c-A, c-ABL, CABL1, c-abl oncogene 1, non-receptor tyrosine kinase, CHDSKM, E430008G22Rik, JTK7, LOC100909750, p145Abl, p150, tyrosine-protein kinase ABL1-like, v-abl
ADAM10	1700031C13Rik, AD10, AD18, ADAM metallopeptidase domain 10, a disintegrin and metallopeptidase domain 10, CD156c, CDw156, DISINTEGRIN-METALLOPROTEASE, HsT18717, kuz, kuzb, kuzbanian, MADM, RAK, RGD1566370, α Secretase
AKT	AKT1/2/3, B/Akt, PKB, RAC-PK
ARHGEF11	GTRAP48, KIAA0380, mKIAA0380, P, PDZ-Rho, PDZ-RHOGEF, Prg, RhoGEF, Rho guanine nucleotide exchange factor 11, Rho guanine nucleotide exchange factor (GEF) 11
ARHGEF12	2310014B11RIK, AU019857, L, LARG, LARG RhoGEF, mKIAA0382, PRO2792, Rho guanine nucleotide exchange factor 12, Rho guanine nucleotide exchange factor (GEF) 12
ARHGEF15	ARGEF15, D130071N09, D530030K12Rik, E5, Ephexin5, Rho guanine nucleotide exchange factor 15, Rho guanine nucleotide exchange factor (GEF) 15, Vms-rhogef, Vsm-RhoGEF
ARHGEF6/7	PIX
Arp2-3	Arp, Arp2-3, ARP2-3 (Actin-related protein complex), Arp Complex
BAIAP2	BAP2, BAP2 alpha, BAP2 α, BAR/IMD domain containing adaptor protein 2, brain-specific angiogenesis inhibitor 1-associated protein 2, FLAF3, IR, IRS-58, IRSP53, R75030, WAML
BCAR1	AI385681, BCAR1 scaffold protein, Cas family member, breast cancer anti-estrogen resistance 1, C, CAS, CAS1, CASS1, Cr, CRKAS, LOC100131601, p130, P130CAP, P130CAS
BDNF	ANON2, Brain derived neurotrophic factor, BULN2, OCD1
BMP	BMP3, BMP-3A, BONE MORPHOGENIC, Osteogenin
BMP7	bone morphogenetic protein 7, O, OP-1, osteogenic protein 1
Ca2+	14127-61-8, Ca+2, calcium, calcium(2+), calcium cation, calcium citrate, calcium ion, calcium, ion (Ca2+), calcium ions, Citracal, tricalcium dicitrate
Calcineurin	Calcineurin, Calcineurin protein(s), CaN, Pp3
cAMP	11002-78-1, 33116-15-3, 3',5'-cyclic AMP, 3',5'-monophosphate, adenosine cyclic, 37839-81-9, (4aR,6R,7R,7aS)-6-(6-aminopurin-9-yl)-2-hydroxy-2-oxo-4a,6,7,7a-tetrahydro-4H-furo[3,2-d][1,3,2]dioxaphosphinin-7-ol, 54532-48-8, 55576-98-2, 60-92-4, 66067-13-8, 68407-13-6, adenosine 3',5'-phosphate, adenosine, cyclic 3',5'-(hydrogen phosphate), adenosine cyclic 3,5 monophosphate, adenosine cyclic 3',5'-monophosphate, adenosine cyclic monophosphate, C10H12N5O6P, cAMP, cyclic-3',5'-monophosphate, adenosine, cyclic adenosine monophosphate, cyclic adenylic acid, cyclic AMP, disodium salt, cyclic AMP, monoammonium salt, cyclic AMP, monopotassium salt, cyclic AMP, monosodium salt, cyclic AMP, sodium salt
CDC42	AI747189, AU018915, CDC42Hs, cell division cycle 42, CELLULAR GROWTH REGULATING, G25K, TKS
CDK5	AW048668, Crk, Crk6, cyclin-dependent kinase 5, LIS7, Neuronal cdc2-like kinase, PSSALRE
cGMP	3',5'-cyclic GMP, 7665-99-8, 9-[(4aR,6R,7R,7aS)-2,7-dihydroxy-2-oxo-4a,6,7,7a-tetrahydro-4H-furo[3,2-d][1,3,2]dioxaphosphinin-6-yl]-2-amino-1H-purin-6-one, C10H12N5O7P, cGMP, guanosine-3',5'-cyclic monophosphate, guanosine 3',5'-cyclic phosphate, guanosine cyclic 3',5'-(hydrogen phosphate)
Cofilin	CFL
CRK/CRKL	CRK, CrkI/II and CrkL
CXCL12	chemokine (C-X-C motif) ligand 12, CXCL12 isoform 1, C-X-C motif chemokine ligand 12, hIRH, IRH, LOC105378278, PB, PBSF, PBSF/SD, Scyb1, SCYB12, SDF-, SDF1, SDF-1alpha, SDF-1α, Stromal cell derived factor 1, TLS, TLSF, TP, TPAR1
CXCR4	b2b220C, b2b220Clo, CD184, CHEMOKINE CXC4 receptor, chemokine (C-X-C motif) receptor 4, CHEMOKINE receptor 4, Cmka, Cmkar4, C-X-C motif chemokine receptor 4, D2S201E, FB22, fu, FUSIN, HM89, HSY3RR, LAP-3, LCR1, LESTR, LOC100047410, NPY3R, NPYR, NPYRL, NPYY3R, PB-C, PB-CKR, PBSF/SDF-1, Sdf, Sdf1r, WHIM, WHIMS
DCC	C030036D22RIK, Colorectal Ca+ supp, CRC18, CRCR1, DCC netrin 1 receptor, deleted in colorectal carcinoma, HGPPS2, Igdcc, IGDCC1, MRMV1, NTN1R1
DOCK1	9130006G06Rik, AI854900, b2b3190C, b2b3190Clo, ced5, D630004B07Rik, dedicator of cyto-kinesis 1, Dock18, DOCK180, LOC679295, RGD1566072
DPYSL2	AI851130, Crm, CRMP-2, DHPRP2, dihydropyrimidinase-like 2, DR, DRP-2, Musunc33, N2A3, TOAD-, TOAD-64, Tuc-2a, Ul, ULIP-2
DPYSL5	CR, CRAM, Crm, CRMP, CRMP-5, CV2, dihydropyrimidinase-like 5, DRP-5, Ulip6
EGF	AI790464, EGF-1, epidermal growth factor, HOMG4, URG
EIF4E	AUTS19, CAP-binding, CBP, EG668879, eIF-4, EIF4E1, EIF4EL1, Eif4e-ps, EIF4F, eukaryotic translation initiation factor 4E, If4, If4e
EphReceptor	EPHA/B
EPHA	EphA receptor, Ephrin A Receptor
EPHB	EphB receptor, Eph Receptor B, Ephrin B Receptor
Ephrin A	Bsk ligand, Ephrin-A
Ephrin B	Ephrin B
ERBB2	1 NDF1, CD340, c-erbB, C-erb-b2, c-ne, c-neu, EGFR2, epidermal growth factor receptor 2, ErbB-, ERBB2 receptor, erb-b2 receptor tyrosine kinase 2, HER-, HER-2, HER-2 (ERBB2), HER2/Neu, I11Ju, I11Jus8, mKIAA3023, MLN 19, Ne, NEU, NGL, p185HER2, TKR1, V-ERBB2 NEURO/GLIOBLASTOMA-DERIVED ONCOGENE
ERK1/2	MAPK p44/42, MAPK p44/p42, p42/44 mapk, P42/p44 erk, P42/p44 mapk, p42/p44 MAP KINASE
FARP2	AI465173, BC009153, D030026M03Rik, F, FERM, ARH/RhoGEF and pleckstrin domain protein 2, FERM, RhoGEF and pleckstrin domain protein 2, FIR, FLJ31039, FRG, mKIAA0793, PLEKHC3
FES	AI586313, BB137047, c-fe, c-fes, feline sarcoma oncogene, FES proto-oncogene, tyrosine kinase, FPS, RGD1564385, V-FPS
Frizzled	Frizzled receptor, FZ, FZD, Wnt receptor
FYN	AI448320, AW552119, C-FYN, Fyn proto-oncogene, FYN proto-oncogene, Src family tyrosine kinase, FYNT, LOC102724705, p59-FYN, p59 Fyn B, SLK, SRC-LIKE KINASE, SYN
G protein	Galphabetagamma, Galpha-Gbeta-Ggamma, Galphai-Gbeta-Ggamma, Galphaq-Gbeta-Ggamma, Gpro, G protein alpha beta gamma, G protein alpha-G protein beta-GDP-G protein gamma, G protein alpha-G protein beta-G protein gamma, G-protein complex, G protein α-G protein β-GDP-G protein γ, G protein α-G protein β-G protein γ, G-protein α-β-γ, Guanine nucleotide binding protein, Gα-Gβ-Gγ, Gαi-Gβ-Gγ, Gαq-Gβ-Gγ, Gαβγ
G proteinalpha	Galpha, G-Protein Alpha Subunit, G protein α, G-Protein α Subunit, Gα
G proteinalpha I	Galphai, Gi, GI alpha, GI α, GNAI, Gn alpha, Gn α, G protein ai, G protein alpha I SUBUNITS, G protein α I, G protein α I SUBUNITS, Gαi
G proteinbeta	Gbeta, G-protein β, Gβ
G proteingamma	Ggamma, G protein gamma SUBUNITS, G-protein γ, G protein γ SUBUNITS, Gγ
GDF7	BMP1, BMP12, growth differentiation factor 7
GDP	146-91-8, [(2R,3S,4R,5R)-5-(2-amino-6-oxo-1H-purin-9-yl)-3,4-dihydroxyoxolan-2-yl]methyl phosphono hydrogen phosphate, C10H15N5O11P2, guanosine 5'-(trihydrogen diphosphate), guanosine diphosphate
GIT1	Cat-, Cat-1, GIT ArfGAP 1, p95C, p95Cat
GRB2	AA408164, ASH, Ash-psi, EGFRBP-GRB2, GRAB2, GRBS, growth factor receptor bound protein 2, MST084, MSTP084, NCKAP2
GSK3B	7330414F15Rik, 8430431H08Rik, C86142, glycogen synthase kinase 3 beta, glycogen synthase kinase 3 β, GSK-, GSK-3, GSK-3be, GSK-3beta, GSK-3β, GSKbeta, GSKβ, Tpk1
GTP	[[(2R,3S,4R,5R)-5-(2-amino-6-oxo-1H-purin-9-yl)-3,4-dihydroxyoxolan-2-yl]methoxy-hydroxyphosphoryl] phosphono hydrogen phosphate, 86-01-1, C10H16N5O14P3, GTP, guanosine 5'-(tetrahydrogen triphosphate), Mg-GTP
HERC2	D15F32S1, D15F32S1h, D15F37S1, D7H15F32S, D7H15F32S1, D7H15F37S, D7H15F37S1, HECT and RLD domain containing E3 ubiquitin protein ligase 2, jdf2, LOC101929832, mKIAA0393, MRT38, p528, r, rjs, SHEP1
HHIP	H, Hedgehog-interacting protein, Hhi, Hhip1, Hi, HIP, Hip1, LOC100366089, RGD1564108
IGF1	C730016P09Rik, IGF, IGF-I, IGF-I-smC, INSULIN-LIKE GROWTH FACTOR 1, insulin like growth factor I, insulin-like somatomedin peptide I, MAD isoform 4, mecasermin, mecasermin recombinant, MGF, Preproinsulin-like growth factor ia, somatomedin C
Integrin	Integrin alpha-beta, integrin-extracellular matrix, INTEGRIN receptor, Integrin α-β
IP3	108340-81-4, 1,4,5-InsP3, [(1R,2S,3R,4R,5S,6R)-2,3,5-trihydroxy-4,6-diphosphonooxycyclohexyl] dihydrogen phosphate, 85166-31-0, 88269-39-0, C6H15O15P3, D-myo-Inositol, 1,4,5-tris(dihydrogen phosphate), D-myo-inositol (1,4,5)-trisphosphate, D-myo-inositol 1,4,5-trisphosphate, inositol 1,4,5-triphosphate, inositol 1,4,5-trisphosphate, Ins(1,4,5)P3, InsP3, IP3, myo-inositol 1,4,5-trisphosphate, phosphatidylinositol 1,4,5-triphosphate
ITGB1	4633401G24RIK, AA409975, AA960159, beta1-Integrin, CD29, CD29 antigen, Fn, FNRB, Gm9863, GPIIA, integrin beta 1 (fibronectin receptor beta), integrin subunit beta 1, integrin subunit β 1, Integrin β 1, integrin β 1 (fibronectin receptor β), Integrin β 1 receptor, MDF2, MSK12, VLAB, VLA-BETA, VLA-β, β1-Integrin, β(1)-integrins
ITSN1	AA517634, AA545208, AI316805, AI839402, AI848451, EHSH, EHSH1, Ese, Ese1, INTERSECTIN, Intersectin 1, intersectin 1 (SH3 domain protein 1A), ITSN, SH3D1A, SH3 domain protein-1A, Sh3p, SH3P17
KALRN	2210407G14RIK, ARHGEF24, AV235988, CHD5, CHDS5, DUET, DUO, E530005C20RIK, Gm539, H, HAPIP, Kalirin, Kalirin7, kalirin RhoGEF kinase, kalirin, RhoGEF kinase, Pcip10, TRAD
KIF7	ACLS, AGBK, HLS2, JBTS12, kinesin family member 7, LOC254571, UNQ340
KLC1	AI874768, kinesin light chain 1, KLC, Kn, KNS2, KNS2A
L1CAM	CAML1, CD171, HSAS, HSAS1, Hyd, L, L1, L1 cell adhesion molecule, L1 isoform, L1-N, MASA, MIC5, NCAM, N-CAM-L1, NEUROGLIAN, NgCAM, S10, SPG1

Symbol	Synonym(s)
LIMK	LIMK1/2, Lim Kinase
LINGO1	4930471K13RIK, AV148400, FLJ14594, L, LE, LERN1, leucine rich repeat and Ig domain containing 1, LIN, LRRN6A, MRT64, UNQ20, UNQ201
LRRC4C	6430556C10RIK, KIAA1580, leucine rich repeat containing 4C, NGL-1, RGD1311013
MAG	1B236, Gm, GMA, myelin-associated glycoprotein, sigle, SIGLEC-4A, SPG75
MAP2K1/2	MEK1/2, MKK1/2
MET	AI838057, AUTS9, c-Met, DFNB97, HGF, HGF Binding, HGFR, LOC360378, met proto-oncogene, MET proto-oncogene, receptor tyrosine kinase, MetR, P, PAR4, RCCP2
Metalloprotease	metallopeptidase, metallopeptidase activity, METALLOPROTEINASE, Mp
MICAL1	MICAL, microtubule associated monooxygenase, calponin and LIM domain containing 1, N, NICAL
MKNK1	2410048M24Rik, MAP kinase-interacting serine/threonine kinase 1, MAPK interacting serine/threonine kinase 1, Mnk, MNK1
MLC	MYL, Myosin Light Chain, Rlc
NCK1	6330586M15Rik, D230010O13RIK, Nc, NCK, NCK adaptor protein 1, NCKalpha, Nck α, non-catalytic region of tyrosine kinase adaptor protein 1, p47Nck
NCK2	4833426I10RIK, Grb, GRB4, LOC100503894, NCK adaptor protein 2, NCKbe, NCKbeta, Nck β, non-catalytic region of tyrosine kinase adaptor protein 2
NEUROPILIN	NRP
NF-AT	NFATc
NGEF	ARHGEF27, BESH3, ephe, EPHEXIN, Ephexin1, neuronal guanine nucleotide exchange factor, Tim, Tims2
NGF	2.5S NGF, Beta-NGF, HSAN5, nerve growth factor, Nerve growth factor, β, NGFB, Ngf beta, Ngf β, β-nerve growth factor, β-NGF
NGFR	CD271, FL-P75NTR, fNGFR, Gp80-LNGFR, LN, LNGFR, nerve growth factor receptor, nerve growth factor receptor (TNFR superfamily, member 16), Ngf Receptor Subtype1, Np75, p7, p75, p75lnfgr, p75LNGFR, p75N, p75 NEUROTROPHIN receptor, p75NGFR, p75NTR, p75(NTR), RNNGFRR, Tnfrs, TNFRSF16
NTNG1	A930010C08RIK, AI853992, KIAA0976, Laminet-1, Lmn, Lmnt1, LOC102724829, Netrin G, NETRIN G1, RGD1563465
P110(pi3k)	p110 PI3K, p110 (pi3k), PI3K P110
Patched	PTC, PTCH
PDGF	Pdgf Receptor Ligand
PFN	Profilin
PI3K	1-phosphatidylinositol 3-kinase, 2.7.1.137, ATP:1-phosphatidyl-1D-myo-inositol 3-phosphotransferase, Phosphatidylinositol 3 kinase, phosphatidylinositol 3'-kinase, PI3-kinase, PtdIns 3 Kinase, type III phosphoinositide 3-kinase, type I phosphatidylinositol kinase, Vps34p
PI3Kp85	p85, p85 PI3K, p85 (pik3r)
PKA	A-Kinase, cAMP-Dependent Protein Kinase, cyclic AMP depended protein kinase, protein KINASE A
PKC	Cnpkc, PKC, Pkc(s), Protein Kinase C
PLC	3.1.4.3, alpha-toxin, Clostridium oedematiens beta- and g-toxins, Clostridium oedematiens β- and g-toxins, Clostridium welchii alpha-toxin, Clostridium welchii α-toxin, heat-labile haemolysin, heat-labile hemolysin, lecithinase C, lipophosphodiesterase C, lipophosphodiesterase I, phosphatidase C, phosphatidylcholine cholinephosphohydrolase, PHOSPHOINOSITIDE SPECIFIC PHOSPHOLIPASE C, Phospholipase C, Pi-plc, α-toxin
Plexin A	PLXN-A
PLXNB3	AI451018, KIAA1206, Pl, PLEXB3, Plexin B3, PLEXR, PLXN6, RGD1560615
PLXNC1	2510048K12Rik, AW742158, CD232, Plexin C, PLEXIN C1, v, VESPR
PLXND1	6230425C21Rik, b2b1863C, b2b1863Clo, b2b553C, b2b553Clo, PLEXD1, Plexin D1
PTK2	FA, Fad, FADK, FADK 1, FAK, FAK1, FAK related non-kinase, FR, p125FAK, pp125FAK, PPP1R71, protein tyrosine kinase 2, PTK2 protein tyrosine kinase 2, TYROSINE KINASE 2
PTPN11	2700084A17Rik, AW536184, BPTP3, CFC, JMML, METCDS, MGC14433, Noonan syndrome 1, NS1, protein tyrosine phosphatase non-receptor type 11, protein tyrosine phosphatase, non-receptor type 11, PTP, PTP-1D, PTP2C, S, SAP-2, Sh, SH-P, SHP-2, SH-PTP2, SH-PTP3, Src homology protein 2, SYP
PXN	AW108311, AW123232, FLJ23042, P, PAX, PAXILLIN
RAF1	6430402F14Rik, AA990557, BB129353, CMD1NN, c-R, Cra, CRAF, Craf1, D830050J10Rik, leukaemiA ONCOGENE HOMOLOG1, LEUKEMIA ONCOGENE HOMOLOG1, NS5, Raf-1 proto-oncogene, serine/threonine kinase, v-, v-Raf, v-raf-leukaemia viral oncogene 1, v-raf-leukemia viral oncogene 1
RAP1	RAP1A/B
RASA1	CM-AVM, CMAVM1, G, GAP, GAPX, p120-, p120GAP, P120RASGAP, PKWS, RASA, RASGAP, RAS p21 protein activator 1
RASSF5	1300019G20Rik, AU042887, MAXP1, No, Nor, NORE1, Nore1A, NORE1B, RAPL, Ras association domain family member 5, Ras association (RalGDS/AF-6) domain family member 5, RASSF3
RGS3	4930506N09Rik, C2, C2PA-, C2PA-RGS3, GRS3, PDZ-R, PDZ-RGS3, regulator of G-protein signaling 3, RG, RGP3, RGS3S, SRB-RGS
RHOA	A, Ar, ARH12, ARHA, Arha1, ARHA2, EDFAOB, R, ras homolog family member A, ras-related homolog 12, RHO1, RHO12, RHOH12
RHOD	AI326383, Ar, ARHD, ras homolog family member D, Rho, RHOHP1, RHOM
RND1	A, A830014L09RIK, ARHS, RHO6, Rho family GTPase 1, RHOS
ROBO3	HGPPS, HGPPS1, HGPS, Rbi, RBIG1, Ri, Rig, RIG1, Ro, Rob, Robo3a, Robo3b, Robo3 (predicted), roundabout guidance receptor 3, Roundabout homolog 3
ROCK	RhoA-Binding Kinase alpha/beta, RhoA-Binding Kinase α/β, Rho Kinase, ROK, ROK alpha/beta, ROK α/β
RRAS	AI573426, p23, R, RAS related, related RAS viral (r-ras) oncogene, Rras1, Rras predicted
RTN4	1110020G17Rik, AA407876, AA409940, AA960376, ASY, C130026I10Rik, mKIAA0886, mKIAA4153, N, Nbla00271, Nbla10545, NgA, NI220/250, No, Nog, NOGO, NOGO-A, Nogo A/B, Nogo B, NSP, NSP-CL, reticulon 4, RTN4-A, RTN4-B1, RTN4-C, RTN-X, Vp20
RTN4R	N, Ng, NGR, NGR1, NOGO66, NOGOR, reticulon 4 receptor
SDC2	4833414L08RIK, AA960457, CD362, fibro, heparan sulphate proteoglycan 1, Hsp, HSPG, HSPG1, Syn, SYND2, syndecan-2
SDCBP	MDA-, MDA-9, ST1, Sy, SYCL, syn, syndecan binding protein, synte, Syntenin, syntenin-1, TACIP18
SEMA3	sema domain, Ig domain, short basic domain, Semaphorin3
SEMA5A	5930434A13, 9130201M22RIK, AI464145, sem, sema domain, seven thrombospondin repeats (type 1 and type 1-like), transmembrane domain (TM) and short cytoplasmic domain, (semaphorin) 5A, SEMAF, semaphorin 5A, semF
SEMA7A	2900057C09RIK, CD108, CDw108, H-SEMA-K1, H-Sema-L, JMH, M-Sema-L, Se, sema domain, immunoglobulin domain (Ig), and GPI membrane anchor, (semaphorin) 7A, SEMAK1, SEMAL, semaphorin 7A (John Milton Hagen blood group)
SHANK2	AUTS17, CORTBP1, CTTNBP1, mKIAA1022, P, ProSAP1, SH3 and multiple ankyrin repeat domains 2, SHANK, SPANK-3
SHC1	p52SHC, p6, p66, p66s, P66shc, Sh, SHC, Shc (46 kDa isoform), SHCA, SHC adaptor protein 1, Shc p66 isoform, src homology 2 domain-containing transforming protein C1
SHH	9530036O11Rik, Dsh, HHG1, HLP3, HPE3, HX, Hxl3, LOC105375595, M100081, MCOPCB5, ShhNC, SMMCI, sonic hedgehog, sonic hedgehog signaling molecule, TPT, TPTPS
SMO	bnb, CRJS, E130215L21Rik, FZD11, Gx, PHLS, SMOH, Smoothened, smoothened, frizzled class receptor
STK36	1700112N14RIK, B930045J24, FU, Fuse, Fused, mKIAA1278, serine/threonine kinase 36, Stk36 (predicted)
SUFU	b2b273C, JBTS32, PRO1280, Su, SUFUH, SUFU negative regulator of hedgehog signaling, SUFUXL
Trk Receptor	NTRK, TRK, tropomyosin-receptor-kinase
Tubulin	microtubule, tubulin complex
UNC5	UNC5H
VASP	vasodilator-stimulated phosphoprotein
WNT5A	8030457G12Rik, hWNT5A, LOC102724616, wingless-type MMTV integration site family, member 5A, Wnt-, Wnt family member 5A

Pathway Analysis Using IPA Software; canonical pathway

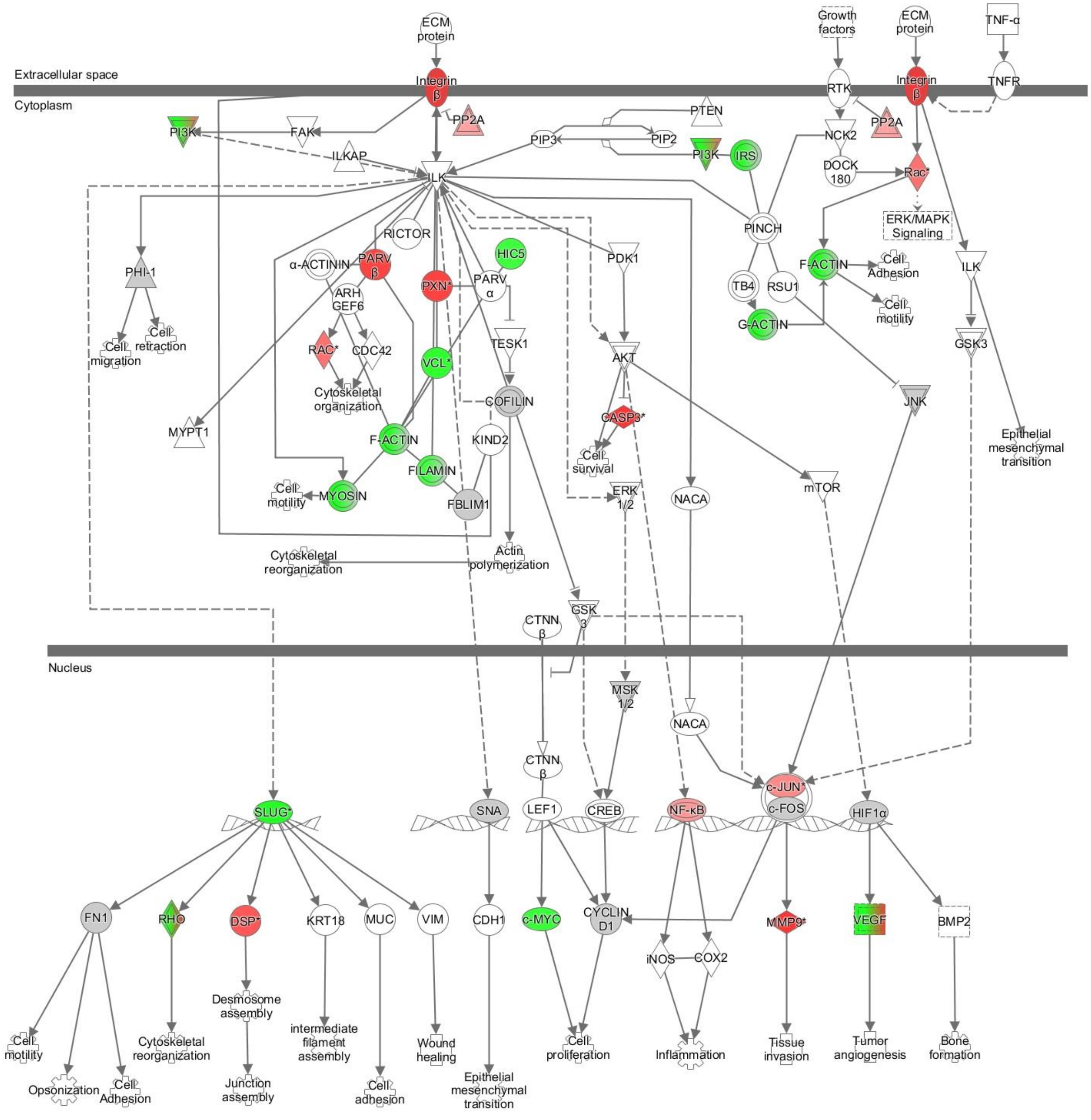

Figure S13. ILK Signaling at 6 h

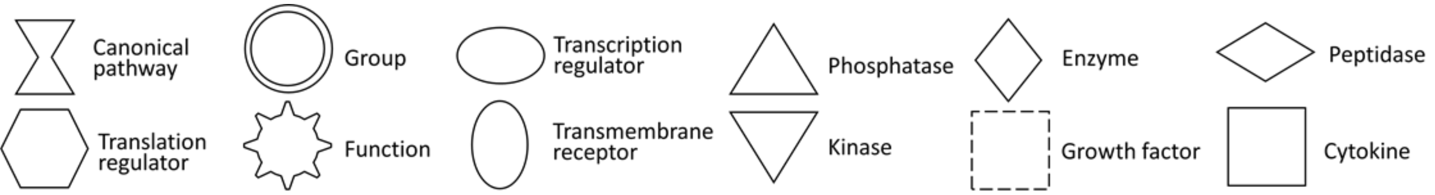

Red: Increased, FDR<0.05 versus solvent control

Green: Decreased, FDR<0.05 versus solvent control

Symbol	Synonym(s)
AKT	AKT1/2/3, B/Akt, PKB, RAC-PK
Alphaactinin	ACTININ, Actinin alpha, Actinin α, ACTN, α-Actinin, α Actinin human
Ap1	activator protein-1, c-Jun
ARHGEF6	1600028C08Rik, 1700038J06Rik, 4930592P22RIK, alp, alpha-PIX, COOL2, MRX46, PIXA, Pix-alpha, Pix-α, Rac/Cdc42 guanine nucleotide exchange factor 6, Rac/Cdc42 guanine nucleotide exchange factor (GEF) 6, α-PIX
Betaintegrin	beta-Integrin, Integrin beta, β- Integrin
BMP2	AI467020, BDA2, Bmp, BMP2A, bone morphogenetic protein 2, BONE MORPHOGENIC protein 2, SSFSC1, SSFSC
CASP3	A830040C14Rik, AC-, AC-3, Casp, Caspase-3, CASPASE-3 p20, CC3, CPP, CPP-32, CPP32B, CPP32-beta, CPP32-β, Ice-like cysteine protease, Lice, mld, mldy, SCA-1, Ya, YAMA
CCND1	AI327039, B-CELL CLL/LYMPHOMA 1, bcl-, BCL1, cD1, CycD1, CYCLIN D1, Cyl-, Cyl-1, D11S287E, G1/S-Specific Cyclin D1, PR, PRAD1, U21B31
CDC42	AI747189, AU018915, CDC42Hs, cell division cycle 42, CELLULAR GROWTH REGULATING, G25K, TKS
CDH1	AA960649, ARC-1, BCDS1, cadherin 1, Cadherin E, CD324, CDHE, CSEIL, E-ca, ECAD, E-cadh, E-cadherin, L-C, L-CAM, Um, UVO, uvomorulin
COFILIN	CFL
CREB	Cbp, Cyclic AMP response element binding
CTNNB1	armadillo, Beta-cat, beta CATENIN, Bfc, Cat, CATENIN beta, catenin beta 1, catenin (cadherin associated protein), beta 1, catenin (cadherin associated protein), β 1, CATENIN β, catenin β 1, CATNB, CTNB1, CTNNB, CTNN beta, CTNN β, EVR7, Mesc, MRD19, NEDSDV, β-cat, β-catenin
DOCK1	9130006G06Rik, AI854900, b2b3190C, b2b3190Clo, ced5, D630004B07Rik, dedicator of cyto-kinesis 1, Dock18, DOCK180, LOC679295, RGD1566072
DSP	2300002E22Rik, 5730453H04RIK, AA407887, AA407888, AW109828, D, DCWHKTA, desmoplakin, DP, DPII, r, rul
ERK1/2	MAPK p44/42, MAPK p44/p42, p42/44 mapk, P42/p44 erk, P42/p44 mapk, p42/p44 MAP KINASE
F Actin	Filamentous Actin
FBLIM1	2410043F08Rik, C, CAL, DKFZp434G171, Fbl, FBLP-1, filamin binding LIM protein 1, Gt10, hnRNP L, mig, migfi, MIGFILIN, migfilin(s)
FERMT2	AA960555, FERM domain containing kindlin 2, fermitin family member 2, Kind, KIND2, KINDLIN 2, Mig, MIG2, Plekh, PLEKHC1, UNC112, UNC112B
FILAMIN	Abp, ABP280/FH1
FN1	cFn, CIG, E330027I09, ED-B, F, FIBNEC, Fibronectin, FIBRONECTIN 1, Fibronectin3 M1, Fibronectin i, FINC, FN, FN1 isoform 1, FNZ, GFND, GFND2, LETS, MSF, SMDCF
FOS	AP-1, c-f, C-FOS, D12Rfj, D12Rfj1, FBJ osteosarcoma oncogene, Fos proto-oncogene, AP-1 transcription factor subunit, p55
Gsk3	Glycogen synthase kinase, Gsk, GSK3 alpha/beta, GSK3 α/β
HIF1A	AA959795, bHLHe7, bHLHe78, HIF-1, HIF1-ALPHA, HIF-1alpha (hydroxylated), HIF-1-α, HIF-1α (hydroxylated), Hypoxia inducible factor 1 alpha subunit, hypoxia inducible factor 1, alpha subunit, hypoxia inducible factor 1 subunit alpha, hypoxia inducible factor 1 subunit α, Hypoxia inducible factor 1 α subunit, hypoxia inducible factor 1, α subunit, MO, MOP1, PASD8
ILK	AA511515, ESTM2, ESTM24, HEL-S-28, ILK-1, ILK-2, integrin-linked kinase, P59, p59ILK
ILKAP	0710007A14RIK, 1600009O09Rik, AF095927, AK055417, ILKAP2, ILKAP3, ILK associated serine/threonine phosphatase, integrin-linked kinase-associated serine/threonine phosphatase 2C, PP2C-D, PP2C-DELTA, PP2C-δ, PPM1O
IRS	INSULIN receptor SUBSTRATE
JNK	JNK 54/46, Jnk p56, JNK/SAPK, JUN KINASE, p40, p47, Sapk/Jnk
JUN	Activator protein 1, AP-1, API-1, c-ju, cJUN, Junc, jun proto-oncogene, Jun proto-oncogene, AP-1 transcription factor subunit, LOC100288387, LOC100291417, LOC100293034, p39, v-Jun, V-jun Avian Sarcoma Virus 17 Oncogene Homolog, V-jun Sarcoma Virus 17 Oncogene Homolog
KRT18	48-kD Keratin, CK-18, Ck D, CYK18, K18, K1cr, keratin 18, Keratin complex 1 acidic gene 18, Krt1-1, KRT1-18
LEF1	3000002B05, AI451430, Lef-, lymphoid enhancer binding factor 1, TCF10, TCF1ALPHA, TCF7L3, TCF/LEF
LIMS	PINCH
MAP2K6	MAPKK6, Mapk kinase 6, MEK6, mitogen-activated protein kinase kinase 6, MKK6, MKK6BE, Prkm, PRKMK6, Rac, SAP, SAPKK-3
MMP9	AW743869, B/MMP, B/MMP9, Clg4, CLG4B, COLLAGENASE type IV, Gelatinase B, GELB, GI 92-kda, MANDP2, matrix metallopeptidase 9, METALLOPROTEINASE 9, MMP-, pro-MMP-9
MTOR	2610315D21Rik, AI327068, fl, Flat, Fr, FRAP, FRAP1, FRAP2, FRB, mechanistic target of rapamycin kinase, RA, RAF, RAFT1, RAPT1, RRAFT1, SKS
MUC1	ADMCKD1, ADMCKD, ADTKD2, CA 15-3, CD227, EM, EMA, Episialin, H23AG, KL-6, MAM6, MCD, MCKD1, MCKD, MUC1-CT, Mucin1, Mucin, mucin 1, cell surface associated, Mucin-1 subunit beta, Mucin-1 subunit β, PANCREATIC MUCIN, PEM, PEMT, PUM
MYC	AU016757, bHLHe3, bHLHe39, CMYC, C-MYC-P64, mMyc, MRTL, Myc2, MYCC, MYC proto-oncogene, bHLH transcription factor, myelocytomatosis oncogene, N, Niard, Nird, RNCMYC
NACA	AL022831, AL024382, alpha-NAC, D12Mgi4, Gm1878, HSD48, mKIAA0363, NACA1, NAC-alpha, NAC-α, Nascent polypeptide associated complex alpha chain, Nascent polypeptide-associated complex alpha polypeptide, nascent polypeptide associated complex subunit alpha, nascent polypeptide associated complex subunit α, Nascent polypeptide associated complex α chain, Nascent polypeptide-associated complex α polypeptide, sk, skNAC, α-NAC
NCK2	4833426I10RIK, GRB4, Grb, LOC100503894, NCK adaptor protein 2, Nck β, NCKbe, NCKbeta, non-catalytic region of tyrosine kinase adaptor protein 2
NFkB	NF-KAPPA B, NF-κ B, nuclear factor-κ b, transcription factor nuclear factor κ b
NOS2	CALCIUM-INDEPENDENT NOS, Hepatocyte NOS, HEP-NOS, Inducible NOS, INOS, LOC497963, MAC-NOS, N, nitric oxide synthase 2, nitric oxide synthase 2, inducible, No, NOS, NOS2A, NOS-II, similar to Nitric oxide synthase, inducible (NOS type II) (Inducible NO synthase) (Inducible NOS) (iNOS)
PARVA	2010012A22Rik, 5430400F08Rik, act, Actopaxin, Actp, AI225929, alpha PARVIN, AU042898, CH-IL, CH-ILKBP, MXRA2, Parvin, Parvin-alpha, parvin, alpha, Parvin-α, parvin, α, α PARVIN
PARVB	aff, affixin, AI595373, AW742462, CGI-56, D15Gsk, D15Gsk1, Parvin-beta, parvin, beta, Parvin-β, parvin, β
PDPK1	3'-PDK, 3-phosphoinositide dependent protein kinase-1, Pdk, PDK1, PDPK2, PDPK2P, PRO0461
PI3K	1-phosphatidylinositol 3-kinase, 2.7.1.137, ATP:1-phosphatidyl-1D-myo-inositol 3-phosphotransferase, Phosphatidylinositol 3 kinase, phosphatidylinositol 3'-kinase, PI3-kinase, PtdIns 3 Kinase, type I phosphatidylinositol kinase, type III phosphoinositide 3-kinase, Vps34p
PIP2	1,2-diacyl-sn-glycero-3-phospho-(1'-myo-inositol-4',5'-bisphosphate), 1-O-(3-sn-phosphatidyl)-1D-myo-inositol 4,5-bis(dihydrogen phosphate), 1-phosphatidyl-1D-myo-inositol 4,5-bisphosphate, C11H19O19P3R2
PIP3	1-phosphatidyl-1D-myo-inositol 3,4,5-trisphosphate, phosphatidylinositol-3,4,5-trisphosphate, phosphoinositide (3,4,5) P3, PI(3,4,5)P3, PIns(3,4,5)P3, PIP3, PtdIns(3,4,5)P3
PP2A	protein PHOSPHATASE 2A, Protein Phosphatase Type2a
PPP1R12A	1200015F06Rik, 5730577I22Rik, AA792106, AV099298, D10Ertd625, D10Ertd625e, GUBS, M110, M130, MBS, MBS130, MBSP, Mypt, MYPT1, protein phosphatase 1 regulatory subunit 12A, protein phosphatase 1, regulatory subunit 12A
PPP1R14B	AOM1, AOM172, FG-4095, P, PHI, PHI-1, Phospholipase c neighboring, PLC, PLCB3N, PNG, Protein phosphatase 1, protein phosphatase 1 regulatory inhibitor subunit 14B, protein phosphatase 1, regulatory (inhibitor) subunit 14B, protein phosphatase 1, regulatory inhibitor subunit 14B, SOM172
PTEN	10q23del, 2310035O07RIK, A130070J02Rik, AI463227, B430203M17RIK, BZS, CWS1, DEC, GLM2, MHAM, MMAC, MMAC1, MUTATED IN MULTIPLE ADVANCED CANCERS, mutated in multiple advanced cancers 1, phosphatase and tensin homolog, PTEN1, PTENbeta, TEP, TEP1
PTGS2	COX, COX-2, CYCLO-OXYGENASE 2, GRIPGHS, hCox-2, hnCOX-2, INDUCIBLE CYCLOOXYGENASE, PES-2, PGG/HS, Pgh, PGHS, PGHS-2, PGH synthase 2, Pgi2 synthase, Pgs2, Pgsi, PHS, PHS-2, PHS II, Prostaglandin endoperoxide synthase 2, Tis, TIS10
PTK2	FA, Fad, FADK, FADK 1, FAK, FAK1, FAK related non-kinase, FR, p125FAK, pp125FAK, PPP1R71, protein tyrosine kinase 2, PTK2 protein tyrosine kinase 2, TYROSINE KINASE 2
PXN	AW108311, AW123232, FLJ23042, P, PAX, PAXILLIN
RHO	GTPase Rho, Rho, Rho Family, RHO-GTPASE, Rho-like Gtpase
RICTOR	4921505C17Rik, 6030405M08Rik, AVO3, AW492497, D530039E11Rik, hAVO3, KIAA1999, Mtorc2, PIA, RPTOR independent companion of MTOR complex 2, RPTOR independent companion of MTOR, complex 2
RSU1	LOC684929, R, Ras suppressor protein 1, rs, RSP-1, RsuI
SNAI1	AI194338, dJ710H13.1, S, SLUGH2, SNA, Sna1, SNAH, SNAIL, SNAIL1, snail family transcriptional repressor 1, snail family zinc finger 1
SNAI2	S, SI, Slu, SLUG, SLUGH, SLUGH1, SNAIL2, snail family transcriptional repressor 2, snail family zinc finger 2, WS2D
TESK1	AI326901, p36-MBPK, testis associated actin remodelling kinase 1, testis specific protein kinase 1
TGFB1I1	AR, ARA55, hic-, HIC-5, TGF beta 1I1, TGF β 1I1, transforming growth factor beta 1 induced transcript 1, transforming growth factor β 1 induced transcript 1, TSC-, TSC-5
TMSB4	TB4, THYMOSIN B4, Thymosin beta4, Thymosin β 4, thymosin β(4)
TNF	AT-TNF, DI, DIF, RATTNF, TMTNF, Tn, TNF-a, TNF-alpha, Tnfs, Tnfsf1a, TNFSF2, TNF-α, TNLG1F, tumor necrosis factor, Tumor Necrosis Factor α, tumor necrosis factor, α, tumour necrosis factor, tumour Necrosis Factor Alpha, tumour necrosis factor, alpha, tumour Necrosis Factor α, tumour necrosis factor, α
TNFRSF1A	CD120, CD120a, FPF, p5, p55, p55-R, p55 TNF alpha receptor, p55 TNF α receptor, p60, TBP1, TN, TNF-, TNF-a, TNFaI, TNF-alphaR1, Tnf alpha receptor p60, TNFAR, TNF-R, TNF-R1, Tnfr-2, TNF-R55, TNFR60, TNF receptor 1, TNF receptor superfamily member 1A, TNF-R-I, TNFRp55, Tnf-α Receptor 2, Tnf α receptor p60, Tnf α Receptor Type1, Tnf-α receptor type I, tumor necrosis factor receptor superfamily, member 1a, tumour necrosis factor receptor 1, tumour necrosis factor receptor superfamily, member 1a
VCL	9430097D22, AA571387, AI462105, AW545629, CMD1W, CMH15, HEL114, MV, MVCL, Vcl predicted, Vinculin
VIM	PAL-E, Vimentin

Pathway Analysis Using IPA Software; canonical pathway

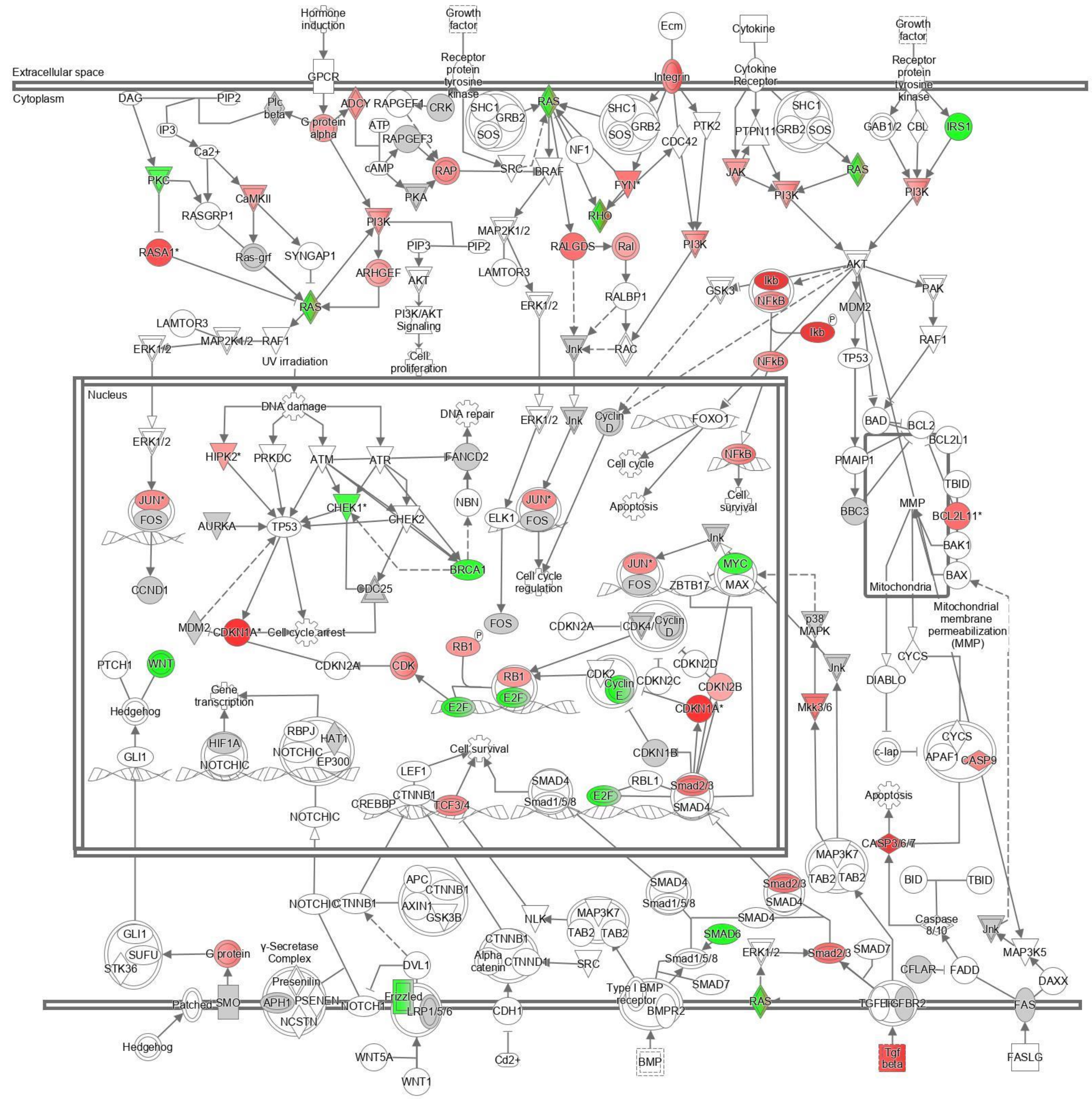

Figure S14. Molecular Mechanism of cancer at 6 h

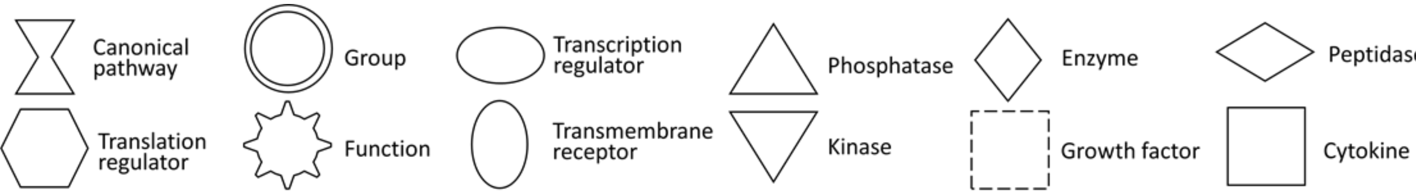

Red: Increased, FDR<0.05 versus solvent control

Green: Decreased, FDR<0.05 versus solvent control

Symbol	Synonym(s)
ADCY	3',5'-cyclic AMP synthetase, 4.6.1.1, AC, Adenylate Cyclase, Adenyl Cyclase, Adenylyl cyclase, ATP diphosphate-lyase (cyclizing), mAC, sAC
AKT	AKT1/2/3, B/Akt, PKB, RAC-PK
Alphacatenin	CTNN alpha, CTNN α, α catenin
Ap1	activator protein-1, c-Jun
APAF1	6230400I06RIK, Ap, Apaf1l, apoptotic peptidase activating factor 1, CED4, fog, mKIAA0413
APC	AI047805, APC1, Apc7, APC (PROC), APC regulator of WNT signaling pathway, APC, WNT signaling pathway regulator, AU020952, AW124434, BTPS2, CC1, DESMD, DP2, DP2.5, DP3, Familial adenomatous polyposis, GS, M, mAPC, Min, PPP1R46, RATAPC
Apoptosome	APAF1-Caspase 9-CytoC, apoptosis adaptor protein complex, Cytochrome C-APAF1-Caspase 9
ARHGEF	Ras GEF, RhOGEF
ATM	AI256621, AT1, ATA, ataxia telangiectasia mutated, ATC, ATD, ATDC, ATE, ATM serine/threonine kinase, C030026E19RIK, TEL1, TELO1
ATP	[[(2R,3S,4R,5R)-5-(6-aminopurin-9-yl)-3,4-dihydroxyoxolan-2-yl]methoxy-hydroxyphosphoryl] phosphono hydrogen phosphate, 56-65-5, 9-beta-D-arabinofuranosyladenine 5'-triphosphate, 9-β-D-arabinofuranosyladenine 5'-triphosphate, adenosine 5'-(tetrahydrogen triphosphate), adenosine 5'-triphosphate, ATP, ATP4-, C10H16N5O13P3
ATR	ataxia telangiectasia and Rad3 related, Ataxia-telangiectasia-like, ATR serine/threonine kinase, FCTCS, FRP1, LOC100365674, LOC367198, LOC684113, MEC1, SCKL, SCKL1
AURKA	AI, AIK, AIRK1, Ar, ARK-1, Au, AU019385, AURA, AURORA 2, AURORA A, AURORA KINASE, aurora kinase A, Aurora Related Kinase1, AW539821, Ayk, Ayk1, BTAK, I, IA, IAK, IAK1, PPP1R47, Stk, STK15, STK6, STK7
AXIN1	AI316800, AXIN, AXIN form I, Fu, fused, Kb, Ki, kinky, knobbly, PPP1R49
BAD	AI325008, Bad v1, Bad v2, BBC2, BCL2-associated agonist of cell death, BCL2L8
BAK1	Ba, BAK, BAK-LIKE, BCL2-antagonist/killer 1, BCL2L7, CDN1, N-B, N-BAK1
BAX	Bcl2-associated X, BCL2 associated X, apoptosis regulator, BCL2-associated X protein, BCL2L4
BBC3	BCL2 binding component 3, JFY-1, PU, PUMA, PUMA/JFY1
BCL2	AW986256, B cell leukaemia/lymphoma 2, B cell leukemia/lymphoma 2, Bcl-, Bcl2 alpha, BCL2 apoptosis regulator, BCL2, apoptosis regulator, Bcl2 α, C430015F12Rik, D630044D05RIK, D830018M01RIK, LOC100046608, ORF16, PPP1R50
BCL2L1	bBclxl, Bcl, BCL2L, BCL2-like 1, BCLX, Bcl-X beta, Bclx gamma, BCL-XL/S, Bcl-X β, Bclx γ, PPP1R52
BCL2L11	1500006F24RIK, BAM, BCL2 like 11, BCL2-like 11 (apoptosis facilitator), Bi, BIM, Bo, BOD, BODL, LOC150819
BMP	BMP3, BMP-3A, BONE MORPHOGENIC, Osteogenin
BMPR2	2610024H22RIK, AL117858, AW546137, BB189135, BM, BMP-, BMP-2, BMPR3, BMPR-II, BMR2, bone morphogenetic protein receptor type 2, bone morphogenetic protein receptor, type II (serine/threonine kinase), BRK-3, Gm20272, POVD1, PPH1, T-ALK, Type ii bmp receptor
BRAF	9930012E13RIK, AA120551, AA387315, AA473386, AI447469, Bra, B-RAF1, Braf-2, B-Raf proto-oncogene, serine/threonine kinase, Braf transforming gene, C230098H17, C87398, D6Ertd631, D6Ertd631e, NS7, RAFB, RAFB1
BRCA1	BRCA1 DNA repair associated, BRCA1, DNA repair associated, BRCAI, BRCC1, breast cancer 1, early onset, BROVCA1, FANCS, PNCA4, PPP1R53, PSCP, RNF53
c-Iap	IAP, NAIP
Ca2+	14127-61-8, Ca+2, calcium, calcium(2+), calcium cation, calcium citrate, calcium ion, calcium, ion (Ca2+), calcium ions, Citracal, tricalcium dicitrate
CaMKII	CA2+/CALMODULIN DEPENDENT KINASE II, Ca+/calmodulin-dependent protein kinase ii, calmodulin-dependent protein kinase 2, Calmodulin Kinase II, CAMK2, CaM Kinase II, Ccdpk ii
cAMP	11002-78-1, 33116-15-3, 3',5'-cyclic AMP, 3',5'-monophosphate, adenosine cyclic, 37839-81-9, (4aR,6R,7R,7aS)-6-(6-aminopurin-9-yl)-2-hydroxy-2-oxo-4a,6,7,7a-tetrahydro-4H-furo[3,2-d][1,3,2]dioxaphosphinin-7-ol, 54532-48-8, 55576-98-2, 60-92-4, 66067-13-8, 68407-13-6, adenosine 3',5'-phosphate, adenosine, cyclic 3',5'-(hydrogen phosphate), adenosine cyclic 3,5 monophosphate, adenosine cyclic 3',5'-monophosphate, adenosine cyclic monophosphate, C10H12N5O6P, cAMP, cyclic-3',5'-monophosphate, adenosine, cyclic adenosine monophosphate, cyclic adenylic acid, cyclic AMP, disodium salt, cyclic AMP, monoammonium salt, cyclic AMP, monopotassium salt, cyclic AMP, monosodium salt, cyclic AMP, sodium salt
CASP3/6/7	CASP3/6/7, Caspase 3, 6, 7, Caspase-3, -6, and -7
CASP9	AI115399, APAF-3, AW493809, Casp, Casp9 v1, Caspase-9, ICE-, ICE-LAP6, MCH6, PPP1R56
Caspase8/10	Casp8/10, Caspase 8,10
CBL	4732447J05Rik, Casitas B-lineage lymphoma, CBL2, CBLA, Cbl proto-oncogene, Cbl ubiquitin ligase, c-Cb, C-CBL, FRA11B, LOC283153, NSLL, p120 Cbl, RGD1561386, RNF55
CCND1	AI327039, B-CELL CLL/LYMPHOMA 1, bcl-, BCL1, cD1, CycD1, CYCLIN D1, Cyl-, Cyl-1, D11S287E, G1/S-Specific Cyclin D1, PR, PRAD1, U21B31
Cd2+	22537-48-0, cadmium(2+), cadmium acetate, cadmium cation, cadmium ion, cadmium, ion (Cd2+), Cd+2
CDC25	mRNA encoding Cdc25-like
CDC42	AI747189, AU018915, CDC42Hs, cell division cycle 42, CELLULAR GROWTH REGULATING, G25K, TKS
CDH1	AA960649, ARC-1, BCDS1, cadherin 1, Cadherin E, CD324, CDHE, CSEIL, E-ca, ECAD, E-cadh, E-cadherin, L-C, L-CAM, Um, UVO, uvomorulin
CDK	Cdks, cyclin-dependent kinase, Cyclin-Dependent Kinases, G1 CDK
CDK2	A630093N05Rik, CDC2-RELATED KINASE, CDKN2, Cyclin A associated kinase, cyclin-dependent kinase 2, CYCLIN E ASSOCIATED KINASE, p33(CDK2)
CDK2-CyclinE	Cyclin E-CDK2
CDKN1A	CAP, CAP20, CDK, CDKI, Cdkn, CDKN1, CDKNA1, CI, CIP1, cyclin-dependent kinase inhibitor 1A, cyclin-dependent kinase inhibitor 1A (P21), mda, MDA-6, P2, P21, p21C, p21Cip, p21CIP1, P21W, p21WAF, p21Waf1, PzI Cyclin-Dependent Kinase Inhibitor, SD, SDI1, UV96, Waf, WAF1
CDKN1B	AA408329, AI843786, Cdki1b, CDKN4, cyclin-dependent kinase inhibitor 1B, CYCLIN-DEPENDENT KINASE INHIBITOR P27, KIP1, MEN1B, MEN4, p2, p27, p27K, P27kip, P27KIP1, P28-ICK
CDKN2A	A, Arf, ARF-INK4a, CDK4I, CDKN2, CMM2, CYCLIN-DEPENDENT KINASE INHIBITOR 2A, INK4, INK4A, INK4a-ARF, Ink4a/Arf, MLM, MTS, MTS-1, p1, p14ARF/ p16INK4a, p16, p16/ARF, p16Cdkn2a, p16I, p16 INK4, p16/ INK4a, P19, p19ARF, Pct, PCTR1, TP16
CDKN2B	AV083695, CDK4I, cyclin-dependent kinase inhibitor 2B, INK4B, MTS, MTS2, p1, P15, p15IN, p15INK4, p15INK4b, p15(INK4b)
CDKN2C	C77269, CDKN6, cyclin-dependent kinase inhibitor 2C, INK, INK4C, p1, p18, p18IN, p18-INK4C, p18-INK6
CDKN2D	cyclin dependent kinase inhibitor 2D, INK, INK4D, p1, p19, p19IN, p19-INK4D
CFLAR	2310024N18RIK, A430105C05Rik, AU021929, Ca, CASH, CASP8 and FADD-like apoptosis regulator, CASP8AP1, Caspase 8 associated, Casper, c-F, c-FLIP, CLARP, F, FLAME, FLAME-1, FLICE-LIKE IP, FLIP, Gm9845, I-FLICE, LOC102724614, MRIT
CHEK1	C85740, checkpoint kinase 1, CHK1, rad27
CHEK2	CDS1, Check2, checkpoint kinase 2, CHK2, hCds1, HUCDS1, LFS2, PP1425, Rad, RAD53
CREBBP	AW558298, CB, CBP, CBP/p300, CREB binding protein, KAT, KAT3A, MKHK1, p300/CBP, RSTS, RSTS1, RTS
CRK	c-Crk, c-Crk2, Cr, CRK2, Crko, CRK proto-oncogene, adaptor protein, FLJ11558, p38, v-crk avian sarcoma virus CT10 oncogene homolog
CTNNB1	armadillo, Beta-cat, beta CATENIN, Bfc, Cat, CATENIN beta, catenin beta 1, catenin (cadherin associated protein), beta 1, catenin (cadherin associated protein), β 1, CATENIN β, catenin β 1, CATNB, CTNB1, CTNNB, CTNN beta, CTNN β, EVR7, Mesc, MRD19, NEDSDV, β-cat, β-catenin
CTNND1	AA409437, AU019353, BCDS2, Ca, CAS, catenin (cadherin associated protein), delta 1, catenin (cadherin associated protein), δ 1, catenin delta 1, catenin δ 1, CATNS, Ctn, CTNND, CTNN delta, CTNN delta1, CTNN δ, CTNN δ1, mKIAA0384, P12, P120, P120CAS, p120(CAS), p120-Catenin, P120CTN, p120(CTN), Pp120
CTNNα-CTNN β-CTNNδ	CTNNalpha-CTNNbeta-CTNNdelta
CyclinD	CycD, Cyclin D1
CYCS	CYC, CYCSA, CYTC, CYTOC, CYTOCHROME C, cytochrome c, somatic, cytochrome c, somatic-like, ENSMUSG00000058927, HCS, LOC100363502, THC4, X laevis XLCL2
DAG	DAG, diacylglycerides, diglyceride
DAXX	BING2, DAP6, death-domain associated protein, EAP1, Fas death domain-associated protein, PML ASSOCIATED FACTOR
DIABLO	0610041G12Rik, 1700006L01RIK, AU040403, DFNA64, diablo IAP-binding mitochondrial protein, diablo, IAP-binding mitochondrial protein, Sm, SMAC
DVL1	DISHEVELED, dishevelled segment polarity protein 1, DRS2, DSH, DVL, DVL1L1, DVL1P1, mKIAA4029
ELK1	ELK, ELK1, member of ETS oncogene family, ETS transcription factor ELK1, p62TCF, RGD:2549, TCF/ELK
EP300	A430090G16, A730011L11, E1A binding protein p300, KAT3, KAT3B, MKHK2, p30, p300, p300 HAT, RSTS2
ERK1/2	MAPK p44/42, MAPK p44/p42, p42/44 mapk, P42/p44 erk, P42/p44 mapk, p42/p44 MAP KINASE
FADD	DEATH domain-containing ADAPTOR, Fas associated via death domain, Fas (TNFRSF6)-associated via death domain, GIG3, MORT1, Mort1/F, Mort1/FADD
FANCD2	2410150O07RIK, AU015151, BB137857, FA4, FACD, FA complementation group D2, FAD, FA-D2, FANCD, Fanconi anaemia, complementation group D2, Fanconi anemia, complementation group D2
FAS	AI196731, ALPS1A, AP, APO-1, APT1, CD95, CD95L, CD95 receptor, FAS1, FAS/APO1, Fas cell surface death receptor, FasR, FASTM, Fas (TNF receptor superfamily member 6), lpr, Receptor for Fas Ligand, Receptors for Fas Ligand, TNF, Tnfr, TNFR6, Tnf receptor member 6, TNFRSF6
FASLG	ALPS1B, APT1, APT1LG1, APTL, CD178, CD95, CD95-L, F, Fa, FASL, Fas Ligand, Fas ligand (TNF superfamily, member 6), gld, mFasL, Tnfl6, Tnfs, TNFSF6, TNLG1A
FOS	AP-1, c-f, C-FOS, D12Rfj, D12Rfj1, FBJ osteosarcoma oncogene, Fos proto-oncogene, AP-1 transcription factor subunit, p55
FOXO1	Afx, Afxh, AI876417, FKH, FKH1, FKHR, FKHR1, Forkhead, forkhead box O1, Fox, FOXO1A
Frizzled	Frizzled receptor, FZ, FZD, Wnt receptor
Frizzled-LRP	FZD-LRP1/5/6
FYN	AI448320, AW552119, C-FYN, Fyn proto-oncogene, FYN proto-oncogene, Src family tyrosine kinase, FYNT, LOC102724705, p59-FYN, p59 Fyn B, SLK, SRC-LIKE KINASE, SYN
G protein	Galphabetagamma, Galpha-Gbeta-Ggamma, Galphai-Gbeta-Ggamma, Galphaq-Gbeta-Ggamma, Gpro, G protein alpha beta gamma, G protein alpha-G protein beta-GDP-G protein gamma, G protein alpha-G protein beta-G protein gamma, G-protein complex, G protein α-G protein β-GDP-G protein γ, G protein α-G protein β-G protein γ, G-protein α-β-γ, Guanine nucleotide binding protein, Gα-Gβ-Gγ, Gαi-Gβ-Gγ, Gαq-Gβ-Gγ, Gαβγ
G proteinalpha	Galpha, G-Protein Alpha Subunit, G protein α, G-Protein α Subunit, Gα
Gammasecretase	Gamma Secretase, Secretase γ, γ-Secretase
GLI1	AV235269, GL1, GLI, GLI family zinc finger 1, GLI-Kruppel family member GLI1, PAPA8, PPD1, Zfp-, ZFP5
GRB2	AA408164, ASH, Ash-psi, EGFRBP-GRB2, GRAB2, GRBS, growth factor receptor bound protein 2, MST084, MSTP084, NCKAP2
Grb2-Shc1-Sos	Grb2-Sos-Shc, SHC-GRB2-SOS
GSK3	Glycogen synthase kinase, Gsk, GSK3 alpha/beta, GSK3 α/β
GSK3beta-Axin-APC-Ctnnbeta	APC-CTNNbeta-AXIN-GSK3beta, APC-CTNNβ-AXIN-GSK3β, AXIN-APC-GSKbeta-CTNNbeta, AXIN-APC-GSKβ-CTNNβ, GSK3β-AXIN-APC-CTNNβ
GSK3B	7330414F15Rik, 8430431H08Rik, C86142, glycogen synthase kinase 3 beta, glycogen synthase kinase 3 β, GSK-, GSK-3, GSK-3be, GSK-3beta, GSK-3β, GSKbeta, GSKβ, Tpk1
HAT1	2410071B14Rik, AA536933, histone acetyltransferase 1, histone aminotransferase 1, KAT, KAT1
Hedgehog	Hh
HIF1A	AA959795, bHLHe7, bHLHe78, HIF-1, HIF1-ALPHA, HIF-1alpha (hydroxylated), HIF-1-α, HIF-1α (hydroxylated), Hypoxia inducible factor 1 alpha subunit, hypoxia inducible factor 1, alpha subunit, hypoxia inducible factor 1 subunit alpha, hypoxia inducible factor 1 subunit α, Hypoxia inducible factor 1 α subunit, hypoxia inducible factor 1, α subunit, MO, MOP1, PASD8

Symbol	Synonym(s)
HIF1alpha-NICD	HIF1α-NICD
HIPK2	1110014O20RIK, B230339E18RIK, homeodomain interacting protein kinase 2, LOC100505582, LOC653052, PRO0593, St, Stank
Ikb	I KAPPA B, Ikbeta, Ikβ, Iκ-B
IkB-NfkB	IkappaB-NFkappaB, IκB-NFκB, NFkB-IkB
Integrin	Integrin alpha-beta, integrin-extracellular matrix, INTEGRIN receptor, Integrin α-β
IP3	27121-73-9, inositol trisphosphate, IP3, myo-inositol, tris(dihydrogen phosphate)
IRS1	ENSMUSG00000022591, G972, G972R, HIRS-1, insulin receptor substrate 1, IR, IRS1IRM
JAK	JAK kinase
Jnk	JNK 54/46, Jnk p56, JNK/SAPK, JUN KINASE, p40, p47, Sapk/Jnk
JUN	Activator protein 1, AP-1, API-1, c-ju, cJUN, Junc, jun proto-oncogene, Jun proto-oncogene, AP-1 transcription factor subunit, LOC100288387, LOC100291417, LOC100293034, p39, v-Jun, V-jun Avian Sarcoma Virus 17 Oncogene Homolog, V-jun Sarcoma Virus 17 Oncogene Homolog
LAMTOR3	AW556229, late endosomal/lysosomal adaptor, MAPK and MTOR activator 3, LOC100132990, Map, Map2k, MAP2K1IP1, MAPBP, MAPKSP1, Mek binding partner 1, Mp, MP1, PRO0633, Ragulator3
LEF1	3000002B05, AI451430, Lef-, lymphoid enhancer binding factor 1, TCF10, TCF1ALPHA, TCF7L3, TCF/LEF
MAP2K1/2	MEK1/2, MKK1/2
MAP3K5	7420452D20Rik, A, APOPTOSIS SIGNAL REGULATED KINASE 1, AS, ASK, ASK1, M3K5, MAPKKK5, MEKK5, mitogen-activated protein kinase kinase kinase 5, RGD1306565
MAP3K7	CSCF, FMD2, Map3k7 predicted, MEKK7, mitogen-activated protein kinase kinase kinase 7, TAK1, TGF1a, tgf β activated kinase 1
Map3k7-Map3k7ip1-	Tab1-Tab2-Tak1
MAX	AA960152, AI875693, bHLHd, bHLHd4, bHLHd5, bHLHd6, bHLHd7, bHLHd8, Max protein, MYC associated factor X, Myn
Max-Myc	cMyc-MAX, Myc-MAX
MDM2	1700007J15Rik, AA415488, ACTFS, hdm2, HDMX, LSKB, MDM2-A1, MDM2 proto-oncogene, MGC5370, Transformed 3t3 cell double minute 2, transformed mouse 3T3 cell double minute 2
Mkk3/6	MEK3/6, Mkk3/6 (mitogen activated protein kinase kinase 3/6), MKK3/MKK6
MYC	AU016757, bHLHe3, bHLHe39, CMYC, C-MYC-P64, mMyc, MRTL, Myc2, MYCC, MYC proto-oncogene, bHLH transcription factor, myelocytomatosis oncogene, N, Niard, Nird, RNCMYC
NBN	ATV, AT-V1, AT-V2, Nb, NBS, NBS1, NIBRIN, P95
NCSTN	9430068N19Rik, AA727311, APH2, ATAG1874, D1Dau13, D1Dau13e, Kiaa0253, mKIAA0253, Nc, NCT, ni, NICASTRIN
NF1	AW494271, Dsk, Dsk9, E030030H24RIK, LOC646021, Mhdads, Mhdadsk9, Neurofibromatosis 1, NEUROFIBROMIN, neurofibromin 1, Nf-, NF1-GAP, NFNS, VRNF, WSS
NFkB	NF-KAPPA B, NF-κ B, nuclear factor-κ b, transcription factor nuclear factor κ b
NLK	AI194375, LOC100044468, nemo like kinase, RGD1561602
NOTCHIC	9930111A19Rik, AOS5, AOVD1, hN1, lin, lin-12, Mi, Mis6, N, N1, NOTCH, notch receptor 1, Ta, TAN1
p38MAPK	P38, p38 MAP KINASE, P38 MITOGEN-ACTIVATED protein KINASE
Patched	PTC, PTCH
PI3K	1-phosphatidylinositol 3-kinase, 2.7.1.137, ATP:1-phosphatidyl-1D-myo-inositol 3-phosphotransferase, Phosphatidylinositol 3 kinase, phosphatidylinositol 3'-kinase, PI3-kinase, PtdIns 3 Kinase, type III phosphoinositide 3-kinase, type I phosphatidylinositol kinase, Vps34p
PIP2	1,2-diacyl-sn-glycero-3-phospho-(1'-myo-inositol-4',5'-bisphosphate), 1-O-(3-sn-phosphatidyl)-1D-myo-inositol 4,5-bis(dihydrogen phosphate), 1-phosphatidyl-1D-myo-inositol 4,5-bisphosphate, C11H19O19P3R2
PIP3	1-phosphatidyl-1D-myo-inositol 3,4,5-trisphosphate, phosphatidylinositol-3,4,5-trisphosphate, phosphoinositide (3,4,5) P3, PI(3,4,5)P3, PIns(3,4,5)P3, PIP3, PtdIns(3,4,5)P3
PKA	A-Kinase, cAMP-Dependent Protein Kinase, cyclic AMP depended protein kinase, protein KINASE A
PKC	Cnpkc, PKC, Pkc(s), Protein Kinase C
Plcbeta	Phospholipase c beta, Phospholipase C β, PLCB, PLCβ
PMAIP1	APR, N, NOXA, phorbol-12-myristate-13-acetate-induced protein 1
Presenilin	PS, PS1/2, PSEN1/2
PRKDC	AI326420, AU019811, DNA-, DNA-DEPENDENT protein KINASE, DNAPDcs, DNAPK, DNA-PKC, DNA-PKcs, DNPK1, DOX, DOXNPH, dxn, dxnph, HYRC, HYRC1, IMD26, p350, p460, Prkdc predicted, protein kinase, DNA activated, catalytic polypeptide, protein kinase, DNA-activated, catalytic subunit, scid, slip, XRCC, XRCC7
PSENEN	1700023M09Rik, ACNINV2, MDS033, MSTP064, PEN-2, presenilin enhancer gamma secretase subunit, presenilin enhancer, gamma-secretase subunit, presenilin enhancer γ secretase subunit, presenilin enhancer, γ-secretase subunit, RGD1312037
PTCH1	A230106A15RIK, BCNS, mes, NBCCS, patched 1, Pt, PTC, PTC1, PTCH, Ptch2, wi, wig
PTK2	FA, Fad, FADK, FADK 1, FAK, FAK1, FAK related non-kinase, FR, p125FAK, pp125FAK, PPP1R71, protein tyrosine kinase 2, PTK2 protein tyrosine kinase 2, TYROSINE KINASE 2
PTPN11	2700084A17Rik, AW536184, BPTP3, CFC, JMML, METCDS, MGC14433, Noonan syndrome 1, NS1, protein tyrosine phosphatase non-receptor type 11, protein tyrosine phosphatase, non-receptor type 11, PTP, PTP-1D, PTP2C, S, SAP-2, Sh, SH-P, SHP-2, SH-PTP2, SH-PTP3, Src homology protein 2, SYP
RAF1	6430402F14Rik, AA990557, BB129353, CMD1NN, c-R, Cra, CRAF, Craf1, D830050J10Rik, leukaemiA ONCOGENE HOMOLOG1, LEUKEMIA ONCOGENE HOMOLOG1, NS5, Raf-1 proto-oncogene, serine/threonine kinase, v-, v-Raf, v-raf-leukaemia viral oncogene 1, v-raf-leukemia viral oncogene 1
Ral	Ral A/B
RALBP1	DNP-SG ATPase, R, ralA binding protein 1, Ral GAP, Rik, RIP1, RL, RLIP1, RLIP76
RALGDS	Gn, Gnds, Hs.560937, mKIAA1308, Ra, RalGDSB, RalGEF, ral guanine nucleotide dissociation stimulator, Rg, RGDS, RGF
RAPGEF1	4932418O06Rik, C3G, C3G-1, C3G-2, Grf, GRF2, Rap guanine nucleotide exchange factor 1, Rap guanine nucleotide exchange factor (GEF) 1
RAPGEF3	2310016P22Rik, 9330170P05Rik, bcm910, CAMP-GEFI, CGEF1, Epa, EPAC, EPAC1, HSU79275, Rap guanine nucleotide exchange factor 3, Rap guanine nucleotide exchange factor (GEF) 3
Ras-grf	Guanine Nucleotide Releasing
RASA1	CM-AVM, CMAVM1, G, GAP, GAPX, p120-, p120GAP, P120RASGAP, PKWS, RASA, RASGAP, RAS p21 protein activator 1
RASGRP1	CALDAG-GEFI, CALDAG-GEFII, IMD64, RASGRP, RAS guanyl releasing protein 1
Rb-E2Ftranscriptionrepression	Rb1-E2f1, Rb-E2F, Rb-E2F1
RB1	OSRC, p, p105, p105-Rb, p110 RB, p110-RB1, pp105, pp110, PPP1R130, pRb, R, RB, RB-ASSOCIATED, RB transcriptional corepressor 1, Retinoblastome tumor-suppression protein rb
RBL1	AW547426, CP107, LOC683869, p10, p107, PRB1, RB transcriptional corepressor like 1
RBPJ	AI843960, AOS3, CBF-1, csl, Igk, Igkj, IGKJRB, IGKJRB1, Igkrsbp, KBF2, RBP, RBP 2N, RBP-JK, RBP-J kappa, RBP-J κ, RBPSUH, Rbpsuh1, Recombination signal binding, recombination signal binding protein for immunoglobulin kappa J region, recombination signal binding protein for immunoglobulin κ J region, SUH
RHO	GTPase Rho, Rho, Rho Family, RHO-GTPASE, Rho-like Gtpase
SHC1	p52SHC, p6, p66, p66s, P66shc, Sh, SHC, Shc (46 kDa isoform), SHCA, SHC adaptor protein 1, Shc p66 isoform, src homology 2 domain-containing transforming protein C1
Smad1/5/8	SMAD1/5/8
Smad1/5/8-	SMAD 1,4,5,8
Smad2/3-	Smad 2/3/4
SMAD4	AW743858, D18Wsu70, D18Wsu70e, DPC, DPC4, JIP, Madh, MADH4, MYHRS, SMAD family member 4, Smaug1
SMAD6	AOVD2, b2b390C, b2b390Clo, HsT17432, Madh, MADH6, MADH7, SMAD family member 6
SMAD7	CRCS3, Madh, MADH7, MADH8, SMAD family member 7
SMO	bnb, CRJS, E130215L21Rik, FZD11, Gx, PHLS, SMOH, Smoothened, smoothened, frizzled class receptor
SRC	ASV, AW259666, BS27, c-SRC, p60-Src, PP60, Pp60/c-Src, pp60c, pp60c-src, Rous sarcoma oncogene, SRC1, SRC proto-oncogene, non-receptor tyrosine kinase, THC6, TVHUSC
STK36	1700112N14RIK, B930045J24, FU, Fuse, Fused, mKIAA1278, serine/threonine kinase 36, Stk36 (predicted)
SUFU	b2b273C, JBTS32, PRO1280, Su, SUFUH, SUFU negative regulator of hedgehog signaling, SUFUXL
SYNGAP1	Gm1963, MRD5, RASA1, RASA5, Sy, Synaptic Ras-GAP 1, synaptic Ras GTPase activating protein 1, synaptic Ras GTPase activating protein 1 homolog (rat), SYNGAP
TAB2	1110030N06Rik, A530078N03Rik, CHTD2, LOC101928709, Map3k, MAP3K7IP2, mKIAA0733, RP1 111D63, TGF-beta activated kinase 1 (MAP3K7) binding protein 2, TGF-beta activated kinase 1/MAP3K7 binding protein 2, TGF-β activated kinase 1 (MAP3K7) binding protein 2, TGF-β activated kinase 1/MAP3K7 binding protein 2
TBID	2700049M22Rik, AI875481, AU022477, BH3 interacting domain death agonist, cBid, FP497
Tgfbeta	Tgfb, TGF-beta 1, 2, and 3, TGF β, TGF-β 1, 2, and 3, transforming growth factor-β
Tgfbetarecept	TGFbetaR, TGFBR, TGFβR, Tgf β receptor
TGFBR1	AAT5, ACVRLK4, AL, Alk, ALK-5, AU017191, ESK2, ESS1, LDS1, LDS1A, LDS2A, LOC103690035, LOC666236, MSSE, SKR4, Tbet, Tbeta, TbetaR-I, TBR-I, TGFbeta1R1, TGF-beta1 receptor, TGF beta I receptor, Tgf-beta-r1, TGF beta receptor type I, TGFbetaRI, Tgfb receptor I, TGFR-1, TGFβ1R1, TGF β I receptor, TGFβ R1, Tgf -β R1/R4, Tgf-β receptor1, TGF β receptor type I, TGFβRI, TGF β type 1 receptors, Tgf β type i receptor, transforming growth factor beta receptor 1, transforming growth factor, beta receptor 1, transforming growth factor, beta receptor I, transforming growth factor β receptor 1, transforming growth factor, β receptor 1, transforming growth factor, β receptor I, transforming growth factor-β receptor type 1, T β R1
TGFBR2	1110020H15Rik, AAT3, AU042018, DNIIR, FAA3, LDS1B, LDS2, LDS2B, MFS2, RIIC, RIIDN, TAAD2, Tbet, Tbeta, TbetaR-II, TBR, TBR-II, TBRLL, TGFbeta1R2, TGF-beta 2, Tgf beta2 receptor, TGFbeta R2, TGF-beta receptor 2, TGF-beta receptor type 2, TGF beta receptor type II, TGFbeta-RII, Tgfbr2T, TGFB receptor II, Tgfbrii, TGFR-2, TGFβ1R2, TGF-β 2, TGFβ R2, TGF-β receptor 2, TGF-β receptor type 2, TGF β receptor type II, TGFβRII, Tgf-β type ii receptor, transforming growth factor beta receptor 2, transforming growth factor, beta receptor 2, transforming growth factor, beta receptor II, transforming growth factor β receptor 2, transforming growth factor, β receptor 2, transforming growth factor, β receptor II, type 2 TGF-β receptors, T β r2
TP53	bbl, BCC7, bfy, bhy, BMFS5, LFS1, p4, p44, p5, P53, P53 cellular tumour antigen, p53 tumor suppressor, transformation related protein 53, TRP53, tumor protein p53, tumour protein p53
Type I BMP	ALK 3,6, ALK 3/6, BMP2 receptor type1, BMP4 receptor type1, Bmpr1, Bmpri
WNT1	BMND16, Int, INT1, OI15, sw, swaying, Wg, wingless-type MMTV integration site family, member 1, Wnt-, Wnt family member 1
WNT5A	8030457G12Rik, hWNT5A, LOC102724616, wingless-type MMTV integration site family, member 5A, Wnt-, Wnt family member 5A
ZBTB17	AA589413, Lp-1, Miz, MIZ-1, mZ13, pHZ-67, Zfp10, Zfp100, zinc finger and BTB domain containing 17, ZNF151, ZNF60

Pathway Analysis Using IPA Software; canonical pathway

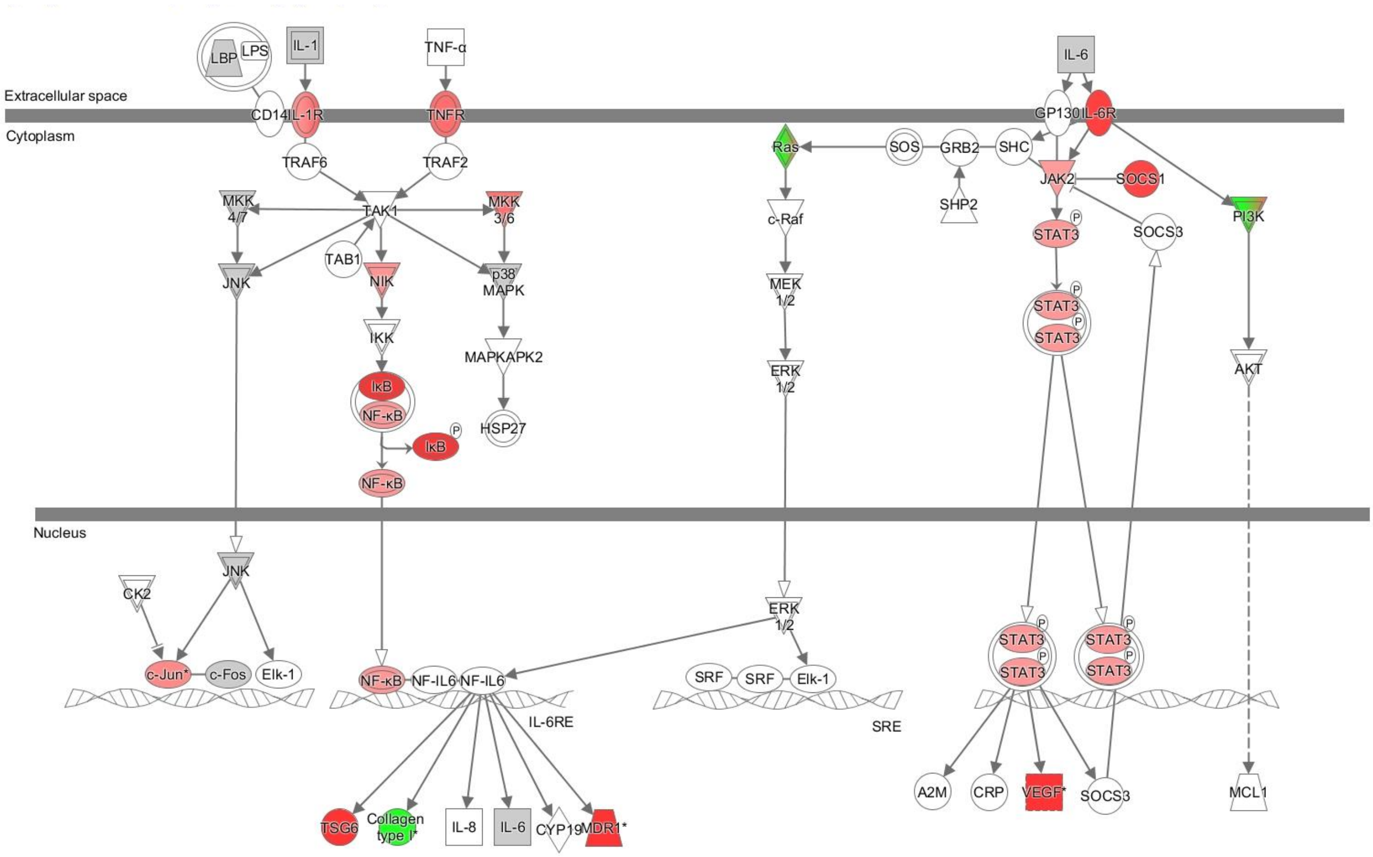

Figure S15. IL-6 Signaling at 6 h

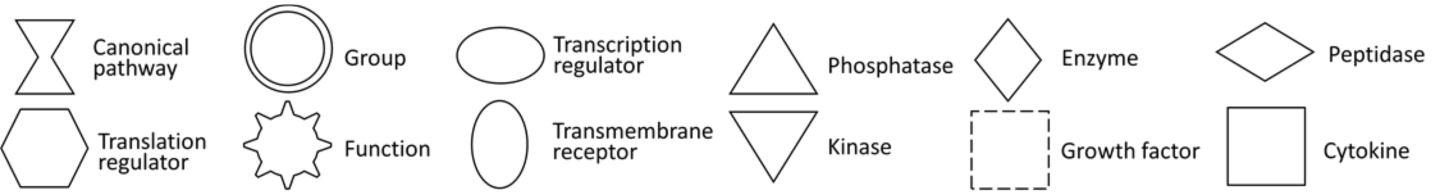

Red: Increased, FDR<0.05 versus solvent control

Green: Decreased, FDR<0.05 versus solvent control

Symbol	Synonym(s)
A2M	A2, A2m1, A2maa, A2MAC1, A2mb, A2MD, A2mp, Alpha2 M, Alpha2 macrofetoprotein, alpha2 MACROGLOBIN, Alpha 2 Macroglobulin, alpha-2-macroglobulin-like, Alpha-2-microglobulin, CPAMD5, FWP007, H-2 Class I Histocompatibility Antigen Alpha-Chain, H-2 Class I Histocompatibility Antigen α-Chain, LOC100911545, Mam, S863-7, α2M, α-2-macroglobin, α-2-macroglobulin, α-2-macroglobulin-like, α-2-microglobulin, α Macroglobulin
ABCB1	ABC20, Abcb1a, Abcb4, ATP-binding cassette, sub-family B (MDR/TAP), member 1A, ATP binding cassette subfamily B member 1, ATP binding cassette subfamily B member 1A, CD243, CLCS, Evi, Evi32, GP170, Mdr, MDR1, Mdr1a, Mdr3, P-, p-170, P-GLYCOPROTEIN, P-GP, Pgy-, PGY1, Pgy-3
AKT	AKT1/2/3, B/Akt, PKB, RAC-PK
CD14	CD14 ANTIGEN, CD14 molecule, lipopolysaccharide (LPS) receptor
CEBPB	Agp/eb, ANF-1, ANF-2, CCAAT enhancer binding protein beta, CCAAT/enhancer binding protein beta, CCAAT/enhancer binding protein (C/EBP), beta, CCAAT/enhancer binding protein (C/EBP), β, CCAAT enhancer-binding protein β, CCAAT/enhancer binding protein β, C/EBPbe, C/EBP-beta, C/Ebp Beta-Lip, C/EBP-β, CEBP-β, C/Ebp β-Lip, CR, CRP2, IL-6, IL-6DBP, NF-, NF-IL6, NF-M, TCF5
Ck2	Casein Kinase II, CKII
COL1A1	Alpha1-1 Collagen, alpha 1(I) COLLAGEN, alpha 1 (I) PROCOLLAGEN, Alpha1 type i collagen, Alpha1 Type I Procollagen, CAFYD, Co, Col, COL1, Col1a, Cola-1, COL I, COLIA1, COLLAGEN 1 alpha1, Collagen alpha1, COLLAGEN alpha1 (I), Collagen alpha 1(I) chain precursor, Collagen I, Collagen i-alpha, COLLAGEN I alpha1, Collagen i-α, Collagen Type1 Alpha1, Collagen type I, COLLAGEN type I alpha 1, collagen, type I, alpha 1, collagen type I alpha 1 chain, Collagen type I pro α 1, COLLAGEN type I α 1, collagen, type I, α 1, collagen type I α 1 chain, Collagen α1(I), Collagen α 1(I) chain precursor, EDSARTH1, EDSC, Mov-, Mov-13, OI1, OI2, OI3, OI4, PREPRO COLLAGEN alpha1(I), PROCOLLAGEN 1alpha1, PROCOLLAGEN 1(I), Procollagen alpha1 (1), PROCOLLAGEN alpha1(I), Procollagen alpha1 type I, PROCOLLAGEN-IALPHA1, Procollagen type 1, Procollagen α 1(i), type1 alpha 1 COLLAGEN, type1 alpha1 PROCOLLAGEN, type1 α 1 COLLAGEN, Type I (alpha 1) procollagen, Type I (α 1) procollagen, α 1(I) COLLAGEN, α 1 (I) PROCOLLAGEN
CRP	Aa1249, Ab1-341, Ab2-196, Ac1-114, Ac1262, Ac2-069, AI255847, Ba2-693, C REACTIVE, C-reactive protein, C-reactive protein, pentraxin-related, PTX1
CXCL8	C-X-C motif chemokine ligand 8, GCP-1, hnIL-8, IL8, LECT, LUCT, LYNAP, MDNCF, MONAP, Monocyte-derived neutrophil chemotactic factor, NAF, NAP-1, SCYB8
CYP19A1	A, Ar, ArKO, ARO, ARO1, Aromatase, CPV1, CYAR, Cyp1, CYP19, Cyp19a, CYP19P1, CYPXIX, cytochrome P450 family 19 subfamily A member 1, cytochrome P450, family 19, subfamily a, polypeptide 1, In, Int, Int-5, LOC100359906, p450, P-450AROM, p450 aromatase
ELK1	ELK, ELK1, member of ETS oncogene family, ETS transcription factor ELK1, p62TCF, RGD:2549, TCF/ELK
ERK1/2	MAPK p44/42, MAPK p44/p42, p42/44 mapk, P42/p44 erk, P42/p44 mapk, p42/p44 MAP KINASE
FOS	AP-1, c-f, C-FOS, D12Rfj, D12Rfj1, FBJ osteosarcoma oncogene, Fos proto-oncogene, AP-1 transcription factor subunit, p55
GRB2	AA408164, ASH, Ash-psi, EGFRBP-GRB2, GRAB2, GRBS, growth factor receptor bound protein 2, MST084, MSTP084, NCKAP2
Hsp27	Heat Shock Protein 27
Ikb	I KAPPA B, Ikbeta, Ikβ, Iκ-B
IkB-NfkB	IkappaB-NFkappaB, IκB-NFκB, NFkB-IkB
IKK	I Kappa B Kinase, IKKALPHABETA, IKK Complex, I κ B Kinase
IL1	Interleukin-1
Il1 receptor	Il1 receptor, Interleukin-1 Receptor
IL6	BSF-2, CDF, FDGI, HGF, HSF, IFN beta 2A, IFN β 2A, IFN-beta-2, IFN-β-2, IFNB2, Il, ILg6, interleukin-6
IL6R	CD126, Gp80, HIES5, I, IL, IL-1Ra, IL6Q, IL6QTL, IL-6R-1, IL-6RA, IL-6R-alpha, Il6 receptor, IL6RQ, IL-6R-α, interleukin 6 receptor, interleukin 6 receptor, alpha, INTERLEUKIN-6 receptors, interleukin 6 receptor, α, Interleukin 6 receptor α chain
IL6ST	5133400A03Rik, AA389424, Ac1055, BB405851, CD130, CDW130, D13Ertd699, D13Ertd699e, Glycoprotein 130, gp13, GP130, HIES4, IL-27R-beta, IL-27R-β, IL-6RB, Il6 transd, interleukin 6 cytokine family signal transducer, Interleukin-6 receptor β chain, interleukin 6 signal transducer, Interleukin 6 signal transduction molecule
JAK2	AI504024, C81284, Fd17, Janus kinase 2, JTK10
Jnk	JNK 54/46, Jnk p56, JNK/SAPK, JUN KINASE, p40, p47, Sapk/Jnk
JUN	Activator protein 1, AP-1, API-1, c-ju, cJUN, Junc, jun proto-oncogene, Jun proto-oncogene, AP-1 transcription factor subunit, LOC100288387, LOC100291417, LOC100293034, p39, v-Jun, V-jun Avian Sarcoma Virus 17 Oncogene Homolog, V-jun Sarcoma Virus 17 Oncogene Homolog
LBP	Bpif, BPIFD2, Lipopolysaccharide binding, lipopolysaccharide binding protein, Ly88
Lbp-lipopolysaccharide	
LPS	C211H376N8O126P6, endotoxin, endotoxin protein, lipopolysaccharides, LPS, TLR4 agonist LPS
MAP2K1/2	MEK1/2, MKK1/2
MAP2K4/7	Jnkk, MEK 4/7, MKK 4/7
MAP3K7	CSCF, FMD2, Map3k7 predicted, MEKK7, mitogen-activated protein kinase kinase kinase 7, TAK1, TGF1a, tgf β activated kinase 1
MAPKAPK2	AA960234, MAPK activated protein kinase 2, Mapkap2, MAPKAP Kinase 2, MAP kinase-activated protein kinase 2, MK-2, Rps6, Rps6kc1
MCL1	AW556805, BCL2L3, EAT, Mcl-, MCL1 apoptosis regulator, BCL2 family member, mcl1/EAT, myeloid cell leukaemia sequence 1, myeloid cell leukemia sequence 1, TM
Mkk3/6	MEK3/6, Mkk3/6 (mitogen activated protein kinase kinase 3/6), MKK3/MKK6
NFkB	NF-KAPPA B, NF-κ B, nuclear factor-κ b, transcription factor nuclear factor κ b
NIK	
p38 MAPK	P38, p38 MAP KINASE, P38 MITOGEN-ACTIVATED protein KINASE
PI3K	1-phosphatidylinositol 3-kinase, 2.7.1.137, ATP:1-phosphatidyl-1D-myo-inositol 3-phosphotransferase, Phosphatidylinositol 3 kinase, phosphatidylinositol 3'-kinase, PI3-kinase, PtdIns 3 Kinase, type III phosphoinositide 3-kinase, type I phosphatidylinositol kinase, Vps34p
PTPN11	2700084A17Rik, AW536184, BPTP3, CFC, JMML, METCDS, MGC14433, Noonan syndrome 1, NS1, protein tyrosine phosphatase non-receptor type 11, protein tyrosine phosphatase, non-receptor type 11, PTP, PTP-1D, PTP2C, S, SAP-2, Sh, SH-P, SHP-2, SH-PTP2, SH-PTP3, Src homology protein 2, SYP
RAF1	6430402F14Rik, AA990557, BB129353, CMD1NN, c-R, Cra, CRAF, Craf1, D830050J10Rik, leukaemiA ONCOGENE HOMOLOG1, LEUKEMIA ONCOGENE HOMOLOG1, NS5, Raf-1 proto-oncogene, serine/threonine kinase, v-, v-Raf, v-raf-leukaemia viral oncogene 1, v-raf-leukemia viral oncogene 1
Ras	
SHC1	p52SHC, p6, p66, p66s, P66shc, Sh, SHC, Shc (46 kDa isoform), SHCA, SHC adaptor protein 1, Shc p66 isoform, src homology 2 domain-containing transforming protein C1
SOCS1	Cis, CIS1, CISH1, Cish7, JA, JAB, JBP, SOC, Sosc1, SS, SSI-1, STAT INDUCED STAT INHIBITOR-1, suppressor of cytokine signaling 1, TIP-3
SOCS3	ATOD4, CI, Cis, CIS3, CISH3, EF-10, SOC, Soc3, Sosc3, SS, SSI-3, Suppressor of cytokine signaling 3
Sos	
SRF	AW049942, AW240594, MCM1, PRTF, RGD1559787, serum response factor, Sfr
STAT3	1110034C02Rik, A, acute-phase response factor, ADMIO, ADMIO1, APRF, AW109958, HIES, MGC16063, signal transducer and activator of transcription 3, Stat3 alpha isoform, Stat3 beta isoform, Stat3 delta, Stat3 α isoform, Stat3 β isoform, Stat3 δ
Stat3-Stat3	STAT3 dimer
TAB1	2310012M03Rik, 3'-Tab1, b2b449C, b2b449Clo, Map3k, MAP3K7IP1, TGF-beta activated kinase 1 (MAP3K7) binding protein 1, TGF-beta activated kinase 1/MAP3K7 binding protein 1, TGF Beta-Activated Kinase-Binding Protein 1, TGF-β activated kinase 1 (MAP3K7) binding protein 1, TGF-β activated kinase 1/MAP3K7 binding protein 1, TGF β-Activated Kinase-Binding Protein 1
TNF	AT-TNF, DI, DIF, RATTNF, TMTNF, Tn, TNF-a, TNF-alpha, Tnfs, Tnfsf1a, TNFSF2, TNF-α, TNLG1F, tumor necrosis factor, Tumor Necrosis Factor α, tumor necrosis factor, α, tumour necrosis factor, tumour Necrosis Factor Alpha, tumour necrosis factor, alpha, tumour Necrosis Factor α, tumour necrosis factor, α
Tnf receptor	member of the tumour necrosis factor receptor family, TNFR, TNF R1, Tnf receptor superfamily, tumour necrosis factor receptor
TNFAIP6	Tnf, TNF alpha induced protein 6, TNFIP6, TNF α induced protein 6, TSG-, TSG-6, tumor necrosis factor alpha induced protein 6, tumor necrosis factor α induced protein 6, tumour necrosis factor alpha induced protein 6, tumour necrosis factor α induced protein 6
TRAF2	AI325259, MGC:45012, RNF117, TNF receptor-associated factor 2, TRAP, TRAP3
TRAF6	2310003F17Rik, AI851288, C630032O20Rik, LOC100042930, LOC100048242, MGC:3310, RNF85, TNF receptor-associated factor 6
VEGFA	Gd-vegf, MVCD1, V, vascular endothelial growth factor A, Veg, VEGF, VEGF111, VEGF12, VEGF16, VEGF18, Vegf-3, VPF

Pathway Analysis Using IPA Software; canonical pathway

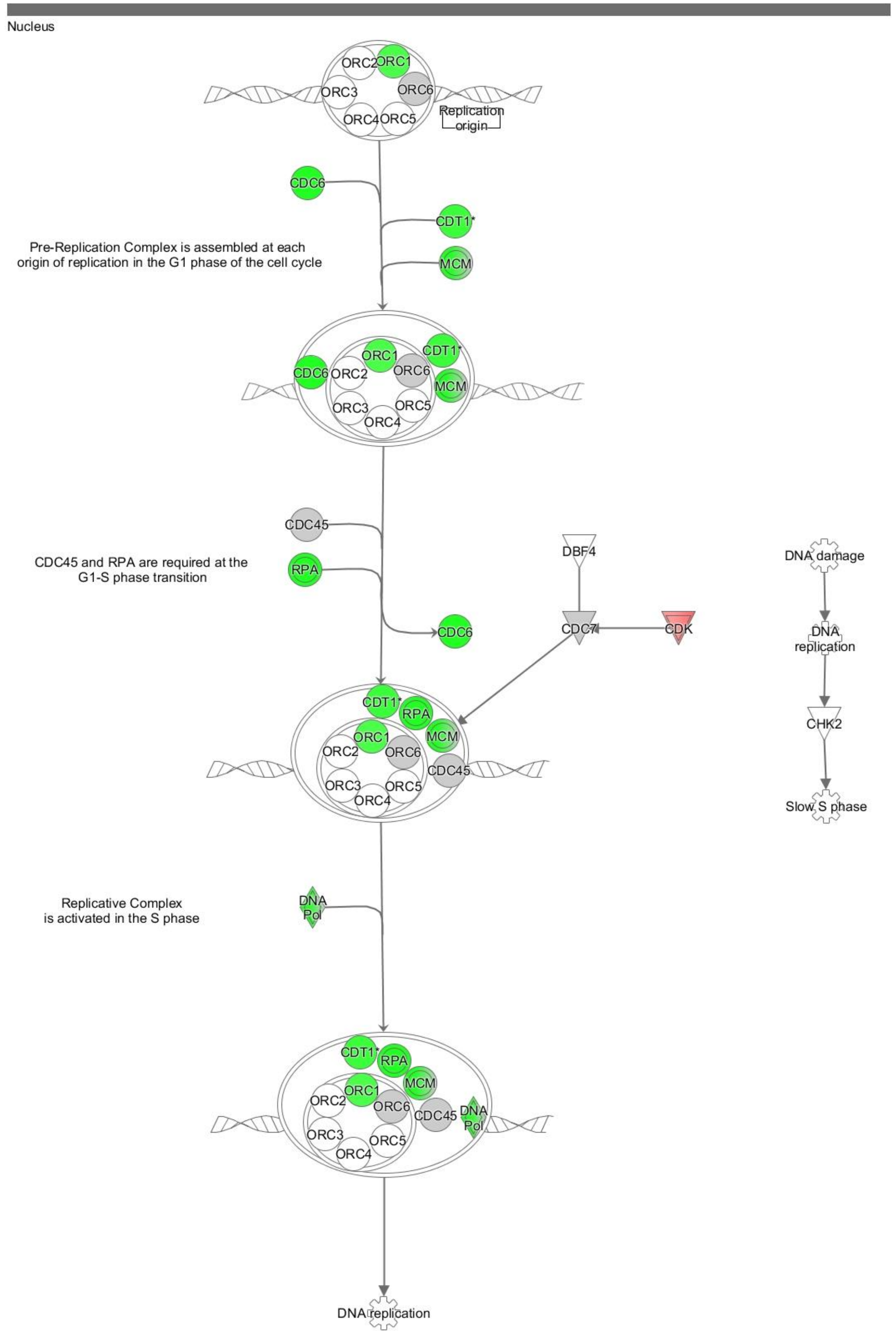

Figure S16. Cell Cycle Control of Chromosomal Replication at 6 h

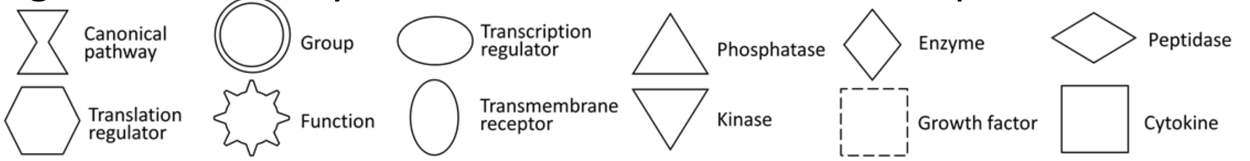

Red: Increased, FDR<0.05 versus solvent control

Green: Decreased, FDR<0.05 versus solvent control

Symbol	Synonym(s)
CDC45	Cdc45i, CDC45L, CDC45L2, cell division cycle 45, LOC287961, MGORS7, PORC-PI-1
CDC6	CDC18, CDC18L, Cdc6-related, cell division cycle 6, HsCDC18, HsCDC6, MGORS5, p62(cdc6)
CDC7	AI597260, Cdc7l, CDC7L1, CDC7-RELATED KINASE, cell division cycle 7, cell division cycle 7 (S. cerevisiae), HsCDC7, Hsk1, huCDC7, muCdc7
CDK	Cdks, cyclin-dependent kinase, Cyclin-Dependent Kinases, G1 CDK
CDT1	2610318F11Rik, AW545653, C76791, chromatin licensing and DNA replication factor 1, DUP, R, RIS2
CHEK2	CDS1, Check2, checkpoint kinase 2, CHK2, hCds1, HUCDS1, LFS2, PP1425, Rad, RAD53
DBF4	A, AA545217, ASK, CHIF, DBF4A, DBF4 zinc finger, LOC100912278, RGD1305854, ZDBF1
DNAPolymerase	DNA Pol
ORC1	AA545195, HSORC1, MmOR, MmORC1, Or, ORC1L, Origin recognition, origin recognition complex subunit 1, origin recognition complex, subunit 1, PARC1
ORC2	AU041563, Or, ORC2L, origin recognition complex subunit 2, origin recognition complex, subunit 2
ORC3	LAT, LATHEO, Or, ORC3L, origin recognition complex subunit 3, origin recognition complex, subunit 3
ORC4	mMmOR, mMmORC4, Or, ORC4L, Orc4l2, ORC4P, origin recognition complex subunit 4, origin recognition complex, subunit 4
ORC5	AL033327, MmOR, MmORC5, Or, ORC5L, ORC5P, origin recognition complex subunit 5, origin recognition complex, subunit 5, PPP1R117
ORC6	6720420I10Rik, Or, ORC6L, origin recognition complex subunit 6, origin recognition complex, subunit 6
Prereplicative Complex	pre-RC

Pathway Analysis Using IPA Software; canonical pathway

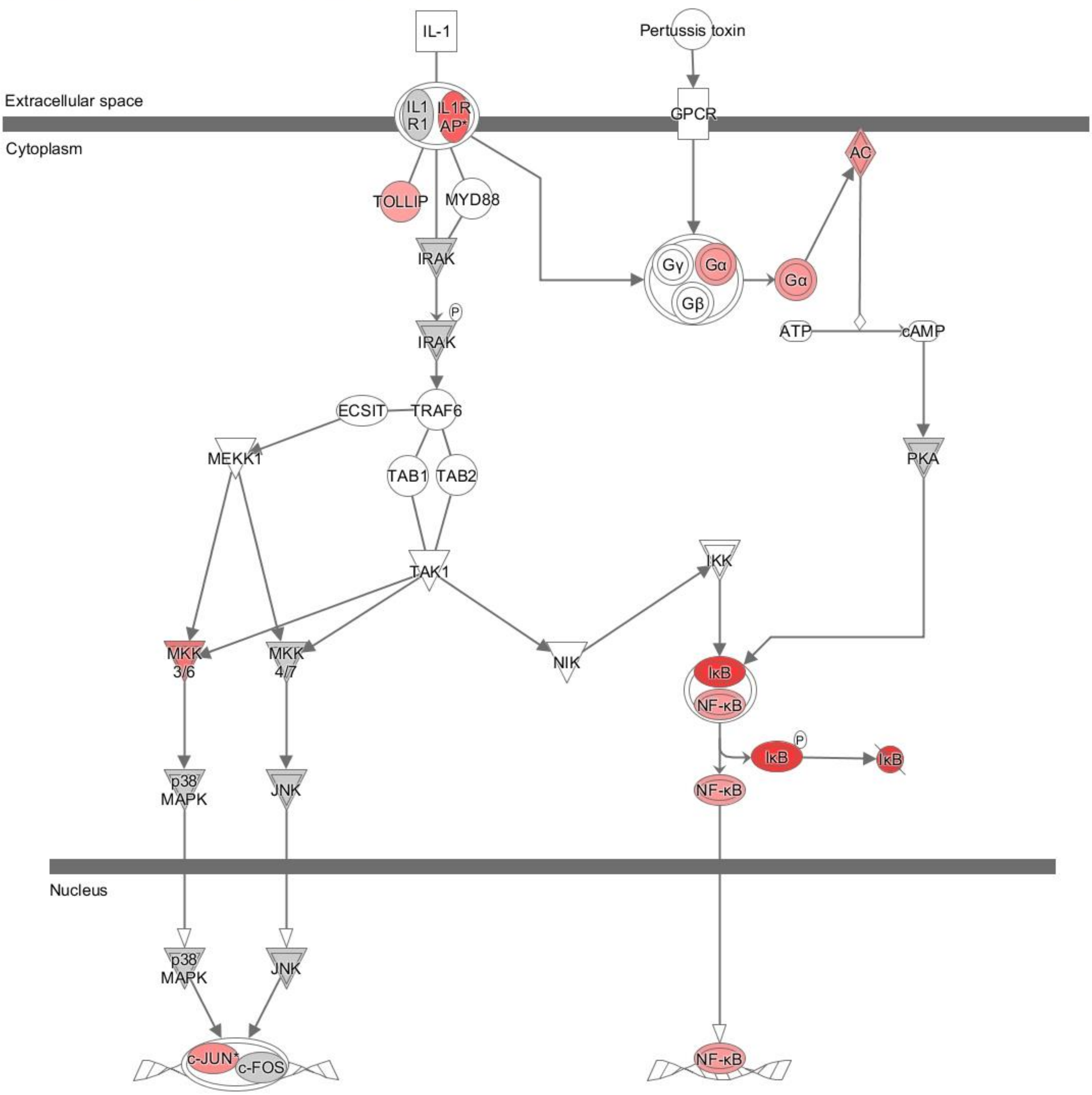

Figure S17. IL-1 Signaling at 6 h

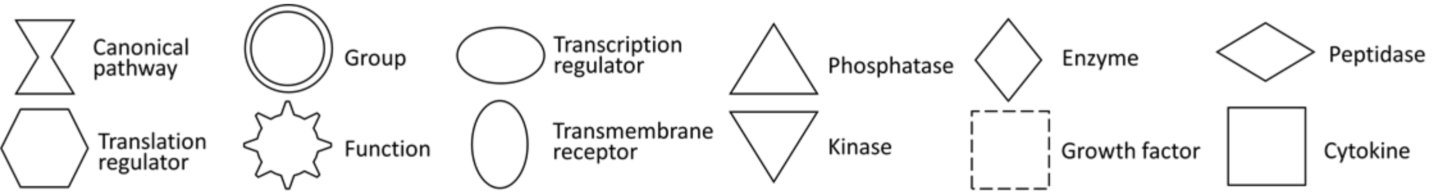

Red: Increased, FDR<0.05 versus solvent control

Green: Decreased, FDR<0.05 versus solvent control

Symbol	Synonym(s)
ADCY	3',5'-cyclic AMP synthetase, 4.6.1.1, AC, Adenyl Cyclase, Adenylate Cyclase, Adenylyl cyclase, ATP diphosphate-lyase (cyclizing), mAC, sAC
Ap1	activator protein-1, c-Jun
ATP	[[(2R,3S,4R,5R)-5-(6-aminopurin-9-yl)-3,4-dihydroxyoxolan-2-yl]methoxy-hydroxyphosphoryl] phosphono hydrogen phosphate, 56-65-5, 9-beta-D-arabinofuranosyladenine 5'-triphosphate, 9-β-D-arabinofuranosyladenine 5'-triphosphate, adenosine 5'-(tetrahydrogen triphosphate), adenosine 5'-triphosphate, ATP, ATP4-, C10H16N5O13P3
cAMP	11002-78-1, 33116-15-3, 3',5'-cyclic AMP, 3',5'-monophosphate, adenosine cyclic, 37839-81-9, (4aR,6R,7R,7aS)-6-(6-aminopurin-9-yl)-2-hydroxy-2-oxo-4a,6,7,7a-tetrahydro-4H-furo[3,2-d][1,3,2]dioxaphosphinin-7-ol, 54532-48-8, 55576-98-2, 60-92-4, 66067-13-8, 68407-13-6, adenosine 3',5'-phosphate, adenosine, cyclic 3',5'-(hydrogen phosphate), adenosine cyclic 3,5 monophosphate, adenosine cyclic 3',5'-monophosphate, adenosine cyclic monophosphate, C10H12N5O6P, cAMP, cyclic-3',5'-monophosphate, adenosine, cyclic adenosine monophosphate, cyclic adenylic acid, cyclic AMP, disodium salt, cyclic AMP, monoammonium salt, cyclic AMP, monopotassium salt, cyclic AMP, monosodium salt, cyclic AMP, sodium salt
ECSIT	ECSIT signaling integrator, ECSIT signalling integrator, Sit, SITPEC
FOS	AP-1, c-f, C-FOS, D12Rfj, D12Rfj1, FBJ osteosarcoma oncogene, Fos proto-oncogene, AP-1 transcription factor subunit, p55
G protein	Galphabetagamma, Galpha-Gbeta-Ggamma, Galphai-Gbeta-Ggamma, Galphaq-Gbeta-Ggamma, Gpro, G protein alpha beta gamma, G protein alpha-G protein beta-GDP-G protein gamma, G protein alpha-G protein beta-G protein gamma, G-protein complex, G protein α-G protein β-GDP-G protein γ, G protein α-G protein β-G protein γ, G-protein α-β-γ, Guanine nucleotide binding protein, Gα-Gβ-Gγ, Gαi-Gβ-Gγ, Gαq-Gβ-Gγ, Gαβγ
G proteinalpha	Galpha, G-Protein Alpha Subunit, G protein α, G-Protein α Subunit, Gα
G proteinbeta	Gbeta, G-protein β, Gβ
G proteingamma	Ggamma, G protein gamma SUBUNITS, G-protein γ, G protein γ SUBUNITS, Gγ
Ikb	I KAPPA B, Ikbeta, Ikβ, Iκ-B
IkB-NfkB	IkappaB-NFkappaB, IκB-NFκB, NFkB-IkB
IKK	I Kappa B Kinase, IKKALPHABETA, IKK Complex, I κ B Kinase
IL1A	IL1, Il, IL-1 α, IL-1F1, IL1-ALPHA, interleukin 1 alpha, interleukin-1 α, Interleukin-A
IL1R1	CD121, CD121A, CD121b, D2S1473, I, IL-, Il1, IL1bRa, IL1R, IL-1RA, IL-1R-alpha, IL-1RI, IL-1R-α, IL-iR, INTERLEUKIN 1 beta receptor, interleukin 1 receptor type 1, interleukin 1 receptor, type I, INTERLEUKIN 1 β receptor, P80
IL1RAP	6430709H04RIK, AI255955, AV239853, C3orf13, IL-, IL1R3, IL-1RAcP, Il1racpb, Il-1 receptor accessory, ILRAP, interleukin 1 receptor accessory protein
JNK	JNK 54/46, Jnk p56, JNK/SAPK, JUN KINASE, p40, p47, Sapk/Jnk
JUN	Activator protein 1, AP-1, API-1, c-ju, cJUN, Junc, jun proto-oncogene, Jun proto-oncogene, AP-1 transcription factor subunit, LOC100288387, LOC100291417, LOC100293034, p39, v-Jun, V-jun Avian Sarcoma Virus 17 Oncogene Homolog, V-jun Sarcoma Virus 17 Oncogene Homolog
MAP2K4/7	Jnkk, MEK 4/7, MKK 4/7
MAP3K1	LOC100912399, MAPK, MAPKKK1, MEKK, MEKK 1, MEK KINASE, MEK KINASE 1, mitogen-activated protein kinase kinase kinase 1, mitogen-activated protein kinase kinase kinase 1-like, Raf, SRXY6
MAP3K14	aly, FTDCR1B, HS, HSNIK, mitogen-activated protein kinase kinase kinase 14, N, NFKB INDUCING KINASE, nf κ b inducing kinase, NIK
MAP3K7	CSCF, FMD2, Map3k7 predicted, MEKK7, mitogen-activated protein kinase kinase kinase 7, TAK1, TGF1a, tgf β activated kinase 1
Mkk3/6	MEK3/6, Mkk3/6 (mitogen activated protein kinase kinase 3/6), MKK3/MKK6
MYD88	IMD68, MYD88D, MYD88 innate immune signal transduction adaptor, MYD88, innate immune signal transduction adaptor, myeloid differentiation primary response gene 88
NFkB	NF-KAPPA B, NF-κ B, nuclear factor-κ b, transcription factor nuclear factor κ b
p38MAPK	P38, p38 MAP KINASE, P38 MITOGEN-ACTIVATED protein KINASE
PKA	A-Kinase, cAMP-Dependent Protein Kinase, cyclic AMP depended protein kinase, protein KINASE A
TAB1	2310012M03Rik, 3'-Tab1, b2b449C, b2b449Clo, Map3k, MAP3K7IP1, TGF-beta activated kinase 1 (MAP3K7) binding protein 1, TGF-beta activated kinase 1/MAP3K7 binding protein 1, TGF Beta-Activated Kinase-Binding Protein 1, TGF-β activated kinase 1 (MAP3K7) binding protein 1, TGF-β activated kinase 1/MAP3K7 binding protein 1, TGF β-Activated Kinase-Binding Protein 1
TAB2	1110030N06Rik, A530078N03Rik, CHTD2, LOC101928709, Map3k, MAP3K7IP2, mKIAA0733, RP1 111D63, TGF-beta activated kinase 1 (MAP3K7) binding protein 2, TGF-beta activated kinase 1/MAP3K7 binding protein 2, TGF-β activated kinase 1 (MAP3K7) binding protein 2, TGF-β activated kinase 1/MAP3K7 binding protein 2
TOLLIP	4930403G24Rik, 4931428G15Rik, IL-1RAcPIP, toll interacting protein
TRAF6	2310003F17Rik, AI851288, C630032O20Rik, LOC100042930, LOC100048242, MGC:3310, RNF85, TNF receptor-associated factor 6

Pathway Analysis Using IPA Software; canonical pathway

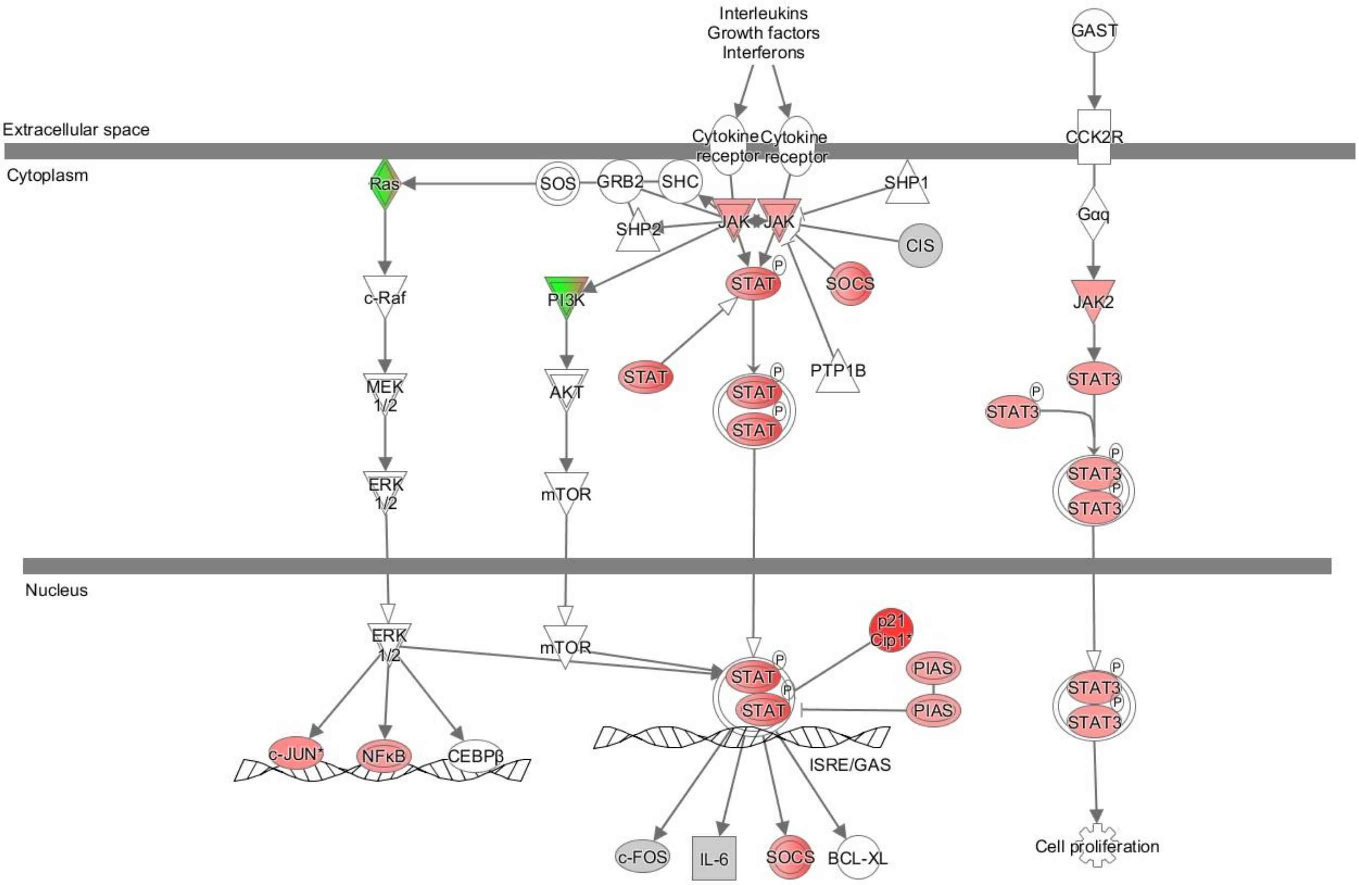

Figure S18. JAK/Stat Signaling at 6 h

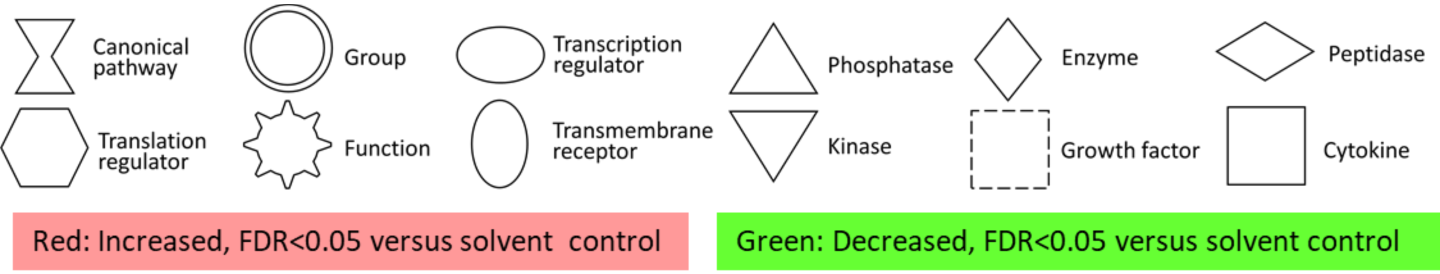

Symbol	Synonym(s)
AKT	AKT1/2/3, B/Akt, PKB, RAC-PK
BCL2L1	bBclxl, Bcl, BCL2L, BCL2-like 1, BCLX, Bcl-X beta, Bclx gamma, BCL-XL/S, Bcl-X β, Bclx γ, PPP1R52
CCKBR	CCK2, CCK2-R, CCK2 receptor, CCK-B, CCKR-, CCKR-2, cholecystokinin B receptor, Cholrec, GASR
CDKN1A	CAP, CAP20, CDK, CDKI, Cdkn, CDKN1, CDKNA1, CI, CIP1, cyclin-dependent kinase inhibitor 1A, cyclin-dependent kinase inhibitor 1A (P21), mda, MDA-6, P2, P21, p21C, p21Cip, p21CIP1, p21W, p21WAF, p21Waf1, PzI Cyclin-Dependent Kinase Inhibitor, SD, SDI1, UV96, Waf, WAF1
CEBPB	Agp/eb, ANF-1, ANF-2, CCAAT enhancer binding protein beta, CCAAT/enhancer binding protein beta, CCAAT/enhancer binding protein (C/EBP), beta, CCAAT/enhancer binding protein (C/EBP), β, CCAAT enhancer-binding protein β, CCAAT/enhancer binding protein β, C/EBPbe, C/EBP-beta, C/Ebp Beta-Lip, C/EBP-β, CEBP-β, C/Ebp β-Lip, CR, CRP2, IL-6, IL-6DBP, NF-, NF-IL6, NF-M, TCF5
CISH	BACTS2, C, CI, CIS, CIS-1, cytokine inducible SH2-containing protein, F17, F23, G18, SOCS
ERK1/2	MAPK p44/42, MAPK p44/p42, p42/44 mapk, P42/p44 erk, P42/p44 mapk, p42/p44 MAP KINASE
FOS	AP-1, c-f, C-FOS, D12Rfj, D12Rfj1, FBJ osteosarcoma oncogene, Fos proto-oncogene, AP-1 transcription factor subunit, p55
GAST	G, GAS, GASTRIN, Gastrin-17, PPG34
GNAQ	1110005L02Rik, 6230401I02Rik, AA408290, AW060788, CMC1, DKFZp686D0521, Dsk, Dsk1, Dsk10, Gal, G-ALPHA-q, GAQ, G protein alpha Q, G protein alpha Q/11, G protein subunit alpha q, G protein subunit α q, G protein α Q, G protein α Q/11, Gq, Gqalpha, GqI, Gq protein alpha subunit, Gq protein α subunit, Gqα, guanine nucleotide binding protein, alpha q polypeptide, guanine nucleotide binding protein, α q polypeptide, G-α-q, Pst receptor, SWS
GRB2	AA408164, ASH, Ash-psi, EGFRBP-GRB2, GRAB2, GRBS, growth factor receptor bound protein 2, MST084, MSTP084, NCKAP2
IL6	BSF-2, CDF, FDGI, HGF, HSF, IFNB2, IFN-beta-2, IFN beta 2A, IFN-β-2, IFN β 2A, Il, ILg6, interleukin-6
JAK	JAK kinase
JAK2	AI504024, C81284, Fd17, Janus kinase 2, JTK10
Jun	Activator protein 1, AP-1, API-1, c-ju, cJUN, Junc, jun proto-oncogene, Jun proto-oncogene, AP-1 transcription factor subunit, LOC100288387, LOC100291417, LOC100293034, p39, v-Jun, V-jun Avian Sarcoma Virus 17 Oncogene Homolog, V-jun Sarcoma Virus 17 Oncogene Homolog
MAP2K1/2	MEK1/2, MKK1/2
MTOR	2610315D21Rik, AI327068, fl, Flat, Fr, FRAP, FRAP1, FRAP2, FRB, mechanistic target of rapamycin kinase, RA, RAF, RAFT1, RAPT1, RRAFT1, SKS
NFKB1	NF-KAPPA B, NF-κ B, nuclear factor-κ b, transcription factor nuclear factor κ b
PI3K	1-phosphatidylinositol 3-kinase, 2.7.1.137, ATP:1-phosphatidyl-1D-myo-inositol 3-phosphotransferase, Phosphatidylinositol 3 kinase, phosphatidylinositol 3'-kinase, PI3-kinase, PtdIns 3 Kinase, type III phosphoinositide 3-kinase, type I phosphatidylinositol kinase, Vps34p
PTPN1	protein-TYROSINE PHOSPHATASE, protein tyrosine phosphatase non-receptor type 1, protein tyrosine phosphatase, non-receptor type 1, PTP-, PTP-1B, PTP1B alpha, PTP1BB, PTP1B α, Ptpase 1, PTPASE 1B, PTP-HA2
PTPN11	2700084A17Rik, AW536184, BPTP3, CFC, JMML, METCDS, MGC14433, Noonan syndrome 1, NS1, protein tyrosine phosphatase non-receptor type 11, protein tyrosine phosphatase, non-receptor type 11, PTP, PTP-1D, PTP2C, S, SAP-2, Sh, SH-P, SHP-2, SH-PTP2, SH-PTP3, Src homology protein 2, SYP
PTPN6	70Z-SHP, hc, HCP, HCPH, HPTP1C, me, protein tyrosine phosphatase non-receptor type 6, protein tyrosine phosphatase, non-receptor type 6, Ptp, PTP-1C, Ptph6, PTPTY-42, SHP, SHP-1, SHP-1L, SH-PTP1, Sr Homology Protein 1 Phosphatase
RAF1	6430402F14Rik, AA990557, BB129353, CMD1NN, c-R, Cra, CRAF, Craf1, D830050J10Rik, leukaemiA ONCOGENE HOMOLOG1, LEUKEMIA ONCOGENE HOMOLOG1, NS5, Raf-1 proto-oncogene, serine/threonine kinase, v-, v-Raf, v-raf-leukaemia viral oncogene 1, v-raf-leukemia viral oncogene 1
SHC1	p52SHC, p6, p66, p66s, P66shc, Sh, SHC, Shc (46 kDa isoform), SHCA, SHC adaptor protein 1, Shc p66 isoform, src homology 2 domain-containing transforming protein C1
SOCS	JAB
STAT3	1110034C02Rik, A, acute-phase response factor, ADMIO, ADMIO1, APRF, AW109958, HIES, MGC16063, signal transducer and activator of transcription 3, Stat3 alpha isoform, Stat3 beta isoform, Stat3 delta, Stat3 α isoform, Stat3 β isoform, Stat3 δ
Stat3-Stat3	STAT3 dimer

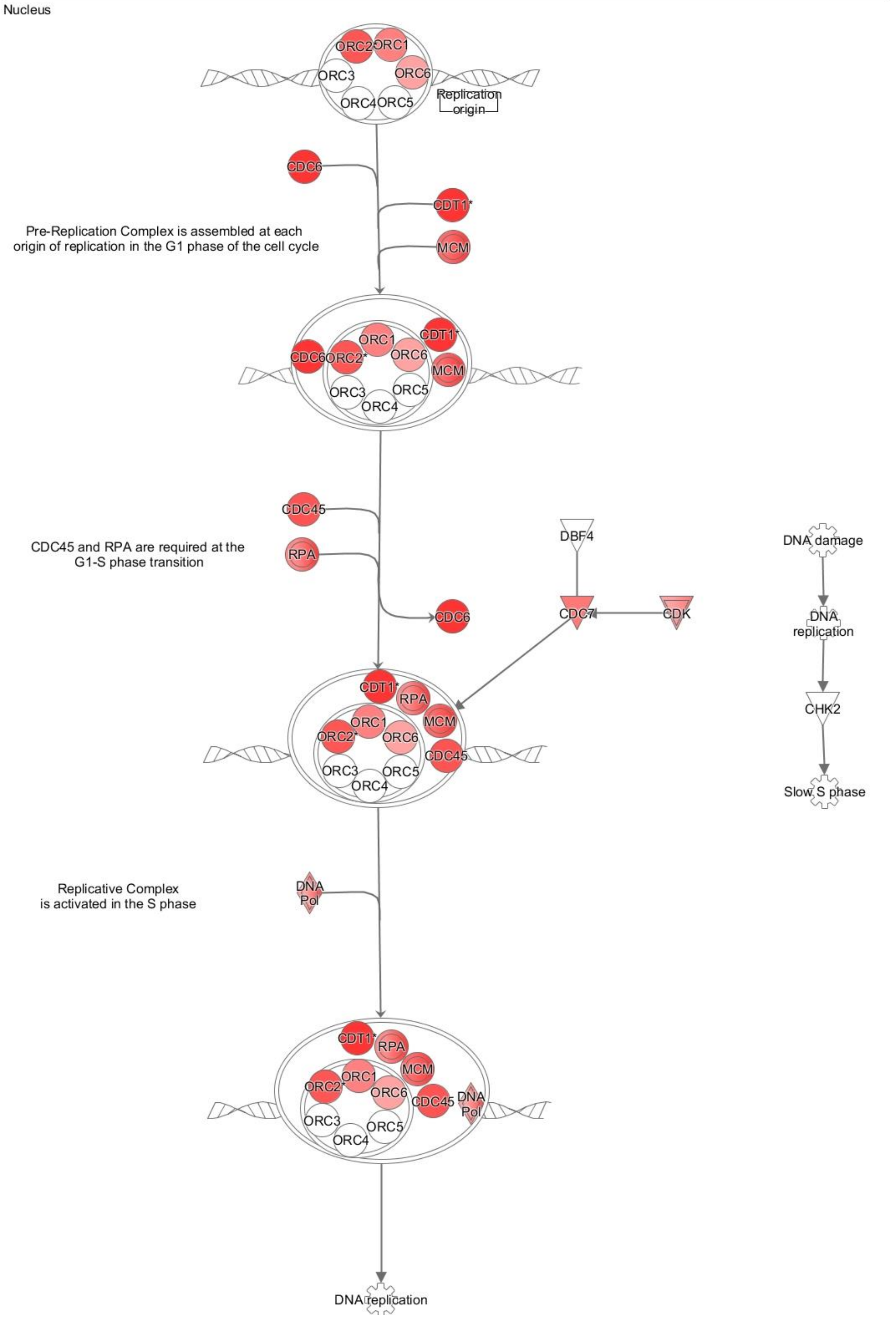

Figure S19. Cell Cycle Control of Chromosomal Replication at 24 h

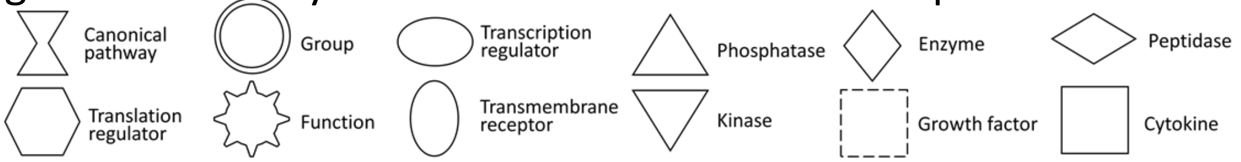

Red: Increased, FDR<0.05 versus solvent control

Green: Decreased, FDR<0.05 versus solvent control

Symbol	Synonym(s)
CDC45	Cdc45i, CDC45L, CDC45L2, cell division cycle 45, LOC287961, MGORS7, PORC-PI-1
CDC6	CDC18, CDC18L, Cdc6-related, cell division cycle 6, HsCDC18, HsCDC6, MGORS5, p62(cdc6)
CDC7	AI597260, Cdc7l, CDC7L1, CDC7-RELATED KINASE, cell division cycle 7, cell division cycle 7 (S. cerevisiae), HsCDC7, Hsk1, huCDC7, muCdc7
CDK	Cdks, cyclin-dependent kinase, Cyclin-Dependent Kinases, G1 CDK
CDT1	2610318F11Rik, AW545653, C76791, chromatin licensing and DNA replication factor 1, DUP, R, RIS2
CHEK2	CDS1, Check2, checkpoint kinase 2, CHK2, hCds1, HUCDS1, LFS2, PP1425, Rad, RAD53
DBF4	A, AA545217, ASK, CHIF, DBF4A, DBF4 zinc finger, LOC100912278, RGD1305854, ZDBF1
DNAPolymerase	DNA Pol
ORC1	AA545195, HSORC1, MmOR, MmORC1, Or, ORC1L, Origin recognition, origin recognition complex subunit 1, origin recognition complex, subunit 1, PARC1
ORC2	AU041563, Or, ORC2L, origin recognition complex subunit 2, origin recognition complex, subunit 2
ORC3	LAT, LATHEO, Or, ORC3L, origin recognition complex subunit 3, origin recognition complex, subunit 3
ORC4	mMmOR, mMmORC4, Or, ORC4L, Orc4l2, ORC4P, origin recognition complex subunit 4, origin recognition complex, subunit 4
ORC5	AL033327, MmOR, MmORC5, Or, ORC5L, ORC5P, origin recognition complex subunit 5, origin recognition complex, subunit 5, PPP1R117
ORC6	6720420I10Rik, Or, ORC6L, origin recognition complex subunit 6, origin recognition complex, subunit 6
Prereplicative Complex	pre-RC

Pathway Analysis Using IPA Software; canonical pathway

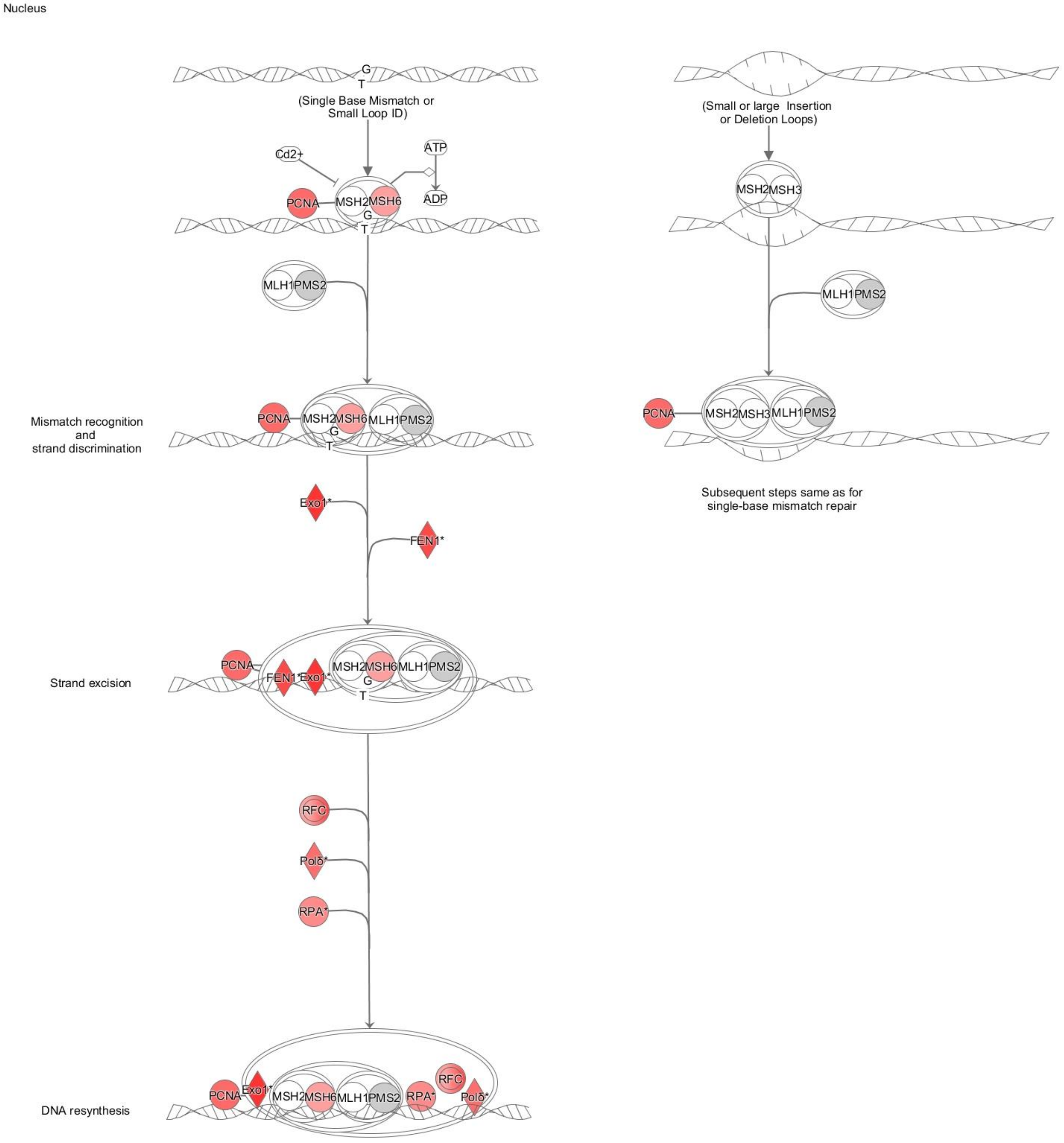

Figure S20. Mismatch Repair in Eukaryotes at 24 h

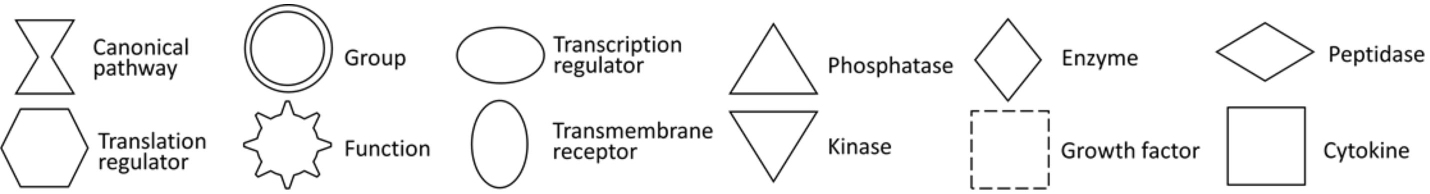

Red: Increased, FDR<0.05 versus solvent control

Green: Decreased, FDR<0.05 versus solvent control

Symbol	Synonym(s)
ADP	20398-34-9, [(2R,3S,4R,5R)-5-(6-aminopurin-9-yl)-3,4-dihydroxyoxolan-2-yl]methyl phosphono hydrogen phosphate, 58-64-0, 9-beta-D-arabinofuranosyladenine 5'-diphosphate, 9-β-D-arabinofuranosyladenine 5'-diphosphate, adenosine 5'-(trihydrogen diphosphate), adenosine diphosphate, C10H15N5O10P2
ATP	[[(2R,3S,4R,5R)-5-(6-aminopurin-9-yl)-3,4-dihydroxyoxolan-2-yl]methoxy-hydroxyphosphoryl] phosphono hydrogen phosphate, 56-65-5, 9-beta-D-arabinofuranosyladenine 5'-triphosphate, 9-β-D-arabinofuranosyladenine 5'-triphosphate, adenosine 5'-(tetrahydrogen triphosphate), adenosine 5'-triphosphate, ATP, ATP4-, C10H16N5O13P3
Cd2+	22537-48-0, cadmium(2+), cadmium acetate, cadmium cation, cadmium ion, cadmium, ion (Cd2+), Cd+2
EXO1	5730442G03RIK, exonuclease 1, HEX1, hExoI, M, Msa
FEN1	AW538437, flap structure-specific endonuclease 1, MF1, RAD2
MLH1	1110035C23Rik, AI317206, AI325952, AI561766, COCA2, FCC2, hMLH1, HNPCC, HNPCC2, MMRCS1, mutL homolog 1
MSH2	AI788990, COCA1, FCC1, hMSH2, HNPCC, HNPCC1, LCFS2, MMRCS2, mutS homolog 2
MSH3	D13Em1, DUP, FAP4, LOC100046843, MRP1, mutS homolog 3, Re, Rep, Rep-3
MSH6	AU044881, AW550279, GTB, GTBP, Gtmb, GTMBP, hMSH6, HNPCC5, HSAP, MMRCS3, Msh, mutS homolog 6, p160
MutLalpha	MLH1-PMS2, MutL α
MutLα-MutSα	MutLalpha-MutSalpha
MutLα-MutSα-Exo1-FEN1	MutLalpha-MutSalpha-Exo1-FEN1
MutLα-MutSα-Exo1-Polδ-RFC-RPA	MutLalpha-MutSalpha-Exo1-Poldelta-RFC-RPA
MutLα-MutSβ	MutLalpha-MutSbeta
MutSalpha	hMutS alpha, hMutS α, MSH2-MSH6, MutS α
MutSbeta	MutS β
PCNA	ATLD2, Pcna/cyclin, PCNAR, proliferating cell nuclear antigen
PMS2	AW555130, HNPCC4, LOC115486439, MLH4, MMRCS4, PMS1 homolog 2, mismatch repair system component, PMS2CL, PMSL2, Rsph10b
POLD1	CDC2, CRCS10, DNA POLYD, DNA POLYMERASE delta, DNA polymerase delta 1, catalytic subunit, DNA POLYMERASE δ, DNA polymerase δ 1, catalytic subunit, MDPL, POLD, polymerase (DNA directed), delta 1, catalytic subunit, polymerase (DNA directed), δ 1, catalytic subunit, Polδ
RPA1	5031405K23Rik, AA589576, AW557552, Cb1-727, HSSB, MST075, p70Rpa1, R, REPA1, replication protein A1, RF, RF-A, RP-A, RPA70

Pathway Analysis Using IPA Software; canonical pathway

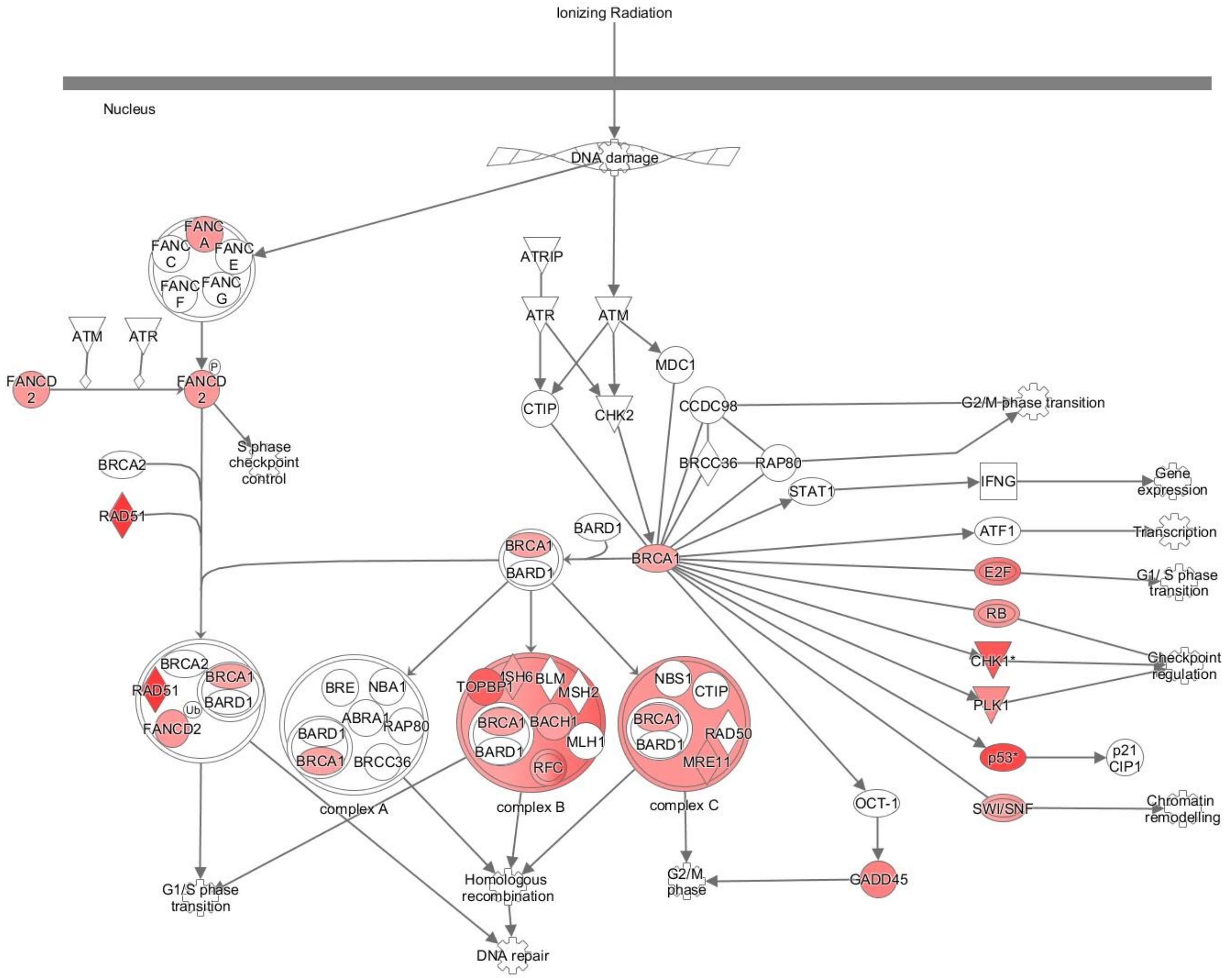

Figure S21. Role of BRCA1 in DNA Damage Response at 24 h

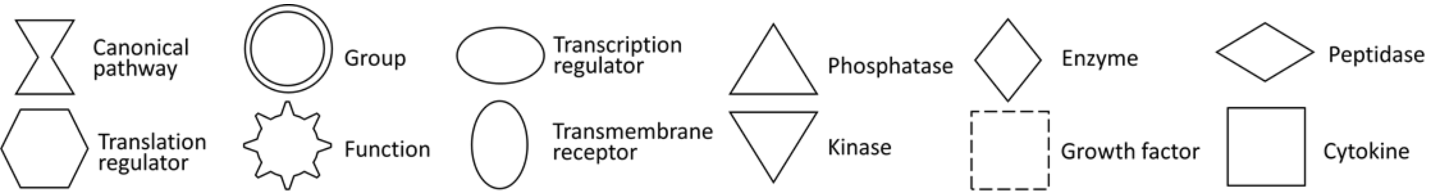

Red: Increased, FDR<0.05 versus solvent control

Green: Decreased, FDR<0.05 versus solvent control

Symbol	Synonym(s)
ABRAXAS1	3830405G04RIK, 5630400M01RIK, ABRA1, abraxas 1, BRCA1 A complex subunit, AI506069, AL024423, AV118690, BRCA1 A complex subunit, CCDC98, Fam17, FAM175A, FLJ13614, RGD1305287
ATF1	activating transcription factor 1, activating transcription factor 1, pseudogene, Atf1-ps, Gm1862, LOC100047705, TREB36
ATM	AI256621, AT1, ATA, ataxia telangiectasia mutated, ATC, ATD, ATDC, ATE, ATM serine/threonine kinase, C030026E19RIK, TEL1, TELO1
ATR	ataxia telangiectasia and Rad3 related, Ataxia-telangiectasia-like, ATR serine/threonine kinase, FCTCS, FRP1, LOC100365674, LOC367198, LOC684113, MEC1, SCKL, SCKL1
ATRIP	6620401K05RIK, ATR interacting protein
BABAM1	5430437P03Rik, BRISC and BRCA1 A complex member 1, C19orf62, FLJ20571, HSPC142, MERIT40, NBA1
BABAM2	6030405P19Rik, AI429776, B830038C02Rik, Br, BRCC4, BRCC45, BRE, BRISC and BRCA1 A complex member 2
BARD1	BRAD1, BRCA1 associated RING domain 1, ENSMUSG00000060893, ENSMUSG00000073653
BLM	BLM RecQ like helicase, Blooms syndrome, Bloom syndrome, Bloom syndrome, RecQ like helicase, BS, MGRISCE1, RECQ2, RECQL2, RECQL3
BRCA1	BRCA1 DNA repair associated, BRCA1, DNA repair associated, BRCAI, BRCC1, breast cancer 1, early onset, BROVCA1, FANCS, PNCA4, PPP1R53, PSCP, RNF53
BRCA2	AW045498, BRCA2 DNA repair associated, BRCA2, DNA repair associated, BRCC2, breast cancer 2, early onset, BROVCA2, FACD, FAD, FAD1, Fanc, FANCD, FANCD1, GLM3, PNCA2, RAB1, RAB163, XRCC11
BRCC3	B36, BRCA1/BRCA2-containing complex subunit 3, BRCA1/BRCA2-containing complex, subunit 3, BRCC36, C6.1, C6.1A, CXorf53
BRIP1	3110009N10Rik, 8030460J03Rik, BACH, BACH1, BRCA1 interacting protein C-terminal helicase 1, FACJ, FANCJ, OF
CDKN1A	CAP, CAP20, CDK, CDKI, Cdkn, CDKN1, CDKNA1, CI, CIP1, cyclin-dependent kinase inhibitor 1A, cyclin-dependent kinase inhibitor 1A (P21), mda, MDA-6, P2, P21, p21C, p21Cip, p21CIP1, p21W, p21WAF, p21Waf1, PzI Cyclin-Dependent Kinase Inhibitor, SD, SDI1, UV96, Waf, WAF1
CHEK1	C85740, checkpoint kinase 1, CHK1, rad27
CHEK2	CDS1, Check2, checkpoint kinase 2, CHK2, hCds1, HUCDS1, LFS2, PP1425, Rad, RAD53
FA	FA Core Complex
FANCA	AW208693, FA, FA1, FAA, FACA, FA complementation group A, FA-H, FANCH, Fanconi anaemia, complementation group A, Fanconi anemia, complementation group A
FANCC	FA3, FAC, FACC, FA complementation group C, Fanconi anaemia, complementation group C, Fanconi anemia, complementation group C
FANCD2	2410150O07RIK, AU015151, BB137857, FA4, FACD, FA complementation group D2, FAD, FA-D2, FANCD, Fanconi anaemia, complementation group D2, Fanconi anemia, complementation group D2
FANCE	2810451D06RIK, AI415634, AW209126, FACE, FA complementation group E, FAE, Fanconi anaemia, complementation group E, Fanconi anemia, complementation group E, RGD1561045
FANCF	A730016A17, FA complementation group F, FAF, Fanconi anaemia, complementation group F, Fanconi anemia, complementation group F, RGD1561456
FANCG	AU041407, FA complementation group G, FAG, Fanconi anaemia, complementation group G, Fanconi anemia, complementation group G, LOC684204, Xrcc, XRCC9
GADD45A	AA545191, Ddit, DDIT1, Gadd, GADD45, GADD45 alpha, GADD45α, growth arrest and DNA-damage-inducible 45 alpha, growth arrest and DNA-damage-inducible 45 α, Growth arrest and DNA-damage-inducible 45, α, growth arrest and DNA damage inducible alpha, growth arrest and DNA-damage-inducible, alpha, growth arrest and DNA damage inducible α, growth arrest and DNA-damage-inducible, α
IFNG	If, If2f, IFG, IFI, IFN-2, IFNG2, IFN gamma, IFN-II, IFN type II, IFN-γ, IMD69, INF-γ, Interferon gamma, Interferon γ, type II INTERFERON, γ-ifn, γ interferon
MDC1	6820401C03, AA413496, mediator of DNA damage checkpoint 1, mKIAA0170, NFBD, NFBD1
MLH1	1110035C23Rik, AI317206, AI325952, AI561766, COCA2, FCC2, hMLH1, HNPCC, HNPCC2, MMRCS1, mutL homolog 1
MRE11	ATLD, HNGS1, MRE11A, MRE11A homolog A, double strand break repair nuclease, MRE11B, MRE11 homolog, double strand break repair nuclease
MSH2	AI788990, COCA1, FCC1, hMSH2, HNPCC, HNPCC1, LCFS2, MMRCS2, mutS homolog 2
MSH6	AU044881, AW550279, GTB, GTBP, Gtmb, GTMBP, hMSH6, HNPCC5, HSAP, MMRCS3, Msh, mutS homolog 6, p160
NBN	ATV, AT-V1, AT-V2, Nb, NBS, NBS1, NIBRIN, P95
PLK1	P, PLK, polo-like kinase 1, STPK, STPK13
POU2F1	2810482H01Rik, LOC100503933, NF-A1, Oct-, OCT1, oct-1B, Otf-, OTF1, POU class 2 homeobox 1, POU domain, class 2, transcription factor 1
RAD50	hRad50, Mr, Mrell, NBSLD, Rad, RAD502, RAD50 double strand break repair protein, Rad50l
RAD51	AV304093, BRCC5, FANCR, HRAD51, HsRad51, HsT16930, MRMV2, Rad, RAD51A, RAD51 recombinase, Re, RECA, RECA-LIKE, RGD1563603
RB	pRb, Rb Tumor Suppressor, Rb tumour Suppressor
RBBP8	9930104E21RIK, COM1, Ct, CTIP, JWDS, RB binding protein 8, endonuclease, Rbbp8-rs, retinoblastoma binding protein 8, endonuclease, RGD1308872, RIM, SAE2, SCKL2
STAT1	2010005J02Rik, AA408197, CANDF7, DD6G4-4, IMD31A, IMD31B, IMD31C, ISGF-3, p91, signal transducer and activator of transcription 1, STAT1 alpha, Stat1 beta, Stat1 p91, STAT1 α, Stat1 β, STAT91, TRANSCRIPTION FACTOR SIGNAL TRANSDUCER and ACTIVATOR
Swi-Snf	SWI/SNF
TOPBP1	1110031N14RIK, 2810429C13Rik, AI256758, D430026L04Rik, DNA topoisomerase II binding protein 1, Dpb11, mKIAA0259, RGD1562949, TOP2BP1, TOPO2 BP, TOPOISOMERASE2 binding, topoisomerase (DNA) II binding protein 1
TP53	bbl, BCC7, bfy, bhy, BMFS5, LFS1, p4, p44, p5, P53, P53 cellular tumour antigen, p53 tumor suppressor, transformation related protein 53, TRP53, tumor protein p53, tumour protein p53
UIMC1	9430016E08Rik, D330018D10Rik, D630032M02Rik, RAP80, RETINOID X receptor INTERACTING, RGD1307009, RIP110, Rxri, Rxrip110, ubiquitin interaction motif containing 1, X2HRIP110

Pathway Analysis Using IPA Software; canonical pathway

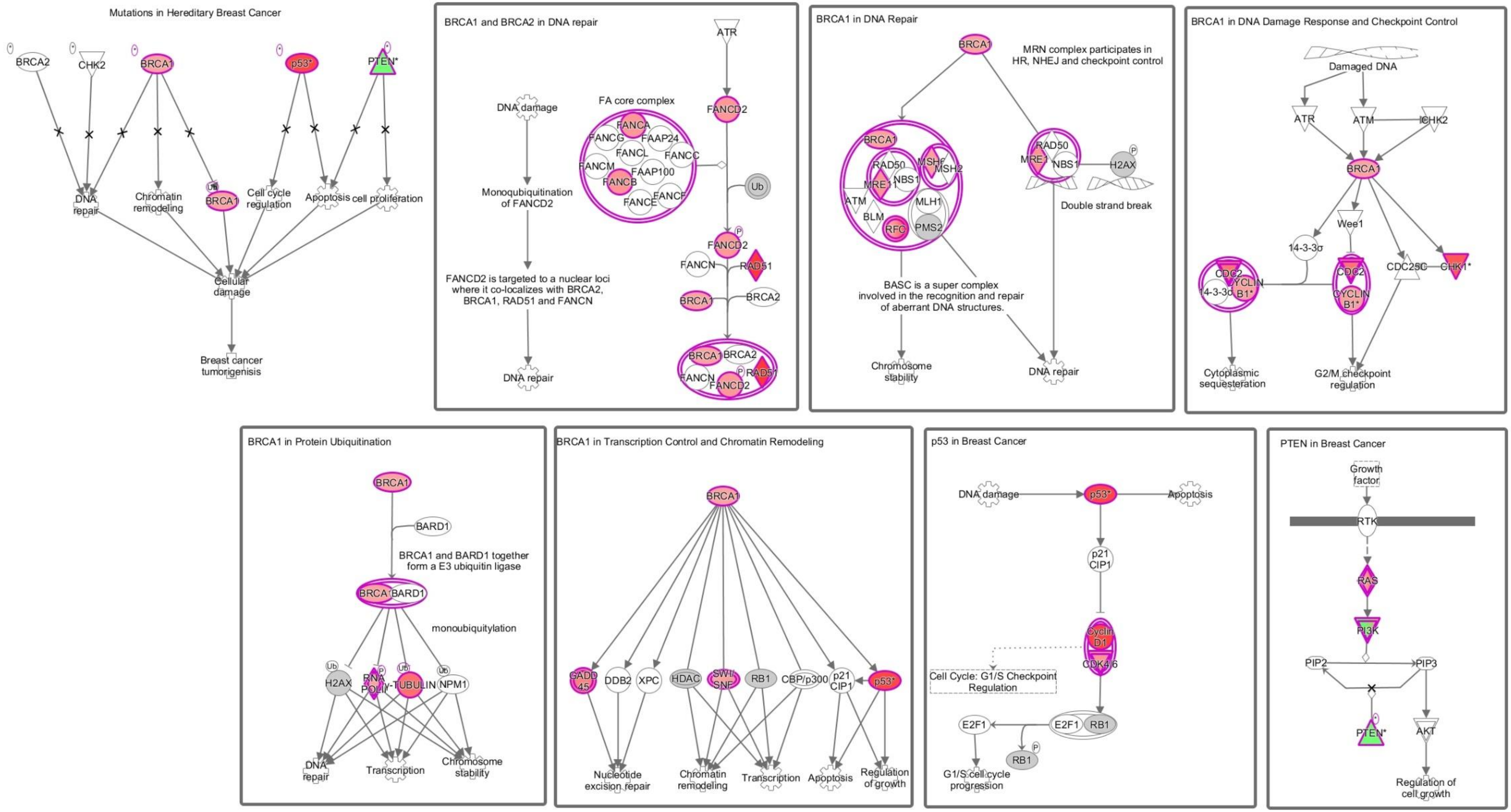

Figure S22. Hereditary Breast Cancer Signaling at 24 h

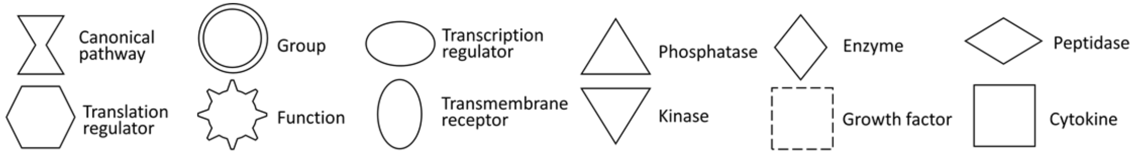

Red: Increased, FDR<0.05 versus control

Green: Decreased, FDR<0.05 versus control

Symbol	Synonym(s)
14-3-3\u03C3-CyclinB-CDC2	14-3-3sigma-Cyclin B-CDC2
AKT	AKT1/2/3, B/Akt, PKB, RAC-PK
ATM	AI256621, AT1, ATA, ataxia telangiectasia mutated, ATC, ATD, ATDC, ATE, ATM serine/threonine kinase, C030026E19RIK, TEL1, TELO1
ATR	ataxia telangiectasia and Rad3 related, Ataxia-telangiectasia-like, ATR serine/threonine kinase, FCTCS, FRP1, LOC100365674, LOC367198, LOC684113, MEC1, SCKL, SCKL1
BARD1	BRAD1, BRCA1 associated RING domain 1, ENSMUSG00000060893, ENSMUSG00000073653
BLM	BLM RecQ like helicase, Blooms syndrome, Bloom syndrome, Bloom syndrome, RecQ like helicase, BS, MGRISCE1, RECQ2, RECQL2, RECQL3
BRCA1	BRCA1 DNA repair associated, BRCA1, DNA repair associated, BRCAI, BRCC1, breast cancer 1, early onset, BROVCA1, FANCS, PNCA4, PPP1R53, PSCP, RNF53
BRCA2	AW045498, BRCA2 DNA repair associated, BRCA2, DNA repair associated, BRCC2, breast cancer 2, early onset, BROVCA2, FACD, FAD, FAD1, Fanc, FANCD, FANCD1, GLM3, PNCA2, RAB1, RAB163, XRCC11
CBP/p300	CBP, CBP-p300
CCNB1	CCNB, Ccnb1-ps, Ccnb1-r, CCNB1-RS1, Ccnb1-rs13, CycB1, Cycb1-rs1, Cycb-4, Cycb-5, CYCLIN B, CYCLIN B1, cyclin B1, pseudogene, Cyclin b4, EG434175, Gm5593
CCND1	AI327039, B-CELL CLL/LYMPHOMA 1, bcl-, BCL1, cD1, CycD1, CYCLIN D1, Cyl-, Cyl-1, D11S287E, G1/S-Specific Cyclin D1, PR, PRAD1, U21B31
CDC25C	CDC25, cdc25c-64, cell division cycle 25C, PPP1R60
CDKN1A	CAP, CAP20, CDK, CDKI, Cdkn, CDKN1, CDKNA1, CI, CIP1, cyclin-dependent kinase inhibitor 1A, cyclin-dependent kinase inhibitor 1A (P21), mda, MDA-6, P2, P21, p21C, p21Cip, p21CIP1, p21W, p21WAF, p21Waf1, PzI Cyclin-Dependent Kinase Inhibitor, SD, SDI1, UV96, Waf, WAF1
CHEK1	C85740, checkpoint kinase 1, CHK1, rad27
CHEK2	CDS1, Check2, checkpoint kinase 2, CHK2, hCds1, HUCDS1, LFS2, PP1425, Rad, RAD53
Cyclinb1/Cdc2	Cyclin B1-Cdc2
DDB2	2610043A19Rik, damage specific DNA binding protein 2, DDBB, p4, UV-DDB2, XPE
E2F1	E2f, E2F transcription factor 1, mKIAA4009, RBAP1, RBBP3, RBP3, Tg(Wnt1-cre)2Sor
FA	FA Core Complex
FAAP100	2310003H01Rik, C17orf70, FA core complex associated protein 100, Fanconi anaemia core complex associated protein 100, Fanconi anemia core complex associated protein 100, RGD1306926
FAAP24	AW124591, AW538696, C19orf40, C230052I12Rik, FA core complex associated protein 24, Fanconi anaemia core complex associated protein 24, Fanconi anemia core complex associated protein 24, RGD1564719
FANCA	AW208693, FA, FA1, FAA, FACA, FA complementation group A, FA-H, FANCH, Fanconi anaemia, complementation group A, Fanconi anemia, complementation group A
FANCB	BC022692, FA2, FAAP90, FAAP95, FAB, FACB, FA complementation group B, Fanconi anaemia, complementation group B, Fanconi anemia, complementation group B, FLJ34064, RGD1561555
FANCC	FA3, FAC, FACC, FA complementation group C, Fanconi anaemia, complementation group C, Fanconi anemia, complementation group C
FANCD2	2410150O07RIK, AU015151, BB137857, FA4, FACD, FA complementation group D2, FAD, FA-D2, FANCD, Fanconi anaemia, complementation group D2, Fanconi anemia, complementation group D2
FANCE	2810451D06RIK, AI415634, AW209126, FACE, FA complementation group E, FAE, Fanconi anaemia, complementation group E, Fanconi anemia, complementation group E, RGD1561045
FANCF	A730016A17, FA complementation group F, FAF, Fanconi anaemia, complementation group F, Fanconi anemia, complementation group F, RGD1561456
FANCG	AU041407, FA complementation group G, FAG, Fanconi anaemia, complementation group G, Fanconi anemia, complementation group G, LOC684204, Xrcc, XRCC9
FANCL	2010322C19Rik, AW554273, B230118H11Rik, FAAP43, FA complementation group L, Fanconi anaemia, complementation group L, Fanconi anemia, complementation group L, FLJ10335, gcd, P, Phf, PHF9, POG
FANCM	AI427100, C730036B14Rik, D12Ertd364, D12Ertd364e, FAAP250, FA complementation group M, Fanconi anaemia, complementation group M, Fanconi anemia, complementation group M, KIAA1596, POF15, SPGF28
H2AX	AW228881, gammaH, Gamma-h2afx, gamma-H2AX, gamma-H2A.X, H2af, H2AFX, H2A/X, H2A.X, H2A.X variant histone, Hist5-, RGD1566119, γ-h2afx, γ-H2AX, γ-H2A.X
HDAC	Histone Deacetylase, Histone deacetyltransferase
MLH1	1110035C23Rik, AI317206, AI325952, AI561766, COCA2, FCC2, hMLH1, HNPCC, HNPCC2, MMRCS1, mutL homolog 1
MRE11	ATLD, HNGS1, MRE11A, MRE11A homolog A, double strand break repair nuclease, MRE11B, MRE11 homolog, double strand break repair nuclease
MRN	MRE11-Rad50-NBS1, NBS1-Rad50-MRE11
MSH2	AI788990, COCA1, FCC1, hMSH2, HNPCC, HNPCC1, LCFS2, MMRCS2, mutS homolog 2
MSH6	AU044881, AW550279, GTB, GTBP, Gtmb, GTMBP, hMSH6, HNPCC5, HSAP, MMRCS3, Msh, mutS homolog 6, p160
MutL alpha	MLH1-PMS2, MutL α
MutS alpha	hMutS alpha, hMutS α, MSH2-MSH6, MutS α
NBN	ATV, AT-V1, AT-V2, Nb, NBS, NBS1, NIBRIN, P95
NPM1	B23, B23.1, B23NP, NO, NO38, NPM, Nucleolar protein B23.2, NUCLEOPHOSMIN, nucleophosmin 1, NUMATRIN
PALB2	4732427B05, BC066140, FANCN, FLJ21816, partner and localizer of BRCA2, PNCA3, RGD1304759
PI3K	1-phosphatidylinositol 3-kinase, 2.7.1.137, ATP:1-phosphatidyl-1D-myo-inositol 3-phosphotransferase, Phosphatidylinositol 3 kinase, phosphatidylinositol 3'-kinase, PI3-kinase, PtdIns 3 Kinase, type III phosphoinositide 3-kinase, type I phosphatidylinositol kinase, Vps34p
PIP2	1,2-diacyl-sn-glycero-3-phospho-(1'-myo-inositol-4',5'-bisphosphate), 1-O-(3-sn-phosphatidyl)-1D-myo-inositol 4,5-bis(dihydrogen phosphate), 1-phosphatidyl-1D-myo-inositol 4,5-bisphosphate, C11H19O19P3R2
PIP3	1-phosphatidyl-1D-myo-inositol 3,4,5-trisphosphate, phosphatidylinositol-3,4,5-trisphosphate, phosphoinositide (3,4,5) P3, PI(3,4,5)P3, PIns(3,4,5)P3, PIP3, PtdIns(3,4,5)P3
PMS2	AW555130, HNPCC4, LOC115486439, MLH4, MMRCS4, PMS1 homolog 2, mismatch repair system component, PMS2CL, PMSL2, Rsph10b
PTEN	10q23del, 2310035O07RIK, A130070J02Rik, AI463227, B430203M17RIK, BZS, CWS1, DEC, GLM2, MHAM, MMAC, MMAC1, MUTATED IN MULTIPLE ADVANCED CANCERS, mutated in multiple advanced cancers 1, phosphatase and tensin homolog, PTEN1, PTENbeta, TEP, TEP1
RAD50	hRad50, Mr, MreII, NBSLD, Rad, RAD502, RAD50 double strand break repair protein, Rad50l
RAD51	AV304093, BRCC5, FANCR, HRAD51, HsRad51, HsT16930, MRMV2, Rad, RAD51A, RAD51 recombinase, Re, RECA, RECA-LIKE, RGD1563603
RB1	OSRC, p, p105, p105-Rb, p110 RB, p110-RB1, pp105, pp110, PPP1R130, pRb, R, RB, RB-ASSOCIATED, RB transcriptional corepressor 1, Retinoblastome tumor-suppression protein rb
RNApolymeraseII	POL II, Polymerase II, RNAP-II, RNA POL II, RPase2
SFN	14-3-3, 14-3-3 Sigma, 14-3-3 σ, E, ER, HME1, Mme1, Stratifin, Ywh, YWHAS
Swi-Snf	SWI/SNF
TP53	bbl, BCC7, bfy, bhy, BMFS5, LFS1, p4, p44, p5, P53, P53 cellular tumour antigen, p53 tumor suppressor, transformation related protein 53, TRP53, tumor protein p53, tumour protein p53
TUBG1	1500010O08RIK, AI451582, AI503389, CDCBM4, Gamma-1 tubulin, Gamma-tubulin, GCP-1, TUBG, TUBGCP1, Tubulin gamma, tubulin gamma 1, tubulin, gamma 1, Tubulin γ, tubulin γ 1, tubulin, γ 1, γ-1 tubulin, γ-Tubulin
Ubiquitin	Polyubiquitin, Ub
WEE1	WEE1A, Wee1b, WEE1 G2 checkpoint kinase, WEE 1 homolog 1 (S. pombe), WEE1hu, WEE1-LIKE protein KINASE
XPC	p125, RAD4, xeroderma pigmentosum, complementation group C, XP3, XPCC, XPC complex subunit, DNA damage recognition and repair factor

Pathway Analysis Using IPA Software; canonical pathway

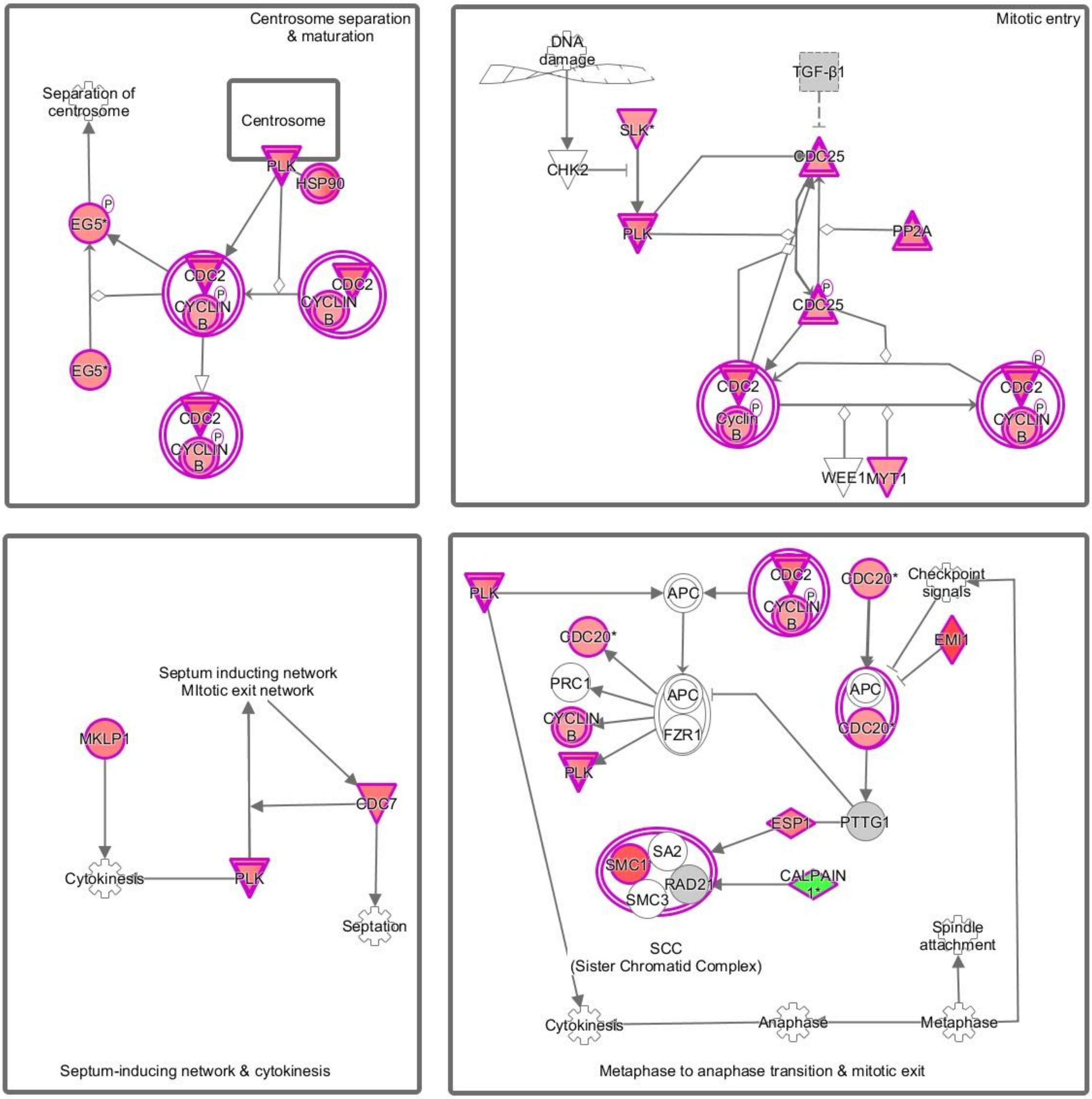

Figure S23. Mitotic Roles of Polo-Like Kinase at 24 h

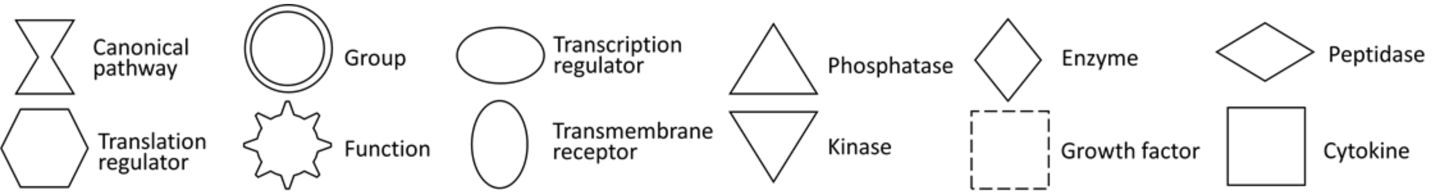

Red: Increased, FDR<0.05 versus solvent control

Green: Decreased, FDR<0.05 versus solvent control

Symbol	Synonym(s)
APC	Anaphase Promoting Complex, Anaphase Promoting Complex/Cyclosome, APC-C, APC/C, APC holoprotein
APC-CDC20	Anaphase promoting complex-CDC20
APC-FZR1	Anaphase promoting complex-FZR1
CAPN1	Ca, CALCIUM ACTIVATED NEUTRAL PROTEASE, Calpain-1, CALPAIN I, CANP, CANP1, CANPL1, Cap, Capa-1, mu-c, MU-CALPAIN, mu-calpin, muCANP, muCL, SPG76
CDC20	2310042N09Rik, bA276H19.3, C87100, CDC20A, cell division cycle 20, p55CD, p55CDC
CDC25	mRNA encoding Cdc25-like
CDC7	AI597260, Cdc7l, CDC7L1, CDC7-RELATED KINASE, cell division cycle 7, cell division cycle 7 (S. cerevisiae), HsCDC7, Hsk1, huCDC7, muCdc7
CHEK2	CDS1, Check2, checkpoint kinase 2, CHK2, hCds1, HUCDS1, LFS2, PP1425, Rad, RAD53
ESPL1	AL024103, AU045071, Ce, CERP, ES, ESP1, extra spindle pole bodies 1, separase, extra spindle pole bodies like 1, separase, LOC100912244, PR, PRCE, S, se, SEPA, SEPARASE, SSE
FBXO5	2510044I10Rik, C85305, Emi, EMI1, F-box protein 5, FBX5, Fbxo3, Fbxo31
FZR1	AW108046, CDC20C, CDH1, Cdh1/Hct1 homolog, fizzy and cell division cycle 20 related 1, Fy, FYR, FZR, FZR2, HCDH, HCDH1, HCT1
HSP90	HSC90, Hsp84
KIF11	EG5, HKSP, Kif, Kif8, Kifl1, Kinesin-5, kinesin family member 11, Kn, KNSL1, MCLMR, TRIP5
KIF23	3110001D19Rik, C87313, CHO, CHO1, kinesin family member 23, Kinesin-like 5, Kn, KNSL5, MKL, MKLP, MKLP-1
MPF	M-Phase Promoting Factor
PKMYT1	6230424P17, AW209059, MYT1, PPP1R126, protein kinase, membrane associated tyrosine/threonine 1, RGD1305434
PP2A	protein PHOSPHATASE 2A, Protein Phosphatase Type2a
PRC1	ASE1, D7Ertd348, D7Ertd348e, protein REGULATING CYTOKINESIS 1, protein regulator of cytokinesis 1
PTTG1	AW555095, C87862, EAP1, HPTTG, pituitary tumor-transforming gene 1, PITUITARY TUMOR TRANSFORMING GENE protein1, Pituitary tumour transforming 1, pituitary tumour-transforming gene 1, PITUITARY tumour TRANSFORMING GENE protein1, PTT, PTTG, PTTG1 regulator of sister chromatid separation, securin, Pttg3, sec, Securin, TUTR1
RAD21	CDLS4, hHR21, HR21, HRAD21, MCD1, MGS, mHR21, mKIAA0078, NXP1, Pw29, RAD21 cohesin complex component, SCC, SCC1
SLK	9A, 9A2, AV021402, AW411554, bA16H23.1, Etk4, KIAA0204, LOSK, mKIAA0204, mS, mSLK, S, se20-9, SK2, SMAK, STE20-like kinase, Stk, STK2
SMC1A	5830426I24Rik, CDLS2, DEE85, DXS423E, EIEE85, mKIAA0178, SB1., SB1.8, SMC1, SMC1alpha, SMC1 beta, SMC1L1, Smc1 α, SMC1 β, SMCB, STRUCTURAL MAINTENANCE of CHROMOSOMES 1, structural maintenance of chromosomes 1A
SMC3	BAM, Bamacan, BMH, CDLS3, Csp, CSPG6, HCAP, Mmi, MMIP1, Smc, SMC3L1, SmcD, structural maintenance of chromosomes 3
STAG2	9230105L23Rik, B230112I07Rik, bA517O1.1, HPE13, MKMS, NEDXCF, RGD1562042, S, SA, SA-2, SAP2, SCC3B, Stromal antigen 2
TGFB1	Beta Ig-h3, CED, DPD1, IBDIMDE, LAP, TGF-beta1, TGF-beta, TGF-β1, TGF-β, tgf-β(1), TGFB, transforming growth factor beta 1, transforming growth factor, beta 1, transforming growth factor, β 1, transforming growth factor-β 1, Transforming growth factor-β(1), β Ig-h3
WEE1	WEE1A, Wee1b, WEE1 G2 checkpoint kinase, WEE 1 homolog 1 (S. pombe), WEE1hu, WEE1-LIKE protein KINASE

Pathway Analysis Using IPA Software; canonical pathway

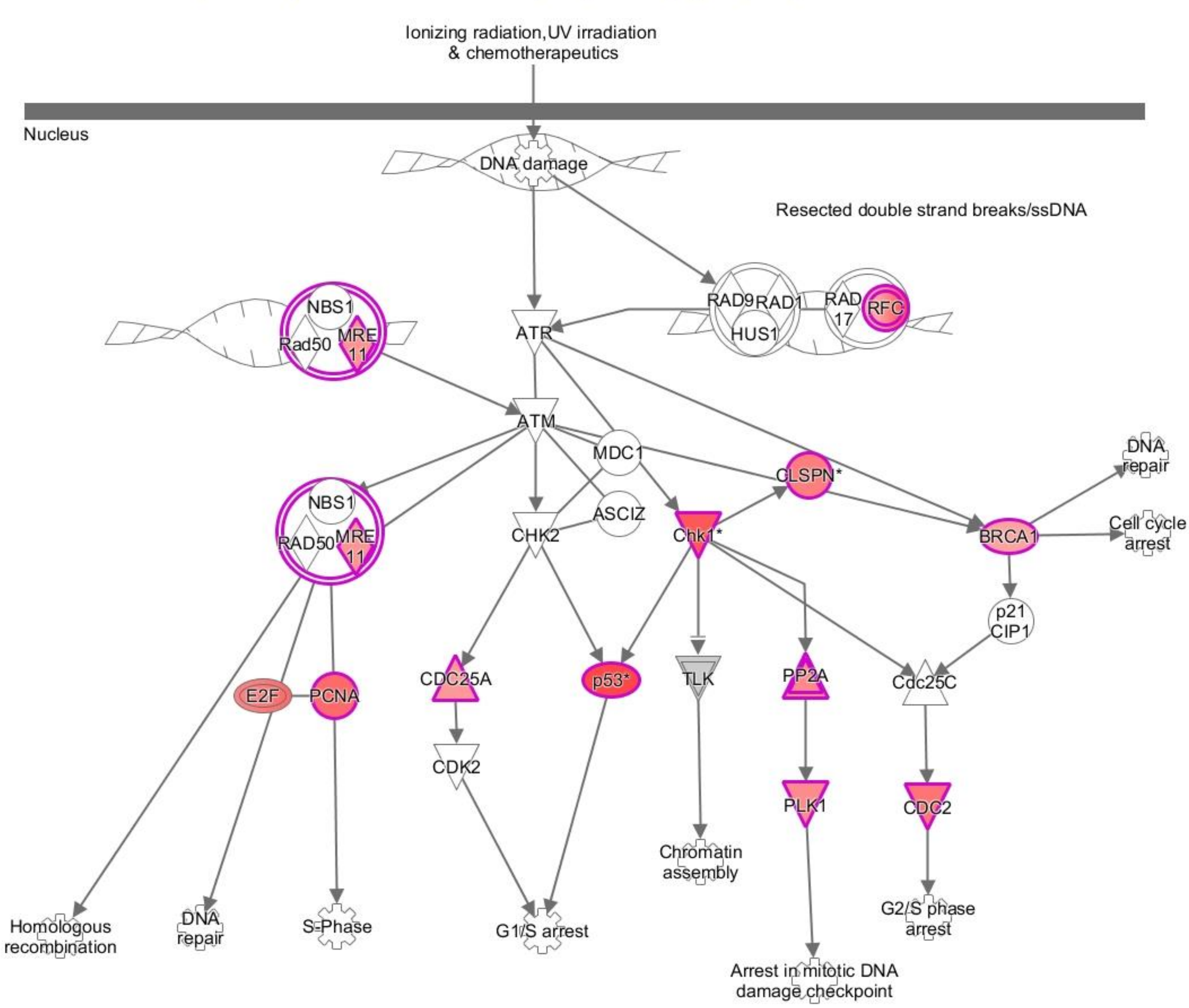

Figure S24. Role of CHK Proteins in Cell Cycle Checkpoint Control at 24 h

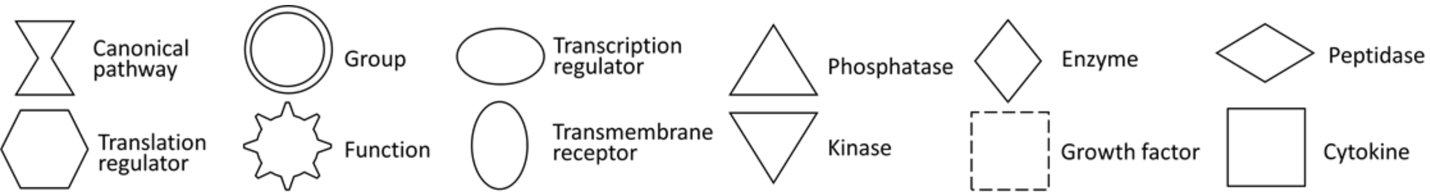

Red: Increased, FDR<0.05 versus solvent control

Green: Decreased, FDR<0.05 versus solvent control

Symbol	Synonym(s)
ATM	AI256621, AT1, ATA, ataxia telangiectasia mutated, ATC, ATD, ATDC, ATE, ATM serine/threonine kinase, C030026E19RIK, TEL1, TELO1
ATMIN	As, ASCIZ, ATM interactor, gpg, gpg6, KIAA0431, mKIAA0431, RGD1305781, ZNF822
ATR	ataxia telangiectasia and Rad3 related, Ataxia-telangiectasia-like, ATR serine/threonine kinase, FCTCS, FRP1, LOC100365674, LOC367198, LOC684113, MEC1, SCKL, SCKL1
BRCA1	BRCA1 DNA repair associated, BRCA1, DNA repair associated, BRCAI, BRCC1, breast cancer 1, early onset, BROVCA1, FANCS, PNCA4, PPP1R53, PSCP, RNF53
CDC25A	CDC25A2, cell division cycle 25A, D9Ertd393, D9Ertd393e
CDC25C	CDC25, cdc25c-64, cell division cycle 25C, PPP1R60
CDK1	CDC2, CDC28A, Cdc2a, CDC2 kinase, cyclin-dependent kinase 1, GROWTH-ASSOCIATED HISTONE H1 KINASE, p34, P34CDC2
CDK2	A630093N05Rik, CDC2-RELATED KINASE, CDKN2, Cyclin A associated kinase, cyclin-dependent kinase 2, CYCLIN E ASSOCIATED KINASE, p33(CDK2)
CDKN1A	CAP, CAP20, CDK, CDKI, Cdkn, CDKN1, CDKNA1, CI, CIP1, cyclin-dependent kinase inhibitor 1A, cyclin-dependent kinase inhibitor 1A (P21), mda, MDA-6, P2, P21, p21C, p21Cip, p21CIP1, p21W, p21WAF, p21Waf1, Pzl Cyclin-Dependent Kinase Inhibitor, SD, SDI1, UV96, Waf, WAF1
CHEK1	C85740, checkpoint kinase 1, CHK1, rad27
CHEK2	CDS1, Check2, checkpoint kinase 2, CHK2, hCds1, HUCDS1, LFS2, PP1425, Rad, RAD53
CLSPN	B130025E01, C85083, claspin, E130314M08RIK
HUS1	hHUS1, HUS1 checkpoint clamp component
MDC1	6820401C03, AA413496, mediator of DNA damage checkpoint 1, mKIAA0170, NFBD, NFBD1
MRE11	ATLD, HNGS1, MRE11A, MRE11A homolog A, double strand break repair nuclease, MRE11B, MRE11 homolog, double strand break repair nuclease
MRN	MRE11-Rad50-NBS1, NBS1-Rad50-MRE11
NBN	ATV, AT-V1, AT-V2, Nb, NBS, NBS1, NIBRIN, P95
PCNA	ATLD2, Pcna/cyclin, PCNAR, proliferating cell nuclear antigen
PLK1	P, PLK, polo-like kinase 1, STPK, STPK13
PP2A	protein PHOSPHATASE 2A, Protein Phosphatase Type2a
RAD17	CCYC, HRAD17, MmRad, MmRad24, R24L, RAD17 checkpoint clamp loader component, RAD17SP, RAD24
RAD50	hRad50, Mr, Mrell, NBSLD, Rad, RAD502, RAD50 double strand break repair protein, Rad50l
RAD9A	Ra, RAD9, RAD9 checkpoint clamp component A
TLK1/2	TLK
TP53	bbl, BCC7, bfy, bhy, BMFS5, LFS1, p4, p44, p5, P53, P53 cellular tumour antigen, p53 tumor suppressor, transformation related protein 53, TRP53, tumor protein p53, tumour protein p53

Pathway Analysis Using IPA Software; canonical pathway

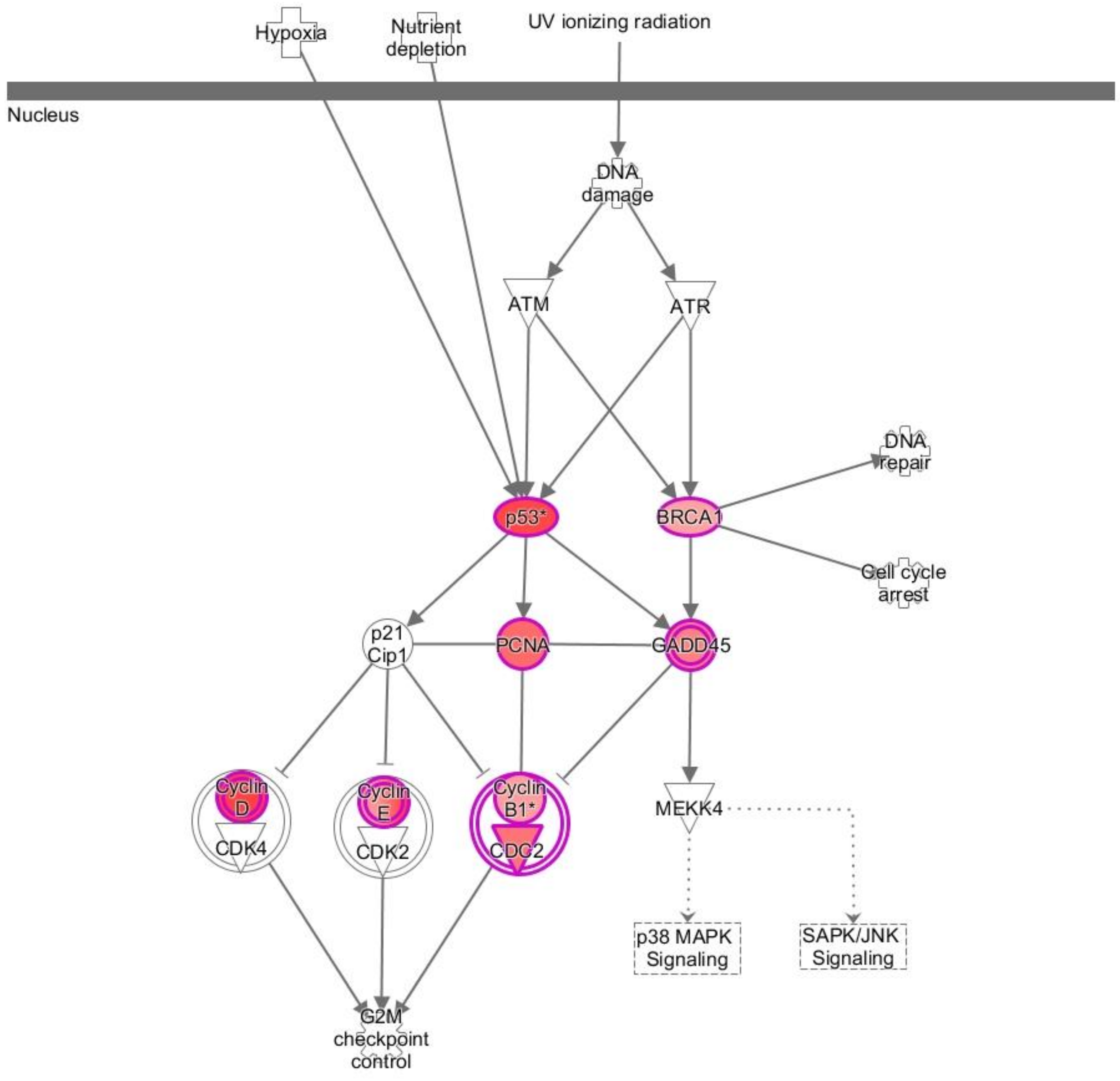

Figure S25. GADD45 Signaling at 24 h

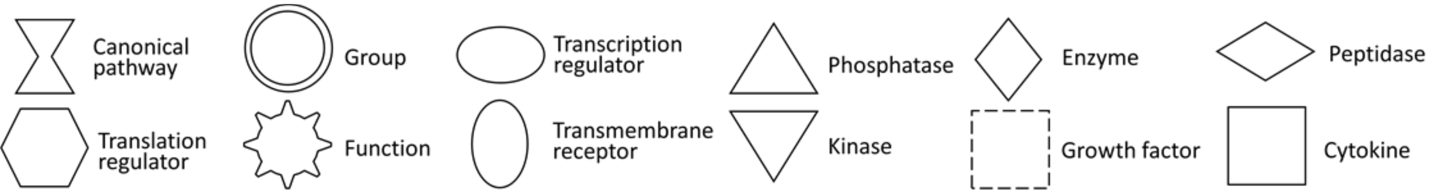

Red: Increased, FDR<0.05 versus solvent control

Green: Decreased, FDR<0.05 versus solvent control

Symbol	Synonym(s)
ATM	AI256621, AT1, ATA, ataxia telangiectasia mutated, ATC, ATD, ATDC, ATE, ATM serine/threonine kinase, C030026E19RIK, TEL1, TELO1
ATR	ataxia telangiectasia and Rad3 related, Ataxia-telangiectasia-like, ATR serine/threonine kinase, FCTCS, FRP1, LOC100365674, LOC367198, LOC684113, MEC1, SCKL, SCKL1
BRCA1	BRCA1 DNA repair associated, BRCA1, DNA repair associated, BRCAI, BRCC1, breast cancer 1, early onset, BROVCA1, FANCS, PNCA4, PPP1R53, PSCP, RNF53
CCNB1	CCNB, Ccnb1-ps, Ccnb1-r, CCNB1-RS1, Ccnb1-rs13, CycB1, Cycb1-rs1, Cycb-4, Cycb-5, CYCLIN B, CYCLIN B1, cyclin B1, pseudogene, Cyclin b4, EG434175, Gm5593
CDK1	CDC2, CDC28A, Cdc2a, CDC2 kinase, cyclin-dependent kinase 1, GROWTH-ASSOCIATED HISTONE H1 KINASE, p34, P34CDC2
CDK2	A630093N05Rik, CDC2-RELATED KINASE, CDKN2, Cyclin A associated kinase, cyclin-dependent kinase 2, CYCLIN E ASSOCIATED KINASE, p33(CDK2)
CDK2-CyclinE	Cyclin E-CDK2
CDK4	CMM3, Crk, Crk3, cyclin-dependent kinase 4, LOC100362034, PSK-J3
CDKN1A	CAP, CAP20, CDK, CDKI, Cdkn, CDKN1, CDKNA1, CI, CIP1, cyclin-dependent kinase inhibitor 1A, cyclin-dependent kinase inhibitor 1A (P21), mda, MDA-6, P2, P21, p21C, p21Cip, p21CIP1, p21W, p21WAF, p21Waf1, PzI Cyclin-Dependent Kinase Inhibitor, SD, SDI1, UV96, Waf, WAF1
Cyclinb1/Cdc2	Cyclin B1-Cdc2
CyclinD	CycD, Cyclin D1
FOXO3	1110048B16RIK, 2010203A17RIK, AF6q21, C76856, FKHR, Fkhr2, FKHRL1, FKHRL1P2, forkhead box O3, Fox, FOXO2, FOXO3A
GADD45A	AA545191, Ddit, DDIT1, Gadd, GADD45, GADD45 alpha, GADD45α, growth arrest and DNA-damage-inducible 45 alpha, growth arrest and DNA-damage-inducible 45 α, Growth arrest and DNA-damage-inducible 45, α, growth arrest and DNA damage inducible alpha, growth arrest and DNA-damage-inducible, alpha, growth arrest and DNA damage inducible α, growth arrest and DNA-damage-inducible, α
GADD45B	AI323528, GADD45beta, Gadd45β, growth arrest and DNA-damage-inducible 45 beta, growth arrest and DNA-damage-inducible 45 β, growth arrest and DNA damage inducible beta, growth arrest and DNA-damage-inducible, beta, growth arrest and DNA damage inducible β, growth arrest and DNA-damage-inducible, β, MYD118, Myeloid differentiation primary response
GADD45G	AI327420, C86281, CR, CR6, DDIT2, GADD45gamma, Gadd45-γ, Growth Arrest And DNA Damage Inducible, growth arrest and DNA-damage-inducible 45 gamma, growth arrest and DNA-damage-inducible 45 γ, growth arrest and DNA damage inducible gamma, growth arrest and DNA-damage-inducible, gamma, growth arrest and DNA damage inducible γ, growth arrest and DNA-damage-inducible, γ, GRP17, OIG, OIG37
Genistein	446-72-0, 4',5,7-trihydroxyisoflavone, 4H-1-benzopyran-4-one, 5,7-dihydroxy-3-(4-hydroxyphenyl)-, 5,7-dihydroxy-3-(4-hydroxyphenyl)chromen-4-one, BIO 300, C15H10O5, G103, genestein, gentistein, Prunetol, PTI-G4660, SIPI-9764-I, Sophoricol
IL1B	IL-, IL-1, IL1-BETA, IL-1F2, IL-1 β, interleukin 1 beta, Interleukin 1 β, OAF, Osteoclast-Activating Factor, Pro-IL-1beta, Pro-IL-1β
JNK	JNK 54/46, Jnk p56, JNK/SAPK, JUN KINASE, p40, p47, Sapk/Jnk
MAP2K3	AW212142, LOC100911550, MAP KINASE KINASE 3B, MAPKK3, MEK3, mitogen-activated protein kinase kinase 3, MKK3, Mkk3b, Prkm, PRKMK3, SAPKK-2
MAP2K4	JNKK, JNKK1, MAPK/ERK KINASE-1, MAPKK4, MEK4, mitogen-activated protein kinase kinase 4, MKK4, PRKMK4, SAPKK-1, Sek, SEK1, Ser, SERK1, SKK1
MAP2K6	MAPKK6, Mapk kinase 6, MEK6, mitogen-activated protein kinase kinase 6, MKK6, MKK6BE, Prkm, PRKMK6, Rac, SAP, SAPKK-3
MAP2K7	5930412N11Rik, JNKK, JNKK 2, MAPK2K7, MAPKK 7, Mapkk7 protein 2, MEK, MEK 7, mitogen-activated protein kinase kinase 7, MKK7, Mkk7 beta1, Prkm, PRKMK7, SAPKK-4, sek, sek2
MAP3K4	D17Rp, D17Rp1, D17Rp17, D17Rp17e, MAPK3K4, MAPKK, MAPKKK4, Mek4b, MEKK1, MEKK 4, mitogen-activated protein kinase kinase kinase 4, mKIAA0213, MTK1, PRO0412, RP, RP17, Rp17a, T, TAS
MIR130B	bta-mir-130a, cfa-mir-130a, HSA-MIR-130, hsa-mir-130a, hsa-mir-130b, hsa-mir-301, hsa-mir-301a, hsa-mir-301b, microRNA 130, microRNA 130a, microRNA 130b, microRNA 301, microRNA 301a, microRNA 301b, MIR130A, MIR130B, mir-30, MIR301, MIR301A, MIR301B, Mirn, Mirn1, Mirn130, MIRN130A, MIRN130B, Mirn3, MIRN301, MIRN301A, MIRN301B, miRNA130A, mml-mir-130a, Mmu-mir-130, mmu-mir-130a, mmu-mir-130b, mmu-mir-3, mmu-mir-301, mmu-mir-301a, mmu-mir-301b, rno-mir-130a, rno-mir-130b, rno-mir-301a, rno-mir-301b, ssc-mir-130a
MIR383	hsa-mir-383, microRNA 383, Mirn, MIRN383, mmu-mir-3, mmu-mir-383, rno-mir-383
MMS	66-27-3, C2H6O3S, methanesulfonic acid methyl ester, methanesulfonic acid, methyl ester, methyl mesylate, methyl methanesulfonate, methyl methanesulphonate, MMS
MYC	AU016757, bHLHe3, bHLHe39, CMYC, C-MYC-P64, mMyc, MRTL, Myc2, MYCC, MYC proto-oncogene, bHLH transcription factor, myelocytomatosis oncogene, N, Niard, Nird, RNCMYC
NFkB	NF-KAPPA B, NF-κ B, nuclear factor-κ b, transcription factor nuclear factor κ b
NFYA	AA407810, CBF-A, CBF-B, FLJ11236, HAP2, LOC100129914, NFY, nuclear transcription factor-Y alpha, nuclear transcription factor Y subunit alpha, nuclear transcription factor Y subunit α, nuclear transcription factor-Y α, Nuclear transcription factor y, α, isoform 1, Nuclear transcription factor y, α, isoform 2, Sez1, SEZ-10
p38 MAPK	P38, p38 MAP KINASE, P38 MITOGEN-ACTIVATED protein KINASE
PCNA	ATLD2, Pcna/cyclin, PCNAR, proliferating cell nuclear antigen
POU2F1	2810482H01Rik, LOC100503933, NF-A1, Oct-, OCT1, oct-1B, Otf-, OTF1, POU class 2 homeobox 1, POU domain, class 2, transcription factor 1
Smad2/3-Smad4	Smad 2/3/4
SMAD4	AW743858, D18Wsu70, D18Wsu70e, DPC, DPC4, JIP, Madh, MADH4, MYHRS, SMAD family member 4, Smaug1
Tgfbeta	Tgfb, TGF-beta 1, 2, and 3, TGF β, TGF-β 1, 2, and 3, transforming growth factor-β
TNF	AT-TNF, DI, DIF, RATTNF, TMTNF, Tn, TNF-a, TNF-alpha, Tnfs, Tnfsf1a, TNFSF2, TNF-α, TNLG1F, tumor necrosis factor, Tumor Necrosis Factor α, tumor necrosis factor, α, tumour necrosis factor, tumour Necrosis Factor Alpha, tumour necrosis factor, alpha, tumour Necrosis Factor α, tumour necrosis factor, α
TP53	bbl, BCC7, bfy, bhy, BMFS5, LFS1, p4, p44, p5, P53, P53 cellular tumour antigen, p53 tumor suppressor, transformation related protein 53, TRP53, tumor protein p53, tumour protein p53
TSA	2,4-heptadienamide, 7-(4-(dimethylamino)phenyl)-N-hydroxy-4,6-dimethyl-7-oxo-, (2E,4E,6R)-, 2,4-heptadienamide, 7-[4-(dimethylamino)phenyl]-N-hydroxy-4,6-dimethyl-7-oxo-, (2E,4E,6R)- (9CI), (2E,4E,6R)-7-[4-(dimethylamino)phenyl]-N-hydroxy-4,6-dimethyl-7-oxohepta-2,4-dienamide, 58880-19-6, C17H22N2O3, trichstatin A, TSA
WT1	D630046I19RIK, GUD, NPHS4, WAGR, Wilms tumor 1 homolog, wilms' tumour, wilms tumour 1, Wilms tumour 1 homolog, Wilms tumour-suppressor, Wilms' tumour suppressor, WIT-2, WT, WT1 transcription factor, WT33
ZNF350	ZBRK1, ZFQR, zinc finger protein 350

Pathway Analysis Using IPA Software; canonical pathway

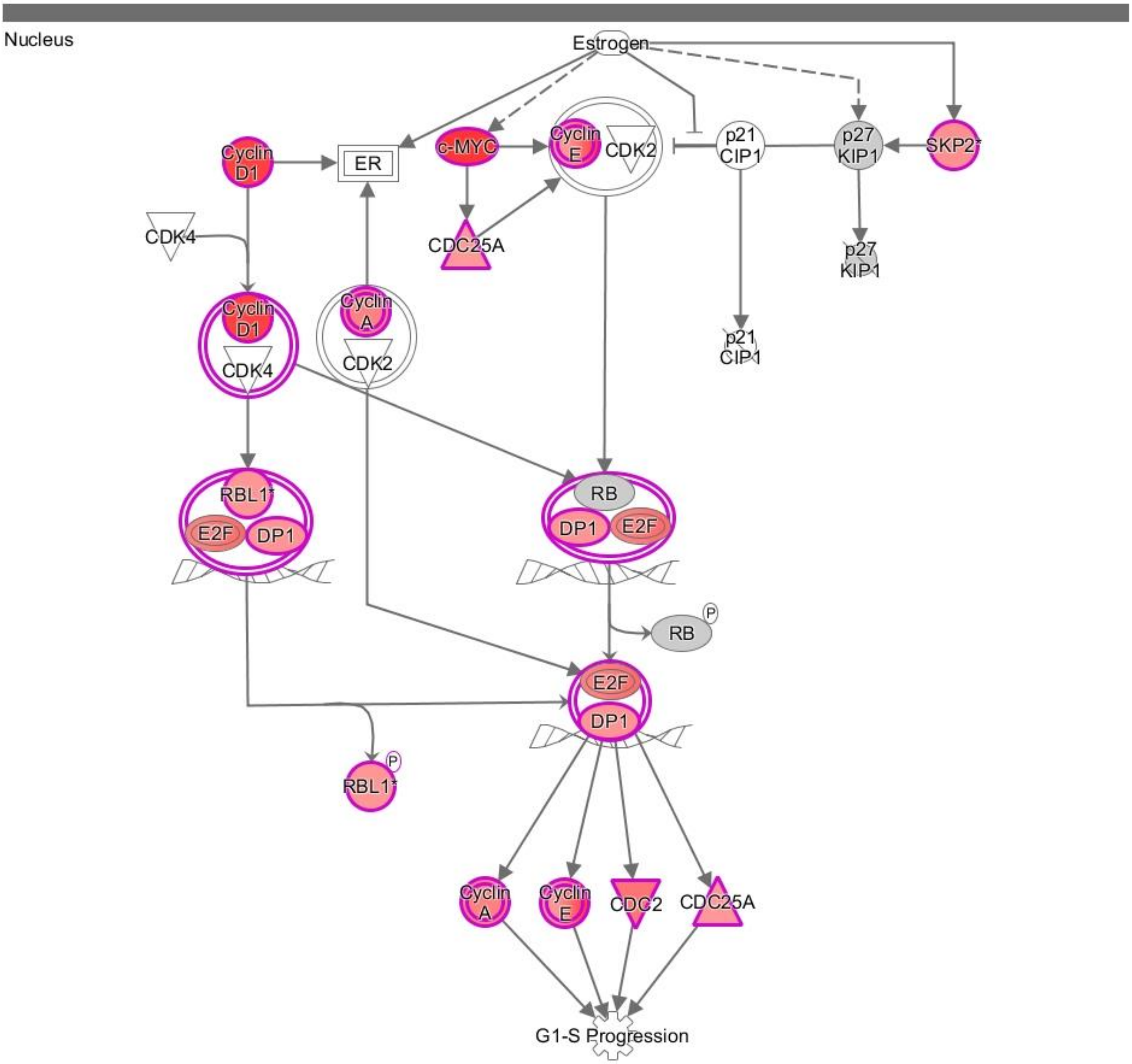

Figure S26. Estrogen-mediated S-phase Entry at 24 h

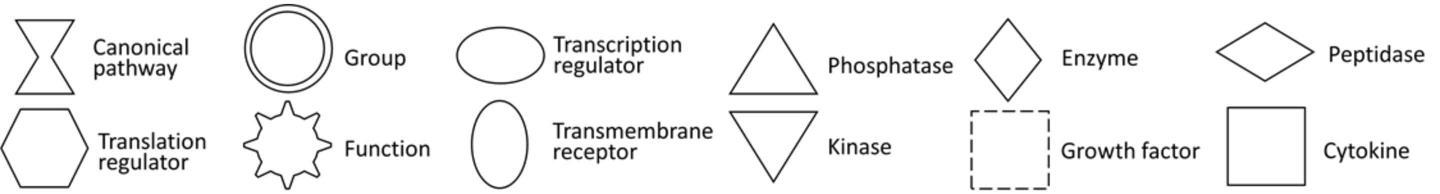

Red: Increased, FDR<0.05 versus solvent control

Green: Decreased, FDR<0.05 versus solvent control

Symbol	Synonym(s)
CCND1	AI327039, B-CELL CLL/LYMPHOMA 1, bcl-, BCL1, cD1, CycD1, CYCLIN D1, Cyl-, Cyl-1, D11S287E, G1/S-Specific Cyclin D1, PR, PRAD1, U21B31
CDC25A	CDC25A2, cell division cycle 25A, D9Ertd393, D9Ertd393e
CDK1	CDC2, CDC28A, Cdc2a, CDC2 kinase, cyclin-dependent kinase 1, GROWTH-ASSOCIATED HISTONE H1 KINASE, p34, P34CDC2
CDK2	A630093N05Rik, CDC2-RELATED KINASE, CDKN2, Cyclin A associated kinase, cyclin-dependent kinase 2, CYCLIN E ASSOCIATED KINASE, p33(CDK2)
CDK2-CyclinE	Cyclin E-CDK2
CDK4	CMM3, Crk, Crk3, cyclin-dependent kinase 4, LOC100362034, PSK-J3
CDKN1A	CAP, CAP20, CDK, CDKI, Cdkn, CDKN1, CDKNA1, CI, CIP1, cyclin-dependent kinase inhibitor 1A, cyclin-dependent kinase inhibitor 1A (P21), mda, MDA-6, P2, P21, p21C, p21Cip, p21CIP1, p21W, p21WAF, p21Waf1, Pzl Cyclin-Dependent Kinase Inhibitor, SD, SDI1, UV96, Waf, WAF1
CDKN1B	AA408329, AI843786, Cdki1b, CDKN4, cyclin-dependent kinase inhibitor 1B, CYCLIN-DEPENDENT KINASE INHIBITOR P27, KIP1, MEN1B, MEN4, p2, p27, p27K, P27kip, P27KIP1, P28-ICK
CyclinA/Cdk2	CDK2-Cyclin A
CyclinD1/cdk4	CDK4-Cyclin D1, Cyclin D1-CDK4
E2f-Tfdp1	E2F-DP1
Estrogen	C18 steroids, oestrogen
Estrogenreceptor	ER, ESR, ESR1/2, esr1/esr2
MYC	AU016757, bHLHe3, bHLHe39, CMYC, C-MYC-P64, mMyc, MRTL, Myc2, MYCC, MYC proto-oncogene, bHLH transcription factor, myelocytomatosis oncogene, N, Niard, Nird, RNCMYC
Rb-E2F-DP1	DP1-E2F-Rb
RB1	OSRC, p, p105, p105-Rb, p110 RB, p110-RB1, pp105, pp110, PPP1R130, pRb, R, RB, RB-ASSOCIATED, RB transcriptional corepressor 1, Retinoblastome tumor-suppression protein rb
RBL1	AW547426, CP107, LOC683869, p10, p107, PRB1, RB transcriptional corepressor like 1
SKP2	4930500A04Rik, AC139209.1, cyclin A-associated kinase, FBL1, F-box protein Skp2, FBXL, FBXL1, FLB1, FWD1, p45, p45Skp2, RGD1562456, S-PHASE KINASE-ASSOCIATED protein 2
TFDP1	DILC, Dp, DP-1, Drtf, DRTF1, TB2/DP1, transcription factor Dp-1

Pathway Analysis Using IPA Software; canonical pathway

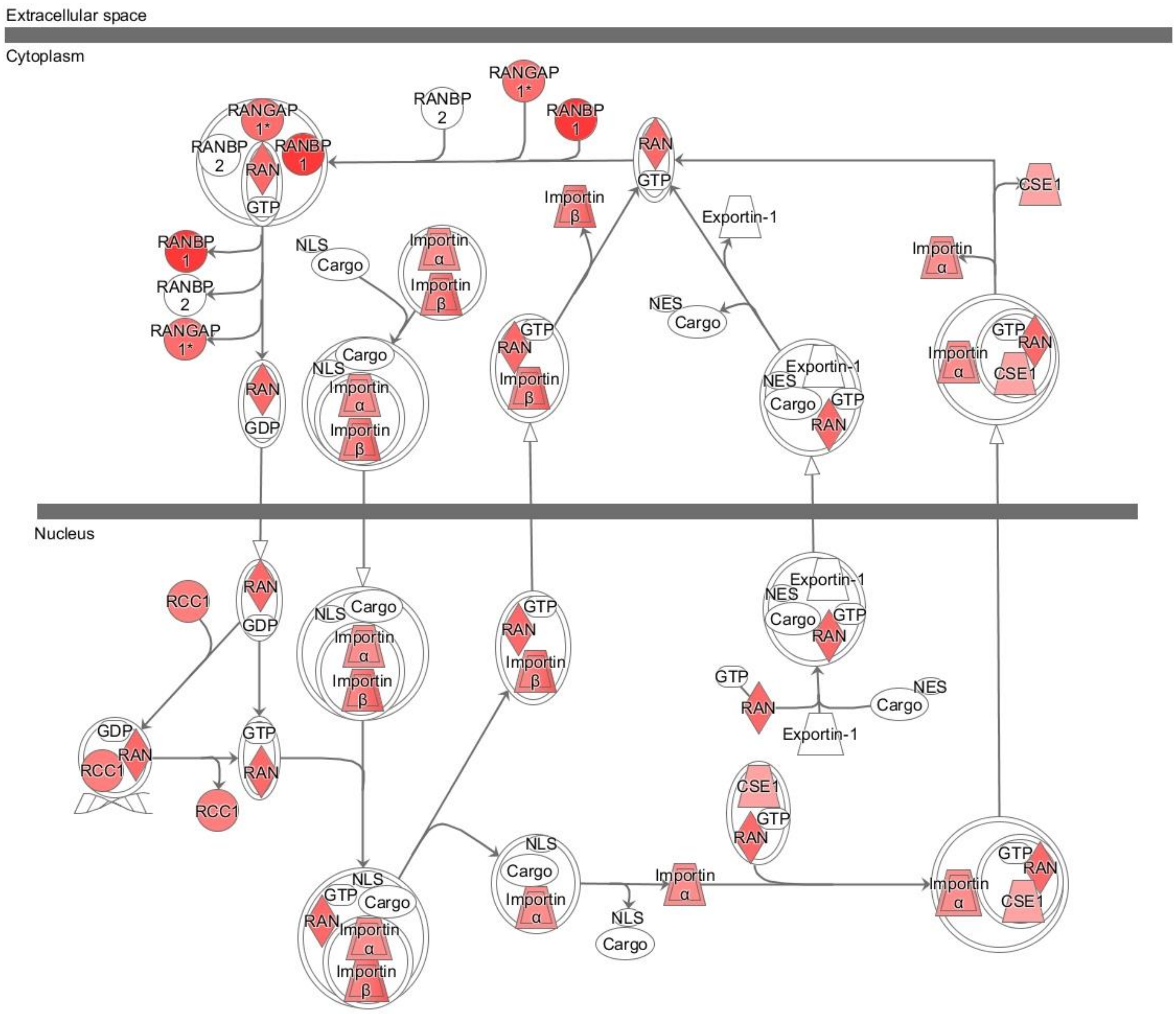

Figure S27. RAN Signaling at 24 h

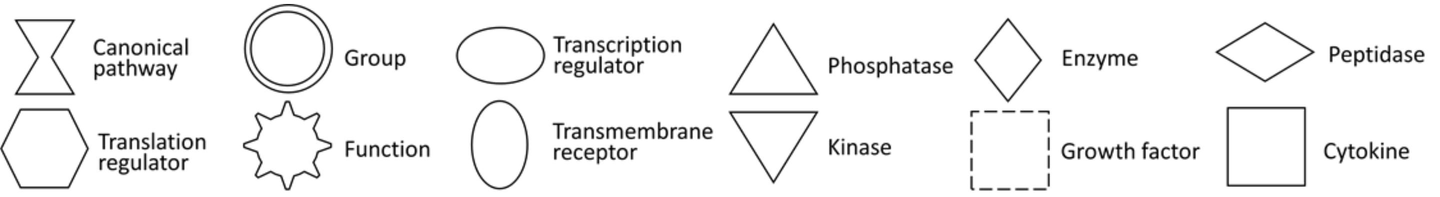

Red: Increased, FDR<0.05 versus solvent control

Green: Decreased, FDR<0.05 versus solvent control

Symbol	Synonym(s)
CSE1L	2610100P18Rik, AA407533, C, Ca, Capts, CAS, chromosome segregation 1 like, chromosome segregation 1-like (S. cerevisiae), CSE1, Exportin-2, Xp, XPO2
GDP	146-91-8, [(2R,3S,4R,5R)-5-(2-amino-6-oxo-1H-purin-9-yl)-3,4-dihydroxyoxolan-2-yl]methyl phosphono hydrogen phosphate, C10H15N5O11P2, guanosine 5'-(trihydrogen diphosphate), guanosine diphosphate
GTP	[[(2R,3S,4R,5R)-5-(2-amino-6-oxo-1H-purin-9-yl)-3,4-dihydroxyoxolan-2-yl]methoxy-hydroxyphosphoryl] phosphono hydrogen phosphate, 86-01-1, C10H16N5O14P3, GTP, guanosine 5'-(tetrahydrogen triphosphate), Mg-GTP
Importinalpha	IMP alpha, Importin α, IMP α, Kap alpha, Kap α, Karyopherin alpha, Karyopherin α
Importinalpha/beta	Importin, Importin α/β
Importinbeta	Imp beta, Importin β, Imp β, Karyopherin beta, Karyopherin β
Importinα-Cargo	Importinalpha-Cargo
Importinα-RAN-GTP-CSE1	Importinalpha-RAN-GTP-CSE1
Importinβ-RAN-GTP	Importinbeta-RAN-GTP
RAN	ARA24, Gsp1, GTPase Ran, RANGTPASE, RAN, member RAS oncogene family, Rasl2-9, RAS-like, family 2, locus 9, TC4
RANBP1	Htf9, HTF9A, RAN binding protein 1, Ran-Specific GTPase-Activating
RANBP2	A430087B05Rik, ADANE, AI256741, ANE1, IIAE3, NUP358, RAN binding protein 2, RGD1560047, TRP1, TRP2
RANGAP1	C79654, Fug1, mKIAA1835, RANGAP, RAN GTPase activating protein 1, SD
RCC1	4931417M11Rik, AI326872, CHC1, RCC1-I, regulator of chromosome condensation 1, RENAL CELL CARCINOMA 1, SNHG3-RCC1
XPO1	AA420417, Crm, CRM-1, CRMA, emb, Exp1, Exportin-1

Pathway Analysis Using IPA Software; canonical pathway

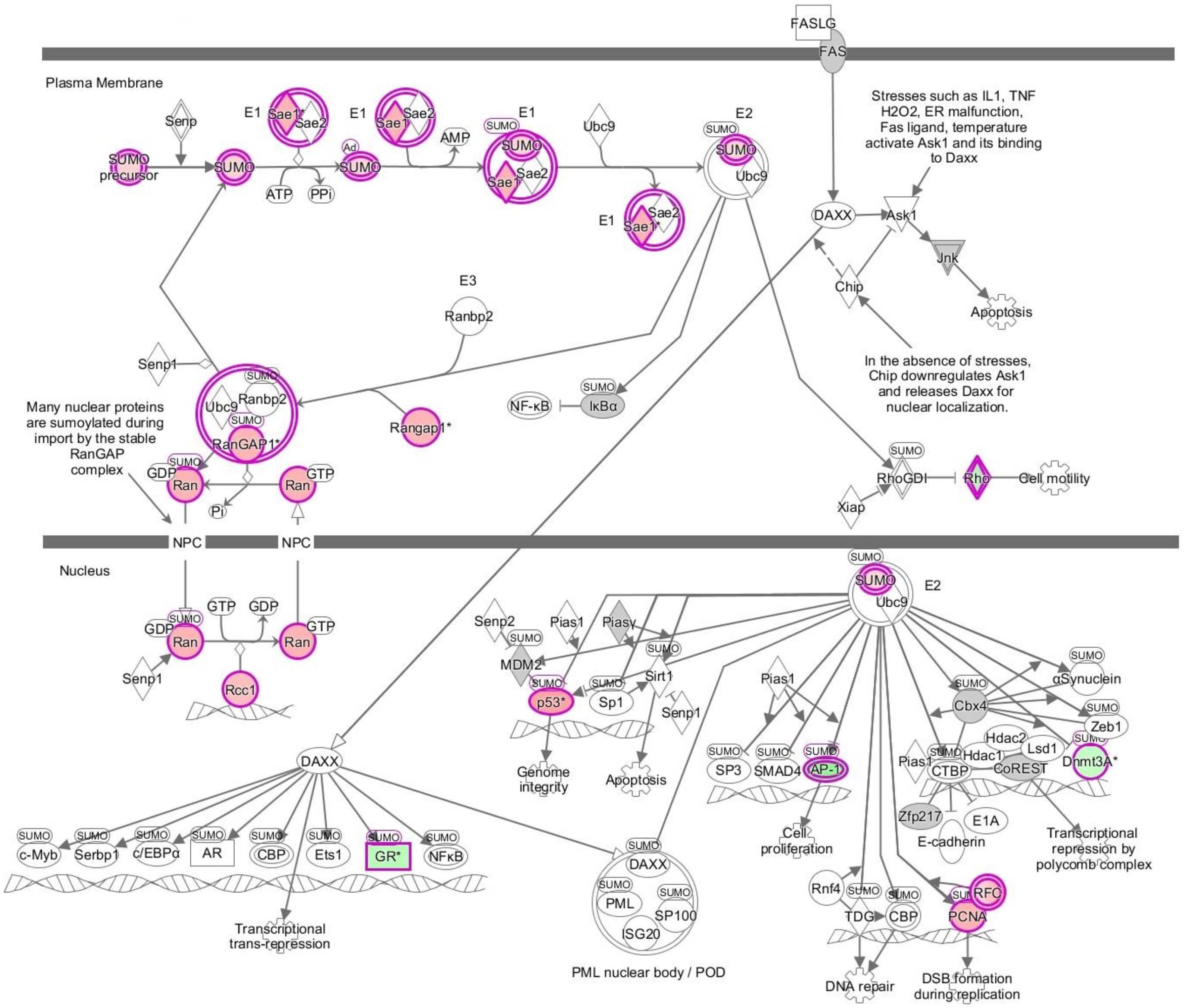

Figure S28. Sumoylation Pathway at 24 h

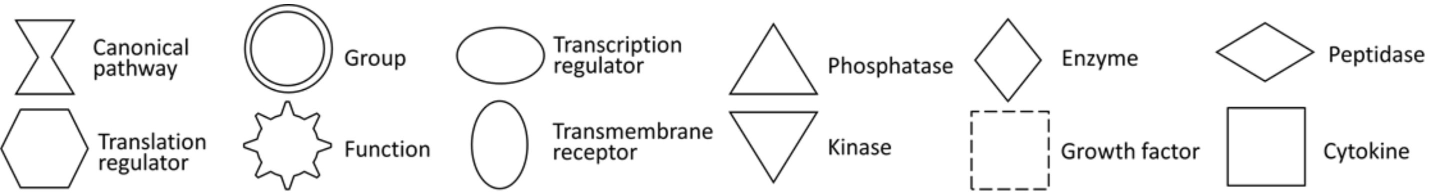

Red: Increased, FDR<0.05 versus solvent control

Green: Decreased, FDR<0.05 versus solvent control

Symbol	Synonym(s)
AMP	149022-20-8, [(2R,3S,4R,5R)-5-(6-aminopurin-9-yl)-3,4-dihydroxyoxolan-2-yl]methyl dihydrogen phosphate, 5'-adenylic acid, 5' AMP, 5'-AMP, 61-19-8, adenosine-5-monophosphate, adenosine-5-phosphate, adenosine monophosphate, C10H14N5O7P
Ap1	activator protein-1, c-Jun
AR	AIS, Andr, androgen receptor, AW320017, DHTR, HUMARA, HYSP1, KD, NR3C4, SBMA, SMAX1, Testosterone receptor, TFM
ATP	[[(2R,3S,4R,5R)-5-(6-aminopurin-9-yl)-3,4-dihydroxyoxolan-2-yl]methoxy-hydroxyphosphoryl] phosphono hydrogen phosphate, 56-65-5, 9-beta-D-arabinofuranosyladenine 5'-triphosphate, 9-β-D-arabinofuranosyladenine 5'-triphosphate, adenosine 5'-(tetrahydrogen triphosphate), adenosine 5'-triphosphate, ATP, ATP4-, C10H16N5O13P3
CBP/p300	CBP, CBP-p300
CBX4	chromobox 4, Hpc2, MPc, MPc2, NBP16, PC, PC2
CDH1	AA960649, ARC-1, BCDS1, cadherin 1, Cadherin E, CD324, CDHE, CSEIL, E-ca, ECAD, E-cadh, E-cadherin, L-C, L-CAM, Um, UVO, uvomorulin
CEBPA	CBF-A, CCAAT enhancer binding protein alpha, CCAAT/enhancer binding protein alpha, CCAAT/enhancer binding protein (C/EBP), alpha, CCAAT/enhancer binding protein (C/EBP), α, CCAAT enhancer binding protein α, CCAAT/enhancer binding protein α, Ceb, CEBP, C/ebp, Cebp1a, C/EBP-alpha, Cebp Alpha, C/EBP alpha P30, C/EBP alpha P42, CEBPα, C/EBP-α, C/EBP α P30, C/EBP α P42, C/ERB, DBPCEP, p42, Zinc Finger Homeobox 1b
CTBP	CTBP1/2
DAXX	BING2, DAP6, death-domain associated protein, EAP1, Fas death domain-associated protein, PML ASSOCIATED FACTOR
DNMT3A	DNA Methyltransferase 3A, DNA methyltransferase 3 alpha, DNA methyltransferase 3 α, DNA MTase HsaIIIA, DNMT3A2, HESJAS, M.HsaIIIA, MmuIIIA, TBRS
ETS1	AI196000, AI448617, c-ets-1, D230050P06, E26 avian leukaemia oncogene 1, 5' domain, E26 avian leukemia oncogene 1, 5' domain, Ets-, Etsoncb, ETS proto-oncogene 1, transcription factor, EWSR2, p42Ets, p42 ETS1, p51Ets, p54, Tp, TPL1, v-Ets, vs
FAS	AI196731, ALPS1A, AP, APO-1, APT1, CD95, CD95 receptor, CD95L, FAS1, Fas (TNF receptor superfamily member 6), Fas cell surface death receptor, FAS/APO1, FasR, FASTM, lpr, Receptor for Fas Ligand, Receptors for Fas Ligand, TNF, Tnf receptor member 6, TNFR6, Tnfr, TNFRSF6
FASLG	ALPS1B, APT1, APT1LG1, APTL, CD178, CD95, CD95-L, F, Fa, FASL, Fas Ligand, Fas ligand (TNF superfamily, member 6), gld, mFasL, Tnfl6, Tnfs, TNFSF6, TNLG1A
GDP	146-91-8, [(2R,3S,4R,5R)-5-(2-amino-6-oxo-1H-purin-9-yl)-3,4-dihydroxyoxolan-2-yl]methyl phosphono hydrogen phosphate, C10H15N5O11P2, guanosine 5'-(trihydrogen diphosphate), guanosine diphosphate
GTP	[[(2R,3S,4R,5R)-5-(2-amino-6-oxo-1H-purin-9-yl)-3,4-dihydroxyoxolan-2-yl]methoxy-hydroxyphosphoryl] phosphono hydrogen phosphate, 86-01-1, C10H16N5O14P3, GTP, guanosine 5'-(tetrahydrogen triphosphate), Mg-GTP
HDAC1	GON-10, HD1, HDAC, Hdac1-ps, histone deacetylase 1, KDAC1, LOC630524, MommeD, MommeD5, RP, RPD3, RPD3L1
HDAC2	D10Wsu179, D10Wsu179e, HD2, histone deacetylase 2, KDAC2, mRPD3, RGD: 619976, RPD3, YAF1, Yy1b, Yy1bp
ISG20	1600023I01Rik, 2010107M23Rik, CD25, Dn, DnaQL, HEM45, HEM46, interferon stimulated exonuclease gene 20, Interferon Stimulated Gene 20kd, interferon-stimulated protein, IS320
Jnk	JNK 54/46, Jnk p56, JNK/SAPK, JUN KINASE, p40, p47, Sapk/Jnk
KDM1A	1810043O07Rik, AA408884, Ao, AOF2, BHC110, CPRF, D4Ertd478e, KDM1, KIAA0601, LS, LSD1, lysine demethylase 1A, lysine (K)-specific demethylase 1A, mKIAA0601, RGD1562975
MAP3K5	7420452D20Rik, A, APOPTOSIS SIGNAL REGULATED KINASE 1, AS, ASK, ASK1, M3K5, MAPKKK5, MEKK5, mitogen-activated protein kinase kinase kinase 5, RGD1306565
MDM2	1700007J15Rik, AA415488, ACTFS, hdm2, HDMX, LSKB, MDM2-A1, MDM2 proto-oncogene, MGC5370, Transformed 3t3 cell double minute 2, transformed mouse 3T3 cell double minute 2
MYB	AI550390, Cmyb, c-myb CDS, efg, M16449, MYB proto-oncogene, transcription factor, myeloblastosis oncogene
NFkB	NF Kappa B, NF-kappaB p50/p52, NF-κB, NF-κB p50/p52
NFKBIA	AI462015, EDAID2, I kappa B alpha, IkappaB α, IKBA, Ikb Kinase Alpha, Ikb Kinase α, IKBM, IkB α, I κ B α, I(κ)B(α), MAD-3, Nfk, NF KAPPA beta alpha, NFKBI, NFKB inhibitor alpha, NFKB inhibitor α, NF κ β α, nuclear factor of kappa light polypeptide gene enhancer in B cells inhibitor, alpha, nuclear factor of κ light polypeptide gene enhancer in B cells inhibitor, α, pIkappaB α, RL/IF-1
NR3C1	G, GCCR, GCR, GCR alpha, GCRST, GCR α, Glucocorticoid receptor, Glucocorticoid receptor α-2, GR, GR-A, Gr alpha, Gr beta, GRL, Grl-1, Gr α, Gr β, nuclear receptor subfamily 3 group C member 1, nuclear receptor subfamily 3, group C, member 1, Type ii corticosteroid receptor
PCNA	ATLD2, Pcna/cyclin, PCNAR, proliferating cell nuclear antigen
Pi	14265-44-2, inorganic phosphate, O4P-3, P, phosphate, phosphate(3-), phosphate ion, Pi
PIAS1	2900068C24Rik, Ddxbp, DDXBP1, GB, GBP, GU/RH-II, protein inhibitor of activated STAT 1, protein inhibitor of activated STAT, 1, ZMIZ3
PIAS4	P, Piasg, PIAS-gamma, PIASY, PIAS-γ, protein inhibitor of activated STAT 4, protein inhibitor of activated STAT, 4, ZMIZ6
PML	1200009E24Rik, AI661194, PML nuclear body scaffold, PP8675, promyelocytic leukaemia, promyelocytic leukemia, RGD1562602, RNF71, Trim, TRIM19
PPi	14000-31-8, 2466-09-3, diphosphate, diphosphate(4-), inorganic pyrophosphate, O7P2-4, phosphonato phosphate, PPi, pyrophosphate ion
RAN	ARA24, Gsp1, GTPase Ran, RANGTPASE, RAN, member RAS oncogene family, Rasl2-9, RAS-like, family 2, locus 9, TC4
RANBP2	A430087B05Rik, ADANE, AI256741, ANE1, IIAE3, NUP358, RAN binding protein 2, RGD1560047, TRP1, TRP2
RANGAP1	C79654, Fug1, mKIAA1835, RANGAP, RAN GTPase activating protein 1, SD
RCC1	4931417M11Rik, AI326872, CHC1, RCC1-I, regulator of chromosome condensation 1, RENAL CELL CARCINOMA 1, SNHG3-RCC1
RCOR1	5730409O11, 6720480E22Rik, AU042633, COREST, D12Wsu95, D12WSU95E, mKIAA0071, RCOR, REST corepressor 1, RGD1305743, Ro, Rocr1
Rho	GTPase Rho, Rho, Rho Family, RHO-GTPASE, Rho-like Gtpase
Rho-GDI	Rabgdi, RHO GUANINE NUCLEOTIDE DISSOCIATION INHIBITOR
RNF4	AU018689, Gtrge, GTRGEO8, RES4-26, ring finger protein 4, SLX5, SNURF
SAE1	2400010M20Rik, 2610044L12Rik, A, AL033372, AOS1, AW743391, D7Ertd177, D7Ertd177e, HSPC1, HSPC140, SUA1, SUMO1 activating enzyme subunit 1, Uble, UBLE1A
SENP	SUMO-specific protease
SENP1	2310046A20Rik, D15Ertd528, D15Ertd528e, E330036L07RIK, SUMO1/sentrin specific peptidase 1, SUMO specific peptidase 1, SuPr-2
SENP2	2310007L05RIK, 4930538C18Rik, AI646780, AW554757, AXAM, AXAM2, LOC78973, mKIAA1331, SMT3IP2, SUMO/sentrin specific peptidase 2, SUMO specific peptidase 2, SuPr-1
SERBP1	1200009K13RIK, 9330147J08Rik, AL022786, CGI-55, CHD3IP, HABP4L, PAI1 binding, PAI1 MRNA binding, PAI-RBP1, SERPINE1 mRNA binding protein 1
SIRT1	AA673258, S, Si, SIR2, Sir2a, SIR2alpha, SIR2L1, Sir2 α, SIRT, sirtuin 1
SMAD4	AW743858, D18Wsu70, D18Wsu70e, DPC, DPC4, JIP, Madh, MADH4, MYHRS, SMAD family member 4, Smaug1
SNCA	AD AMYLOID, al, alp, alphaSYN, alpha SYNUCLEIN, ASYN, NACP, PARK1, PARK4, PD1, synuclein alpha, synuclein, alpha, Synuclein-α, synuclein, α, α-Syn, α SYNUCLEIN
SP1	1110003E12RIK, AA450830, AI845540, Sp1-1, Sp1 transcription factor, Sp1 (trans spliced isoform), Trans-acting transcription factor 1
SP100	A430075G10Rik, lysp100b, SP100 nuclear antigen, Speckled 100 kDa
SP3	D130027J01RIK, Sp3 transcription factor, SPR2, trans-acting transcription factor 3
STUB1	0610033N24Rik, 2210017D18Rik, 2310040B03RIK, AW046544, CH, CHIP, E3 U box, HSPABP2, NY-CO-7, SCA48, SCAR16, SDCCAG7, STIP1 homology and U-Box containing protein 1, UBOX1
TDG	E130317C12Rik, EG545124, Gm5806, hTDG, Jza, Jza1, JZA-3, Tdg-ps, Thymine-DNA glycosylase, thymine DNA glycosylase, pseudogene
TP53	bbl, BCC7, bfy, bhy, BMFS5, LFS1, p4, p44, p5, P53, P53 cellular tumour antigen, p53 tumor suppressor, transformation related protein 53, TRP53, tumor protein p53, tumour protein p53
UBA2	A, AA986091, ARX, HRIHFB2115, LOC100505842, SA, SAE2, UBA, UBA1, ubiquitin-like modifier activating enzyme 2, Ubl1a2, Uble1, UBLE1B
UBE2I	5830467E05Rik, C358B7.1, F830028O17RIK, P18, SUMO E2, UBC9, UbcE2A, Ubce2i, Ubce9, ubiquitin-conjugating enzyme E2I
XIAP	1110015C02RIK, A, Aipa, API3, APOPTOSIS INHIBITOR3, Bir, BIRC4, hIAP-3, I, IAP-3, IL, ILP-1, MIHA, riap3, Xiap-4, X-linked inhibitor of apoptosis, XLP2
ZEB1	3110032K11Rik, ARE, AREB6, BZP, [delta]E, DELTAEF1, [delta]EF1, FECD6, LOC100996668, MEB1, N, Nil2, NIL2A, PPCD3, Tcf18, TCF-8, TCP8, TF8, TRANSCRIPTION FACTOR 8, Tw, ZEB, Zfhe, ZFHEP, Zfhep2, Zfhx1, ZFHX1A, Zfx1, Zfx1a, Zfx1ha, zinc finger E-box binding homeobox 1, [δ]E, [δ]EF1
Zfp217	4933431C08Rik, AW987152, Gm562, ZABC1, Zfp217, ZINC FINGER protein 217

Pathway Analysis Using IPA Software; canonical pathway

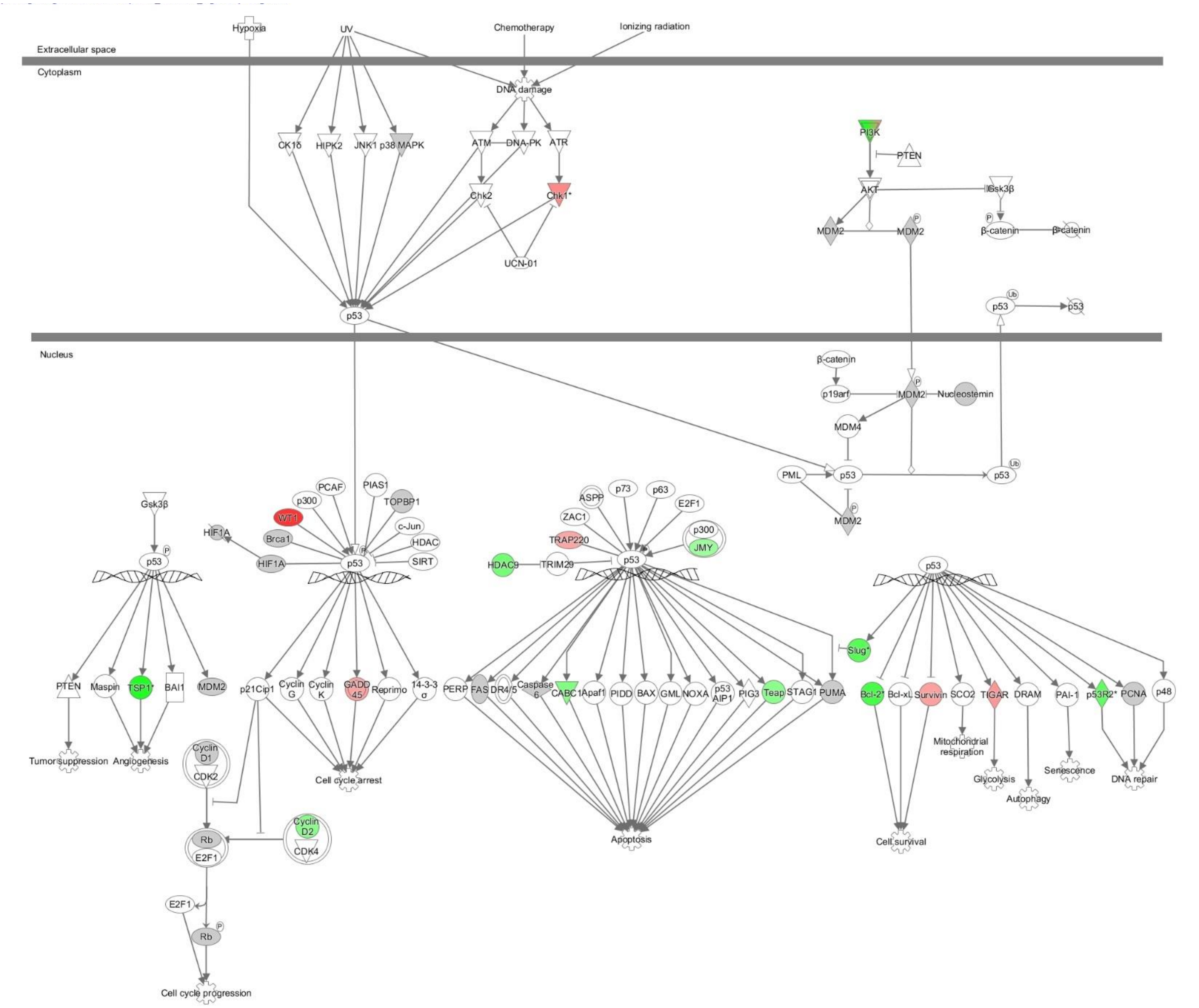

Figure S29. p53 Signaling at 8 days

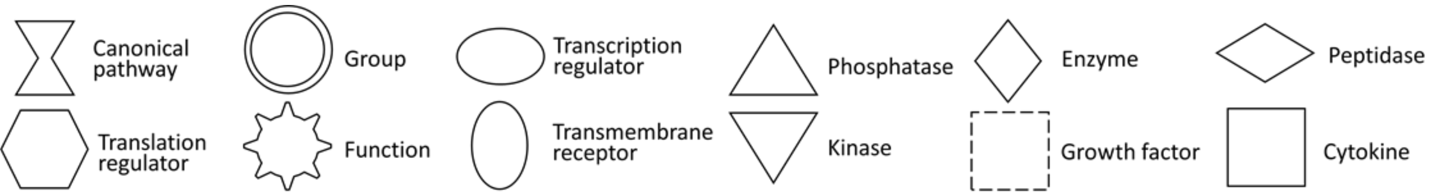

Red: Increased, FDR<0.05 versus solvent control

Green: Decreased, FDR<0.05 versus solvent control

Symbol	Synonym(s)
ADGRB1	adhesion G protein-coupled receptor B1, B830018M07Rik, Ba, BAI1, Brain-specific angiogenesis inhibitor 1, GDAIF, mKIAA4089, R75078
AKT	AKT1/2/3, B/Akt, PKB, RAC-PK
APAF1	6230400I06RIK, Ap, Apaf1l, apoptotic peptidase activating factor 1, CED4, fog, mKIAA0413
ATM	AI256621, AT1, ATA, ataxia telangiectasia mutated, ATC, ATD, ATDC, ATE, ATM serine/threonine kinase, C030026E19RIK, TEL1, TELO1
ATR	ataxia telangiectasia and Rad3 related, Ataxia-telangiectasia-like, ATR serine/threonine kinase, FCTCS, FRP1, LOC100365674, LOC367198, LOC684113, MEC1, SCKL, SCKL1
BAX	Bcl2-associated X, BCL2 associated X, apoptosis regulator, BCL2-associated X protein, BCL2L4
BBC3	BCL2 binding component 3, JFY-1, PU, PUMA, PUMA/JFY1
BCL2	AW986256, B cell leukaemia/lymphoma 2, B cell leukemia/lymphoma 2, Bcl-, Bcl2 alpha, BCL2 apoptosis regulator, BCL2, apoptosis regulator, Bcl2 α, C430015F12Rik, D630044D05RIK, D830018M01RIK, LOC100046608, ORF16, PPP1R50
BCL2L1	bBclxl, Bcl, BCL2L, BCL2-like 1, BCLX, Bcl-X beta, Bclx gamma, BCL-XL/S, Bcl-X β, Bclx γ, PPP1R52
BIRC5	A, AAC-11, AP14, API4, baculoviral IAP repeat-containing 5, EPR-1, s, Survivin, SURVIVIN 2B, T, TIAP
BRCA1	BRCA1 DNA repair associated, BRCA1, DNA repair associated, BRCAI, BRCC1, breast cancer 1, early onset, BROVCA1, FANCS, PNCA4, PPP1R53, PSCP, RNF53
CASP6	caspase 6, LOC103689977, mCAS, MCH2
CCND1	AI327039, B-CELL CLL/LYMPHOMA 1, bcl-, BCL1, cD1, CycD1, CYCLIN D1, Cyl-, Cyl-1, D11S287E, G1/S-Specific Cyclin D1, PR, PRAD1, U21B31
CCND2	2600016F06RIK, AI256817, BF642806, C86853, cD2, cyclin D2, CYL2, KIAK0002, MPPH3, V, Vi, Vin-1
CCNG1	AI314029, CCNG, CYCG, cyclin G1
CCNK	AW123198, AW413594, CP, CPR4, CycK, cyclin K, IDDHDF
CDK2	A630093N05Rik, CDC2-RELATED KINASE, CDKN2, Cyclin A associated kinase, cyclin-dependent kinase 2, CYCLIN E ASSOCIATED KINASE, p33(CDK2)
CDK4	CMM3, Crk, Crk3, cyclin-dependent kinase 4, LOC100362034, PSK-J3
CDKN1A	CAP, CAP20, CDK, CDKI, Cdkn, CDKN1, CDKNA1, CI, CIP1, cyclin-dependent kinase inhibitor 1A, cyclin-dependent kinase inhibitor 1A (P21), mda, MDA-6, P2, P21, p21C, p21Cip, p21CIP1, p21W, p21WAF, p21Waf1, Pzl Cyclin-Dependent Kinase Inhibitor, SD, SDI1, UV96, Waf, WAF1
CHEK1	C85740, checkpoint kinase 1, CHK1, rad27
CHEK2	CDS1, Check2, checkpoint kinase 2, CHK2, hCds1, HUCDS1, LFS2, PP1425, Rad, RAD53
COQ8A	4632432J16Rik, Adck, ADCK3, AI462003, ARCA2, Cabc, CABC1, coenzyme Q8A, COQ10D4, COQ8, LOC100506460, mKIAA0451, SCAR9
CSNK1D	1200006A05Rik, AA409348, ASPS, casein kinase 1 delta, casein kinase 1, delta, casein kinase 1 δ, casein kinase 1, δ, Ck1 delta, CK1δ, CKId, CKI-delta, CKI-δ, D930010H05RIK, FASPS2, HCKID
CTNNB1	armadillo, Beta-cat, beta CATENIN, Bfc, Cat, CATENIN beta, catenin beta 1, catenin (cadherin associated protein), beta 1, catenin (cadherin associated protein), β 1, CATENIN β, catenin β 1, CATNB, CTNB1, CTNNB, CTNN beta, CTNN β, EVR7, Mesc, MRD19, NEDSDV, β-cat, β-catenin
DRAM1	1200002N14Rik, DNA-damage regulated autophagy modulator 1, DRAM, FLJ11259
E2F1	E2f, E2F transcription factor 1, mKIAA4009, RBAP1, RBBP3, RBP3, Tg(Wnt1-cre)2Sor
EP300	A430090G16, A730011L11, E1A binding protein p300, KAT3, KAT3B, MKHK2, p30, p300, p300 HAT, RSTS2
FAS	AI196731, ALPS1A, AP, APO-1, APT1, CD95, CD95L, CD95 receptor, FAS1, FAS/APO1, Fas cell surface death receptor, FasR, FASTM, Fas (TNF receptor superfamily member 6), lpr, Receptor for Fas Ligand, Receptors for Fas Ligand, TNF, Tnfr, TNFR6, Tnf receptor member 6, TNFRSF6
GML	1700057K19Rik, EG625599, glycosylphosphatidylinositol anchored molecule like, glycosylphosphatidylinositol anchored molecule like 2, Gml2, HemT, Hemt1, LY6DL
GNL3	C77032, E2IG3, G protein nucleolar 3, guanine nucleotide binding protein-like 3 (nucleolar), NNP47, NS, NUCLEOSTEMIN, NUG1
GSK3B	7330414F15Rik, 8430431H08Rik, C86142, glycogen synthase kinase 3 beta, glycogen synthase kinase 3 β, GSK-, GSK-3, GSK-3be, GSK-3beta, GSK-3β, GSKbeta, GSKβ, Tpk1
HDAC1	GON-10, HD1, HDAC, Hdac1-ps, histone deacetylase 1, KDAC1, LOC630524, MommeD, MommeD5, RP, RPD3, RPD3L1
HDAC9	AK143298, AV022454, D030072B18, D030072B18Rik, HD, HD7, HD7B, HD9, HDAC, HDAC7, HDAC7B, HDAC9B, HDAC9FL, HDAC C, HDRP, histone deacetylase 9, LOC100504541, LOC100861990, Mi, MITR, mKIAA0744, RGD1310748, RGD1563092
HIF1A	AA959795, bHLHe7, bHLHe78, HIF-1, HIF1-ALPHA, HIF-1alpha (hydroxylated), HIF-1-α, HIF-1α (hydroxylated), Hypoxia inducible factor 1 alpha subunit, hypoxia inducible factor 1, alpha subunit, hypoxia inducible factor 1 subunit alpha, hypoxia inducible factor 1 subunit α, Hypoxia inducible factor 1 α subunit, hypoxia inducible factor 1, α subunit, MO, MOP1, PASD8
HIPK2	1110014O20RIK, B230339E18RIK, homeodomain interacting protein kinase 2, LOC100505582, LOC653052, PRO0593, St, Stank
JMY	AA591059, junction-mediating and regulatory protein, junction mediating and regulatory protein, p53 cofactor, WHAMM2, WHDC1L3
JUN	Activator protein 1, AP-1, API-1, c-ju, cJUN, Junc, jun proto-oncogene, Jun proto-oncogene, AP-1 transcription factor subunit, LOC100288387, LOC100291417, LOC100293034, p39, v-Jun, V-jun Avian Sarcoma Virus 17 Oncogene Homolog, V-jun Sarcoma Virus 17 Oncogene Homolog
KAT2B	A930006P13RIK, AI461839, AW536563, CAF, K(lysine) acetyltransferase 2B, lysine acetyltransferase 2B, Pc, PCAF, P/CAF, Pcaf-b
MAPK14	Crk, CRK1, CSB, CSBP, CSBP1, CSBP2, CSPB1, EXIP, Hog, LOC101929346, MAPK p38, mitogen-activated protein kinase 14, Mxi, Mxi2, p3, p38-, p38a, p38ALPHA, p38Hog, p38 kinase, p38M, p38 MAPK, P38 Map Kinase, p38 MAP kinases alpha and beta, p38 MAP kinases α and β, p38 MAPK-α, p38-α, PRKM14, PRKM15, RK, SAPK2A, SAPK p38 alpha, SAPK p38 α
MAPK8	AI849689, C-JUN N-TERMINAL KINASE1, JNK, JNK1, JNK1A2, JNK21B1/2, JNK-46, mitogen-activated protein kinase 8, p46JNK1, p46JNK1 alpha, p46JNK1 α, Prk, PRKM8, SAPK1, SAPK1c, Sapk gamma, SAPK P46, Sapk γ, STRESS-ACTIVATED protein KINASE-LIKE KINASE
MDM2	1700007J15Rik, AA415488, ACTFS, hdm2, HDMX, LSKB, MDM2-A1, MDM2 proto-oncogene, MGC5370, Transformed 3t3 cell double minute 2, transformed mouse 3T3 cell double minute 2
MDM4	4933417N07RIK, AA414968, AL023055, AU018793, AU021806, BMFS6, C85810, HDMX, LOC102633382, MDM4 regulator of p53, MDMX, MRP1, transformed mouse 3T3 cell double minute 4
MED1	AI480703, ARC205, CRSP, CRSP1, CRSP200, CRSP205, CRSP210, DRIP, DRIP205, DRIP230, I11Jus, I11Jus15, Med220, mediator complex subunit 1, P, PBP, PPARBP, PPARGBP, RB18A, RGD1559552, TRAP, TRAP220, TRIP-2
p19 Arf	A, Arf, ARF-INK4a, CDK4I, CDKN2, CMM2, CYCLIN-DEPENDENT KINASE INHIBITOR 2A, INK4, INK4A, INK4a-ARF, Ink4a/Arf, MLM, MTS, MTS-1, p1, p14ARF/ p16INK4a, p16, p16/ARF, p16Cdkn2a, p16I, p16 INK4, p16/ INK4a, P19, p19ARF, Pct, PCTR1, TP16
PCNA	ATLD2, Pcna/cyclin, PCNAR, proliferating cell nuclear antigen
PERP	1110017A08Rik, dJ496H19.1, ineligibleperp, KCP, KCP1, KRTC, KRTCAP1, p53 apoptosis-associated target, p53 apoptosis effector related to PMP22, PERP, TP53 apoptosis effector, PIG, PIGPC1, THW
PI3K	1-phosphatidylinositol 3-kinase, 2.7.1.137, ATP:1-phosphatidyl-1D-myo-inositol 3-phosphotransferase, Phosphatidylinositol 3 kinase, phosphatidylinositol 3'-kinase, PI3-kinase, PtdIns 3 Kinase, type III phosphoinositide 3-kinase, type I phosphatidylinositol kinase, Vps34p
PIAS1	2900068C24Rik, Ddxbp, DDXBP1, GB, GBP, GU/RH-II, protein inhibitor of activated STAT 1, protein inhibitor of activated STAT, 1, ZMIZ3
PIDD1	1200011D09Rik, AU042446, LOC100911519, Lr, LRDD, p53-induced death domain protein 1, p53-induced protein with a death domain-like, Pi, PIDD
PLAGL1	2610311E24R, 2610311E24Rik, LOT1, PLAG1 like zinc finger 1, pleiomorphic adenoma gene-like 1, ZAC, ZAC1
PMAIP1	APR, N, NOXA, phorbol-12-myristate-13-acetate-induced protein 1
PML	1200009E24Rik, AI661194, PML nuclear body scaffold, PP8675, promyelocytic leukaemia, promyelocytic leukemia, RGD1562602, RNF71, Trim, TRIM19
PRKDC	AI326420, AU019811, DNA-, DNA-DEPENDENT protein KINASE, DNAPDcs, DNAPK, DNA-PKC, DNA-PKcs, DNPK1, DOX, DOXNPH, dxn, dxnph, HYRC, HYRC1, IMD26, p350, p460, Prkdc predicted, protein kinase, DNA activated, catalytic polypeptide, protein kinase, DNA-activated, catalytic subunit, scid, slip, XRCC, XRCC7
PTEN	10q23del, 2310035O07RIK, A130070J02Rik, AI463227, B430203M17RIK, BZS, CWS1, DEC, GLM2, MHAM, MMAC, MMAC1, MUTATED IN MULTIPLE ADVANCED CANCERS, mutated in multiple advanced cancers 1, phosphatase and tensin homolog, PTEN1, PTENbeta, TEP, TEP1
RB1	OSRC, p, p105, p105-Rb, p110 RB, p110-RB1, pp105, pp110, PPP1R130, pRb, R, RB, RB-ASSOCIATED, RB transcriptional corepressor 1, Retinoblastome tumor-suppression protein rb
Rprm	2410012A13Rik, Re, REPRIMO, reprimo, TP53 dependent G2 arrest mediator candidate, reprimo, TP53 dependent G2 arrest mediator homolog
RRM2B	MGC42116, MTDPS8A, MTDPS8B, P53R2, Ribonucleotide Reductase M2 B (TP53 Inducible), ribonucleotide reductase regulatory TP53 inducible subunit M2B
SCO2	CEMCOX1, ECGF1, Gliostatin, MC4DN2, MYP6, PD-ECGF, SCO1L, SCO2 cytochrome c oxidase assembly protein, synthesis of cytochrome C oxidase 2, TdRPase, TP, TYMP
SERPINB5	1110036M19RIK, AI462524, AI646751, M, Maspin, ova, ovalbumin, PI-5, S, serine (or cysteine) peptidase inhibitor, clade B, member 5, serpin family B member 5, Spi7
SERPINE2	B230326M24Rik, CRG, GDN, GDNPF, GDNPN1, GLIA DERIVED NEXIN, ne, NEXIN1, P, PAI-1, PI-7, PN, PN-1, PNI, PROTEASE NEXIN1, S, serine (or cysteine) peptidase inhibitor, clade E, member 2, Serpin-4, serpin family E member 2, Spi4, Spin4
SFN	14-3-3, 14-3-3 Sigma, 14-3-3 σ, E, ER, HME1, Mme1, Stratifin, Ywh, YWHAS
SIRT1	AA673258, S, Si, SIR2, Sir2a, SIR2alpha, SIR2L1, Sir2 α, SIRT, sirtuin 1
SNAI2	S, Sl, Slu, SLUG, SLUGH, SLUGH1, SNAIL2, snail family transcriptional repressor 2, snail family zinc finger 2, WS2D
ST13	1110007I03RIK, 3110002K08RIK, AAG2, AW555194, FAM10A1, FAM10A4, HIP, HOP, HSP, HSPABP, HSPABP1, NA+,K+ atpase 1, p4, P48, PRO0, PRO0786, SN, SNC6, ST13 Hsp70 interacting protein, ST13, Hsp70 interacting protein, suppression of tumorigenicity 13, suppression of tumourigenicity 13
STAG1	AU045003, COHESIN subunit SA-1, MRD47, SA, SA-1, Scc, Scc3, SCC3A, Stromal antigen 1
THBS1	tbs, tbsp1, THBS, thrombospondin 1, THSP, TS, TS-1, TSP, TSP-1
TIGAR	9630033F20Rik, AA793651, AI595337, C12orf5, C79710, C85509, FR2BP, Ti, TP53 induced glycolysis regulatory phosphatase, Trp53 induced glycolysis regulatory phosphatase
TOPBP1	1110031N14RIK, 2810429C13Rik, AI256758, D430026L04Rik, DNA topoisomerase II binding protein 1, Dpb11, mKIAA0259, RGD1562949, TOP2BP1, TOPO2 BP, TOPOISOMERASE2 binding, topoisomerase (DNA) II binding protein 1
TP53	bbl, BCC7, bfy, bhy, BMFS5, LFS1, p4, p44, p5, P53, P53 cellular tumour antigen, p53 tumor suppressor, transformation related protein 53, TRP53, tumor protein p53, tumour protein p53
TP53AIP1	P53AIP1, tumor protein p53 regulated apoptosis inducing protein 1, tumour protein p53 regulated apoptosis inducing protein 1
TP53I3	PIG3, tumor protein p53 inducible protein 3, tumour protein p53 inducible protein 3
TP53INP1	2700057G22Rik, DKFZp434M1317, p53DINP1, S, SI, SIP, STINP, Te, Teap, Thymus Expressed Acidic, TP53DINP1, TP53DINP1alpha, TP53INP1A, TP53INP1B, transformation related protein 53 inducible nuclear protein 1, Trp53inp1, tumor protein p53 inducible nuclear protein 1, tumour protein p53 inducible nuclear protein 1
TP63	AI462811, AIS, B(p51A), B(p51B), delta, Delta N p63, Delta N p63 Alpha, EEC3, KET, LMS, NBP, OFC8, p51/p, P51/P63, p53CP, p6, p63, p63 alpha isoform, p63 gamma isoform, p63/p40, p63 α isoform, p63 γ isoform, p7, p73H, P73l, RHS, SHFM4, TAp, TAp63, TP53CP, TP53L, TP73L, transformation related protein 63, Trp5, Trp53rp1, Trp63, TUMOR protein 63 KDA with STRONG HOMOLOGY TO P53, tumor protein p63, tumour protein 63 KDA with STRONG HOMOLOGY TO P53, tumour protein p63, δ, δ N p63, δ N p63 α
TRIM29	1110047J21Rik, 2810431N19Rik, 4732461M22Rik, AI119726, ATDC, tripartite motif-containing 29
Trp73	delta, p7, P73, p73RhoGAP, TAp, transformation related protein 73, Trp73, tumour protein p73, tumour protein p73, δ
UCN-01	112953-11-4, (2S,3R,4R,6R,18R)-18-hydroxy-3-methoxy-2-methyl-4-(methylamino)-29-oxa-1,7,17-triazaoctacyclo[12.12.2.12,6.07,28.08,13.015,19.020,27.021,26]nonacosa-8,10,12,14,19,21,23,25,27-nonaen-16-one, 7-hydroxystaurosporine, 8,12-epoxy-1H,8H-2,7b,12a-triazadibenzo(a,g)cyclonona(cde)triinden-1-one, 2,3,9,10,11,12-hexahydro-3-hydroxy-9-methoxy-8-methyl-10-(methylamino)-, C28H26N4O4, KW-2401, NSC-638850
WT1	D630046I19RIK, GUD, NPHS4, WAGR, Wilms tumor 1 homolog, wilms' tumour, wilms tumour 1, Wilms tumour 1 homolog, Wilms tumour-suppressor, Wilms' tumour suppressor, WIT-2, WT, WT1 transcription factor, WT33

Pathway Analysis Using IPA Software; canonical pathway

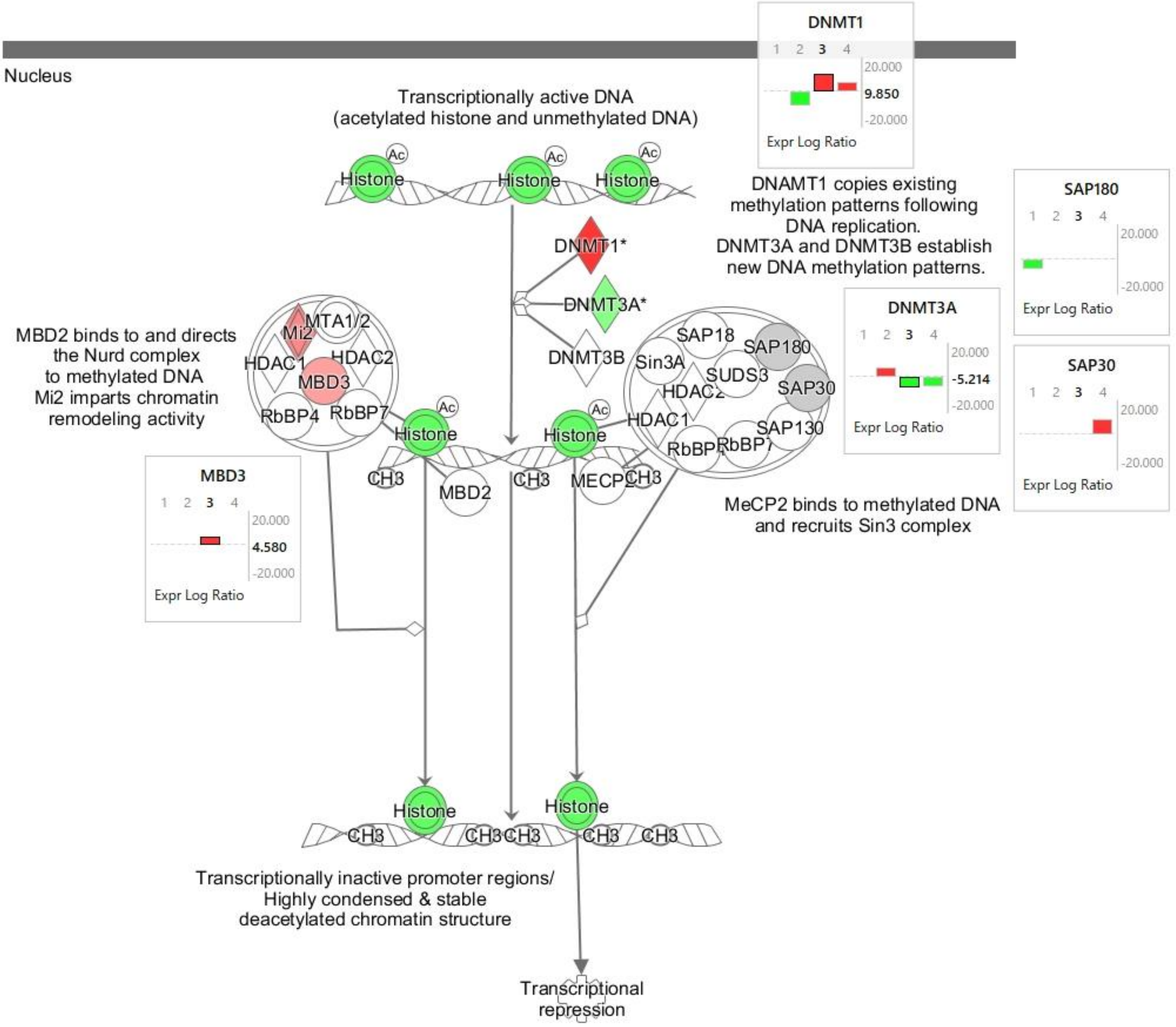

Figure S30. DNA Methylation and Transcriptional Repression Signaling
1. 1 h; 2. 6 h; 3. 24 h; 4. 8 days.

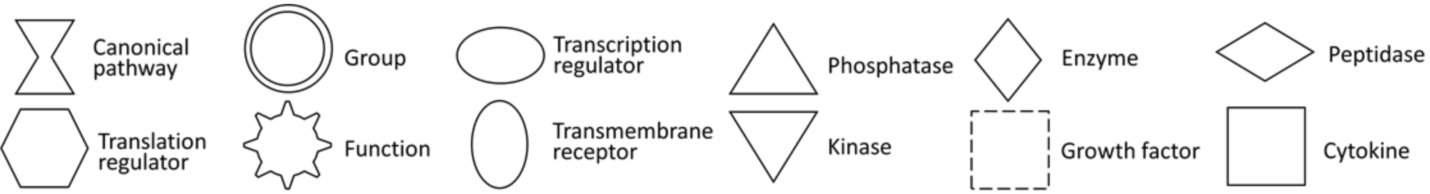

Red: Increased, FDR<0.05 versus solvent control

Green: Decreased, FDR<0.05 versus solvent control

Symbol	Synonym(s)
ARID4B	AT-rich interaction domain 4B, AT rich interactive domain 4B (RBP1-like), AT-rich interactive domain-containing protein 4B-like, BCAA, BRC, BRCAA1, LOC100912163, RBBP, RBBP1L1, Rbp, RBP1L1, RBP1-LIKE, SAP, SAP180
DNMT1	ADCADN, AIM, CXXC9, DMT, DNA methyltransferase 1, DNA methyltransferase (cytosine-5) 1, DNA MTASE, DNA MTase HsaI, DNMT, HSN1E, MCMT, Met-1, m.HsaI, m.MmuI, MommeD, MommeD2, MTa, MTase
DNMT3A	DNA Methyltransferase 3A, DNA methyltransferase 3 alpha, DNA methyltransferase 3 α, DNA MTase HsaIIIA, DNMT3A2, HESJAS, M.HsaIIIA, MmuIIIA, TBRS
DNMT3B	DNA Methyltransferase 3B, DNA methyltransferase 3 beta, DNA methyltransferase 3 β, DNA MTase HsaIIIB, ICF, ICF1, M.HsaIIIB, MmuIIIB
HDAC1	GON-10, HD1, HDAC, Hdac1-ps, histone deacetylase 1, KDAC1, LOC630524, MommeD, MommeD5, RP, RPD3, RPD3L1
HDAC2	D10Wsu179, D10Wsu179e, HD2, histone deacetylase 2, KDAC2, mRPD3, RGD: 619976, RPD3, YAF1, Yy1b, Yy1bp
HISTONE	HISTONES
MBD2	DMTase, LOC684150, MBD2a, methyl-CpG binding domain protein 2, NY-CO-41
MBD3	AI181826, AU019209, methyl-CpG binding domain protein 3
MECP2	1500041B07Rik, AUTSX3, D630021H01RIK, Mbd5, Mecp2 Beta, Mecp2 β, methyl-CpG binding protein 2, MRX16, MRX79, MRXS13, MRXSL, PPMX, RS, RTS, RTT, WBP1, WBP10
Mi2	Mi-2 alpha/beta, Mi-2 α/β
NuRD	Mi2-NuRD
RBBP4	CAF1 P48, CAF1/p48, CAF-1 subunit C, CAF-I 48 kDa subunit, lin-53, LOC681419, LOC685491, mRbAp48, NURF55, p46/48, RBA, RBAP48, Rb-associated protein p48, RB binding protein 4, chromatin remodeling factor, retinoblastoma binding protein 4, chromatin remodeling factor, YQ51D06
RBBP7	AA409861, AI173248, AU019541, BB114024, mRbAp46, RbAp46, Rb-associated protein p46, RBB7, RB binding protein 7, chromatin remodeling factor, retinoblastoma binding protein 7, chromatin remodeling factor
SAP130	2610304F09RIK, 6720406D06, RGD1311657, Sin3A associated protein, Sin3A associated protein 130
SAP18	2HOR0202, C530046K05Rik, D11Ertd539, D11Ertd539e, EMegR, EMegR4, Gm10094, RGD1561590, Sa, Sap18a, Sap18b, SAP18P, Si, similar to SAP18, Sin3A associated protein 18, Sin3-associated polypeptide 18, Sin3-associated polypeptide 18B, Sinbp1
SAP30	Sin3A associated protein 30, sin3 associated polypeptide
SIN3A	AW553200, mKIAA4126, mS, MSIN3A, S, SIN3, SIN3 transcription regulator family member A, transcriptional regulator, SIN3A (yeast), WITKOS
SUDS3	2400003N08RIK, 2410008L21Rik, AU067672, LOC105378257, mSds3, RGD1305986, SAP45, SDS3, SDS3 homolog, SIN3A corepressor complex component, suppressor of defective silencing 3 homolog (S. cerevisiae)

Table S31. DNA methylation

Gene Symbol	Gene Title	Probe_ID	1 h		6 h		24 h		8 days	
			FDR increase	FDR decrease	FDR increase	FDR decrease	FDR increase	FDR decrease	FDR increase	FDR decrease
Dnmt1	DNA methyltransferase (cytosine-5) 1	1422946_a_at	1.1810	1.3288	1.0094	0.0068	0.0011	1.0017	0.2069	1.0211
		1435122_x_at	0.6479	0.9653	1.0076	0.0043	0.0033	1.0040	0.0944	1.0170
		1447877_x_at	1.1074	1.3172	1.0088	0.0057	0.0094	1.0077	0.0265	1.0081
Dnmt3a	DNA methyltransferase 3A	1423063_at	0.9960	0.9920	0.0331	1.0213	1.0227	0.0602	1.0278	0.0912
		1423064_at	1.0156	0.9159	0.7573	1.1016	1.3930	1.3869	0.9028	1.1325
		1423065_at	1.1675	1.3113	0.0331	1.0213	1.0086	0.3005	1.0214	0.0518
		1423066_at	1.1132	1.3032	0.0294	1.0200	1.0693	0.1280	1.0575	0.1882
		1460324_at	1.1477	1.0148	0.0494	1.0265	1.0129	0.0269	1.0196	0.0454
Dnmt3b	DNA methyltransferase 3B	1418351_a_at	1.2163	0.9903	1.0438	0.1238	1.1472	1.3373	0.9622	1.1789
		1442655_at	1.1603	0.9688	1.1114	1.1061	1.0355	1.3452	0.8646	1.1652
		1449052_a_at	0.3989	0.9673	1.0380	0.0991	1.2969	1.2444	1.1440	1.2240
Dnmt3l	DNA (cytosine-5-)-methyltransferase 3-like	1425035_s_at	1.0413	1.0820	1.0638	1.3174	1.0121	1.3655	1.0853	1.2078
Sap130	Sin3A associated protein	1428857_at	1.1612	0.9657	1.0675	1.3788	1.1763	1.0828	1.1387	1.2193
Sap18	Sin3-associated polypeptide 18	1419443_at	1.0219	1.0343	1.0660	1.3713	0.5996	1.0178	1.1598	1.0619
		1449480_at	1.1917	0.9732	1.0645	1.3695	0.3978	1.0732	1.0724	1.2128
Sap30	sin3 associated polypeptide	1417719_at	0.3031	0.9775	0.3017	1.0809	0.0628	1.0235	0.0028	1.0029
Sap30bp	SAP30 binding protein	1418977_at	1.1168	1.0043	1.0328	1.1058	1.3403	1.0286	1.1004	1.2185
		1449295_at	0.9673	1.1825	1.1455	1.1613	1.0277	1.3298	0.9110	1.1259

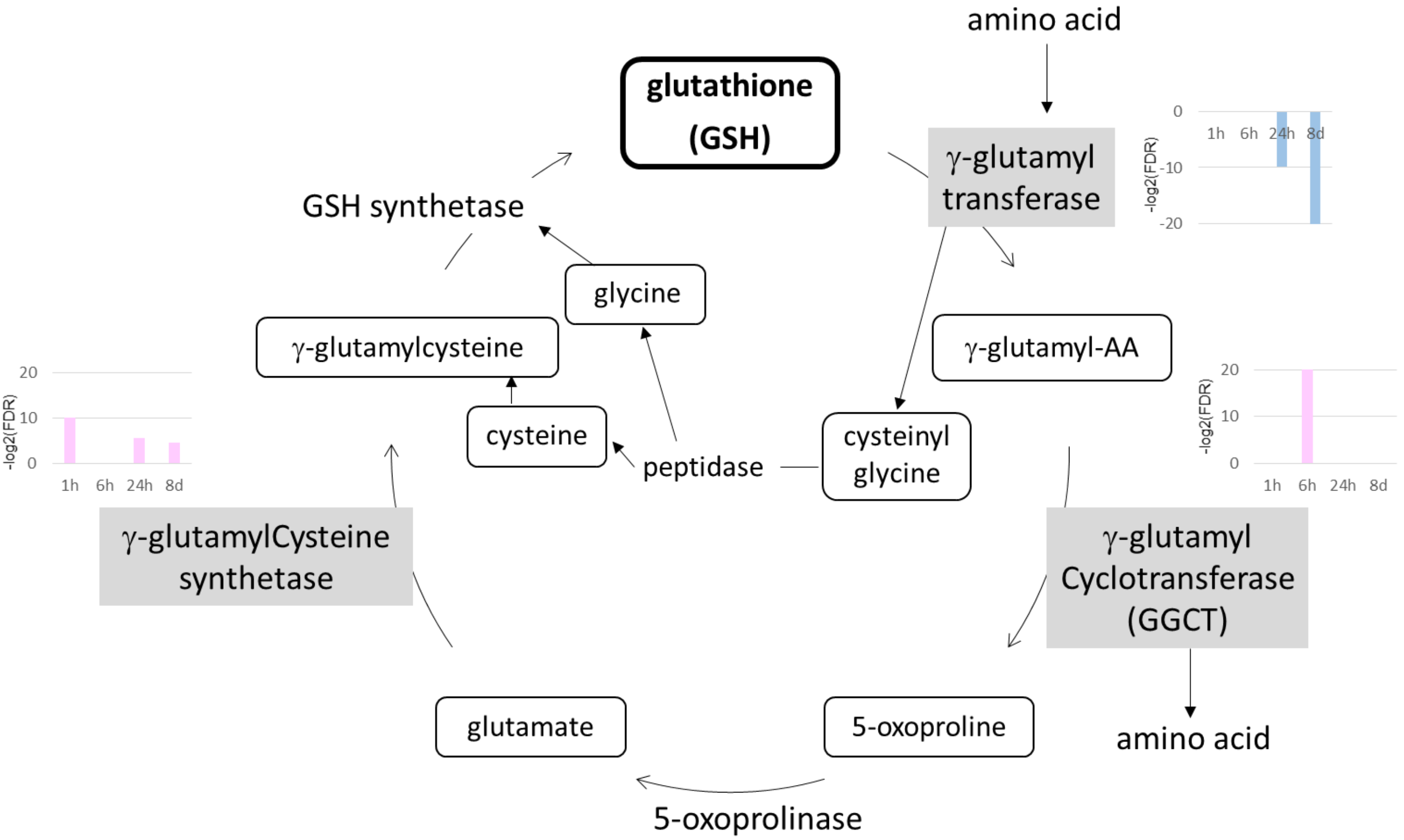

Figure S32. Glutamyl Cycle
8d. 8 days.

Table S33. Gene expression variation associated with cytochromes P450

Gene symbol		1 h	6 h	24 h	8 days
Cyp1a1	cytochrome P450, family 1, subfamily a, polypeptide 1	↑ (1422217_a_at)			
Cyp1b1	cytochrome P450, family 1, subfamily b, polypeptide 1	↑ (1416612_at)	↓ (1416612_at) (1416613_at)		
Cyp26b1	cytochrome P450, family 26, subfamily b, polypeptide 1	↓ (1460011_at)			↓ (1460011_at)
Cyp39a1	cytochrome P450, family 39, subfamily a, polypeptide 1			↓ (1418780_at)	

↑ : Increased, FDR<0.05 versus solvent control
↓ : Decreased, FDR<0.05 versus solvent control
( ) : Probe ID

Pathway Analysis Using IPA Software; canonical pathway

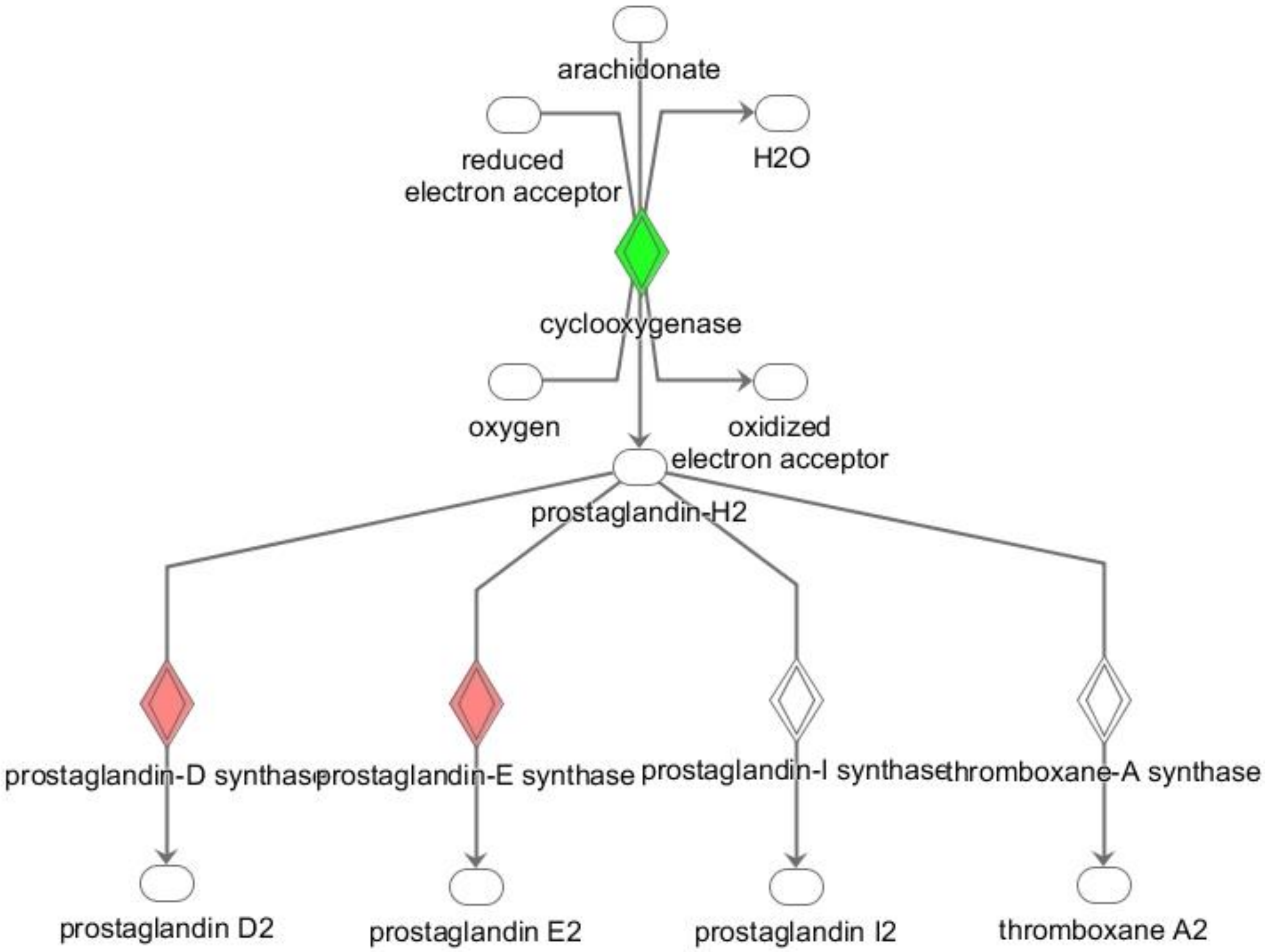

Figure S34. Prostanodid Biosynthesis at 1 h

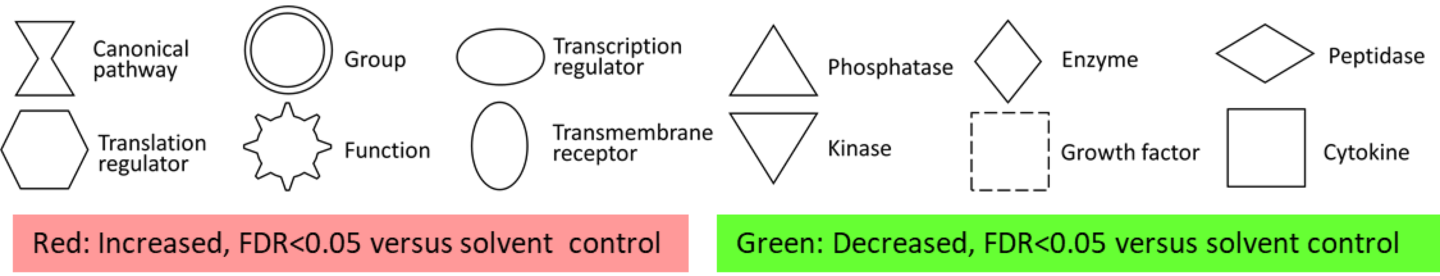

Symbol	Synonym(s)
arachidonate	20:4n-6, 506-32-1, 5,8,11,14-eicosatetraenoic acid, (all-Z)-, 5Z,8Z,11Z,14Z-arachidonic acid, (5Z,8Z,11Z,14Z)-icosa-5,8,11,14-tetraenoic acid, AA, AA-d8, ARA, arachidonate, C20:4(n-6), C20:4w6, C20H32O2, eicosa-5Z,8Z,11Z,14Z-tetraenoic acid, sodium arachidonate
cyclooxygenase	1.14.99.1, (5Z,8Z,11Z,14Z)-icosa-5,8,11,14-tetraenoate,hydrogen-donor:oxygen oxidoreductase, COX, COX1/2, fatty acid cyclooxygenase, PGHS, (PG)H synthase, PG synthetase, Prostaglandin-endoperoxide synthase, prostaglandin endoperoxide synthetase, prostaglandin G/H synthase, prostaglandin G/H synthase and cyclooxygenase, Prostaglandin h synthase, Prostaglandin Peroxidase, prostaglandin synthase, prostaglandin synthetase, PTGS, PTGS1/2
prostaglandin D2	11-dehydroprostaglandin F2-alpha, 11-dehydroprostaglandin F2-α, 41598-07-6, (5Z,13E)-9alpha-hydroxy-11,15-dioxoprosta-5,13-dienoate, (5Z,13E)-9alpha-hydroxy-11,15-dioxoprosta-5,13-dienoic acid, C20H32O5, PGD2, prosta-5,13-dien-1-oic acid, 9,15-dihydroxy-11-oxo-, (5Z,9-alpha,13E,15S)-, prosta-5,13-dien-1-oic acid, 9,15-dihydroxy-11-oxo-, (5Z,9-α,13E,15S)-, (Z)-7-[(1R,2R,5S)-5-hydroxy-2-[(E,3S)-3-hydroxyoct-1-enyl]-3-oxocyclopentyl]hept-5-enoic acid
prostaglandin E2	363-24-6, (5Z, 11a, 13E, 15S)-11, 15-Dihydroxy-9-oxo-prosta-5, 13-dien-1-oic acid, C20H32O5, Cervidil, dinoprostone, PGE2, Prepidil, Propess, Prostarmon E, Prostin E, Prostin E2, Prostin E2 Vaginal Suppository, (Z)-7-[(1R,2R,3R)-3-hydroxy-2-[(E,3S)-3-hydroxyoct-1-enyl]-5-oxocyclopentyl]hept-5-enoic acid
prostaglandin I2	35121-78-9, (5Z)-5-[(3aR,4R,5R,6aS)-5-hydroxy-4-[(E,3S)-3-hydroxyoct-1-enyl]-3,3a,4,5,6,6a-hexahydrocyclopenta[b]furan-2-ylidene]pentanoic acid, 61849-14-7, C20H32O5, epoprostenol sodium, Flolan, PGI2, PGX, prosta-5,13-dien-1-oic acid, 6,9-epoxy-11,15-dihydroxy-, (5Z,9alpha,11alpha,13E,15S)-, prostacyclin, prostaglandin I, prostaglandin I2, sodium PGI2
prostaglandin-D synthase	(5,13)-(15S)-9alpha,11alpha-epidioxy-15-hydroxyprosta-5,13-dienoate D-isomerase, 5.3.99.2, PGH-PGD isomerase, prostaglandin-H2 D-isomerase, prostaglandin-R-prostaglandin D isomerase
prostaglandin-E synthase	5.3.99.3, (5Z,13E)-(15S)-9alpha,11alpha-epidioxy-15-hydroxyprosta-5,13-dienoate E-isomerase, endoperoxide isomerase, PGE2 isomerase, PGE isomerase, PGH-PGE isomerase, prostaglandin endoperoxide E2 isomerase, prostaglandin endoperoxide E isomerase, prostaglandin-H2 E-isomerase, prostaglandin H-E isomerase, prostaglandin R-prostaglandin E isomerase
prostaglandin-H2	42935-17-1, 9,11-epoxymethano-PGH2, C20H32O5, PGH2, PGH2 endoperoxide, prosta-5,13-dien-1-oic acid, 9,11-epidioxy-15-hydroxy-, (5Z,9alpha,11alpha,13E,15S)-, (Z)-7-[(1R,4S,5R,6R)-6-[(E,3S)-3-hydroxyoct-1-enyl]-2,3-dioxabicyclo[2.2.1]heptan-5-yl]hept-5-enoic acid
prostaglandin-I synthase	5.3.99.4, (5Z,13E)-(15S)-9alpha,11alpha-epidioxy-15-hydroxyprosta-5,13-dienoate 6-isomerase, PGI2 synthase, PGI2 synthetase, prostacycline synthetase, prostacyclin synthase, prostagladin I2 synthetase
thromboxane A2	57576-52-0, 5-heptenoic acid, 7-(3-(3-hydroxy-1-octenyl)-2,6-dioxabicyclo(3.1.1)hept-4-yl)-, (1S-(1alpha,3alpha,3R*),4beta(Z),5alpha)-, C20H32O5, thromboxa-5,13-dien-1-oic acid, 9,11-epoxy-15-hydroxy-, (5Z,9alpha,11alpha,13E,15S)-, TxA2, (Z)-7-[(1S,3R,4S,5S)-3-[(E,3S)-3-hydroxyoct-1-enyl]-2,6-dioxabicyclo[3.1.1]heptan-4-yl]hept-5-enoic acid
thromboxane-A synthase	5.3.99.5, (5Z,13E)-(15S)-9alpha,11alpha-epidioxy-15-hydroxyprosta-5,13-dienoate thromboxane-A2-isomerase, thromboxane synthase

Pathway Analysis Using IPA Software; canonical pathway

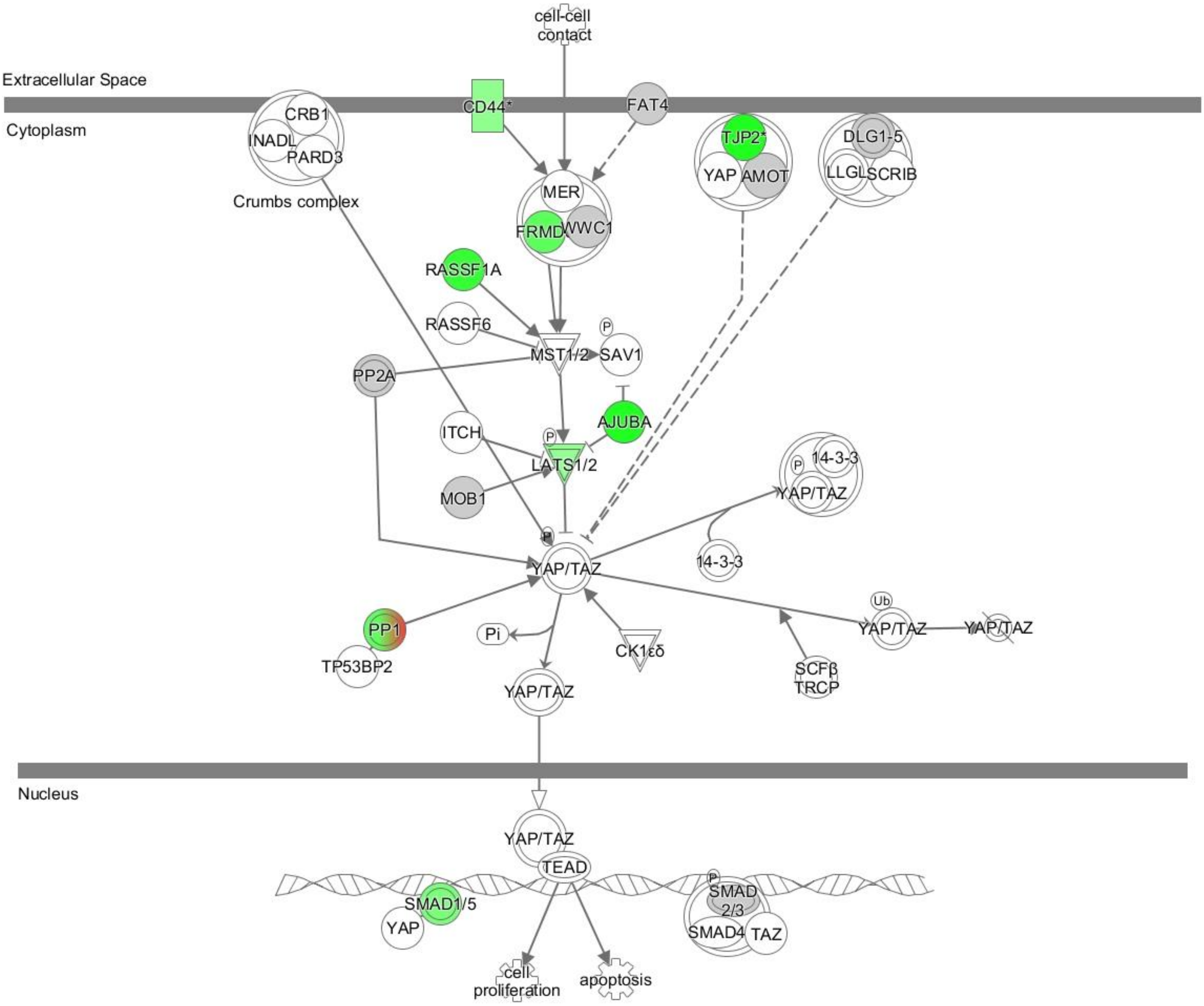

Figure S35. Hippo Signaling at 1 h

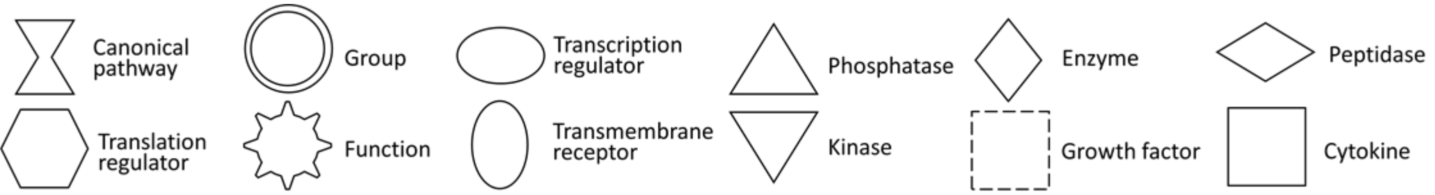

Red: Increased, FDR<0.05 versus solvent control

Green: Decreased, FDR<0.05 versus solvent control

Symbol	Synonym(s)
14-3-3	CBP
AJUBA	ajuba LIM protein, JUB
AMOT	Angiomotin, CAG-2, D0Kis, D0Kist1, RGD1564027, S, SII6
CD44	216062 AT, AU023126, AW121933, AW146109, CD44A, CD44 Antigen, CD44 (containing exon 5), Cd44i, CD44 molecule (Indian blood group), CD44 (soluble), CD44 STANDARD FROM, CDW44, CSPG8, ECMR-III, Epican, HCELL, HERM, HERMES, Hermes antigen, HUTCH-I, IN, LHR, Ly-2, Ly-24, MC56, MDU2, MDU3, METAA, MIC4, NKT.44, Pgp, Pgp-1, RHAMM
CK1ε/CK1δ	CK1 epsilon/CK1 delta, CK1epsilondelta, CK1εδ
CRB1	7530426H14Rik, A930008G09Rik, CRB1-A, CRB1-A2, CRB1-B, CRB1-C, crumbs cell polarity complex component 1, crumbs family member 1, photoreceptor morphogenesis associated, LCA8, RP12
FAT4	6030410K14Rik, 9430004M15, CDHF14, CDHR11, FAT atypical cadherin 4, FAT-J, HKLLS2, NBLA00548, VMLDS2
FRMD6	2610019M19Rik, 4930488L10RIK, AW212977, c14 5320, C14orf31, EX, EX1, FERM domain containing 6, LOC257646, Willin
ITCH	6720481N21Rik, 8030492O04RIK, A, A130065M08, ADMFD, AIF4, AIP4, C230047C07RIK, itchy E3 ubiquitin protein ligase, itchy, E3 ubiquitin protein ligase, NAPP1, Ubiquitin-Protein Ligase
LATS	LATS1/2, LATS 1 and 2
Mob1a	4022402H07Rik, C2orf6, hMOB1, LOC100366014, MATS1, MOB1, MOB4B, Mobk1, MOBK1B, MOB kinase activator 1A, Mobkl, MOBKL1B
MST/KRS	hpo, Mst, MST1/2
NF2	ACN, BANF, me, Merlin, neurofibromatosis 2 tumor suppressor, neurofibromatosis 2 tumour suppressor, neurofibromin 2, SCH, schw, SCHWANNOMIN
PARD3	AA960621, AI256638, ASIP, atypical pkc-specific binding, Baz, D8Ertd580, D8Ertd580e, MPAR3, P, PA, PAR3, PAR3A, PAR3alpha, par-3 family cell polarity regulator, PARD3A, Phip, PPP1R118, SE2-5, SE2-5L16, SE2-5LT1, SE2-5T2
PATJ	C, CIPP, hINADL, I, INADL, Inadl2, InaD-like, PATJ crumbs cell polarity complex component, PATJ, crumbs cell polarity complex component, RGD1564282, RGD1565362
Pi	14265-44-2, inorganic phosphate, O4P-3, P, phosphate, phosphate(3-), phosphate ion, Pi
PP1	any Pp1 (protein phosphatase 1 complex), Atp-Mg2+-Dependent Protein Phosphatase, PP1, Pp1 (protein phosphatase 1 complex), PPA1, Protein Phosphatase 1
PP2A	protein PHOSPHATASE 2A, Protein Phosphatase Type2a
RASSF1A	123F2, AA536941, AU044980, D4Mgi37, NO, NORE2A, Ra, Ras, Ras association domain family member 1, Ras association (RalGDS/AF-6) domain family member 1, Rassf1A, Rassf1B, Rassf1C, RDA, RDA32, REH3P, REH3P21
RASSF6	1600016B17Rik, Ras association domain family member 6, Ras association (RalGDS/AF-6) domain family member 6
SAV1	1700040G09Rik, Salv, salvador family WW domain containing 1, salvador family WW domain containing protein 1, SAV, WW45, Wwp3, WWP4
ScfTrcpbeta	SCFbeta TRCP, Scf Trcp β, SCFβ TRCP
SCRIB	AI118201, Cr, CRC, CRIB, CRIB1, KIAA0147, mKIAA0147, RGD1565055, Scr, SCRB1, SCRIB1, scribbled planar cell polarity, scribble planar cell polarity, scribble planar cell polarity protein, Vartul
Smad2/3-Smad4	Smad 2/3/4
SMAD4	AW743858, D18Wsu70, D18Wsu70e, DPC, DPC4, JIP, Madh, MADH4, MYHRS, SMAD family member 4, Smaug1
TEAD	TEA domain family
TJP2	C9DUPq21.11, DFNA51, DUP9q21.11, PFIC4, tight junction protein 2, X104, ZO-, ZO-2
TP53BP2	53BP, 53BP2, A, AI746547, ASPP2, p53-Binding, P53BP2, PPP1R13A, transformation related protein 53 binding protein 2, Trp53bp2, tumor protein p53 binding protein 2, tumor protein p53 binding protein, 2, tumour protein p53 binding protein 2, tumour protein p53 binding protein, 2, X98550
WWC1	AA408228, AU017197, BC037006, HBEBP3, HBEBP36, Ki, KIAA0869, KIBRA, MEMRYQTL, PPP1R168, RGD1308329, WW and C2 domain containing 1, WW, C2 and coiled-coil domain containing 1
Wwtr1	2310058J06Rik, 2610021I22Rik, C78399, DKFZp586I1419, TA, TAZ, TRANSCRIPTIONAL COACTIVATOR with PDZ-binding MOTIF, WW domain containing transcription regulator 1
YAP/TAZ	YAP/WWTR1, YAP/WWTRT1
YAP1	AI325207, COB1, Y, YAP, Yap2, YAP65, Yes1 associated transcriptional regulator, yes-associated protein 1, Yk, YKI, yor, Yorkie

Pathway Analysis Using IPA Software; canonical pathway

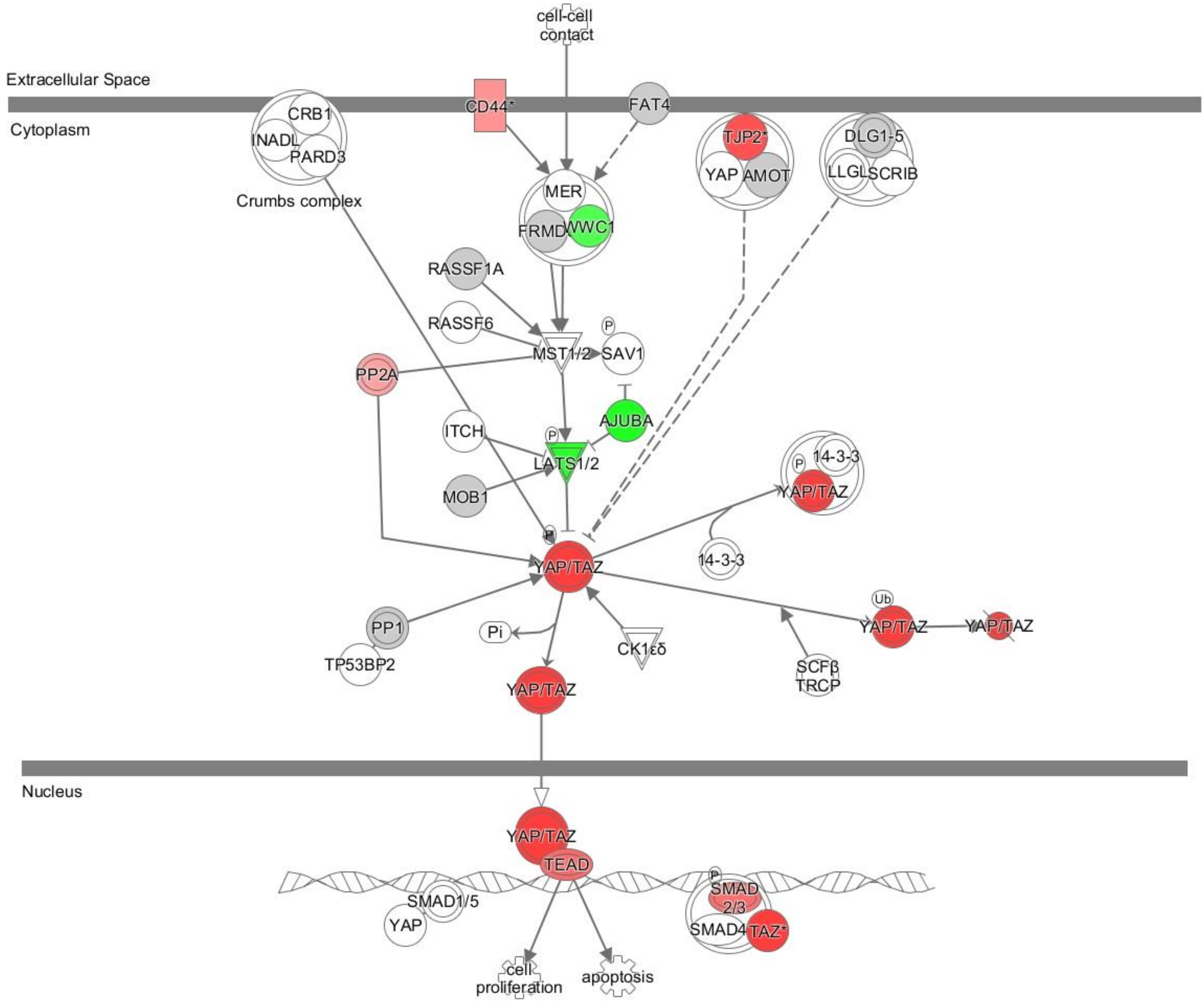

Figure S36. HIPPO Signaling at 6 h

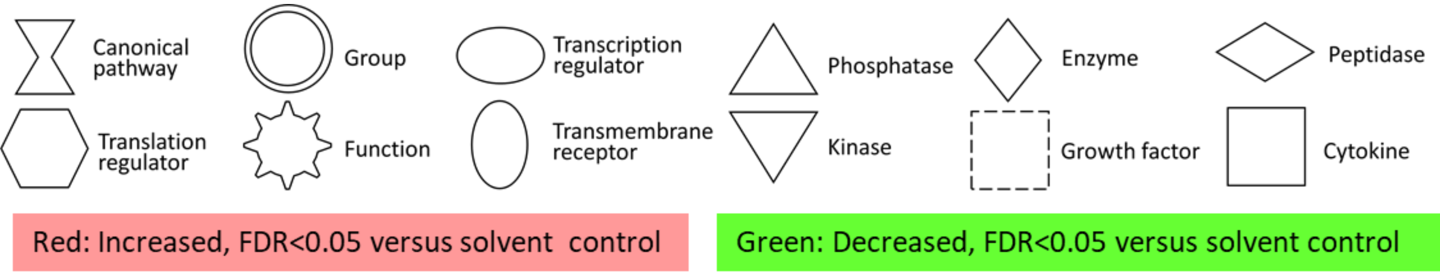

Symbol	Synonym(s)
14-3-3	CBP
AJUBA	ajuba LIM protein, JUB
AMOT	Angiomotin, CAG-2, D0Kis, D0Kist1, RGD1564027, S, SII6
CD44	216062 AT, AU023126, AW121933, AW146109, CD44A, CD44 Antigen, CD44 (containing exon 5), Cd44i, CD44 molecule (Indian blood group), CD44 (soluble), CD44 STANDARD FROM, CDW44, CSPG8, ECMR-III, Epican, HCELL, HERM, HERMES, Hermes antigen, HUTCH-I, IN, LHR, Ly-2, Ly-24, MC56, MDU2, MDU3, METAA, MIC4, NKT.44, Pgp, Pgp-1, RHAMM
CK1ε/CK1δ	CK1 epsilon/CK1 delta, CK1epsilondelta, CK1εδ
CRB1	7530426H14Rik, A930008G09Rik, CRB1-A, CRB1-A2, CRB1-B, CRB1-C, crumbs cell polarity complex component 1, crumbs family member 1, photoreceptor morphogenesis associated, LCA8, RP12
FAT4	6030410K14Rik, 9430004M15, CDHF14, CDHR11, FAT atypical cadherin 4, FAT-J, HKLLS2, NBLA00548, VMLDS2
FRMD6	2610019M19Rik, 4930488L10RIK, AW212977, c14 5320, C14orf31, EX, EX1, FERM domain containing 6, LOC257646, Willin
ITCH	6720481N21Rik, 8030492O04RIK, A, A130065M08, ADMFD, AIF4, AIP4, C230047C07RIK, itchy E3 ubiquitin protein ligase, itchy, E3 ubiquitin protein ligase, NAPP1, Ubiquitin-Protein Ligase
LATS	LATS1/2, LATS 1 and 2
Mob1a	4022402H07Rik, C2orf6, hMOB1, LOC100366014, MATS1, MOB1, MOB4B, Mobk1, MOBK1B, MOB kinase activator 1A, Mobkl, MOBKL1B
MST/KRS	hpo, Mst, MST1/2
NF2	ACN, BANF, me, Merlin, neurofibromatosis 2 tumor suppressor, neurofibromatosis 2 tumour suppressor, neurofibromin 2, SCH, schw, SCHWANNOMIN
PARD3	AA960621, AI256638, ASIP, atypical pkc-specific binding, Baz, D8Ertd580, D8Ertd580e, MPAR3, P, PA, PAR3, PAR3A, PAR3alpha, par-3 family cell polarity regulator, PARD3A, Phip, PPP1R118, SE2-5, SE2-5L16, SE2-5LT1, SE2-5T2
PATJ	C, CIPP, hINADL, I, INADL, Inadl2, InaD-like, PATJ crumbs cell polarity complex component, PATJ, crumbs cell polarity complex component, RGD1564282, RGD1565362
Pi	14265-44-2, inorganic phosphate, O4P-3, P, phosphate, phosphate(3-), phosphate ion, Pi
PP1	any Pp1 (protein phosphatase 1 complex), Atp-Mg2+-Dependent Protein Phosphatase, PP1, Pp1 (protein phosphatase 1 complex), PPA1, Protein Phosphatase 1
PP2A	protein PHOSPHATASE 2A, Protein Phosphatase Type2a
RASSF1A	123F2, AA536941, AU044980, D4Mgi37, NO, NORE2A, Ra, Ras, Ras association domain family member 1, Ras association (RalGDS/AF-6) domain family member 1, Rassf1A, Rassf1B, Rassf1C, RDA, RDA32, REH3P, REH3P21
RASSF6	1600016B17Rik, Ras association domain family member 6, Ras association (RalGDS/AF-6) domain family member 6
SAV1	1700040G09Rik, Salv, salvador family WW domain containing 1, salvador family WW domain containing protein 1, SAV, WW45, Wwp3, WWP4
ScfTrcpbeta	SCFbeta TRCP, Scf Trcp β, SCFβ TRCP
SCRIB	AI118201, Cr, CRC, CRIB, CRIB1, KIAA0147, mKIAA0147, RGD1565055, Scr, SCRB1, SCRIB1, scribbled planar cell polarity, scribble planar cell polarity, scribble planar cell polarity protein, Vartul
Smad2/3-Smad4	Smad 2/3/4
SMAD4	AW743858, D18Wsu70, D18Wsu70e, DPC, DPC4, JIP, Madh, MADH4, MYHRS, SMAD family member 4, Smaug1
TEAD	TEA domain family
TJP2	C9DUPq21.11, DFNA51, DUP9q21.11, PFIC4, tight junction protein 2, X104, ZO-, ZO-2
TP53BP2	53BP, 53BP2, A, AI746547, ASPP2, p53-Binding, P53BP2, PPP1R13A, transformation related protein 53 binding protein 2, Trp53bp2, tumor protein p53 binding protein 2, tumor protein p53 binding protein, 2, tumour protein p53 binding protein 2, tumour protein p53 binding protein, 2, X98550
WWC1	AA408228, AU017197, BC037006, HBEBP3, HBEBP36, Ki, KIAA0869, KIBRA, MEMRYQTL, PPP1R168, RGD1308329, WW and C2 domain containing 1, WW, C2 and coiled-coil domain containing 1
Wwtr1	2310058J06Rik, 2610021I22Rik, C78399, DKFZp586I1419, TA, TAZ, TRANSCRIPTIONAL COACTIVATOR with PDZ-binding MOTIF, WW domain containing transcription regulator 1
YAP/TAZ	YAP/WWTR1, YAP/WWTRT1
YAP1	AI325207, COB1, Y, YAP, Yap2, YAP65, Yes1 associated transcriptional regulator, yes-associated protein 1, Yk, YKI, yor, Yorkie

Pathway Analysis Using IPA Software; canonical pathway

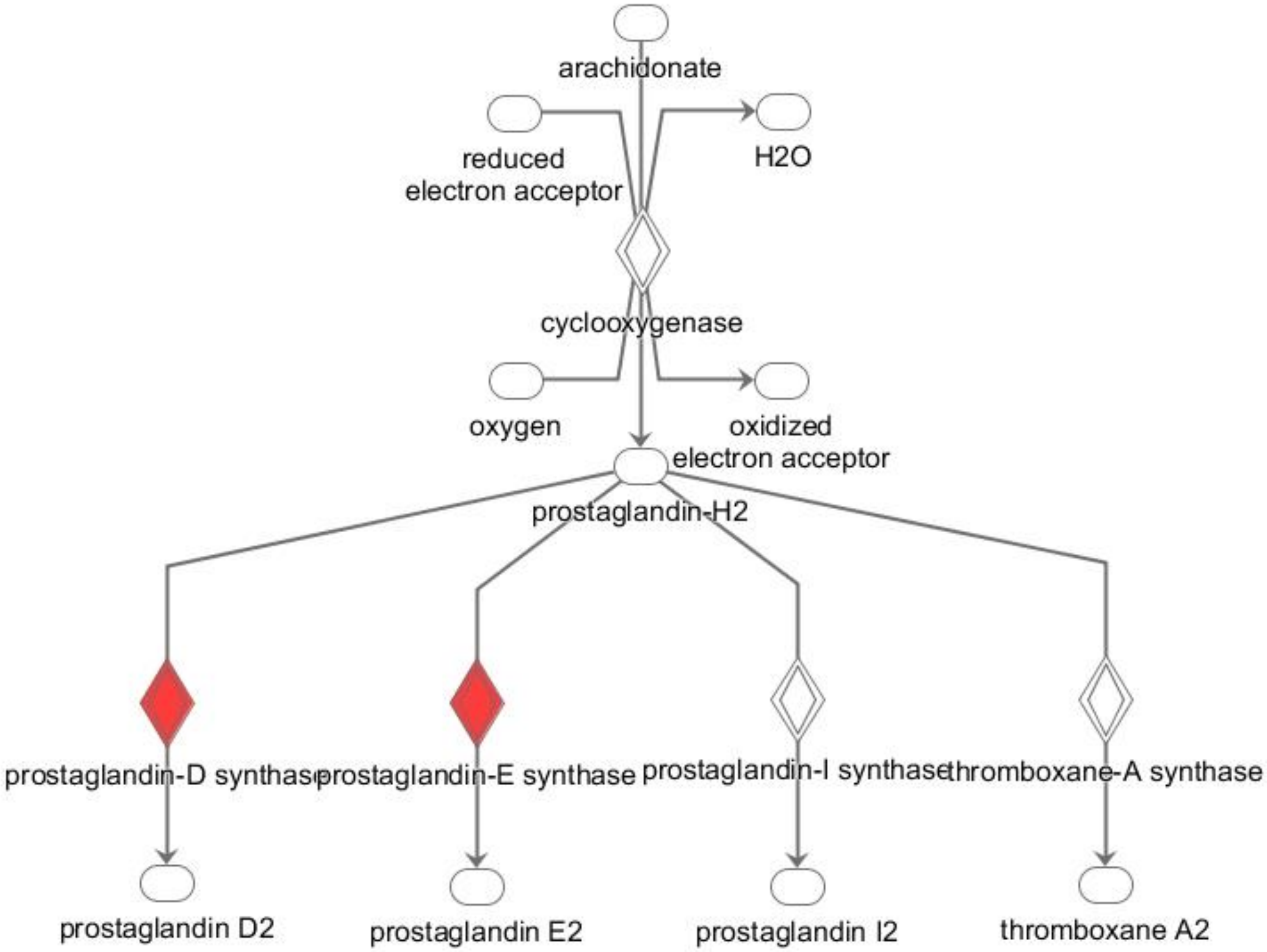

Figure S37. Prostanoid Biosynthesis at 6 h

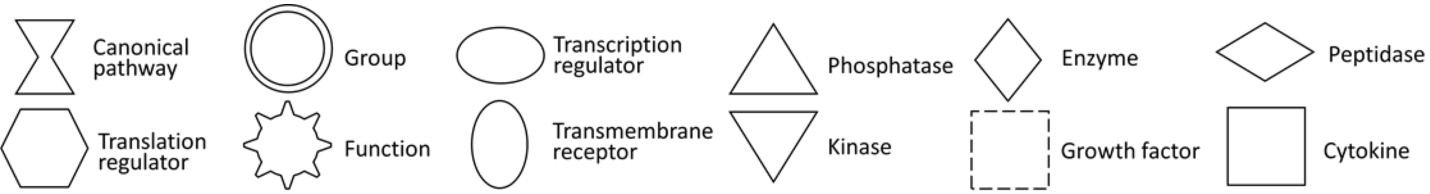

Red: Increased, FDR<0.05 versus solvent control

Green: Decreased, FDR<0.05 versus solvent control

Symbol	Synonym(s)
arachidonate	20:4n-6, 506-32-1, 5,8,11,14-eicosatetraenoic acid, (all-Z)-, 5Z,8Z,11Z,14Z-arachidonic acid, (5Z,8Z,11Z,14Z)-icosa-5,8,11,14-tetraenoic acid, AA, AA-d8, ARA, arachidonate, C20:4(n-6), C20:4w6, C20H32O2, eicosa-5Z,8Z,11Z,14Z-tetraenoic acid, sodium arachidonate
cyclooxygenase	1.14.99.1, (5Z,8Z,11Z,14Z)-icosa-5,8,11,14-tetraenoate,hydrogen-donor:oxygen oxidoreductase, COX, COX1/2, fatty acid cyclooxygenase, PGHS, (PG)H synthase, PG synthetase, Prostaglandin-endoperoxide synthase, prostaglandin endoperoxide synthetase, prostaglandin G/H synthase, prostaglandin G/H synthase and cyclooxygenase, Prostaglandin h synthase, Prostaglandin Peroxidase, prostaglandin synthase, prostaglandin synthetase, PTGS, PTGS1/2
prostaglandin D2	11-dehydroprostaglandin F2-alpha, 11-dehydroprostaglandin F2-α, 41598-07-6, (5Z,13E)-9alpha-hydroxy-11,15-dioxoprosta-5,13-dienoate, (5Z,13E)-9alpha-hydroxy-11,15-dioxoprosta-5,13-dienoic acid, C20H32O5, PGD2, prosta-5,13-dien-1-oic acid, 9,15-dihydroxy-11-oxo-, (5Z,9-alpha,13E,15S)-, prosta-5,13-dien-1-oic acid, 9,15-dihydroxy-11-oxo-, (5Z,9-α,13E,15S)-, (Z)-7-[(1R,2R,5S)-5-hydroxy-2-[(E,3S)-3-hydroxyoct-1-enyl]-3-oxocyclopentyl]hept-5-enoic acid
prostaglandin E2	363-24-6, (5Z, 11a, 13E, 15S)-11, 15-Dihydroxy-9-oxo-prosta-5, 13-dien-1-oic acid, C20H32O5, Cervidil, dinoprostone, PGE2, Prepidil, Propess, Prostarmon E, Prostin E, Prostin E2, Prostin E2 Vaginal Suppository, (Z)-7-[(1R,2R,3R)-3-hydroxy-2-[(E,3S)-3-hydroxyoct-1-enyl]-5-oxocyclopentyl]hept-5-enoic acid
prostaglandin I2	35121-78-9, (5Z)-5-[(3aR,4R,5R,6aS)-5-hydroxy-4-[(E,3S)-3-hydroxyoct-1-enyl]-3,3a,4,5,6,6a-hexahydrocyclopenta[b]furan-2-ylidene]pentanoic acid, 61849-14-7, C20H32O5, epoprostenol sodium, Flolan, PGI2, PGX, prosta-5,13-dien-1-oic acid, 6,9-epoxy-11,15-dihydroxy-, (5Z,9alpha,11alpha,13E,15S)-, prostacyclin, prostaglandin I, prostaglandin I2, sodium PGI2
prostaglandin-D synthase	(5,13)-(15S)-9alpha,11alpha-epidioxy-15-hydroxyprosta-5,13-dienoate D-isomerase, 5.3.99.2, PGH-PGD isomerase, prostaglandin-H2 D-isomerase, prostaglandin-R-prostaglandin D isomerase
prostaglandin-E synthase	5.3.99.3, (5Z,13E)-(15S)-9alpha,11alpha-epidioxy-15-hydroxyprosta-5,13-dienoate E-isomerase, endoperoxide isomerase, PGE2 isomerase, PGE isomerase, PGH-PGE isomerase, prostaglandin endoperoxide E2 isomerase, prostaglandin endoperoxide E isomerase, prostaglandin-H2 E-isomerase, prostaglandin H-E isomerase, prostaglandin R-prostaglandin E isomerase
prostaglandin-H2	42935-17-1, 9,11-epoxymethano-PGH2, C20H32O5, PGH2, PGH2 endoperoxide, prosta-5,13-dien-1-oic acid, 9,11-epidioxy-15-hydroxy-, (5Z,9alpha,11alpha,13E,15S)-, (Z)-7-[(1R,4S,5R,6R)-6-[(E,3S)-3-hydroxyoct-1-enyl]-2,3-dioxabicyclo[2.2.1]heptan-5-yl]hept-5-enoic acid
prostaglandin-I synthase	5.3.99.4, (5Z,13E)-(15S)-9alpha,11alpha-epidioxy-15-hydroxyprosta-5,13-dienoate 6-isomerase, PGI2 synthase, PGI2 synthetase, prostacycline synthetase, prostacyclin synthase, prostagladin I2 synthetase
thromboxane A2	57576-52-0, 5-heptenoic acid, 7-(3-(3-hydroxy-1-octenyl)-2,6-dioxabicyclo(3.1.1)hept-4-yl)-, (1S-(1alpha,3alpha,3R*),4beta(Z),5alpha)-, C20H32O5, thromboxa-5,13-dien-1-oic acid, 9,11-epoxy-15-hydroxy-, (5Z,9alpha,11alpha,13E,15S)-, TxA2, (Z)-7-[(1S,3R,4S,5S)-3-[(E,3S)-3-hydroxyoct-1-enyl]-2,6-dioxabicyclo[3.1.1]heptan-4-yl]hept-5-enoic acid
thromboxane-A synthase	5.3.99.5, (5Z,13E)-(15S)-9alpha,11alpha-epidioxy-15-hydroxyprosta-5,13-dienoate thromboxane-A2-isomerase, thromboxane synthase

Pathway Analysis Using IPA Software; canonical pathway

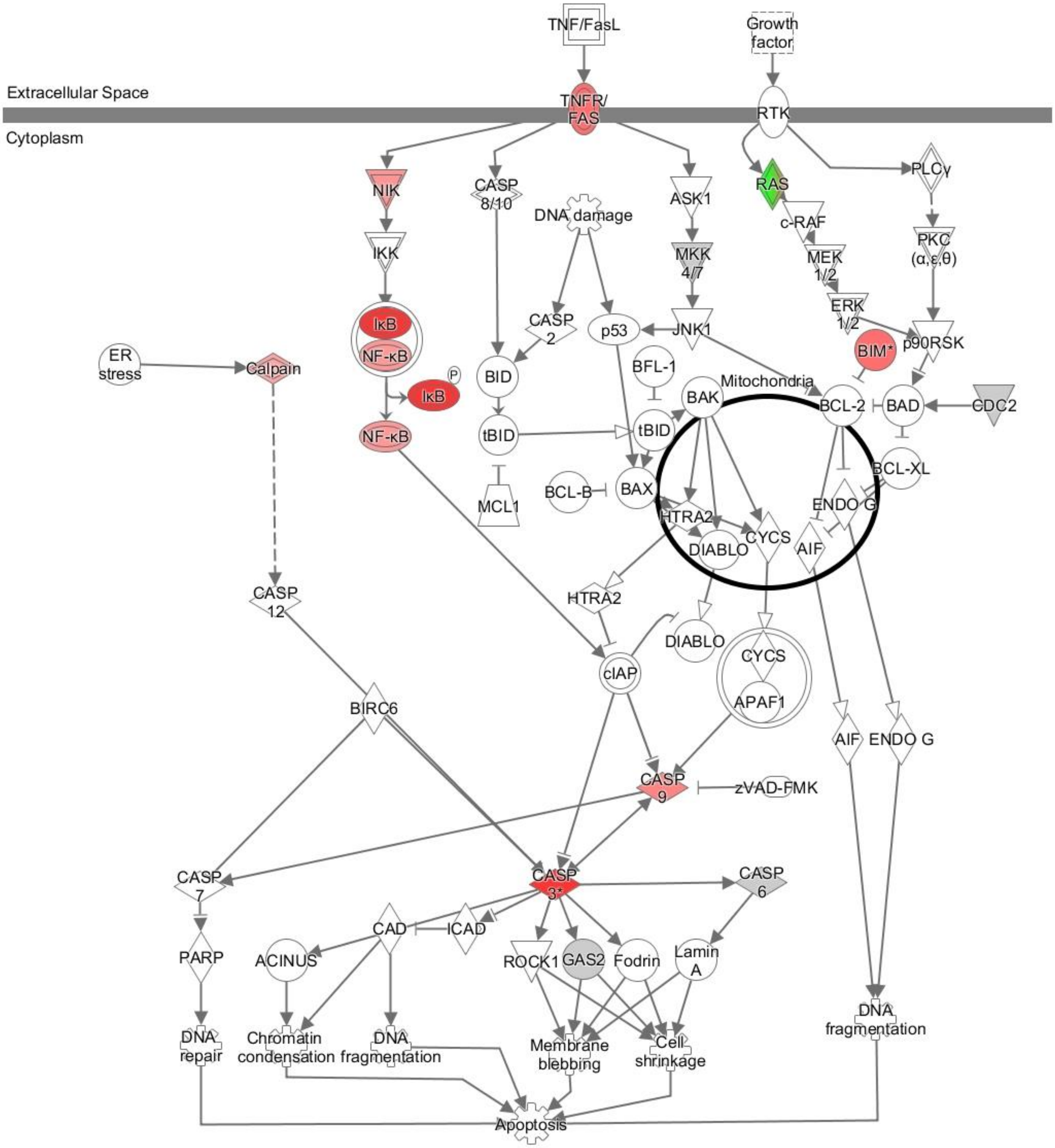

Figure S38. Apoptosis Signaling at 6 h

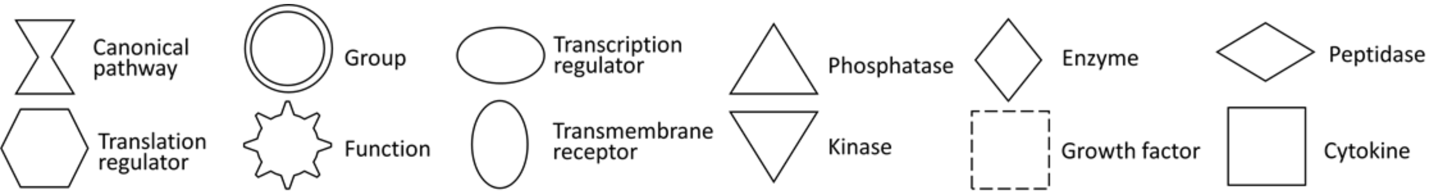

Red: Increased, FDR<0.05 versus solvent control

Green: Decreased, FDR<0.05 versus solvent control

Symbol	Synonym(s)
ACIN1	2610036I19Rik, 2610510L13Rik, Ac, ACINUS, ACINUS-1, acinusL, acinusS, ACN, apoptotic chromatin condensation inducer 1, Apoptotic Chromatin Condensation Inducer In The Nucleus, C79325, fSAP152, mKIAA0670
AIFM1	A, AIF, apoptosis inducing factor mitochondria associated 1, apoptosis inducing factor, mitochondria associated 1, apoptosis-inducing factor, mitochondrion-associated 1, AUNX1, CMT2D, CMTX4, COWCK, COXPD6, DFNX5, Hq, NADMR, NAMSD, Pdcd, PDCD8, SEMDHL
APAF1	6230400I06RIK, Ap, Apaf1l, apoptotic peptidase activating factor 1, CED4, fog, mKIAA0413
Apaf1-Cycs	Apaf1-CytoC, Cyt C-APAF1, CytochromeC-APAF1
BAD	AI325008, Bad v1, Bad v2, BBC2, BCL2-associated agonist of cell death, BCL2L8
BAK1	Ba, BAK, BAK-LIKE, BCL2-antagonist/killer 1, BCL2L7, CDN1, N-B, N-BAK1
BAX	Bcl2-associated X, BCL2 associated X, apoptosis regulator, BCL2-associated X protein, BCL2L4
BCL-XL	bBclxl, Bcl, BCL2L, BCL2-like 1, BCLX, Bcl-X beta, Bclx gamma, BCL-XL/S, Bcl-X β, Bclx γ, PPP1R52
BCL2	AW986256, B cell leukaemia/lymphoma 2, B cell leukemia/lymphoma 2, Bcl-, Bcl2 alpha, BCL2 apoptosis regulator, BCL2, apoptosis regulator, Bcl2 α, C430015F12Rik, D630044D05RIK, D830018M01RIK, LOC100046608, ORF16, PPP1R50
BCL2A1	A, A1-, A1-b, A1-d, ACC-1, ACC-2, BB218357, B cell leukaemia/lymphoma 2 related protein A1a, B cell leukaemia/lymphoma 2 related protein A1b, B cell leukaemia/lymphoma 2 related protein A1d, B cell leukemia/lymphoma 2 related protein A1a, B cell leukemia/lymphoma 2 related protein A1b, B cell leukemia/lymphoma 2 related protein A1d, Bcl2, BCL2A1A, Bcl2a1b, BCL2A1D, BCL2L5, BCL2-related protein A1, Bfl-, Bfl-1, BFL1/A1, GRS, Hbp, HBPA1, U23778, U23781
BCL2L10	AA420380, AU023065, B, BCL2 like 10, BCL-B, Boo, C85687, D, Diva
BCL2L11	1500006F24RIK, BAM, BCL2 like 11, BCL2-like 11 (apoptosis facilitator), Bi, BIM, Bo, BOD, BODL, LOC150819
BID	2700049M22Rik, AI875481, AU022477, BH3 interacting domain death agonist, cBid, FP497
BIRC6	A430032G04Rik, A430040A19Rik, AA501170, APOLLON, Baculoviral IAP repeat-containing 6, Bruc, BRUCE, D630005A10Rik, mKIAA1289, Ubiquitin-conjugating enzyme e2
c-Iap	IAP, NAIP
Calpain	CALCIUM DEPENDENT PROTEASE, M calpain
CASP12	CASP12P1, CASPASE12, caspase 12 (gene/pseudogene)
CASP2	Casp, caspase 2, Ich-, ICH-1, Nedd, NEDD-2, PPP1R57
CASP3	A830040C14Rik, AC-, AC-3, Casp, Caspase-3, CASPASE-3 p20, CC3, CPP, CPP-32, CPP32B, CPP32-beta, CPP32-β, Ice-like cysteine protease, Lice, mld, mldy, SCA-1, Ya, YAMA
CASP6	caspase 6, LOC103689977, mCAS, MCH2
CASP7	AI314680, casp, Caspase-7, CMH-1, ICE-, ICE-IAP3, ICE-LAP3, LICE2, Lice2 cysteine protease, mCASP-7, MCH3
CASP9	AI115399, APAF-3, AW493809, Casp, Casp9 v1, Caspase-9, ICE-, ICE-LAP6, MCH6, PPP1R56
Caspase8/10	Casp8/10, Caspase 8,10
CDK1	CDC2, CDC28A, Cdc2a, CDC2 kinase, cyclin-dependent kinase 1, GROWTH-ASSOCIATED HISTONE H1 KINASE, p34, P34CDC2
CYCS	CYC, CYCSA, CYTC, CYTOC, CYTOCHROME C, cytochrome c, somatic, cytochrome c, somatic-like, ENSMUSG00000058927, HCS, LOC100363502, THC4, X laevis XLCL2
DFFA	A330085O09Rik, DFF1, DFF35, DFF-45, DNA fragmentation factor, alpha subunit, DNA fragmentation factor subunit alpha, DNA fragmentation factor subunit α, DNA fragmentation factor, α subunit, ICA, ICAD, ICAD-S
DFFB	5730477D02Rik, C, CA, CAD, caspase-activated DNase, CPAN, DFF2, DFF-40, Didf, Didff, DNA fragmentation factor, beta subunit, DNA fragmentation factor subunit beta, DNA fragmentation factor subunit β, DNA fragmentation factor, β subunit
DIABLO	0610041G12Rik, 1700006L01RIK, AU040403, DFNA64, diablo IAP-binding mitochondrial protein, diablo, IAP-binding mitochondrial protein, Sm, SMAC
ENDOG	ENDONUCLEASE G
ERK1/2	MAPK p44/42, MAPK p44/p42, p42/44 mapk, P42/p44 erk, P42/p44 mapk, p42/p44 MAP KINASE
GAS2	Gas, growth arrest-specific 2, RGD1563167
HTRA2	AI481710, Htr, HtrA serine peptidase 2, MGCA8, mnd, mnd2, O, OMI, PARK13, Pr, PRSS25
Ikb	I KAPPA B, Ikbeta, Ikβ, Iκ-B
IkB-NfkB	IkappaB-NFkappaB, IκB-NFκB, NFkB-IkB
IKK	I Kappa B Kinase, IKKALPHABETA, IKK Complex, I κ B Kinase
LMNA	CDCD1, CDDC, CMD1A, CMT2B1, Dhe, EMD2, FPL, FPLD, FPLD2, HGPS, IDC, lamin A, LAMIN A/C, LAMININ A/C, LDP1, LFP, LGMD1B, LMN1, LMNC, LMNL1, MADA, Prelamin-A/C, PRO1
MAP2K1/2	MEK1/2, MKK1/2
MAP2K4/7	Jnkk, MEK 4/7, MKK 4/7
MAP3K5	7420452D20Rik, A, APOPTOSIS SIGNAL REGULATED KINASE 1, AS, ASK, ASK1, M3K5, MAPKKK5, MEKK5, mitogen-activated protein kinase kinase kinase 5, RGD1306565
MAPK8	AI849689, C-JUN N-TERMINAL KINASE1, JNK, JNK1, JNK1A2, JNK21B1/2, JNK-46, mitogen-activated protein kinase 8, p46JNK1, p46JNK1 alpha, p46JNK1 α, Prk, PRKM8, SAPK1, SAPK1c, Sapk gamma, SAPK P46, Sapk γ, STRESS-ACTIVATED protein KINASE-LIKE KINASE
MCL1	AW556805, BCL2L3, EAT, Mcl-, MCL1 apoptosis regulator, BCL2 family member, mcl1/EAT, myeloid cell leukaemia sequence 1, myeloid cell leukemia sequence 1, TM
NFkB	NF-KAPPA B, NF-κ B, nuclear factor-κ b, transcription factor nuclear factor κ b
Parp1	5830444G22Rik, A, Adp, Adprp, Adprp1, ADPRT, ADPRT 1, AI893648, ARTD1, C80510, msPARP, pa, pADPRT-1, PARP, PARS, POLY(ADP-RIBOSE) POLYMERASE 1, poly (ADP-ribose) polymerase family, member 1, PPOL, sP, sPARP-1
PKC(α,ε,θ)	PKC (alpha, epsilon, theta), PKC (α,ε,θ)
PLC-gamma	Phospholipase C gamma, Phospholipase C γ, PLCG, PLCγ
RAF1	6430402F14Rik, AA990557, BB129353, CMD1NN, c-R, Cra, CRAF, Craf1, D830050J10Rik, leukaemiA ONCOGENE HOMOLOG1, LEUKEMIA ONCOGENE HOMOLOG1, NS5, Raf-1 proto-oncogene, serine/threonine kinase, v-, v-Raf, v-raf-leukaemia viral oncogene 1, v-raf-leukemia viral oncogene 1
ROCK1	1110055K06Rik, LOC100129157, P160ROCK, p160 ROCK-1, Rho-associated coiled-coil containing protein kinase 1, Roc, ROCK, ROCK-I, ROK, ROK beta, ROK β
RPS6KA1	HU-1, MAPKAPK1, MAPKAPK1A, MAPKAP kinase 1, MAPKAP Kinase 1 Alpha, MAPKAP Kinase 1 α, p90Rsk, p90-RSK 1, p90S6K, RIBOSOMAL protein S6 KINASE A, ribosomal protein S6 kinase A1, ribosomal protein S6 kinase polypeptide 1, Rs, RSK, RSK1, S6K-alpha-1, S6K-α-1
SPTAN1	2610027H02Rik, A2a, Alpha fodrin, (alpha)II-SPECTRIN, AlphaII spectrin, Alpha-spectorin, Alpha spectrin, alpha SPECTRIN 2, DEE5, EIEE5, Fodrin, IPF, NEAS, S, Sp, Spectrin alpha 2, spectrin alpha, non-erythrocytic 1, spectrin, alpha, non-erythrocytic 1, Spectrin α 2, spectrin α, non-erythrocytic 1, spectrin, α, non-erythrocytic 1, SPNA2, SPTA2, α fodrin, (α)II-SPECTRIN, α-spectorin, α spectrin, α SPECTRIN 2
TP53	bbl, BCC7, bfy, bhy, BMFS5, LFS1, p4, p44, p5, P53, P53 cellular tumour antigen, p53 tumor suppressor, transformation related protein 53, TRP53, tumor protein p53, tumour protein p53
zVAD-FMK	benzyloxycarbonyl-VAD-fluoromethyl ketone, N-benzyloxycarbonyl-Val-Ala-Asp-fluoromethyl ketone, ZVAD, z-VAD.FMK

Pathway Analysis Using IPA Software; canonical pathway

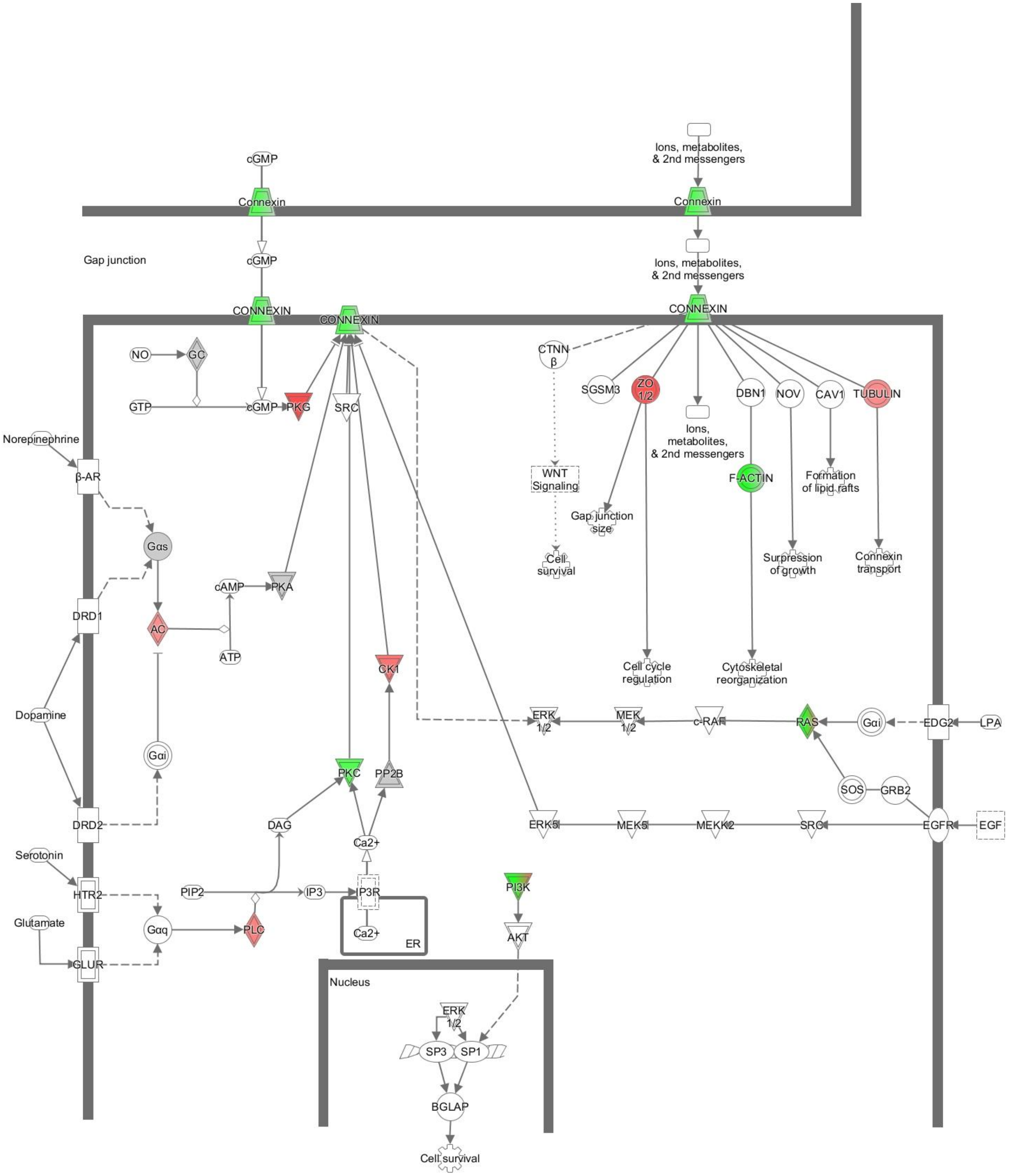

Figure S39. Gap Junction Signaling at 6 h

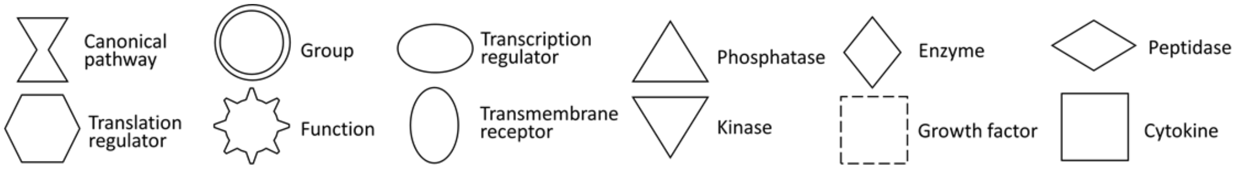

Red: Increased, FDR<0.05 versus solvent control

Green: Decreased, FDR<0.05 versus solvent control

Symbol	Synonym(s)
ADCY	3',5'-cyclic AMP synthetase, 4.6.1.1, AC, Adenylate Cyclase, Adenyl Cyclase, Adenylyl cyclase, ATP diphosphate-lyase (cyclizing), mAC, sAC
ADRB1	Adrb, ADRB1R, ADR-beta1, Adrenergic Receptor Beta 1, adrenergic receptor, beta 1, Adrenergic Receptor β 1, adrenergic receptor, β 1, adrenoceptor beta 1, adrenoceptor β 1, ADR-β1, B1AR, beta-1 adrenergic receptor, beta1-ADRENORECEPTOR, BETA1AR, beta2-AR, beta-AR, FNSS2, RATB1AR, RHR, β 1-adrenergic receptor, β1-AR, β2-AR, β-AR
AKT	AKT1/2/3, B/Akt, PKB, RAC-PK
ATP	[[(2R,3S,4R,5R)-5-(6-aminopurin-9-yl)-3,4-dihydroxyoxolan-2-yl]methoxy-hydroxyphosphoryl] phosphono hydrogen phosphate, 56-65-5, 9-beta-D-arabinofuranosyladenine 5'-triphosphate, 9-β-D-arabinofuranosyladenine 5'-triphosphate, adenosine 5'-(tetrahydrogen triphosphate), adenosine 5'-triphosphate, ATP, ATP4-, C10H16N5O13P3
BGLAP	AI461847, Bgl, Bgla, Bglap1, Bglap2, Bglap3, Bglap-rs1, BGP, BGP2, Bgpr, Bgpra, bone gamma-carboxyglutamate protein, bone gamma-carboxyglutamate protein 2, bone gamma-carboxyglutamate protein 3, Bone Gla-protein, bone γ-carboxyglutamate protein, bone γ-carboxyglutamate protein 2, bone γ-carboxyglutamate protein 3, mOC-, mOC-A, mOC-B, mOC-X, O, OC, OCN, OC-X, OG, OG1, Og2, ORG, oste, Osteocalcin, Osteocalcin2
Ca2+	14127-61-8, Ca+2, calcium, calcium(2+), calcium cation, calcium citrate, calcium ion, calcium, ion (Ca2+), calcium ions, Citracal, tricalcium dicitrate
cAMP	11002-78-1, 33116-15-3, 3',5'-cyclic AMP, 3',5'-monophosphate, adenosine cyclic, 37839-81-9, (4aR,6R,7R,7aS)-6-(6-aminopurin-9-yl)-2-hydroxy-2-oxo-4a,6,7,7a-tetrahydro-4H-furo[3,2-d][1,3,2]dioxaphosphinin-7-ol, 54532-48-8, 55576-98-2, 60-92-4, 66067-13-8, 68407-13-6, adenosine 3',5'-phosphate, adenosine, cyclic 3',5'-(hydrogen phosphate), adenosine cyclic 3,5 monophosphate, adenosine cyclic 3',5'-monophosphate, adenosine cyclic monophosphate, C10H12N5O6P, cAMP, cyclic-3',5'-monophosphate, adenosine, cyclic adenosine monophosphate, cyclic adenylic acid, cyclic AMP, disodium salt, cyclic AMP, monoammonium salt, cyclic AMP, monopotassium salt, cyclic AMP, monosodium salt, cyclic AMP, sodium salt
CAV1	BSCL3, Cav, cave, Cavelolin 1, CAVEOLIN, Caveolin1, caveolin 1, caveolae protein, CGL3, LCCNS, LOC100362870, MSTP085, PPH3, VIP21
CCN3	C130088N23Rik, CCN, cellular communication network factor 3, IBP-9, IGFBP-9, IGFBP-RP3, NOV, NOVH
cGMP	3',5'-cyclic GMP, 7665-99-8, 9-[(4aR,6R,7R,7aS)-2,7-dihydroxy-2-oxo-4a,6,7,7a-tetrahydro-4H-furo[3,2-d][1,3,2]dioxaphosphinin-6-yl]-2-amino-1H-purin-6-one, C10H12N5O7P, cGMP, guanosine-3',5'-cyclic monophosphate, guanosine 3',5'-cyclic phosphate, guanosine cyclic 3',5'-(hydrogen phosphate)
CK1	Casein Kinase I, CKI
CTNNB1	armadillo, Beta-cat, beta CATENIN, Bfc, Cat, CATENIN beta, catenin beta 1, catenin (cadherin associated protein), beta 1, catenin (cadherin associated protein), β 1, CATENIN β, catenin β 1, CATNB, CTNB1, CTNNB, CTNN beta, CTNN β, EVR7, Mesc, MRD19, NEDSDV, β-cat, β-catenin
DAG	DAG, diacylglycerides, diglyceride
DBN1	D0S117E, Drebrin, drebrin 1, Drebrin E, DREBRIN E2
Dopamine	1,2-benzenediol, 4-(2-aminoethyl)-, 1,2-benzenediol, 4-(2-aminoethyl)- (9Cl), 4-(2-aminoethyl)benzene-1,2-diol, 50444-17-2, 51-61-6, 62-31-7, C8H11NO2, DA, dopamine HCl, dopamine hydrochloride, hydroxytyramine, Intropin, Revimine
DRD1	C030036C15Rik, D1, D1a, D1DR, D1R, D1 receptor, D1 receptors, Da-d1 receptor, DADR, Dopamine d1 receptor, dopamine receptor D1, DR1, Drd-, DRD1A, Gpcr, Gpcr15
DRD2	D2, D2a dopamine receptor, D2 DOPAMINE receptor, D2 dopaminergic receptor, D2DR, D2-like receptors, D2R, dopamine D2, Dopamine D2L receptor, dopamine D2 receptor, dopamine receptor D2, Drd-
EGF	AI790464, EGF-1, epidermal growth factor, HOMG4, URG
EGFR	9030024J15RIK, AI552599, C-ERBB, EGFR1, EGF receptor, EGFR vIII, EGF-TK, epidermal growth factor receptor, Erb, ERBB, ERBB1, Err, Errb1, ERRP, HER1, HER1 (EGFR), MENA, NISBD2, PIG61, Wa, wa-2, Wa5
ERK1/2	MAPK p44/42, MAPK p44/p42, p42/44 mapk, P42/p44 erk, P42/p44 mapk, p42/p44 MAP KINASE
F Actin	Filamentous Actin
G proteinalpha I	Galphai, Gi, GI alpha, GI α, GNAI, Gn alpha, Gn α, G protein ai, G protein alpha I SUBUNITS, G protein α I, G protein α I SUBUNITS, Gαi
Glutamate	142-47-2, 19473-49-5, (2S)-2-aminopentanedioic acid, 56-86-0, C5H9NO4, Glu, glutamate, glutamic acid, glutaminol, L-Glu, L-glutamate, L-glutamic acid, monosodium glutamate, MPG, potassium glutamate, potassium L-glutamate, S-glutamate, sodium glutamate
Glutamatereceptor	GluR
GNAQ	1110005L02Rik, 6230401I02Rik, AA408290, AW060788, CMC1, DKFZp686D0521, Dsk, Dsk1, Dsk10, Gal, G-ALPHA-q, GAQ, G protein alpha Q, G protein alpha Q/11, G protein subunit alpha q, G protein subunit α q, G protein α Q, G protein α Q/11, Gq, Gqalpha, GqI, Gq protein alpha subunit, Gq protein α subunit, Gqα, guanine nucleotide binding protein, alpha q polypeptide, guanine nucleotide binding protein, α q polypeptide, G-α-q, Pst receptor, SWS
GNAS	5530400H20RIK, A930027G11RIK, AHO, AHO2, ALEX, C130027O20Rik, C20orf45, G, Ga, G-alpha-8, G alpha S, GANGLIOSIDE EXPRESSION FACTOR 2, Gn, GNAS1, GNAS complex locus, GNAS (guanine nucleotide binding protein, alpha stimulating) complex locus, GNAS (guanine nucleotide binding protein, α stimulating) complex locus, Gnpas, G protein α s, GPSA, Gs-, GSA, Gs-alpha, Gs alpha subunit, Gs GTP-Binding, GSP, GS α, Gs α subunit, Guanine nucleotide binding protein, alpha stimulating, Guanine nucleotide binding protein, α stimulating, G-α-8, G α S, LOC100361691, LOC690994, N, Nes, NESP, Nesp55, NESPL, Oed, OEDSML, P, P1, P2, P3, PHP1A, PHP1B, PITA3, POH, RGD:621483, SCG, SCG6, SgVI, XL, XLalphas
GRB2	AA408164, ASH, Ash-psi, EGFRBP-GRB2, GRAB2, GRBS, growth factor receptor bound protein 2, MST084, MSTP084, NCKAP2
GTP	[[(2R,3S,4R,5R)-5-(2-amino-6-oxo-1H-purin-9-yl)-3,4-dihydroxyoxolan-2-yl]methoxy-hydroxyphosphoryl] phosphono hydrogen phosphate, 86-01-1, C10H16N5O14P3, GTP, guanosine 5'-(tetrahydrogen triphosphate), Mg-GTP
Guanylatecyclase	4.6.1.2, GC, GC activity, GTP diphosphate-lyase (cyclizing), Guanylate cyclase, guanyl cyclase, Guanylyl Cyclase
HTR2	5-HT2, 5-Ht2 Receptor, 5-HTR2
Insp3r	Inositol 1,4,5-triphosphate receptor, Inositol Triphosphate Receptor, INSP3R, Ip3r, IP3 receptor, IP3-Sensitive Calcium Channel
IP3	27121-73-9, inositol trisphosphate, IP3, myo-inositol, tris(dihydrogen phosphate)
LPA	LPA, lysophosphatidic acids, lysophosphatidyl acid
LPAR1	AI326300, clone 4.9, EDG2, ENDOTHELIAL DIFFERENTIATION LYSOPHOSPHATIDIC ACID G-protein-COUPLED receptor 2, Gpcr, Gpcr26, Kdt2, L, LPA1, LPA1 receptor, LPA2, LPA receptor 1, LYSOPHOSPHATIDIC ACID G-protein-COUPLED receptor, lysophosphatidic acid receptor 1, Mrec1.3, rec.1.3, vzg-, VZG1
MAP2K1/2	MEK1/2, MKK1/2
MAP2K5	AI324775, AI428457, HsT17454, MAP kinase kinase 5, MAPKK5, MEK5, mitogen-activated protein kinase kinase 5, MKK5, PRKMK5
MAP3K2	9630061B06RIK, AI585793, LOC100506904, M3K2, MEKK2, MEKK2B, mitogen-activated protein kinase kinase kinase 2
MAPK7	b2b2346C, b2b2346Clo, BMK-1, ERK, ERK4, ERK-5, Erk5-T, ERK7, FRK, LOC100912585, mitogen-activated protein kinase 7, mitogen-activated protein kinase 7-like, PRKM7
NO	10102-43-9, Amidogen, oxo-, EDRF, gaseous nitric oxide, Genosyl, inhaled nitric oxide, INOmax, Mononitrogen monoxide, nitric oxide, nitric oxide gas, nitric oxide gas radical, Nitric oxide trimer, Nitrogen monooxide, nitrogen monoxide, nitrogen oxide (NO), nitrogen protoxide, Nitrosyl radical, NMO, NO
Norepinephrine	108341-18-0, 1,2-benzenediol, 4-(2-amino-1-hydroxyethyl)-, (R)- (9Cl), 1,2-benzenediol, 4-((R)-2-amino-1-hydroxyethyl)-, [3H]-norepinephrine, 4-[(1R)-2-amino-1-hydroxyethyl]benzene-1,2-diol, 51-41-2, benzyl alcohol, alpha-(aminomethyl)-3,4-dihydroxy-, (-)-, benzyl alcohol, α-(aminomethyl)-3,4-dihydroxy-, (-)-, C8H11NO3, D-(-)-noradrenaline, Levophed, Levophed Bitartrate, L-noradrenaline, L-norepinephrine, NE, NE-hydrochloride, noradrenalin, noradrenaline, (-)-noradrenaline, (-)-norepinephrine, norepinephrine bitartrate, (R)-noradrenaline, (R)-norepinephrine, (R)-(-)-norepinephrine
PI3K	1-phosphatidylinositol 3-kinase, 2.7.1.137, ATP:1-phosphatidyl-1D-myo-inositol 3-phosphotransferase, Phosphatidylinositol 3 kinase, phosphatidylinositol 3'-kinase, PI3-kinase, PtdIns 3 Kinase, type III phosphoinositide 3-kinase, type I phosphatidylinositol kinase, Vps34p
PIP2	1,2-diacyl-sn-glycero-3-phospho-(1'-myo-inositol-4',5'-bisphosphate), 1-O-(3-sn-phosphatidyl)-1D-myo-inositol 4,5-bis(dihydrogen phosphate), 1-phosphatidyl-1D-myo-inositol 4,5-bisphosphate, C11H19O19P3R2
PKA	A-Kinase, cAMP-Dependent Protein Kinase, cyclic AMP depended protein kinase, protein KINASE A
PKC	Cnpkc, PKC, Pkc(s), Protein Kinase C
PKG	cgk, protein KINASE G
PLC	3.1.4.3, alpha-toxin, Clostridium oedematiens beta- and g-toxins, Clostridium oedematiens β- and g-toxins, Clostridium welchii alpha-toxin, Clostridium welchii α-toxin, heat-labile haemolysin, heat-labile hemolysin, lecithinase C, lipophosphodiesterase C, lipophosphodiesterase I, phosphatidase C, phosphatidylcholine cholinephosphohydrolase, PHOSPHOINOSITIDE SPECIFIC PHOSPHOLIPASE C, Phospholipase C, Pi-plc, α-toxin
RAF1	6430402F14Rik, AA990557, BB129353, CMD1NN, c-R, Cra, CRAF, Craf1, D830050J10Rik, leukaemiA ONCOGENE HOMOLOG1, LEUKEMIA ONCOGENE HOMOLOG1, NS5, Raf-1 proto-oncogene, serine/threonine kinase, v-, v-Raf, v-raf-leukaemia viral oncogene 1, v-raf-leukemia viral oncogene 1
Serotonin	3-(2-aminoethyl)-1H-indol-5-ol, 3-(2-aminoethyl)indol-5-ol, 50-67-9, 5-HT, C10H12N2O, indol-5-ol, 3-(2-aminoethyl)-, serotonin
SGSM3	1810012I01RIK, AI428557, BB175482, bdif-1, CIP, CIP85, MAP, R75178, RABGAP5, RABGAPLP, RUSC3, Rutbc, RUTBC3, small G protein signaling modulator 3
SP1	1110003E12RIK, AA450830, AI845540, Sp1-1, Sp1 transcription factor, Sp1 (trans spliced isoform), Trans-acting transcription factor 1
SP3	D130027J01RIK, Sp3 transcription factor, SPR2, trans-acting transcription factor 3
SRC	ASV, AW259666, BS27, c-SRC, p60-Src, PP60, pp60c, Pp60/c-Src, pp60c-src, Rous sarcoma oncogene, SRC1, SRC proto-oncogene, non-receptor tyrosine kinase, THC6, TVHUSC
TUBULIN	microtubule, tubulin complex

Pathway Analysis Using IPA Software; canonical pathway

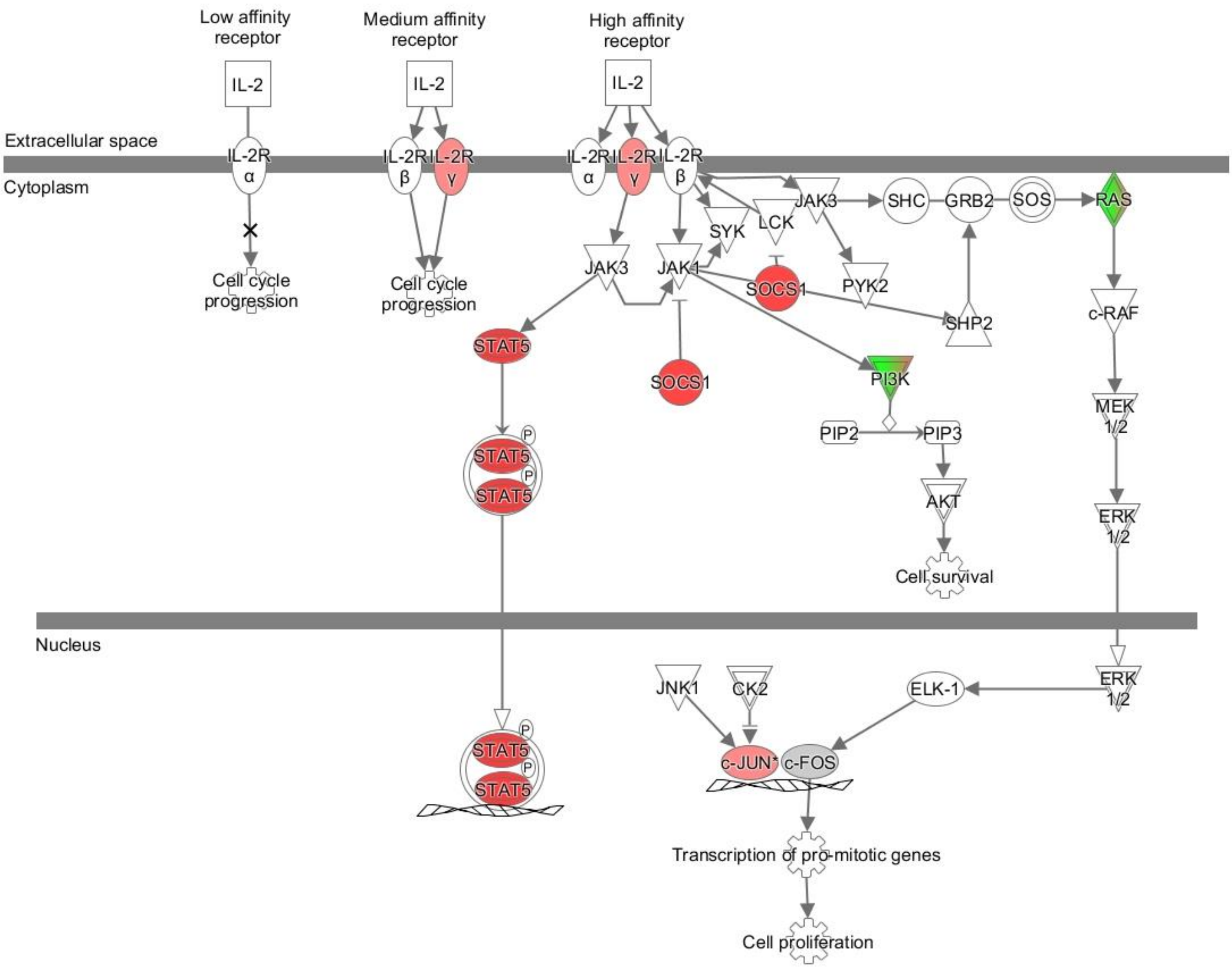

Figure S40. IL-2 Signaling at 6 h

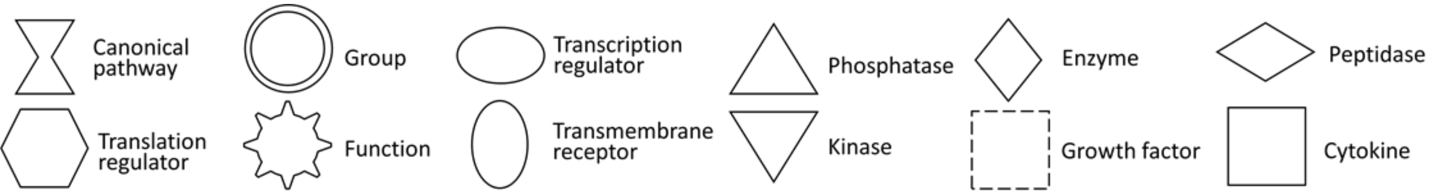

Red: Increased, FDR<0.05 versus solvent control

Green: Decreased, FDR<0.05 versus solvent control

Symbol	Synonym(s)
AKT	AKT1/2/3, B/Akt, PKB, RAC-PK
CK2	Casein Kinase II, CKII
ELK1	ELK, ELK1, member of ETS oncogene family, ETS transcription factor ELK1, p62TCF, RGD:2549, TCF/ELK
ERK1/2	MAPK p44/42, MAPK p44/p42, p42/44 mapk, P42/p44 erk, P42/p44 mapk, p42/p44 MAP KINASE
FOS	AP-1, c-f, C-FOS, D12Rfj, D12Rfj1, FBJ osteosarcoma oncogene, Fos proto-oncogene, AP-1 transcription factor subunit, p55
GRB2	AA408164, ASH, Ash-psi, EGFRBP-GRB2, GRAB2, GRBS, growth factor receptor bound protein 2, MST084, MSTP084, NCKAP2
IL2	IL, interleukin 2, lymphokine, TCGF
IL2RA	CD25, I, IDDM10, IL2R, IL2RAC, Il 2 receptor α subunit, IL-2 Rα, IMD41, interleukin 2 receptor, alpha chain, interleukin 2 receptor subunit alpha, interleukin 2 receptor subunit α, interleukin 2 receptor α, Interleukin 2 receptor α chain, interleukin 2 receptor, α chain, Ly-4, Ly-43, p55, TAC, TAC ANTIGEN, TCGFR
IL2RB	CD122, IL-15R, IL15RB, IL-15R beta, IL-15R β, Il-2/15Rbeta, Il-2R, IL2RBC, Il-2Rbeta, IL-2R β, Il2r β C, IMD63, interleukin 2 receptor, beta chain, interleukin 2 receptor subunit beta, interleukin 2 receptor subunit β, interleukin 2 receptor, β chain, p70, P70-75
IL2RG	Ab2-183, CD132, CIDX, common cytokine receptor γ chain, Common Gamma Chain Receptor, Common γ Chain, Common γ Chain Receptor, Cr gamma, Cr γ, CYTOKINE receptor COMMON gamma CHAIN, CYTOKINE receptor COMMON γ CHAIN, gamm, gamma(c), gc, [g]c, IL-12R gamma, IL-12R γ, IL15RG, IL-2/15R gamma, IL-2/15R γ, Il-2 receptor γ c, IL2R gamma, IL-2R γ, IL4R gamma, IL4R γ, IL7 Rgamma, IMD4, interleukin 2 receptor, gamma chain, interleukin 2 receptor subunit gamma, interleukin 2 receptor subunit γ, interleukin 2 receptor, γ chain, P64, SCIDX, SCIDX1, γ C, γ(c), γ chain
JAK1	AA960307, AIIDE, BAP0, BAP004, C130039L05Rik, JAK1A, JAK1B, Janus kinase 1, JTK3, LOC105378775
JAK3	fae, JAKL, Janus kinase 3, L-JAK, RATJAK3, wil
JUN	Activator protein 1, AP-1, API-1, c-ju, cJUN, Junc, jun proto-oncogene, Jun proto-oncogene, AP-1 transcription factor subunit, LOC100288387, LOC100291417, LOC100293034, p39, v-Jun, V-jun Avian Sarcoma Virus 17 Oncogene Homolog, V-jun Sarcoma Virus 17 Oncogene Homolog
LCK	Hck-3, IMD22, Lck1, LCK proto-oncogene, Src family tyrosine kinase, Lcktkr, LSK, Lskt, lymphocyte protein tyrosine kinase, p56Lck, pp58lck, YT16
MAP2K1/2	MEK1/2, MKK1/2
MAPK8	AI849689, C-JUN N-TERMINAL KINASE1, JNK, JNK1, JNK1A2, JNK21B1/2, JNK-46, mitogen-activated protein kinase 8, p46JNK1, p46JNK1 alpha, p46JNK1 α, Prk, PRKM8, SAPK1, SAPK1c, Sapk gamma, SAPK P46, Sapk γ, STRESS-ACTIVATED protein KINASE-LIKE KINASE
PI3K	1-phosphatidylinositol 3-kinase, 2.7.1.137, ATP:1-phosphatidyl-1D-myo-inositol 3-phosphotransferase, Phosphatidylinositol 3 kinase, phosphatidylinositol 3'-kinase, PI3-kinase, PtdIns 3 Kinase, type III phosphoinositide 3-kinase, type I phosphatidylinositol kinase, Vps34p
PIP2	1,2-diacyl-sn-glycero-3-phospho-(1'-myo-inositol-4',5'-bisphosphate), 1-O-(3-sn-phosphatidyl)-1D-myo-inositol 4,5-bis(dihydrogen phosphate), 1-phosphatidyl-1D-myo-inositol 4,5-bisphosphate, C11H19O19P3R2
PIP3	1-phosphatidyl-1D-myo-inositol 3,4,5-trisphosphate, phosphatidylinositol-3,4,5-trisphosphate, phosphoinositide (3,4,5) P3, PI(3,4,5)P3, PIns(3,4,5)P3, PIP3, PtdIns(3,4,5)P3
PTK2B	CADTK, CAKB, CAKbe, CAK beta, CAK β, cell adhesion kinase β, E430023O05Rik, FADK2, FAK2, PKB, protein tyrosine kinase 2 beta, protein tyrosine kinase 2 β, PTK, PTK2 protein tyrosine kinase 2 beta, PTK2 protein tyrosine kinase 2 β, PYK, PYK2, Raf, RAFTK
PTPN11	2700084A17Rik, AW536184, BPTP3, CFC, JMML, METCDS, MGC14433, Noonan syndrome 1, NS1, protein tyrosine phosphatase non-receptor type 11, protein tyrosine phosphatase, non-receptor type 11, PTP, PTP-1D, PTP2C, S, SAP-2, Sh, SH-P, SHP-2, SH-PTP2, SH-PTP3, Src homology protein 2, SYP
RAF1	6430402F14Rik, AA990557, BB129353, CMD1NN, c-R, Cra, CRAF, Craf1, D830050J10Rik, leukaemiA ONCOGENE HOMOLOG1, LEUKEMIA ONCOGENE HOMOLOG1, NS5, Raf-1 proto-oncogene, serine/threonine kinase, v-, v-Raf, v-raf-leukaemia viral oncogene 1, v-raf-leukemia viral oncogene 1
SHC1	p52SHC, p6, p66, p66s, P66shc, Sh, SHC, Shc (46 kDa isoform), SHCA, SHC adaptor protein 1, Shc p66 isoform, src homology 2 domain-containing transforming protein C1
SOCS1	Cis, CIS1, CISH1, Cish7, JA, JAB, JBP, SOC, Sosc1, SS, SSI-1, STAT INDUCED STAT INHIBITOR-1, suppressor of cytokine signaling 1, TIP-3
STAT5	Mgf, STAT5
SYK	p72-Syk, Ptk72, spleen associated tyrosine kinase, spleen tyrosine kinase

Pathway Analysis Using IPA Software; canonical pathway

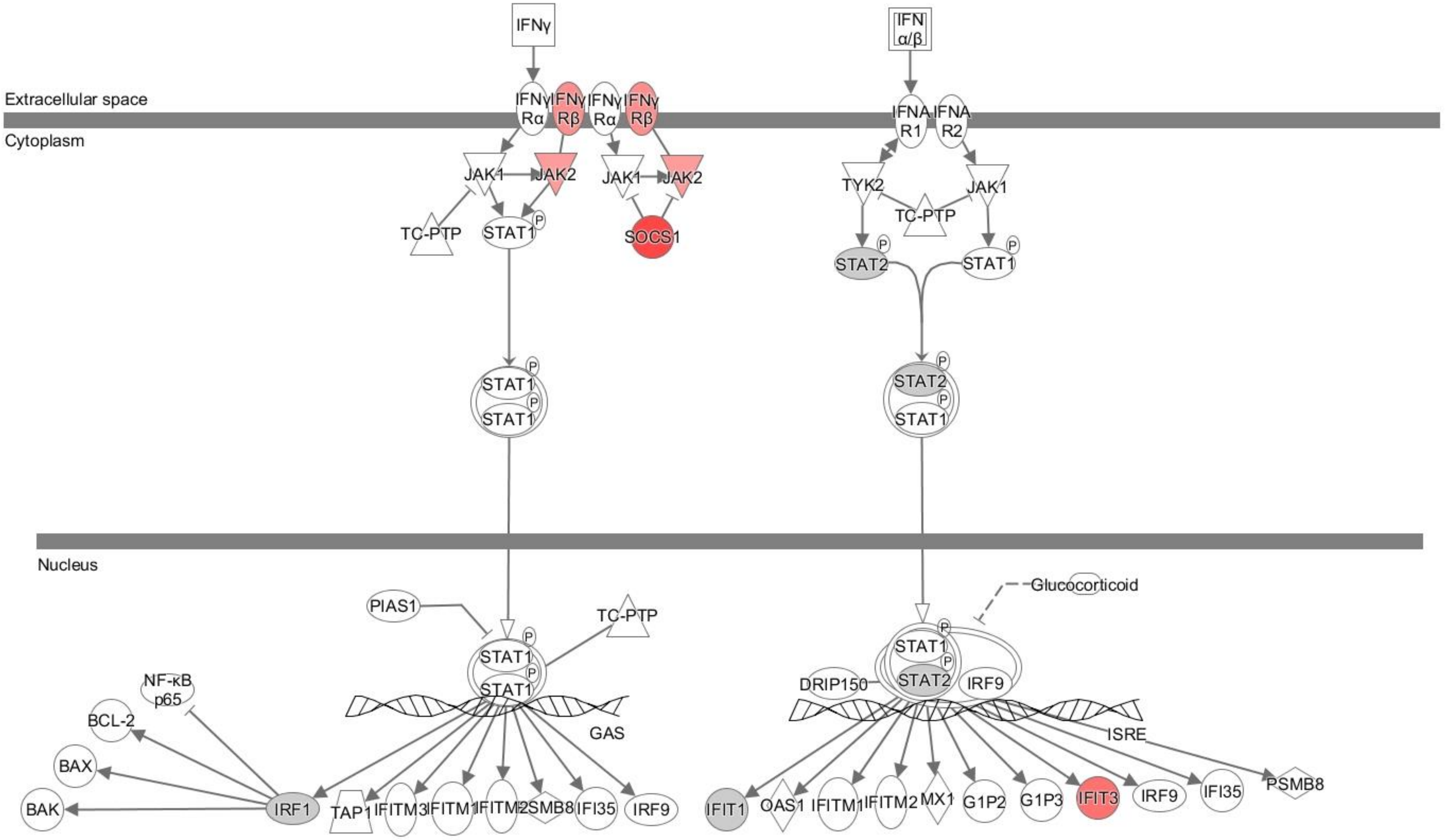

Figure S41. Interferon Signaling at 6 h

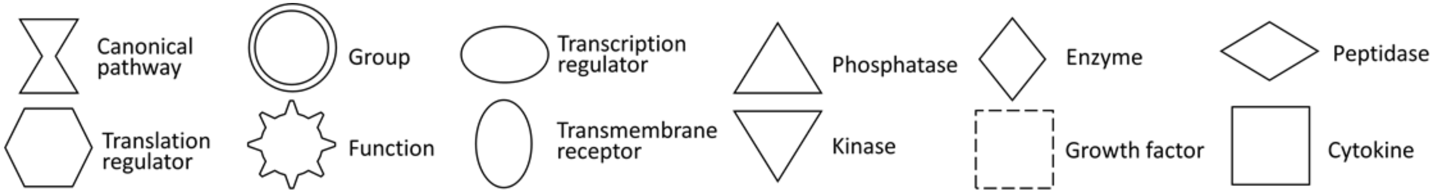

Red: Increased, FDR<0.05 versus control

Green: Decreased, FDR<0.05 versus control

Symbol	Synonym(s)
BAK1	Ba, BAK, BAK-LIKE, BCL2-antagonist/killer 1, BCL2L7, CDN1, N-B, N-BAK1
BAX	Bcl2-associated X, BCL2 associated X, apoptosis regulator, BCL2-associated X protein, BCL2L4
BCL2	AW986256, B cell leukaemia/lymphoma 2, B cell leukemia/lymphoma 2, Bcl-, Bcl2 alpha, BCL2 apoptosis regulator, BCL2, apoptosis regulator, Bcl2 α, C430015F12Rik, D630044D05RIK, D830018M01RIK, LOC100046608, ORF16, PPP1R50
Glucocorticoid	glucocorticoid hormone, glucocorticosteroid, glucocorticosteroids
IFI35	2010008K16RIK, AW986054, IFP, IFP35, interferon-induced protein 35, IPF35
IFI6	6-16, FAM14C, G1P3, IFI-6-16, IFI6-26, IFN6-16, IFN alpha REGULATED, IFN α REGULATED, INTERFERON alpha INDUCIBLE, interferon alpha inducible protein 6, INTERFERON INDUCIBLE PEPTIDE (6-16), INTERFERON α INDUCIBLE, interferon α inducible protein 6, ISG6-16
IFIT1	2010002M12Rik, AW412491, C56, G10P1, Gm14446, Ifi, IFI-56, IFI-56K, Ifit1b, Ifit1bl, Ifit1bl1, Ifit1bl2, Ifit1c, Ifit1lb, Ifit5, IFN56K, IFNAI1, IFN STIMULATED GENE 56, IFN/TETRA1, interferon induced protein with tetratricopeptide repeats 1, interferon-induced protein with tetratricopeptide repeats 1B-like, interferon induced protein with tetratricopeptide repeats 1B like 2, interferon induced protein with tetratricpeptide repeats 1B like 1, INTERFERON INDUCIBLE, INTERFERON INDUCIBLE 56KD, ISG, ISG56, P56, RNM561
IFIT3	CIG-41, CIG-49, GARG-49, I830012O16Rik, Ifi, Ifi49, IFI60, IFI-60K, Ifit3b, IFIT4, interferon-induced protein with tetratricopeptide repeats 3, interferon-induced protein with tetratricopeptide repeats 3B, IRG2, ISG-561, ISG60, P49, P60, RIG-G
IFITM1	1110036C17Rik, 9-27, CD225, DSPA2a, fra, HUM927A, IFI17, IFI27SEP, IFIM1, IFM1 9-27, IFN9-27, interferon induced transmembrane protein 1, LEU13, Mi, Mil-2
IFITM2	1-8D, DSPA2c, fra, fragilis3, Ifi 16, IFI1-8U, Ifit, IFITM3L, IFITML, IFN1-8D, interferon induced transmembrane protein 2, Interferon induced transmembrane protein 3-like, mil, mil-3
IFITM3	1110004C05Rik, 1-8U, Cd225, Cdw217, DSPA2b, Fg, FGLS, fr, IFN1-8U, Interferon beta induced, Interferon induced, interferon induced transmembrane protein 3, Interferon inducible, Interferon β induced, IP, IP15, mil, mil-1, rat8
Ifnalpha/beta	IFNalpha/beta late, IFN-I, Ifn type i, IFN-α β, IFNα/β, IFNα/β late, Interferon alpha/beta, Interferon α/β
IFNAR1	alpha CHAIN of type I IFNR, AVP, I, If, Ifar, IFN-alpha-beta-R, IFNalpha/betaR, Ifn-alpha/beta-receptor, IFN alpha/beta receptor 1, IFN-alpha-REC, IFNAR, IFNBR, IFN receptor CHAIN 1, IFN receptor type 1, IFN type 1 receptor, IFN-α-REC, IFN α/βR, IFN-α-β-R, Ifn-α/β-receptor, IFN α/β receptor 1, IFRC, Infar, interferon (alpha and beta) receptor 1, interferon alpha and beta receptor subunit 1, Interferon Receptor, interferon (α and β) receptor 1, interferon α and β receptor subunit 1, LOC284829, type 1 interferon receptor, Type I IFNR, Type I infr, α CHAIN of type I IFNR, β r1
IFNAR2	AI747302, beta subunit of type I IFNR, Ifn, IFNABR, IFNalphabetaR, IFN-alpha-REC, IFNARB, IFN-R, IFN-α-REC, IFNαβR, IMD45, interferon (alpha and beta) receptor 2, interferon alpha and beta receptor subunit 2, interferon (α and β) receptor 2, interferon α and β receptor subunit 2, Type II IFNR, β subunit of type I IFNR
IFNG	If, If2f, IFG, IFI, IFN-2, IFNG2, IFN gamma, IFN-II, IFN type II, IFN-γ, IMD69, INF-γ, Interferon gamma, Interferon γ, type II INTERFERON, γ-ifn, γ interferon
IFNGR1	CD119, If, Ifgr, IFN-g, IFN-gammaR, IFN gamma R alpha, IFN-gamma receptor, IFNGR, Ifngra, IFNR, IFNγR, IFN-γR1, IFN-γ receptor, IFNγ Rα, IMD27A, IMD27B, interferon gamma receptor 1, INTERFERON gamma receptor alpha CHAIN, interferon γ receptor, interferon γ receptor 1, INTERFERON γ receptor α CHAIN, MAF receptor, Nk, Nktar
IFNGR2	AF-1, Ifg, IFGR2, Ifgt, IFNgamma Rbeta, IFNGRB, IFNGT1, IFNγ R2, IFNγ Rβ, IMD28, interferon gamma receptor 2, interferon γ receptor 2
IRF1	AU020929, IFN REGULATORY FACTOR 1, interferon regulatory factor 1, Irf, ISGF2, LSIR, MAR
IRF9	interferon regulatory factor 9, INTERFERON-STIMULATING TRANSCRIPTION FACTOR 3 gamma, INTERFERON-STIMULATING TRANSCRIPTION FACTOR 3 γ, Irf, Isgf, ISGF3, ISGF3G, isgf3 γ, p4, p48
ISG15	G1p, G1P2, Gip2, hUCRP, HUMIFN15K, IFI15, IFI-15K, IFN15/17, IFN-alpha-INDUCIBLE, IFN-INDUCIBLE protein 15 KD, IFN-α-INDUCIBLE, IGI15, IMD38, INTERFERON-INDUCED 17-KDA, INTERFERON-STIMULATED protein 15 KDa, IP17, Irfp, Isg15/17, ISG15 ubiquitin-like modifier, LOC100044225, UCRP
JAK1	AA960307, AIIDE, BAP0, BAP004, C130039L05Rik, JAK1A, JAK1B, Janus kinase 1, JTK3, LOC105378775
JAK2	AI504024, C81284, Fd17, Janus kinase 2, JTK10
MED14	9930001L01RIK, AU041628, Cr, CRSP150, CRSP2, CSRP, CXorf4, DRIP150, ENSMUSG00000073278, EXLM1, Gm641, mediator complex subunit 14, ORF1, RGD1560170, RGR1, Trap, TRAP170
MX1	AI893580, IFI78, IFI-78K, lncMX1-215, MX, Mx2, Mx2 + Mx3, Mx3, MxA, MX dynamin like GTPase 1, MX dynamin like GTPase 2, MYX1
OAS1	2-5A SYNTHETASE, 2',5'-oligoadenylate synthetase, 2'5' OLIGOADENYLATE SYNTHETASE1, 2'-5' oligoadenylate synthetase 1A, 2'-5' oligoadenylate synthetase 1G, 2'-5' OLIGO A SYNTHETASE E, AI449562, E18/E16, IFI-4, L, L2, L3, Mmu-, Mmu-L, Mmu-L2, O, OAS1A, Oas1b, Oas1c, Oas1g, OAS p40/46, Oi, OIAS, Oias-1, OIASI
PIAS1	2900068C24Rik, Ddxbp, DDXBP1, GB, GBP, GU/RH-II, protein inhibitor of activated STAT 1, protein inhibitor of activated STAT, 1, ZMIZ3
PSMB8	20s proteosome subunit, ALDD, Beta 5i, beta 5I IMMUNOPROTEASOME subunit, D6S216, D6S216E, JMP, large multifunctional protease-7, Lm, Lmp, LMP7, Lmp8, NKJO, PRAAS1, proteasome 20S subunit beta 8, proteasome 20S subunit β 8, proteasome (prosome, macropain) subunit, beta type 8 (large multifunctional peptidase 7), proteasome (prosome, macropain) subunit, β type 8 (large multifunctional peptidase 7), proteasome subunit Y, PSMB5i, Rc1, RING10, β 5i, β 5I IMMUNOPROTEASOME subunit
PTPN2	AI325124, protein tyrosine phosphatase non-receptor type 2, protein tyrosine phosphatase, non-receptor type 2, Pt, PTN2, PTPase, Ptps, PTPT, Tc45, TCELLPTP, T CELL PTPASE, TC-P, TC-PTP
RELA	CMCU, NF-kappa B, NF-kappa B (p65), NF KAPPA B subunit P65, NFkB, NFKB3, NF-κ B (p65), NFkB/p65, NF-κ B, NF-κ B (p65), NF-κB p65, NF κ B subunit P65, nos2, p6, p65, p65 NF-kappa B, p65 NFkB, p65 NF-κ B, p65/Rela, RELA proto-oncogene, NF-kB subunit, v-rel reticuloendotheliosis viral oncogene homolog A (avian)
SOCS1	Cis, CIS1, CISH1, Cish7, JA, JAB, JBP, SOC, Sosc1, SS, SSI-1, STAT INDUCED STAT INHIBITOR-1, suppressor of cytokine signaling 1, TIP-3
STAT1	2010005J02Rik, AA408197, CANDF7, DD6G4-4, IMD31A, IMD31B, IMD31C, ISGF-3, p91, signal transducer and activator of transcription 1, STAT1 alpha, Stat1 beta, Stat1 p91, STAT1 α, Stat1 β, STAT91, TRANSCRIPTION FACTOR SIGNAL TRANSDUCER and ACTIVATOR
STAT2	1600010G07Rik, AW496480, IMD44, ISGF-3, P113, PTORCH3, signal transducer and activator of transcription 2, STAT113
TAP1	ABC17, Abcb, ABCB2, APT1, Cim, D6S114E, Ham-, Ham-1, MTP, MTP1, PSF, PSF-1, RI, RING4, T, TAP, TAP1*0102N, TAP1N, Tap2, TRANSPORTER 1 ATP-binding CASSETTE SUBFAMILY B, transporter 1, ATP-binding cassette, sub-family B (MDR/TAP), transporter 1, ATP binding cassette subfamily B member, TRANSPORTER 1 (MDR/TAP), Y3
TYK2	IMD35, JTK1, tyrosine kinase 2

Pathway Analysis Using IPA Software; canonical pathway

1 h

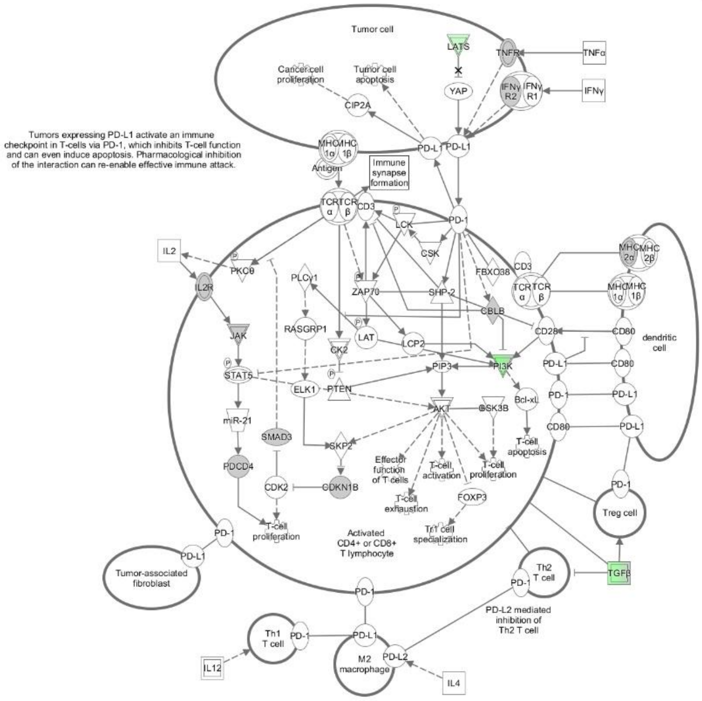

6 h

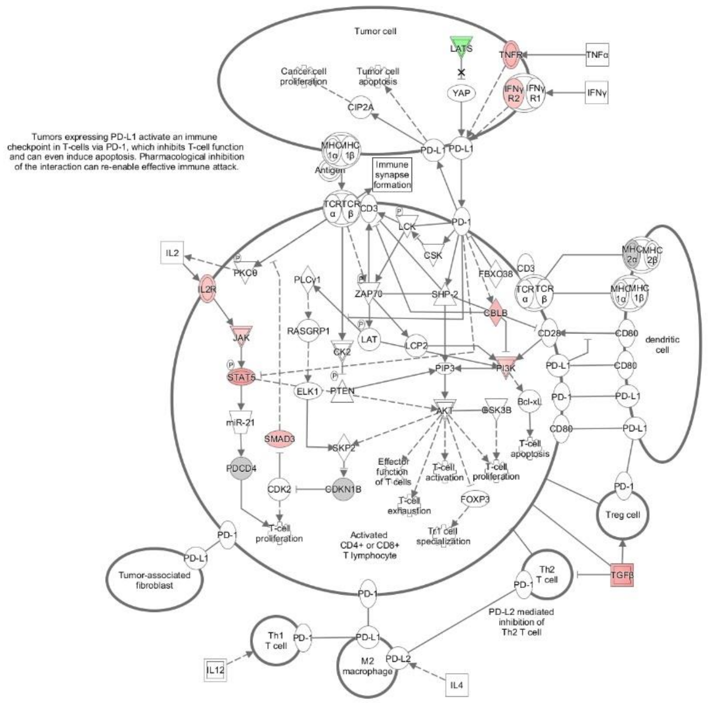

24 h

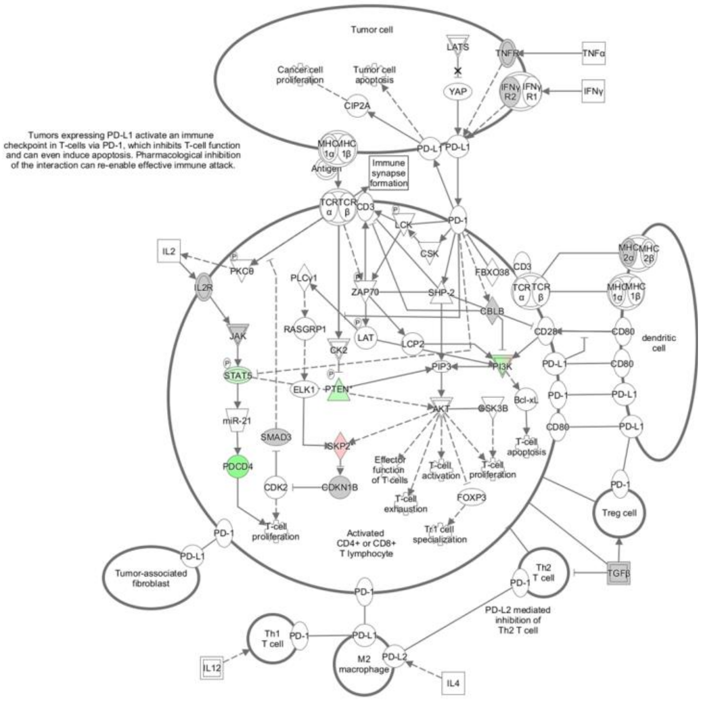

8 days

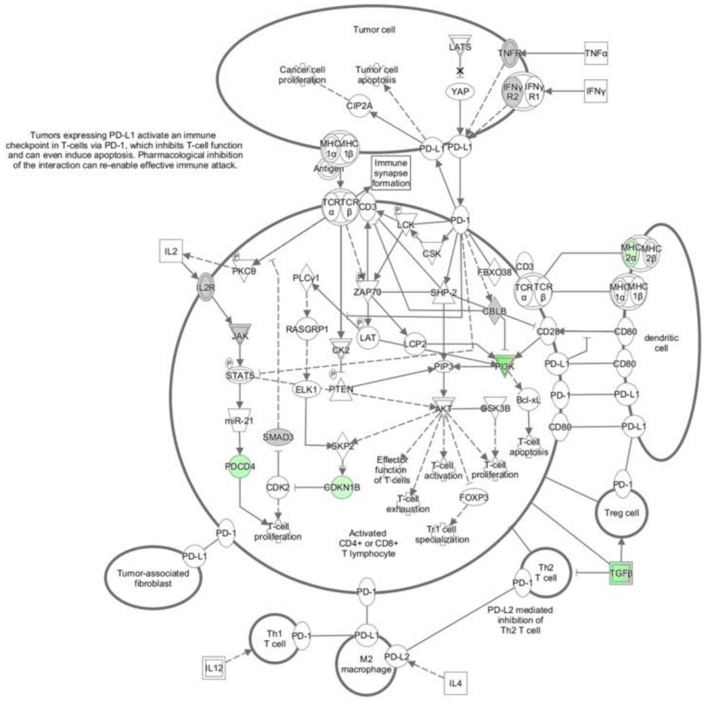

Figure S42. PD-1, PD-L1 cancer immunotherapy pathway

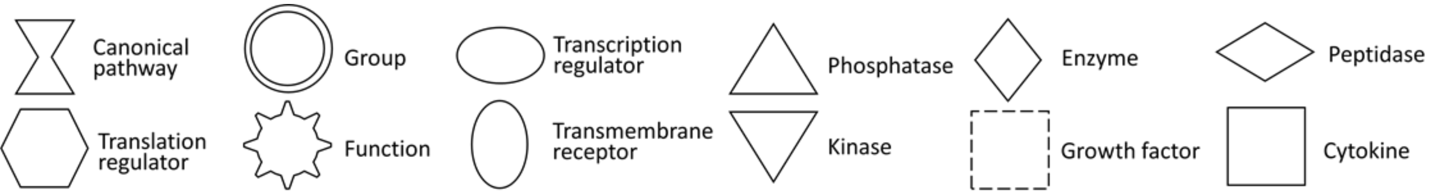

Red: Increased, FDR<0.05 versus solvent control

Green: Decreased, FDR<0.05 versus solvent control

Symbol	Synonym(s)
AKT	AKT1/2/3, B/Akt, PKB, RAC-PK
Bcl-xL	bBclxl, Bcl-X beta, Bcl-X β, BCL-XL/S, BCL2-like 1, BCL2L, BCLX, Bclx gamma, Bclx γ, PPP1R52
CBLB	AI429560, AI851073, Casitas B-lineage lymphoma b, Cbl proto-oncogene B, Nbla00127, RNF56
CD28	CD28 ANTIGEN, CD28l, CD28 molecule, CD28RNA, LOC100048845, Tp44
CD3	4930549J05RIK, A430104F18RIK, AW552088, CD16Z, CD247 antigen, CD247 molecule, Cd3, CD3H, CD3 NU, CD3Q, CD3Z, CD3-ZETA, CD3-ζ, IMD25, T3Z, Tcrk, TCRZ, TCRzeta, Tcr ζ
CD80	B7, B7-1, B7.1, BB1, Cd28l, CD28LG, CD28LG1, CD80 antigen, CD80 molecule, LAB7, LOC100360171, Ly-53, MIC17, TSA1
CDK2	A630093N05Rik, CDC2-RELATED KINASE, CDKN2, Cyclin A associated kinase, CYCLIN E ASSOCIATED KINASE, cyclin-dependent kinase 2, p33(CDK2)
CDKN1B	AA408329, AI843786, Cdki1b, CDKN4, cyclin-dependent kinase inhibitor 1B, CYCLIN-DEPENDENT KINASE INHIBITOR P27, KIP1, MEN4, MEN1B, p27, P27KIP1, P27kip, P28-ICK
CIP2A	AA408511, AU018569, C330027C09, C330027C09Rik, cell proliferation regulating inhibitor of protein phosphatase 2A, cellular inhibitor of PP2A, KIAA1524, p90, RGD1310335
CK2	Casein Kinase II, CKII
CSK	AW212630, c-src tyrosine kinase, C-terminal Src kinase, p50CSK
ELK1	ELK, ELK1, member of ETS oncogene family, ETS transcription factor ELK1, p62TCF, RGD:2549, TCF/ELK
FBXO38	6030410I24RIK, AU044865, AW214031, F-box protein 38, Fbx38, FLJ13962, HMN2D, MOKA, SP329
FOXP3	AIID, DIETER, Forkhead box P3, FOXP3A, IPEX, JM2, PIDX, RGD1562112, scurfin, sf, XPID
GSK3B	7330414F15Rik, 8430431H08Rik, C86142, glycogen synthase kinase 3 beta, glycogen synthase kinase 3 β, GSK-3, GSK-3beta, GSK-3β, GSKbeta, GSKβ, Tpk1
IFNγ	IFG, IFI, IFN-2, IFNG2, IFN gamma, IFN-II, IFN type II, IFN-γ, INF-γ, Interferon gamma, Interferon γ, type II INTERFERON, γ-ifn, γ interferon
IFNγR1	CD119, Ifgr, IFN-gammaR, IFN gamma R alpha, IFN-gamma receptor, IFNGR, Ifngra, IFNR, IFNγR, IFNγ R1, IFN-γ receptor, IFNγ Rα, IMD27A, IMD27B, interferon gamma receptor 1, INTERFERON gamma receptor alpha CHAIN, interferon γ receptor, interferon γ receptor 1, INTERFERON γ receptor α CHAIN, MAF receptor, Nktar
IFNγR2	AF-1, IFGR2, Ifgt, IFNgamma Rbeta, IFNGRB, IFNGT1, IFNγ R2, IFNγ Rβ, IMD28, interferon gamma receptor 2, interferon γ receptor 2
IFNγR	IFN-gammaR, IFN-gamma receptor, Ifn type ii receptor, IFNγR, IFN-γ receptor, Receptors Activated by Interferon-gamma, Receptors Activated by Interferon-γ, type II IFN receptor
IL12	interleukin-12
IL2	interleukin 2, lymphokine, TCGF
IL2R	Il2 Receptor
IL4	BCGF, BCGF-1, BSF-1, Il4e12, interleukin 4
JAK	JAK kinase
LAT	IMD52, LAT1, linker for activation of T cells, p36-38, pp36
LATS	LATS1/2, LATS 1 and 2
LCK	Hck-3, IMD22, Lck1, LCK proto-oncogene, Src family tyrosine kinase, Lcktkr, LSK, Lskt, lymphocyte protein tyrosine kinase, p56Lck, pp58lck, YT16
LCP2	AI323664, BB161688, Lymphocyte cytosolic protein 2, m1Khoe, SLP-76, twm
MHC1β	AS, B-4901, Bw-50, Bw-52, Bw-54, Bw-55, Bw-56, HLA-B27, HLA B7, HLAC, LOC730410, major histocompatibility complex, class I, B, MHC 1-beta, MHC 1β
MHC2α	Mhc2 α, MHC II-alpha, MHC II alpha CHAIN, MHC II-α, MHC II α CHAIN
MHC2β	MHC 2beta, MHC 2β, MHC II-beta, MHC II beta CHAIN, MHC II β CHAIN
MHC 1α	MHC 1-alpha, MHC 1-α, MHC CLASS I alpha, MHC CLASS I α, MHC I-alpha
MHC Class I	HLA Class I, MHC-1
MHC Class II	HLA Class II, MHC II
miR-21	HSA-MIR-104, HSA-MIR-21, MI0000110, microRNA 21, microRNA 21a, MIR-021, Mir21a, MIRN21, miRNA21, mmu-mir-21, mmu-mir-21a, pre-mir-21, rno-mir-21
PD-1	B7H1, CD279, hPD-1, hPD-l, hSLE1, LOC100911478, Ly101, PD, PD-1, Pd-1 receptor, Pdc1, PhLP, programmed cell death 1, SLEB2
PD-L1	A530045L16RIK, B7-H, B7H1, CD274 antigen, CD274 molecule, hPD-L1, PD-L1, Pd1l, PDCD1L1, PDCD1LG1, RGD1566211
PD-L2	B7-DC, bA574F11.2, Btdc, CD273, F730015O22Rik, PDCD1L2, PD-L2, programmed cell death 1 ligand 2
PDCD4	197/15A, D19Ucla1, Dug, H731, Ma3, programmed cell death 4, Tis, Topoisomerase-inhibitor suppressed
PI3K	1-phosphatidylinositol 3-kinase, 2.7.1.137, ATP:1-phosphatidyl-1D-myo-inositol 3-phosphotransferase, Phosphatidylinositol 3 kinase, phosphatidylinositol 3'-kinase, PI3-kinase, PtdIns 3 Kinase, type III phosphoinositide 3-kinase, type I phosphatidylinositol kinase, Vps34p
PIP3	1-phosphatidyl-1D-myo-inositol 3,4,5-trisphosphate, phosphatidylinositol-3,4,5-trisphosphate, phosphoinositide (3,4,5) P3, PI(3,4,5)P3, PIns(3,4,5)P3, PIP3, PtdIns(3,4,5)P3
PKCθ	A130035A12Rik, AW494342, nPKC-theta, nPKC-θ, PKC-0, Pkcq, PKC-theta, PKC-θ, PRKCT, protein kinase C theta, protein kinase C, theta, protein kinase C θ, protein kinase C, θ
PLCγ1	AI894140, CDED, NCKAP3, phospholipase C gamma 1, phospholipase C, gamma 1, phospholipase C-γ-1, phospholipase C, γ 1, PIPLC gamma, PI-PLCgamma1, PIPLC γ, PI-PLCγ 1, PLC1, PLC148, PLC Gamma, PLCgamma1, PLC-II, PLC γ, PLCγ1, PPLCA
PTEN	10q23del, 2310035O07RIK, A130070J02Rik, AI463227, B430203M17RIK, BZS, CWS1, DEC, GLM2, MHAM, Mmac, MMAC1, MUTATED IN MULTIPLE ADVANCED CANCERS, mutated in multiple advanced cancers 1, phosphatase and tensin homolog, PTEN1, PTENbeta, TEP1
RASGRP1	CALDAG-GEFI, CALDAG-GEFII, IMD64, RASGRP, RAS guanyl releasing protein 1
SHP-2	2700084A17Rik, AW536184, BPTP3, CFC, JMML, METCDS, MGC14433, Noonan syndrome 1, NS1, protein tyrosine phosphatase non-receptor type 11, protein tyrosine phosphatase, non-receptor type 11, PTP-1D, PTP2C, SAP-2, SHP-2, SH-PTP2, SH-PTP3, Src homology protein 2, SYP
SKP2	4930500A04Rik, AC139209.1, cyclin A-associated kinase, FBL1, F-box protein Skp2, FBXL1, FLB1, FWD1, p45, p45Skp2, RGD1562456, S-PHASE KINASE-ASSOCIATED protein 2, S-phase kinase-associated protein 2 (p45)
SMAD3	AU022421, DKFZP586N0721, hMAD-3, HSPC193, HsT17436, JV15-2, LDS1C, LDS3, MAD3, MADH3, SMAD family member 3
STAT5	Mgf, STAT5
TCRα	IMD7, PT alpha, PT α, T cell receptor alpha chain, T cell receptor alpha locus, T cell receptor α chain, T cell receptor α locus, TCRA, Tcralpha, TCR α, TRA@
TCRβ	RATTCB, RATTCBC1, TCB, TCBC1, T-cell receptor beta chain, T cell receptor beta locus, T-cell receptor β chain, T cell receptor β locus, TCRB, TCRbeta, TCR β, Tib, TRB@
TGFβ	TGF β, TGF-beta 1, 2, and 3, TGF-β 1, 2, and 3, Tgfb, transforming growth factor-β
TNFR	member of the tumour necrosis factor receptor family, TNFR, TNF R1, Tnf receptor superfamily, tumour necrosis factor receptor
TNFα	AT-TNF, DIF, RATTNF, TMTNF, TNF-a, TNF-alpha, Tnfsf1a, TNFSF2, TNF-α, TNLG1F, tumor necrosis factor, Tumor Necrosis Factor α, tumor necrosis factor, α, tumour necrosis factor, tumour Necrosis Factor Alpha, tumour necrosis factor, alpha, tumour Necrosis Factor α, tumour necrosis factor, α
Tra@-Trb@	alpha/beta TCR, TCR, TCRalpha-beta, TCRalpha-β, α/β TCR
YAP	AI325207, COB1, YAP, Yap2, YAP65, Yes1 associated transcriptional regulator, yes-associated protein 1, YKI, Yorkie
ZAP70	ADMIO2, IMD48, mrtle, mur, SRK, STCD, STD, TZK, zeta chain of T cell receptor associated protein kinase 70, zeta-chain (TCR) associated protein kinase, ζ chain of T cell receptor associated protein kinase 70, ζ-chain (TCR) associated protein kinase

Pathway Analysis Using IPA Software; canonical pathway

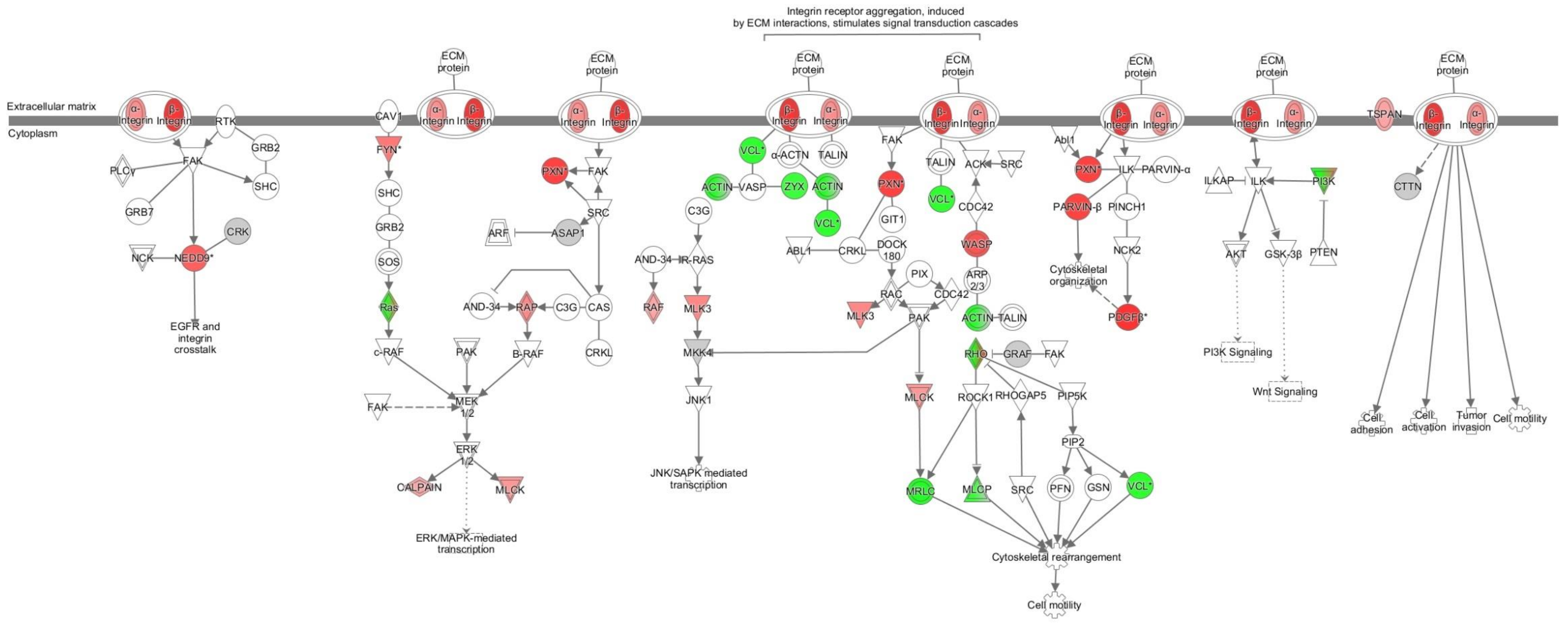

Figure S43. Integrin Signaling at 6 h

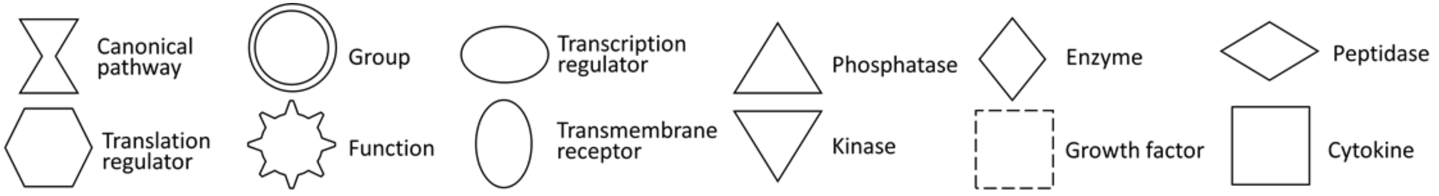

Red: Increased, FDR<0.05 versus control

Green: Decreased, FDR<0.05 versus control

Symbol	Synonym(s)
ABL1	ABL, ABL proto-oncogene 1, non-receptor tyrosine kinase, AI325092, BCR-ABL, c-A, c-ABL, CABL1, c-abl oncogene 1, non-receptor tyrosine kinase, CHDSKM, E430008G22Rik, JTK7, LOC100909750, p145Abl, p150, tyrosine-protein kinase ABL1-like, v-abl
ACTIN	CLEC9A Ligand, G-actin
AKT	AKT1/2/3, B/Akt, PKB, RAC-PK
Alphaactinin	ACTININ, Actinin alpha, Actinin α, ACTN, α-Actinin, α Actinin human
Alphaintegrin	Adhesion Receptors, alpha-Integrin, CD11, Cd11b/c, Integrin alpha, α- Integrin
ARHGAP26	1810044B20RIK, 2610010G17RIK, 4933432P15RIK, AI853435, GRAF, GRAF1, GTPASE REGULATOR ASSOCIATED with FOCAL ADHESION KINASE PP125(FAK), mKIAA0621, OLIGOPHRENIN-1 LIKE, OPHN1L, OPHN1L1, Rho GTPase activating protein 26
ARHGAP5	AU014947, GFI2, LRRGT00098, p190-, p190-B, p190BRhoGAP, p190Rhogap, p190RhoGAP-B, RhoGAP5, Rho GTPase Activating Protein 5
ARHGEF7	beta1PIX, betaPi, BETA-PIX, betaPix-b, betaPix-c, Beta-Pix Cool, C, coo, Cool, COOL-1, mKIAA0142, Nbla10314, P, P50, P50BP, p8, P85, P85 beta pix, P85COOL1, P85SPR, P85 β pix, PAK3, Pak3bp, PAK-INTERACTING EXCHANGE FACTOR beta, PAK-INTERACTING EXCHANGE FACTOR β, PIX, PIXB, Rho guanine nucleotide exchange factor 7, Rho guanine nucleotide exchange factor (GEF7), β-PIX, β Pix-a, β-Pix Cool
Arp2-3	Arp, Arp2-3, ARP2-3 (Actin-related protein complex), Arp Complex
ASAP1	AMAP1, ArfGAP with SH3 domain, ankyrin repeat and PH domain 1, AV239055, CENTB4, DDEF1, DEF-1, LOC100039024, mKIAA1249, PAG2, PAP, s19, ZG14P
BCAR1	AI385681, BCAR1 scaffold protein, Cas family member, breast cancer anti-estrogen resistance 1, C, CAS, CAS1, CASS1, Cr, CRKAS, LOC100131601, p130, P130CAP, P130CAS
BCAR3	AI131758, AND-, AND-34, BCAR3 adaptor protein, NSP family member, breast cancer anti-estrogen resistance 3, LOC101928013, MIG7, NSP2, RP11 488P31, SH2D3B
Betaintegrin	beta-Integrin, Integrin beta, β- Integrin
BRAF	9930012E13RIK, AA120551, AA387315, AA473386, AI447469, Bra, B-RAF1, Braf-2, B-Raf proto-oncogene, serine/threonine kinase, Braf transforming gene, C230098H17, C87398, D6Ertd631, D6Ertd631e, NS7, RAFB, RAFB1
CALPAIN	CALCIUM DEPENDENT PROTEASE, M calpain
CAV1	BSCL3, Cav, cave, Cavelolin 1, CAVEOLIN, Caveolin1, caveolin 1, caveolae protein, CGL3, LCCNS, LOC100362870, MSTP085, PPH3, VIP21
CDC42	AI747189, AU018915, CDC42Hs, cell division cycle 42, CELLULAR GROWTH REGULATING, G25K, TKS
CRK	c-Crk, c-Crk2, Cr, CRK2, Crko, CRK proto-oncogene, adaptor protein, FLJ11558, p38, v-crk avian sarcoma virus CT10 oncogene homolog
CRKL	1110025F07Rik, AA589403, AI325100, Cr, crk-like protein-like, CRK like proto-oncogene, adaptor protein, Crkol, LOC100911248, mgc94609, snoop, v-crk avian sarcoma virus CT10 oncogene homolog-like
CTTN	1110020L01Rik, amplaxin, Cortactin, Cttnb, Ems, EMS1
DOCK1	9130006G06Rik, AI854900, b2b3190C, b2b3190Clo, ced5, D630004B07Rik, dedicator of cyto-kinesis 1, Dock18, DOCK180, LOC679295, RGD1566072
ERK1/2	MAPK p44/42, MAPK p44/p42, p42/44 mapk, P42/p44 erk, P42/p44 mapk, p42/p44 MAP KINASE
FYN	AI448320, AW552119, C-FYN, Fyn proto-oncogene, FYN proto-oncogene, Src family tyrosine kinase, FYNT, LOC102724705, p59-FYN, p59 Fyn B, SLK, SRC-LIKE KINASE, SYN
GIT1	Cat-, Cat-1, GIT ArfGAP 1, p95C, p95Cat
GRB2	AA408164, ASH, Ash-psi, EGFRBP-GRB2, GRAB2, GRBS, growth factor receptor bound protein 2, MST084, MSTP084, NCKAP2
GRB7	growth factor receptor bound protein 7, mKIAA4028
GSK3B	7330414F15Rik, 8430431H08Rik, C86142, glycogen synthase kinase 3 beta, glycogen synthase kinase 3 β, GSK-, GSK-3, GSK-3be, GSK-3beta, GSK-3β, GSKbeta, GSKβ, Tpk1
GSN	ADF, AGEL, Gelsolin, Gelsolin plasma isoform, LOC105376337
ILK	AA511515, ESTM2, ESTM24, HEL-S-28, ILK-1, ILK-2, integrin-linked kinase, P59, p59ILK
ILKAP	0710007A14RIK, 1600009O09Rik, AF095927, AK055417, ILKAP2, ILKAP3, ILK associated serine/threonine phosphatase, integrin-linked kinase-associated serine/threonine phosphatase 2C, PP2C-D, PP2C-DELTA, PP2C-δ, PPM1O
Integrin	Integrin alpha-beta, integrin-extracellular matrix, INTEGRIN receptor, Integrin α-β
LIMS1	2310016J22Rik, 4921524A02Rik, AI507642, AU021743, AW551584, C430041B13RIK, Li, LIM and senescent cell antigen-like domains 1, LIM zinc finger domain containing 1, Lims1l, PIN, PINCH, PINCH-1, RGD1560732
MAP2K1/2	MEK1/2, MKK1/2
MAP2K4	JNKK, JNKK1, MAPK/ERK KINASE-1, MAPKK4, MEK4, mitogen-activated protein kinase kinase 4, MKK4, PRKMK4, SAPKK-1, Sek, SEK1, Ser, SERK1, SKK1
MAP3K11	2610017K16RIK, MEKK11, mitogen-activated protein kinase kinase kinase 11, Mlk, MLK-3, PTK1, RHOE, SPRK
MAPK8	AI849689, C-JUN N-TERMINAL KINASE1, JNK, JNK1, JNK1A2, JNK21B1/2, JNK-46, mitogen-activated protein kinase 8, p46JNK1, p46JNK1 alpha, p46JNK1 α, Prk, PRKM8, SAPK1, SAPK1c, Sapk gamma, SAPK P46, Sapk γ, STRESS-ACTIVATED protein KINASE-LIKE KINASE
MLCP	3.1.3.53, Myosin-bound phosphatase, myosin light chain kinase phosphatase, myosin-light-chain-phosphatase, [myosin-light-chain]-phosphate phosphohydrolase, Myosin Phosphatase, Myosin PPTase, MYPT, protein phosphatase 2A
MRLC	Myosin subunit regulatory light chain, Rlc
Mylk	MLCK, Mylk
NCK	NCK alpha,beta, NCK α,β
NCK2	4833426I10RIK, Grb, GRB4, LOC100503894, NCK adaptor protein 2, NCKbe, NCKbeta, Nck β, non-catalytic region of tyrosine kinase adaptor protein 2
NEDD9	C, Ca, CAS2, CAS-L, CASS2, enhancer of filamentation 1, HEF1, MEF1, neural precursor cell expressed, developmentally down-regulated 9, neural precursor cell expressed, developmentally down-regulated gene 9, p105, P105hef1
PARVA	2010012A22Rik, 5430400F08Rik, act, Actopaxin, Actp, AI225929, alpha PARVIN, AU042898, CH-IL, CH-ILKBP, MXRA2, Parvin, parvin, alpha, parvin, α, Parvin-alpha, Parvin-α, α PARVIN
PARVB	aff, affixin, AI595373, AW742462, CGI-56, D15Gsk1, D15Gsk, parvin, beta, parvin, β, Parvin-beta, Parvin-β
PDGFB	c-sis, IBGC5, PDGF-, PDGF-2, PDGF-BB, PDGF beta, PDGFbetaR, Pdgfrb, PDGFRbeta, PDGF-β, platelet derived growth factor, B polypeptide, platelet derived growth factor subunit B, SIS, SSV
PI3K	1-phosphatidylinositol 3-kinase, 2.7.1.137, ATP:1-phosphatidyl-1D-myo-inositol 3-phosphotransferase, Phosphatidylinositol 3 kinase, phosphatidylinositol 3'-kinase, PI3-kinase, PtdIns 3 Kinase, type III phosphoinositide 3-kinase, type I phosphatidylinositol kinase, Vps34p
PIKFYVE	5230400C17RIK, CFD, FAB1, HEL37, KIAA0981, P, p235, phosphoinositide kinase, FYVE-type zinc finger containing, PI5K, Pip, PIP5K, PIP5K3, Pipk5k3, PipkIII, Type III PI 5-kinase, ZFYVE29
PIP2	C11H19O19P3R2, phosphatidylinositol-4,5-bisphosphate, phosphatidyl-myo-inositol 4,5-bisphosphate, PI(4,5)P2, PI4,5P2, PIP2, PtdIns(4,5)P2
PLC-gamma	Phospholipase C gamma, Phospholipase C γ, PLCG, PLCγ
Profilin	PFN
PTEN	10q23del, 2310035O07RIK, A130070J02Rik, AI463227, B430203M17RIK, BZS, CWS1, DEC, GLM2, MHAM, MMAC, MMAC1, MUTATED IN MULTIPLE ADVANCED CANCERS, mutated in multiple advanced cancers 1, phosphatase and tensin homolog, PTEN1, PTENbeta, TEP, TEP1
PTK2	FA, Fad, FADK, FADK 1, FAK, FAK1, FAK related non-kinase, FR, p125FAK, pp125FAK, PPP1R71, protein tyrosine kinase 2, PTK2 protein tyrosine kinase 2, TYROSINE KINASE 2
PXN	AW108311, AW123232, FLJ23042, P, PAX, PAXILLIN
RAF1	6430402F14Rik, AA990557, BB129353, CMD1NN, c-R, Cra, CRAF, Craf1, D830050J10Rik, leukaemiA ONCOGENE HOMOLOG1, LEUKEMIA ONCOGENE HOMOLOG1, NS5, Raf-1 proto-oncogene, serine/threonine kinase, v-, v-Raf, v-raf-leukaemia viral oncogene 1, v-raf-leukemia viral oncogene 1
Ral	Ral A/B
RAPGEF1	4932418O06Rik, C3G, C3G-1, C3G-2, Grf, GRF2, Rap guanine nucleotide exchange factor 1, Rap guanine nucleotide exchange factor (GEF) 1
RHO	GTPase Rho, Rho, Rho Family, RHO-GTPASE, Rho-like Gtpase
ROCK1	1110055K06Rik, LOC100129157, P160ROCK, p160 ROCK-1, Rho-associated coiled-coil containing protein kinase 1, Roc, ROCK, ROCK-I, ROK, ROK beta, ROK β
RRAS	AI573426, p23, R, RAS related, related RAS viral (r-ras) oncogene, Rras1, Rras predicted
SHC1	p52SHC, p6, p66, p66s, P66shc, Sh, SHC, Shc (46 kDa isoform), SHCA, SHC adaptor protein 1, Shc p66 isoform, src homology 2 domain-containing transforming protein C1
SRC	ASV, AW259666, BS27, c-SRC, p60-Src, PP60, pp60c, Pp60/c-Src, pp60c-src, Rous sarcoma oncogene, SRC1, SRC proto-oncogene, non-receptor tyrosine kinase, THC6, TVHUSC
TALIN	TLN
TNK2	Ac, ACK, ACK1, Cdgip, LOC682784, p21cdc42Hs, Pyk, Pyk1, tyrosine kinase non receptor 2, tyrosine kinase, non-receptor, 2
TSPAN	TETRASPAN, TRANSMEMBRANE 4 SUPERFAMILY
VASP	vasodilator-stimulated phosphoprotein
VCL	9430097D22, AA571387, AI462105, AW545629, CMD1W, CMH15, HEL114, MV, MVCL, Vcl predicted, Vinculin
ZYX	9530098H06Rik, ESP-2, HED-2, R7515, R75157, ZIXIN, Zyxin

Pathway Analysis Using IPA Software; canonical pathway

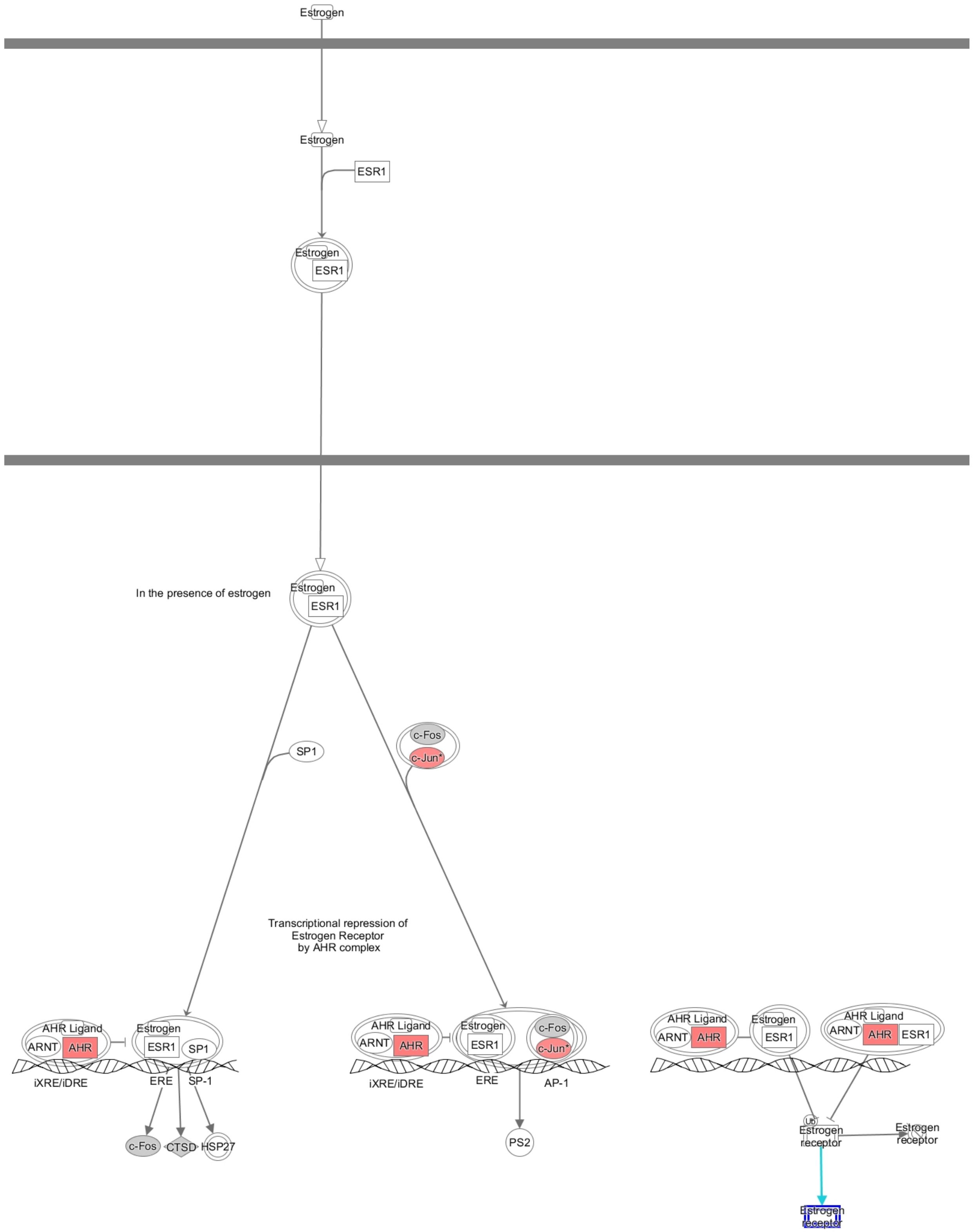

Figure S44. Aryl Hydrocarbon Receptor Signaling at 6 h

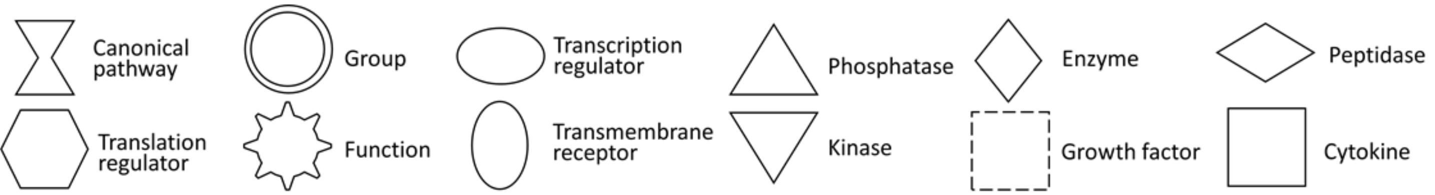

Red: Increased, FDR<0.05 versus solvent control

Green: Decreased, FDR<0.05 versus solvent control

Pathway Analysis Using IPA Software; canonical pathway

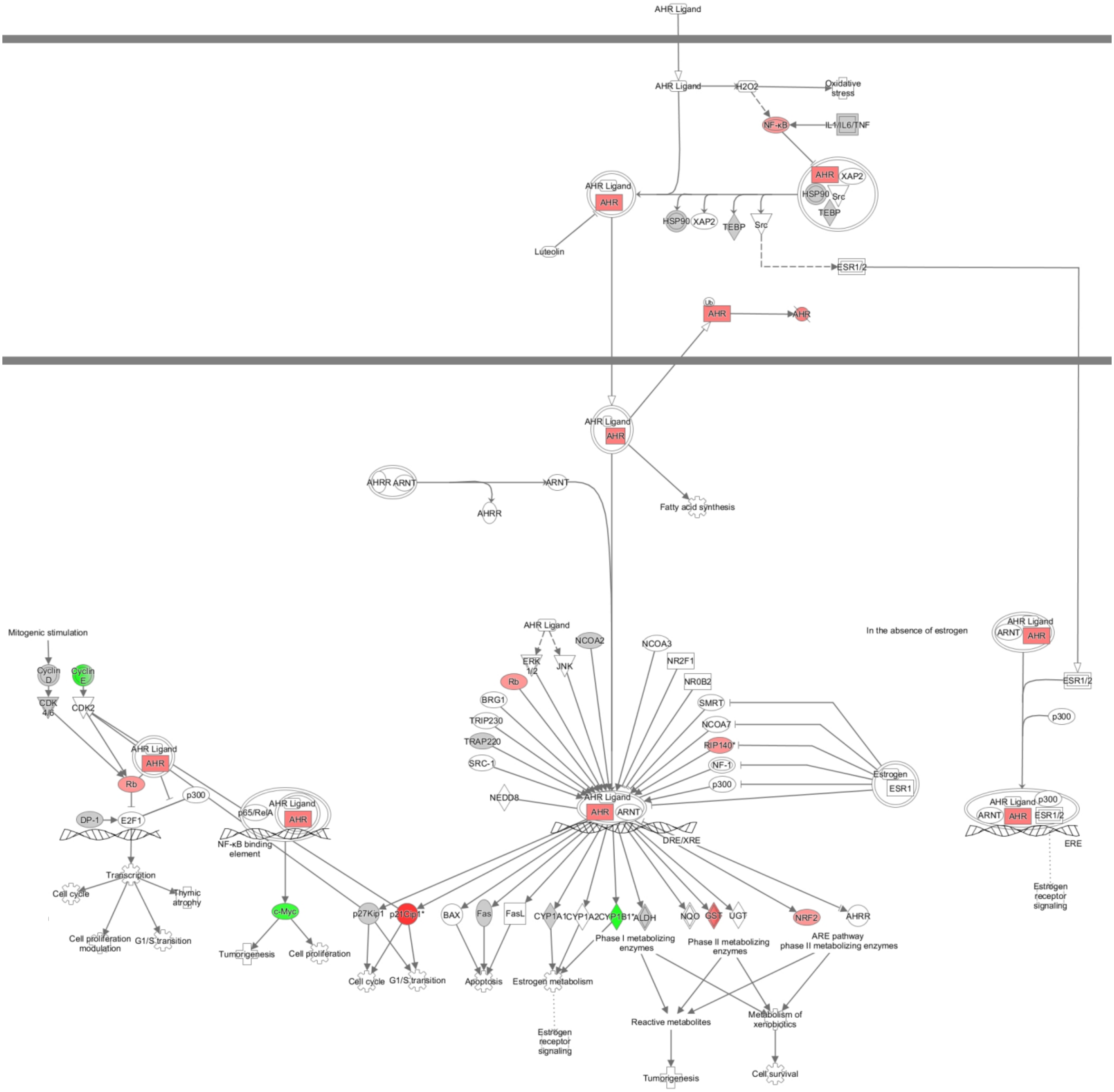

Figure S44. Aryl Hydrocarbon Receptor Signaling at 6 h (continued)

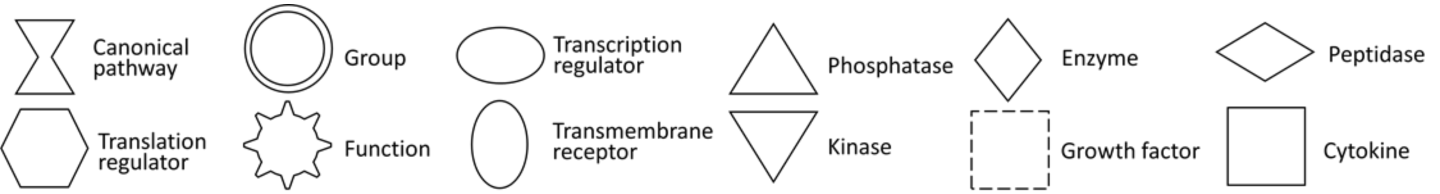

Red: Increased, FDR<0.05 versus solvent control

Green: Decreased, FDR<0.05 versus solvent control

Pathway Analysis Using IPA Software; canonical pathway

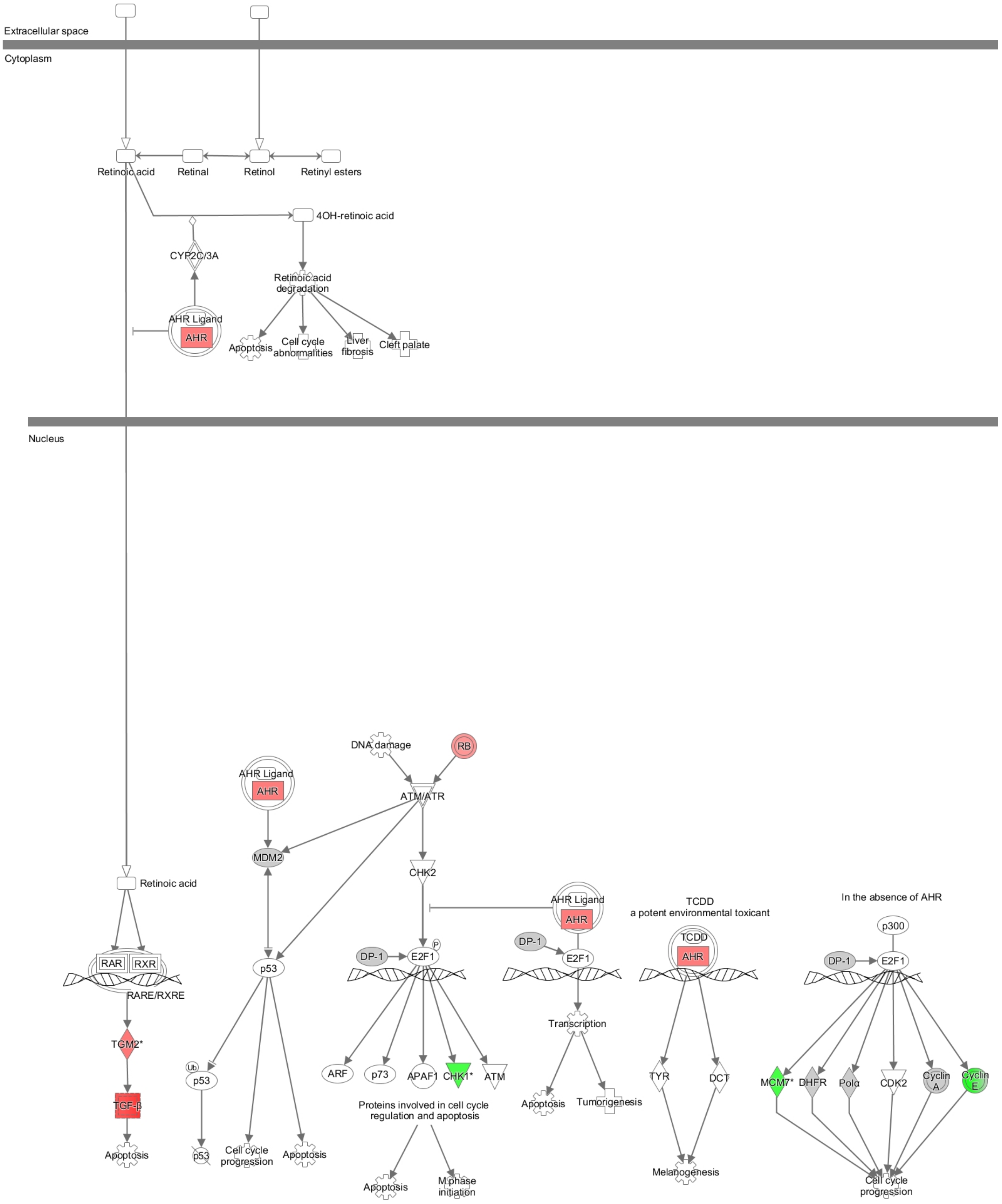

Figure S44. Aryl Hydrocarbon Receptor Signaling at 6 h (continued)

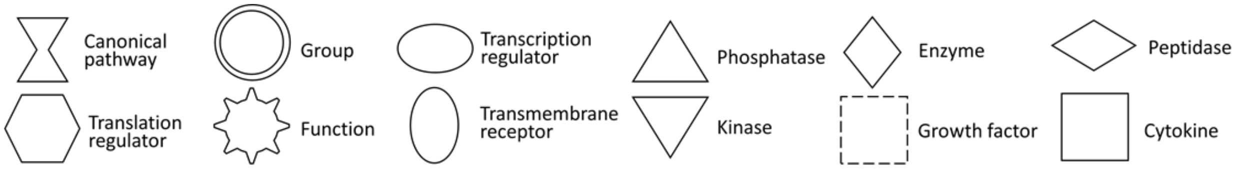

Red: Increased, FDR<0.05 versus solvent control

Green: Decreased, FDR<0.05 versus solvent control

Symbol	Synonym(s)
4OH-retinoic acid	(2E,4E,6E,8E)-9-(3-hydroxy-2,6,6-trimethylcyclohexen-1-yl)-3,7-dimethylnona-2,4,6,8-tetraenoic acid, 4OH-retinoic acid, 66592-72-1, C20H28O3
AHR	A, Ah, Ahh, Ahre, AH receptor, aryl-hydrocarbon receptor, bHLHe7, bHLHe76, DIOXIN receptor, In, RP85
AHR Ligand	AHR ligand, aromatic hydrocarbon
AHRR	AHH, AHHR, aryl-hydrocarbon receptor repressor, bHLHe77, mKIAA1234
AIP	A, AA408703, ARA9, aryl-hydrocarbon receptor-interacting protein, AW476050, D19Bwg1412e, Fkbp1, FKBP16, FKBP37, PITA1, SMTPHN, Xa, XAP-2
ALDH	ALDEHYDE DEHYDROGENASE
Ap1	activator protein-1, c-Jun
APAF1	6230400I06RIK, Ap, Apaf1l, apoptotic peptidase activating factor 1, CED4, fog, mKIAA0413
ARNT	Arnt1, aryl hydrocarbon receptor nuclear translocator, bHLHe, bHLHe2, D3Ertd557, D3Ertd557e, DIOXIN receptor, Drnt, ESTM4, ESTM42, Hif1, HIF1B, HIF1BETA, HIF-1-β, HIF beta, HIF β, mKIAA4051, TANGO, W08714
ATM	AI256621, AT1, ATA, ataxia telangiectasia mutated, ATC, ATD, ATDC, ATE, ATM serine/threonine kinase, C030026E19RIK, TEL1, TELO1
ATM/ATR	ATR/ATM
BAX	Bcl2-associated X, BCL2 associated X, apoptosis regulator, BCL2-associated X protein, BCL2L4
CDK2	A630093N05Rik, CDC2-RELATED KINASE, CDKN2, Cyclin A associated kinase, cyclin-dependent kinase 2, CYCLIN E ASSOCIATED KINASE, p33(CDK2)
CDKN1A	CAP, CAP20, CDK, CDKI, Cdkn, CDKN1, CDKNA1, CI, CIP1, cyclin-dependent kinase inhibitor 1A, cyclin-dependent kinase inhibitor 1A (P21), mda, MDA-6, P2, P21, p21C, p21Cip, p21CIP1, p21W, p21WAF, p21Waf1, Pzl Cyclin-Dependent Kinase Inhibitor, SD, SDI1, UV96, Waf, WAF1
CDKN1B	AA408329, AI843786, Cdki1b, CDKN4, cyclin-dependent kinase inhibitor 1B, CYCLIN-DEPENDENT KINASE INHIBITOR P27, KIP1, MEN1B, MEN4, p2, p27, p27K, P27kip, P27KIP1, P28-ICK
CHEK1	C85740, checkpoint kinase 1, CHK1, rad27
CHEK2	CDS1, Check2, checkpoint kinase 2, CHK2, hCds1, HUCDS1, LFS2, PP1425, Rad, RAD53
CTSD	Cat, CATD, cathepsin D, Cathespin D, CD, CLN10, CPSD, EAI, HEL-S-130P, LOC196214
CyclinD	CycD, Cyclin D1
CYP1A1	AHH, AH Hydroxylase, AHRR, CP11, CYP1, Cyp45c, Cypc45c, CYPIA1, cytochrome P450 family 1 subfamily A member 1, cytochrome P450, family 1, subfamily a, polypeptide 1, EROD, P1-450, P450-, P450-1, P-450bnf-b, P450-C, P450DX, P450 IA1, P-450MC, P450-P1
CYP1A2	CP1, CP12, CYPD45, CYPIA2, cytochrome P450 family 1 subfamily A member 2, cytochrome P450, family 1, subfamily a, polypeptide 2, P3-450, P450-, P450-3, P-450d, P450 IA2, P-450isf-g, P450-P3, P450(PA), RATCYPD45
CYP1B1	ASGD6, CP1B, CYPIB1, cytochrome P450 family 1 subfamily B member 1, cytochrome P450, family 1, subfamily b, polypeptide 1, GLC3A, P4501B1, P450Rap
DCT	dopachrome tautomerase, DT, LOC102724113, OCA8, RGD1564975, slaty, slt, TR, TRP, TRP-2, Tyr, Tyrp, TYRP2
DHFR	8430436I03RIK, AA607882, AI662710, AW555094, Dhfr1, DHFR4, DHFRP1, dihydrofolate reductase, dihydrofolic acid reductase, DYR
E2F1	E2f, E2F transcription factor 1, mKIAA4009, RBAP1, RBBP3, RBP3, Tg(Wnt1-cre)2Sor
EP300	A430090G16, A730011L11, E1A binding protein p300, KAT3, KAT3B, MKHK2, p30, p300, p300 HAT, RSTS2
ERK1/2	MAPK p44/42, MAPK p44/p42, p42/44 mapk, P42/p44 erk, P42/p44 mapk, p42/p44 MAP KINASE
ESR1	Alpha estrogen receptor, E, ER-, ERa, ER-alpha, Er alpha (46 kDa isoform), ER-α, Er α (46 kDa isoform), Es, ESR, ESRA, Estr, Estra, estrogen receptor, estrogen receptor 1, estrogen receptor 1 (alpha), estrogen receptor 1 (α), Estrogen receptor α, ESTRR, Nr, NR3A1, RNESTROR, TERP-1, α estrogen receptor
Estrogen	C18 steroids, oestrogen
Estrogenreceptor	ER, ESR, ESR1/2, esr1/esr2
Estrogen-ER	Esr1-Estrogen
FAS	AI196731, ALPS1A, AP, APO-1, APT1, CD95, CD95L, CD95 receptor, FAS1, FAS/APO1, Fas cell surface death receptor, FasR, FASTM, Fas (TNF receptor superfamily member 6), lpr, Receptor for Fas Ligand, Receptors for Fas Ligand, TNF, Tnfr, TNFR6, Tnf receptor member 6, TNFRSF6
FASLG	ALPS1B, APT1, APT1LG1, APTL, CD178, CD95, CD95-L, F, Fa, FASL, Fas Ligand, Fas ligand (TNF superfamily, member 6), gld, mFasL, Tnfl6, Tnfs, TNFSF6, TNLG1A
FOS	AP-1, c-f, C-FOS, D12Rfj, D12Rfj1, FBJ osteosarcoma oncogene, Fos proto-oncogene, AP-1 transcription factor subunit, p55
GST	Glutathione s-transferase, GSH Transferase
H2O2	7722-84-1, A-101, Colgate Peroxyl, Eskata, H2O2, hydrogen dioxide, hydrogen peroxide, peroxyl mouthwash, urea hydrogen peroxide
HSP27	Heat Shock Protein 27
HSP90	HSC90, Hsp84
JUN	Activator protein 1, AP-1, API-1, c-ju, cJUN, Junc, jun proto-oncogene, Jun proto-oncogene, AP-1 transcription factor subunit, LOC100288387, LOC100291417, LOC100293034, p39, v-Jun, V-jun Avian Sarcoma Virus 17 Oncogene Homolog, V-jun Sarcoma Virus 17 Oncogene Homolog
Luteolin	2-(3,4-dihydroxyphenyl)-5,7-dihydroxy-4-benzopyrone, 2-(3,4-dihydroxyphenyl)-5,7-dihydroxychromen-4-one, 3',4',5,7-tetrahydroxyflavone, 491-70-3, 4H-1-benzopyran-4-one, 2-(3,4-dihydroxyphenyl)-5,7-dihydroxy- (9Cl), C15H10O6, cyanidenon 1470, digitoflavone, flacitran, luteolol
MAPK8	AI849689, C-JUN N-TERMINAL KINASE1, JNK, JNK1, JNK1A2, JNK21B1/2, JNK-46, mitogen-activated protein kinase 8, p46JNK1, p46JNK1 alpha, p46JNK1 α, Prk, PRKM8, SAPK1, SAPK1c, Sapk gamma, SAPK P46, Sapk γ, STRESS-ACTIVATED protein KINASE-LIKE KINASE
MCM7	AI747533, CDC47, D16Mgi24, mCDC47, MCM2, Mcmd7, minichromosome maintenance complex component 7, P1.1-MCM3, P1CDC47, P85MCM, PNAS146, PPP1R104
MDM2	1700007J15Rik, AA415488, ACTFS, hdm2, HDMX, LSKB, MDM2-A1, MDM2 proto-oncogene, MGC5370, Transformed 3t3 cell double minute 2, transformed mouse 3T3 cell double minute 2
MED1	AI480703, ARC205, CRSP, CRSP1, CRSP200, CRSP205, CRSP210, DRIP, DRIP205, DRIP230, l11Jus, l11Jus15, Med220, mediator complex subunit 1, P, PBP, PPARBP, PPARGBP, RB18A, RGD1559552, TRAP, TRAP220, TRIP-2
MYC	AU016757, bHLHe3, bHLHe39, CMYC, C-MYC-P64, mMyc, MRTL, Myc2, MYCC, MYC proto-oncogene, bHLH transcription factor, myelocytomatosis oncogene, N, Niard, Nird, RNCMYC
NCOA2	bHLHe7, bHLHe75, D1Ertd433, D1Ertd433e, Gr, GRIP-1, KAT13, KAT13C, nuclear receptor coactivator 2, SRC, SRC-2, TIF-, TIF-2, TIF2/GR
NCOA3	2010305B15RIK, ACTR, AI, AIB-1, AW321064, bHLHe4, bHLHe42, CAGH16, CTG26, KAT13, KAT13B, nuclear receptor coactivator 3, p, pCIP, p/Cip, RA, RAC3, Sr, SRC-1, SRC-3, TNRC14, TNRC16, TRA, TRAM, TRAM-1
NCOA7	9030406N13RIK, dJ187J11.3, ERAP140, ESNA1, Nbla00052, Nbla10993, NCOA7-AS, nuclear receptor coactivator 7, RGD1566426, TLDC4
NEDD8	CDK8, NEDD8 ubiquitin like modifier, neural precursor cell expressed, developmentally down-regulated gene 8, Rub, Rub1, Similar to nedd8
NF-1	NF-1, Nfi
NFE2L2	BM974200, HEBP1, IMDDHH, Nr, NRF2, nuclear factor, erythroid 2-like 2, nuclear factor, erythroid derived 2, like 2
NFkB	NF-KAPPA B, NF-κ B, nuclear factor-κ b, transcription factor nuclear factor κ b
NQO	Nadph-d, NADPH QUINONE OXIDOREDUCTASE
NR0B2	nuclear receptor subfamily 0 group B member 2, nuclear receptor subfamily 0, group B, member 2, S, SHP, SHP-1
NR2F1	BBOAS, BBSOAS, COUP-, COUP-T, COUP-TF1, COUPTFA, COUP-TFI, EAR-3, Erb, ERBAL3, nuclear receptor subfamily 2 group F member 1, nuclear receptor subfamily 2, group F, member 1, SVP44, Tcfcou, TCFCOUP1, TFCOUP1
NRIP1	6030458L20Rik, 8430438I05RIK, 9630050P12, AA959574, AW456757, CAKUT3, NUCLEAR FACTOR receptor INTERACTING protein 140, nuclear receptor interacting protein 1, RIP, RIP140
p19 Arf	A, Arf, ARF-INK4a, CDK4I, CDKN2, CMM2, CYCLIN-DEPENDENT KINASE INHIBITOR 2A, INK4, INK4A, INK4a-ARF, Ink4a/Arf, MLM, MTS, MTS-1, p1, p14ARF/ p16INK4a, p16, p16/ARF, p16Cdkn2a, p16I, p16 INK4, p16/ INK4a, P19, p19ARF, Pct, PCTR1, TP16
POLA1	alpha PRIMASE p180, AW321876, DNA polymerase alpha, DNA polymerase alpha 1, catalytic subunit, DNA polymerase alpha subunit 1, DNA polymerase α, DNA polymerase α 1, catalytic subunit, DNA polymerase α subunit 1, NSX, p180, POLA, Polalpha, POLYMERASE alpha, polymerase (DNA directed), alpha 1, polymerase (DNA directed), α 1, POLYMERASE α, Polα, VEODS, α PRIMASE p180
PTGES3	5730442A20Rik, cPG, cPGES, Gm9769, p23, p23 COCHAPERONE, p23 PR RELATED, PGES3, prostaglandin E synthase 3, prostaglandin E synthase 3, pseudogene, Ptg, Ptges, Ptges3-ps, RGD1561913, sid31, sid3177, Teb, TEBP, Telomerase Binding Protein p23, Zhf6
RAR	retinoic acid nuclear receptor, RETINOIC ACID NUCLEAR receptors, retinoic acid receptor, retinoic acid receptors
RB	pRb, Rb Tumor Suppressor, Rb tumour Suppressor
RB1	OSRC, p, p105, p105-Rb, p110 RB, p110-RB1, pp105, pp110, PPP1R130, pRb, R, RB, RB-ASSOCIATED, RB transcriptional corepressor 1, Retinoblastome tumor-suppression protein rb
RELA	CMCU, NF-kappa B, NF-kappa B (p65), NF KAPPA B subunit P65, NFkB, NFKB3, NF-k B (p65), NFkB/p65, NF-κ B, NF-κ B (p65), NF-κB p65, NF κ B subunit P65, nos2, p6, p65, p65 NF-kappa B, p65 NFkB, p65 NF-κ B, p65/Rela, RELA proto-oncogene, NF-kB subunit, v-rel reticuloendotheliosis viral oncogene homolog A (avian)
Retinal	116-31-4, (2E,4E,6E,8E)-3,7-dimethyl-9-(2,6,6-trimethylcyclohexen-1-yl)nona-2,4,6,8-tetraenal, all-trans-retinal, all-trans-retinaldehyde, atRAL, C20H28O, retinal, retinal, all-trans-, vitamin A aldehyde
Retinoic acid	13497-05-7, 187175-63-9, 22232-80-0, (2E,4E,6E,8E)-3,7-dimethyl-9-(2,6,6-trimethylcyclohexen-1-yl)nona-2,4,6,8-tetraenoic acid, 302-79-4, 56573-65-0, 7005-78-9, 75980-27-7, all-trans RA, all-trans-retinoate, all-trans retinoic acid, all-trans-retinoic acid liposomal, Altinac, Altreno, AR-623, atra, Atragen, atralin, Atralin Gel, Avita, beta all trans retinoic acid, C20H28O2, liposomal all-trans-retinoic acid, liposomal tretinoin, Renova, Retin A, Retin-A, Retin-A Micro, Retin A Micro Gel, retinoic acid, retinoic acid, all-trans-, retinoic acid, sodium salt, trans retinoic acid, tretinoinLF, tretinoin liposomal, tretinoin liposome, tretinoin potassium salt, tretinoin sodium salt, Tretinoin Topical, tretinoin zinc salt, Vesanoid, vitamin A acid, β all trans retinoic acid
Retinol	11103-57-4, (2E,4E,6E,8E)-3,7-dimethyl-9-(2,6,6-trimethylcyclohexen-1-yl)nona-2,4,6,8-tetraen-1-ol, 3,7-dimethyl-9-(2,6,6-trimethyl-1-cyclohexen-1-yl)-2,4,6,8-nonate-traen-1-ol, 68-26-8, all-trans retinol, C20H30O, vitamin A, vitamin-A (all-trans-retinol)
Retinyl esters	C21H29O2R, retinyl esters
RXR	Retinoid receptor, RXR alpha/beta/gamma, RXR α/β/γ
SLC35A2	AI327289, CDG2M, CDGX, Had-, Had-1, Sfc, Sfc8, solute carrier family 35 member A2, UDP-Gal-Tr, Uga, UGALT, UGAT, UGT, UGT1, UGT2, UGTL
SMARCA4	b2b508.1C, b2b508.1Clo, b2b692C, b2b692Clo, BAF190, BAF190A, BRG, BRG1, CSS4, HP1-BP72, hSNF2b, MRD16, RTPS2, SNF2, SNF2b, SNF2-beta, SNF2L4, SNF2LB, SNF2-β, SW1/, SW1/SNF, SWI2, SWI/SNF related, matrix associated, actin dependent regulator of chromatin, subfamily a, member 4
SMRTalpha	CTG26, N-CoR, nuclear receptor co-repressor 2, RETINOID SILENCER, SM, SMAP270, SMR, SMRT, SMRTE, SMRTE-tau, TNRC14, TRAC, TRAC-1
SP1	1110003E12RIK, AA450830, AI845540, Sp1-1, Sp1 transcription factor, Sp1 (trans spliced isoform), Trans-acting transcription factor 1
SRC	ASV, AW259666, BS27, c-SRC, p60-Src, PP60, pp60c, Pp60/c-Src, pp60c-src, Rous sarcoma oncogene, SRC1, SRC proto-oncogene, non-receptor tyrosine kinase, THC6, TVHUSC
TCDD	1746-01-6, 2,3,7,8-techachlorodibenzo-p-dioxin, 2,3,7,8-tetrachlorodibenzo(b,e)(1,4)dioxin, 2,3,7,8-tetrachlorodibenzo-p-dioxin, C12H4Cl4O2, dibenzo(b,e)(1,4)dioxin, 2,3,7,8-tetrachloro-, dibenzo-p-dioxin, 2,3,7,8-tetrachloro-, dioxin, TCDD
TFDP1	DILC, Dp, DP-1, Drtf, DRTF1, TB2/DP1, transcription factor Dp-1
TFF1	Bce, BCEI, D21S21, HP1.A, HPS2, NEPHROCALCIN, P, pNR-2, PS2, TIFF1, trefoil factor 1
Tgfbeta	Tgfb, TGF-beta 1, 2, and 3, TGF β, TGF-β 1, 2, and 3, transforming growth factor-β
TGM2	Ftg, G[a], G[a]h, TG2, TGA2, TGas, TGASE, TGase2, TGase C, TgaseII, TGC, TG(C), TISSUE TRANSGLUTAMINASE, TISSUE TRANSGLUTAMINASE 2, Tranglutaminase 2, Transglutaminase, TRANSGLUTAMINASE 2, transglutaminase 2, C polypeptide, tTG, tTGas, T-Tgase
TP53	bbl, BCC7, bfy, bhy, BMFS5, LFS1, p4, p44, p5, P53, P53 cellular tumour antigen, p53 tumor suppressor, transformation related protein 53, TRP53, tumor protein p53, tumour protein p53
TRIP11	2610511G22Rik, 3110031G15Rik, 6030460N08RIK, ACG1A, AI450776, CEV14, GMAP-2, GMAP-210, ODCD, THR COACTIVATOR, thyroid hormone receptor interactor 11, TRIP, TRIP230
Trp73	delta, p7, P73, p73RhoGAP, TAp, transformation related protein 73, Trp73, tumor protein p73, tumour protein p73, δ
TYR	albino, ATN, CMM8, Dopa oxidase, Melanogenesis Related Tyrosinase, Oc, OCA1, OCA1A, OCAIA, SHEP3, skc3, skc35, tyrosinase

Pathway Analysis Using IPA Software; canonical pathway

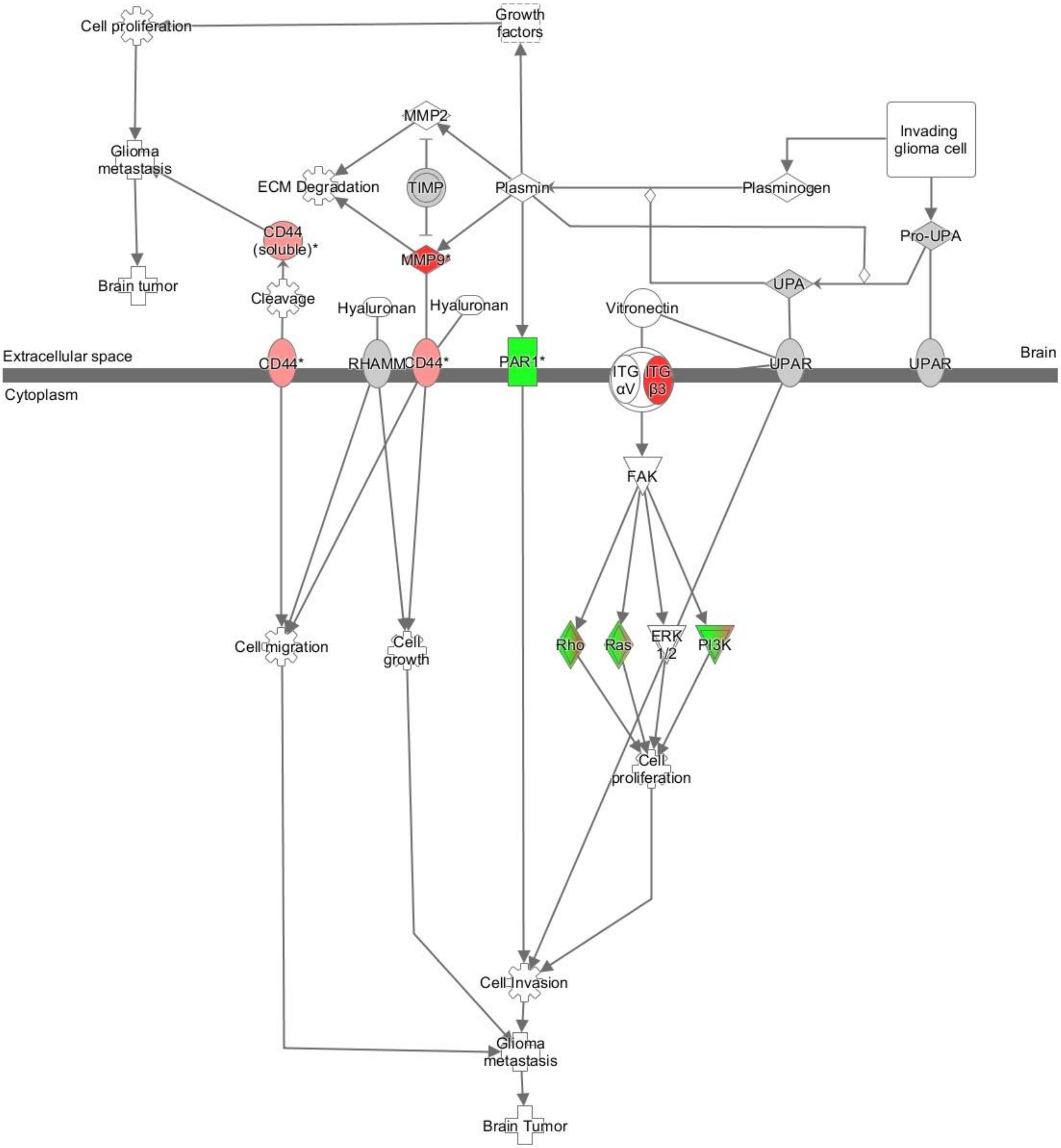

Figure S45. Glioma Invasiveness Signaling at 6 h

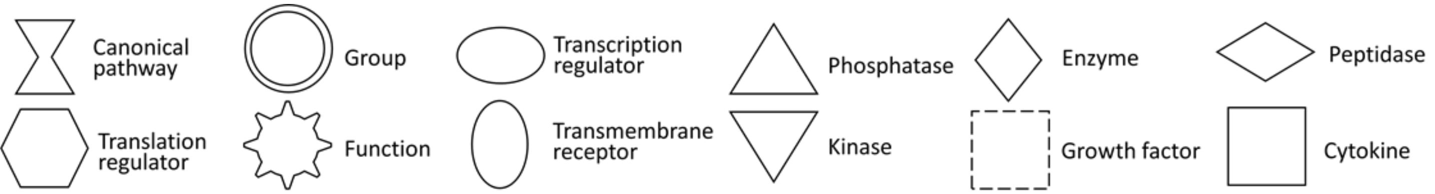

Red: Increased, FDR<0.05 versus solvent control

Green: Decreased, FDR<0.05 versus solvent control

Symbol	Synonym(s)
CD44	216062 AT, AU023126, AW121933, AW146109, CD44A, CD44 Antigen, CD44 (containing exon 5), Cd44i, CD44 molecule (Indian blood group), CD44 (soluble), CD44 STANDARD FROM, CDW44, CSPG8, ECMR-III, Epican, HCELL, HERM, HERMES, Hermes antigen, HUTCH-I, IN, LHR, Ly-2, Ly-24, MC56, MDU2, MDU3, METAA, MIC4, NKT.44, Pgp, Pgp-1, RHAMM
ERK1/2	MAPK p44/42, MAPK p44/p42, p42/44 mapk, P42/p44 erk, P42/p44 mapk, p42/p44 MAP KINASE
F2R	AI482343, Cf2, CF2R, coagulation factor II (thrombin) receptor, coagulation factor II thrombin receptor, HTR, P, PAR-1, Th, ThrR, TR, TRGPC, α Thrombin Receptor
HMMR	AA386826, CD168, hyaluronan-mediated motility receptor, hyaluronan mediated motility receptor (RHAMM), IHABP, Rha, RHAMM
Hyaluronan	34448-35-6, 9004-61-9, 9067-32-7, Amvisc, Amvisc Plus, Biolon, C28H44N2NaO23+, Duovisc, etamucine, EUFLEXXA, HA, Healon, Healon5, Hyalgan, hyaluronan, hyaluronan acid, hyaluronate, hyaluronate sodium, hyaluronic acid oligosaccharide, hyaluronic acid, sodium salt, Hyruan Plus, Hyvisc, luronit, Orthovisc, Provisc, sodium;(2S,3S,4S,5R,6R)-6-[(2S,3R,4R,5S,6R)-3-acetamido-2-[(2S,3S,4R,5R,6R)-6-[(2R,3R,4R,5S,6R)-3-acetamido-2,5-dihydroxy-6-(hydroxymethyl)oxan-4-yl]oxy-2-carboxy-4,5-dihydroxyoxan-3-yl]oxy-5-hydroxy-6-(hydroxymethyl)oxan-4-yl]oxy-3,4,5-trihydroxyoxane-2-carboxylic acid, sodium hyaluronate, Viscoat, Vitrax
Integrin alpha-V beta 3	alpha-v beta-3, alpha V beta 3 Integrin, Integrin-alpha-beta3, Integrin-α-beta3, Integrin α V beta3, Integrin α V β 3, Vitronectin Receptor, VnR, α-5-beta3, α 5 β 3, α-v β-3, α V β 3 Integrin
ITGAV	1110004F14RIK, 2610028E01Rik, alpha V, CD51, D430040G12RIK, integrin alpha V, integrin subunit alpha V, integrin subunit α V, Integrin α V, MSK8, VNRA, VTNR, α V
ITGB3	BDPLT16, BDPLT2, beta 3, CD61, GP3A, GPIIIa, GT, HPA-4, INGRB3, integrin beta 3, integrin subunit beta 3, integrin subunit β 3, Integrin-β 3, β 3
MMP2	Clg, CLG4, CLG4A, Ge, GelA, GELATINASE, Gelatinase A, matrix metallopeptidase 2, METALLOPROTEINASE 2, MMP-, MMP-II, MONA, TBE-1
MMP9	AW743869, B/MMP, B/MMP9, Clg4, CLG4B, COLLAGENASE type IV, Gelatinase B, GELB, GI 92-kda, MANDP2, matrix metallopeptidase 9, METALLOPROTEINASE 9, MMP-, pro-MMP-9
PI3K	1-phosphatidylinositol 3-kinase, 2.7.1.137, ATP:1-phosphatidyl-1D-myo-inositol 3-phosphotransferase, Phosphatidylinositol 3 kinase, phosphatidylinositol 3'-kinase, PI3-kinase, PtdIns 3 Kinase, type III phosphoinositide 3-kinase, type I phosphatidylinositol kinase, Vps34p
PLAU	ATF, BDPLT5, plasminogen activator, urokinase, Pro-UPA, QPD, u-, UPA, uPA 50 kd form, UPA-H, UPAM, URK
PLAUR	CD87, Par, plasminogen activator, urokinase receptor, Plaur3, u-, U-PAR, uPAR-2, UPAR-3, Urinary plasminogen activator receptor 2, URKR, UROKINASE R, Urokinase-type plasminogen activator receptor
PLG	Ab1-346, AI649309, GLU-PG, LPA, P, Pg, PG2, plasminogen, Scdp
PTK2	FA, Fad, FADK, FADK 1, FAK, FAK1, FAK related non-kinase, FR, p125FAK, pp125FAK, PPP1R71, protein tyrosine kinase 2, PTK2 protein tyrosine kinase 2, TYROSINE KINASE 2
Rho	GTPase Rho, Rho, Rho Family, RHO-GTPASE, Rho-like Gtpase
VTN	Aa1018, AI256434, V75, vitronectin, VN, VNT

Pathway Analysis Using IPA Software; canonical pathway

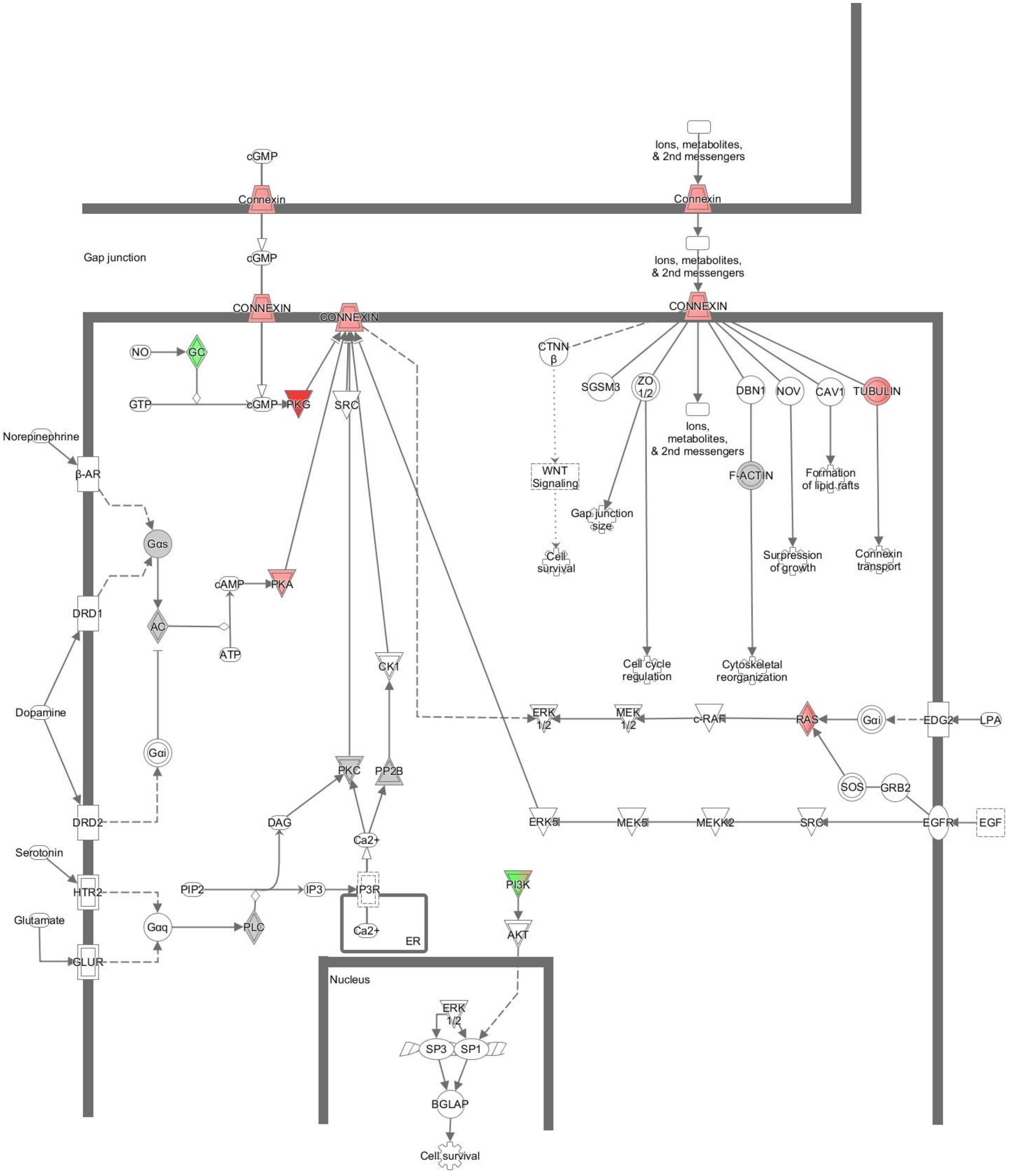

Figure S46. Gap Junction Signaling at 24 h

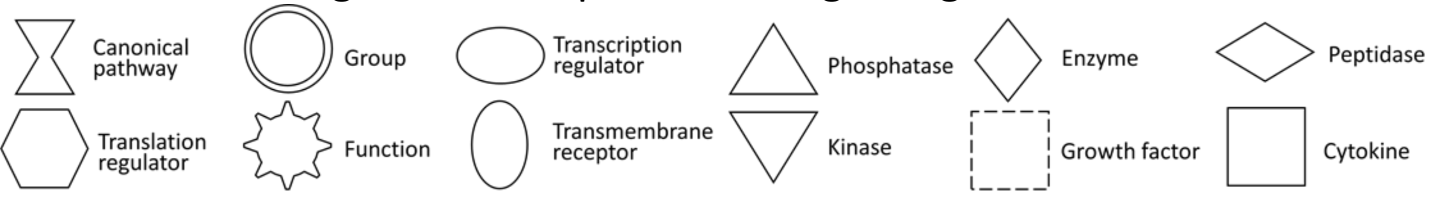

Red: Increased, FDR<0.05 versus solvent control

Green: Decreased, FDR<0.05 versus solvent control

Symbol	Synonym(s)
ADCY	3',5'-cyclic AMP synthetase, 4.6.1.1, AC, Adenylate Cyclase, Adenyl Cyclase, Adenylyl cyclase, ATP diphosphate-lyase (cyclizing), mAC, sAC
ADRB1	Adrb, ADRB1R, ADR-beta1, Adrenergic Receptor Beta 1, adrenergic receptor, beta 1, Adrenergic Receptor β 1, adrenergic receptor, β 1, adrenoceptor beta 1, adrenoceptor β 1, ADR-β1, B1AR, beta-1 adrenergic receptor, beta1-ADRENORECEPTOR, BETA1AR, beta2-AR, beta-AR, FNSS2, RATB1AR, RHR, β 1-adrenergic receptor, β1-AR, β2-AR, β-AR
AKT	AKT1/2/3, B/Akt, PKB, RAC-PK
ATP	[[(2R,3S,4R,5R)-5-(6-aminopurin-9-yl)-3,4-dihydroxyoxolan-2-yl]methoxy-hydroxyphosphoryl] phosphono hydrogen phosphate, 56-65-5, 9-beta-D-arabinofuranosyladenine 5'-triphosphate, 9-β-D-arabinofuranosyladenine 5'-triphosphate, adenosine 5'-(tetrahydrogen triphosphate), adenosine 5'-triphosphate, ATP, ATP4-, C10H16N5O13P3
BGLAP	AI461847, Bgl, Bgla, Bglap1, Bglap2, Bglap3, Bglap-rs1, BGP, BGP2, Bgpr, Bgpra, bone gamma-carboxyglutamate protein, bone gamma-carboxyglutamate protein 2, bone gamma-carboxyglutamate protein 3, Bone Gla-protein, bone γ-carboxyglutamate protein, bone γ-carboxyglutamate protein 2, bone γ-carboxyglutamate protein 3, mOC-, mOC-A, mOC-B, mOC-X, O, OC, OCN, OC-X, OG, OG1, Og2, ORG, oste, Osteocalcin, Osteocalcin2
Ca2+	14127-61-8, Ca+2, calcium, calcium(2+), calcium cation, calcium citrate, calcium ion, calcium, ion (Ca2+), calcium ions, Citracal, tricalcium dicitrate
cAMP	11002-78-1, 33116-15-3, 3',5'-cyclic AMP, 3',5'-monophosphate, adenosine cyclic, 37839-81-9, (4aR,6R,7R,7aS)-6-(6-aminopurin-9-yl)-2-hydroxy-2-oxo-4a,6,7,7a-tetrahydro-4H-furo[3,2-d][1,3,2]dioxaphosphinin-7-ol, 54532-48-8, 55576-98-2, 60-92-4, 66067-13-8, 68407-13-6, adenosine 3',5'-phosphate, adenosine, cyclic 3',5'-(hydrogen phosphate), adenosine cyclic 3,5 monophosphate, adenosine cyclic 3',5'-monophosphate, adenosine cyclic monophosphate, C10H12N5O6P, cAMP, cyclic-3',5'-monophosphate, adenosine, cyclic adenosine monophosphate, cyclic adenylic acid, cyclic AMP, disodium salt, cyclic AMP, monoammonium salt, cyclic AMP, monopotassium salt, cyclic AMP, monosodium salt, cyclic AMP, sodium salt
CAV1	BSCL3, Cav, cave, Cavelolin 1, CAVEOLIN, Caveolin1, caveolin 1, caveolae protein, CGL3, LCCNS, LOC100362870, MSTP085, PPH3, VIP21
CCN3	C130088N23Rik, CCN, cellular communication network factor 3, IBP-9, IGFBP-9, IGFBP-RP3, NOV, NOVH
cGMP	3',5'-cyclic GMP, 7665-99-8, 9-[(4aR,6R,7R,7aS)-2,7-dihydroxy-2-oxo-4a,6,7,7a-tetrahydro-4H-furo[3,2-d][1,3,2]dioxaphosphinin-6-yl]-2-amino-1H-purin-6-one, C10H12N5O7P, cGMP, guanosine-3',5'-cyclic monophosphate, guanosine 3',5'-cyclic phosphate, guanosine cyclic 3',5'-(hydrogen phosphate)
CK1	Casein Kinase I, CKI
CTNNB1	armadillo, Beta-cat, beta CATENIN, Bfc, Cat, CATENIN beta, catenin beta 1, catenin (cadherin associated protein), beta 1, catenin (cadherin associated protein), β 1, CATENIN β, catenin β 1, CATNB, CTNB1, CTNNB, CTNN beta, CTNN β, EVR7, Mesc, MRD19, NEDSDV, β-cat, β-catenin
DAG	DAG, diacylglycerides, diglyceride
DBN1	D0S117E, Drebrin, drebrin 1, Drebrin E, DREBRIN E2
Dopamine	1,2-benzenediol, 4-(2-aminoethyl)-, 1,2-benzenediol, 4-(2-aminoethyl)- (9CI), 4-(2-aminoethyl)benzene-1,2-diol, 50444-17-2, 51-61-6, 62-31-7, C8H11NO2, DA, dopamine HCl, dopamine hydrochloride, hydroxytyramine, Intropin, Revimine
DRD1	C030036C15Rik, D1, D1a, D1DR, D1R, D1 receptor, D1 receptors, Da-d1 receptor, DADR, Dopamine d1 receptor, dopamine receptor D1, DR1, Drd-, DRD1A, Gpcr, Gpcr15
DRD2	D2, D2a dopamine receptor, D2 DOPAMINE receptor, D2 dopaminergic receptor, D2DR, D2-like receptors, D2R, dopamine D2, Dopamine D2L receptor, dopamine D2 receptor, dopamine receptor D2, Drd-
EGF	AI790464, EGF-1, epidermal growth factor, HOMG4, URG
EGFR	9030024J15RIK, AI552599, C-ERBB, EGFR1, EGF receptor, EGFR vIII, EGF-TK, epidermal growth factor receptor, Erb, ERBB, ERBB1, Err, Errb1, ERRP, HER1, HER1 (EGFR), MENA, NISBD2, PIG61, Wa, wa-2, Wa5
ERK1/2	MAPK p44/42, MAPK p44/p42, p42/44 mapk, P42/p44 erk, P42/p44 mapk, p42/p44 MAP KINASE
F Actin	Filamentous Actin
G proteinalpha I	Galphai, Gi, GI alpha, GI α, GNAI, Gn alpha, Gn α, G protein ai, G protein alpha I SUBUNITS, G protein α I, G protein α I SUBUNITS, Gαi
Glutamate	142-47-2, 19473-49-5, (2S)-2-aminopentanedioic acid, 56-86-0, C5H9NO4, Glu, glutamate, glutamic acid, glutaminol, L-Glu, L-glutamate, L-glutamic acid, monosodium glutamate, MPG, potassium glutamate, potassium L-glutamate, S-glutamate, sodium glutamate
Glutamatereceptor	GluR
GNAQ	1110005L02Rik, 6230401I02Rik, AA408290, AW060788, CMC1, DKFZp686D0521, Dsk, Dsk1, Dsk10, Gal, G-ALPHA-q, GAQ, G protein alpha Q, G protein alpha Q/11, G protein subunit alpha q, G protein subunit α q, G protein α Q, G protein α Q/11, Gq, Gqalpha, GqI, Gq protein alpha subunit, Gq protein α subunit, Gqα, guanine nucleotide binding protein, alpha q polypeptide, guanine nucleotide binding protein, α q polypeptide, G-α-q, Pst receptor, SWS
GNAS	5530400H20RIK, A930027G11RIK, AHO, AHO2, ALEX, C130027O20Rik, C20orf45, G, Ga, G-alpha-8, G alpha S, GANGLIOSIDE EXPRESSION FACTOR 2, Gn, GNAS1, GNAS complex locus, GNAS (guanine nucleotide binding protein, alpha stimulating) complex locus, GNAS (guanine nucleotide binding protein, α stimulating) complex locus, Gnpas, G protein α s, GPSA, Gs-, GSA, Gs-alpha, Gs alpha subunit, Gs GTP-Binding, GSP, GS α, Gs α subunit, Guanine nucleotide binding protein, alpha stimulating, Guanine nucleotide binding protein, α stimulating, G-α-8, G α S, LOC100361691, LOC690994, N, Nes, NESP, Nesp55, NESPL, Oed, OEDSML, P, P1, P2, P3, PHP1A, PHP1B, PITA3, POH, RGD:621483, SCG, SCG6, SgVI, XL, XLalphas
GRB2	AA408164, ASH, Ash-psi, EGFRBP-GRB2, GRAB2, GRBS, growth factor receptor bound protein 2, MST084, MSTP084, NCKAP2
GTP	[[(2R,3S,4R,5R)-5-(2-amino-6-oxo-1H-purin-9-yl)-3,4-dihydroxyoxolan-2-yl]methoxy-hydroxyphosphoryl] phosphono hydrogen phosphate, 86-01-1, C10H16N5O14P3, GTP, guanosine 5'-(tetrahydrogen triphosphate), Mg-GTP
Guanylatecyclase	4.6.1.2, GC, GC activity, GTP diphosphate-lyase (cyclizing), Guanylate cyclase, guanyl cyclase, Guanylyl Cyclase
HTR2	5-HT2, 5-Ht2 Receptor, 5-HTR2
Insp3r	Inositol 1,4,5-triphosphate receptor, Inositol Triphosphate Receptor, INSP3R, Ip3r, IP3 receptor, IP3-Sensitive Calcium Channel
IP3	27121-73-9, inositol trisphosphate, IP3, myo-inositol, tris(dihydrogen phosphate)
LPA	LPA, lysophosphatidic acids, lysophosphatidyl acid
LPAR1	AI326300, clone 4.9, EDG2, ENDOTHELIAL DIFFERENTIATION LYSOPHOSPHATIDIC ACID G-protein-COUPLED receptor 2, Gpcr, Gpcr26, Kdt2, L, LPA1, LPA1 receptor, LPA2, LPA receptor 1, LYSOPHOSPHATIDIC ACID G-protein-COUPLED receptor, lysophosphatidic acid receptor 1, Mrec1.3, rec.1.3, vzg-, VZG1
MAP2K1/2	MEK1/2, MKK1/2
MAP2K5	AI324775, AI428457, HsT17454, MAP kinase kinase 5, MAPKK5, MEK5, mitogen-activated protein kinase kinase 5, MKK5, PRKMK5
MAP3K2	9630061B06RIK, AI585793, LOC100506904, M3K2, MEKK2, MEKK2B, mitogen-activated protein kinase kinase kinase 2
MAPK7	b2b2346C, b2b2346Clo, BMK-1, ERK, ERK4, ERK-5, Erk5-T, ERK7, FRK, LOC100912585, mitogen-activated protein kinase 7, mitogen-activated protein kinase 7-like, PRKM7
NO	10102-43-9, Amidogen, oxo-, EDRF, gaseous nitric oxide, Genosyl, inhaled nitric oxide, INOmax, Mononitrogen monoxide, nitric oxide, nitric oxide gas, nitric oxide gas radical, Nitric oxide trimer, Nitrogen monooxide, nitrogen monoxide, nitrogen oxide (NO), nitrogen protoxide, Nitrosyl radical, NMO, NO
Norepinephrine	108341-18-0, 1,2-benzenediol, 4-(2-amino-1-hydroxyethyl)-, (R)- (9CI), 1,2-benzenediol, 4-((R)-2-amino-1-hydroxyethyl)-, [3H]-norepinephrine, 4-[(1R)-2-amino-1-hydroxyethyl]benzene-1,2-diol, 51-41-2, benzyl alcohol, alpha-(aminomethyl)-3,4-dihydroxy-, (-)-, benzyl alcohol, α-(aminomethyl)-3,4-dihydroxy-, (-)-, C8H11NO3, D-(-)-noradrenaline, Levophed, Levophed Bitartrate, L-noradrenaline, L-norepinephrine, NE, NE-hydrochloride, noradrenalin, noradrenaline, (-)-noradrenaline, (-)-norepinephrine, norepinephrine bitartrate, (R)-noradrenaline, (R)-norepinephrine, (R)-(-)-norepinephrine
PI3K	1-phosphatidylinositol 3-kinase, 2.7.1.137, ATP:1-phosphatidyl-1D-myo-inositol 3-phosphotransferase, Phosphatidylinositol 3 kinase, phosphatidylinositol 3'-kinase, PI3-kinase, PtdIns 3 Kinase, type III phosphoinositide 3-kinase, type I phosphatidylinositol kinase, Vps34p
PIP2	1,2-diacyl-sn-glycero-3-phospho-(1'-myo-inositol-4',5'-bisphosphate), 1-O-(3-sn-phosphatidyl)-1D-myo-inositol 4,5-bis(dihydrogen phosphate), 1-phosphatidyl-1D-myo-inositol 4,5-bisphosphate, C11H19O19P3R2
PKA	A-Kinase, cAMP-Dependent Protein Kinase, cyclic AMP depended protein kinase, protein KINASE A
PKC	Cnpkc, PKC, Pkc(s), Protein Kinase C
PKG	cgk, protein KINASE G
PLC	3.1.4.3, alpha-toxin, Clostridium oedematiens beta- and g-toxins, Clostridium oedematiens β- and g-toxins, Clostridium welchii alpha-toxin, Clostridium welchii α-toxin, heat-labile haemolysin, heat-labile hemolysin, lecithinase C, lipophosphodiesterase C, lipophosphodiesterase I, phosphatidase C, phosphatidylcholine cholinephosphohydrolase, PHOSPHOINOSITIDE SPECIFIC PHOSPHOLIPASE C, Phospholipase C, Pi-plc, α-toxin
RAF1	6430402F14Rik, AA990557, BB129353, CMD1NN, c-R, Cra, CRAF, Craf1, D830050J10Rik, leukaemiA ONCOGENE HOMOLOG1, LEUKEMIA ONCOGENE HOMOLOG1, NS5, Raf-1 proto-oncogene, serine/threonine kinase, v-, v-Raf, v-raf-leukaemia viral oncogene 1, v-raf-leukemia viral oncogene 1
Serotonin	3-(2-aminoethyl)-1H-indol-5-ol, 3-(2-aminoethyl)indol-5-ol, 50-67-9, 5-HT, C10H12N2O, indol-5-ol, 3-(2-aminoethyl)-, serotonin
SGSM3	1810012I01RIK, AI428557, BB175482, bdif-1, CIP, CIP85, MAP, R75178, RABGAP5, RABGAPLP, RUSC3, Rutbc, RUTBC3, small G protein signaling modulator 3
SP1	1110003E12RIK, AA450830, AI845540, Sp1-1, Sp1 transcription factor, Sp1 (trans spliced isoform), Trans-acting transcription factor 1
SP3	D130027J01RIK, Sp3 transcription factor, SPR2, trans-acting transcription factor 3
SRC	ASV, AW259666, BS27, c-SRC, p60-Src, PP60, pp60c, Pp60/c-Src, pp60c-src, Rous sarcoma oncogene, SRC1, SRC proto-oncogene, non-receptor tyrosine kinase, THC6, TVHUSC
TUBULIN	microtubule, tubulin complex

Pathway Analysis Using IPA Software; canonical pathway

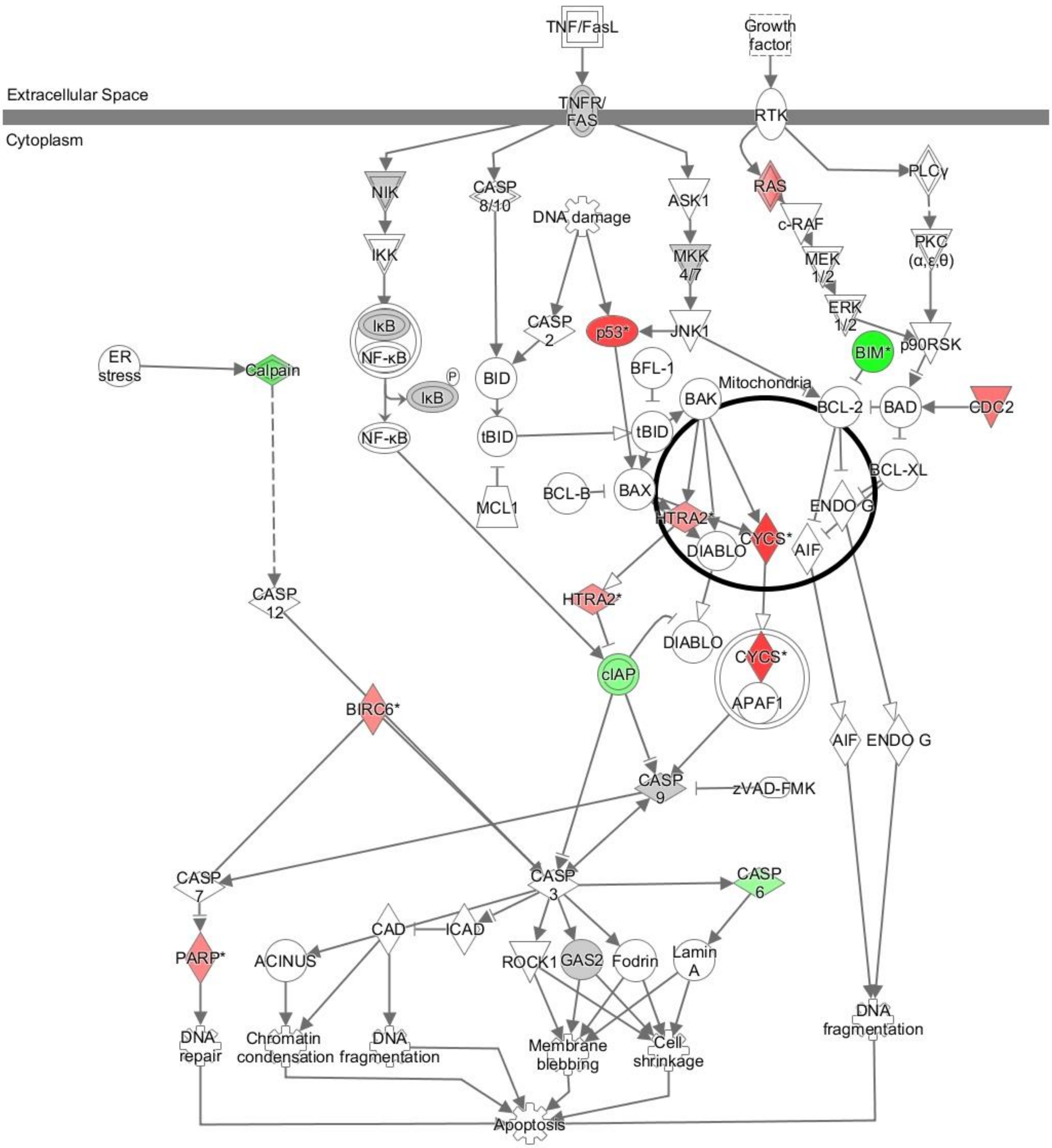

Figure S47. Apoptosis Signaling at 24 h

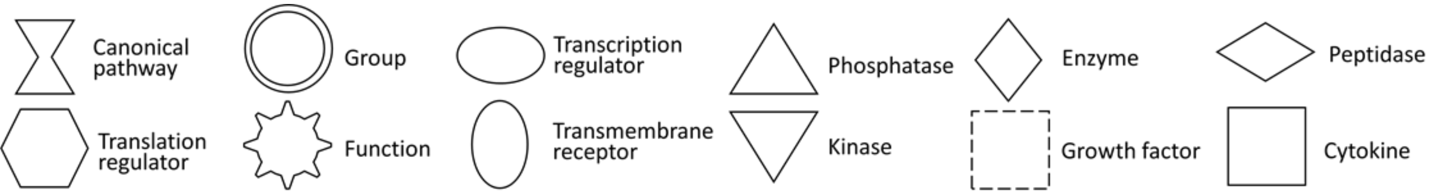

Red: Increased, FDR<0.05 versus solvent control

Green: Decreased, FDR<0.05 versus solvent control

Symbol	Synonym(s)
ACIN1	2610036I19Rik, 2610510L13Rik, Ac, ACINUS, ACINUS-1, acinusL, acinusS, ACN, apoptotic chromatin condensation inducer 1, Apoptotic Chromatin Condensation Inducer In The Nucleus, C79325, fSAP152, mKIAA0670
AIFM1	A, AIF, apoptosis inducing factor mitochondria associated 1, apoptosis inducing factor, mitochondria associated 1, apoptosis-inducing factor, mitochondrion-associated 1, AUNX1, CMT2D, CMTX4, COWCK, COXPD6, DFNX5, Hq, NADMR, NAMSD, Pdcd, PDCD8, SEMDHL
APAF1	6230400I06RIK, Ap, Apaf1l, apoptotic peptidase activating factor 1, CED4, fog, mKIAA0413
Apaf1-Cycs	Apaf1-CytoC, Cyt C-APAF1, CytochromeC-APAF1
BAD	AI325008, Bad v1, Bad v2, BBC2, BCL2-associated agonist of cell death, BCL2L8
BAK1	Ba, BAK, BAK-LIKE, BCL2-antagonist/killer 1, BCL2L7, CDN1, N-B, N-BAK1
BAX	Bcl2-associated X, BCL2 associated X, apoptosis regulator, BCL2-associated X protein, BCL2L4
BCL-XL	bBclxl, Bcl, BCL2L, BCL2-like 1, BCLX, Bcl-X beta, Bclx gamma, BCL-XL/S, Bcl-X β, Bclx γ, PPP1R52
BCL2	AW986256, B cell leukaemia/lymphoma 2, B cell leukemia/lymphoma 2, Bcl-, Bcl2 alpha, BCL2 apoptosis regulator, BCL2, apoptosis regulator, Bcl2 α, C430015F12Rik, D630044D05RIK, D830018M01RIK, LOC100046608, ORF16, PPP1R50
BCL2A1	A, A1-, A1-b, A1-d, ACC-1, ACC-2, BB218357, B cell leukaemia/lymphoma 2 related protein A1a, B cell leukaemia/lymphoma 2 related protein A1b, B cell leukaemia/lymphoma 2 related protein A1d, B cell leukemia/lymphoma 2 related protein A1a, B cell leukemia/lymphoma 2 related protein A1b, B cell leukemia/lymphoma 2 related protein A1d, Bcl2, BCL2A1A, Bcl2a1b, BCL2A1D, BCL2L5, BCL2-related protein A1, Bfl-, Bfl-1, BFL1/A1, GRS, Hbp, HBPA1, U23778, U23781
BCL2L10	AA420380, AU023065, B, BCL2 like 10, BCL-B, Boo, C85687, D, Diva
BCL2L11	1500006F24RIK, BAM, BCL2 like 11, BCL2-like 11 (apoptosis facilitator), Bi, BIM, Bo, BOD, BODL, LOC150819
BID	2700049M22Rik, AI875481, AU022477, BH3 interacting domain death agonist, cBid, FP497
BIRC6	A430032G04Rik, A430040A19Rik, AA501170, APOLLON, Baculoviral IAP repeat-containing 6, Bruc, BRUCE, D630005A10Rik, mKIAA1289, Ubiquitin-conjugating enzyme e2
c-lap	IAP, NAIP
Calpain	CALCIUM DEPENDENT PROTEASE, M calpain
CASP12	CASP12P1, CASPASE12, caspase 12 (gene/pseudogene)
CASP2	Casp, caspase 2, Ich-, ICH-1, Nedd, NEDD-2, PPP1R57
CASP3	A830040C14Rik, AC-, AC-3, Casp, Caspase-3, CASPASE-3 p20, CC3, CPP, CPP-32, CPP32B, CPP32-beta, CPP32-β, Ice-like cysteine protease, Lice, mld, mldy, SCA-1, Ya, YAMA
CASP6	caspase 6, LOC103689977, mCAS, MCH2
CASP7	AI314680, casp, Caspase-7, CMH-1, ICE-, ICE-IAP3, ICE-LAP3, LICE2, Lice2 cysteine protease, mCASP-7, MCH3
CASP9	AI115399, APAF-3, AW493809, Casp, Casp9 v1, Caspase-9, ICE-, ICE-LAP6, MCH6, PPP1R56
Caspase8/10	Casp8/10, Caspase 8,10
CDK1	CDC2, CDC28A, Cdc2a, CDC2 kinase, cyclin-dependent kinase 1, GROWTH-ASSOCIATED HISTONE H1 KINASE, p34, P34CDC2
CYCS	CYC, CYCSA, CYTC, CYTOC, CYTOCHROME C, cytochrome c, somatic, cytochrome c, somatic-like, ENSMUSG00000058927, HCS, LOC100363502, THC4, X laevis XLCL2
DFFA	A330085O09Rik, DFF1, DFF35, DFF-45, DNA fragmentation factor, alpha subunit, DNA fragmentation factor subunit alpha, DNA fragmentation factor subunit α, DNA fragmentation factor, α subunit, ICA, ICAD, ICAD-S
DFFB	5730477D02Rik, C, CA, CAD, caspase-activated DNase, CPAN, DFF2, DFF-40, Didf, Didff, DNA fragmentation factor, beta subunit, DNA fragmentation factor subunit beta, DNA fragmentation factor subunit β, DNA fragmentation factor, β subunit
DIABLO	0610041G12Rik, 1700006L01RIK, AU040403, DFNA64, diablo IAP-binding mitochondrial protein, diablo, IAP-binding mitochondrial protein, Sm, SMAC
ENDOG	ENDONUCLEASE G
ERK1/2	MAPK p44/42, MAPK p44/p42, p42/44 mapk, P42/p44 erk, P42/p44 mapk, p42/p44 MAP KINASE
GAS2	Gas, growth arrest-specific 2, RGD1563167
HTRA2	AI481710, Htr, HtrA serine peptidase 2, MGCA8, mnd, mnd2, O, OMI, PARK13, Pr, PRSS25
Ikb	I KAPPA B, Ikbeta, Ikβ, Iκ-B
IkB-NfkB	IkappaB-NFkappaB, IκB-NFκB, NFkB-IkB
IKK	I Kappa B Kinase, IKKALPHABETA, IKK Complex, I κ B Kinase
LMNA	CDCD1, CDDC, CMD1A, CMT2B1, Dhe, EMD2, FPL, FPLD, FPLD2, HGPS, IDC, lamin A, LAMIN A/C, LAMININ A/C, LDP1, LFP, LGMD1B, LMN1, LMNC, LMNL1, MADA, Prelamin-A/C, PRO1
MAP2K1/2	MEK1/2, MKK1/2
MAP2K4/7	Jnkk, MEK 4/7, MKK 4/7
MAP3K5	7420452D20Rik, A, APOPTOSIS SIGNAL REGULATED KINASE 1, AS, ASK, ASK1, M3K5, MAPKKK5, MEKK5, mitogen-activated protein kinase kinase kinase 5, RGD1306565
MAPK8	AI849689, C-JUN N-TERMINAL KINASE1, JNK, JNK1, JNK1A2, JNK21B1/2, JNK-46, mitogen-activated protein kinase 8, p46JNK1, p46JNK1 alpha, p46JNK1 α, Prk, PRKM8, SAPK1, SAPK1c, Sapk gamma, SAPK P46, Sapk γ, STRESS-ACTIVATED protein KINASE-LIKE KINASE
MCL1	AW556805, BCL2L3, EAT, Mcl-, MCL1 apoptosis regulator, BCL2 family member, mcl1/EAT, myeloid cell leukaemia sequence 1, myeloid cell leukemia sequence 1, TM
NFkB	NF-KAPPA B, NF-κ B, nuclear factor-κ b, transcription factor nuclear factor κ b
Parp1	5830444G22Rik, A, Adp, Adprp, Adprp1, ADPRT, ADPRT 1, AI893648, ARTD1, C80510, msPARP, pa, pADPRT-1, PARP, PARS, POLY(ADP-RIBOSE) POLYMERASE 1, poly (ADP-ribose) polymerase family, member 1, PPOL, sP, sPARP-1
PKC(α,ε,θ)	PKC (alpha, epsilon, theta), PKC (α,ε,θ)
PLC-gamma	Phospholipase C gamma, Phospholipase C γ, PLCG, PLCγ
RAF1	6430402F14Rik, AA990557, BB129353, CMD1NN, c-R, Cra, CRAF, Craf1, D830050J10Rik, leukaemiA ONCOGENE HOMOLOG1, LEUKEMIA ONCOGENE HOMOLOG1, NS5, Raf-1 proto-oncogene, serine/threonine kinase, v-, v-Raf, v-raf-leukaemia viral oncogene 1, v-raf-leukemia viral oncogene 1
ROCK1	1110055K06Rik, LOC100129157, P160ROCK, p160 ROCK-1, Rho-associated coiled-coil containing protein kinase 1, Roc, ROCK, ROCK-I, ROK, ROK beta, ROK β
RPS6KA1	HU-1, MAPKAPK1, MAPKAPK1A, MAPKAP kinase 1, MAPKAP Kinase 1 Alpha, MAPKAP Kinase 1 α, p90Rsk, p90-RSK 1, p90S6K, RIBOSOMAL protein S6 KINASE A, ribosomal protein S6 kinase A1, ribosomal protein S6 kinase polypeptide 1, Rs, RSK, RSK1, S6K-alpha-1, S6K-α-1
SPTAN1	2610027H02Rik, A2a, Alpha fodrin, (alpha)II-SPECTRIN, AlphaII spectrin, Alpha-spectorin, Alpha spectrin, alpha SPECTRIN 2, DEE5, EIEE5, Fodrin, IPF, NEAS, S, Sp, Spectrin alpha 2, spectrin alpha, non-erythrocytic 1, spectrin, alpha, non-erythrocytic 1, Spectrin α 2, spectrin α, non-erythrocytic 1, spectrin, α, non-erythrocytic 1, SPNA2, SPTA2, α fodrin, (α)II-SPECTRIN, α-spectorin, α spectrin, α SPECTRIN 2
TP53	bbl, BCC7, bfy, bhy, BMFS5, LFS1, p4, p44, p5, P53, P53 cellular tumour antigen, p53 tumor suppressor, transformation related protein 53, TRP53, tumor protein p53, tumour protein p53
zVAD-FMK	benzyloxycarbonyl-VAD-fluoromethyl ketone, N-benzyloxycarbonyl-Val-Ala-Asp-fluoromethyl ketone, ZVAD, z-VAD.FMK

Pathway Analysis Using IPA Software; canonical pathway

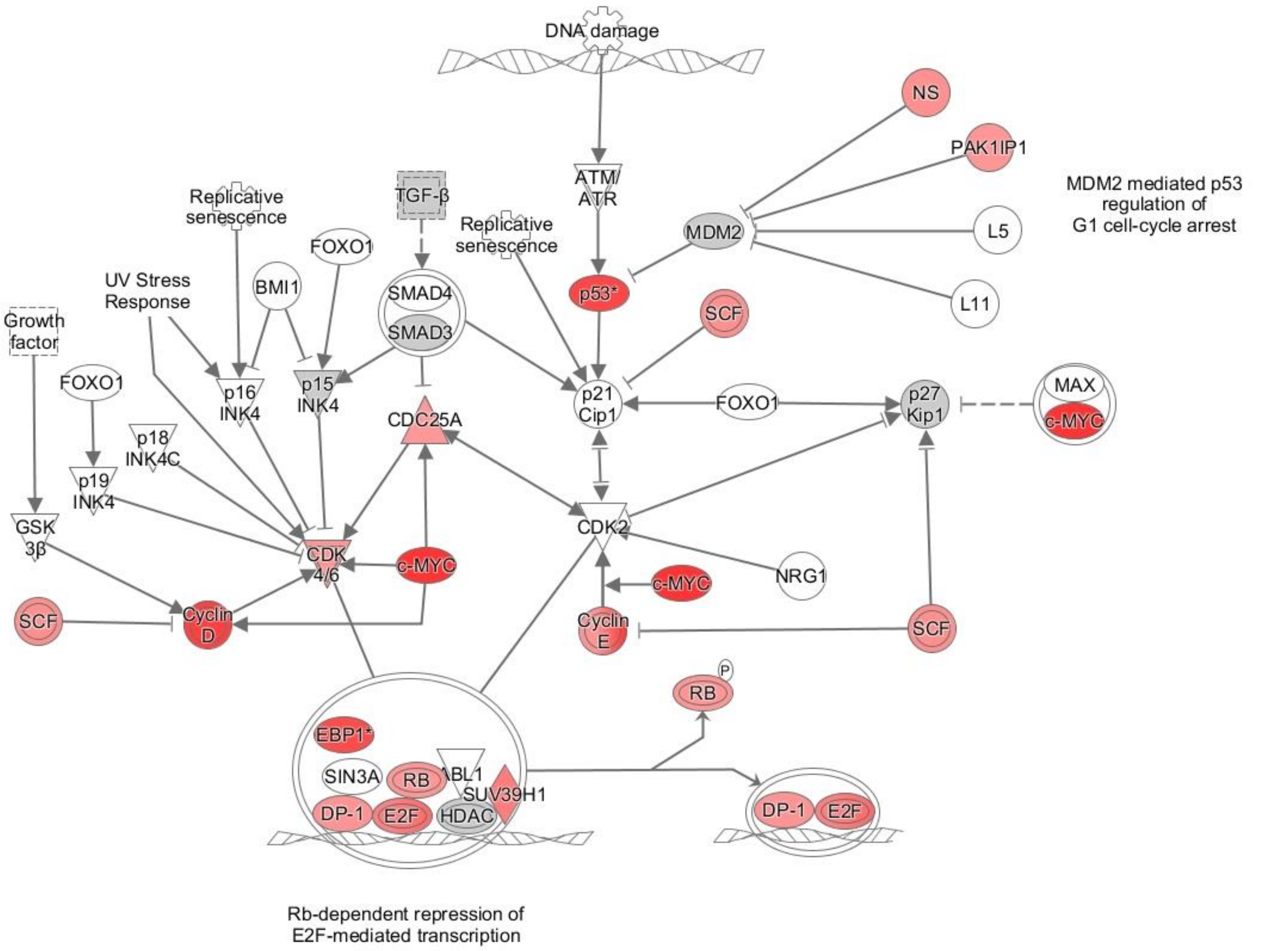

Figure S48. Cell Cycle G1/S Checkpoint Regulation at 24 h

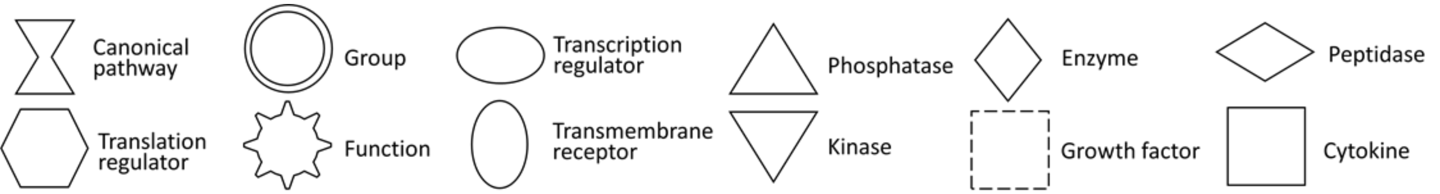

Red: Increased, FDR<0.05 versus solvent control

Green: Decreased, FDR<0.05 versus solvent control

Symbol	Synonym(s)
ABL1	ABL, ABL proto-oncogene 1, non-receptor tyrosine kinase, AI325092, BCR-ABL, c-A, c-ABL, CABL1, c-abl oncogene 1, non-receptor tyrosine kinase, CHDSKM, E430008G22Rik, JTK7, LOC100909750, p145Abl, p150, tyrosine-protein kinase ABL1-like, v-abl
ATM/ATR	ATR/ATM
BMI1	AW546694, Bmi-, BMI1 proto-oncogene, polycomb ring finger, FLVI2/BMI1, Pcgf, PCGF4, RNF51
CDC25A	CDC25A2, cell division cycle 25A, D9Ertd393, D9Ertd393e
CDK2	A630093N05Rik, CDC2-RELATED KINASE, CDKN2, Cyclin A associated kinase, cyclin-dependent kinase 2, CYCLIN E ASSOCIATED KINASE, p33(CDK2)
CDKN1A	CAP, CAP20, CDK, CDKI, Cdkn, CDKN1, CDKNA1, CI, CIP1, cyclin-dependent kinase inhibitor 1A, cyclin-dependent kinase inhibitor 1A (P21), mda, MDA-6, P2, P21, p21C, p21Cip, p21CIP1, p21W, p21WAF, p21Waf1, PzI Cyclin-Dependent Kinase Inhibitor, SD, SDI1, UV96, Waf, WAF1
CDKN1B	AA408329, AI843786, Cdki1b, CDKN4, cyclin-dependent kinase inhibitor 1B, CYCLIN-DEPENDENT KINASE INHIBITOR P27, KIP1, MEN1B, MEN4, p2, p27, p27K, P27kip, P27KIP1, P28-ICK
CDKN2A	A, Arf, ARF-INK4a, CDK4I, CDKN2, CMM2, CYCLIN-DEPENDENT KINASE INHIBITOR 2A, INK4, INK4A, INK4a-ARF, Ink4a/Arf, MLM, MTS, MTS-1, p1, p14ARF/ p16INK4a, p16, p16/ARF, p16Cdkn2a, p16I, p16 INK4, p16/ INK4a, P19, p19ARF, Pct, PCTR1, TP16
CDKN2B	AV083695, CDK4I, cyclin-dependent kinase inhibitor 2B, INK4B, MTS, MTS2, p1, P15, p15IN, p15INK4, p15INK4b, p15(INK4b)
CDKN2C	C77269, CDKN6, cyclin-dependent kinase inhibitor 2C, INK, INK4C, p1, p18, p18IN, p18-INK4C, p18-INK6
CDKN2D	cyclin dependent kinase inhibitor 2D, INK, INK4D, p1, p19, p19IN, p19-INK4D
CyclinD	CycD, Cyclin D1
E2f-Tfdp1	E2F-DP1
FOXO1	Afx, Afxh, AI876417, FKH, FKH1, FKHR, FKHR1, Forkhead, forkhead box O1, Fox, FOXO1A
GNL3	C77032, E2IG3, G protein nucleolar 3, guanine nucleotide binding protein-like 3 (nucleolar), NNP47, NS, NUCLEOSTEMIN, NUG1
GSK3B	7330414F15Rik, 8430431H08Rik, C86142, glycogen synthase kinase 3 beta, glycogen synthase kinase 3 β, GSK-, GSK-3, GSK-3be, GSK-3beta, GSK-3β, GSKbeta, GSKβ, Tpk1
HDAC	Histone Deacetylase, Histone deacetyltransferase
MAX	AA960152, AI875693, bHLHd, bHLHd4, bHLHd5, bHLHd6, bHLHd7, bHLHd8, Max protein, MYC associated factor X, Myn
Max-Myc	cMyc-MAX, Myc-MAX
MDM2	1700007J15Rik, AA415488, ACTFS, hdm2, HDMX, LSKB, MDM2-A1, MDM2 proto-oncogene, MGC5370, Transformed 3t3 cell double minute 2, transformed mouse 3T3 cell double minute 2
MYC	AU016757, bHLHe3, bHLHe39, CMYC, C-MYC-P64, mMyc, MRTL, Myc2, MYCC, MYC proto-oncogene, bHLH transcription factor, myelocytomatosis oncogene, N, Niard, Nird, RNCMYC
NRG1	6030402G23RIK, ARIA, D230005F13Rik, GGF, GGFII, GP30, hereg, Heregulin, HG, HGL, HR, HRG, HRG1, HRGA, HRGalpha, Hrg α, MST131, MSTP131, NAF, ND, NDF, Ndf44, Neuregulin 1, Nrg1a 44 kda isoform, Nrg1 alpha 2c, NRG1B1, Nrg1 isoform 7, NRG1-IT2, NRG1 SECRETED, Nrg1 α 2c, Nrg alpha, Nrg alpha 2c, Nrg beta, Nrg α, Nrg α 2c, Nrg β, SMD, SMDF
PA2G4	AA672939, Ebp, EBP1, HG4-1, P, p38-2G4, Plfap, proliferation-associated 2G4, Proliveration-associated protein 1
PAK1IP1	5830431I15Rik, 5930415H02Rik, AA419825, AI314040, AW556169, bA421M1.5, Gdpd, Gdpd1, hPIP1, MAK11, P, PAK1 interacting protein 1, PIP1, Riken cDNA 5830431i15, WDR84
RB	pRb, Rb Tumor Suppressor, Rb tumour Suppressor
Rb-E2Ftranscriptionrepression	Rb1-E2f1, Rb-E2F, Rb-E2F1
RPL11	2010203J19RIK, DBA7, GIG34, L11, ribosomal protein L11, uL5
RPL5	L5, MSTP030, PPP1R135, ribosomal L5, Ribosomal protein l1a, ribosomal protein L5, U21, U21RNA, uL18
SCF	SCF complex
SIN3A	AW553200, mKIAA4126, mS, MSIN3A, S, SIN3, SIN3 transcription regulator family member A, transcriptional regulator, SIN3A (yeast), WITKOS
SMAD3	AU022421, DKFZP586N0721, hMAD-3, HSPC193, HsT17436, JV15-2, LDS1C, LDS3, MAD3, Madh, MADH3, SMAD family member 3
SMAD4	AW743858, D18Wsu70, D18Wsu70e, DPC, DPC4, JIP, Madh, MADH4, MYHRS, SMAD family member 4, Smaug1
SUV39H1	AI852103, AL022883, DXHXS7466, DXHXS7466e, H3-K9-HMTase 1, KMT1, KMT1A, MG44, mI, mIS6, RGD1565028, suppressor of variegation 3-9 1, suppressor of variegation 3-9 homolog 1, suppressor of variegation 3-9 homolog 1 (Drosophila)-like 1, SUV39H, Suv39h1l1
TFDP1	DILC, Dp, DP-1, Drtf, DRTF1, TB2/DP1, transcription factor Dp-1
Tgfbeta	Tgfb, TGF-beta 1, 2, and 3, TGF β, TGF-β 1, 2, and 3, transforming growth factor-β
TP53	bbl, BCC7, bfy, bhy, BMFS5, LFS1, p4, p44, p5, P53, P53 cellular tumour antigen, p53 tumor suppressor, transformation related protein 53, TRP53, tumor protein p53, tumour protein p53

Pathway Analysis Using IPA Software; canonical pathway

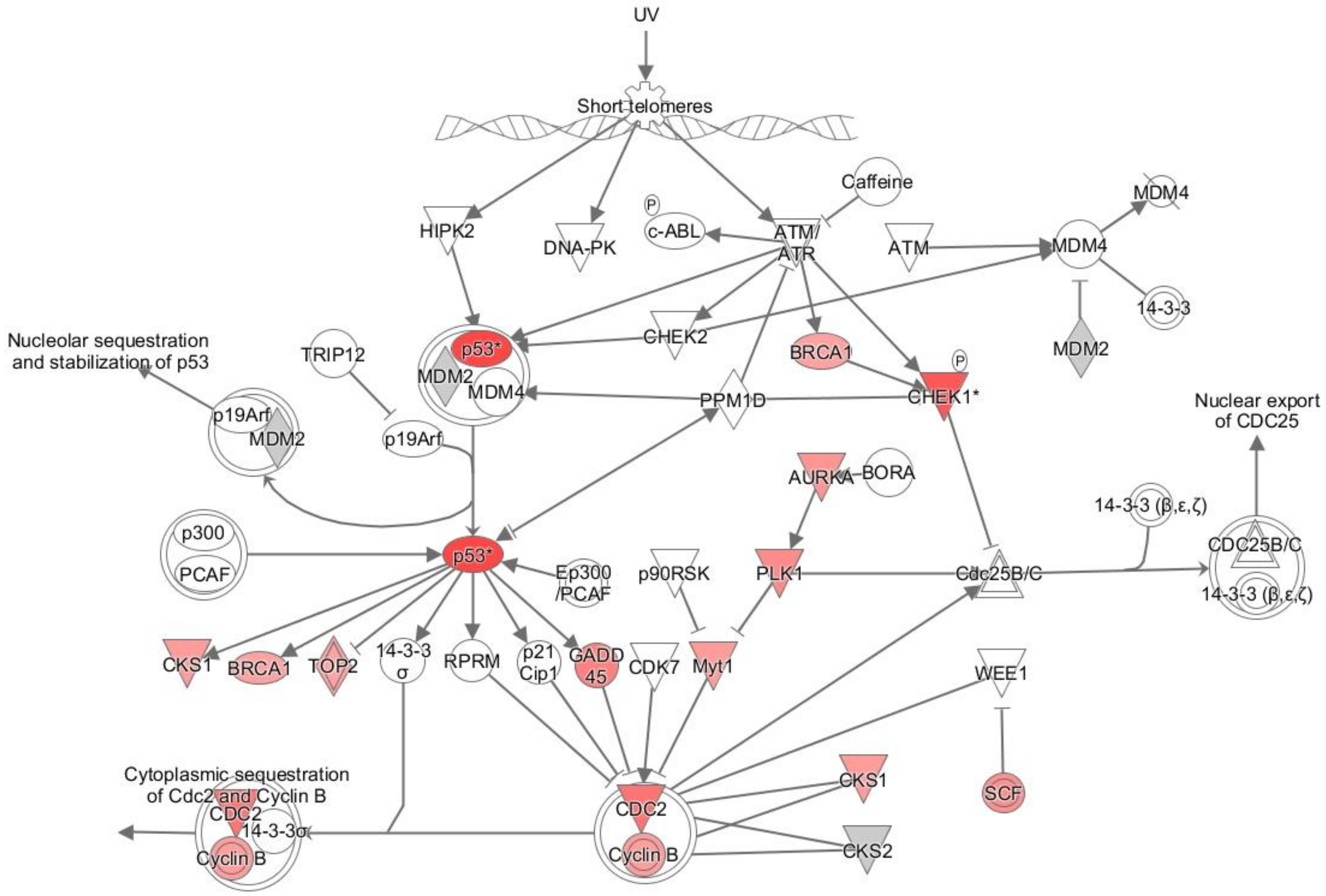

Figure S49. Cell Cycle G2/M DNA Damage Checkpoint Regulation at 24 h

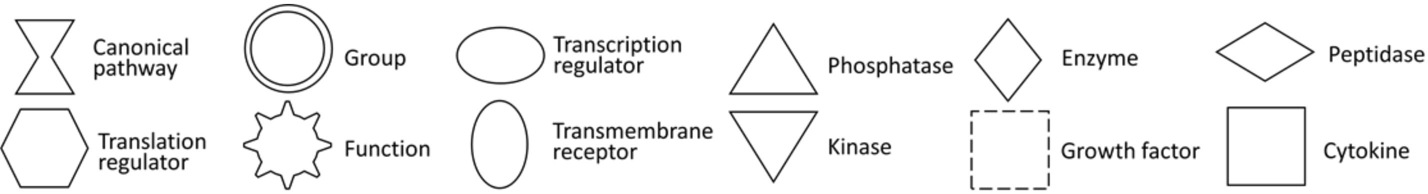

Red: Increased, FDR<0.05 versus solvent control

Green: Decreased, FDR<0.05 versus solvent control

Symbol	Synonym(s)
14-3-3	CBP
14-3-3(β,ε,ζ)	14-3-3 (beta, epsilon, zeta), 14-3-3 (β,ε,ζ)
ABL1	ABL, ABL proto-oncogene 1, non-receptor tyrosine kinase, AI325092, BCR-ABL, c-A, c-ABL, CABL1, c-abl oncogene 1, non-receptor tyrosine kinase, CHDSKM, E430008G22Rik, JTK7, LOC100909750, p145Abl, p150, tyrosine-protein kinase ABL1-like, v-abl
ATM	AI256621, AT1, ATA, ataxia telangiectasia mutated, ATC, ATD, ATDC, ATE, ATM serine/threonine kinase, C030026E19RIK, TEL1, TELO1
ATM/ATR	ATR/ATM
AURKA	AI, AIK, AIRK1, Ar, ARK-1, Au, AU019385, AURA, AURORA 2, AURORA A, AURORA KINASE, aurora kinase A, Aurora Related Kinase1, AW539821, Ayk, Ayk1, BTAK, I, IA, IAK, IAK1, PPP1R47, Stk, STK15, STK6, STK7
BORA	6720463M24Rik, AI317232, BORA aurora kinase A activator, bora, aurora kinase A activator, C13orf34, RGD1309522
BRCA1	BRCA1 DNA repair associated, BRCA1, DNA repair associated, BRCAI, BRCC1, breast cancer 1, early onset, BROVCA1, FANCS, PNCA4, PPP1R53, PSCP, RNF53
Caffeine	1,3,7-trimethylpurine-2,6-dione, 1H-purine-2,6-dione, 3,7-dihydro-1,3,7-trimethyl-, 58-08-2, 69-22-7, 8000-95-1, Alert-Pep, C8H10N4O2, Cafamil, Cafcit, Cafecon, Caffedrine, Caffedrine Caplets, Caffeine Anhydrous, caffeine citrate, Caffeine-Sodium Benzoate, Caffine, Cafipel, Coffein, Coffeine, Darvon Compound, Dasin, Dexitac, Dexitac Stay Alert Stimulant, Dhc Plus, Diurex, Durvitan, Eldiatric C, Enerjets, Ercatab, Gencebok, Guaranine, Hycomine, Invagesic, Invagesic Forte, Keep Alert, Kofein, Koffein, Lanorinal, Mateina, Maximum Strength Snapback Stimulant Powders, Medigesic Plus, methyltheobromine, Migergot, Miudol, Natural Caffeinum, Nix Nap, Nodaca, No-Doz, Nodoz Maximum Strength Caplets, Organex, Pep-Back, Peyona, Phensal, Propoxyphene Compound-65, Quick Pep, Refresh'n, SK 65 Compound, Stim, Thein, Theine, Ultra Pep-Back, Vivarin, Wake-Up, xanthine, 1,3,7-trimethyl
CDK1	CDC2, CDC28A, Cdc2a, CDC2 kinase, cyclin-dependent kinase 1, GROWTH-ASSOCIATED HISTONE H1 KINASE, p34, P34CDC2
CDK1-Cyclin B	Cdc2-CyclinB
CDK7	AI323415, AI528512, C230069N13, CAK, CAK1, Cdkn, CDKN7, Crk, Crk4, cyclin-dependent kinase 7, ENSMUSG00000074700, HCAK, MO15, p39MO15, STK1
CDKN1A	CAP, CAP20, CDK, CDKI, Cdkn, CDKN1, CDKNA1, CI, CIP1, cyclin-dependent kinase inhibitor 1A, cyclin-dependent kinase inhibitor 1A (P21), mda, MDA-6, P2, P21, p21C, p21Cip, p21CIP1, p21W, p21WAF, p21Waf1, PzI Cyclin-Dependent Kinase Inhibitor, SD, SDI1, UV96, Waf, WAF1
CHEK1	C85740, checkpoint kinase 1, CHK1, rad27
CHEK2	CDS1, Check2, checkpoint kinase 2, CHK2, hCds1, HUCDS1, LFS2, PP1425, Rad, RAD53
CKS1B	2410005G18Rik, 2610005D03Rik, AA407784, CDC28 protein kinase 1b, CDC28 protein kinase regulatory subunit 1B, CDC28 REGULATORY subunit 1B, Cks, CKS1, ckshs1, PNAS-16, PNAS-18, RGD1561797, sid1334, Sid1334p, Suc1
CKS2	1110038L14Rik, CDC28 protein KINASE REGULATORY subunit 2, CDC28 REGULATORY subunit 2, CKSH, CKSHS2, P13SUC1, RGD1562047
EP300	A430090G16, A730011L11, E1A binding protein p300, KAT3, KAT3B, MKHK2, p30, p300, p300 HAT, RSTS2
GADD45A	AA545191, Ddit, DDIT1, Gadd, GADD45, GADD45 alpha, GADD45α, growth arrest and DNA-damage-inducible 45 alpha, growth arrest and DNA-damage-inducible 45 α, Growth arrest and DNA-damage-inducible 45, α, growth arrest and DNA damage inducible alpha, growth arrest and DNA-damage-inducible, alpha, growth arrest and DNA damage inducible α, growth arrest and DNA-damage-inducible, α
HIPK2	1110014O20RIK, B230339E18RIK, homeodomain interacting protein kinase 2, LOC100505582, LOC653052, PRO0593, St, Stank
KAT2B	A930006P13RIK, AI461839, AW536563, CAF, K(lysine) acetyltransferase 2B, lysine acetyltransferase 2B, Pc, PCAF, P/CAF, Pcaf-b
MDM2	1700007J15Rik, AA415488, ACTFS, hdm2, HDMX, LSKB, MDM2-A1, MDM2 proto-oncogene, MGC5370, Transformed 3t3 cell double minute 2, transformed mouse 3T3 cell double minute 2
MDM4	4933417N07RIK, AA414968, AL023055, AU018793, AU021806, BMFS6, C85810, HDMX, LOC102633382, MDM4 regulator of p53, MDMX, MRP1, transformed mouse 3T3 cell double minute 4
p19 Arf	A, Arf, ARF-INK4a, CDK4I, CDKN2, CMM2, CYCLIN-DEPENDENT KINASE INHIBITOR 2A, INK4, INK4A, INK4a-ARF, Ink4a/Arf, MLM, MTS, MTS-1, p1, p14ARF/ p16INK4a, p16, p16/ARF, p16Cdkn2a, p16I, p16 INK4, p16/ INK4a, P19, p19ARF, Pct, PCTR1, TP16
PKMYT1	6230424P17, AW209059, MYT1, PPP1R126, protein kinase, membrane associated tyrosine/threonine 1, RGD1305434
PLK1	P, PLK, polo-like kinase 1, STPK, STPK13
PPM1D	AV338790, IDDGIP, JDVS, PP2C-DELTA, PP2C-δ, Ppm1d predicted, protein phosphatase 1D magnesium-dependent, delta isoform, protein phosphatase 1D magnesium-dependent, δ isoform, protein phosphatase, Mg2+/Mn2+ dependent 1D, protein phosphatase, Mg2+/Mn2+ dependent, 1D, PTP delta P1, PTP δ P1, Wi, WIP1
PRKDC	AI326420, AU019811, DNA-, DNA-DEPENDENT protein KINASE, DNAPDcs, DNAPK, DNA-PKC, DNA-PKcs, DNPK1, DOX, DOXNPH, dxn, dxnph, HYRC, HYRC1, IMD26, p350, p460, Prkdc predicted, protein kinase, DNA activated, catalytic polypeptide, protein kinase, DNA-activated, catalytic subunit, scid, slip, XRCC, XRCC7
RPRM	2410012A13Rik, Re, REPRIMO, reprimo, TP53 dependent G2 arrest mediator candidate, reprimo, TP53 dependent G2 arrest mediator homolog
RPS6KA1	HU-1, MAPKAPK1, MAPKAPK1A, MAPKAP kinase 1, MAPKAP Kinase 1 Alpha, MAPKAP Kinase 1 α, p90Rsk, p90-RSK 1, p90S6K, RIBOSOMAL protein S6 KINASE A, ribosomal protein S6 kinase A1, ribosomal protein S6 kinase polypeptide 1, Rs, RSK, RSK1, S6K-alpha-1, S6K-α-1
SCF	SCF complex
SFN	14-3-3, 14-3-3 Sigma, 14-3-3 σ, E, ER, HME1, Mme1, Stratifin, Ywh, YWHAS
TOP2	DNA Topoisomerase II, Topo II, Topoisomerase II
TP53	bbl, BCC7, bfy, bhy, BMFS5, LFS1, p4, p44, p5, P53, P53 cellular tumour antigen, p53 tumor suppressor, transformation related protein 53, TRP53, tumor protein p53, tumour protein p53
TRIP12	1110036I07RIK, 6720416K24RIK, AA410158, GtI, GTL6, KIAA0045, MRD49, thyroid hormone receptor interactor 12, TRIPC, ULF
WEE1	WEE1A, Wee1b, WEE1 G2 checkpoint kinase, WEE 1 homolog 1 (S. pombe), WEE1hu, WEE1-LIKE protein KINASE

Pathway Analysis Using IPA Software; canonical pathway

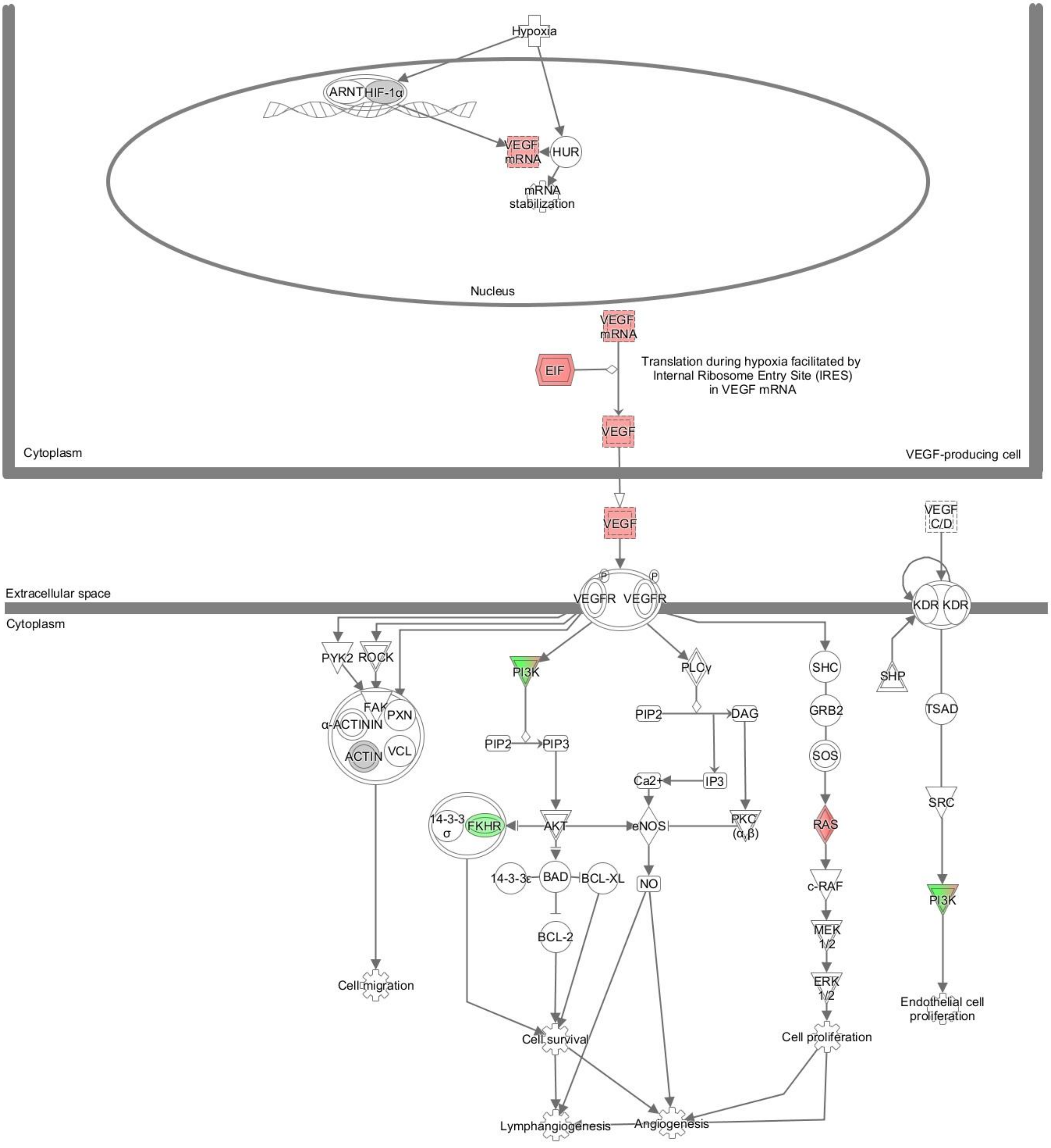

Figure S50. VEGF Signaling at 24 h

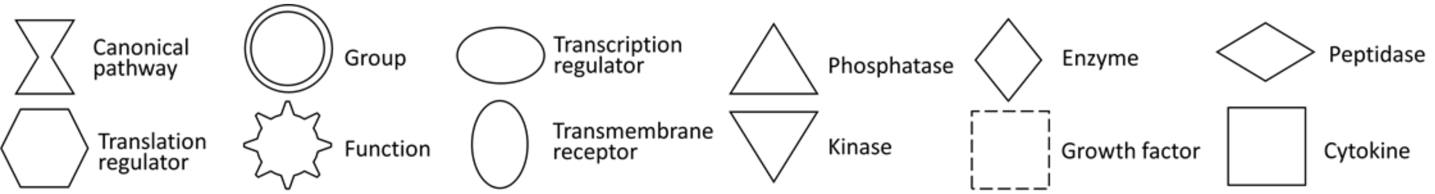

Red: Increased, FDR<0.05 versus solvent control

Green: Decreased, FDR<0.05 versus solvent control

Symbol	Synonym(s)
ACTIN	CLEC9A Ligand, G-actin
AKT	AKT1/2/3, B/Akt, PKB, RAC-PK
Alphaactinin	ACTININ, Actinin alpha, Actinin α, ACTN, α-Actinin, α Actinin human
ARNT	Arnt1, aryl hydrocarbon receptor nuclear translocator, bHLHe, bHLHe2, D3Ertd557, D3Ertd557e, DIOXIN receptor, Drnt, ESTM4, ESTM42, Hif1, HIF1B, HIF1BETA, HIF-1-β, HIF beta, HIF β, mKIAA4051, TANGO, W08714
BAD	AI325008, Bad v1, Bad v2, BBC2, BCL2-associated agonist of cell death, BCL2L8
BCL-XL	bBclxl, Bcl, BCL2L, BCL2-like 1, BCLX, Bcl-X beta, Bclx gamma, BCL-XL/S, Bcl-X β, Bclx γ, PPP1R52
BCL2	AW986256, B cell leukaemia/lymphoma 2, B cell leukemia/lymphoma 2, Bcl-, Bcl2 alpha, BCL2 apoptosis regulator, BCL2, apoptosis regulator, Bcl2 α, C430015F12Rik, D630044D05RIK, D830018M01RIK, LOC100046608, ORF16, PPP1R50
Ca2+	14127-61-8, Ca+2, calcium, calcium(2+), calcium cation, calcium citrate, calcium ion, calcium, ion (Ca2+), calcium ions, Citracal, tricalcium dicitrate
DAG	DAG, diacylglycerides, diglyceride
ELAVL1	2410055N02Rik, DKFZP667b083, ELAV1, ELAV (embryonic lethal, abnormal vision)-like 1 (Hu antigen R), ELAV like RNA binding protein 1, Hu, Hua, Hu antigen R, HUR, MelG, RGD:731215, RNA binding protein HuR, W91709
ERK1/2	MAPK p44/42, MAPK p44/p42, p42/44 mapk, P42/p44 erk, P42/p44 mapk, p42/p44 MAP KINASE
FKHR	FOXO1/3A
GRB2	AA408164, ASH, Ash-psi, EGFRBP-GRB2, GRAB2, GRBS, growth factor receptor bound protein 2, MST084, MSTP084, NCKAP2
HIF1A	AA959795, bHLHe7, bHLHe78, HIF-1, HIF1-ALPHA, HIF-1alpha (hydroxylated), HIF-1-α, HIF-1α (hydroxylated), Hypoxia inducible factor 1 alpha subunit, hypoxia inducible factor 1, alpha subunit, hypoxia inducible factor 1 subunit alpha, hypoxia inducible factor 1 subunit α, Hypoxia inducible factor 1 α subunit, hypoxia inducible factor 1, α subunit, MO, MOP1, PASD8
IP3	108340-81-4, 1,4,5-InsP3, [(1R,2S,3R,4R,5S,6R)-2,3,5-trihydroxy-4,6-diphosphonooxycyclohexyl] dihydrogen phosphate, 85166-31-0, 88269-39-0, C6H15O15P3, D-myo-Inositol, 1,4,5-tris(dihydrogen phosphate), D-myo-inositol (1,4,5)-trisphosphate, D-myo-inositol 1,4,5-trisphosphate, inositol 1,4,5-triphosphate, inositol 1,4,5-trisphosphate, Ins(1,4,5)P3, InsP3, IP3, myo-inositol 1,4,5-trisphosphate, phosphatidylinositol 1,4,5-triphosphate
KDR	6130401C07, CD309, Flk, FLK1, Kinase Insert Domain, kinase insert domain protein receptor, kinase insert domain receptor, Krd-1, Ly73, orv, VEGF, VEGFR, VEGFR-2
MAP2K1/2	MEK1/2, MKK1/2
NO	10102-43-9, Amidogen, oxo-, EDRF, gaseous nitric oxide, Genosyl, inhaled nitric oxide, INOmax, Mononitrogen monoxide, nitric oxide, nitric oxide gas, nitric oxide gas radical, Nitric oxide trimer, Nitrogen monooxide, nitrogen monoxide, nitrogen oxide (NO), nitrogen protoxide, Nitrosyl radical, NMO, NO
NOS3	2310065A03Rik, e, ec, ECNOS, eNOS, nitric oxide synthase 3, nitric oxide synthase 3, endothelial cell, nNOS, No
PI3K	1-phosphatidylinositol 3-kinase, 2.7.1.137, ATP:1-phosphatidyl-1D-myo-inositol 3-phosphotransferase, Phosphatidylinositol 3 kinase, phosphatidylinositol 3'-kinase, PI3-kinase, PtdIns 3 Kinase, type III phosphoinositide 3-kinase, type I phosphatidylinositol kinase, Vps34p
PIP2	1,2-diacyl-sn-glycero-3-phospho-(1'-myo-inositol-4',5'-bisphosphate), 1-O-(3-sn-phosphatidyl)-1D-myo-inositol 4,5-bis(dihydrogen phosphate), 1-phosphatidyl-1D-myo-inositol 4,5-bisphosphate, C11H19O19P3R2
PIP3	1-phosphatidyl-1D-myo-inositol 3,4,5-trisphosphate, phosphatidylinositol-3,4,5-trisphosphate, phosphoinositide (3,4,5) P3, PI(3,4,5)P3, PIns(3,4,5)P3, PIP3, PtdIns(3,4,5)P3
PKCalpha/beta	PKC (alpha, beta), PKCalpha/betaII, PKC alphaI/betaII, PKC (α,β), PKC α/β, PKCα/βII, PRKCA/B
PLC-gamma	Phospholipase C gamma, Phospholipase C γ, PLCG, PLCγ
PTK2	FA, Fad, FADK, FADK 1, FAK, FAK1, FAK related non-kinase, FR, p125FAK, pp125FAK, PPP1R71, protein tyrosine kinase 2, PTK2 protein tyrosine kinase 2, TYROSINE KINASE 2
PTK2B	CADTK, CAKB, CAKbe, CAK beta, CAK β, cell adhesion kinase β, E430023O05Rik, FADK2, FAK2, PKB, protein tyrosine kinase 2 beta, protein tyrosine kinase 2 β, PTK, PTK2 protein tyrosine kinase 2 beta, PTK2 protein tyrosine kinase 2 β, PYK, PYK2, Raf, RAFTK
PXN	AW108311, AW123232, FLJ23042, P, PAX, PAXILLIN
RAF1	6430402F14Rik, AA990557, BB129353, CMD1NN, c-R, Cra, CRAF, Craf1, D830050J10Rik, leukaemiA ONCOGENE HOMOLOG1, LEUKEMIA ONCOGENE HOMOLOG1, NS5, Raf-1 proto-oncogene, serine/threonine kinase, v-, v-Raf, v-raf-leukaemia viral oncogene 1, v-raf-leukemia viral oncogene 1
ROCK	RhoA-Binding Kinase alpha/beta, RhoA-Binding Kinase α/β, Rho Kinase, ROK, ROK alpha/beta, ROK α/β
SFN	14-3-3, 14-3-3 Sigma, 14-3-3 σ, E, ER, HME1, Mme1, Stratifin, Ywh, YWHAS
SH2D2A	F2771, L, LAD, R, Ribp, Rlk-binding, SCAP, SH2 domain containing 2A, TS, TSAD, VRAP
SHC1	p52SHC, p6, p66, p66s, P66shc, Sh, SHC, Shc (46 kDa isoform), SHCA, SHC adaptor protein 1, Shc p66 isoform, src homology 2 domain-containing transforming protein C1
SHP	Ptpn6/11, SHP-1/2
SRC	ASV, AW259666, BS27, c-SRC, p60-Src, PP60, pp60c, Pp60/c-Src, pp60c-src, Rous sarcoma oncogene, SRC1, SRC proto-oncogene, non-receptor tyrosine kinase, THC6, TVHUSC
VCL	9430097D22, AA571387, AI462105, AW545629, CMD1W, CMH15, HEL114, MV, MVCL, Vcl predicted, Vinculin
VEGFR	VEGFR
YWHAE	14-3-3E, 14-3-3 epsilon, 14-3-3 L, 14-3-3 ε, AU019196, HEL2, KCIP-1, LOC727845, MDCR, MDS, tyrosine 3-monooxygenase/tryptophan 5-monooxygenase activation protein epsilon, tyrosine 3-monooxygenase/tryptophan 5-monooxygenase activation protein, epsilon, tyrosine 3-monooxygenase/tryptophan 5-monooxygenase activation protein, epsilon polypeptide, tyrosine 3-monooxygenase/tryptophan 5-monooxygenase activation protein ε, tyrosine 3-monooxygenase/tryptophan 5-monooxygenase activation protein, ε, tyrosine 3-monooxygenase/tryptophan 5-monooxygenase activation protein, ε polypeptide

Pathway Analysis Using IPA Software; canonical pathway

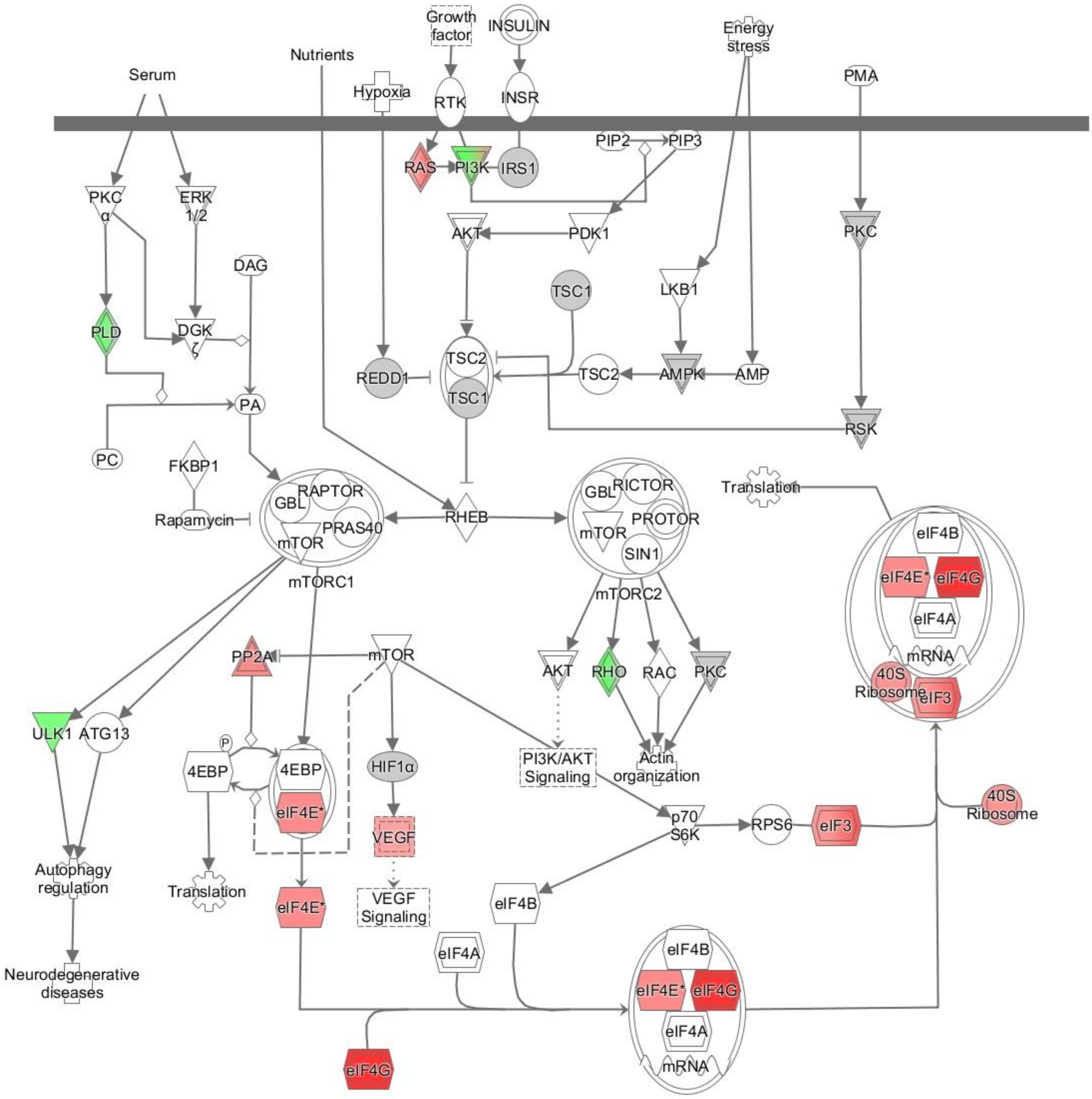

Figure S51. mTOR Signaling at 24 h

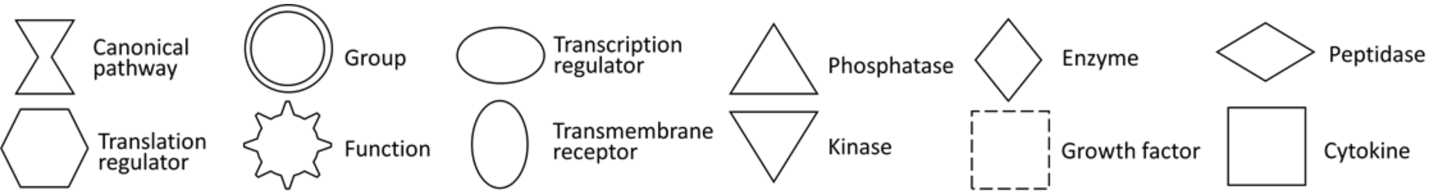

Red: Increased, FDR<0.05 versus solvent control

Green: Decreased, FDR<0.05 versus solvent control

Symbol	Synonym(s)
4EBP-eIF4E	eIF4E-eIF4EBP
AKT	AKT1/2/3, B/Akt, PKB, RAC-PK
AKT1S1	1110012J22RIK, AI227026, AI430011, AKT1 substrate 1, AKT1 substrate 1 (proline-rich), Lo, Lobe, Lobel, PR, PRAS, PRAS40, Proline-rich AKT substrate
AMP	149022-20-8, [(2R,3S,4R,5R)-5-(6-aminopurin-9-yl)-3,4-dihydroxyoxolan-2-yl]methyl dihydrogen phosphate, 5'-adenylic acid, 5' AMP, 5'-AMP, 61-19-8, adenosine-5-monophosphate, adenosine-5-phosphate, adenosine monophosphate, C10H14N5O7P
AMPK	AMP-activated kinase, AMP KINASE, Amp-pk
ATG13	1110053A20Rik, autophagy related 13, D2Ertd391, D2Ertd391e, Harbi1, Harbi1l, KIAA0652, PARATARG8, RGD1310685
DAG	DAG, diacylglycerides, diglyceride
DDIT4	5830413E08Rik, AA415483, Dig, Dig2, DKFZP564O2071, DNA-damage-inducible transcript 4, FLJ20500, REDD, REDD-1, Rtp8, Rtp801
DGKZ	80-kDa Dg Kinase, DAGK5, DAGK6, Dgk4, DGK-ZETA, DGK-ζ, Diacylglycerol kinase, diacylglycerol kinase zeta, diacylglycerol kinase ζ, E130307B02Rik, F730209L11Rik, hDGKzeta, KDGZ, mDGK[z]
Eif-4a	Eukaryotic translation initiation factor 4a, Homologous to SP P44586 ATP-dependent RNA helicase DEAD
EIF4B	2310046H11RIK, AL024095, C85189, Eif4a2, eIF4B, eukaryotic translation initiation factor 4B, Initiation Factor M3, PRO1843
EIF4E	AUTS19, CAP-binding, CBP, EG668879, eIF-4, EIF4E1, EIF4EL1, Eif4e-ps, EIF4F, eukaryotic translation initiation factor 4E, If4, If4e
EIF4EBP1	4e-bp, 4E-BP1, AA959816, BP-1, Eukaryotic translation initiation factor 4e binding protein 1, PH, PHAS-I
eIF4G	eIF4gamma, eIF4γ
ERK1/2	MAPK p44/42, MAPK p44/p42, p42/44 mapk, P42/p44 erk, P42/p44 mapk, p42/p44 MAP KINASE
FKBP1A	FK506 binding protein 1a, Fkb, Fkbp, FKBP1, FKBP-12, Fkbp2, FKBP prolyl isomerase 1A, FPK1, macrophilin-12, PKC12, PKCI2, PPIASE
HIF1A	AA959795, bHLHe7, bHLHe78, HIF-1, HIF1-ALPHA, HIF-1alpha (hydroxylated), HIF-1-α, HIF-1α (hydroxylated), Hypoxia inducible factor 1 alpha subunit, hypoxia inducible factor 1, alpha subunit, hypoxia inducible factor 1 subunit alpha, hypoxia inducible factor 1 subunit α, Hypoxia inducible factor 1 α subunit, hypoxia inducible factor 1, α subunit, MO, MOP1, PASD8
INSR	4932439J01Rik, alpha subunit INSULIN receptor, CD220, D630014A15RIK, HHF5, I, insulin receptor, INSULIN receptor B, Insulin receptor beta, INSULIN receptor KINASE, Insulin receptor β, INSULIN RPTK, IR, IR alpha, IR-B, IRK, IR α, α subunit INSULIN receptor
INSULIN	Ins, Ins1/2, proinsulin
IRS1	ENSMUSG00000022591, G972, G972R, HIRS-1, insulin receptor substrate 1, IR, IRS1IRM
MAPKAP1	AI591529, D230039K05Rik, JC310, MAPK associated protein 1, MIP1, mitogen-activated protein kinase associated protein 1, mSIN1, S, SIN1
MLST8	0610033N12Rik, AA409454, AI505104, AI851821, Gb, GbetaL, GBL, GβL, LST8, mLS, MTOR associated protein, LST8 homolog, MTOR associated protein, LST8 homolog (S. cerevisiae), POP3, WAT1
MTOR	2610315D21Rik, AI327068, fl, Flat, Fr, FRAP, FRAP1, FRAP2, FRB, mechanistic target of rapamycin kinase, RA, RAF, RAFT1, RAPT1, RRAFT1, SKS
PA	1,2-diacyl-sn-glycerol-3-phosphate, diacylglycerophosphates, PA, phospholipids alcohol, PtdOH
PC	3-sn-phosphatidylcholine, C10H18NO8PR2, choline glycerophospholipid, diacylglycerophosphocholines, lecithin, lecithins, lecithin, soy, lecithin, soybean, LT-02, PC, phosphotidylcholine, soya phosphatidyl choline, soybean phospholipids, soy lecithin
PDPK1	3'-PDK, 3-phosphoinositide dependent protein kinase-1, Pdk, PDK1, PDPK2, PDPK2P, PRO0461
PI3K	1-phosphatidylinositol 3-kinase, 2.7.1.137, ATP:1-phosphatidyl-1D-myo-inositol 3-phosphotransferase, Phosphatidylinositol 3 kinase, phosphatidylinositol 3'-kinase, PI3-kinase, PtdIns 3 Kinase, type III phosphoinositide 3-kinase, type I phosphatidylinositol kinase, Vps34p
PIP2	1,2-diacyl-sn-glycero-3-phospho-(1'-myo-inositol-4',5'-bisphosphate), 1-O-(3-sn-phosphatidyl)-1D-myo-inositol 4,5-bis(dihydrogen phosphate), 1-phosphatidyl-1D-myo-inositol 4,5-bisphosphate, C11H19O19P3R2
PIP3	1-phosphatidyl-1D-myo-inositol 3,4,5-trisphosphate, phosphatidylinositol-3,4,5-trisphosphate, phosphoinositide (3,4,5) P3, PI(3,4,5)P3, PIns(3,4,5)P3, PIP3, PtdIns(3,4,5)P3
PKC	Cnpkc, PKC, Pkc(s), Protein Kinase C
PLD	3.1.4.4, choline phosphatase, lecithinase D, lipophosphodiesterase II, phosphatidylcholine phosphatidohydrolase, PHOSPHOLIPASE D
PMA	12-O-tetradecanoylphorbol-13-acetate, 16561-29-8, [(1S,2S,6R,10S,11R,13S,14R,15R)-13-acetyloxy-1,6-dihydroxy-8-(hydroxymethyl)-4,12,12,15-tetramethyl-5-oxo-14-tetracyclo[8.5.0.02,6.011,13]pentadeca-3,8-dienyl] tetradecanoate, 4beta-PMA, beta-PMA, C36H56O8, myristic acid, 9-ester with 1,1a-alpha,1b-beta,4,4a,7a-alpha,7b,8,9,9a-decahydro-4a-beta,7b-alpha,9-beta,9a-alpha-tetrahydroxy-3-(hydroxymethyl)-1,1,6,8-alpha-tetramethyl-5H-cyclopropa(3,4)benz(1,2-e)azulen-5-one, 9a-acetate, myristic acid, 9-ester with 1,1a-α,1b-β,4,4a,7a-α,7b,8,9,9a-decahydro-4a-β,7b-α,9-β,9a-α-tetrahydroxy-3-(hydroxymethyl)-1,1,6,8-α-tetramethyl-5H-cyclopropa(3,4)benz(1,2-e)azulen-5-one, 9a-acetate, phorbol 12-myristate 13-acetate, phorbol myristate acetate, PMA, tetradecanoic acid, 9a-(acetyloxy)-1a,1b,4,4a,5,7a,7b,8,9,9a-decahydro-4a,7b-dihydroxy-3-(hydroxymethyl)-1,1,6,8-tetramethyl-5-oxo-1H-cyclopropa(3,4)benz(1,2-e)azulen-9-yl ester, (1aR-(1aalpha,1bbeta,4abeta,7aalpha,7balpha,8alpha,9beta,9aalpha))-, tetradecanoyl-phorbol-13-acetate, TPA, β-12-O-tetradecanoylphorbol-13-acetate, β-phorbol 12-myristate 13-acetate, β-phorbol-12 β-myristate-13 α-acetate, β-PMA
PP2A	protein PHOSPHATASE 2A, Protein Phosphatase Type2a
PRKCA	AAG6, AI875142, LOC146784, Pk, PKCA, PKC-alpha, PKCI+/-, PKC-α, PKRCA, PRKACA, protein kinase C alpha, protein kinase C, alpha, protein kinase C α, protein kinase C, α, α-protein kinase C
RAC1	AL023026, D5Ertd559, D5Ertd559e, MIG5, MRD48, p21-Rac1, p21-RAC, Rac, Rac family small GTPase 1, TC-25
Rapamycin	1402453-65-9, (1R,9S,12S,15R,16E,18R,19R,21R,23S,24E,26E,28E,30S,35R)-1,18-dihydroxy-12-[(2R)-1-[(1S,3R,4R)-4-hydroxy-3-methoxycyclohexyl]propan-2-yl]-19,30-dimethoxy-15,17,21,23,29,35-hexamethyl-11,36-dioxa-4-azatricyclo[30.3.1.04,9]hexatriaconta-16,24,26,28-tetraene-2,3,10,14,20-pentone, (3S,6R,7E,9R,10R,12R,14S,15E,17E,19E,21S,23S,26R,27R,34aS)-9,10,12,13,14,21,22,23,24,25,26,27,32,33,34,34a-Hexadecahydro-9,27-dihydroxy-3-[(1R)-2-[(1S,3R,4R)-4-hydroxy-3-methoxycyclohexyl]-1-methylethyl]-10,21-dimethoxy-6,8,12,14,20,26-hexamethyl-23,27-epoxy-3H-pyrido[2,1-c][1,4]oxaazacyclohentriacontine-1,5,11,28,29(4H,6H,31H)-pentone, 53123-88-9, ABI-009, AY 22-989, C51H79NO13, erapa, I-2190A, nab-rapamycin, nanoparticle albumin-bound rapamycin, NSC 226080, Rapamune, Rapamycin, SEL-110, SILA 9268A, SVP-rapamycin, WY-090217
RHEB	Ras homolog enriched in brain, Ras homolog, mTORC1 binding, RHEB1, RHEB2
RHO	GTPase Rho, Rho, Rho Family, RHO-GTPASE, Rho-like Gtpase
Ribosomal40s subunit	40s, 40S ribosomal subunit, 40S RIBOSOME
RICTOR	4921505C17Rik, 6030405M08Rik, AVO3, AW492497, D530039E11Rik, hAVO3, KIAA1999, Mtorc2, PIA, RPTOR independent companion of MTOR complex 2, RPTOR independent companion of MTOR, complex 2
RPS6	40S ribosomal protein S6-like, LOC100911372, pp33, Q9BZU1, RIBOSOMAL protein S6, S, S6, S6R, S6RP
RPS6KB1	2610318I15RIK, 4732464A07RIK, AA959758, AI256796, AI314060, P70, p70/85s, p70/85s6k, p70-alpha, p70s, p70S6, p70s6k, P70S6K1, p70 S6K-alpha, p70S6 kinase, p70 S6K-α, p70(S6K)-α, p70-α, PS6K, ribosomal protein S6 kinase B1, ribosomal protein S6 kinase, polypeptide 1, S6K, S6K1, S6K-beta-1, S6K-β-1, STK14A
RPTOR	4932417H02Rik, KOG1, Mip1, mKIAA1303, r, Rap, RAPTOR, regulatory associated protein of MTOR complex 1, regulatory associated protein of MTOR, complex 1, RGD1311784
RSK	p90RSK
STK11	AA408040, hLKB1, Lkb, LKB1, LKB1-L, LKB1(S), Pa, Par-4, PJS, R75140, serine/threonine kinase 11, Stk11 isoform 2, Stk11 short isoform
TSC1	ham, Hamartin, LAM, TSC, TSC complex subunit 1
Tsc1-Tsc2	TSC, TSC1/2
TSC2	LAM, Na, Nafld, PPP1R160, Rc, Tcs2, TSC4, TSC complex subunit 2, tube, TUBERIN
ULK1	ATG1, ATG1A, AU041434, hATG1, mKIAA0722, ULK, Ulk1 mapped, UNC51, Unc51., Unc51.1, unc-51 like autophagy activating kinase 1, unc-51 like kinase 1

Pathway Analysis Using IPA Software; canonical pathway

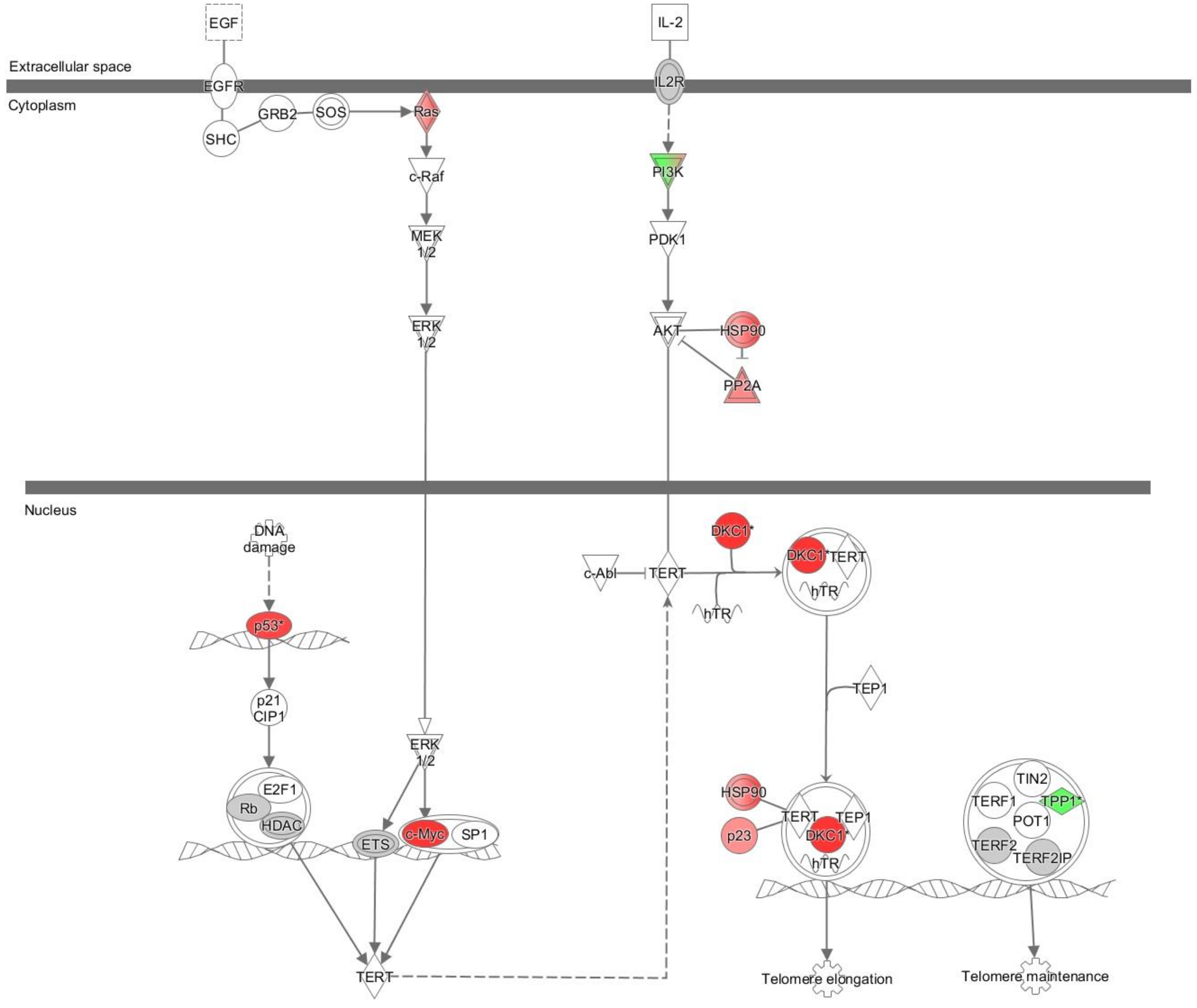

Figure S52. Telomerase Signaling at 24 h

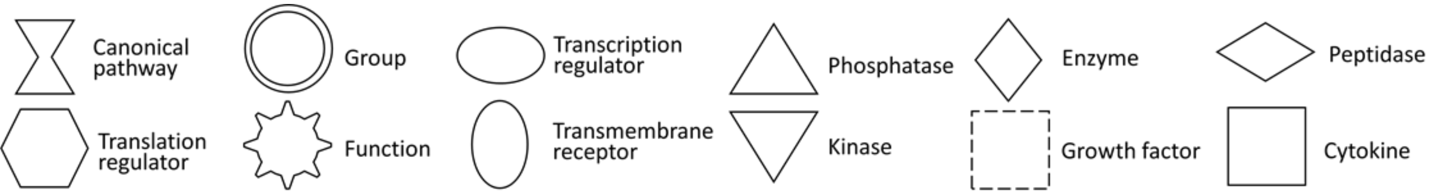

Red: Increased, FDR<0.05 versus solvent control

Green: Decreased, FDR<0.05 versus solvent control

Symbol	Synonym(s)
ABL1	ABL, ABL proto-oncogene 1, non-receptor tyrosine kinase, AI325092, BCR-ABL, c-A, c-ABL, CABL1, c-abl oncogene 1, non-receptor tyrosine kinase, CHDSKM, E430008G22Rik, JTK7, LOC100909750, p145Abl, p150, tyrosine-protein kinase ABL1-like, v-abl
AKT	AKT1/2/3, B/Akt, PKB, RAC-PK
CDKN1A	CAP, CAP20, CDK, CDKI, Cdkn, CDKN1, CDKNA1, CI, CIP1, cyclin-dependent kinase inhibitor 1A, cyclin-dependent kinase inhibitor 1A (P21), mda, MDA-6, P2, P21, p21C, p21Cip, p21CIP1, p21W, p21WAF, p21Waf1, Pzl Cyclin-Dependent Kinase Inhibitor, SD, SDI1, UV96, Waf, WAF1
DKC1	BC068171, CBF5, DKC, DKCX, dyskeratosis congenita 1, dyskerin, dyskerin pseudouridine synthase 1, NAP57, NOLA4, Weakly similar to tyrosine-prtein kinase jak3, XAP101
E2F1	E2f, E2F transcription factor 1, mKIAA4009, RBAP1, RBBP3, RBP3, Tg(Wnt1-cre)2Sor
EGF	AI790464, EGF-1, epidermal growth factor, HOMG4, URG
EGFR	9030024J15RIK, AI552599, C-ERBB, EGFR1, EGF receptor, EGFR vIII, EGF-TK, epidermal growth factor receptor, Erb, ERBB, ERBB1, Err, Errb1, ERRP, HER1, HER1 (EGFR), MENA, NISBD2, PIG61, Wa, wa-2, Wa5
ERK1/2	MAPK p44/42, MAPK p44/p42, p42/44 mapk, P42/p44 erk, P42/p44 mapk, p42/p44 MAP KINASE
GRB2	AA408164, ASH, Ash-psi, EGFRBP-GRB2, GRAB2, GRBS, growth factor receptor bound protein 2, MST084, MSTP084, NCKAP2
HDAC	Histone Deacetylase, Histone deacetyltransferase
HSP90	HSC90, Hsp84
IL2	IL, interleukin 2, lymphokine, TCGF
IL2R	Il2 Receptor
MAP2K1/2	MEK1/2, MKK1/2
MYC	AU016757, bHLHe3, bHLHe39, CMYC, C-MYC-P64, mMyc, MRTL, Myc2, MYCC, MYC proto-oncogene, bHLH transcription factor, myelocytomatosis oncogene, N, Niard, Nird, RNCMYC
PDPK1	3'-PDK, 3-phosphoinositide dependent protein kinase-1, Pdk, PDK1, PDPK2, PDPK2P, PRO0461
PI3K	1-phosphatidylinositol 3-kinase, 2.7.1.137, ATP:1-phosphatidyl-1D-myo-inositol 3-phosphotransferase, Phosphatidylinositol 3 kinase, phosphatidylinositol 3'-kinase, PI3-kinase, PtdIns 3 Kinase, type III phosphoinositide 3-kinase, type I phosphatidylinositol kinase, Vps34p
POT1	1500031H18Rik, AI851169, CMM10, GLM9, HPOT1, Po, Pot1a, protection of telomeres 1, protection of telomeres 1A
PP2A	protein PHOSPHATASE 2A, Protein Phosphatase Type2a
PTGES3	5730442A20Rik, cPG, cPGES, Gm9769, p23, p23 COCHAPERONE, p23 PR RELATED, PGES3, prostaglandin E synthase 3, prostaglandin E synthase 3, pseudogene, Ptg, Ptges, Ptges3-ps, RGD1561913, sid31, sid3177, Teb, TEBP, Telomerase Binding Protein p23, Zhf6
RAF1	6430402F14Rik, AA990557, BB129353, CMD1NN, c-R, Cra, CRAF, Craf1, D830050J10Rik, leukaemiA ONCOGENE HOMOLOG1, LEUKEMIA ONCOGENE HOMOLOG1, NS5, Raf-1 proto-oncogene, serine/threonine kinase, v-, v-Raf, v-raf-leukaemia viral oncogene 1, v-raf-leukemia viral oncogene 1
RB1	OSRC, p, p105, p105-Rb, p110 RB, p110-RB1, pp105, pp110, PPP1R130, pRb, R, RB, RB-ASSOCIATED, RB transcriptional corepressor 1, Retinoblastome tumor-suppression protein rb
SHC1	p52SHC, p6, p66, p66s, P66shc, Sh, SHC, Shc (46 kDa isoform), SHCA, SHC adaptor protein 1, Shc p66 isoform, src homology 2 domain-containing transforming protein C1
SP1	1110003E12RIK, AA450830, AI845540, Sp1-1, Sp1 transcription factor, Sp1 (trans spliced isoform), Trans-acting transcription factor 1
TEP1	p240, telomerase associated protein 1, TLP1, Tp, TP1, TROVE1, VAULT2
TERF1	hTRF1-AS, P, PIN2, telomeric repeat binding factor 1, Trbf, TRBF1, TRF, TRF1, t-TRF1
TERF2	telomeric repeat binding factor 2, TRBF2, TRF, TRF2
TERF2IP	DRIP5, R, RAP1, telomeric repeat binding factor 2, interacting protein, TERF2 interacting protein
TERT	CMM9, DKCA2, DKCB4, EST2, hEST2, HTERT, hTRT, PFBMFT1, T, TCS1, TELOMERASE, Telomerase Catalytic Subunit, Telomerase Reverse Transcriptase, TP2, TR, TRT
TINF2	AW552114, D14Wsu146, D14Wsu146e, DKCA3, TERF1 interacting nuclear factor 2, Terf1 (TRF1)-interacting nuclear factor 2, TI, TIN2
TP53	bbl, BCC7, bfy, bhy, BMFS5, LFS1, p4, p44, p5, P53, P53 cellular tumour antigen, p53 tumor suppressor, transformation related protein 53, TRP53, tumor protein p53, tumour protein p53
TPP1	CI, CLN2, GIG1, LPIC, SCAR7, TPP-I, tripeptidyl peptidase 1, Tripeptidyl peptidase i

Pathway Analysis Using IPA Software; canonical pathway

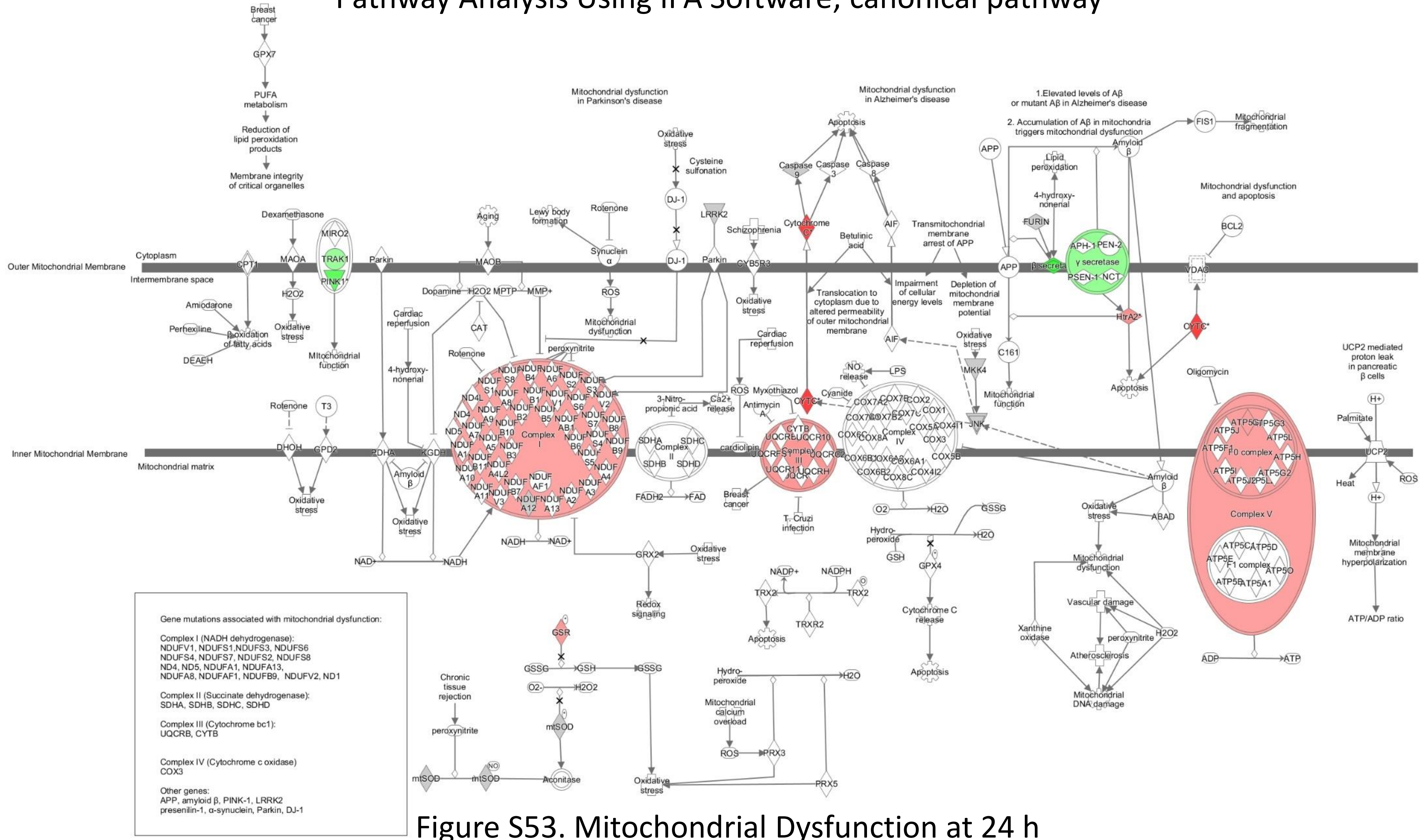

Figure S53. Mitochondrial Dysfunction at 24 h

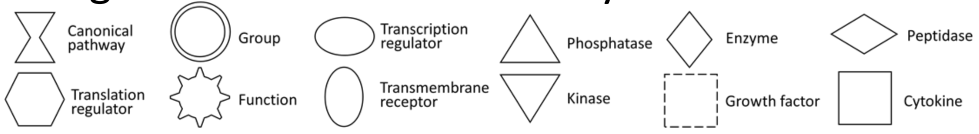

Red: Increased, FDR<0.05 versus control

Green: Decreased, FDR<0.05 versus control

Symbol	Synonym(s)
3-Nitro-propionic acid	3-nitropropanoic acid, 3-nitropropionate, 3-NP, 3-NP acid, 504-88-1, beta-nitropropanoate, beta-nitropropionic acid, BNP, C3H5NO4, propanoic acid, 3-nitro-, propanoic acid, 3-nitro- (9CI), β-nitropropanoate, β-nitropropionic acid
4-hydroxy-nonenal	29343-52-0, 2-Nonenal, 4-hydroxy-, 4-HNE, 4-hydroxy-2,3-nonena, 4-hydroxy-2,3-nonenal, 4-hydroxy-2-nonenal, 4-hydroxynon-2-enal, 75899-68-2, C9H16O2
Aconitase	4.2.1.3, aconitate hydratase, cis-aconitase, citrate(isocitrate) hydro-lyase
ADP	20398-34-9, [(2R,3S,4R,5R)-5-(6-aminopurin-9-yl)-3,4-dihydroxyoxolan-2-yl]methyl phosphono hydrogen phosphate, 58-64-0, 9-beta-D-arabinofuranosyladenine 5'-diphosphate, 9-β-D-arabinofuranosyladenine 5'-diphosphate, adenosine 5'-(trihydrogen diphosphate), adenosine diphosphate, C10H15N5O10P2
AIFM1	A, AIF, apoptosis inducing factor mitochondria associated 1, apoptosis inducing factor, mitochondria associated 1, apoptosis-inducing factor, mitochondrion-associated 1, AUNX1, CMT2D, CMTX4, COWCK, COXPD6, DFNX5, Hq, NADMR, NAMSD, Pdcd, PDCD8, SEMDHL
Amiodarone	1951-25-3, 19774-82-4, (2-butyl-1-benzofuran-3-yl)-[4-[2-(diethylamino)ethoxy]-3,5-diiodophenyl]methanone, 2-butyl-3-benzofuryl 4-(2-(diethylamino)ethoxy)-3,5-diiodophenyl ketone hydrochloride, AMD, Aminodarone, Amio-Aqueous IV, Amiodarex, amiodarone HCl, amiodarone hydrochloride, Amiodarons, Amiohexal, Amiorone, Aratac, Arycor, C25H29I2NO3, Cardarone, Cordarone, Cordarone Intravenous, Cordarone I.V., Labaz, Nexterone, Pacerone, pms-Amiodarone, Rythmarone
Antimycin A	1397-94-0, ALA, AMA, anthimycin A, antimycin, antimycin A
APP	A, AAA, Abe, Abetapp, ABPP, AD1, Adap, Ag, alpha-sAPP, amyloid beta (A4) precursor protein, amyloid beta precursor protein, amyloid-beta-protein, Amyloidogenic glycoprotein, Amyloid precursor, amyloid β (A4) precursor, Amyloid β A4 precursor, amyloid β (A4) precursor protein, amyloid β A4 precursor protein, amyloid β precursor, amyloid β precursor protein, amyloid-β-protein, amyloid β-protein precursor, APPI, appican, APP isoform 1, bet, beta amyloid precursor, betaApp, C, CTFgamma, CVAP, E030013M08RIK, P3, PN2, PN-II, PreA4, protease nexin2, α-sAPP, β-amyloid precursor, β APP, β PP
ATP	[[(2R,3S,4R,5R)-5-(6-aminopurin-9-yl)-3,4-dihydroxyoxolan-2-yl]methoxy-hydroxyphosphoryl] phosphono hydrogen phosphate, 56-65-5, 9-beta-D-arabinofuranosyladenine 5'-triphosphate, 9-β-D-arabinofuranosyladenine 5'-triphosphate, adenosine 5'-(tetrahydrogen triphosphate), adenosine 5'-triphosphate, ATP, ATP4-, C10H16N5O13P3
ATPSynthase	COMPLEX V, C V, Electron Transport Chain Complex V, ETC complex V, F0,f1-Atpase, F0F1 ATP Synthase, F1f0-ATPase, F1F0 ATP Synthase, OxPhosV, RESPIRATORY CHAIN COMPLEX V, Respiratory Complex V
ATP5F1A	AI035633, AL022851, AL023067, alpha subunit of the F1F0 ATP SYNTHASE (COMPLEX V), At, ATP5A, ATP5A1, ATP5AL2, ATPA, ATPM, ATP synthase alpha, Atp synthase alpha chain, ATP SYNTHASE alpha subunit, Atp synthase (f0f1), subunit alpha, Atp synthase (f0f1), subunit α, ATP Synthase F1 alpha, ATP synthase F1 subunit alpha, ATP synthase F1 subunit α, ATP Synthase F1 α, ATP synthase, H+ transporting, mitochondrial F1 complex, alpha subunit 1, ATP synthase, H+ transporting, mitochondrial F1 complex, α subunit 1, ATP synthase mitochondrial f1complex, ATP synthase α, Atp synthase α chain, ATP SYNTHASE α subunit, Complex V subunit I, COXPD22, D18Ertd206, D18Ertd206e, F1 atpase, F1 ATPase alpha, F1 ATPase α, hATP1, HEL-S-123m, MC5DN4, Mitochondrial ATPS, Mom, MOM2, OMR, ORM, Q3u452, α subunit of the F1F0 ATP SYNTHASE (COMPLEX V)
ATP5F1B	ATP5B, ATP5beta, ATP5β, ATPB, Atpd, ATPMB, ATPSB, ATP Synthase Beta, ATP Synthase F1 Beta, ATP synthase F1 subunit beta, ATP synthase F1 subunit β, ATP Synthase F1 β, ATP synthase, H+ transporting mitochondrial F1 complex, beta subunit, ATP synthase, H+ transporting mitochondrial F1 complex, β subunit, ATP Synthase β, Beta atp synthase, F1 ATPase beta, F1 ATPase β, F1 Atp Synthase β, F1 β atpase, F-type ATPase b, H+ ATP Synthase Beta, H+ ATP Synthase β, HEL-S-271, OXPHOS COMPLEX V, subunit B, β atp synthase, β subunit ATP synthase
ATP5F1C	1700094F02Rik, ATP5C, ATP5C1, ATP5CL1, ATP synthase F1 subunit gamma, ATP synthase F1 subunit γ, ATP synthase, H+ transporting, mitochondrial F1 complex, gamma polypeptide 1, ATP synthase, H+ transporting, mitochondrial F1 complex, γ polypeptide 1, F1 γ, γ subunit f1f0 atpase
ATP5F1D	0610008F14RIK, 1500000I11Rik, AA960090, AI876556, ATP5D, Atpase D, ATPD, ATP Synthase F1 Delta, ATP synthase F1 subunit delta, ATP synthase F1 subunit δ, ATP Synthase F1 δ, ATP synthase, H+ transporting, mitochondrial F1 complex, delta subunit, ATP synthase, H+ transporting, mitochondrial F1 complex, δ subunit, AU020773, C85518, F1F0 ATPase delta, F1F0 ATPase δ, LOC100910032, LOC687032, LOC690935, MC5DN5
ATP5F1E	2410043G19Rik, ATP5E, ATPE, ATP synthase F1 subunit epsilon, ATP synthase F1 subunit ε, AV000645, MC5DN3
ATP5MC1	ATP5A, ATP5G, ATP5G1, ATP9C, ATP synthase, H+ transporting, mitochondrial F0 complex, subunit C1 (subunit 9), ATP synthase membrane subunit c locus 1
ATP5MC2	1810041M08Rik, ATP5A, ATP5G2, ATP synthase, H+ transporting, mitochondrial F0 complex, subunit C2 (subunit 9), Atp Synthase Lipid-Binding Protein P2 Precursor, ATP synthase membrane subunit c locus 2, LOC100504871
ATP5MC3	6030447M23, ATP5G3, ATP synthase, H+ transporting, mitochondrial F0 complex, subunit C3 (subunit 9), ATP synthase membrane subunit c locus 3, ATP Synthase Subunit c, P3
ATP5ME	2610008D24Rik, ATP5I, ATP5K, ATP synthase membrane subunit e, Lfm, Lfm1
ATP5MF	1110019H14Rik, ATP5J2, ATP5JL, ATP synthase, H+ transporting, mitochondrial F0 complex, subunit F2, ATP synthase membrane subunit f, LOC684567
ATP5MG	4933437C06RIK, ATP5JG, ATP5L, Atp synthase (f0f1), subunit g, ATP synthase, H+ transporting, mitochondrial F0 complex, subunit G, ATP synthase membrane subunit g, F1F0-ATP Synthase G Subunit, RGD:1303259
ATP5MGL	ATP5K2, ATP5L2, ATP synthase membrane subunit g like
ATP5PB	Atp5, ATP5F1, ATP synthase peripheral stalk-membrane subunit b, ATP synthase subunit b, mitochondrial-like, C76477, LOC100911417, PIG47
ATP5PD	0610009D10RIK, APT5H, ATP5H, Atp5jd, ATPQ, Atp synthase d subunit, ATP synthase, H+ transporting, mitochondrial F0 complex, subunit D, ATP synthase peripheral stalk subunit d, ATP Synthase Subunit D, H+ ATP Synthase Subunit D
ATP5PF	ATP5, ATP5A, ATP5J, ATPM, ATP SYNTHASE, Atp synthase (f0f1), subunit f, ATP synthase, H+ transporting, mitochondrial F0 complex, subunit F, ATP synthase peripheral stalk subunit F6, CF6, F6
ATP5PO	ATP5O, ATPO, Atp Synthase, H+ Transporting, Mitochondrial F1 Complex, O Subunit, Atp synthase-mitochondrial f1, ATP synthase peripheral stalk subunit OSCP, D12Wsu28, D12Wsu28e, HMC08D05, OSCP
BCL2	AW986256, B cell leukaemia/lymphoma 2, B cell leukemia/lymphoma 2, Bcl-, Bcl2 alpha, BCL2 apoptosis regulator, BCL2, apoptosis regulator, Bcl2 α, C430015F12Rik, D630044D05RIK, D830018M01RIK, LOC100046608, ORF16, PPP1R50
Betasecretase	β Secretase
Betulinicacid	(1R,3aS,5aR,5bR,7aR,9S,11aR,11bR,13aR,13bR)-9-hydroxy-5a,5b,8,8,11a-pentamethyl-1-prop-1-en-2-yl-1,2,3,4,5,6,7,7a,9,10,11,11b,12,13,13a,13b-hexadecahydrocyclopenta[a]chrysene-3a-carboxylic acid, 3-hydroxylup-20(29)-en-28-oic acid, 472-15-1, C30H48O3, lup-20(29)-en-28-oic acid, 3beta-hydroxy- (8CI), lup-20(29)-en-28-oic acid, 3-hydroxy-, (3beta)-, topical ALS-357, topical betulinic acid
cardiolipin	[(2R)-3-[[3-[[(2R)-2,3-di(octadecanoyloxy)propoxy]-hydroxyphosphoryl]oxy-2-hydroxypropoxy]-hydroxyphosphoryl]oxy-2-octadecanoyloxypropyl] octadecanoate, C81H158O17P2, diphosphatidylglycerol
CASP3	A830040C14Rik, AC-, AC-3, Casp, Caspase-3, CASPASE-3 p20, CC3, CPP, CPP-32, CPP32B, CPP32-beta, CPP32-β, Ice-like cysteine protease, Lice, mld, mldy, SCA-1, Ya, YAMA
CASP8	ALPS2B, CAP4, Casp, Caspase-8, FLI, FLICE, MAC, MACH, MCH5, PROCASP8
CASP9	AI115399, APAF-3, AW493809, Casp, Casp9 v1, Caspase-9, ICE-, ICE-LAP6, MCH6, PPP1R56
CAT	2210418N07, ACATALASIA, Ca, Cas, Cas-1, Cat01, Catalase, Catalase1, Catl, Cs-, CS1
COX4I1	AL024441, CcO IVi1, CO, COX, COX4, COX4-1, COX4A, COX4I, COXIV, COX IV-1, cytochrome c oxidase subunit 4I1, IV-1, MC4DN16
COX4I2	COX4, COX4-2, COX4B, COX4L2, COXIV-2, cytochrome c oxidase subunit 4I2, dJ857M17.2
COX5A	AA959768, CcO, CcOX, COX, COX-VA, cytochrome c oxidase subunit 5A, Cytochrome c oxidase subunit va, MC4DN20, Mitochondrial Cytochrome C Oxidase Va, VA
COX5B	COXVB, cytochrome c oxidase subunit 5B
COX6A1	CMTRID, COX6A, COX6AL, cytochrome c oxidase subunit 6A1, VIaL
COX6A2	COX, COX6AH, COXVIAH, Cytochrome c oxidase, polypeptide VIB, cytochrome c oxidase subunit 6A2, MC4DN18, V, VIaH
COX6B1	2010000G05Rik, COX6B, COXG, COX VIb-1, cytochrome c oxidase subunit 6B1, cytochrome c oxidase, subunit 6B1, MC4DN7
COX6B2	1700067P11Rik, BC048670, COXV, COXVIB2, CT59, cytochrome c oxidase subunit 6B2
COX6C	COVIc, Cox6c2, COX-VIc, cytochrome c oxidase subunit 6C, cytochrome c oxidase subunit 6C2, EG621837, Gm6265
COX7A1	COX, COX7, COX7A, COX7AH, COX7AM, cytochrome c oxidase subunit 7A1
COX7A2	COX, Cox7, COX7A3, COX7AL, COX7AL1, COXVIIAL, cytochrome c oxidase subunit 7A2, VIIAL
COX7B	1110004F07Rik, APLCC, C80563, cytochrome c oxidase subunit 7B, LSDMCA2
COX7B2	4930503B16Rik, cytochrome c oxidase subunit 7B2
COX7C	AI648091, COX, Cox7c1, COXVIIc, cytochrome c oxidase subunit 7C
COX8A	COX, COX8, COX8-2, COX8L, COX VIII, cytochrome c oxidase subunit 8A, cytochrome c oxidase subunit viii, Cytochrome C Oxidase Subunit Viiia, Cytochrome oxidase subunit 8, MC4DN15, VIII, VIII-L
COX8C	1700007F21Rik, COX8-, COX8-3, COXV, COXVIII-3, cytochrome c oxidase subunit 8C
CPT1	Cpt-i
Cyanide	57-12-5, CN-, cyanide, cyanide(1-)
CYB5R3	0610016L08Rik, 2500002N19Rik, B5R, C85115, cytochrome b5 reductase 3, Di, Dia, DIA1, Nadhcb5, NADH Cytochrome B5 Reductase, RNNADHCB5, WU:Cyb5r3
CYCS	CYC, CYCSA, CYTC, CYTOC, CYTOCHROME C, cytochrome c, somatic, cytochrome c, somatic-like, ENSMUSG00000058927, HCS, LOC100363502, THC4, X laevis XLCL2
Cytochromebc1	1.10.2.2, coenzyme Q-cytochrome c reductase, coenzyme QH2-cytochrome c reductase, Complex III, CoQH2-cytochrome c oxidoreductase, dihydrocoenzyme Q-cytochrome c reductase, ETC complex III, Mitochondrial Complex 3, Mitochondrial Complex III, Mitochondrial Electron Transport Chain Complex 3, mitochondrial electron transport complex III, Oxphos Complex III, QH2:cytochrome c oxidoreductase, reduced coenzyme Q-cytochrome c reductase, reduced ubiquinone-cytochrome c oxidoreductase, reduced ubiquinone-cytochrome c reductase, complex III (mitochondrial electron transport), RESPIRATORY CHAIN COMPLEX III, RESPIRATORY CHAIN III COMPLEX III, ubihydroquinol:cytochrome c oxidoreductase, ubiquinol-cytochrome c1 oxidoreductase, ubiquinol-cytochrome c-2 oxidoreductase, ubiquinol-cytochrome c2 reductase, ubiquinol-cytochrome c oxidoreductase, ubiquinol-cytochrome-c reductase, ubiquinol:ferricytochrome-c oxidoreductase, ubiquinone-cytochrome b-c1 oxidoreductase, ubiquinone-cytochrome c oxidoreductase, ubiquinone-cytochrome c reductase

Symbol	Synonym(s)
Cytochrome-coxidase	1.9.3.1, Complex IV, complex IV (mitochondrial electron transport), COX, COXIV, cytochrome a3, cytochrome aa3, cytochrome oxidase, Cyto-c oxi, ETC complex IV, ferrocytochrome c oxidase, ferrocytochrome-c:oxygen oxidoreductase, indophenolase, indophenol oxidase, MITOCHONDRIAL COMPLEX IV, MITOCHONDRIAL RESPIRATORY CHAIN COMPLEX IV, NADH cytochrome c oxidase, Oxphos Complex IV, RESPIRATORY CHAIN COMPLEX IV, Warburg respiratory enzyme, Warburg's respiratory enzyme, Warburgs respiratory enzyme
DEAEH	2,2'-((1,2-diethylethylene)bis(p-phenyleneoxy))bis(triethyl)amine, 2-[4-[4-[4-[2-(diethylamino)ethoxy]phenyl]hexan-3-yl]phenoxy]-N,N-diethylethanamine, 2691-45-4, 4,4'-bis(beta-diethylaminoethoxy)alpha,beta-diethyldiphenylethane, 4,4'-bis(β-diethylaminoethoxy)α,β-diethyldiphenylethane, 4,4'-diethylaminoethoxyhexestrol, 69-14-7, C30H48N2O2, coralgil, diethylaminoethoxyhexestrol, trimanyl
Dexamethasone	137098-19-2, 23495-06-9, 50-02-2, 8054-59-9, (8S,9R,10S,11S,13S,14S,16R,17R)-9-fluoro-11,17-dihydroxy-17-(2-hydroxyacetyl)-10,13,16-trimethyl-6,7,8,11,12,14,15,16-octahydrocyclopenta[a]phenanthren-3-one, 906422-84-2, 9-fluoro-11b,17,21-trihydroxy- 16a-methylpregna-1,4-diene-3,20-dione, Adexone, Aeroseb-D, Aeroseb-Dex, Anaflogistico, Aphtasolon, Aphthasolone, AR-1105-CF1, AR-1105-CF2, Auxiron, Azium, Bisu Ds, C22H29FO5, Calonat, Corson, Corsone, Cortisumman, Decacort, Decacortin, Decaderm, Decadron, Decadron-La, Decadron Tablets, Decadron Tablets, Elixir, Decagel, Decaject, Decaject L.A., Decalix, Decameth, Decarex, Decasone, Decaspray, Dectancyl, Dekacort, Deltafluorene, Dergramin, Deronil, Desadrene, Desameton, Deseronil, DEX, Dexa, Dexacen-4, Dexacort, Dexacortal, Dexa-Cortidelt, Dexacortin, Dexa-Cortisyl, Dexadeltone, Dexafarma, Dexair, Dexalona, Dexaltin, Dexa Mamallet, Dexa-Mamallet, Dexameth, Dexamethasone Intensol, dexamethasone lipid microsphere, dexamethazone, Dexamonozon, Dexapolcort, Dexapos, Dexaprol, Dexart, Dexa-Scheroson, Dexa-Sine, Dexason, Dexasone, Dex-Ide, Dexinolon, Dexinoral, Dexone, Dexone 0.5, Dexone 0.75, Dexone 1.5, Dexone 4, Dexonium, Dexpak, Dextelan, Dextenza, dextromethasone, Dexycu, Dexycu Kit, Dezone, Dinormon, Dms, Fluormone, Fluorocort, Fortecortin, Gammacorten, glucocorticoid dexamethasone, Hemady, Hexadecadrol, Hexadrol, Hexadrol Elixir, Hexadrol Tablets, Hl-Dex, IontoDex, Isopto-Dex, LenaDex, Lokalison F, Loverine, Luxazone, Maxidex, Mediamethasone, methylfluorprednisolone, Mexidex, Millicorten, Mymethasone, Oradexon, Orgadrone, Ozurdex, Pet Derm Iii, Pet-Derm Iii, Policort, Posurdex, Prednisolone F, Prednisolon F, pregna-1,4-diene-3,20-dione, 9-fluoro-11,17,21-trihydroxy-16-methyl-, (11beta,16alpha)-, Sk-Dexamethasone, Spoloven, Sunia Sol D, Superprednol, sustained release episcleral dexamethasone, Turbinaire, Visumetazone
DHODH	2810417D19Rik, AI834883, DHOdehase, DHOH, dihydroorotate dehydrogenase, dihydroorotate dehydrogenase (quinone), POADS, URA1
Dopamine	1,2-benzenediol, 4-(2-aminoethyl)-, 1,2-benzenediol, 4-(2-aminoethyl)- (9CI), 4-(2-aminoethyl)benzene-1,2-diol, 50444-17-2, 51-61-6, 62-31-7, C8H11NO2, DA, dopamine HCl, dopamine hydrochloride, hydroxytyramine, Intropin, Revimine
F1 ATPsynthase	F1, F1 ATP Synthase
FAD	146-14-5, 1H-purin-6-amine, flavin dinucleotide, 1H-purin-6-amine, flavine dinucleotide, [[(2R,3S,4R,5R)-5-(6-aminopurin-9-yl)-3,4-dihydroxyoxolan-2-yl]methoxy-hydroxyphosphoryl] [5-(7,8-dimethyl-2,4-dioxobenzo[g]pteridin-10-yl)-2,3,4-trihydroxypentyl] hydrogen phosphate, adenosine 5'-(trihydrogen pyrophosphate), 5'-5'-ester with riboflavine, C27H33N9O15P2, FAD, flavin adenine dinucleotide, flavine adenosine diphosphate, flavitan, riboflavin 5'-adenosine diphosphate, riboflavin 5'-(trihydrogen diphosphate), 5'-5'-ester with adenosine, riboflavin 5'-(trihydrogen diphosphate), P'-5'-ester with adenosine
FADH2	1,5-dihydro-FAD, 1910-41-4, [[(2R,3S,4R,5R)-5-(6-aminopurin-9-yl)-3,4-dihydroxyoxolan-2-yl]methoxy-hydroxyphosphoryl] [5-(7,8-dimethyl-2,4-dioxo-1,5-dihydrobenzo[g]pteridin-10-yl)-2,3,4-trihydroxypentyl] hydrogen phosphate, C27H35N9O15P2
FIS1	2010003O14Rik, CGI-135, fission, mitochondrial 1, Riken cDNA 2010003o14, TTC11
FURIN	9130404I01RIK, BASIC-AMINO-ACID-SPECIFIC FURIN, Fu, FUR, FURIN FROM PACE, furin (paired basic amino acid cleaving enzyme), furin, paired basic amino acid cleaving enzyme, PA, PACE, Pcs, PCSK3, SP, SPC1
Gammasecretase	Gamma Secretase, Secretase γ, γ-Secretase
GLRX2	1700010P22Rik, AI645710, CGI-133, glutaredoxin 2, glutaredoxin 2 (thioltransferase), Grx, GRX2
GPD2	AA408484, AI448216, Alpha-gpd, AU021455, AW494132, GDH2, Gdm1, glycerol-3-phosphate dehydrogenase 2, glycerol phosphate dehydrogenase 2, mitochondrial, Glycerophosphate dehydrogenase, GPDH, Gpdh-m, GPDM, m-GDH, mGPDH, mtGPDH, Tisp38, α-gpd
GPX4	glutathione peroxidase 4, GSHPx-4, Lipid peroxidase, MCSP, mtPHG, mtPHGPx, PHG, PHGPx, phospholipid hydroperoxidase, SMDS, sn, snGPx, snPHGPx
GPX7	3110050F08RIK, AI327032, CL683, glutathione peroxidase 7, GPX, GPX6, GSHPx-7, NPGPX
GSH	(2S)-2-amino-5-[[(2R)-1-(carboxymethylamino)-1-oxo-3-sulfanylpropan-2-yl]amino]-5-oxopentanoic acid, (2S)-2-amino-5-[[(2R)-1-(carboxymethylamino)-1-oxo-3-sulphanylpropan-2-yl]amino]-5-oxopentanoic acid, 70-18-8, C10H17N3O6S, gamma-Glu-Cys-Gly, gamma L-Glu L-Cys Gly, gamma-L-glutamylcysteinylglycine, gamma L glutamyl L cysteinylglycine, glutathione-reduced, glycine, N-(N-L-gamma-glutamyl-L-cysteinyl)-, glycine, N-(N-L-γ-glutamyl-L-cysteinyl)-, GSH, L-glutathione, reduced glutathione, γ-Glu-Cys-Gly, γ L-Glu L-Cys Gly, γ-L-glutamylcysteinylglycine, γ L glutamyl L cysteinylglycine
GSR	AI325518, D8Ertd238, D8Ertd238e, glutathione-disulfide reductase, Glutathione reductase, GR, Gr-1, Gred, GRX, GSRD, HEL-75, HEL-S-122m
GSSG	27025-41-8, (2S)-2-amino-5-[[(2R)-3-[[(2R)-2-[[(4S)-4-amino-4-carboxybutanoyl]amino]-3-(carboxymethylamino)-3-oxopropyl]disulfanyl]-1-(carboxymethylamino)-1-oxopropan-2-yl]amino]-5-oxopentanoic acid, bis(gamma-glutamyl-L-cysteinylglycine) disulfide, bis(γ-glutamyl-L-cysteinylglycine) disulfide, C20H32N6O12S2, glutathione, oxidized, GSSG, oxiglutatione
H2O2	7722-84-1, A-101, Colgate Peroxyl, Eskata, H2O2, hydrogen dioxide, hydrogen peroxide, peroxyl mouthwash, urea hydrogen peroxide
HSD17B10	17b-HSD10, ABAD, Ad, Ads9, CAMR, DUPXp11.22, ER, ERAB, Hadh, HADH2, HCD2, HSD10, HSD10MD, hydroxysteroid (17-beta) dehydrogenase 10, hydroxysteroid 17-beta dehydrogenase 10, hydroxysteroid (17-β) dehydrogenase 10, hydroxysteroid 17-β dehydrogenase 10, Hydroxysteroid dehydrogenase 10, MHBD, MRPP2, MRX17, MRX31, MRXS10, SCHAD, SDR5C1, XH98G2
HTRA2	AI481710, Htr, HtrA serine peptidase 2, MGCA8, mnd, mnd2, O, OMI, PARK13, Pr, PRSS25
Hydro-peroxide	RO2H
JNK	JNK 54/46, Jnk p56, JNK/SAPK, JUN KINASE, p40, p47, Sapk/Jnk
LPS	C211H376N8O126P6, endotoxin, endotoxin protein, lipopolysaccharides, LPS, TLR4 agonist LPS
LRRK2	4921513O20RIK, 9330188B09Rik, AURA17, AW561911, cI-4, cI-46, D630001M17RIK, DARDARIN, DKFZP434H2111, FLJ45829, Gm927, leucine-rich repeat kinase 2, PARK8, RIPK7, ROCO2
MAOA	1110061B18Rik, AA407771, BRNRS, MA, Mao, Monoamine Oxidase A, NC61C12.R1, type A monoamine oxidase
MAOB	6330414K01Rik, MAO, monoamine oxidase B
MAP2K4	JNKK, JNKK1, MAPK/ERK KINASE-1, MAPKK4, MEK4, mitogen-activated protein kinase kinase 4, MKK4, PRKMK4, SAPKK-1, Sek, SEK1, Ser, SERK1, SKK1
Mitochondrialcomplex 1	1.6.5.3, coenzyme Q reductase, complex 1 dehydrogenase, COMPLEX I, complex I (electron transport chain), complex I (mitochondrial electron transport), complex I (NADH:Q1 oxidoreductase), dihydronicotinamide adenine dinucleotide-coenzyme Q reductase, DPNH-coenzyme Q reductase, DPNH-ubiquinone reductase, electron transfer complex I, ETC complex I, Mitochondrial Complex I, mitochondrial dehydrogenase, Mitochondrial Electron Transport Chain Complex 1, mitochondrial electron transport complex 1, mitochondrial electron transport complex I, Mitochondrial Respiratory Chain Complex 1, NADH2 dehydrogenase (ubiquinone), NADH2:ubiquinone oxidoreductase, NADH coenzyme Q1 reductase, NADH-coenzyme Q oxidoreductase, NADH Coenzyme Q Reductase, NADH-CoQ oxidoreductase, NADH-CoQ reductase, NADH Cytochrome C Reductase, NADH Dehydrogenase, Nadh:O2 Oxidoreductase, NADH oxidoreductase, NADH-Q6 oxidoreductase, NADH-ubiquinone-1 reductase, NADH-ubiquinone oxidoreductase, NADH:ubiquinone oxidoreductase complex, NADH Ubiquinone Reductase, reduced nicotinamide adenine dinucleotide-coenzyme Q reductase, Respiratory Chain Complex I, respiratory complex I, type 1 dehydrogenase, ubiquinone, ubiquinone reductase
MMP+	1-methyl-4-phenylpyridin-1-ium, 1-methyl-4-phenylpyridine, [3H]MPP+, 48134-75-4, C12H12N+, cyperquat, MPP+, N-methyl-4-phenylpyridine, N-methyl-4-phenylpyridinium, pyridinium, 1-methyl-4-phenyl-, pyridinium, 1-methyl-4-phenyl- (9CI)
MPTP	1,2,3,6-tetrahydro-1-methyl-4-phenylpyridine, 1-methyl-4-phenyl-3,6-dihydro-2H-pyridine, 28289-54-5, C12H15N, MPTP, pyridine, 1,2,3,6-tetrahydro-1-methyl-4-phenyl-
MT-CO1	Co1, COI, Complex IV Mitochondrial Subunit I, COX1, COX-I, cytochrome c oxidase I, Cytochrome C Oxidase Subunit 1, cytochrome c oxidase subunit I, MT-COI, X57780
MT-CO2	CIV-II, Co2, COII, COX2, COX II, CYTC OXIDASE II, cytochrome c oxidase subunit II, CYTOCHROME OXIDASE subunit 2, CytOX II, Pil7
MT-CO3	COIII, COX3, COX III, CY3, Cytochrome C Oxidase Subunit 3, cytochrome c oxidase subunit III, CytOX III, Mitochondrial cytochrome oxidase III, Similar to bcdo
MT-CYB	CYTB, cytochrome b, MITOCHONDRIAL CYTOCHROME B, mt-Cytb, mt-Cytb-201, mt-Cytb-NP 904340, Similar to cytochrome b
MT-ND4	NADH dehydrogenase subunit 4, Nadh ubiquinone oxidoreductase chain 4, ND4
MT-ND4L	NADH dehydrogenase subunit 4L, ND4L
MT-ND5	0610010I05RIK, Nadh5, NADH dehydrogenase subunit 5, ND5
Myxothiazol	2,6-heptadienamide, 7-(2'-(1,6-dimethyl-2,4-heptadienyl)(2,4'-bithiazol)-4-yl)-3,5-dimethoxy-4-methyl-, 2,6-heptadienamide, 7-(2'-((1S,2E,4E)-1,6-dimethyl-2,4-heptadienyl)(2,4'-bithiazol)-4-yl)-3,5-dimethoxy-4-methyl-, (2E,4R,5S,6E)-, (2E,4R,5S,6E)-3,5-dimethoxy-4-methyl-7-[2-[2-[(3E,5E)-7-methylocta-3,5-dien-2-yl]-1,3-thiazol-4-yl]-1,3-thiazol-4-yl]hepta-2,6-dienamide, 76706-55-3, C25H33N3O3S2
NAD+	[[(2R,3S,4R,5R)-5-(6-aminopurin-9-yl)-3,4-dihydroxyoxolan-2-yl]methoxy-hydroxyphosphoryl] [(2R,3S,4R,5R)-5-(3-carbamoylpyridin-1-ium-1-yl)-3,4-dihydroxyoxolan-2-yl]methyl hydrogen phosphate, 53-84-9, adenosine 5'-(trihydrogen diphosphate), P'-5'-ester with 3-(aminocarbonyl)-1-beta-D-ribofuranosylpyridinium, inner salt, adenosine 5'-(trihydrogen diphosphate), P'-5'-ester with 3-(aminocarbonyl)-1-β-D-ribofuranosylpyridinium, inner salt, beta-NAD+, beta-nicotinamide adenine dinucleotide+, C21H28N7O14P2+, NAD, β-NAD+, β-nicotinamide adenine dinucleotide+
NADH	[[(2R,3S,4R,5R)-5-(6-aminopurin-9-yl)-3,4-dihydroxyoxolan-2-yl]methoxy-hydroxyphosphoryl] [(2R,3S,4R,5R)-5-(3-carbamoyl-4H-pyridin-1-yl)-3,4-dihydroxyoxolan-2-yl]methyl hydrogen phosphate, 58-68-4, 606-68-8, adenosine 5'-(trihydrogen diphosphate), P'-5'-ester with 1,4-dihydro-1-beta-D-ribofuranosyl-3-pyridinecarboxamide, adenosine 5'-(trihydrogen diphosphate), P'-5'-ester with 1,4-dihydro-1-β-D-ribofuranosyl-3-pyridinecarboxamide, beta-NADH, C21H29N7O14P2, dihydronicotinamide-adenine dinucleotide, dihydronicotinamide mononucleotide, NADH2, nicotinamide dinucleotide, β-NADH
NADPH	2646-71-1, [[(2R,3R,4R,5R)-5-(6-aminopurin-9-yl)-3-hydroxy-4-phosphonooxyoxolan-2-yl]methoxy-hydroxyphosphoryl] [(2R,3S,4R,5R)-5-(3-carbamoyl-4H-pyridin-1-yl)-3,4-dihydroxyoxolan-2-yl]methyl hydrogen phosphate, 53-57-6, adenosine 5'-(trihydrogen diphosphate), 2'-(dihydrogen phosphate), P'-5'-ester with 1,4-dihydro-1-beta-D-ribofuranosyl-3-pyridinecarboxamide, adenosine 5'-(trihydrogen diphosphate), 2'-(dihydrogen phosphate), P'-5'-ester with 1,4-dihydro-1-β-D-ribofuranosyl-3-pyridinecarboxamide, C21H30N7O17P3, dihydronicotinamide-adenine dinucleotide phosphate, NADPH tetrasodium salt
NCSTN	9430068N19Rik, AA727311, APH2, ATAG1874, D1Dau13, D1Dau13e, Kiaa0253, mKIAA0253, Nc, NCT, ni, NICASTRIN

Symbol	Synonym(s)
NDUFA10	2900053E13Rik, CI-42k, CI-42KD, Complex I-42KD, LOC101927147, MC1DN22, NADH dehydrogenase (ubiquinone) 1 alpha subcomplex 10-like 1, Nadh dehydrogenase [ubiquinone] 1 alpha subcomplex subunit 10, NADH dehydrogenase (ubiquinone) 1 α subcomplex 10-like 1, Nadh dehydrogenase [ubiquinone] 1 α subcomplex subunit 10, NADH:ubiquinone oxidoreductase subunit A10, ndufa10l, Ndufa10l1
NDUFA11	2010012C24Rik, AV006275, B14.7, CI-B14.7, Complex I-B14.7, EG239760, Gm4943, MC1DN14, NADH:ubiquinone oxidoreductase subunit A11, NADH:ubiquinone oxidoreductase subunit A11B, Ndufa11b, ZINC FINGER
NDUFA12	2410011G03RIK, AW112974, B17.2, CI-B17.2, DAP13, MC1DN23, NADH-ubiquinone oxidoreductase b17.2, NADH:ubiquinone oxidoreductase subunit A12, RGD1311462
NDUFA13	2700054G14Rik, AU022060, B16.6, CDA01, CDA016, CGI-3, CGI-39, CI-B16.6, GRIM-, GRIM-19, MC1DN28, NADH:ubiquinone oxidoreductase subunit A13, NADH:ubiquinone oxidoreductase subunit A13, pseudogene 1, Ndufa13-ps, Ndufa13-ps1, RGD1565358
NDUFA2	AV000592, B8, C1-B8, CD14, CI-B8, Complex I-B8, MC1DN13, Nadh dehydrogenase(ubiquinone) 1 alpha subcomplex 7, Nadh dehydrogenase(ubiquinone) 1 subcomplex 2, Nadh dehydrogenase(ubiquinone) 1 α subcomplex 7, NADH:ubiquinone oxidoreductase subunit A2
NDUFA3	1010001M12Rik, 1700022J01Rik, AC012314.1, AC012314.11, AC012314.13, AC012314.16, AC012314.18, AC012314.5, AC012314.8, AC012314.9, B9, CI-B9, LOC683547, LOC684509, NADH:ubiquinone oxidoreductase subunit A3, similar to NADH-ubiquinone oxidoreductase B9 subunit (Complex I-B9) (CI-B9)
NDUFA4	CI-9k, CI-MLRQ, Complex I-MLRQ, COXFA4, LOC679503, LOC681024, MC4DN21, ML, MLRQ, Nadh dehydrogenase (ubiquinone) 1 alpha subcomplex 4, Nadh dehydrogenase (ubiquinone) 1 α subcomplex 4, NDUA4, NDUFA4 mitochondrial complex associated, NDUFA4, mitochondrial complex associated
NDUFA4L2	BC064011, COXFA4L2, LOC56901, NDUFA4 mitochondrial complex associated like 2, NDUFA4, mitochondrial complex associated like 2, NUOMS
NDUFA5	2900002J19Rik, B13, CI-13kB, CI-13KD-B, Complex i 13 kda subunit, NADH:ubiquinone oxidoreductase subunit A5, NADHUO, NUFM, UQOR13
NDUFA6	2700038D15Rik, B14, B230217P19Rik, CI-B14, LYRM6, MC1DN33, NADHB14, NADH DEHYDROGENASE 1 alpha, NADH DEHYDROGENASE 1 α, NADH:ubiquinone oxidoreductase subunit A6
NDUFA7	14.5kD, 14.5kDa, 2400007M02Rik, B14.5a, CI-B14.5a, NADH:ubiquinone oxidoreductase subunit A7
NDUFA8	0610033L03Rik, Aa2-258, AW261656, CI-19KD, CI-PGIV, Complex I-19KD, Complex I-PGIV, NADH:ubiquinone oxidoreductase subunit A8, PGIV
NDUFA9	1010001N11Rik, 39 Kda, ci, CC6, CI-39k, Complex I-39KD, COQ11, MC1DN26, Nadh-ubiquinone oxidoreductase 39-kda subunit, NADH:ubiquinone oxidoreductase subunit A9, NADH UBIQUINONE OXYDOREDUCTASE, ND39, NDUFS2L, SDR22E1
NDUFAB1	2210401F17Rik, 2310039H15Rik, 2610003B19Rik, 9130423F15Rik, ACP, ACP1, CI-SDAP, FASN2A, Gm17257, NADH:ubiquinone oxidoreductase subunit AB1, NADH:ubiquinone oxidoreductase subunit AB1b, Ndufab1-ps, SDAP
NDUFAF1	2410001M24Rik, CGI-6, CGI-65, CIA, CIA30, MC1DN11, NADH:ubiquinone oxidoreductase complex assembly factor 1
NDUFB1	AC121965.1, CI-MNLL, CI-SGDH, Gm2695, LOC100046667, MNLL, NADH:ubiquinone oxidoreductase subunit B1, Ndufb1-ps
NDUFB10	0610011B04Rik, Complex I-PDSW, LOC681867, MC1DN35, NADH:ubiquinone oxidoreductase subunit B10, PDSW
NDUFB11	CI-ESSS, D5Bwg0566e, D5Bwg0577e, ESSS, MC1DN30, NADH:ubiquinone oxidoreductase subunit B11, Neuronal protein 15.6, Np, Np15, NP15.6, NP17.3, p15.6, P17.3, RGD1563698
NDUFB2	1810011O01Rik, AGGG, AI325567, CI-AGGG, LOC100503140, NADH:ubiquinone oxidoreductase subunit B2
NDUFB3	2700033I16Rik, B12, CI-B12, MC1DN25, NADH:ubiquinone oxidoreductase subunit B3
NDUFB4	0610006N12Rik, 1300010H20Rik, B15, CI-B15, Complex I-B15, Gm3244, Gm3873, LOC100361934, LOC687888, LOC691675, LOC727762, NADH dehydrogenase (ubiquinone) 1 beta subcomplex 4, NADH dehydrogenase (ubiquinone) 1 beta subcomplex, 4-like 1, pseudogene 1, NADH dehydrogenase (ubiquinone) 1 β subcomplex 4, NADH dehydrogenase (ubiquinone) 1 β subcomplex, 4-like 1, pseudogene 1, NADH:ubiquinone oxidoreductase subunit B4, NADH:ubiquinone oxidoreductase subunit B4B, NADH:ubiquinone oxidoreductase subunit B4C, Ndufb4b, Ndufb4c, Ndufb4l1, Ndufb4l1-ps1, RGD1560088, RGD1560413, similar to NADH dehydrogenase (ubiquinone) 1 beta subcomplex, 4, 15kDa, similar to NADH dehydrogenase (ubiquinone) 1 β subcomplex, 4, 15kDa, similar to NADH:ubiquinone oxidoreductase B15 subunit
NDUFB5	0610007D05Rik, AU015782, CI-SGDH, Complex I-SGDH, Nadh, NADH:ubiquinone oxidoreductase subunit B5, Nadufb5, SGDH
NDUFB6	17 Kda, ci, B17, CI, CI-B17, DD6C4-3, DD6C4-4, Gm137, NADH UBIQUINONE OXIDOREDUCTASE B17, NADH:ubiquinone oxidoreductase subunit B6
NDUFB7	1110002H15Rik, B18, CI-B18, NADH DEHYDROGENASE (UBIQUINONE) 1 beta SUBCOMPLEX, 7, 18KDA, NADH DEHYDROGENASE (UBIQUINONE) 1 β SUBCOMPLEX, 7, 18KDA, NADH:ubiquinone oxidoreductase subunit B7
NDUFB8	2900010I05Rik, AI987932, ASHI, CI-ASHI, Complex-I Ubiquinone Oxidoreductase Subunit Ashi, MC1DN32, NADH:ubiquinone oxidoreductase subunit B8
NDUFB9	1190008J14Rik, B22, CI-B22, Complex I-B22, LYRM3, MC1DN24, NADH Dehydrogenase (Ubiquinone) 1 Beta B22 Subunit, NADH Dehydrogenase (Ubiquinone) 1 β B22 Subunit, Nadh-Q Oxidoreductase B22, Nadh Ubiquinone Oxidoreductase B22 Subunit, NADH:ubiquinone oxidoreductase subunit B9, NDUB9, UQOR22
NDUFS1	5830412M15RIK, 9930026A05Rik, CI-75k, CI-75Kd, MC1DN5, MITOCHONDRIAL COMPLEX I 75-KDA subunit, NADH-coenzyme Q reductase, NADH dehydrogenase precursor, 75kDa subunit, Nadh Ubiquinone Oxidoreductase 75 Kda Subunit, NADH-ubiquinone oxidoreductase 75 kDa subunit, mitochondrial, NADH:ubiquinone oxidoreductase core subunit S1, PRO1304
NDUFS2	CI-49, CI-49kD, Complex I-49KD, MC1DN6, Nadh-coenzyme q reductase, Nadh Ubiquinone Oxidoreductase 49 Kda Subunit, NADH:ubiquinone oxidoreductase core subunit S2
NDUFS3	0610010M09Rik, 30 Kda, ci, CI-30, CI-30kD, MC1DN8, NADH Dehydrogenase 30kDa, NADH:ubiquinone oxidoreductase core subunit S3, NADH-UBIQUINONE REDUCTASE
NDUFS4	18 kda subunit of complex i, 6720411N02RIK, AQDQ, C1-18k, CI-18, CI-18 kDa, CI-AQDQ, Complex I-18 kDa, MC1DN1, NADH:ubiquinone oxidoreductase core subunit S4, NADH:ubiquinone oxidoreductase subunit S4
NDUFS5	AA407369, AI256693, CI-15k, NADH:ubiquinone oxidoreductase subunit S5, Ndufs5b
NDUFS6	BC059730, CI-13kA, CI-13kD-A, EG623286, Gm6415, IP, IP13, Ip13dis, LOC100912599, LOC679739, MC1DN9, NADH dehydrogenase (ubiquinone) Fe-S protein 6, NADH dehydrogenase [ubiquinone] iron-sulfur protein 6, mitochondrial-like, NADH dehydrogenase [ubiquinone] iron-sulphur protein 6, mitochondrial-like, Nadh ubiquinone oxidoreductase 13 kda a subunit, NADH:ubiquinone oxidoreductase core subunit S6, NADH:ubiquinone oxidoreductase subunit S6, NADH:ubiquinone oxidoreductase subunit S6B, Ndub13, Ndufs6b, RATIp13dis
NDUFS7	1010001M04Rik, CI-20, CI-20KD, MC1DN3, MY017, NADH-coenzyme Q reductase, NADH:ubiquinone oxidoreductase core subunit S7, PSST
NDUFS8	BC021616, CI-23k, CI-23kD, MC1DN2, NADH dehydrogenase (ubiquinone) Fe-S protein 8, NADH:ubiquinone oxidoreductase core subunit S8, TYKY
NDUFV1	CI-51K, CI-51kD, MC1DN4, MITOCHONDRIAL COMPLEX I-51KDA subunit, NADH DEHYDROGENASE 51KD, NADH:ubiquinone oxidoreductase core subunit V1, UQOR1
NDUFV2	24KDA subunit-MITOCHONDRIAL COMPLEX 1, 2900010C23Rik, CI-24k, MC1DN7, NADH Dehydrogenase (Ubiquinone) Flavoprotein 2, NADH:ubiquinone oxidoreductase core subunit V2
NDUFV3	1500032D16RIK, CI-10k, CI-9KD, Mipp65, MITOCHONDRIAL COMPLEX I-10KDA subunit, NADH:ubiquinone oxidoreductase core subunit V3, NADH:ubiquinone oxidoreductase subunit V3, Ndufv3l
nicotinamide adenine dinucleotide phosphate	1184-16-3, [[(2R,3R,4R,5R)-5-(6-aminopurin-9-yl)-3-hydroxy-4-phosphonooxyoxolan-2-yl]methoxy-hydroxyphosphoryl] [(2R,3S,4R,5R)-5-(3-carbamoylpyridin-1-ium-1-yl)-3,4-dihydroxyoxolan-2-yl]methyl hydrogen phosphate, 53-59-8, adenosine 5'-(trihydrogen diphosphate), 2'-(dihydrogen phosphate), P'-5'-ester with 3-(aminocarbonyl)-1-beta-D-ribofuranosylpyridinium, inner salt, adenosine 5'-(trihydrogen diphosphate), 2'-(dihydrogen phosphate), P'-5'-ester with 3-(aminocarbonyl)-1-β-D-ribofuranosylpyridinium, inner salt, beta-NADP, C21H29N7O17P3+, NADP, NAD(P), NAD(P)+, NADP+, NAD phosphate, β-NADP
O2-	11062-77-4, O2-, oxygen peroxide, superoxide, superoxide anion, superoxide ion, superoxide radical
OGDH	2210403E04RIK, 2210412K19Rik, AA409584, AKGDH, Alpha-ketoglutarate dehydrogenase, d1401, E1k, Kdh E1, KGD1, KGDH, LOC103693780, mKIAA4192, ODO1, OGDC, OGDH2, oxoglutarate (alpha-ketoglutarate) dehydrogenase (lipoamide), oxoglutarate dehydrogenase, oxoglutarate (α-ketoglutarate) dehydrogenase (lipoamide), α-ketoglutarate dehydrogenase
Oligomycin	1404-19-9, oligomycin
Palmitate	143-20-4, 16:0 fatty acid, 57-10-3, C16:0, C16:0-COOH, C16:0 fatty acid, C16:0 free fatty acid, c16 fatty acid, C16H32O2, cetylic acid, hexadecanoate, hexadecanoic acid, hexadecylic acid, n-hexadecanoic acid, n-palmitic acid, palmitate, palmitate acid, sodium palmitate
PARK7	CAP1, DJ-1, GATD2, HEL-S-67p, Parkinson disease (autosomal recessive, early onset) 7, Parkinsonism associated deglycase, SP22
PDHA1	E1 alpha PDH, E1 α PDH, PDHA, PDHAD, PDHCE1A, PDHE1a, PDHE1alpha, PDHE1α, PHE1A, Pyruvate dehydrogenase-alpha, Pyruvate dehydrogenase e1 alpha, Pyruvate dehydrogenase e1 alpha1, pyruvate dehydrogenase E1 subunit alpha 1, pyruvate dehydrogenase E1 subunit α 1, Pyruvate dehydrogenase e1 α, pyruvate dehydrogenase E1 α 1, Pyruvate dehydrogenase-α
Perhexiline	2-(2,2-dicyclohexylethyl)piperidine, 6621-47-2, C19H35N, perhexiline maleate, pherexiline, piperidine, 2-(2,2-dicyclohexylethyl)-
peroxynitrite	19059-14-4, NO3-, oxido nitrite, peroxinitrite, peroxynitrile, peroxynitrite, sodium peroxynitrite
PINK1	1190006F07Rik, AU042772, AW557854, br, BRPK, mFLJ00387, PARK6, PTEN-induced kinase, PTEN-induced kinase 1, PTEN induced putative kinase 1
PRDX3	A, AOP-1, AW822249, D0Toh, D0Tohi1, Ef2, Ef2l, HBC189, Mer, MER5, MTAP, peroxiredoxin 3, PRO1748, Prx3, PRX III, SP, SP-22, TDXM, THIOREDUCTASE DEPENDANT PEROXIDE REDUCTASE
PRDX5	ACR1, AOEB1, AOEB166, AOPP, B166, HEL-S-55, P, Peroxiredoxin 5, PLP, PMP, PMP20, Prd, PRDX6, PRX5, PRXV, SBBI10, THIOREDOXIN PEROXIDASE 5
PRKN	AR-JP, LPRS2, P, Pa, Park, PARK2, Parkin, parkin RBR E3 ubiquitin protein ligase, PDJ, Pk
PSEN1	ACNINV3, AD3, Ad3h, FAD, P, presenilin 1, PS, PS-1, S18, S182
PSENEN	1700023M09RIK, ACNINV2, MDS033, MSTP064, PEN-2, presenilin enhancer gamma secretase subunit, presenilin enhancer, gamma-secretase subunit, presenilin enhancer γ secretase subunit, presenilin enhancer, γ-secretase subunit, RGD1312037
RHOT2	Arh, ARHT2, C16orf39, Mi, MIRO-2, ras homolog family member T2, RASL
ROS	oxygen and reactive oxygen species, reactive oxygen metabolites, ROI, ROS
Rotenone	(1)Benzopyrano(3,4-b)furo(2,3-h)(1)benzopyran-6(6aH)-one, 1,2,12,12a-tetrahydro-8,9-dimethoxy-2-(1-methylethenyl)-, (2R-(2alpha,6aalpha,12aalpha))-, (1S,6R,13S)-16,17-dimethoxy-6-prop-1-en-2-yl-2,7,20-trioxapentacyclo[11.8.0.03,11.04,8.014,19]henicosa-3(11),4(8),9,14,16,18-hexaen-12-one, (2R,6aS,12aS)-1,2,6,6a,12,12a-hexahydro-2-isopropenyl-8,9-dimethoxychromeno(3,4-b)furo(2,3-h)chromen-6-one, 83-79-4, C23H22O6, rot

Symbol	Synonym(s)
SDHA	1500032O14RIK, 2310034D06Rik, 4921513A11, C81073, CMD1GG, Complex II Flavoprotein Subunit, Electron-transfer-flavoprotein, alpha polypeptide, Electron-transfer-flavoprotein, α polypeptide, F, FP, MC2DN1, PGL5, SDH, SDH1, SDH2, SDH70, SDHF, succinate dehydrogenase complex flavoprotein subunit A, Succinate dehydrogenase complex, subunit A flavoprotein (Fp), succinate dehydrogenase complex, subunit A, flavoprotein (Fp), Succinate-ubiquinone oxidoreductase 70-kda subunit
SDHB	0710008N11Rik, CII-30, CII - 30 (Fe S), CWS2, Electron-transfer-flavoprotein, beta polypeptide, Electron-transfer-flavoprotein, β polypeptide, IP, PCHC, PGL4, SDH, SDH1, SDH2, SDH30, SDHIP, succinate dehydrogenase complex iron sulfur subunit B, succinate dehydrogenase complex iron sulphur subunit B, succinate dehydrogenase complex, subunit B, iron sulfur (Ip), succinate dehydrogenase complex, subunit B, iron sulphur (Ip), Succinate Dehydrogenase Cytochrome B Subunit, Succinate Dehydrogenase Ip Cytochrome B Subunit, Succinate dehydrogenase putative iron sulfur subunit, Succinate dehydrogenase putative iron sulphur subunit
SDHC	0610010E03Rik, AI316496, AU019277, CYB560, CYBL, PGL3, QPs-1, SDH3, succinate dehydrogenase complex subunit C, succinate dehydrogenase complex, subunit C, integral membrane protein
SDHD	3110001M13RIK, AVLL5809, C78570, CBT1, CII-4, CWS3, cybS, MC2DN3, PGL, PGL1, PRO19626, QPs3, SDH4, SDHD1, succinate dehydrogenase complex subunit D, succinate dehydrogenase complex, subunit D, integral membrane protein
SNCA	AD AMYLOID, al, alp, alphaSYN, alpha SYNUCLEIN, ASYN, NACP, PARK1, PARK4, PD1, synuclein alpha, synuclein, alpha, Synuclein-α, synuclein, α, α-Syn, α SYNUCLEIN
SOD2	GClnc1, IMAGE:4711494, IPO-B, MANGANESE DEPENDENT SOD, Manganese Superoxide Dismutase, Manganese Superoxide Dismutase 2, MGC5618, MITOCHONDRIAL SOD, Mn, MNSOD, Mn superoxide dismutase, mtSOD, MVCD6, Sod, Superoxide dismutase, superoxide dismutase 2, superoxide dismutase 2, mitochondrial
Succinatedeh ydrogenase	1.3.99.1, Complex II, fumarate reductase, fumaric hydrogenase, Oxphos Complex II, succinate:(acceptor) oxidoreductase, Succinate INT Dehydrogenase, succinate oxidoreductase, succinic acid dehydrogenase, succinic dehydrogenase, succinodehydrogenase, succinyl dehydrogenase, SUO
TRAK1	2310001H13RIK, AI413908, AI467545, DEE68, EIEE68, hyr, hyrt, KIAA1042, MILT1, mKIAA1042, OIP106, RGD1307844, trafficking kinesin protein 1, trafficking protein, kinesin binding 1
TXN2	2510006J11Rik, AI788873, COXPD29, MTRX, MT-TRX, thioredoxin 2, Trx, TRX2, TXN
TXNRD2	AA118373, ESTM57301, ESTM573010, GCCD5, SELZ, TG, TGR, thioredoxin reductase 2, TR, TR3, TR-BETA, TRXR2, Trxrd2, TR-β
UCP2	BMIQ4, SLC25A8, UCPH, uncoupling protein 2, Uncoupling protein 2, mitochondrial, uncoupling protein 2 (mitochondrial, proton carrier)
UQCR10	1110020P15Rik, AA960494, HSPC051, HSPC119, HSPC151, LOC683838, LOC685322, QCR9, Ubiquinol Cytochrome C Reductase 7.2 kd, ubiquinol-cytochrome c reductase, complex III subunit X, UCCR7.2, UCRC
UQCR11	0710008D09Rik, AL022707, LOC686951, QCR10, Ubiquinol cytochrome c reductase 6.4kd, ubiquinol-cytochrome c reductase, complex III subunit XI, Uqc, UQCR
UQCRB	2210415M14Rik, MC3DN3, QCR7, QP-, QP-C, ubiquinol-cytochrome c reductase binding protein, UQBC, UQBP, UQCR6, Uqcrbl, UQPC
UQCRC1	1110032G10Rik, COR1, D3S3191, QCR1, Ubiquinol cytochrome c reductase 1, Ubiquinol Cytochrome C Reductase Core 1, ubiquinol-cytochrome c reductase core protein 1, UQCR1
UQCRC2	1500004O06Rik, AURA11, Core 2, CORE protein 2 of COMPLEX III, Cytochrome b-c1 complex subunit 2, MC3DN5, MGC94368, Mitochondrial Core Protein2, QCR2, Rikubiquinol Cytochrome C Reductase Core Protein 2, ubiquinol-cytochrome c reductase core protein 2, Ubiquinol Cytochrome C Reductase Core Protein 2 Precursor, UQCR2
UQCRFS1	4430402G14RIK, AI875505, Complex III FeS, Fes subunit of complex iii, ISP, LRRGT00195, MC3DN10, Rieske, RIP1, RIS1, RISP, UBIQUINOL CYTOCHROME C REDUCTASE, ubiquinol-cytochrome c reductase, Rieske iron-sulfur polypeptide 1, ubiquinol-cytochrome c reductase, Rieske iron-sulphur polypeptide 1, UQCR5
UQCRH	2210416J04Rik, 2310021J10Rik, 2610041P16Rik, Gm9763, LOC100046686, QCR6, ubiquinol-cytochrome c reductase hinge protein, ubiquinol-cytochrome c reductase hinge protein, pseudogene 1, UQCR8, Uqcrh-ps1
XDH	X, XAN1, Xanthine Dehydrogenase, Xanthine Oxidase, XO, XOR, Xox-, Xox-1

Pathway Analysis Using IPA Software; canonical pathway

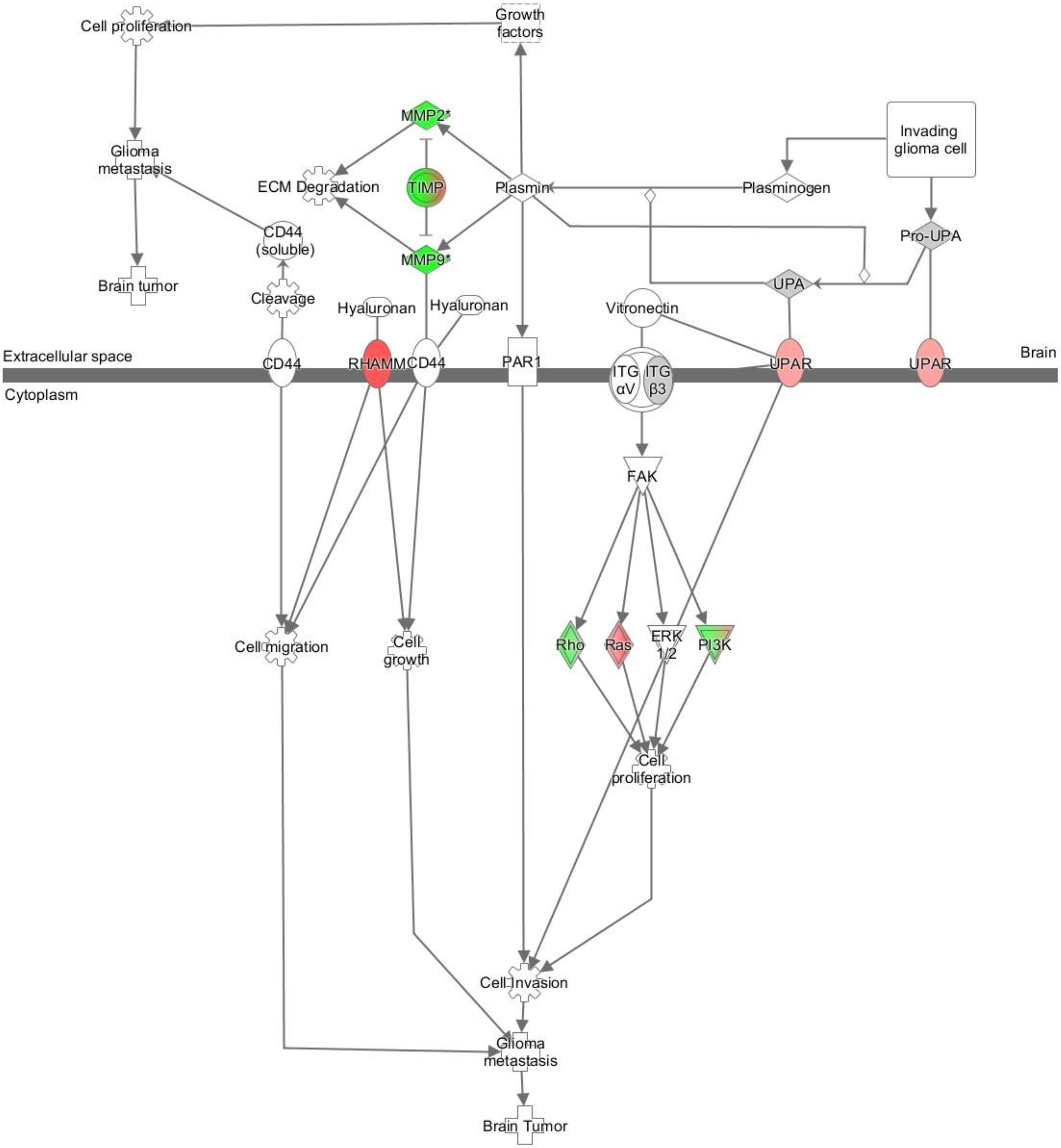

Figure S54. Glioma Invasiveness Signaling at 24 h

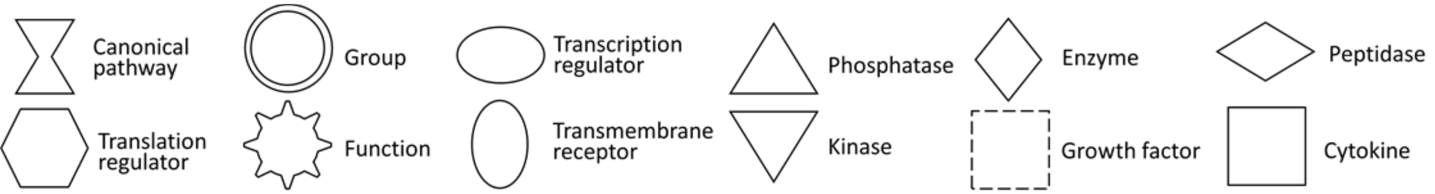

Red: Increased, FDR<0.05 versus solvent control

Green: Decreased, FDR<0.05 versus solvent control

Symbol	Synonym(s)
CD44	216062 AT, AU023126, AW121933, AW146109, CD44A, CD44 Antigen, CD44 (containing exon 5), Cd44i, CD44 molecule (Indian blood group), CD44 (soluble), CD44 STANDARD FROM, CDW44, CSPG8, ECMR-III, Epican, HCELL, HERM, HERMES, Hermes antigen, HUTCH-I, IN, LHR, Ly-2, Ly-24, MC56, MDU2, MDU3, METAA, MIC4, NKT.44, Pgp, Pgp-1, RHAMM
ERK1/2	MAPK p44/42, MAPK p44/p42, p42/44 mapk, P42/p44 erk, P42/p44 mapk, p42/p44 MAP KINASE
F2R	AI482343, Cf2, CF2R, coagulation factor II (thrombin) receptor, coagulation factor II thrombin receptor, HTR, P, PAR-1, Th, ThrR, TR, TRGPC, α Thrombin Receptor
HMMR	AA386826, CD168, hyaluronan-mediated motility receptor, hyaluronan mediated motility receptor (RHAMM), IHABP, Rha, RHAMM
Hyaluronan	34448-35-6, 9004-61-9, 9067-32-7, Amvisc, Amvisc Plus, Biolon, C28H44N2NaO23+, Duovisc, etamucine, EUFLEXXA, HA, Healon, Healon5, Hyalgan, hyaluronan, hyaluronan acid, hyaluronate, hyaluronate sodium, hyaluronic acid oligosaccharide, hyaluronic acid, sodium salt, Hyruan Plus, Hyvisc, luronit, Orthovisc, Provisc, sodium;(2S,3S,4S,5R,6R)-6-[(2S,3R,4R,5S,6R)-3-acetamido-2-[(2S,3S,4R,5R,6R)-6-[(2R,3R,4R,5S,6R)-3-acetamido-2,5-dihydroxy-6-(hydroxymethyl)oxan-4-yl]oxy-2-carboxy-4,5-dihydroxyoxan-3-yl]oxy-5-hydroxy-6-(hydroxymethyl)oxan-4-yl]oxy-3,4,5-trihydroxyoxane-2-carboxylic acid, sodium hyaluronate, Viscoat, Vitrax
Integrin alpha-V beta 3	alpha-v beta-3, alpha V beta 3 Integrin, Integrin-alpha-beta3, Integrin-α-beta3, Integrin α V beta3, Integrin α V β 3, Vitronectin Receptor, VnR, α-5-beta3, α 5 β 3, α-v β-3, α V β 3 Integrin
ITGAV	1110004F14RIK, 2610028E01Rik, alpha V, CD51, D430040G12RIK, integrin alpha V, integrin subunit alpha V, integrin subunit α V, Integrin α V, MSK8, VNRA, VTNR, α V
ITGB3	BDPLT16, BDPLT2, beta 3, CD61, GP3A, GPIIIa, GT, HPA-4, INGRB3, integrin beta 3, integrin subunit beta 3, integrin subunit β 3, Integrin-β 3, β 3
MMP2	Clg, CLG4, CLG4A, Ge, GelA, GELATINASE, Gelatinase A, matrix metallopeptidase 2, METALLOPROTEINASE 2, MMP-, MMP-II, MONA, TBE-1
MMP9	AW743869, B/MMP, B/MMP9, Clg4, CLG4B, COLLAGENASE type IV, Gelatinase B, GELB, GI 92-kda, MANDP2, matrix metallopeptidase 9, METALLOPROTEINASE 9, MMP-, pro-MMP-9
PI3K	1-phosphatidylinositol 3-kinase, 2.7.1.137, ATP:1-phosphatidyl-1D-myo-inositol 3-phosphotransferase, Phosphatidylinositol 3 kinase, phosphatidylinositol 3'-kinase, PI3-kinase, PtdIns 3 Kinase, type III phosphoinositide 3-kinase, type I phosphatidylinositol kinase, Vps34p
PLAU	ATF, BDPLT5, plasminogen activator, urokinase, Pro-UPA, QPD, u-, UPA, uPA 50 kd form, UPA-H, UPAM, URK
PLAUR	CD87, Par, plasminogen activator, urokinase receptor, Plaur3, u-, U-PAR, uPAR-2, UPAR-3, Urinary plasminogen activator receptor 2, URKR, UROKINASE R, Urokinase-type plasminogen activator receptor
PLG	Ab1-346, AI649309, GLU-PG, LPA, P, Pg, PG2, plasminogen, Scdp
PTK2	FA, Fad, FADK, FADK 1, FAK, FAK1, FAK related non-kinase, FR, p125FAK, pp125FAK, PPP1R71, protein tyrosine kinase 2, PTK2 protein tyrosine kinase 2, TYROSINE KINASE 2
Rho	GTPase Rho, Rho, Rho Family, RHO-GTPASE, Rho-like Gtpase
VTN	Aa1018, AI256434, V75, vitronectin, VN, VNT

Pathway Analysis Using IPA Software; canonical pathway

24 h

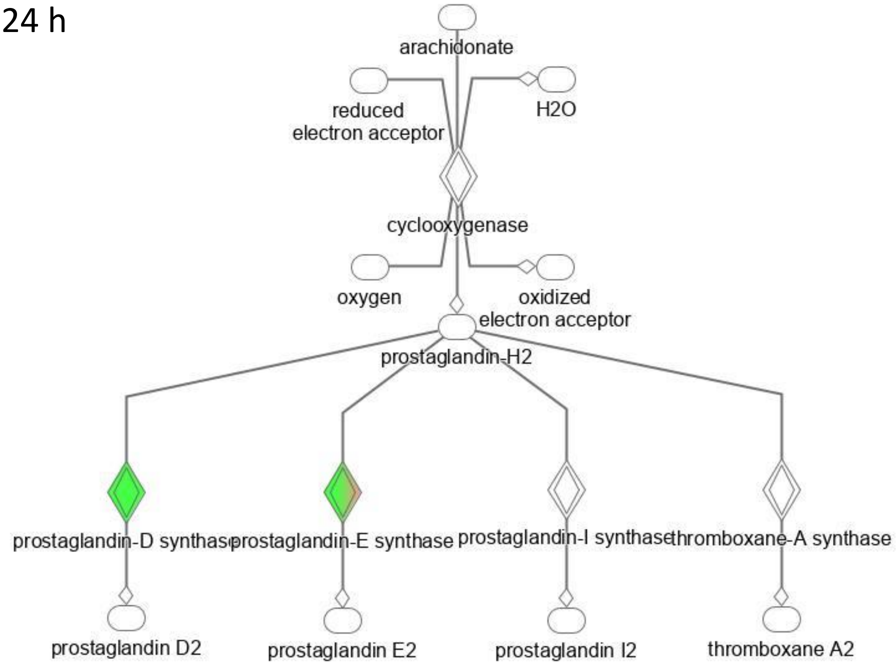

8 days

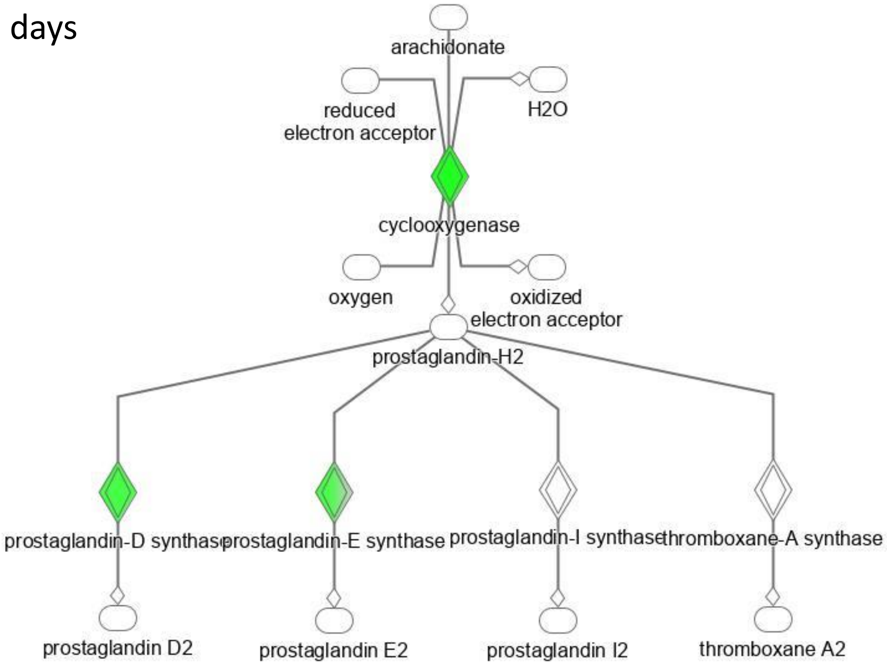

Figure S55. Prostanoid Biosynthesis at 24 h and 8 days

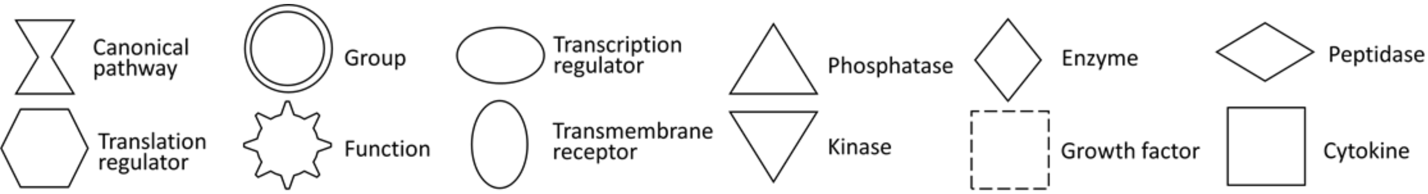

Red: Increased, FDR<0.05 versus solvent control

Green: Decreased, FDR<0.05 versus solvent control

Symbol	Synonym(s)
arachidonate	20:4n-6, 506-32-1, 5,8,11,14-eicosatetraenoic acid, (all-Z)-, 5Z,8Z,11Z,14Z-arachidonic acid, (5Z,8Z,11Z,14Z)-icosa-5,8,11,14-tetraenoic acid, AA, AA-d8, ARA, arachidonate, C20:4(n-6), C20:4w6, C20H32O2, eicosa-5Z,8Z,11Z,14Z-tetraenoic acid, sodium arachidonate
cyclooxygenase	1.14.99.1, (5Z,8Z,11Z,14Z)-icosa-5,8,11,14-tetraenoate,hydrogen-donor:oxygen oxidoreductase, COX, COX1/2, fatty acid cyclooxygenase, PGHS, (PG)H synthase, PG synthetase, Prostaglandin-endoperoxide synthase, prostaglandin endoperoxide synthetase, prostaglandin G/H synthase, prostaglandin G/H synthase and cyclooxygenase, Prostaglandin h synthase, Prostaglandin Peroxidase, prostaglandin synthase, prostaglandin synthetase, PTGS, PTGS1/2
prostaglandin D2	11-dehydroprostaglandin F2-alpha, 11-dehydroprostaglandin F2-α, 41598-07-6, (5Z,13E)-9alpha-hydroxy-11,15-dioxoprosta-5,13-dienoate, (5Z,13E)-9alpha-hydroxy-11,15-dioxoprosta-5,13-dienoic acid, C20H32O5, PGD2, prosta-5,13-dien-1-oic acid, 9,15-dihydroxy-11-oxo-, (5Z,9-alpha,13E,15S)-, prosta-5,13-dien-1-oic acid, 9,15-dihydroxy-11-oxo-, (5Z,9-α,13E,15S)-, (Z)-7-[(1R,2R,5S)-5-hydroxy-2-[(E,3S)-3-hydroxyoct-1-enyl]-3-oxocyclopentyl]hept-5-enoic acid
prostaglandin E2	363-24-6, (5Z, 11a, 13E, 15S)-11, 15-Dihydroxy-9-oxo-prosta-5, 13-dien-1-oic acid, C20H32O5, Cervidil, dinoprostone, PGE2, Prepidil, Propess, Prostarmon E, Prostin E, Prostin E2, Prostin E2 Vaginal Suppository, (Z)-7-[(1R,2R,3R)-3-hydroxy-2-[(E,3S)-3-hydroxyoct-1-enyl]-5-oxocyclopentyl]hept-5-enoic acid
prostaglandin I2	35121-78-9, (5Z)-5-[(3aR,4R,5R,6aS)-5-hydroxy-4-[(E,3S)-3-hydroxyoct-1-enyl]-3,3a,4,5,6,6a-hexahydrocyclopenta[b]furan-2-ylidene]pentanoic acid, 61849-14-7, C20H32O5, epoprostenol sodium, Flolan, PGI2, PGX, prosta-5,13-dien-1-oic acid, 6,9-epoxy-11,15-dihydroxy-, (5Z,9alpha,11alpha,13E,15S)-, prostacyclin, prostaglandin I, prostaglandin I2, sodium PGI2
prostaglandin-D synthase	(5,13)-(15S)-9alpha,11alpha-epidioxy-15-hydroxyprosta-5,13-dienoate D-isomerase, 5.3.99.2, PGH-PGD isomerase, prostaglandin-H2 D-isomerase, prostaglandin-R-prostaglandin D isomerase
prostaglandin-E synthase	5.3.99.3, (5Z,13E)-(15S)-9alpha,11alpha-epidioxy-15-hydroxyprosta-5,13-dienoate E-isomerase, endoperoxide isomerase, PGE2 isomerase, PGE isomerase, PGH-PGE isomerase, prostaglandin endoperoxide E2 isomerase, prostaglandin endoperoxide E isomerase, prostaglandin-H2 E-isomerase, prostaglandin H-E isomerase, prostaglandin R-prostaglandin E isomerase
prostaglandin-H2	42935-17-1, 9,11-epoxymethano-PGH2, C20H32O5, PGH2, PGH2 endoperoxide, prosta-5,13-dien-1-oic acid, 9,11-epidioxy-15-hydroxy-, (5Z,9alpha,11alpha,13E,15S)-, (Z)-7-[(1R,4S,5R,6R)-6-[(E,3S)-3-hydroxyoct-1-enyl]-2,3-dioxabicyclo[2.2.1]heptan-5-yl]hept-5-enoic acid
prostaglandin-I synthase	5.3.99.4, (5Z,13E)-(15S)-9alpha,11alpha-epidioxy-15-hydroxyprosta-5,13-dienoate 6-isomerase, PGI2 synthase, PGI2 synthetase, prostacycline synthetase, prostacyclin synthase, prostagladin I2 synthetase
thromboxane A2	57576-52-0, 5-heptenoic acid, 7-(3-(3-hydroxy-1-octenyl)-2,6-dioxabicyclo(3.1.1)hept-4-yl)-, (1S-(1alpha,3alpha,3R*),4beta(Z),5alpha)-, C20H32O5, thromboxa-5,13-dien-1-oic acid, 9,11-epoxy-15-hydroxy-, (5Z,9alpha,11alpha,13E,15S)-, TxA2, (Z)-7-[(1S,3R,4S,5S)-3-[(E,3S)-3-hydroxyoct-1-enyl]-2,6-dioxabicyclo[3.1.1]heptan-4-yl]hept-5-enoic acid
thromboxane-A synthase	5.3.99.5, (5Z,13E)-(15S)-9alpha,11alpha-epidioxy-15-hydroxyprosta-5,13-dienoate thromboxane-A2-isomerase, thromboxane synthase

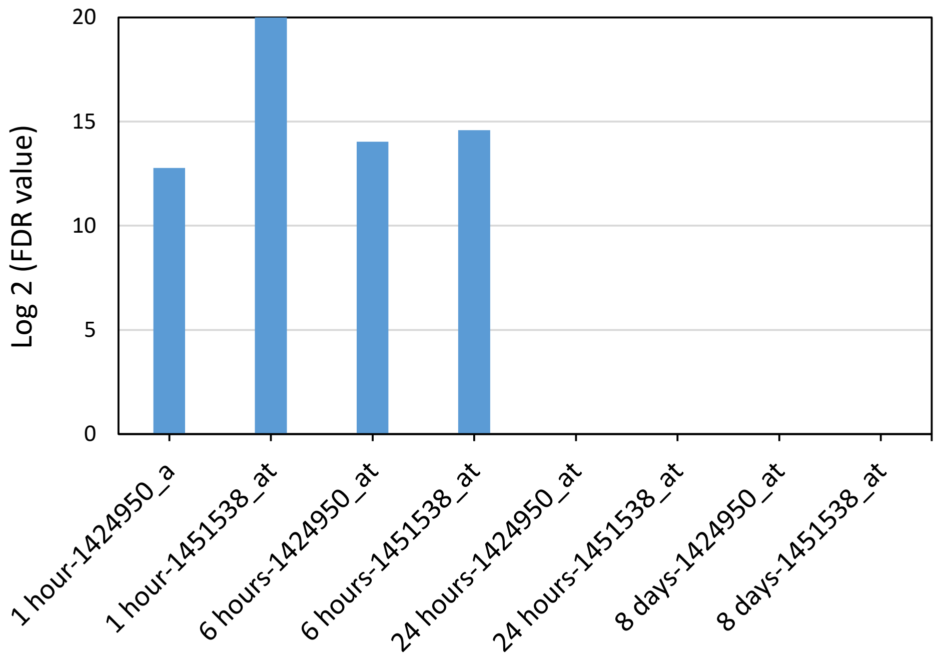

Figure S56. SOX9 gene expression

The FDRs that were up-regulated (FDR < 0.05) were transformed into logarithm (log 2). The transformed value with FDR of 0 was entered as 20 for up-regulation.

Pathway Analysis Using IPA Software; canonical pathway

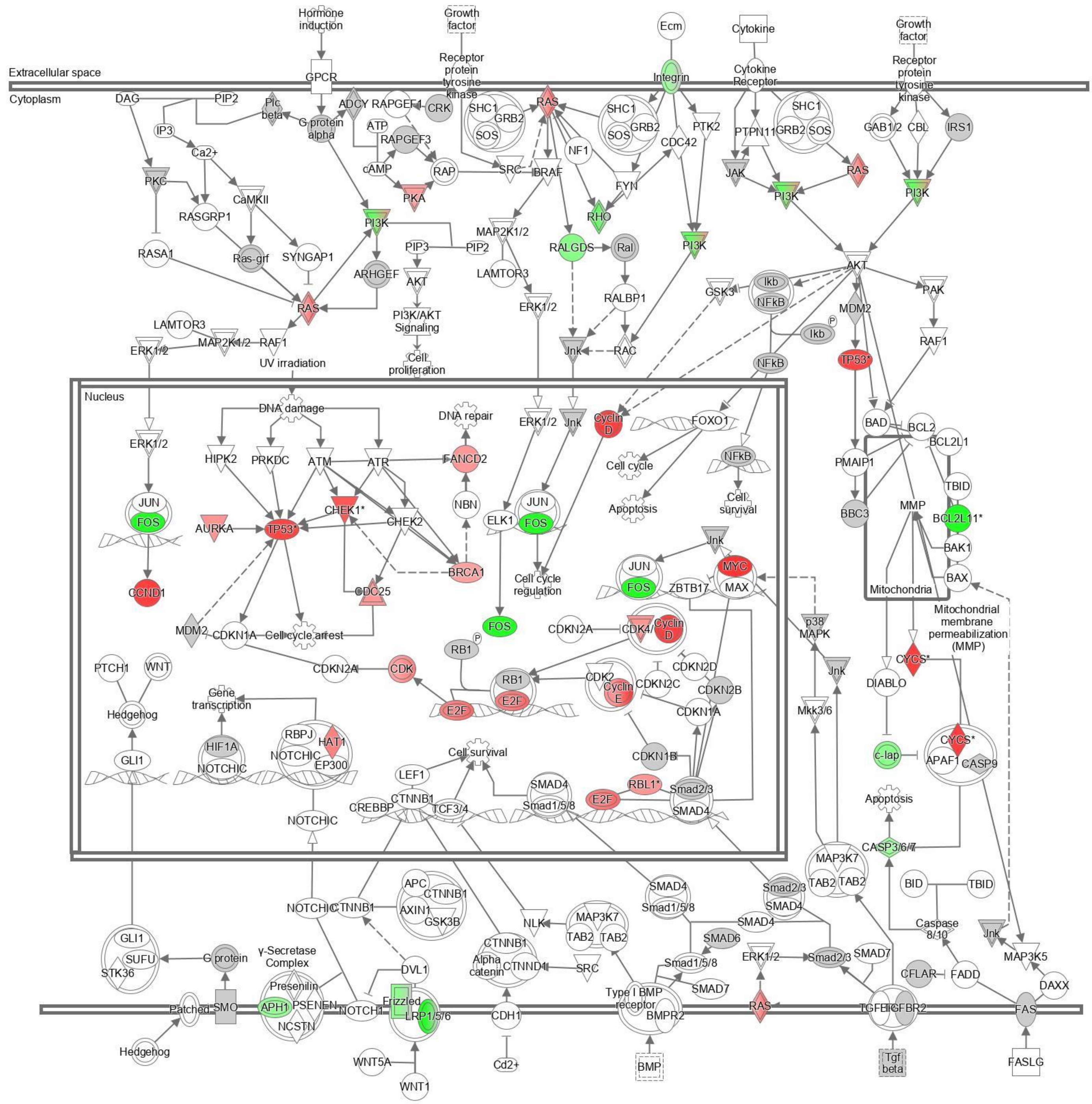

Figure S57. Molecular Mechanism of cancer at 24 h

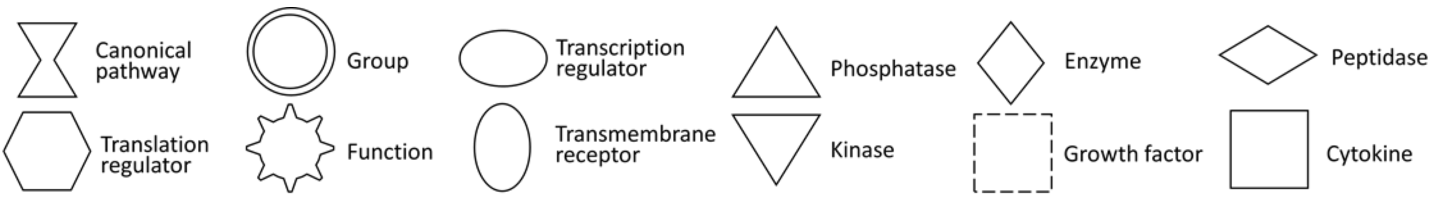

Red: Increased, FDR<0.05 versus solvent control

Green: Decreased, FDR<0.05 versus solvent control

Symbol	Synonym(s)
ADCY	3',5'-cyclic AMP synthetase, 4.6.1.1, AC, Adenylate Cyclase, Adenyl Cyclase, Adenylyl cyclase, ATP diphosphate-lyase (cyclizing), mAC, sAC
AKT	AKT1/2/3, B/Akt, PKB, RAC-PK
Alphacatenin	CTNN alpha, CTNN α, α catenin
Ap1	activator protein-1, c-Jun
APAF1	6230400I06RIK, Ap, Apaf1l, apoptotic peptidase activating factor 1, CED4, fog, mKIAA0413
APC	AI047805, APC1, Apc7, APC (PROC), APC regulator of WNT signaling pathway, APC, WNT signaling pathway regulator, AU020952, AW124434, BTPS2, CC1, DESMD, DP2, DP2.5, DP3, Familial adenomatous polyposis, GS, M, mAPC, Min, PPP1R46, RATAPC
Apoptosome	APAF1-Caspase 9-CytoC, apoptosis adaptor protein complex, Cytochrome C-APAF1-Caspase 9
ARHGEF	Ras GEF, RhOGEF
ATM	AI256621, AT1, ATA, ataxia telangiectasia mutated, ATC, ATD, ATDC, ATE, ATM serine/threonine kinase, C030026E19RIK, TEL1, TELO1
ATP	[[(2R,3S,4R,5R)-5-(6-aminopurin-9-yl)-3,4-dihydroxyoxolan-2-yl]methoxy-hydroxyphosphoryl] phosphono hydrogen phosphate, 56-65-5, 9-beta-D-arabinofuranosyladenine 5'-triphosphate, 9-β-D-arabinofuranosyladenine 5'-triphosphate, adenosine 5'-(tetrahydrogen triphosphate), adenosine 5'-triphosphate, ATP, ATP4-, C10H16N5O13P3
ATR	ataxia telangiectasia and Rad3 related, Ataxia-telangiectasia-like, ATR serine/threonine kinase, FCTCS, FRP1, LOC100365674, LOC367198, LOC684113, MEC1, SCKL, SCKL1
AURKA	AI, AIK, AIRK1, Ar, ARK-1, Au, AU019385, AURA, AURORA 2, AURORA A, AURORA KINASE, aurora kinase A, Aurora Related Kinase1, AW539821, Ayk, Ayk1, BTAK, I, IA, IAK, IAK1, PPP1R47, Stk, STK15, STK6, STK7
AXIN1	AI316800, AXIN, AXIN form I, Fu, fused, Kb, Ki, kinky, knobbly, PPP1R49
BAD	AI325008, Bad v1, Bad v2, BBC2, BCL2-associated agonist of cell death, BCL2L8
BAK1	Ba, BAK, BAK-LIKE, BCL2-antagonist/killer 1, BCL2L7, CDN1, N-B, N-BAK1
BAX	Bcl2-associated X, BCL2 associated X, apoptosis regulator, BCL2-associated X protein, BCL2L4
BBC3	BCL2 binding component 3, JFY-1, PU, PUMA, PUMA/JFY1
BCL2	AW986256, B cell leukaemia/lymphoma 2, B cell leukemia/lymphoma 2, Bcl-, Bcl2 alpha, BCL2 apoptosis regulator, BCL2, apoptosis regulator, Bcl2 α, C430015F12Rik, D630044D05RIK, D830018M01RIK, LOC100046608, ORF16, PPP1R50
BCL2L1	bBclxl, Bcl, BCL2L, BCL2-like 1, BCLX, Bcl-X beta, Bclx gamma, BCL-XL/S, Bcl-X β, Bclx γ, PPP1R52
BCL2L11	1500006F24RIK, BAM, BCL2 like 11, BCL2-like 11 (apoptosis facilitator), Bi, BIM, Bo, BOD, BODL, LOC150819
BMP	BMP3, BMP-3A, BONE MORPHOGENIC, Osteogenin
BMPR2	2610024H22RIK, AL117858, AW546137, BB189135, BM, BMP-, BMP-2, BMPR3, BMPR-II, BMR2, bone morphogenetic protein receptor type 2, bone morphogenetic protein receptor, type II (serine/threonine kinase), BRK-3, Gm20272, POVD1, PPH1, T-ALK, Type ii bmp receptor
BRAF	9930012E13RIK, AA120551, AA387315, AA473386, AI447469, Bra, B-RAF1, Braf-2, B-Raf proto-oncogene, serine/threonine kinase, Braf transforming gene, C230098H17, C87398, D6Ertd631, D6Ertd631e, NS7, RAFB, RAFB1
BRCA1	BRCA1 DNA repair associated, BRCA1, DNA repair associated, BRCAI, BRCC1, breast cancer 1, early onset, BROVCA1, FANCS, PNCA4, PPP1R53, PSCP, RNF53
c-lap	IAP, NAIP
Ca2+	14127-61-8, Ca+2, calcium, calcium(2+), calcium cation, calcium citrate, calcium ion, calcium, ion (Ca2+), calcium ions, Citracal, tricalcium dicitrate
CaMKII	CA2+/CALMODULIN DEPENDENT KINASE II, Ca+/calmodulin-dependent protein kinase ii, calmodulin-dependent protein kinase 2, Calmodulin Kinase II, CAMK2, CaM Kinase II, Ccdpk ii
cAMP	11002-78-1, 33116-15-3, 3',5'-cyclic AMP, 3',5'-monophosphate, adenosine cyclic, 37839-81-9, (4aR,6R,7R,7aS)-6-(6-aminopurin-9-yl)-2-hydroxy-2-oxo-4a,6,7,7a-tetrahydro-4H-furo[3,2-d][1,3,2]dioxaphosphinin-7-ol, 54532-48-8, 55576-98-2, 60-92-4, 66067-13-8, 68407-13-6, adenosine 3',5'-phosphate, adenosine, cyclic 3',5'-(hydrogen phosphate), adenosine cyclic 3,5 monophosphate, adenosine cyclic 3',5'-monophosphate, adenosine cyclic monophosphate, C10H12N5O6P, cAMP, cyclic-3',5'-monophosphate, adenosine, cyclic adenosine monophosphate, cyclic adenylic acid, cyclic AMP, disodium salt, cyclic AMP, monoammonium salt, cyclic AMP, monopotassium salt, cyclic AMP, monosodium salt, cyclic AMP, sodium salt
CASP3/6/7	CASP3/6/7, Caspase 3, 6, 7, Caspase-3, -6, and -7
CASP9	AI115399, APAF-3, AW493809, Casp, Casp9 v1, Caspase-9, ICE-, ICE-LAP6, MCH6, PPP1R56
Caspase8/10	Casp8/10, Caspase 8,10
CBL	4732447J05Rik, Casitas B-lineage lymphoma, CBL2, CBLA, Cbl proto-oncogene, Cbl ubiquitin ligase, c-Cb, C-CBL, FRA11B, LOC283153, NSLL, p120 Cbl, RGD1561386, RNF55
CCND1	AI327039, B-CELL CLL/LYMPHOMA 1, bcl-, BCL1, cD1, CycD1, CYCLIN D1, Cyl-, Cyl-1, D11S287E, G1/S-Specific Cyclin D1, PR, PRAD1, U21B31
Cd2+	22537-48-0, cadmium(2+), cadmium acetate, cadmium cation, cadmium ion, cadmium, ion (Cd2+), Cd+2
CDC25	mRNA encoding Cdc25-like
CDC42	AI747189, AU018915, CDC42Hs, cell division cycle 42, CELLULAR GROWTH REGULATING, G25K, TKS
CDH1	AA960649, ARC-1, BCDS1, cadherin 1, Cadherin E, CD324, CDHE, CSEIL, E-ca, ECAD, E-cadh, E-cadherin, L-C, L-CAM, Um, UVO, uvomorulin
CDK	Cdks, cyclin-dependent kinase, Cyclin-Dependent Kinases, G1 CDK
CDK2	A630093N05Rik, CDC2-RELATED KINASE, CDKN2, Cyclin A associated kinase, cyclin-dependent kinase 2, CYCLIN E ASSOCIATED KINASE, p33(CDK2)
CDK2-CyclinE	Cyclin E-CDK2
CDKN1A	CAP, CAP20, CDK, CDKI, Cdkn, CDKN1, CDKNA1, CI, CIP1, cyclin-dependent kinase inhibitor 1A, cyclin-dependent kinase inhibitor 1A (P21), mda, MDA-6, P2, P21, p21C, p21Cip, p21CIP1, P21W, p21WAF, p21Waf1, PzI Cyclin-Dependent Kinase Inhibitor, SD, SDI1, UV96, Waf, WAF1
CDKN1B	AA408329, AI843786, Cdki1b, CDKN4, cyclin-dependent kinase inhibitor 1B, CYCLIN-DEPENDENT KINASE INHIBITOR P27, KIP1, MEN1B, MEN4, p2, p27, p27K, P27kip, P27KIP1, P28-ICK
CDKN2A	A, Arf, ARF-INK4a, CDK4I, CDKN2, CMM2, CYCLIN-DEPENDENT KINASE INHIBITOR 2A, INK4, INK4A, INK4a-ARF, Ink4a/Arf, MLM, MTS, MTS-1, p1, p14ARF/ p16INK4a, p16, p16/ARF, p16Cdkn2a, p16I, p16 INK4, p16/ INK4a, P19, p19ARF, Pct, PCTR1, TP16
CDKN2B	AV083695, CDK4I, cyclin-dependent kinase inhibitor 2B, INK4B, MTS, MTS2, p1, P15, p15IN, p15INK4, p15INK4b, p15(INK4b)
CDKN2C	C77269, CDKN6, cyclin-dependent kinase inhibitor 2C, INK, INK4C, p1, p18, p18IN, p18-INK4C, p18-INK6
CDKN2D	cyclin dependent kinase inhibitor 2D, INK, INK4D, p1, p19, p19IN, p19-INK4D
CFLAR	2310024N18RIK, A430105C05Rik, AU021929, Ca, CASH, CASP8 and FADD-like apoptosis regulator, CASP8AP1, Caspase 8 associated, Casper, c-F, c-FLIP, CLARP, F, FLAME, FLAME-1, FLICE-LIKE IP, FLIP, Gm9845, I-FLICE, LOC102724614, MRIT
CHEK1	C85740, checkpoint kinase 1, CHK1, rad27
CHEK2	CDS1, Check2, checkpoint kinase 2, CHK2, hCds1, HUCDS1, LFS2, PP1425, Rad, RAD53
CREBBP	AW558298, CB, CBP, CBP/p300, CREB binding protein, KAT, KAT3A, MKHK1, p300/CBP, RSTS, RSTS1, RTS
CRK	c-Crk, c-Crk2, Cr, CRK2, Crko, CRK proto-oncogene, adaptor protein, FLJ11558, p38, v-crk avian sarcoma virus CT10 oncogene homolog
CTNNB1	armadillo, Beta-cat, beta CATENIN, Bfc, Cat, CATENIN beta, catenin beta 1, catenin (cadherin associated protein), beta 1, catenin (cadherin associated protein), β 1, CATENIN β, catenin β 1, CATNB, CTNB1, CTNNB, CTNN beta, CTNN β, EVR7, Mesc, MRD19, NEDSDV, β-cat, β-catenin
CTNND1	AA409437, AU019353, BCDS2, Ca, CAS, catenin (cadherin associated protein), delta 1, catenin (cadherin associated protein), δ 1, catenin delta 1, catenin δ 1, CATNS, Ctn, CTNND, CTNN delta, CTNN delta1, CTNN δ, CTNN δ1, mKIAA0384, P12, P120, P120CAS, p120(CAS), p120-Catenin, P120CTN, p120(CTN), Pp120
CTNNα-CTNN β-CTNNδ	CTNNalpha-CTNNbeta-CTNNdelta
CyclinD	CycD, Cyclin D1
CYCS	CYC, CYCSA, CYTC, CYTOC, CYTOCHROME C, cytochrome c, somatic, cytochrome c, somatic-like, ENSMUSG00000058927, HCS, LOC100363502, THC4, X laevis XLCL2
DAG	DAG, diacylglycerides, diglyceride
DAXX	BING2, DAP6, death-domain associated protein, EAP1, Fas death domain-associated protein, PML ASSOCIATED FACTOR
DIABLO	0610041G12Rik, 1700006L01RIK, AU040403, DFNA64, diablo IAP-binding mitochondrial protein, diablo, IAP-binding mitochondrial protein, Sm, SMAC
DVL1	DISHEVELED, dishevelled segment polarity protein 1, DRS2, DSH, DVL, DVL1L1, DVL1P1, mKIAA4029
ELK1	ELK, ELK1, member of ETS oncogene family, ETS transcription factor ELK1, p62TCF, RGD:2549, TCF/ELK
EP300	A430090G16, A730011L11, E1A binding protein p300, KAT3, KAT3B, MKHK2, p30, p300, p300 HAT, RSTS2
ERK1/2	MAPK p44/42, MAPK p44/p42, p42/44 mapk, P42/p44 erk, P42/p44 mapk, p42/p44 MAP KINASE
FADD	DEATH domain-containing ADAPTOR, Fas associated via death domain, Fas (TNFRSF6)-associated via death domain, GIG3, MORT1, Mort1/F, Mort1/FADD
FANCD2	2410150O07RIK, AU015151, BB137857, FA4, FACD, FA complementation group D2, FAD, FA-D2, FANCD, Fanconi anaemia, complementation group D2, Fanconi anemia, complementation group D2
FAS	AI196731, ALPS1A, AP, APO-1, APT1, CD95, CD95L, CD95 receptor, FAS1, FAS/APO1, Fas cell surface death receptor, FasR, FASTM, Fas (TNF receptor superfamily member 6), lpr, Receptor for Fas Ligand, Receptors for Fas Ligand, TNF, Tnfr, TNFR6, Tnf receptor member 6, TNFRSF6
FASLG	ALPS1B, APT1, APT1LG1, APTL, CD178, CD95, CD95-L, F, Fa, FASL, Fas Ligand, Fas ligand (TNF superfamily, member 6), gld, mFasL, Tnfl6, Tnfs, TNFSF6, TNLG1A
FOS	AP-1, c-f, C-FOS, D12Rfj, D12Rfj1, FBJ osteosarcoma oncogene, Fos proto-oncogene, AP-1 transcription factor subunit, p55
FOXO1	Afx, Afxh, AI876417, FKH, FKH1, FKHR, FKHR1, Forkhead, forkhead box O1, Fox, FOXO1A
Frizzled	Frizzled receptor, FZ, FZD, Wnt receptor
Frizzled-LRP	FZD-LRP1/5/6
FYN	AI448320, AW552119, C-FYN, Fyn proto-oncogene, FYN proto-oncogene, Src family tyrosine kinase, FYNT, LOC102724705, p59-FYN, p59 Fyn B, SLK, SRC-LIKE KINASE, SYN
G protein	Galphabetagamma, Galpha-Gbeta-Ggamma, Galphai-Gbeta-Ggamma, Galphaq-Gbeta-Ggamma, Gpro, G protein alpha beta gamma, G protein alpha-G protein beta-GDP-G protein gamma, G protein alpha-G protein beta-G protein gamma, G-protein complex, G protein α-G protein β-GDP-G protein γ, G protein α-G protein β-G protein γ, G-protein α-β-γ, Guanine nucleotide binding protein, Gα-Gβ-Gγ, Gαi-Gβ-Gγ, Gαq-Gβ-Gγ, Gαβγ
G proteinalpha	Galpha, G-Protein Alpha Subunit, G protein α, G-Protein α Subunit, Gα
Gammasecretase	Gamma Secretase, Secretase γ, γ-Secretase
GLI1	AV235269, GL1, GLI, GLI family zinc finger 1, GLI-Kruppel family member GLI1, PAPA8, PPD1, Zfp-, ZFP5
GRB2	AA408164, ASH, Ash-psi, EGFRBP-GRB2, GRAB2, GRBS, growth factor receptor bound protein 2, MST084, MSTP084, NCKAP2
Grb2-Shc1-Sos	Grb2-Sos-Shc, SHC-GRB2-SOS
GSK3	Glycogen synthase kinase, Gsk, GSK3 alpha/beta, GSK3 α/β
GSK3beta-Axin-APC-Ctnnbeta	APC-CTNNbeta-AXIN-GSK3beta, APC-CTNNβ-AXIN-GSK3β, AXIN-APC-GSKbeta-CTNNbeta, AXIN-APC-GSKβ-CTNNβ, GSK3β-AXIN-APC-CTNNβ
GSK3B	7330414F15Rik, 8430431H08Rik, C86142, glycogen synthase kinase 3 beta, glycogen synthase kinase 3 β, GSK-, GSK-3, GSK-3be, GSK-3beta, GSK-3β, GSKbeta, GSKβ, Tpk1
HAT1	2410071B14Rik, AA536933, histone acetyltransferase 1, histone aminotransferase 1, KAT, KAT1
Hedgehog	Hh
HIF1A	AA959795, bHLHe7, bHLHe78, HIF-1, HIF1-ALPHA, HIF-1alpha (hydroxylated), HIF-1-α, HIF-1α (hydroxylated), Hypoxia inducible factor 1 alpha subunit, hypoxia inducible factor 1, alpha subunit, hypoxia inducible factor 1 subunit alpha, hypoxia inducible factor 1 subunit α, Hypoxia inducible factor 1 α subunit, hypoxia inducible factor 1, α subunit, MO, MOP1, PASD8

Symbol	Synonym(s)
HIF1alpha-NICD	HIF1α-NICD
HIPK2	1110014O20RIK, B230339E18RIK, homeodomain interacting protein kinase 2, LOC100505582, LOC653052, PRO0593, St, Stank
Ikb	I KAPPA B, Ikbeta, Ikβ, Iκ-B
IkB-NfkB	IkappaB-NFkappaB, IκB-NFκB, NFkB-IkB
Integrin	Integrin alpha-beta, integrin-extracellular matrix, INTEGRIN receptor, Integrin α-β
IP3	27121-73-9, inositol trisphosphate, IP3, myo-inositol, tris(dihydrogen phosphate)
IRS1	ENSMUSG00000022591, G972, G972R, HIRS-1, insulin receptor substrate 1, IR, IRS1IRM
JAK	JAK kinase
Jnk	JNK 54/46, Jnk p56, JNK/SAPK, JUN KINASE, p40, p47, Sapk/Jnk
JUN	Activator protein 1, AP-1, API-1, c-ju, cJUN, Junc, jun proto-oncogene, Jun proto-oncogene, AP-1 transcription factor subunit, LOC100288387, LOC100291417, LOC100293034, p39, v-Jun, V-jun Avian Sarcoma Virus 17 Oncogene Homolog, V-jun Sarcoma Virus 17 Oncogene Homolog
LAMTOR3	AW556229, late endosomal/lysosomal adaptor, MAPK and MTOR activator 3, LOC100132990, Map, Map2k, MAP2K1IP1, MAPBP, MAPKSP1, Mek binding partner 1, Mp, MP1, PRO0633, Ragulator3
LEF1	3000002B05, AI451430, Lef-, lymphoid enhancer binding factor 1, TCF10, TCF1ALPHA, TCF7L3, TCF/LEF
MAP2K1/2	MEK1/2, MKK1/2
MAP3K5	7420452D20Rik, A, APOPTOSIS SIGNAL REGULATED KINASE 1, AS, ASK, ASK1, M3K5, MAPKKK5, MEKK5, mitogen-activated protein kinase kinase kinase 5, RGD1306565
MAP3K7	CSCF, FMD2, Map3k7 predicted, MEKK7, mitogen-activated protein kinase kinase kinase 7, TAK1, TGF1a, tgf β activated kinase 1
Map3k7-Map3k7ip1-	Tab1-Tab2-Tak1
MAX	AA960152, AI875693, bHLHd, bHLHd4, bHLHd5, bHLHd6, bHLHd7, bHLHd8, Max protein, MYC associated factor X, Myn
Max-Myc	cMyc-MAX, Myc-MAX
MDM2	1700007J15Rik, AA415488, ACTFS, hdm2, HDMX, LSKB, MDM2-A1, MDM2 proto-oncogene, MGC5370, Transformed 3t3 cell double minute 2, transformed mouse 3T3 cell double minute 2
Mkk3/6	MEK3/6, Mkk3/6 (mitogen activated protein kinase kinase 3/6), MKK3/MKK6
MYC	AU016757, bHLHe3, bHLHe39, CMYC, C-MYC-P64, mMyc, MRTL, Myc2, MYCC, MYC proto-oncogene, bHLH transcription factor, myelocytomatosis oncogene, N, Niard, Nird, RNCMYC
NBN	ATV, AT-V1, AT-V2, Nb, NBS, NBS1, NIBRIN, P95
NCSTN	9430068N19Rik, AA727311, APH2, ATAG1874, D1Dau13, D1Dau13e, Kiaa0253, mKIAA0253, Nc, NCT, ni, NICASTRIN
NF1	AW494271, Dsk, Dsk9, E030030H24RIK, LOC646021, Mhdads, Mhdadsk9, Neurofibromatosis 1, NEUROFIBROMIN, neurofibromin 1, Nf-, NF1-GAP, NFNS, VRNF, WSS
NFkB	NF-KAPPA B, NF-κ B, nuclear factor-κ b, transcription factor nuclear factor κ b
NLK	AI194375, LOC100044468, nemo like kinase, RGD1561602
NOTCHIC	9930111A19Rik, AOS5, AOVD1, hN1, lin, lin-12, Mi, Mis6, N, N1, NOTCH, notch receptor 1, Ta, TAN1
p38MAPK	P38, p38 MAP KINASE, P38 MITOGEN-ACTIVATED protein KINASE
Patched	PTC, PTCH
PI3K	1-phosphatidylinositol 3-kinase, 2.7.1.137, ATP:1-phosphatidyl-1D-myo-inositol 3-phosphotransferase, Phosphatidylinositol 3 kinase, phosphatidylinositol 3'-kinase, PI3-kinase, PtdIns 3 Kinase, type III phosphoinositide 3-kinase, type I phosphatidylinositol kinase, Vps34p
PIP2	1,2-diacyl-sn-glycero-3-phospho-(1'-myo-inositol-4',5'-bisphosphate), 1-O-(3-sn-phosphatidyl)-1D-myo-inositol 4,5-bis(dihydrogen phosphate), 1-phosphatidyl-1D-myo-inositol 4,5-bisphosphate, C11H19O19P3R2
PIP3	1-phosphatidyl-1D-myo-inositol 3,4,5-trisphosphate, phosphatidylinositol-3,4,5-trisphosphate, phosphoinositide (3,4,5) P3, PI(3,4,5)P3, PIns(3,4,5)P3, PIP3, PtdIns(3,4,5)P3
PKA	A-Kinase, cAMP-Dependent Protein Kinase, cyclic AMP depended protein kinase, protein KINASE A
PKC	Cnpkc, PKC, Pkc(s), Protein Kinase C
Plcbeta	Phospholipase c beta, Phospholipase C β, PLCB, PLCβ
PMAIP1	APR, N, NOXA, phorbol-12-myristate-13-acetate-induced protein 1
Presenilin	PS, PS1/2, PSEN1/2
PRKDC	AI326420, AU019811, DNA-, DNA-DEPENDENT protein KINASE, DNAPDcs, DNAPK, DNA-PKC, DNA-PKcs, DNPK1, DOX, DOXNPH, dxn, dxnph, HYRC, HYRC1, IMD26, p350, p460, Prkdc predicted, protein kinase, DNA activated, catalytic polypeptide, protein kinase, DNA-activated, catalytic subunit, scid, slip, XRCC, XRCC7
PSENEN	1700023M09Rik, ACNINV2, MDS033, MSTP064, PEN-2, presenilin enhancer gamma secretase subunit, presenilin enhancer, gamma-secretase subunit, presenilin enhancer γ secretase subunit, presenilin enhancer, γ-secretase subunit, RGD1312037
PTCH1	A230106A15RIK, BCNS, mes, NBCCS, patched 1, Pt, PTC, PTC1, PTCH, Ptch2, wi, wig
PTK2	FA, Fad, FADK, FADK 1, FAK, FAK1, FAK related non-kinase, FR, p125FAK, pp125FAK, PPP1R71, protein tyrosine kinase 2, PTK2 protein tyrosine kinase 2, TYROSINE KINASE 2
PTPN11	2700084A17Rik, AW536184, BPTP3, CFC, JMML, METCDS, MGC14433, Noonan syndrome 1, NS1, protein tyrosine phosphatase non-receptor type 11, protein tyrosine phosphatase, non-receptor type 11, PTP, PTP-1D, PTP2C, S, SAP-2, Sh, SH-P, SHP-2, SH-PTP2, SH-PTP3, Src homology protein 2, SYP
RAF1	6430402F14Rik, AA990557, BB129353, CMD1NN, c-R, Cra, CRAF, Craf1, D830050J10Rik, leukaemiA ONCOGENE HOMOLOG1, LEUKEMIA ONCOGENE HOMOLOG1, NS5, Raf-1 proto-oncogene, serine/threonine kinase, v-, v-Raf, v-raf-leukaemia viral oncogene 1, v-raf-leukemia viral oncogene 1
Ral	Ral A/B
RALBP1	DNP-SG ATPase, R, ralA binding protein 1, Ral GAP, Rik, RIP1, RL, RLIP1, RLIP76
RALGDS	Gn, Gnds, Hs.560937, mKIAA1308, Ra, RalGDSB, RalGEF, ral guanine nucleotide dissociation stimulator, Rg, RGDS, RGF
RAPGEF1	4932418O06Rik, C3G, C3G-1, C3G-2, Grf, GRF2, Rap guanine nucleotide exchange factor 1, Rap guanine nucleotide exchange factor (GEF) 1
RAPGEF3	2310016P22Rik, 9330170P05Rik, bcm910, CAMP-GEFI, CGEF1, Epa, EPAC, EPAC1, HSU79275, Rap guanine nucleotide exchange factor 3, Rap guanine nucleotide exchange factor (GEF) 3
Ras-grf	Guanine Nucleotide Releasing
RASA1	CM-AVM, CMAVM1, G, GAP, GAPX, p120-, p120GAP, P120RASGAP, PKWS, RASA, RASGAP, RAS p21 protein activator 1
RASGRP1	CALDAG-GEFI, CALDAG-GEFII, IMD64, RASGRP, RAS guanyl releasing protein 1
Rb-E2Ftranscriptionrepression	Rb1-E2f1, Rb-E2F, Rb-E2F1
RB1	OSRC, p, p105, p105-Rb, p110 RB, p110-RB1, pp105, pp110, PPP1R130, pRb, R, RB, RB-ASSOCIATED, RB transcriptional corepressor 1, Retinoblastome tumor-suppression protein rb
RBL1	AW547426, CP107, LOC683869, p10, p107, PRB1, RB transcriptional corepressor like 1
RBPJ	AI843960, AOS3, CBF-1, csl, Igk, Igkj, IGKJRB, IGKJRB1, Igkrsbp, KBF2, RBP, RBP 2N, RBP-JK, RBP-J kappa, RBP-J κ, RBPSUH, Rbpsuh1, Recombination signal binding, recombination signal binding protein for immunoglobulin kappa J region, recombination signal binding protein for immunoglobulin κ J region, SUH
RHO	GTPase Rho, Rho, Rho Family, RHO-GTPASE, Rho-like Gtpase
SHC1	p52SHC, p6, p66, p66s, P66shc, Sh, SHC, Shc (46 kDa isoform), SHCA, SHC adaptor protein 1, Shc p66 isoform, src homology 2 domain-containing transforming protein C1
Smad1/5/8	SMAD1/5/8
Smad1/5/8-	SMAD 1,4,5,8
Smad2/3-	Smad 2/3/4
SMAD4	AW743858, D18Wsu70, D18Wsu70e, DPC, DPC4, JIP, Madh, MADH4, MYHRS, SMAD family member 4, Smaug1
SMAD6	AOVD2, b2b390C, b2b390Clo, HsT17432, Madh, MADH6, MADH7, SMAD family member 6
SMAD7	CRCS3, Madh, MADH7, MADH8, SMAD family member 7
SMO	bnb, CRJS, E130215L21Rik, FZD11, Gx, PHLS, SMOH, Smoothened, smoothened, frizzled class receptor
SRC	ASV, AW259666, BS27, c-SRC, p60-Src, PP60, Pp60/c-Src, pp60c, pp60c-src, Rous sarcoma oncogene, SRC1, SRC proto-oncogene, non-receptor tyrosine kinase, THC6, TVHUSC
STK36	1700112N14RIK, B930045J24, FU, Fuse, Fused, mKIAA1278, serine/threonine kinase 36, Stk36 (predicted)
SUFU	b2b273C, JBTS32, PRO1280, Su, SUFUH, SUFU negative regulator of hedgehog signaling, SUFUXL
SYNGAP1	Gm1963, MRD5, RASA1, RASA5, Sy, Synaptic Ras-GAP 1, synaptic Ras GTPase activating protein 1, synaptic Ras GTPase activating protein 1 homolog (rat), SYNGAP
TAB2	1110030N06Rik, A530078N03Rik, CHTD2, LOC101928709, Map3k, MAP3K7IP2, mKIAA0733, RP1 111D63, TGF-beta activated kinase 1 (MAP3K7) binding protein 2, TGF-beta activated kinase 1/MAP3K7 binding protein 2, TGF-β activated kinase 1 (MAP3K7) binding protein 2, TGF-β activated kinase 1/MAP3K7 binding protein 2
TBID	2700049M22Rik, AI875481, AU022477, BH3 interacting domain death agonist, cBid, FP497
Tgfbeta	Tgfb, TGF-beta 1, 2, and 3, TGF β, TGF-β 1, 2, and 3, transforming growth factor-β
Tgfbetarecept	TGFbetaR, TGFBR, TGFβR, Tgf β receptor
TGFBR1	AAT5, ACVRLK4, AL, Alk, ALK-5, AU017191, ESK2, ESS1, LDS1, LDS1A, LDS2A, LOC103690035, LOC666236, MSSE, SKR4, Tbet, Tbeta, TbetaR-I, TBR-I, TGFbeta1R1, TGF-beta1 receptor, TGF beta I receptor, Tgf-beta-r1, TGF beta receptor type I, TGFbetaRI, Tgfb receptor I, TGFR-1, TGFβ1R1, TGF β I receptor, TGFβ R1, Tgf -β R1/R4, Tgf-β receptor1, TGF β receptor type I, TGFβRI, TGF β type 1 receptors, Tgf β type i receptor, transforming growth factor beta receptor 1, transforming growth factor, beta receptor 1, transforming growth factor, beta receptor I, transforming growth factor β receptor 1, transforming growth factor, β receptor 1, transforming growth factor, β receptor I, transforming growth factor-β receptor type 1, T β R1
TGFBR2	1110020H15Rik, AAT3, AU042018, DNIIR, FAA3, LDS1B, LDS2, LDS2B, MFS2, RIIC, RIIDN, TAAD2, Tbet, Tbeta, TbetaR-II, TBR, TBR-II, TBRLL, TGFbeta1R2, TGF-beta 2, Tgf beta2 receptor, TGFbeta R2, TGF-beta receptor 2, TGF-beta receptor type 2, TGF beta receptor type II, TGFbeta-RII, Tgfbr2T, TGFB receptor II, Tgfbrii, TGFR-2, TGFβ1R2, TGF-β 2, TGFβ R2, TGF-β receptor 2, TGF-β receptor type 2, TGF β receptor type II, TGFβRII, Tgf-β type ii receptor, transforming growth factor beta receptor 2, transforming growth factor, beta receptor 2, transforming growth factor, beta receptor II, transforming growth factor β receptor 2, transforming growth factor, β receptor 2, transforming growth factor, β receptor II, type 2 TGF-β receptors, T β r2
TP53	bbl, BCC7, bfy, bhy, BMFS5, LFS1, p4, p44, p5, P53, P53 cellular tumour antigen, p53 tumor suppressor, transformation related protein 53, TRP53, tumor protein p53, tumour protein p53
Type I BMP	ALK 3,6, ALK 3/6, BMP2 receptor type1, BMP4 receptor type1, Bmpr1, Bmpri
WNT1	BMND16, Int, INT1, Ol15, sw, swaying, Wg, wingless-type MMTV integration site family, member 1, Wnt-, Wnt family member 1
WNT5A	8030457G12Rik, hWNT5A, LOC102724616, wingless-type MMTV integration site family, member 5A, Wnt-, Wnt family member 5A
ZBTB17	AA589413, Lp-1, Miz, MIZ-1, mZ13, pHZ-67, Zfp10, Zfp100, zinc finger and BTB domain containing 17, ZNF151, ZNF60

Pathway Analysis Using IPA Software; canonical pathway

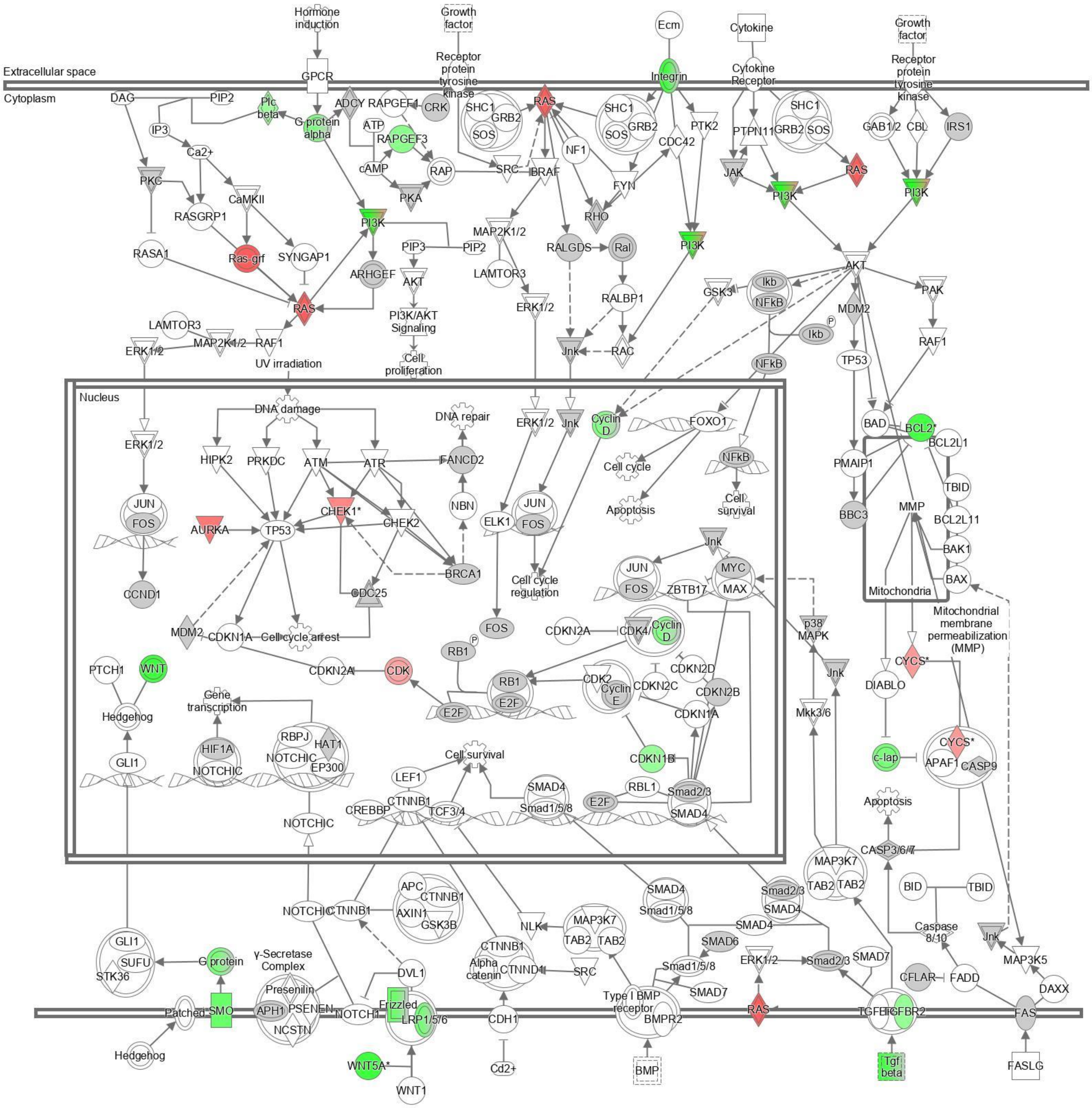

Figure S58. Molecular Mechanism of cancer at 8 days

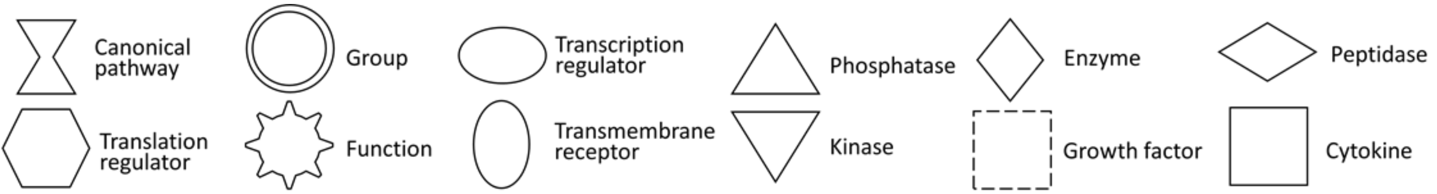

Red: Increased, FDR<0.05 versus solvent control

Green: Decreased, FDR<0.05 versus solvent control

Symbol	Synonym(s)
ADCY	3',5'-cyclic AMP synthetase, 4.6.1.1, AC, Adenylate Cyclase, Adenyl Cyclase, Adenylyl cyclase, ATP diphosphate-lyase (cyclizing), mAC, sAC
AKT	AKT1/2/3, B/Akt, PKB, RAC-PK
Alphacatenin	CTNN alpha, CTNN α, α catenin
Ap1	activator protein-1, c-Jun
APAF1	6230400I06RIK, Ap, Apaf1l, apoptotic peptidase activating factor 1, CED4, fog, mKIAA0413
APC	AI047805, APC1, Apc7, APC (PROC), APC regulator of WNT signaling pathway, APC, WNT signaling pathway regulator, AU020952, AW124434, BTPS2, CC1, DESMD, DP2, DP2.5, DP3, Familial adenomatous polyposis, GS, M, mAPC, Min, PPP1R46, RATAPC
Apoptosome	APAF1-Caspase 9-CytoC, apoptosis adaptor protein complex, Cytochrome C-APAF1-Caspase 9
ARHGEF	Ras GEF, RhOGEF
ATM	AI256621, AT1, ATA, ataxia telangiectasia mutated, ATC, ATD, ATDC, ATE, ATM serine/threonine kinase, C030026E19RIK, TEL1, TELO1
ATP	[[(2R,3S,4R,5R)-5-(6-aminopurin-9-yl)-3,4-dihydroxyoxolan-2-yl]methoxy-hydroxyphosphoryl] phosphono hydrogen phosphate, 56-65-5, 9-beta-D-arabinofuranosyladenine 5'-triphosphate, 9-β-D-arabinofuranosyladenine 5'-triphosphate, adenosine 5'-(tetrahydrogen triphosphate), adenosine 5'-triphosphate, ATP, ATP4-, C10H16N5O13P3
ATR	ataxia telangiectasia and Rad3 related, Ataxia-telangiectasia-like, ATR serine/threonine kinase, FCTCS, FRP1, LOC100365674, LOC367198, LOC684113, MEC1, SCKL, SCKL1
AURKA	AI, AIK, AIRK1, Ar, ARK-1, Au, AU019385, AURA, AURORA 2, AURORA A, AURORA KINASE, aurora kinase A, Aurora Related Kinase1, AW539821, Ayk, Ayk1, BTAK, I, IA, IAK, IAK1, PPP1R47, Stk, STK15, STK6, STK7
AXIN1	AI316800, AXIN, AXIN form I, Fu, fused, Kb, Ki, kinky, knobbly, PPP1R49
BAD	AI325008, Bad v1, Bad v2, BBC2, BCL2-associated agonist of cell death, BCL2L8
BAK1	Ba, BAK, BAK-LIKE, BCL2-antagonist/killer 1, BCL2L7, CDN1, N-B, N-BAK1
BAX	Bcl2-associated X, BCL2 associated X, apoptosis regulator, BCL2-associated X protein, BCL2L4
BBC3	BCL2 binding component 3, JFY-1, PU, PUMA, PUMA/JFY1
BCL2	AW986256, B cell leukaemia/lymphoma 2, B cell leukemia/lymphoma 2, Bcl-, Bcl2 alpha, BCL2 apoptosis regulator, BCL2, apoptosis regulator, Bcl2 α, C430015F12Rik, D630044D05RIK, D830018M01RIK, LOC100046608, ORF16, PPP1R50
BCL2L1	bBclxl, Bcl, BCL2L, BCL2-like 1, BCLX, Bcl-X beta, Bclx gamma, BCL-XL/S, Bcl-X β, Bclx γ, PPP1R52
BCL2L11	1500006F24RIK, BAM, BCL2 like 11, BCL2-like 11 (apoptosis facilitator), Bi, BIM, Bo, BOD, BODL, LOC150819
BMP	BMP3, BMP-3A, BONE MORPHOGENIC, Osteogenin
BMPR2	2610024H22RIK, AL117858, AW546137, BB189135, BM, BMP-, BMP-2, BMPR3, BMPR-II, BMR2, bone morphogenetic protein receptor type 2, bone morphogenetic protein receptor, type II (serine/threonine kinase), BRK-3, Gm20272, POVD1, PPH1, T-ALK, Type ii bmp receptor
BRAF	9930012E13RIK, AA120551, AA387315, AA473386, AI447469, Bra, B-RAF1, Braf-2, B-Raf proto-oncogene, serine/threonine kinase, Braf transforming gene, C230098H17, C87398, D6Ertd631, D6Ertd631e, NS7, RAFB, RAFB1
BRCA1	BRCA1 DNA repair associated, BRCA1, DNA repair associated, BRCAI, BRCC1, breast cancer 1, early onset, BROVCA1, FANCS, PNCA4, PPP1R53, PSCP, RNF53
c-lap	IAP, NAIP
Ca2+	14127-61-8, Ca+2, calcium, calcium(2+), calcium cation, calcium citrate, calcium ion, calcium, ion (Ca2+), calcium ions, Citracal, tricalcium dicitrate
CaMKII	CA2+/CALMODULIN DEPENDENT KINASE II, Ca+/calmodulin-dependent protein kinase ii, calmodulin-dependent protein kinase 2, Calmodulin Kinase II, CAMK2, CaM Kinase II, Ccdpk ii
cAMP	11002-78-1, 33116-15-3, 3',5'-cyclic AMP, 3',5'-monophosphate, adenosine cyclic, 37839-81-9, (4aR,6R,7R,7aS)-6-(6-aminopurin-9-yl)-2-hydroxy-2-oxo-4a,6,7,7a-tetrahydro-4H-furo[3,2-d][1,3,2]dioxaphosphinin-7-ol, 54532-48-8, 55576-98-2, 60-92-4, 66067-13-8, 68407-13-6, adenosine 3',5'-phosphate, adenosine, cyclic 3',5'-(hydrogen phosphate), adenosine cyclic 3,5 monophosphate, adenosine cyclic 3',5'-monophosphate, adenosine cyclic monophosphate, C10H12N5O6P, cAMP, cyclic-3',5'-monophosphate, adenosine, cyclic adenosine monophosphate, cyclic adenylic acid, cyclic AMP, disodium salt, cyclic AMP, monoammonium salt, cyclic AMP, monopotassium salt, cyclic AMP, monosodium salt, cyclic AMP, sodium salt
CASP3/6/7	CASP3/6/7, Caspase 3, 6, 7, Caspase-3, -6, and -7
CASP9	AI115399, APAF-3, AW493809, Casp, Casp9 v1, Caspase-9, ICE-, ICE-LAP6, MCH6, PPP1R56
Caspase8/10	Casp8/10, Caspase 8,10
CBL	4732447J05Rik, Casitas B-lineage lymphoma, CBL2, CBLA, Cbl proto-oncogene, Cbl ubiquitin ligase, c-Cb, C-CBL, FRA11B, LOC283153, NSLL, p120 Cbl, RGD1561386, RNF55
CCND1	AI327039, B-CELL CLL/LYMPHOMA 1, bcl-, BCL1, cD1, CycD1, CYCLIN D1, Cyl-, Cyl-1, D11S287E, G1/S-Specific Cyclin D1, PR, PRAD1, U21B31
Cd2+	22537-48-0, cadmium(2+), cadmium acetate, cadmium cation, cadmium ion, cadmium, ion (Cd2+), Cd+2
CDC25	mRNA encoding Cdc25-like
CDC42	AI747189, AU018915, CDC42Hs, cell division cycle 42, CELLULAR GROWTH REGULATING, G25K, TKS
CDH1	AA960649, ARC-1, BCDS1, cadherin 1, Cadherin E, CD324, CDHE, CSEIL, E-ca, ECAD, E-cadh, E-cadherin, L-C, L-CAM, Um, UVO, uvomorulin
CDK	Cdks, cyclin-dependent kinase, Cyclin-Dependent Kinases, G1 CDK
CDK2	A630093N05Rik, CDC2-RELATED KINASE, CDKN2, Cyclin A associated kinase, cyclin-dependent kinase 2, CYCLIN E ASSOCIATED KINASE, p33(CDK2)
CDK2-CyclinE	Cyclin E-CDK2
CDKN1A	CAP, CAP20, CDK, CDKI, Cdkn, CDKN1, CDKNA1, CI, CIP1, cyclin-dependent kinase inhibitor 1A, cyclin-dependent kinase inhibitor 1A (P21), mda, MDA-6, P2, P21, p21C, p21Cip, p21CIP1, P21W, p21WAF, p21Waf1, PzI Cyclin-Dependent Kinase Inhibitor, SD, SDI1, UV96, Waf, WAF1
CDKN1B	AA408329, AI843786, Cdki1b, CDKN4, cyclin-dependent kinase inhibitor 1B, CYCLIN-DEPENDENT KINASE INHIBITOR P27, KIP1, MEN1B, MEN4, p2, p27, p27K, P27kip, P27KIP1, P28-ICK
CDKN2A	A, Arf, ARF-INK4a, CDK4I, CDKN2, CMM2, CYCLIN-DEPENDENT KINASE INHIBITOR 2A, INK4, INK4A, INK4a-ARF, Ink4a/Arf, MLM, MTS, MTS-1, p1, p14ARF/ p16INK4a, p16, p16/ARF, p16Cdkn2a, p16I, p16 INK4, p16/ INK4a, P19, p19ARF, Pct, PCTR1, TP16
CDKN2B	AV083695, CDK4I, cyclin-dependent kinase inhibitor 2B, INK4B, MTS, MTS2, p1, P15, p15IN, p15INK4, p15INK4b, p15(INK4b)
CDKN2C	C77269, CDKN6, cyclin-dependent kinase inhibitor 2C, INK, INK4C, p1, p18, p18IN, p18-INK4C, p18-INK6
CDKN2D	cyclin dependent kinase inhibitor 2D, INK, INK4D, p1, p19, p19IN, p19-INK4D
CFLAR	2310024N18RIK, A430105C05Rik, AU021929, Ca, CASH, CASP8 and FADD-like apoptosis regulator, CASP8AP1, Caspase 8 associated, Casper, c-F, c-FLIP, CLARP, F, FLAME, FLAME-1, FLICE-LIKE IP, FLIP, Gm9845, I-FLICE, LOC102724614, MRIT
CHEK1	C85740, checkpoint kinase 1, CHK1, rad27
CHEK2	CDS1, Check2, checkpoint kinase 2, CHK2, hCds1, HUCDS1, LFS2, PP1425, Rad, RAD53
CREBBP	AW558298, CB, CBP, CBP/p300, CREB binding protein, KAT, KAT3A, MKHK1, p300/CBP, RSTS, RSTS1, RTS
CRK	c-Crk, c-Crk2, Cr, CRK2, Crko, CRK proto-oncogene, adaptor protein, FLJ11558, p38, v-crk avian sarcoma virus CT10 oncogene homolog
CTNNB1	armadillo, Beta-cat, beta CATENIN, Bfc, Cat, CATENIN beta, catenin beta 1, catenin (cadherin associated protein), beta 1, catenin (cadherin associated protein), β 1, CATENIN β, catenin β 1, CATNB, CTNB1, CTNNB, CTNN beta, CTNN β, EVR7, Mesc, MRD19, NEDSDV, β-cat, β-catenin
CTNND1	AA409437, AU019353, BCDS2, Ca, CAS, catenin (cadherin associated protein), delta 1, catenin (cadherin associated protein), δ 1, catenin delta 1, catenin δ 1, CATNS, Ctn, CTNND, CTNN delta, CTNN delta1, CTNN δ, CTNN δ1, mKIAA0384, P12, P120, P120CAS, p120(CAS), p120-Catenin, P120CTN, p120(CTN), Pp120
CTNNα-CTNN β-CTNNδ	CTNNalpha-CTNNbeta-CTNNdelta
CyclinD	CycD, Cyclin D1
CYCS	CYC, CYCSA, CYTC, CYTOC, CYTOCHROME C, cytochrome c, somatic, cytochrome c, somatic-like, ENSMUSG00000058927, HCS, LOC100363502, THC4, X laevis XLCL2
DAG	DAG, diacylglycerides, diglyceride
DAXX	BING2, DAP6, death-domain associated protein, EAP1, Fas death domain-associated protein, PML ASSOCIATED FACTOR
DIABLO	0610041G12Rik, 1700006L01RIK, AU040403, DFNA64, diablo IAP-binding mitochondrial protein, diablo, IAP-binding mitochondrial protein, Sm, SMAC
DVL1	DISHEVELED, dishevelled segment polarity protein 1, DRS2, DSH, DVL, DVL1L1, DVL1P1, mKIAA4029
ELK1	ELK, ELK1, member of ETS oncogene family, ETS transcription factor ELK1, p62TCF, RGD:2549, TCF/ELK
EP300	A430090G16, A730011L11, E1A binding protein p300, KAT3, KAT3B, MKHK2, p30, p300, p300 HAT, RSTS2
ERK1/2	MAPK p44/42, MAPK p44/p42, p42/44 mapk, P42/p44 erk, P42/p44 mapk, p42/p44 MAP KINASE
FADD	DEATH domain-containing ADAPTOR, Fas associated via death domain, Fas (TNFRSF6)-associated via death domain, GIG3, MORT1, Mort1/F, Mort1/FADD
FANCD2	2410150O07RIK, AU015151, BB137857, FA4, FACD, FA complementation group D2, FAD, FA-D2, FANCD, Fanconi anaemia, complementation group D2, Fanconi anemia, complementation group D2
FAS	AI196731, ALPS1A, AP, APO-1, APT1, CD95, CD95L, CD95 receptor, FAS1, FAS/APO1, Fas cell surface death receptor, FasR, FASTM, Fas (TNF receptor superfamily member 6), lpr, Receptor for Fas Ligand, Receptors for Fas Ligand, TNF, Tnfr, TNFR6, Tnf receptor member 6, TNFRSF6
FASLG	ALPS1B, APT1, APT1LG1, APTL, CD178, CD95, CD95-L, F, Fa, FASL, Fas Ligand, Fas ligand (TNF superfamily, member 6), gld, mFasL, Tnfl6, Tnfs, TNFSF6, TNLG1A
FOS	AP-1, c-f, C-FOS, D12Rfj, D12Rfj1, FBJ osteosarcoma oncogene, Fos proto-oncogene, AP-1 transcription factor subunit, p55
FOXO1	Afx, Afxh, AI876417, FKH, FKH1, FKHR, FKHR1, Forkhead, forkhead box O1, Fox, FOXO1A
Frizzled	Frizzled receptor, FZ, FZD, Wnt receptor
Frizzled-LRP	FZD-LRP1/5/6
FYN	AI448320, AW552119, C-FYN, Fyn proto-oncogene, FYN proto-oncogene, Src family tyrosine kinase, FYNT, LOC102724705, p59-FYN, p59 Fyn B, SLK, SRC-LIKE KINASE, SYN
G protein	Galphabetagamma, Galpha-Gbeta-Ggamma, Galphai-Gbeta-Ggamma, Galphaq-Gbeta-Ggamma, Gpro, G protein alpha beta gamma, G protein alpha-G protein beta-GDP-G protein gamma, G protein alpha-G protein beta-G protein gamma, G-protein complex, G protein α-G protein β-GDP-G protein γ, G protein α-G protein β-G protein γ, G-protein α-β-γ, Guanine nucleotide binding protein, Gα-Gβ-Gγ, Gαi-Gβ-Gγ, Gαq-Gβ-Gγ, Gαβγ
G proteinalpha	Galpha, G-Protein Alpha Subunit, G protein α, G-Protein α Subunit, Gα
Gammasecretase	Gamma Secretase, Secretase γ, γ-Secretase
GLI1	AV235269, GL1, GLI, GLI family zinc finger 1, GLI-Kruppel family member GLI1, PAPA8, PPD1, Zfp-, ZFP5
GRB2	AA408164, ASH, Ash-psi, EGFRBP-GRB2, GRAB2, GRBS, growth factor receptor bound protein 2, MST084, MSTP084, NCKAP2
Grb2-Shc1-Sos	Grb2-Sos-Shc, SHC-GRB2-SOS
GSK3	Glycogen synthase kinase, Gsk, GSK3 alpha/beta, GSK3 α/β
GSK3beta-Axin-APC-Ctnnbeta	APC-CTNNbeta-AXIN-GSK3beta, APC-CTNNβ-AXIN-GSK3β, AXIN-APC-GSKbeta-CTNNbeta, AXIN-APC-GSKβ-CTNNβ, GSK3β-AXIN-APC-CTNNβ
GSK3B	7330414F15Rik, 8430431H08Rik, C86142, glycogen synthase kinase 3 beta, glycogen synthase kinase 3 β, GSK-, GSK-3, GSK-3be, GSK-3beta, GSK-3β, GSKbeta, GSKβ, Tpk1
HAT1	2410071B14Rik, AA536933, histone acetyltransferase 1, histone aminotransferase 1, KAT, KAT1
Hedgehog	Hh
HIF1A	AA959795, bHLHe7, bHLHe78, HIF-1, HIF1-ALPHA, HIF-1alpha (hydroxylated), HIF-1-α, HIF-1α (hydroxylated), Hypoxia inducible factor 1 alpha subunit, hypoxia inducible factor 1, alpha subunit, hypoxia inducible factor 1 subunit alpha, hypoxia inducible factor 1 subunit α, Hypoxia inducible factor 1 α subunit, hypoxia inducible factor 1, α subunit, MO, MOP1, PASD8

Symbol	Synonym(s)
HIF1alpha-NICD	HIF1α-NICD
HIPK2	1110014O20RIK, B230339E18RIK, homeodomain interacting protein kinase 2, LOC100505582, LOC653052, PRO0593, St, Stank
Ikb	I KAPPA B, Ikbeta, Ikβ, Iκ-B
IkB-NfkB	IkappaB-NFkappaB, IκB-NFκB, NFkB-IkB
Integrin	Integrin alpha-beta, integrin-extracellular matrix, INTEGRIN receptor, Integrin α-β
IP3	27121-73-9, inositol trisphosphate, IP3, myo-inositol, tris(dihydrogen phosphate)
IRS1	ENSMUSG00000022591, G972, G972R, HIRS-1, insulin receptor substrate 1, IR, IRS1IRM
JAK	JAK kinase
Jnk	JNK 54/46, Jnk p56, JNK/SAPK, JUN KINASE, p40, p47, Sapk/Jnk
JUN	Activator protein 1, AP-1, API-1, c-ju, cJUN, Junc, jun proto-oncogene, Jun proto-oncogene, AP-1 transcription factor subunit, LOC100288387, LOC100291417, LOC100293034, p39, v-Jun, V-jun Avian Sarcoma Virus 17 Oncogene Homolog, V-jun Sarcoma Virus 17 Oncogene Homolog
LAMTOR3	AW556229, late endosomal/lysosomal adaptor, MAPK and MTOR activator 3, LOC100132990, Map, Map2k, MAP2K1IP1, MAPBP, MAPKSP1, Mek binding partner 1, Mp, MP1, PRO0633, Ragulator3
LEF1	3000002B05, AI451430, Lef-, lymphoid enhancer binding factor 1, TCF10, TCF1ALPHA, TCF7L3, TCF/LEF
MAP2K1/2	MEK1/2, MKK1/2
MAP3K5	7420452D20Rik, A, APOPTOSIS SIGNAL REGULATED KINASE 1, AS, ASK, ASK1, M3K5, MAPKKK5, MEKK5, mitogen-activated protein kinase kinase kinase 5, RGD1306565
MAP3K7	CSCF, FMD2, Map3k7 predicted, MEKK7, mitogen-activated protein kinase kinase kinase 7, TAK1, TGF1a, tgf β activated kinase 1
Map3k7-Map3k7ip1-	Tab1-Tab2-Tak1
MAX	AA960152, AI875693, bHLHd, bHLHd4, bHLHd5, bHLHd6, bHLHd7, bHLHd8, Max protein, MYC associated factor X, Myn
Max-Myc	cMyc-MAX, Myc-MAX
MDM2	1700007J15Rik, AA415488, ACTFS, hdm2, HDMX, LSKB, MDM2-A1, MDM2 proto-oncogene, MGC5370, Transformed 3t3 cell double minute 2, transformed mouse 3T3 cell double minute 2
Mkk3/6	MEK3/6, Mkk3/6 (mitogen activated protein kinase kinase 3/6), MKK3/MKK6
MYC	AU016757, bHLHe3, bHLHe39, CMYC, C-MYC-P64, mMyc, MRTL, Myc2, MYCC, MYC proto-oncogene, bHLH transcription factor, myelocytomatosis oncogene, N, Niard, Nird, RNCMYC
NBN	ATV, AT-V1, AT-V2, Nb, NBS, NBS1, NIBRIN, P95
NCSTN	9430068N19Rik, AA727311, APH2, ATAG1874, D1Dau13, D1Dau13e, Kiaa0253, mKIAA0253, Nc, NCT, ni, NICASTRIN
NF1	AW494271, Dsk, Dsk9, E030030H24RIK, LOC646021, Mhdads, Mhdadsk9, Neurofibromatosis 1, NEUROFIBROMIN, neurofibromin 1, Nf-, NF1-GAP, NFNS, VRNF, WSS
NFkB	NF-KAPPA B, NF-κ B, nuclear factor-κ b, transcription factor nuclear factor κ b
NLK	AI194375, LOC100044468, nemo like kinase, RGD1561602
NOTCHIC	9930111A19Rik, AOS5, AOVD1, hN1, lin, lin-12, Mi, Mis6, N, N1, NOTCH, notch receptor 1, Ta, TAN1
p38MAPK	P38, p38 MAP KINASE, P38 MITOGEN-ACTIVATED protein KINASE
Patched	PTC, PTCH
PI3K	1-phosphatidylinositol 3-kinase, 2.7.1.137, ATP:1-phosphatidyl-1D-myo-inositol 3-phosphotransferase, Phosphatidylinositol 3 kinase, phosphatidylinositol 3'-kinase, PI3-kinase, PtdIns 3 Kinase, type III phosphoinositide 3-kinase, type I phosphatidylinositol kinase, Vps34p
PIP2	1,2-diacyl-sn-glycero-3-phospho-(1'-myo-inositol-4',5'-bisphosphate), 1-O-(3-sn-phosphatidyl)-1D-myo-inositol 4,5-bis(dihydrogen phosphate), 1-phosphatidyl-1D-myo-inositol 4,5-bisphosphate, C11H19O19P3R2
PIP3	1-phosphatidyl-1D-myo-inositol 3,4,5-trisphosphate, phosphatidylinositol-3,4,5-trisphosphate, phosphoinositide (3,4,5) P3, PI(3,4,5)P3, PIns(3,4,5)P3, PIP3, PtdIns(3,4,5)P3
PKA	A-Kinase, cAMP-Dependent Protein Kinase, cyclic AMP depended protein kinase, protein KINASE A
PKC	Cnpkc, PKC, Pkc(s), Protein Kinase C
Plcbeta	Phospholipase c beta, Phospholipase C β, PLCB, PLCβ
PMAIP1	APR, N, NOXA, phorbol-12-myristate-13-acetate-induced protein 1
Presenilin	PS, PS1/2, PSEN1/2
PRKDC	AI326420, AU019811, DNA-, DNA-DEPENDENT protein KINASE, DNAPDcs, DNAPK, DNA-PKC, DNA-PKcs, DNPK1, DOX, DOXNPH, dxn, dxnph, HYRC, HYRC1, IMD26, p350, p460, Prkdc predicted, protein kinase, DNA activated, catalytic polypeptide, protein kinase, DNA-activated, catalytic subunit, scid, slip, XRCC, XRCC7
PSENEN	1700023M09RIK, ACNINV2, MDS033, MSTP064, PEN-2, presenilin enhancer gamma secretase subunit, presenilin enhancer, gamma-secretase subunit, presenilin enhancer γ secretase subunit, presenilin enhancer, γ-secretase subunit, RGD1312037
PTCH1	A230106A15RIK, BCNS, mes, NBCCS, patched 1, Pt, PTC, PTC1, PTCH, Ptch2, wi, wig
PTK2	FA, Fad, FADK, FADK 1, FAK, FAK1, FAK related non-kinase, FR, p125FAK, pp125FAK, PPP1R71, protein tyrosine kinase 2, PTK2 protein tyrosine kinase 2, TYROSINE KINASE 2
PTPN11	2700084A17Rik, AW536184, BPTP3, CFC, JMML, METCDS, MGC14433, Noonan syndrome 1, NS1, protein tyrosine phosphatase non-receptor type 11, protein tyrosine phosphatase, non-receptor type 11, PTP, PTP-1D, PTP2C, S, SAP-2, Sh, SH-P, SHP-2, SH-PTP2, SH-PTP3, Src homology protein 2, SYP
RAF1	6430402F14Rik, AA990557, BB129353, CMD1NN, c-R, Cra, CRAF, Craf1, D830050J10Rik, leukaemiA ONCOGENE HOMOLOG1, LEUKEMIA ONCOGENE HOMOLOG1, NS5, Raf-1 proto-oncogene, serine/threonine kinase, v-, v-Raf, v-raf-leukaemia viral oncogene 1, v-raf-leukemia viral oncogene 1
Ral	Ral A/B
RALBP1	DNP-SG ATPase, R, ralA binding protein 1, Ral GAP, Rik, RIP1, RL, RLIP1, RLIP76
RALGDS	Gn, Gnds, Hs.560937, mKIAA1308, Ra, RalGDSB, RalGEF, ral guanine nucleotide dissociation stimulator, Rg, RGDS, RGF
RAPGEF1	4932418O06Rik, C3G, C3G-1, C3G-2, Grf, GRF2, Rap guanine nucleotide exchange factor 1, Rap guanine nucleotide exchange factor (GEF) 1
RAPGEF3	2310016P22Rik, 9330170P05Rik, bcm910, CAMP-GEFI, CGEF1, Epa, EPAC, EPAC1, HSU79275, Rap guanine nucleotide exchange factor 3, Rap guanine nucleotide exchange factor (GEF) 3
Ras-grf	Guanine Nucleotide Releasing
RASA1	CM-AVM, CMAVM1, G, GAP, GAPX, p120-, p120GAP, P120RASGAP, PKWS, RASA, RASGAP, RAS p21 protein activator 1
RASGRP1	CALDAG-GEFI, CALDAG-GEFII, IMD64, RASGRP, RAS guanyl releasing protein 1
Rb-E2Ftranscriptionrepression	Rb1-E2f1, Rb-E2F, Rb-E2F1
RB1	OSRC, p, p105, p105-Rb, p110 RB, p110-RB1, pp105, pp110, PPP1R130, pRb, R, RB, RB-ASSOCIATED, RB transcriptional corepressor 1, Retinoblastome tumor-suppression protein rb
RBL1	AW547426, CP107, LOC683869, p10, p107, PRB1, RB transcriptional corepressor like 1
RBPJ	AI843960, AOS3, CBF-1, csl, Igk, Igkj, IGKJRB, IGKJRB1, Igkrsbp, KBF2, RBP, RBP 2N, RBP-JK, RBP-J kappa, RBP-J κ, RBPSUH, Rbpsuh1, Recombination signal binding, recombination signal binding protein for immunoglobulin kappa J region, recombination signal binding protein for immunoglobulin κ J region, SUH
RHO	GTPase Rho, Rho, Rho Family, RHO-GTPASE, Rho-like Gtpase
SHC1	p52SHC, p6, p66, p66s, P66shc, Sh, SHC, Shc (46 kDa isoform), SHCA, SHC adaptor protein 1, Shc p66 isoform, src homology 2 domain-containing transforming protein C1
Smad1/5/8	SMAD1/5/8
Smad1/5/8-	SMAD 1,4,5,8
Smad2/3-	Smad 2/3/4
SMAD4	AW743858, D18Wsu70, D18Wsu70e, DPC, DPC4, JIP, Madh, MADH4, MYHRS, SMAD family member 4, Smaug1
SMAD6	AOVD2, b2b390C, b2b390Clo, HsT17432, Madh, MADH6, MADH7, SMAD family member 6
SMAD7	CRCS3, Madh, MADH7, MADH8, SMAD family member 7
SMO	bnb, CRJS, E130215L21Rik, FZD11, Gx, PHLS, SMOH, Smoothened, smoothened, frizzled class receptor
SRC	ASV, AW259666, BS27, c-SRC, p60-Src, PP60, Pp60/c-Src, pp60c, pp60c-src, Rous sarcoma oncogene, SRC1, SRC proto-oncogene, non-receptor tyrosine kinase, THC6, TVHUSC
STK36	1700112N14RIK, B930045J24, FU, Fuse, Fused, mKIAA1278, serine/threonine kinase 36, Stk36 (predicted)
SUFU	b2b273C, JBTS32, PRO1280, Su, SUFUH, SUFU negative regulator of hedgehog signaling, SUFUXL
SYNGAP1	Gm1963, MRD5, RASA1, RASA5, Sy, Synaptic Ras-GAP 1, synaptic Ras GTPase activating protein 1, synaptic Ras GTPase activating protein 1 homolog (rat), SYNGAP
TAB2	1110030N06Rik, A530078N03Rik, CHTD2, LOC101928709, Map3k, MAP3K7IP2, mKIAA0733, RP1 111D63, TGF-beta activated kinase 1 (MAP3K7) binding protein 2, TGF-beta activated kinase 1/MAP3K7 binding protein 2, TGF-β activated kinase 1 (MAP3K7) binding protein 2, TGF-β activated kinase 1/MAP3K7 binding protein 2
TBID	2700049M22Rik, AI875481, AU022477, BH3 interacting domain death agonist, cBid, FP497
Tgfbeta	Tgfb, TGF-beta 1, 2, and 3, TGF β, TGF-β 1, 2, and 3, transforming growth factor-β
Tgfbetarecept	TGFbetaR, TGFBR, TGFβR, Tgf β receptor
TGFBR1	AAT5, ACVRLK4, AL, Alk, ALK-5, AU017191, ESK2, ESS1, LDS1, LDS1A, LDS2A, LOC103690035, LOC666236, MSSE, SKR4, Tbet, Tbeta, TbetaR-I, TBR-I, TGFbeta1R1, TGF-beta1 receptor, TGF beta I receptor, Tgf-beta-r1, TGF beta receptor type I, TGFbetaRI, Tgfb receptor I, TGFR-1, TGFβ1R1, TGF β I receptor, TGFβ R1, Tgf -β R1/R4, Tgf-β receptor1, TGF β receptor type I, TGFβRI, TGF β type 1 receptors, Tgf β type i receptor, transforming growth factor beta receptor 1, transforming growth factor, beta receptor 1, transforming growth factor, beta receptor I, transforming growth factor β receptor 1, transforming growth factor, β receptor 1, transforming growth factor, β receptor I, transforming growth factor-β receptor type 1, T β R1
TGFBR2	1110020H15Rik, AAT3, AU042018, DNIIR, FAA3, LDS1B, LDS2, LDS2B, MFS2, RIIC, RIIDN, TAAD2, Tbet, Tbeta, TbetaR-II, TBR, TBR-II, TBRLL, TGFbeta1R2, TGF-beta 2, Tgf beta2 receptor, TGFbeta R2, TGF-beta receptor 2, TGF-beta receptor type 2, TGF beta receptor type II, TGFbeta-RII, Tgfbr2T, TGFB receptor II, Tgfbrii, TGFR-2, TGFβ1R2, TGF-β 2, TGFβ R2, TGF-β receptor 2, TGF-β receptor type 2, TGF β receptor type II, TGFβRII, Tgf-β type ii receptor, transforming growth factor beta receptor 2, transforming growth factor, beta receptor 2, transforming growth factor, beta receptor II, transforming growth factor β receptor 2, transforming growth factor, β receptor 2, transforming growth factor, β receptor II, type 2 TGF-β receptors, T β r2
TP53	bbl, BCC7, bfy, bhy, BMFS5, LFS1, p4, p44, p5, P53, P53 cellular tumour antigen, p53 tumor suppressor, transformation related protein 53, TRP53, tumor protein p53, tumour protein p53
Type I BMP	ALK 3,6, ALK 3/6, BMP2 receptor type1, BMP4 receptor type1, Bmpr1, Bmpri
WNT1	BMND16, Int, INT1, Ol15, sw, swaying, Wg, wingless-type MMTV integration site family, member 1, Wnt-, Wnt family member 1
WNT5A	8030457G12Rik, hWNT5A, LOC102724616, wingless-type MMTV integration site family, member 5A, Wnt-, Wnt family member 5A
ZBTB17	AA589413, Lp-1, Miz, MIZ-1, mZ13, pHZ-67, Zfp10, Zfp100, zinc finger and BTB domain containing 17, ZNF151, ZNF60

Symbol	Synonym(s)
ACTB	Act, actin, Actin beta, actin, beta, Actin β, actin, β, Actx, A-X actin, beta-a, beta-actin, BRWS1, E430023M04Rik, Melanoma x actin, PS1TP5BP1, RBC G-actin, β-a, β-actin, β Ca
AREG	Amphiregulin, AR, AREGB, CRDGF, Mcub, schwannoma-derived growth factor, Sdg, SDGF
CCND1	AI327039, B-CELL CLL/LYMPHOMA 1, bcl-, BCL1, cD1, CycD1, CYCLIN D1, Cyl-, Cyl-1, D11S287E, G1/S-Specific Cyclin D1, PR, PRAD1, U21B31
CCNE1	AW538188, CCNE, CycE1, CYCLE, cyclin E, Cyclin E1, pCCNE1
CDKN1A	CAP, CAP20, CDK, CDKI, Cdkn, CDKN1, CDKNA1, CI, CIP1, cyclin-dependent kinase inhibitor 1A, cyclin-dependent kinase inhibitor 1A (P21), mda, MDA-6, P2, P21, p21C, p21Cip, p21CIP1, p21W, p21WAF, p21Waf1, Pzl Cyclin-Dependent Kinase Inhibitor, SD, SDI1, UV96, Waf, WAF1
CDKN2A	A, Arf, ARF-INK4a, CDK4I, CDKN2, CMM2, CYCLIN-DEPENDENT KINASE INHIBITOR 2A, INK4, INK4A, INK4a-ARF, Ink4a/Arf, MLM, MTS, MTS-1, p1, p14ARF/ p16INK4a, p16, p16/ARF, p16Cdkn2a, p16I, p16 INK4, p16/ INK4a, P19, p19ARF, Pct, PCTR1, TP16
CEBPB	Agp/eb, ANF-1, ANF-2, CCAAT enhancer binding protein beta, CCAAT/enhancer binding protein beta, CCAAT/enhancer binding protein (C/EBP), beta, CCAAT/enhancer binding protein (C/EBP), β, CCAAT enhancer-binding protein β, CCAAT/enhancer binding protein β, C/EBPbe, C/EBP-beta, C/Ebp Beta-Lip, C/EBP-β, CEBP-β, C/Ebp β-Lip, CR, CRP2, IL-6, IL-6DBP, NF-, NF-IL6, NF-M, TCF5
E2F1	E2f, E2F transcription factor 1, mKIAA4009, RBAP1, RBBP3, RBP3, Tg(Wnt1-cre)2Sor
E2F2	9230110J10, E2F transcription factor 2
E2F3	E2F transcription factor 3, LOC100361421, LOC691420, RGD1561600
EP400	1700020J09Rik, AU023439, CAGH32, E1A binding protein p400, mDo, mDomino, mKIAA1498, NHCP p400, p40, P400, TNRC12
FOXM1	AA408308, AW554517, BB238854, D1Mgi5, D1Mgi56, Fkh16, FKHL16, FOCM1, forkhead box M1, FOXM1B, HFH-11, HFH-11B, HNF-3, INS-1, MPHOSPH2, Mpm, MPM2, MPP-2, PIG29, Trid, TRIDENT, W, WIN
HELLS	AI323785, E130115I21RIK, helicase, lymphoid specific, Helis helicase, ICF4, L, LSH, Ly, Lysh, Nbla10143, P, PASG, SMARCA6, YFK8
LIN9	2700022J23Rik, BARA, BARPsv, lin-9 DREAM MuvB core complex component, lin-9 homolog (C. elegans), LOC360888, LOC690072, mLin-9, TGS, TGS1, TGS2
MXI1	bHLHc11, Gm10197, LOC100360467, LOC100360898, MAD2, MAXD2, Max inter 1, MAX interactor 1, dimerization protein, MX11, MXD2, MXI, MXI-WR
MYC	AU016757, bHLHe3, bHLHe39, C-MYC-P64, CMYC, mMyc, MRTL, Myc2, MYC proto-oncogene, bHLH transcription factor, MYCC, myelocytomatosis oncogene, N, Niard, Nird, RNCMYC
RABL6	B230208H17Rik, C9orf86, FLJ10101, PARF, pp8875, RAB, member RAS oncogene family-like 6, Rbe, Rbel, RBEL1, Rbel1a, Rbel1b, RGD1307615
RB1	OSRC, p, p105, p105-Rb, p110 RB, p110-RB1, pp105, pp110, PPP1R130, pRb, R, RB, RB-ASSOCIATED, RB transcriptional corepressor 1, Retinoblastome tumor-suppression protein rb
RBL1	AW547426, CP107, LOC683869, p10, p107, PRB1, RB transcriptional corepressor like 1
RBL2	p13, P130, PRB2, Rb, Rb2, RB-LIKE protein 2, RBR-2, RB transcriptional corepressor like 2
TBX2	T-box 2, T-box transcription factor 2, THROMBOXANE B2, VETD
TFDP1	DILC, Dp, DP-1, Drtf, DRTF1, TB2/DP1, transcription factor Dp-1
TP53	bbl, BCC7, bfy, bhy, BMFS5, LFS1, p4, p44, p5, P53, P53 cellular tumour antigen, p53 tumor suppressor, transformation related protein 53, TRP53, tumor protein p53, tumour protein p53

Figure S59. Legend of Figure 8
